# Improving Empiric Antibiotic Selection for Patients With Cancer Hospitalized With Infection: Secondary Analysis of the INSPIRE Cluster Randomized Trials

**DOI:** 10.1001/jamanetworkopen.2026.16611

**Published:** 2026-06-10

**Authors:** Shruti K. Gohil, Taliser R. Avery, Ken Kleinman, Edward Septimus, Amarah Mauricio, Kenneth E. Sands, Neha Varma, Selsebil Sljivo, Kaleb Roemer, William S. Cooper, Russell E. Poland, Robert A. Weinstein, Samir M. Fakhry, Jeffrey Guy, Julia Moody, Micaela H. Coady, Kim N. Smith-Sells, Mary K. Hayden, David W. Kubiak, Chenette Burks, Richard Platt, Susan S. Huang, Meghan A. Baker

**Affiliations:** 1 Division of Infectious Diseases, University of California, Irvine School of Medicine, Irvine; 2 Department of Population Medicine, Harvard Pilgrim Healthcare Institute, Harvard Medical School, Boston, Massachusetts; 3 Biostatistics and Epidemiology, University of Massachusetts, Amherst; 4 HCA Healthcare, Nashville, Tennessee; 5 Department of Population Medicine, Harvard Pilgrim Health Care Institute, Boston, Massachusetts; 6 Rush University Medical Center, Cook County Health, Chicago, Illinois; 7 Center for Trauma and Acute Care Surgery Research, Clinical Services Group, HCA Healthcare, Nashville, Tennessee; 8 Thomas F. Frist, Jr. College of Medicine, Belmont University, Nashville, Tennessee; 9 Rush University Medical Center, Chicago, Illinois; 10 Brigham and Women’s Hospital, Boston, Massachusetts

## Abstract

**Importance:**

Patients with cancer are routinely prescribed extended-spectrum antibiotics despite overall low multidrug-resistant organism (MDRO) prevalence. Evidence for effective strategies to reduce antibiotic overuse in this population is limited.

**Objective:**

To evaluate the association of computerized provider order entry (CPOE) prompts providing patient- and pathogen-specific MDRO risk estimates with empiric extended-spectrum antibiotic use in patients with cancer.

**Design, Setting, and Participants:**

This secondary analysis of the 4 Intelligent Stewardship Prompts to Improve Real-Time Empiric Antibiotic Selection (INSPIRE) cluster randomized clinical trials identified non–critically ill hospitalized adults (aged ≥18 years) with discharge diagnosis codes for hematologic or solid organ malignant tumors in the INSPIRE pneumonia, urinary tract infection (UTI), abdominal, and skin and soft tissue infection (SSTI) trials.

**Intervention:**

Each trial evaluated the effect of CPOE prompts that used real-time patient-specific electronic health record data to estimate MDRO infection risk for patients prescribed extended-spectrum antibiotics during the first 3 hospital days; the prompt recommended standard-spectrum antibiotics when the risk of antibiotic-resistant infection was less than 10%.

**Main Outcomes and Measures:**

Extended-spectrum antibiotic days of therapy were evaluated using as-randomized, adjusted difference-in-difference analyses with generalized linear mixed-effects models and clustering by patient, hospital, and period. Days to intensive care unit transfer, hospital length of stay, hospital readmissions, and in-hospital mortality were also assessed.

**Results:**

In all trials, 36 861 patients (mean [SD] age, 69.0 [13.6] years; 19 076 [52%] female), including 18 272 baseline and 18 589 intervention patients, had cancer. Extended-spectrum antibiotic days of therapy decreased by 27% (rate ratio [RR], 0.73; 95% CI, 0.67-0.80; *P* < .001) in the pneumonia trial, 24% (RR, 0.76; 95% CI, 0.68-0.84; *P* < .001) in the UTI trial, 17% (RR, 0.83; 95% CI, 0.74-0.92; *P* < .001) in the SSTI trial, and 24% (RR, 0.76; 95% CI, 0.69-0.84; *P* < .001) in the abdominal infection trial. Pre-post changes in hospital length of stay, intensive care unit transfers, readmissions, and in-hospital mortality were similar in the 2 groups.

**Conclusions and Relevance:**

In this secondary analysis of randomized clinical trials, an antibiotic stewardship bundle that included CPOE prompts recommending standard-spectrum antibiotics for patients at low risk for antimicrobial-resistant infections was associated with reduced extended-spectrum antibiotic use in non–critically ill patients with cancer who were hospitalized with community-acquired pneumonia, UTI, SSTI, or abdominal infection, without observed differences in safety outcomes. The findings support scalable, low-burden strategies to improve antimicrobial use in patients with cancer, a population with limited evidence to guide stewardship.

**Trial Registration:**

ClinicalTrials.gov Identifiers: NCT05423756, NCT05423743, NCT03697070, NCT03697096

## Key Points

**Question:**

Can computerized provider order entry (CPOE) prompts with patient-specific risk estimates for multidrug-resistant organisms (MDROs) safely reduce the use of empiric extended-spectrum antibiotics in patients with cancer hospitalized with infection?

**Findings:**

This secondary analysis of 4 cluster randomized clinical trials evaluating CPOE prompts (plus education and feedback) promoting standard-spectrum antibiotics in patients with low MDRO infection risk found that empiric extended-spectrum antibiotic days of therapy decreased by 17% to 27%, without evidence of inferiority in intensive care unit transfers or length of stay.

**Meaning:**

This study’s results suggest that CPOE-generated recommendations based on patient-specific risk for MDRO-associated pneumonia, urinary tract infections, skin or soft tissue infection, and abdominal infections may substantially and safely reduce the use of empiric extended-spectrum antibiotics in patients with cancer hospitalized for infection.

## Teaser

This secondary analysis of the 4 Intelligent Stewardship Prompts to Improve Real-Time Empiric Antibiotic Selection (INSPRE) cluster randomized clinical trials evaluates the association of computerized provider order entry prompts with clinicians’ antibiotic prescribing for patients with cancer who were hospitalized for infection.

## Introduction

Clinicians often initiate empiric extended-spectrum antibiotics for patients hospitalized for infection, although most will be safely treated with standard-spectrum antibiotics.^1,2,3,4,5,6^ We recently demonstrated that an antibiotic stewardship bundle that included real-time, patient-specific computerized provider order entry (CPOE) prompts recommending standard-spectrum therapy in patients with low risk of antibiotic-resistant infection substantially reduced extended-spectrum antibiotic selection in non–critically ill patients hospitalized for pneumonia, urinary tract infection (UTI), skin and soft tissue infection (SSTI), or abdominal infection.^7,8,9,10^

These trials included patients with cancer, a subset of special interest because clinicians may be less likely to adopt interventions promoting empiric standard-spectrum antibiotic use for this population.^11,12^ The propensity to select extended-spectrum antibiotics in patients with weakened immunity may be driven by concerns that such patients may not manifest symptoms that reflect the severity of their infection, which heightens diagnostic uncertainty and complicates timely diagnosis.^13,14^ Long-term antibacterial prophylaxis, use of devices such as central venous catheters, and frequent health care visits raise concern for higher risk of colonization or infection with resistant organisms in patients with cancer.^15,16,17,18,19^

Extended-spectrum antibiotics are often used in patients with cancer and yet judiciousness is critical in this population.^19,20^ These patients may be more susceptible to the known adverse effects of extended-spectrum antibiotics. However, antibiotic stewardship strategies to minimize exposure to extended-spectrum antibiotics have largely excluded patients who are immunocompromised.^19^ When pursued, efforts have primarily focused on de-escalation rather than optimizing initial antibiotic selection, missing the opportunity to prevent unnecessary exposures to extended-spectrum antibiotics.

The original Intelligent Stewardship Prompts to Improve Real-Time Empiric Antibiotic Selection (INSPIRE) trials evaluated the effectiveness and safety of interventions to reduce the use of extended-spectrum antibiotics and did not exclude patients with cancer. In this secondary analysis, we evaluated the association of the intervention with clinicians’ antibiotic use for patients with cancer.

## Methods

### Study Design and Intervention

The INSPIRE trials were 4 cluster-randomized trials evaluating antibiotic selection in non–critically ill adults hospitalized with pneumonia, UTI, SSTI, and abdominal infection (eFigures 2-5 in Supplement 1).^7,8,9,10^ The pneumonia and UTI trials (implemented simultaneously) involved 59 hospitals with an 18-month baseline and a 15-month intervention period; the abdominal infection and SSTI trials (implemented simultaneously) involved 92 hospitals with 12-month baseline and intervention periods. The analysis reported in this article was approved by the Harvard Pilgrim Health Care institute institutional review board with waiver of informed consent because the study met criteria for minimal risk. The original study followed the Consolidated Standards of Reporting Trials (CONSORT) reporting guideline. (See Supplement 2 for the 4 study protocols and statistical analysis plans.)

Each trial compared rates of extended-spectrum antibiotic days of therapy during the first 3 hospital days (empiric period) in an emergency department or non-ICU location in hospitals randomized to either (1) routine stewardship, where clinicians continued usual antibiotic prescribing practices with coaching calls emphasizing national guidance, or (2) the CPOE bundle stewardship intervention, where clinicians ordering antibiotics received prompts recommending standard-spectrum instead of extended-spectrum antibiotics if they attempted to order an extended-spectrum antibiotic for any patient with a less than 10% risk of multidrug-resistant organisms (MDRO) infection based on information in the electronic health record as well as education and personalized feedback on their prescribing habits. Specifically, there was no exclusion for patients with hematologic or solid organ malignant neoplasms, stem cell transplant recipients, or other types of cancers. Documentation of indication was mandatory for all antibiotic orders and encompassed all infection types. Selection of either sepsis or empiric indication further required specification of the predominant suspected organ system, if known.

The CPOE algorithm and prompt assessed the risk of the MDRO targeted by the prescribed extended-spectrum antibiotic, which included antipseudomonal antibiotics (eTable 1 in Supplement 1). Automated assessment of each patient’s risk of MDRO infection occurred for any patient for whom an extended-spectrum antibiotic was ordered in an emergency department or non-ICU location for pneumonia, UTI, SSTI, or abdominal infection within 72 hours of admission. Documentation of indication was mandatory for all antibiotic orders and was broadly defined to include all infection subtypes. If the patient’s estimated absolute MDRO risk was low (<10%), based on information in the electronic health record, a prompt was triggered recommending standard-spectrum antibiotics. Clinicians were encouraged to select appropriate antibiotics based on national guidelines and clinical judgment and were allowed to override the prompt for any reason, including neutropenia. The CPOE algorithm and prompt were antibiotic and infection syndrome specific. For example, if cefepime was ordered for an abdominal infection, the prompt assessed whether the risk of *Pseudomonas* abdominal infection was less than 10%; if carbapenems were ordered, the risk of abdominal infection with extended-spectrum β-lactamase–producing Enterobacterales (ESBL) or resistant *Pseudomonas* was assessed; and if vancomycin was ordered, risk for abdominal infection with methicillin-resistant *Staphylococcus aureus* (MRSA) was assessed.

Estimated absolute MDRO risk was obtained from classification and regression tree models run separately for each MDRO for each infection syndrome, using datasets that collectively included 927 008 patients. Models assessed more than 60 variables, including demographics, health care use, antibiotic exposures, history or microbiologic evidence of MDROs (any body site), comorbidities, admission laboratory values, and each hospital’s frequency of positive MDRO blood or infection-specific source culture results.

Details on stewardship activities, clinical workflow and prompts, protocols, statistical analyses, and model methods and validation were published in the supplemental materials of the original publications for each trial.^7,8,9,10^ Routine stewardship activities for all patients, including the immunocompromised subset, included requiring an indication for prescribed antibiotics and prospective evaluation to de-escalate antibiotics based on microbiologic test results and national guidelines for antibiotic selection.

### Study Cohort Definition

We retrospectively identified a subset of patients with cancer who had been included in the 4 INSPIRE trials.^21^ Patients with cancer were identified using discharge diagnosis codes for hematologic and solid organ malignant tumors (eTable 2 in Supplement 1). A further subgroup of patients with cancer who had severe neutropenia were defined as those with an absolute neutrophil count of 500/μL or less (to convert to ×10^9^/L, multiply by 0.001) or a white blood cell count less than 1000 /μL (to convert to ×10^9^/L, multiply by 0.001), using the minimum absolute neutrophil count and white blood cell count measured within the first 3 hospital days.

### Data Collection

Trial data have been previously described.^7,8,9,10^ Patient demographics (including self-reported race and ethnicity), unit location, prior hospitalizations and antibiotic use at the same hospital, comorbidities, laboratory data, antibiotics given during admission, hospital readmission, and in-hospital mortality were collected from the HCA Healthcare centralized data warehouse.^7,8,9,10^ Race and ethnicity were included as collected in the electronic health record to address generalizability. MDRO history was obtained from any available microbiology laboratory results from any body site. MDROs included MRSA, vancomycin-resistant *Enterococci*, ESBL, multidrug-resistant *Pseudomonas*, multidrug-resistant *Acinetobacter*, and carbapenem-resistant Enterobacterales. Determination of whether the trial-specific infections (ie, pneumonia, UTI, SSTI, and abdominal infections) were due to MDROs involved assessing cultures from blood and/or site-specific sources with positive results (eg, respiratory cultures for the pneumonia trial, urinary cultures for the UTI trial, skin and soft tissue cultures for the SSTI trial, and intra-abdominal cultures for the abdominal infection trial) collected within 3 days of hospital admission.

### Study Outcomes

Outcomes were as specified in the trials. The primary outcome was extended-spectrum antibiotic days of therapy, the US national standard for evaluating inpatient antibiotic use. This was assessed in the empiric period (first 3 calendar days of hospitalization), calculated as the summed number of different extended-spectrum antibiotics received per patient each calendar day in a non-ICU location, beginning at admission. For example, 2 different extended-spectrum antibiotics administered at least once during each of the first 3 days would yield 6 days of extended-spectrum therapy. Secondary outcomes included days of therapy of the subsets of vancomycin and antipseudomonal antibiotics. The following safety outcomes were also assessed: (1) ICU transfers (patients eligible if length of stay was between day 3 and day 14); (2) days to ICU transfer, defined as days from admission until ICU transfer; (3) hospital length of stay (days from admission to date of discharge among those discharged alive up to hospital day 14); (4) frequency of hospital readmissions within 30 days of discharge; and (5) frequency of mortality at any time during hospitalization.

### Statistical Analysis

Analyses were conducted separately for each of the 4 infection trials. Primary and secondary outcomes were evaluated using generalized linear mixed-effects models assessing differences in empiric extended-spectrum days of therapy between intervention and baseline periods across the groups (difference in differences). Adjusted analyses accounted for age, sex, race and ethnicity, Medicaid insurance, antibiotic or nursing home exposure in the last year, mean Elixhauser comorbidity count, history of MDRO, cancer type (hematologic vs solid tumor), and severe neutropenia (absolute neutrophil count ≤500/μL or white blood cell count <1000/μL).^22^ Models for abdominal infection were additionally adjusted for history of abdominal surgery in the last year. Random effects accounted for clustering within patients, hospitals, and periods within hospitals. The unit of analysis was patient admission (patients with multiple admissions contributed all admissions). The primary outcome was assessed with 2-tailed significance at α = .05, and the 2 secondary outcomes were each assessed with 2-tailed significance at α = .025 to account for multiple comparisons. Subgroup analyses included repeating the above analysis of primary and secondary outcomes within the subpopulations of patients with cancer with and without severe neutropenia. All analyses were performed using SAS, version 9.4 (SAS Institute) or R, version 4.1.3 (R Foundation for Statistical Computing).

## Results

### Patient Characteristics

Of the 580 446 patients included in the 4 INSPIRE trials, 36 861 (6%; mean [SD] age, 69.0 [13.6] years; 19 076 [52%] female, 17 675 [48%] male, and 14 [<0.01%] unknown gender) were identified as having a diagnosis of cancer, including 9923 (9%) of those hospitalized with pneumonia, 10 074 (7%) with UTI, 12 677 (6%) with abdominal infection, and 4187 (4%) with SSTI (Table 1). Among patients meeting the cohort definition for this study, most had solid malignant tumors (810 [77%] to 2822 [89%] across the trials). Including the baseline and intervention periods, the collective CPOE bundle intervention groups had 17 835 patients, and the routine care groups had 19 026 patients.

**Table 1.** Characteristics of Patients With Cancer in the INSPIRE Trials During Baseline and Intervention Periods

Characteristic	No. (%)^a^															
	Pneumonia				Urinary tract infection				Skin and soft tissue infection				Abdominal infection			
	Baseline (12 mo)		Intervention (12 mo)		Baseline (12 mo)		Intervention (12 mo)		Baseline (18 mo)		Intervention (15 mo)		Baseline (18 mo)		Intervention (15 mo)	
	Routine	CPOE	Routine	CPOE	Routine	CPOE	Routine	CPOE	Routine	CPOE	Routine	CPOE	Routine	CPOE	Routine	CPOE
Total No. of patients	2511	2554	2427	2431	2756	2754	2296	2268	1049	943	1217	978	2953	2752	3817	3155
Patients with solid organ tumors																
Metastatic	1158 (46)	1138 (45)	1118 (46)	1084 (45)	1163 (42)	1207 (44)	948 (41)	952 (42)	357 (34)	295 (31)	394 (32)	325 (33)	1206 (41)	1106 (40)	1444 (38)	1268 (40)
No metastasis	921 (37)	957 (38)	899 (37)	882 (36)	1190 (43)	1113 (40)	1053 (46)	963 (43)	453 (43)	425 (45)	598 (49)	475 (49)	1295 (44)	1238 (45)	1832 (48)	921 (37)
Patients with hematologic malignant tumors																
Lymphoma	291 (12)	288 (11)	300 (12)	285 (12)	274 (10)	316 (11)	232 (10)	227 (10)	145 (14)	139 (15)	121 (10)	99 (10)	315 (11)	280 (10)	344 (9)	229 (7)
Leukemia	217 (9)	245 (10)	202 (8)	279 (11)	192 (7)	182 (7)	136 (6)	207 (9)	135 (13)	126 (13)	145 (12)	107 (11)	237 (8)	204 (7)	305 (8)	178 (6)
Age, mean (SD), y	69 (13)	70 (13)	69 (13)	69 (13)	71 (13)	71 (14)	71 (13)	71 (13)	66 (15)	65 (14)	67 (14)	66 (14)	68 (14)	68 (14)	68 (14)	68 (14)
Age group, y																
18-44	97 (4)	103 (4)	108 (4)	101 (4)	103 (4)	111 (4)	81 (4)	83 (4)	88 (8)	72 (8)	95 (8)	82 (8)	169 (6)	171 (6)	238 (6)	209 (7)
45-54	210 (8)	186 (7)	189 (8)	175 (7)	201 (7)	218 (8)	160 (7)	150 (7)	127 (12)	122 (13)	107 (9)	112 (11)	284 (10)	252 (9)	348 (9)	314 (10)
55-64	526 (21)	512 (20)	477 (20)	508 (21)	457 (17)	488 (18)	384 (17)	408 (18)	245 (23)	226 (24)	289 (24)	231 (24)	638 (22)	622 (23)	773 (20)	645 (20)
65-74	792 (32)	749 (29)	753 (31)	746 (31)	780 (28)	769 (28)	671 (29)	629 (28)	258 (25)	256 (27)	345 (28)	261 (27)	819 (28)	814 (30)	1104 (29)	905 (29)
75-84	597 (24)	682 (27)	647 (27)	657 (27)	760 (28)	766 (28)	663 (29)	645 (28)	230 (22)	193 (20)	257 (21)	213 (22)	701 (24)	628 (23)	980 (26)	783 (25)
≥85	289 (12)	322 (13)	253 (10)	244 (10)	455 (17)	402 (15)	337 (15)	353 (16)	101 (10)	74 (8)	124 (10)	79 (8)	342 (12)	265 (10)	374 (10)	299 (9)
Sex																
Male	1397 (56)	1409 (55)	1242 (51)	1323 (54)	1155 (42)	1167 (42)	874 (38)	958 (42)	529 (50)	477 (51)	675 (55)	534 (55)	1382 (47)	1238 (45)	1840 (48)	1475 (47)
Female	1104 (44)	1134 (44)	1174 (48)	1103 (45)	1587 (58)	1580 (57)	1413 (62)	1307 (58)	511 (49)	459 (49)	542 (45)	444 (45)	1559 (53)	1502 (55)	1977 (52)	1680 (53)
Unknown	10 (0.4)	11 (0.4)	11 (0.5)	5 (0.2)	14 (0.5)	7 (0.3)	9 (0.4)	3 (0.1)	9 (0.9)	7 (0.7)	0	0	12 (0.4)	12 (0.4)	0	0
Race and ethnicity																
Black	289 (12)	289 (11)	299 (12)	285 (12)	314 (11)	370 (13)	281 (12)	277 (12)	82 (8)	81 (9)	116 (10)	111 (11)	287 (10)	318 (12)	376 (10)	367 (12)
Hispanic or Latino	291 (12)	453 (18)	305 (13)	389 (16)	387 (14)	498 (18)	339 (15)	396 (17)	140 (13)	147 (16)	164 (13)	172 (18)	503 (17)	481 (17)	702 (18)	670 (21)
White	1890 (75)	1978 (77)	1768 (73)	1883 (77)	2091 (76)	2079 (75)	1689 (74)	1777 (78)	868 (83)	743 (79)	957 (79)	741 (76)	2319 (79)	2090 (76)	2881 (75)	2296 (73)
Unknown	221 (9)	107 (4)	247 (10)	112 (5)	261 (9)	133 (5)	261 (11)	109 (5)	60 (6)	83 (9)	105 (9)	116 (12)	160 (5)	248 (9)	376 (10)	416 (13)
Other^b^	111 (4)	180 (7)	113 (5)	151 (6)	90 (3)	172 (6)	65 (3)	105 (5)	39 (4)	36 (4)	39 (3)	10 (1)	187 (6)	96 (3)	184 (5)	76 (2)
Insurance type																
Medicare	1770 (70)	1837 (72)	1762 (73)	1750 (72)	2059 (75)	2025 (74)	1719 (75)	1700 (75)	658 (63)	600 (64)	767 (63)	588 (60)	1940 (66)	1820 (66)	2445 (64)	2031 (64)
Commercial	312 (12)	333 (13)	302 (12)	310 (13)	97 (4)	110 (4)	258 (11)	245 (11)	192 (18)	163 (17)	198 (16)	161 (16)	133 (5)	110 (4)	607 (16)	490 (16)
Other (eg, self-pay, free care)	176 (7)	127 (5)	150 (6)	128 (5)	361 (13)	366 (13)	144 (6)	129 (6)	79 (8)	74 (8)	144 (12)	119 (12)	619 (21)	574 (21)	441 (12)	358 (11)
Medicaid	253 (10)	257 (10)	213 (9)	243 (10)	239 (9)	253 (9)	175 (8)	194 (9)	120 (11)	106 (11)	108 (9)	110 (11)	261 (9)	248 (9)	324 (8)	276 (9)
Antibiotic and health care exposures in year before admission^c^																
Emergency department visit	1428 (57)	1513 (59)	1432 (59)	1420 (58)	1648 (60)	1679 (61)	1370 (60)	1369 (60)	591 (56)	534 (57)	671 (55)	528 (54)	1552 (53)	1485 (54)	1878 (49)	1589 (50)
Hospitalization	1268 (50)	1310 (51)	1241 (51)	1219 (50)	1410 (51)	1438 (52)	1174 (51)	1170 (52)	528 (50)	506 (54)	646 (53)	478 (49)	1391 (47)	1333 (48)	1738 (46)	1411 (45)
Antibiotics	1023 (41)	1047 (41)	1040 (43)	1036 (43)	1144 (42)	1146 (42)	1006 (44)	1003 (44)	443 (42)	422 (45)	570 (47)	407 (42)	1155 (39)	1094 (40)	1407 (37)	1173 (37)
Nursing home stay	249 (10)	287 (11)	261 (11)	265 (11)	439 (16)	418 (15)	340 (15)	335 (15)	106 (10)	118 (13)	114 (9)	92 (9)	276 (9)	310 (11)	263 (7)	235 (7)
Time to first antibiotics (current admission), median (IQR), h^d^	2 (1-4)	2 (1-4)	1 (1-3)	1 (1-3)	3 (2-6)	3 (2-6)	2 (1-5)	2 (1-5)	2 (1-3)	2 (0.5-3)	2 (1-4)	2 (1-5)	2 (1-5)	3 (1-5)	3 (1-7)	3 (1-7)
Time to positive MDRO culture result, if positive, mean (SD), d^e^	4 (1)	5 (3)	5 (2)	5 (3)	5 (1)	5 (5)	5 (3)	4 (2)	4 (2)	4 (1)	5 (1)	5 (4)	5 (6)	5 (3)	5 (2)	5 (3)
History of pathogen requiring extended-spectrum antibiotics^f^																
Any MDRO	255 (10)	226 (9)	284 (12)	275 (11)	414 (15)	459 (16	396 (17)	382 (16)	205 (20)	206 (22)	233 (19)	168 (17)	392 (13)	373 (14)	437 (11)	395 (13)
MRSA	133 (5)	103 (4)	140 (6)	122 (5)	152 (6)	155 (6)	132 (6)	145 (6)	129 (12)	142 (15)	148 (12)	111 (11)	183 (6)	185 (7)	202 (5)	166 (5)
VRE	10 (0.4)	22 (1)	13 (1)	21 (1)	36 (1)	50 (2)	33 (1)	45 (2)	16 (2)	21 (2)	15 (1)	9 (1)	24 (1)	39 (1)	39 (1)	33 (1)
* Pseudomonas*	85 (3)	91 (4)	100 (4)	113 (5)	152 (6)	183 (7)	143 (6)	145 (6)	52 (5)	60 (6)	80 (7)	52 (5)	106 (4)	99 (4)	128 (3)	132 (4)
ESBL^g^	81 (3)	75 (3)	96 (4)	78 (3)	184 (7)	221 (8)	207 (9)	167 (7)	44 (4)	49 (5)	75 (6)	42 (4)	177 (6)	175 (6)	203 (5)	168 (5)
Carbapenem-resistant gram-negative bacteria^h^	16 (1)	13 (1)	28 (1)	10 (0.4)	25 (1)	36 (1)	33 (1)	19 (1)	8 (1)	7 (1)	19 (2)	7 (1)	15 (1)	24 (1)	16 (0.4)	10 (0.3)
Selected Elixhauser comorbidities^i^																
Hypertension	1595 (64)	1671 (65)	1729 (71)	1687 (69)	1872 (68)	1841 (67)	1703 (74)	1681 (74)	704 (67)	630 (67)	889 (73)	730 (75)	1981 (67)	1854 (67)	2710 (71)	2251 (71)
Diabetes	712 (28)	806 (32)	756 (31)	758 (31)	891 (32)	985 (36)	791 (34)	819 (36)	362 (35)	360 (38)	439 (36)	384 (39)	945 (32)	926 (34)	1259 (33)	1106 (35)
Chronic pulmonary disease	1231 (49)	1137 (45)	1204 (50)	1141 (47)	688 (25)	695 (25)	620 (27)	620 (27)	270 (26)	228 (24)	283 (23)	257 (26)	703 (24)	707 (26)	812 (21)	738 (23)
Neurologic disorders	598 (24)	588 (23)	590 (24)	647 (27)	835 (30)	844 (31)	784 (34)	796 (35)	221 (21)	172 (18)	273 (22)	260 (27)	646 (22)	565 (21)	859 (23)	743 (24)
Obesity	299 (12)	260 (10)	349 (14)	282 (12)	363 (13)	395 (14)	349 (15)	328 (14)	231 (22)	209 (22)	234 (19)	245 (25)	472 (16)	425 (15)	518 (14)	518 (16)
Anemias	1096 (44)	1070 (42)	1186 (49)	1112 (46)	1238 (45)	1183 (43)	1072 (47)	1033 (46)	470 (45)	412 (44)	587 (48)	413 (42)	1391 (47)	1197 (43)	1814 (48)	1387 (44)
Renal disease	493 (20)	511 (20)	580 (24)	540 (22)	696 (25)	695 (25)	602 (26)	631 (28)	276 (26)	215 (23)	250 (21)	231 (24)	669 (23)	596 (22)	759 (20)	647 (21)
Heart failure	572 (23)	550 (22)	618 (25)	616 (25)	492 (18)	436 (16)	448 (20)	441 (19)	202 (19)	175 (19)	230 (19)	216 (22)	428 (14)	398 (14)	572 (15)	466 (15)
Liver disease	155 (6)	129 (5)	206 (8)	197 (8)	210 (8)	163 (6)	197 (9)	241 (11)	75 (7)	74 (8)	135 (11)	119 (12)	353 (12)	336 (12)	678 (18)	523 (17)
Thyroid disorders	438 (17)	418 (16)	499 (21)	444 (18)	540 (20)	531 (19)	496 (22)	443 (20)	199 (19)	171 (18)	237 (19)	202 (21)	545 (18)	515 (19)	704 (18)	543 (17)
Alcohol and drug abuse	132 (5)	119 (5)	134 (6)	133 (5)	110 (4)	102 (4)	93 (4)	93 (4)	75 (7)	54 (6)	87 (7)	79 (8)	163 (6)	154 (6)	212 (6)	182 (6)
Coagulopathy	627 (25)	648 (25)	580 (24)	615 (25)	587 (21)	572 (21)	494 (22)	524 (23)	250 (24)	233 (25)	294 (24)	224 (23)	709 (24)	620 (23)	1017 (27)	720 (23)
Elixhauser count, median (IQR)^j^	5 (3-6)	5 (3-6)	5 (4-7)	5 (4-7)	5 (3-6)	5 (3-6)	5 (4-7)	5 (4-7)	5 (3-6)	5 (3-6)	5 (3-6)	5 (4-7)	5 (3-6)	5 (3-6)	5 (3-6)	5 (3-6)
Neutropenia^k^^,^^l^																
POA diagnostic code	123 (5)	145 (6)	144 (6)	135 (6)	96 (4)	124 (5)	96 (4)	99(4)	55 (5)	55 (6)	86 (7)	61 (6)	163 (6)	186 (7)	288 (8)	204 (7)
Absolute neutrophil count ≤500/μL or white blood cell count <1000/μL	93 (4)	125 (5)	106 (4)	133 (6)	59 (2)	80 (3)	73 (3)	88 (4)	56 (5)	55 (6)	61 (5)	58 (6)	151 (5)	133 (5)	222 (6)	146 (5)
Minimum white blood cell count^l^																
Median (IQR)	9 (5-12)	8 (5-13)	8 (5-12)	8 (5-12)	8 (5-11)	8 (5-11)	8 (5-11)	8 (5-11)	7 (5-11)	8 (5-11)	8 (5-11)	8 (5-12)	7 (5-11)	7 (5-11)	8 (5-12)	8 (5-11)
<1000/μL	85 (3)	104 (4)	85 (4)	104 (4)	50 (2)	68 (2)	58 (3)	68 (3)	45 (4)	46 (5)	33 (3)	38 (4)	129 (4)	116 (4)	155 (4)	102 (3)
Minimum absolute neutrophil count^l^																
Median (IQR)	7 (4-11)	6 (4-10)	7 (4-13)	7 (4-11)	6 (4-9)	6 (3-9)	7 (4-12)	6 (4-10)	5 (3-8)	6 (2-10)	6 (3-11	6 (3-10)	5 (3-9)	5 (3-10)	6 (3-12)	6 (4-11)
≤500/μL	28 (1)	45 (2)	48 (2)	63 (3)	14 (0.5)	29 (1)	32 (1)	46 (2)	29 (3)	23 (2)	54 (4)	52 (5)	52 (2)	41 (2)	182 (5)	118 (4)
>500/μL and ≤1000/μL	16 (1)	26 (1)	30 (1)	31 (1)	12 (0.4)	22 (0.8)	15 (0.7)	26 (1)	12 (1)	6 (0.6)	23 (2)	18 (2)	29 (1)	23 (0.8)	79 (2)	64 (2)
>1000/μL and ≤1500/μL	10 (0.4)	28 (1)	27 (1)	38 (2)	22 (0.8)	30 (1)	23 (1)	31 (1)	11 (1)	9 (1)	20 (2)	20 (2)	28 (0.9)	21 (0.8)	93 (2)	71 (2)
≥1500/μL	573 (23)	778 (31)	1045 (43)	1183 (49)	625 (23)	918 (33)	994 (43)	1090 (48)	253 (24)	185 (20)	1010 (83)	793 (81)	780 (26)	542 (20)	3101 (81)	2537 (80)
Missing	1884 (75)	1677 (66)	1277 (53)	1116 (46)	2083 (76)	1755 (64)	1232 (54)	1075 (47)	744 (71)	720 (76)	110 (9)	95 (10)	2064 (70)	2125 (77)	362 (10)	365 (12)

Abbreviations: CPOE, computerized provider order entry; MDRO, multidrug-resistant organism; MRSA, methicillin-resistant *Staphylococcus aureus*; POA, present on admission; VRE, vancomycin-resistant enterococci.

SI conversion factors: To convert absolute neutrophil and white blood cell counts to ×10^9^/L multiply by 0.001.

^a^ Unless otherwise indicated. Denominators for all percentages calculated are admissions with cancer for each subgroup, unless otherwise specified.

^b^ Other race category included Asian, Hawaiian, Native American, and multiracial.

^c^ Health care exposures are limited to those documented within a prior inpatient or emergency department visit in the HCA Healthcare electronic medical record.

^d^ Time to first antibiotics includes first dose of any antibiotics administered in the emergency department or inpatient wards from 2 days before date of admission up to 3 days of hospitalization.

^e^ Time to first positive MDRO culture result is defined as time from collection to first blood culture that yielded an MDRO within the empiric period (hospital days 2-3).

^f^ History of multidrug resistant pathogen included any prior growth of pathogen requiring extended-spectrum antibiotics and includes any MRSA or VRE polymerase chain reaction positivity, *International Statistical Classification of Diseases and Related Health Problems, Tenth Revision (ICD-10) *coding, or any infection prevention isolation flag placed in the patient’s medical record for any of these organisms.

^g^ ESBL and *Acinetobacter* and *Pseudomonas* species with multidrug resistance to antipseudomonal antibiotics but susceptible to carbapenem and carbapenem-resistant Enterobacterales.

^h^ Carbapenem-resistant Enterobacterales, *Acinetobacter,* and *Pseudomonas*.

^i^ Selected from Elixhauser comorbidity conditions; chronic pulmonary disease includes pulmonary circulation disease; diabetes includes with and without chronic complications; anemias includes anemias due to nutritional and iron deficiencies; liver disease includes mild, moderate, and severe; renal disease includes moderate and severe; and neurologic disease includes dementia, cerebrovascular disease, paralysis, neurologic disorders affecting movement, seizures and epilepsy, and other neurologic diseases. Solid tumor includes with and without metastases; hematologic malignant tumor includes lymphoma and leukemia.

^j^ Elixhauser count is the sum of each comorbid condition (among 38) as available in the electronic health record for each patient.

^k^ Discharge diagnosis code for neutropenia (*ICD-10* codes D70.0-D70.9) present on admission.

^l^ Minimum absolute neutrophil count and white blood cell count measured within first 3 days of admission.

The proportion of patients with severe neutropenia at admission ranged from 2% to 6% across the trials. Study groups were well balanced within each trial, including similar percentages of insurance type, prior health care and antibiotic exposure, comorbid conditions, and history of MDROs.

### MDRO Culture Positivity

The number of patients across both study groups and periods with blood or site-specific cultures sent within 3 days of admission were 8942 of 9923 (90%) for pneumonia, 9292 of 10 074 (92%) for UTI, 3502 of 4187 (84%) for SSTI, and 9011 of 12 677 (71%) for abdominal infections (Table 2). In patients with pneumonia and abdominal infections, culture results were positive for MRSA in less than 2.3%, for *Pseudomonas* in less than 2.7% and ESBL in less than 2.6% of patients across study groups and periods combined (Table 2).

**Table 2.** Group Comparisons of Source-Specific or Blood Culture Collection and MDRO Growth Among Cancer Cohorts Included in the INSPIRE Trials

	No. (%)															
	Pneumonia				Urinary tract infection				Skin and soft tissue infection				Abdominal infection			
	Baseline (12 mo)^a^		Intervention (12 mo)^b^		Baseline (12 mo)^a^		Intervention (12 mo)^b^		Baseline (18 mo)^a^		Intervention (15 mo)^b^		Baseline (18 mo)^a^		Intervention (15 mo)^b^	
	Routine	CPOE^c^	Routine	CPOE^c^	Routine	CPOE^c^	Routine	CPOE^c^	Routine	CPOE^c^	Routine	CPOE^c^	Routine	CPOE^c^	Routine	CPOE^c^
Total patients	2511 (9)	2554 (8)	2427 (10)	2431 (9)	2756 (6)	2754 (6)	2296 (7)	2268 (7)	1049 (4)	943 (3)	1217 (4)	978 (3)	2953 (6)	2752 (6)	3817 (7)	3155 (6)
Cultures collected (any)^d^	2265 (90)	2292 (90)	2217 (91)	2168 (89)	2554 (93)	2569 (93)	2096 (91)	2073 (91)	893 (85)	813 (86)	997 (82)	799 (82)	2078 (70)	1987 (72)	2682 (70)	2264 (72)
Infection source^e^	716 (29)	716 (28)	718 (30)	781 (32)	2361 (86)	2362 (86)	1808 (79)	1691 (75)	375 (36)	325 (34)	370 (30)	299 (31)	698 (24)	711 (26)	714 (19)	654 (21)
Blood infection source	2208 (88)	2223 (87)	2155 (89)	2085 (86)	1931 (70)	2016 (73)	1647 (72)	1664 (73)	803 (77)	752 (80)	922 (76)	748 (76)	1884 (64)	1800 (65)	2466 (65)	2088 (66)
Positive blood or infection source culture results^f^																
Any MDRO^g^	103 (5)	99 (4)	91 (4)	110 (5)	376 (15)	406 (16)	342 (16)	345 (17)	136 (15)	120 (15)	142 (14)	116 (15)	110 (5)	97 (5)	120 (4)	100 (4)
MRSA	51 (2)	33 (1)	32 (1)	34 (2)	48 (2)	43 (2)	24 (1)	43 (2)	73 (8)	66 (8)	66 (7)	52 (7)	26 (1)	22 (1)	25 (1)	16 (1)
VRE	3 (0.1)	7 (0.3)	0	2 (0.1)	28 (1)	35 (1)	17 (0.8)	17 (0.8)	4 (0.4)	5 (0.6)	6 (0.6)	4 (0.5)	10 (0.5)	7 (0.4)	9 (0.3)	6 (0.3)
* Pseudomonas*	37 (2)	49 (2)	47 (2)	58 (3)	107 (4)	142 (6)	116 (6)	108 (5)	51 (6)	50 (6)	59 (6)	52 (7)	29 (1)	34 (2)	24 (1)	35 (2)
ESBL^h^	19 (0.8)	17 (0.7)	16 (0.7)	22 (1)	204 (8)	206 (8)	210 (10)	195 (9)	24 (3)	24 (3)	28 (3)	17 (2)	52 (3)	42 (2)	71 (3)	41 (2)
CRE^i^	4 (0.2)	4 (0.2)	3 (0.1)	2 (0.1)	14 (0.5)	16 (0.6)	12 (0.6)	17 (0.8)	8 (0.9)	3 (0.4)	6 (0.6)	8 (1)	4 (0.2)	0	5 (0.2)	4 (0.2)
Positive blood culture results^j^																
Any MDRO^g^	48 (2)	48 (2)	37 (2)	55 (3)	60 (3)	83 (4)	51 (3)	62 (4)	23 (3)	18 (2)	33 (4)	27 (4)	64 (3)	60 (3)	85 (3)	56 (3)
MRSA	26 (1)	15 (0.7)	14 (0.6)	19 (0.9)	15 (0.8)	16 (0.8)	9 (0.5)	15 (0.9)	9 (1)	8 (1)	18 (2)	8 (1)	15 (0.8)	14 (0.8)	18 (0.7)	11 (0.5)
VRE	3 (0.1)	5 (0.2)	0	2 (0.1)	4 (0.2)	4 (0.2)	0	0	2 (0.2)	1 (0.1)	4 (0.4)	0	4 (0.2)	1 (0.1)	6 (0.2)	1 (0.05)
* Pseudomonas*	11 (0.4)	16 (1)	13 (1)	20 (1)	4 (0.2)	20 (1)	8 (0.5)	12 (1)	7 (0.9)	5 (0.7)	5 (0.5)	14 (2)	13 (0.7)	16 (0.9)	15 (0.6)	19 (0.9)
ESBL^h^	10 (0.5)	14 (0.6)	11 (0.6)	17 (0.8)	38 (2)	44 (2)	34 (2)	36 (2)	7 (0.9)	4 (0.5)	8 (0.9)	5 (0.7)	34 (2)	29 (2)	48 (2)	24 (1)
CRE^i^	2 (0.1)	0	0	0	2 (0.1)	2 (0.1)	1 (0.1)	0	0	0	1 (0.1)	1 (0.1)	0	0	1 (0.04)	1 (0.05)
Total neutropenic patients	93 (4)	125 (5)	106 (4)	133 (5)	59 (2)	80 (3)	72 (3)	88 (4)	56 (5)	55 (6)	61 (5)	58 (6)	151 (5)	133 (5)	222 (6)	146 (5)
Cultures collected (any)^d^	90 (97)	117 (94)	101 (95)	126 (95)	59 (100)	78 (98)	66 (92)	83 (94)	43 (77)	51 (93)	48 (79)	50 (86)	128 (85)	121 (91)	178 (80)	127 (87)
Infection source^e^	28 (30)	29 (23)	34 (32)	45 (34)	53 (90)	69 (86)	46 (64)	53 (60)	12 (21)	10 (18)	11 (18)	8 (14)	60 (40)	46 (35)	42 (19)	42 (29)
Blood infection source	90 (97)	116 (93)	99 (93)	125 (94)	53 (90)	73 (91)	60 (83)	79 (90)	41 (73)	51 (93)	47 (77)	50 (86)	123 (81)	112 (84)	168 (76)	123 (84)
Positive blood or infection source culture results																
Any MDRO^g^	10 (11)	9 (8)	8 (8)	14 (11)	7 (12)	11 (14)	13 (20)	13 (16)	8 (19)	7 (14)	4 (8)	9 (18)	11 (9)	3 (2)	10 (6)	7 (6)
MRSA	3 (3)	1 (1)	1 (1)	2 (2)	0	1 (1)	1 (2)	0	3 (7)	2 (4)	3 (6)	3 (6)	2 (2)	0	1 (0.6)	1 (0.8)
VRE	0	1 (1)	0	0	0	0	0	0	0	0	0	0	2 (2)	0	0	0
* Pseudomonas*	6 (7)	7 (6)	5 (5)	10 (8)	2 (3)	5 (6)	6 (9)	6 (7)	5 (12)	3 (6)	1 (2)	5 (10)	3 (2)	2 (2)	5 (3)	4 (3)
ESBL^h^	2 (2)	0	3 (3)	2 (2)	5 (8)	5 (6)	6 (9)	7 (8)	1 (2)	2 (4)	0	3 (6)	4 (3)	1 (0.8)	5 (3)	2 (2)
CRE^i^	1 (1)	0	0	0	0	0	1 (2)	1 (1)	0	0	0	0	0	0	0	0
Blood cultures positive^j^																
Any MDRO^g^	9 (10)	5 (4)	7 (7)	10 (8)	1 (2)	3 (4)	3 (5)	6 (8)	2 (5)	4 (8)	1 (2)	6 (12)	9 (7)	3 (3)	10 (6)	6 (5)
MRSA	2 (2)	0	0	1 (0.8)	0	0	0	0	0	1 (2)	1 (2)	1 (2)	2 (2)	0	1 (0.6)	1 (0.8)
VRE	0	1 (0.9)	0	0	0	0	0	0	0	0	0	0	1 (0.8)	0	0	0
* Pseudomonas*	6 (7)	4 (3)	5 (5)	7 (6)	0	2 (3)	2 (3)	3 (4)	1 (2)	2 (4)	0	4 (8)	3 (2)	2 (2)	5 (3)	4 (3)
ESBL^h^	2 (2)	0	3 (3)	2 (2)	1 (2)	1 (1)	1 (2)	3 (4)	1 (2)	1 (2)	0	1 (2)	3 (2)	1 (0.9)	5 (3)	1 (1)
CRE^i^	1 (1)	0	0	0	0	0	0	0	0	0	0	0	0	0	0	0

Abbreviations: CPOE, computerized provider order entry; CRE, carbapenem-resistant Enterobacterales; MDRO, multidrug-resistant organism; MRSA, methicillin-resistant *Staphylococcus aureus*; VRE, vancomycin-resistant enterococci.

^a^ Baseline period was 18 months for pneumonia and urinary tract infection and 12 months for skin and soft tissue infection and abdominal infection.

^b^ Intervention period was 15 months for pneumonia and urinary tract infection and 12 months for skin and soft tissue infection and abdominal infection.

^c^ CPOE bundle intervention consisted of education, feedback, and CPOE prompts recommending standard-spectrum antibiotics for patients with low MDRO risk.

^d^ Infection-specific source or blood cultures sent during the empiric period (hospital days 1-3) and the associated emergency department stay; denominator for percentages are among admissions with cancer.

^e^ Respiratory source cultures included any sample collected from the respiratory tract: nares, sputum, trachea, bronchoalveolar lavage, or pleural fluid samples, regardless of colony or leukocyte count. Urinary source cultures included any sample collected from the urinary tract: bladder (including from catheter), ureter, kidney (drain, nephrostomy, aspirate), and urostomy, regardless of colony or leukocyte count. Skin and soft tissue source cultures included any sample collected from the skin or soft tissue source: superficial skin or wound cultures obtained from any skin site of the body from scalp to foot, including decubitus ulcers, gangrene, implant-associated wounds, external genitalia, muscle, or bone. Abdominal source cultures included any sample collected from the abdominal cavity: stool, wounds, fluid or abscess or tissue cultures obtained during procedures or drainage tubes from any intraperitoneal and non-urinary retroperitoneal organ or cavity (eg, peritoneal, hepatobiliary, pancreas, spleen, upper and lower gastrointestinal tract, ovaries, and uterus).

^f^ Denominator for percentages are among cultures collected from infection-specific source or blood source within each study group.

^g^ Growth of one or more of any MDRO pathogen listed in the subsequent rows; some cultures were positive for more than one MDRO

^h^ ESBL and *Acinetobacter* and *Pseudomonas* species with multidrug resistance to antipseudomonal antibiotics but susceptible to carbapenem.

^i^ Carbapenem-resistant *Acinetobacter* and *Pseudomonas* species.

^j^ Denominator for percentages are among cultures collected from blood source within each study group.

We assessed the percentage with positive blood or site-specific culture results requiring extended-spectrum antibiotics across all MDROs, study groups, and study periods. Overall, the percentage of any MDRO was 5% (403 of 8942) for pneumonia, 16% (1469 of 9292) for UTI, 15% (514 of 3502) for SSTI, and 5% (427 of 9011) for abdominal infections. The most common MDRO in UTI was ESBL, which was positive in 9% (815 of 9292 patients); similarly high MDRO rates in SSTIs were attributable to MRSA and *Pseudomonas,* which was positive in 7% (257 of 3502) and 6% (212 of 3502) of patients with cancer, respectively. When evaluating the subset of cultures collected from blood only, 810 of 27 392 (3%) tested positive for an MDRO across all infections, study groups, and study periods (Table 2). Among these, bacteremia from MRSA was in 1% (found in 230 of 27 392 patients), *Pseudomonas* in 1% (198 of 27 392), and ESBL in 363 of 27 392).

### Antibiotic Prescribing and MDRO Risk Estimation

The percentage of patients who received any (≥1) empiric extended-spectrum antibiotics was high during the baseline period for all infections, ranging from 54.7% to 73.3% during baseline for both groups across all trials (eTable 3 and eFigure 1 in Supplement 1). For the CPOE group, baseline percentages of any extended-spectrum use decreased from 72.3% (1869 of 2554) to 60.3% (1466 of 2431) in the pneumonia trial, from 54.7% (1507 of 2754) to 49.4% (1120 of 2268) in the UTI trial, from 64.7% (610 of 943) to 51.2% (501 of 978) in the SSTI trial, and from 61.5% (1692 of 2752) to 47.5% (1498 of 3155) in the abdominal infection trial.

In contrast to the high proportions of patients receiving extended-spectrum antibiotics, the CPOE algorithm classified most patients as low risk. Among patients with pneumonia or abdominal infection, more than 98.3% and 95.4% were classified as low risk for MRSA and *Pseudomonas*, respectively; of these, fewer than 1.4% had MRSA and fewer than 1.9% had *Pseudomonas* (eTable 4 in Supplement 1). For patients with UTI infections, more than 93.7% were classified as low risk; of these, fewer than 3.5% grew *Pseudomonas* and fewer than 9% grew ESBL, MDR *Acinetobacter*, or MDR-*Pseudomonas*. For patients with SSTI, the prompt algorithm classified more than 55.2% as low risk for MRSA yet only 2.1% of these low-risk patients were found to have MRSA-positive cultures; for *Pseudomonas*, more than 93.4% were classified as low risk, and fewer than 4.8% of these patients returned with *Pseudomonas*-positive cultures.

### Extended-Spectrum Antibiotic Days of Therapy

Changes in monthly extended-spectrum days of therapy during baseline and intervention periods for routine and CPOE bundle groups are shown in the Figure. For the primary outcome, relative to routine stewardship, extended-spectrum days of therapy in the CPOE hospitals was lower by 27% (rate ratio [RR], 0.73; 95% CI, 0.66-0.80; *P* < .001) in the pneumonia trial, 24% (RR, 0.76; 95% CI, 0.68-0.84; *P* < .001) in the UTI trial, 17% (RR, 0.83; 95% CI, 0.74-0.92; *P* < .001) in the SSTI trial, and 24% (RR, 0.76; 95% CI, 0.69-0.84; *P* < .001) in the abdominal infection trial (Table 3). Secondary outcomes of vancomycin and antipseudomonal days of therapy showed similar reductions.

**Figure.**
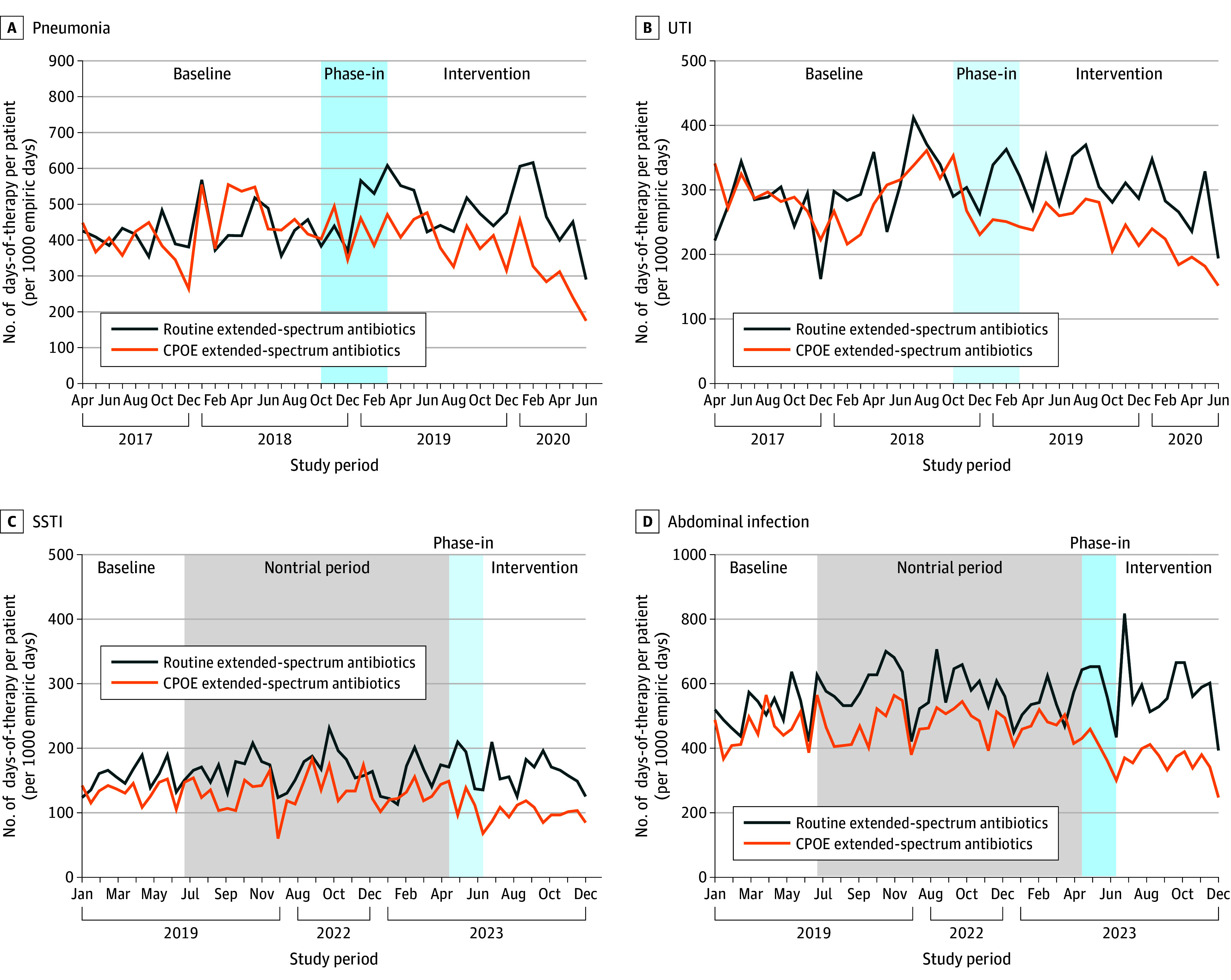
Line Graphs of Monthly Empiric Extended-Spectrum Antibiotic Days of Therapy Among Patients With Cancer in the Routine Stewardship vs Computerized Provider Order Entry (CPOE) Bundle Groups Temporal trends in empiric (hospital days 1-3) extended-spectrum antibiotic days of therapy show sustained reductions in monthly extended-spectrum antibiotic use in the intervention group that was evident early in the phase-in period in immunocompromised patients hospitalized with pneumonia (A), urinary tract infection (UTI) (B), skin and soft tissue infection (SSTI) (C), and abdominal infection (D). The SSTI (1C) and abdominal infection (1D) trials omitted COVID-19 pandemic time (January 2020 to July 2022) from analysis.

**Table 3.** Primary and Secondary Outcomes for Patients With Cancer in the INSPIRE Trials: Adjusted Analysis^a^

Outcome	Routine					CPOE					Overall rate ratio difference in differences (95% CI)	*P* value^c^
	Baseline		Intervention		Rate ratio (95% CI)	Baseline		Intervention		Rate ratio (95% CI)		
	Days of therapy raw rate^b^	No. days of therapy/No. of empiric days	Days of therapy raw rate^b^	No. of days of therapy/No. of empiric days		Days of therapy raw rate^b^	No. of days of therapy /No. of empiric days	Days of therapy raw rate^b^	No. of days of therapy/No. of empiric days			
**Pneumonia**												
Primary outcome												
Extended-spectrum days of therapy	1025.2	7554/7368	1004.1	7115/7086	0.97 (0.91-1.03)	1018.2	7617/7481	755.1	5383/7129	0.70 (0.66-0.75)	0.73 (0.67-0.80)	<.001
Secondary outcomes												
Vancomycin days of therapy	408.9	3013/7368	375.2	2659/7086	0.92 (0.84-0.99)	397.4	2973/7481	277.0	1975/7129	0.69 (0.64-0.75)	0.76 (0.67-0.85)	<.001
Antipseudomonal days of therapy	556.1	4097/7368	569.3	4034/7086	1.01 (0.95-1.07)	564.0	4219/7481	441.6	3148/7129	0.76 (0.72-0.81)	0.76 (0.69-0.83)	<.001
**Urinary tract infection**												
Primary outcome												
Extended-spectrum days of therapy	643.2	5189/8067	666.9	4463/6692	1.02 (0.95-1.10)	635.8	5122/8056	518.9	3452/6653	0.77 (0.71-0.83)	0.76 (0.68-0.84)	<.001
Secondary outcomes												
Vancomycin days of therapy	204.4	1649/8067	198.3	1327/6692	0.94 (0.84-1.05)	200.3	1614/8056	155.9	1037/6653	0.73 (0.65-0.82)	0.77 (0.66-0.91)	.001
Antipseudomonal days of therapy	365.9	2952/8067	377.2	2524/6692	1.03 (0.95-1.11)	351.5	2832/8056	292.5	1946/6653	0.79 (0.73-0.86)	0.77 (0.69-0.86)	<.001
**Skin and soft tissue infection**												
Primary outcome												
Extended-spectrum days of therapy	602.6	1862/3090	545.3	1957/3589	0.89 (0.83-0.96)	568.4	1574/2769	416.0	1196/2875	0.74 (0.68-0.80)	0.83 (0.74-0.92)	<.001
Secondary outcomes												
Vancomycin days of therapy	555.3	1716/3090	582.2	1799/3589	0.91 (0.85-0.98)	499.0	1542/2769	428.8	1325/2875	0.84 (0.78-0.91)	0.92 (0.83-1.02)	.111
Antipseudomonal days of therapy	564.7	1745/3090	498.7	1790/3589	0.87 (0.81-0.94)	525.5	1455/2769	386.1	1110/2875	0.73 (0.67-0.80)	0.84 (0.74-0.94)	.004
**Abdominal infection**												
Primary outcome												
Extended-spectrum days of therapy	708.8	6137/8658	622.8	6992/11 226	0.86 (0.80-0.91)	666.2	5384/8082	459.7	4274/9297	0.65 (0.61-0.70)	0.76 (0.69-0.84)	<.001
Secondary outcomes												
Vancomycin days of therapy	182.3	1578/8658	153.6	1724/11 226	0.81 (0.72-0.91)	179.3	1449/8082	117.6	1093/9297	0.62 (0.55-0.71)	0.77 (0.65-0.91)	.003
Antipseudomonal days of therapy	445.7	3859/8658	401.7	4509/11 226	0.88 (0.82-0.94)	418.2	3380/8082	290.3	2699/9297	0.66 (0.61-0.71)	0.75 (0.68-0.83)	<.001

Abbreviation: CPOE, computerized provider order entry.

^a^ Analysis adjusted for age, sex, race and ethnicity, Medicaid insurance, antibiotic or nursing home exposure in the last year, mean Elixhauser comorbidity count, history of multidrug-resistant organizations, cancer type (hematologic vs solid tumor), and neutropenia status (absolute neutrophil count of 500/μL or less or a white blood cell count less than 1000/μL). Analysis for abdominal infection additionally included history of prior abdominal surgery in the past 1 year.

^b^ Days of therapy rate calculated per admission per empiric day (first 3 days of hospitalization) expressed with multiplier 1000 empiric days.

^c^ Results are based on adjusted generalized linear mixed-effects models that accounted for clustering within patients, hospitals, and periods within hospitals. *P* values were assessed at 2-tailed significance set at α = .05 for null hypothesis that the relative rate ratio in each arm is not different for primary outcome (α = .025 for secondary outcomes to account for multiple comparisons).

### Subgroup Analyses

Point estimates were driven by most of the immunocompromised population without severe neutropenia. Among those with severe neutropenia, counts were small and point estimates were not significantly different from 0. Nevertheless, point estimates were consistently below 1 for this subgroup, except for antipseudomonal agents and outcomes in the SSTI trial, consistent with national guidance for empiric antipseudomonal therapy for febrile neutropenia and anti-MRSA therapy in SSTIs (eTables 5 and 6 in Supplement 1).

### Safety Outcomes

In all trials, frequency of ICU transfers, days to ICU admission, 30-day hospital readmissions, 30-day in-hospital mortality, and median length of stay were similar in the intervention and control groups (Table 4). These outcomes were also similar for each study period (Table 4).

**Table 4.** Group Comparisons of ICU Transfers, Antibiotic Escalations, Length of Stay, and In-Hospital Mortality Among Patients With Cancer in the INSPIRE Trials

Infection	No. (%)^a^							
	Patients with cancer				Patients with cancer and severe neutropenia			
	Baseline^b^		Intervention^c^		Baseline^b^		Intervention^c^	
	Routine	CPOE^d^	Routine	CPOE^d^	Routine	CPOE^d^	Routine	CPOE^d^
Pneumonia								
Cancer admissions with pneumonia	2511	2554	2427	2431	93	125	106	133
No. eligible for ICU transfer^e^	2371 (94)	2393 (94)	2262 (93)	2284 (94)	85 (91)	112 (90)	95 (90)	127 (96)
Transferred to ICU^f^	250 (11)	235 (10)	233 (10)	200 (9)	11 (13)	11 (10)	12 (13)	14 (11)
Time to ICU transfer, d								
Mean (SD)	6 (3)	6 (3)	6 (3)	6 (3)	4 (2)	7 (4)	5 (1)	6 (3)
Median (IQR)	5 (3-8)	5 (4-7)	5 (3-7)	5 (4-8)	3 (3-5)	4 (3-10)	5 (4-6)	5 (4-8)
No. eligible for antibiotic escalation^g^	534 (21)	536 (21)	519 (21)	783 (32)	8 (9)	4 (3)	6 (6)	17 (13)
Received antibiotic escalation^h^	126 (24)	120 (22)	126 (24)	158 (20)	1 (13)	1 (25)	3 (50)	8 (47)
Length of stay, d^i^								
Mean (SD)	7 (4)	8 (4)	7 (4)	8 (4)	8 (4)	8 (4)	7 (4)	9 (4)
Median (IQR)	7 (4-10)	7 (4-11)	7 (4-10)	7 (4-11)	7 (5-11)	8 (5-11)	6 (4-11)	8 (5-14)
Readmission at 30 days^j^	506 (20)	490 (19)	459 (19)	464 (19)	15 (16)	32 (26)	23 (22)	33 (25)
In-hospital mortality^k^	215 (9)	224 (9)	165 (7)	184 (8)	15 (16)	15 (12)	13 (12)	18 (14)
UTI								
Cancer admissions with UTI	2756	2754	2296	2268	59	80	73	88
No. eligible for ICU transfer^e^	2574 (93)	2565 (93)	2122 (92)	2134 (94)	57 (97)	78 (98)	70 (96)	88 (100)
Transferred to ICU^f^	172 (7)	142 (6)	135 (6)	110 (5)	3 (5)	2 (3)	5 (7)	2 (2)
Time to ICU transfer, d								
Mean (SD)	6 (3)	6 (3)	6 (3)	7 (3)	7 (5)	8 (5)	4 (1)	5 (1)
Median (IQR)	5 (4-8)	5 (4-8)	5 (3-8)	6 (4-9)	5 (4-13)	8 (4-11)	5 (3-5)	5 (4-5)
No. eligible for antibiotic escalation^g^	929 (34)	966 (35)	726 (32)	901 (40)	4 (7)	4 (5)	5 (7)	14 (16)
Received antibiotic escalation^h^	168 (18)	158 (16)	124 (17)	142 (16)	0 (0)	1 (25)	3 (60)	4 (29)
Length of stay, d^i^								
Mean (SD)	7 (4)	7 (4)	7 (4)	7 (4)	8 (4)	7 (4)	8 (4)	7 (4)
Median (IQR)	6 (4-10)	6 (4-9)	6 (4-10)	6 (4-10)	7 (5-11)	6 (5-10)	7 (5-11)	7 (5-9)
Readmission at 30 days^j^	547 (20)	570 (21)	478 (21)	458 (20)	14 (24)	19 (24)	21 (29)	24 (27)
In-hospital mortality^k^	134 (5)	127 (5)	94 (4)	85 (4)	3 (5)	2 (3)	8 (11)	3 (3)
SSTI								
Cancer admissions with SSTI	1049	943	1217	978	56	55	61	58
No. eligible for ICU transfer^e^	998 (95)	891 (95)	1160 (95)	924 (95)	55 (98)	53 (96)	58 (95)	56 (97)
Transferred to ICU^f^	61 (6)	56 (6)	61 (5)	62 (7)	8 (15)	3 (6)	4 (7)	7 (13)
Time to ICU transfer, d								
Mean (SD)	7 (3)	7 (3)	7 (3)	6 (3)	6 (3)	6 (4)	6 (2)	6 (2)
Median (IQR)	6 (4-9)	6 (4-9)	6 (4-9)	5 (4-9)	5 (3-9)	4 (3-11)	5 (5-7)	6 (3-7)
No. eligible for antibiotic escalation^g^	122 (12)	106 (11)	187 (15)	201 (21)	6 (11)	1 (2)	11 (18)	10 (17)
Received antibiotic escalation^h^	40 (33)	26 (25)	70 (37)	63 (31)	5 (83)	0 (0)	7 (64)	7 (70)
Length of stay, d^i^								
Mean (SD)	8 (4)	8 (4)	8 (4)	8 (4)	9 (4)	8 (4)	10 (4)	9 (5)
Median (IQR)	7 (5-11)	7 (4-11)	8 (5-12)	8 (4-13)	9 (6-14)	8 (5-11)	10 (5-14)	8 (5-14)
Readmission at 30 days^j^	196 (19)	180 (19)	266 (22)	176 (18)	19 (34)	12 (22)	17 (28)	12 (21)
In-hospital mortality^k^	33 (3)	29 (3)	23 (2)	28 (3)	4 (7)	3 (6)	4 (7)	2 (3)
Abdominal infection								
Patients with cancer and abdominal infection	2953	2752	3817	3155	151	133	222	146
No. eligible for ICU transfer^e^	2772 (94)	2595 (94)	3616 (95)	3000 (95)	144 (95)	127 (96)	218 (98)	137 (94)
Transferred to ICU^f^	249 (9)	213 (8)	274 (7)	213 (7)	15 (10)	11 (9)	15 (7)	12 (9)
Time to ICU transfer, d								
Mean (SD)	6 (3)	6 (3)	6 (3)	6 (3)	5 (2)	5 (3)	7 (3)	7 (3)
Median (IQR)	5 (3-7)	5 (4-7)	5 (4-8)	5 (4-9)	4 (3-8)	4 (3-6)	6 (5-9)	7 (4-10)
No. eligible for antibiotic escalation^g^	847 (29)	834 (30)	1309 (34)	1356 (43)	15 (10)	12 (9)	42 (19)	27 (19)
Received antibiotic escalation^h^	193 (23)	170 (20)	293 (22)	266 (20)	6 (40)	3 (25)	17 (41)	8 (30)
Length of stay, d^i^								
Mean (SD)	8 (4)	8 (4)	8 (4)	8 (4)	8 (4)	8 (4)	9 (4)	8 (4)
Median (IQR)	7 (4-11)	7 (4-10)	7 (4-12)	7 (4-11)	8 (5-12)	7 (5-10)	8 (5-14)	7 (4-12)
Readmission at 30 days^j^	572 (19)	535 (19)	746 (20)	578 (18)	39 (26)	41 (31)	51 (23)	28 (19)
In-hospital mortality^k^	140 (5)	116 (4)	143 (4)	105 (3)	12 (8)	10 (8)	12 (5)	4 (3)

Abbreviations: CPOE, computerized provider order entry; ICU, intensive care unit; SSTI, skin and soft tissue infection; UTI, urinary tract infection.

^a^ Unless otherwise indicated.

^b^ Baseline period was 18 months for pneumonia and urinary tract infection and 12 months for skin and soft tissue infection and abdominal infection.

^c^ Intervention period was 15 months for pneumonia and urinary tract infection and 12 months for skin and soft tissue infection and abdominal infection.

^d^ CPOE bundle intervention consists of education, feedback, and CPOE prompts recommending standard-spectrum antibiotics for patients with low multidrug-resistant organism risk.

^e^ Number of patients eligible for ICU transfer defined as those with length of stay of 3 through 14 days (post hoc evaluation).

^f^ Percentage calculated among those eligible for ICU transfer.

^g^ Number of patients eligible for antibiotic escalation defined as patients who were started using only standard-spectrum antibiotics during the empiric period (post hoc evaluation).

^h^ Percentage calculated among those eligible for antibiotic evaluation.

^i^ Length of stay calculated as days from admission to date of hospital discharge among those discharged alive up to hospital day 14.

^j^ Readmission within 30 days at any HCA Healthcare hospital (does not include admissions at non-HCA hospitals).

^k^ Mortality occurring any time between admission and discharge.

## Discussion

In this secondary analysis of 4 cluster-randomized trials addressing the most common infections requiring hospitalization, a CPOE-based stewardship strategy for empiric antibiotic selection in non–critically ill patients with cancer was associated with significantly reduced extended-spectrum antibiotic use by 17% to 27%, amounts that were comparable to their effect in the general patient populations evaluated in the original trials. Despite these reductions in extended-spectrum antibiotic use, length of stay, frequency of ICU transfer, death, and readmission did not increase. These results suggest that stewardship for initial antibiotic selection is a viable approach for the millions of patients who have cancer, are at low risk for antimicrobial-resistant pathogens, and are hospitalized in the US for infection with one of the conditions studied.

Because patients with cancer frequently require repeated or prolonged courses of antibiotics during their illness, avoiding unnecessary use of extended-spectrum antibiotics in this population can preserve future antibiotic options and effectiveness.^19^ Disruptions in gut microbiota increase the risk of opportunistic infections (eg, *Clostridioides difficile* and invasive candidiasis) but also impact immune regulation, leading to increased susceptibility to future infection.^2,23,24,25,26^ Across all models, patient history of MDRO was the most consistently predictive risk factor, either alone or in combination with a few other variables.^7,8,9,10^

Our finding of low MDRO prevalence across infection syndromes in these large, geographically dispersed cohorts offers reassuring evidence that empiric standard-spectrum antibiotics are adequate for many patients with cancer. Among those identified to be low risk by the CPOE algorithm, MDROs were even less frequently detected, suggesting that risk-based interventions can safely support use of standard-spectrum antibiotics while awaiting culture results.^27,28,29^ Notably, bacteremia with MDRO was rare; *Pseudomonas*, ESBL, and MRSA were each identified in less than 1% of patients across all infection syndromes. This finding is consistent with other studies showing that, although MDRO infection rates are higher among patients who are immunocompromised compared with the general population, the overall rates remain relatively low.^27^ This finding supports the recommendation to start with standard-spectrum antibiotics, which are associated with better clinical outcomes, fewer adverse effects, and a lower risk of promoting antibiotic resistance.^28^

Antibiotic stewardship for patients who have cancer remains a national challenge.^19,30,31^ Despite low MDRO infection rates, we observed high extended-spectrum antibiotic use (54.7%-73.3%). Heightened sensitivity to the fragility of these patients may lead many physicians to start with extended-spectrum antibiotics.^12^ Once started, they are often not deescalated even when microbiology results do not reveal MDROs, likely due to a desire to avoid disrupting clinical recovery.^32,33,34^ Diagnostic uncertainty and concern that cultures may miss MDROs may further contribute to unnecessary use of extended-spectrum antibiotics.^2,35,36^ Findings from this study suggest that providing patient- and pathogen-specific risk estimates that are documented within the electronic health record may offer physicians reassurance to reduce hesitancy toward judicious antibiotic prescribing in this population.

### Limitations

This study has some limitations. First, we identified the immunocompromised population using discharge diagnoses for malignant tumors and were not able to conduct a medical record review to further specify the degree of immunocompromise. Furthermore, the small number of patients with severe neutropenia led to inadequate statistical power to assess safety outcomes in that subset. Second, the study was performed in a national network of community hospitals where MDRO prevalence may be lower than other settings. Third, MDRO risk estimates were not specifically developed for the immunocompromised subpopulation. Fourth, because all clinical specimens were included regardless of their quality, we could not distinguish between true infection and colonization, which may have led to an overestimation of the role of MDROs as pathogens.

## Conclusions

In this secondary analysis of 4 randomized clinical trials, empiric extended-spectrum antibiotic use was significantly lower among non–critically ill adults with cancer admitted for pneumonia, UTI, SSTI, or abdominal infection in hospitals using personalized real-time CPOE prompts coupled with education and prescribing feedback recommending standard-spectrum antibiotics for patients at low risk of MDRO infection. Hospital length of stay, ICU transfer, antibiotic escalations, readmissions, and mortality were unchanged. The findings support scalable, low-burden strategies to improve antimicrobial use in patients with cancer, a population with limited evidence to guide stewardship.

## Notes

Supplement 1. **eTable 1.** INSPIRE Extended-Spectrum Antibiotics **eTable 2.** International Classification Diagnosis (ICD 10) Codes Used to Define Cancer Patient Subsets in the INSPIRE Trials **eTable 3.** Group Comparisons of Patients with Cancer in the INSPIRE Trials Receiving Either Extended- or Standard-Spectrum Antibiotics, or a Combination of Both During the Empiric Period **eFigure 1.** Percent of Patients with Cancer Receiving Any Extended-Spectrum Antibiotics in the INSPIRE Trials **eTable 4.** Group Comparisons of Multidrug-Resistant Organism (MDRO) Growth Among Patients with Cancer in the INSPIRE Trials Classified as Low Absolute Risk (<10%) for Infection due to MDRO **eTable 5.** Adjusted Analysis of Effectiveness Outcomes in Patients with Cancer Who Do Not Have Severe Neutropenia in the INSPIRE Trials **eTable 6.** Adjusted Analysis of Effectiveness Outcomes in Patients with Cancer Who Have Severe Neutropenia in the INSPIRE Trials **eFigure 2.** Hospital Recruitment and Randomization in the INSPIRE Pneumonia Trial **eFigure 3.** Hospital Recruitment and Randomization in the INSPIRE Urinary Tract Infection Trial **eFigure 4.** Hospital Recruitment and Randomization in the INSPIRE Skin and Soft Tissue Trial **eFigure 5.** Hospital Recruitment and Randomization in the INSPIRE Abdominal Infection Trial

Supplement 2.
Trial Protocols and Statistical Analysis Plans

Supplement 3.
Data Sharing Statement

## References

[1] Magill SS, O’Leary E, Ray SM, ; Emerging Infections Program Hospital Prevalence Survey Team. Antimicrobial use in US Hospitals: comparison of results from emerging infections program prevalence surveys, 2015 and 2011. Clin Infect Dis. 2021;72(10):1784-1792. doi:10.1093/cid/ciaa373 32519751 PMC7976440

[2] Centers for Disease Control and Prevention. Antibiotic resistance threats in the United States, 2019. Accessed September 16, 2023. https://www.cdc.gov/antimicrobial-resistance/data-research/threats/index.html

[3] Klompas M. Overuse of broad-spectrum antibiotics for pneumonia. JAMA Intern Med. 2020;180(4):485-486. doi:10.1001/jamainternmed.2019.7251 32065598

[4] Senneville É, Albalawi Z, van Asten SA, . IWGDF/IDSA guidelines on the diagnosis and treatment of diabetes-related foot infections (IWGDF/IDSA 2023). Clin Infect Dis. 2023:ciad527. doi:10.1093/cid/ciad527 37779457

[5] Bonomo RA, Chow AW, Edwards MS, . 2024 Clinical Practice Guideline Update by the Infectious Diseases Society of America on complicated intra-abdominal infections: risk assessment, diagnostic imaging, and microbiological evaluation in adults, children, and pregnant people. Clin Infect Dis. 2024;79(suppl 3):S81-S87. doi:10.1093/cid/ciae346 38965057

[6] Sartelli M, Catena F, Abu-Zidan FM, . Management of intra-abdominal infections: recommendations by the WSES 2016 consensus conference. World J Emerg Surg. 2017;12:22. doi:10.1186/s13017-017-0132-7 28484510 PMC5418731

[7] Gohil SK, Septimus E, Kleinman K, . Stewardship prompts to improve antibiotic selection for pneumonia: the INSPIRE randomized clinical trial. JAMA. 2024;331(23):2007-2017. doi:10.1001/jama.2024.6248 38639729 PMC11185977

[8] Gohil SK, Septimus E, Kleinman K, . Stewardship prompts to improve antibiotic selection for urinary tract infection: the INSPIRE randomized clinical trial. JAMA. 2024;331(23):2018-2028. doi:10.1001/jama.2024.6259 38639723 PMC11185978

[9] Gohil SK, Septimus E, Kleinman K, . Improving empiric antibiotic selection for patients hospitalized with skin and soft tissue infection: the INSPIRE 3 Skin and Soft Tissue Randomized Clinical Trial. JAMA Intern Med. 2025;185(6):680-691. doi:10.1001/jamainternmed.2025.0887 40208610 PMC11986828

[10] Gohil SK, Septimus E, Kleinman K, . Improving empiric antibiotic selection for patients hospitalized with abdominal infection: the INSPIRE 4 cluster randomized clinical trial. JAMA Surg. 2025;160(7):733-743. doi:10.1001/jamasurg.2025.1108 40208583 PMC11986832

[11] Majumdar A, Shah MR, Park JJ, Narayanan N, Kaye KS, Bhatt PJ. Challenges and opportunities in antimicrobial stewardship among hematopoietic stem cell transplant and oncology patients. Antibiotics (Basel). 2023;12(3):592. doi:10.3390/antibiotics12030592 36978459 PMC10044884

[12] Hand J, Imlay H. Antimicrobial stewardship in immunocompromised patients: current state and future opportunities. Infect Dis Clin North Am. 2023;37(4):823-851. doi:10.1016/j.idc.2023.08.002 37741735

[13] Deinhardt-Emmer S, Chousterman BG, Schefold JC, . Sepsis in patients who are immunocompromised: diagnostic challenges and future therapies. Lancet Respir Med. 2025;13(7):623-637. doi:10.1016/S2213-2600(25)00124-9 40409328

[14] Nates JL, Pène F, Darmon M, ; Nine-I Investigators. Septic shock in the immunocompromised cancer patient: a narrative review. Crit Care. 2024;28(1):285. doi:10.1186/s13054-024-05073-0 39215292 PMC11363658

[15] Gupta V, Satlin MJ, Yu K, . Burden of antimicrobial resistance in adult hospitalized patients with cancer: a multicenter analysis. Cancer Med. 2024;13(24):e70495. doi:10.1002/cam4.70495 39673173 PMC11645461

[16] Gupta V, Satlin MJ, Yu KC, . Incidence and prevalence of antimicrobial resistance in outpatients with cancer: a multicentre, retrospective, cohort study. Lancet Oncol. 2025;26(5):620-628. doi:10.1016/S1470-2045(25)00128-7 40318645

[17] Li C, He L, Xu J, . Analysis of risk factors for multidrug-resistant organism (MDRO) infections and construction of a risk prediction model in a cancer specialty hospital. Br J Hosp Med (Lond). 2024;85(10):1-11. doi:10.12968/hmed.2024.0442 39475021

[18] Rink M, Gladstone BP, Nikolai LA, Bitzer M, Tacconelli E, Göpel S. The impact of antibiotic prophylaxis on antibiotic resistance, clinical outcomes, and costs in adult hemato-oncological and surgical patients: a systematic review and meta-analysis. Antibiotics (Basel). 2025;14(9):853. doi:10.3390/antibiotics14090853 41009832 PMC12466468

[19] Liu C, Rosen EA, Stohs EJ, . Tackling antimicrobial resistance in people who are immunocompromised: leveraging diagnostic and antimicrobial stewardship. Lancet Infect Dis. 2026;26(1):e30-e48. doi:10.1016/S1473-3099(25)00311-1 40812337 PMC13218428

[20] Bono R, Sapienza G, Tringali S, . The antibiotic de-escalation strategy in patients with multidrug-resistant bacterial colonization after allogeneic stem cell transplantation. Front Microbiol. 2025;15:1487617. doi:10.3389/fmicb.2024.1487617 39831122 PMC11739814

[21] Agency for Healthcare Research Quality. Patient Safety Index ICD-10, 2022. Healthcare Cost and Utilization Project (HCUP). Accessed January 20, 2024. https://qualityindicators.ahrq.gov/measures/psi_resources

[22] Elixhauser Comorbidity Software Refined for ICD-10-CM. v2023.1, Healthcare Cost and Utilization Project (HCUP). Accessed January 20, 2024. https://hcup-us.ahrq.gov/toolssoftware/comorbidityicd10/comorbidity_icd10.jsp

[23] Hagan T, Cortese M, Rouphael N, . Antibiotics-driven gut microbiome perturbation alters immunity to vaccines in humans. Cell. 2019;178(6):1313-1328.e13. doi:10.1016/j.cell.2019.08.010 31491384 PMC6750738

[24] Yang JH, Bhargava P, McCloskey D, Mao N, Palsson BO, Collins JJ. Antibiotic-induced changes to the host metabolic environment inhibit drug efficacy and alter immune function. Cell Host Microbe. 2017;22(6):757-765.e3. doi:10.1016/j.chom.2017.10.020 29199098 PMC5730482

[25] Tamma PD, Avdic E, Li DX, Dzintars K, Cosgrove SE. Association of adverse events with antibiotic use in hospitalized patients. JAMA Intern Med. 2017;177(9):1308-1315. doi:10.1001/jamainternmed.2017.1938 PMC5710569 28604925

[26] Drummond RA, Desai JV, Ricotta EE, . Long-term antibiotic exposure promotes mortality after systemic fungal infection by driving lymphocyte dysfunction and systemic escape of commensal bacteria. Cell Host Microbe. 2022;30(7):1020-1033.e6. doi:10.1016/j.chom.2022.04.013 35568028 PMC9283303

[27] Lin MY, Weinstein RA, Hota B. Delay of active antimicrobial therapy and mortality among patients with bacteremia: impact of severe neutropenia. Antimicrob Agents Chemother. 2008;52(9):3188-3194. doi:10.1128/AAC.01553-07 18625778 PMC2533498

[28] Saravolatz L, Gandhi TN, Vaughn VM, . Target trial emulation of empiric antibiotics on clinical outcomes in moderately immunocompromised patients hospitalized with pneumonia. Clin Infect Dis. 2025:ciaf344. doi:10.1093/cid/ciaf344 40601818

[29] Danielsen AS, Elstrøm P, Eriksen-Volle HM, . The epidemiology of multidrug-resistant organisms in persons diagnosed with cancer in Norway, 2008-2018: expanding surveillance using existing laboratory and register data. Eur J Clin Microbiol Infect Dis. 2024;43(1):121-132. doi:10.1007/s10096-023-04698-3 37980302 PMC10774199

[30] Majeed A, Rust T. Antimicrobial stewardship in patients with cancer: interventions and future directions to combat the rise of antimicrobial resistance. JCO Oncol Pract. 2025;21(8):1067-1069. doi:10.1200/OP-25-00124 40080740

[31] Lasagna A, Cambieri P, Baldanti F, . How should we manage the impact of antimicrobial resistance in patients with cancer? an oncological and infectious disease specialist point of view. JCO Oncol Pract. 2025;21(8):1097-1105. doi:10.1200/OP-24-00935 39977722

[32] Chen YH, Sun AYE, Narain K, . Efficacy and safety of early antibiotic de-escalation in febrile neutropenia for patients with hematologic malignancy: a systematic review and meta-analysis. Antimicrob Agents Chemother. 2025;69(4):e0159724. doi:10.1128/aac.01597-24 40079575 PMC11963549

[33] Lucena M, Gaffney KJ, Urban T, . Early de-escalation of antibiotic therapy in hospitalized cellular therapy adult patients with febrile neutropenia. Clin Hematol Int. 2024;6(1):59-66. doi:10.46989/001c.94105 38817693 PMC11086988

[34] Ram R, Amit O, Adler A, Early antibiotic deescalation and discontinuation in patients with febrile neutropenia after cellular therapy: a single-center prospective unblinded randomized trial. Transplant Cell Ther. 2023;29(11):708e1-708e8. doi:10.1016/j.jtct.2023.08.013 37591446

[35] Tarrant C, Krockow EM. Antibiotic overuse: managing uncertainty and mitigating against overtreatment. BMJ Qual Saf. 2022;31(3):163-167. doi:10.1136/bmjqs-2021-013615 34285113

[36] Krockow EM, Colman AM, Chattoe-Brown E, . Balancing the risks to individual and society: a systematic review and synthesis of qualitative research on antibiotic prescribing behaviour in hospitals. J Hosp Infect. 2019;101(4):428-439. doi:10.1016/j.jhin.2018.08.007 30099092

