# Supplementary material for: Improving Empiric Antibiotic Selection for Patients With Cancer Hospitalized With Infection: Secondary Analysis of the INSPIRE Cluster Randomized Trials
Source: JAMA Netw Open. 2026 Jun 10;9(6):e2616611. doi: 10.1001/jamanetworkopen.2026.16611 (PMC13254732; doi:10.1001/jamanetworkopen.2026.16611)
Supplement: Supplement 1. — eTable 1. INSPIRE Extended-Spectrum Antibiotics eTable 2. International Classification Diagnosis (ICD 10) Codes Used to Define Cancer Patient Subsets in the INSPIRE Trials eTable 3. Group Comparisons of Patients with Cancer in the INSPIRE Trials Receiving Either Extended- or Standard-Spectrum Antibiotics, or a Combination of Both During the Empiric Period eFigure 1. Percent of Patients with Cancer Receiving Any Extended-Spectrum Antibiotics in the INSPIRE Trials eTable 4. Group Comparisons of Multidrug-Resistant Organism (MDRO) Growth Among Patients with Cancer in the INSPIRE Trials Classified as Low Absolute Risk (<10%) for Infection due to MDRO eTable 5. Adjusted Analysis of Effectiveness Outcomes in Patients with Cancer Who Do Not Have Severe Neutropenia in the INSPIRE Trials eTable 6. Adjusted Analysis of Effectiveness Outcomes in Patients with Cancer Who Have Severe Neutropenia in the INSPIRE Trials eFigure 2. Hospital Recruitment and Randomization in the INSPIRE Pneumonia Trial eFigure 3. Hospital Recruitment and Randomization in the INSPIRE Urinary Tract Infection Trial eFigure 4. Hospital Recruitment and Randomization in the INSPIRE Skin and Soft Tissue Trial eFigure 5. Hospital Recruitment and Randomization in the INSPIRE Abdominal Infection Trial [file jamanetwopen-e2616611-s001.pdf]

Supplement 1

Gohil SK, Avery TR, Kleinman K, et al. Improving empiric antibiotic selection for patients with cancer hospitalized with infection: a secondary analysis of the INSPIRE cluster randomized trials. *JAMA Netw Open*. 2026;9(6):e2616611. doi:10.1001/jamanetworkopen.2026.16611

eTable 1. INSPIRE Extended-Spectrum Antibiotics
eTable 2. International Classification Diagnosis (ICD 10) Codes Used to Define Cancer Patient Subsets in the INSPIRE Trials
eTable 3. Group Comparisons of Patients with Cancer in the INSPIRE Trials Receiving Either Extended- or Standard-Spectrum Antibiotics, or a Combination of Both During the Empiric Period
eFigure 1. Percent of Patients with Cancer Receiving Any Extended-Spectrum Antibiotics in the INSPIRE Trials
eTable 4. Group Comparisons of Multidrug-Resistant Organism (MDRO) Growth in Patients with Cancer in the INSPIRE Trials Classified as Low Absolute Risk (<10%) for Infection due to MDRO
eTable 5. Adjusted Analysis of Effectiveness Outcomes in Patients with Cancer Who Do Not Have Severe Neutropenia in the INSPIRE Trials
eTable 6. Adjusted Analysis of Effectiveness Outcomes in Patients with Cancer Who Have Severe Neutropenia in the INSPIRE Trials
eFigure 2. Hospital Recruitment and Randomization in the INSPIRE Pneumonia Trial
eFigure 3. Hospital Recruitment and Randomization in the INSPIRE Urinary Tract Infection Trial
eFigure 4. Hospital Recruitment and Randomization in the INSPIRE Skin and Soft Tissue Trial
eFigure 5. Hospital Recruitment and Randomization in the INSPIRE Abdominal Infection Trial

This supplemental material has been provided by the authors to give readers additional information about their work.

© 2026 Gohil SK et al. *JAMA Network Open*.

eTable 1. INSPIRE Extended-Spectrum Antibiotics

Pathogen-Directed Antibiotic Category	Extended-Spectrum Antibiotics Targeted in INSPIRE CPOE Prompts
**Anti-MRSA[a]**	Ceftaroline, Daptomycin, Linezolid[b], Vancomycin[c]
**Anti-VRE**	Daptomycin, Linezolid[b], Tigecycline[d]
**Antipseudomonal**	Aztreonam, Cefepime, Ceftazidime, Piperacillin/Tazobactam
**Anti-ESBL**	Ertapenem, Meropenem, Imipenem, Ceftolozane/Tazobactam, Doripenem[e]
**Anti-CRE**	Ceftazidime/Avibactam, Colistin, Imipenem/Relebactam, Meropenem/Vaborbactam, Polymixin B, Tigecycline

[a]Anti-MRSA extended-spectrum antibiotics were not included in the INSPIRE Skin and Soft Tissue Infection trial since prevalence of MRSA exceeded 10%.
[b]Both oral and intravenous (IV) formulations.
[c]IV formulation only.
[d]Tigecycline targeted for skin and soft tissue infection only.
[e]Doripenem targeted for urinary tract infection only.

Abbreviations: CPOE – Computerized Provider Order Entry, MRSA – Methicillin-Resistant *Staphylococcus aureus,* ESBL – Extended-Spectrum Beta-Lactamase Producing Enterobacterales including *Acinetobacter* and *Pseudomonas* species with multidrug-resistance to antipseudomonal antibiotics but can be treated with a carbapenem or ceftolozane/tazobactam, CRE – Carbapenem-Resistant Enterobacterales, including Carbapenem-Resistant *Acinetobacter* and *Pseudomonas* species.

© 2026 Gohil SK et al. *JAMA Network Open*.

eTable 2. International Classification Diagnosis (ICD 10) Codes Used to Define Cancer Patient Subsets in the INSPIRE Trials

Description	ICD10 Code
Malignant Neoplasm	C000-C009,C01,C020-C031,C039-C052, C058-C062, C0680, C0689, C069, C07, C080-C081, C098-C104, C108-C113, C118-C119, C12, C130-132, C138-142, C148, C153-155, C158-166, C168-C173, C178-C189, C19, C20, C210-212, C218, C228-229, C23, C240-C241, C248-C254, C257-261, C269, C300-301, C310-313, C318-C323, C328-C329, C33, C3400-3402, C3410-3412, C3432, C3480-C3482, C3490-C3492, C37, C380-C384, C388, C390, C399, C4000-C4002, C4010-C4012, C4020-4022, C4030-C4032, C4080-C4082, C4090-C4092, C410-C414, C419, C4409, C44101-44102, C441021-C441022, C4409, C44101, C44102, C441021, C441022, C44109, C441091, C441092, C44131, C441321, C441322, C441391, C441392, C44191, C44192, C441921, C441922, C44199, C441991, C441992, C44201, C44202, C44209, C44291, C44292, C44299, C44300, C44301, C44309, C44390, C44391, C44399, C4440, C4449, C44500, C44501, C44509, C44590, C44591, C44599, C44601, C44602, C44609, C44691, C44692, C44699, C44701, C44702, C44709, C44791, C44792, C44799, C4480, C4489, C4490, C4499, C470, C4710-4712, C4720-C4722, C473-C476, C478-C490, C4910-4912, C4920-C4922, C493-C496, C489-C499, C50011, C50012, C50019, C50021, C50022, C50029, C50111, C50112, C50119, C50121, C50122, C50129, C50211, C50212, C50219, C50221, C50222, C50229, C50311, C50312, C50319, C50321, C50322, C50329, C50411, C50412, C50419, C50421, C50422, C50429, C50511, C50512, C50519, C50521, C50522, C50529, C50611, C50612, C50619, C50621, C50622, C50629, C50811, C50812, C50819, C50821, C50822, C50829, C50911, C50912, C50919, C50921, C50922, C50929, C510-C512, C518, C519, C52, C530, C531, C538-C543, C543, C548, C549, C55, C561-C563, C569, C5700-5702, C5710-C5712, C5720-C5722, C573, C574, C577-C579, C58, C600-C602, C689, C609, C61, C6200-C6202, C6210-C6212, C6290-C6292, C6300-C6302, C6310-C6312, C632, C637-C639, C641, C642, C649, C651, C652, C659, C661, C662, C669-C681, C688, C689, C6900-C6902, C6910-C6912, C6920-C6922, C6930-C6932, C6940-C6942, C6950-C6952, C6960-6962, C6980-C6982, C6990-C6992, C700, C701, C709-C721, C7220-

Description	ICD10 Code
Secondary malignant neoplasm	C770-C779, C7800-C7802, C781, C782, C7830, C7839, C784-C787, C7900-C7902, C7910, C7911, C7919, C792, C7931, C7932, C7940, C7949, C7951, C7952, C7960-C7963, C7970-C7972, C7981, C7982, C7989, C799,
Neoplasms	D47Z9
Multiple endocrine neoplasia	E3121-E3123
Malignant Melanoma	C4310-C4312,C43111-43112, C43121-C43122, C4320-C4322, C4339, C434, C4351-C4352, C4359-C4362, C4370-C4372, C438-C439, C4400
Melanoma in situ	D030, D0310, D0311, D03111, D03112, D0312, D03121, D03122, D0320, D0321, D0322, D0330, D0339, D034, D0351, D0352, D0359- D0362, D0370- D0372, D038, D039
Carcinoma in situ	D0000-D0008, D001, D002, D010-D013, D0140, D0149, D015, D017, D019-D021, D0220-D0222, D023, D024, D040, D0410, D0411, D04111, D04112, D0412, D04121, D04122, D0420, D0421, D0422, D0430, D0439, D044, D045, D0460, D0461, D0462, D0470, D0471, D0472, D048, D049, D0500-D0502, D0510-D0512, D0580-D0582, D0590-D0592, D060, D061, D067, D069, D070, D071, D072, D0730, D0739, D074, D075, D0760, D0761, D0769, D090, D0910, D0919, D0920, D0921, D0922, D093, D098, D099
Merkel cell carcinoma	C4A0, C4A10, C4A11, C4A111, C4A112, C4A12, C4A121, C4A122, C4A20, C4A21, C4A22, C4A30, C4A31, C4A39, C4A4, C4A51, C4A52, C4A59, C4A70-C4A72, C4A8, C4A9
Secondary Merkel cell carcinoma	C7B1

© 2026 Gohil SK et al. *JAMA Network Open*.

	7222, C7230-C7232, C7240-C7242, C7250, C7259, C7259, C73, C7400-C7402, C7410-C7412, C7490-C7492, C750-C755, C758-C763, C7640-C7642, C7650-C7652, C768, C800, C9620, C9629, C969, C96Z

Malignant carcinoid tumor	C7A00, C7A010-C7A012, C7A019-C7A026, C7A029, C7A090-C7A096, C7A098,
Secondary carcinoid tumors	C7B00- C7B04, C7B09,

Description	ICD10 Code
Liver cell carcinoma	C220, C227
Intrahepatic bile duct carcinoma	C221
Hepatoblastoma	C222
Sarcomas	C9230-C9232, C223, C224, C9622, C964, C96A
Kaposi's sarcoma	C460-C464, C4650-C4652, C467, C469
Secondary neuroendocrine tumors	C7B8,
Malignant neuroendocrine tumors	C7A1, C7A8,
Nodular lymphocyte predominant Hodgkin lymphoma	C8100-C8109
Nodular sclerosis Hodgkin lymphoma	C8110-C8119
Mixed cellularity Hodgkin lymphoma	C8120-C8129
Lymphocyte depleted Hodgkin lymphoma	C8130-C8139
Lymphocyte-rich Hodgkin lymphoma	C8140-C8149
Non-Hodgkin lymphoma	C8580-C8599
Hodgkin lymphoma	C8170-C8179, C8190-C8199
Follicular Lymphoma	C8200-C8249, C8280-C8299
Diffuse follicle center lymphoma	C8250-C8259
Cutaneous follicle center lymphoma	C8260-C8269
B-cell lymphoma	C8510-C8519, C884, C8520-C8529, C8300-C8309, C8330-C8339
Mantle cell lymphoma	C8310-C8319
Lymphoblastic (diffuse) lymphoma	C8350-C8359
Burkitt lymphoma	C8370- C8379
Non-follicular lymphoma	C8380-C8389, C8390-C8400

Description	ICD10 Code
Lymphoblastic leukemia	C9100-C9102
Lymphocytic leukemia	C9110-C9112
Prolymphocytic leukemia	C9130-C9132, C9160-C9162
Hairy cell leukemia	C9140-C9142
T-cell lymphoma/leukemia	C9150-C9152
Lymphoid leukemia	C9190-C9192, C91Z0-C91Z2
Mature B-cell leukemia Burkitt-type	C91A0-C91A2
Myeloid leukemia	C9200-C9202, C9210-C9212, C9220-C9222, C9260-C9262, C9290-C9292, C92A0-C92A2, C92Z0-C92Z2
Promyelocytic leukemia	C9240-C9242
Monocytic leukemia	C9390-C9392, C93Z0-C93Z2
Myelomonocytic leukemia	C9250-C9252, C9310-C9312, C9330-C9332
Monoblastic/monocytic leukemia	C9300-C9302
Megakaryoblastic leukemia	C9420-C9422
Mast cell leukemia	C9430-C9432
Mesothelioma	C450-C452, C457, C459
Gastrointestinal stromal tumor	C49A0-C49A5, C49A9
Mycosis fungoides	C8400-C8409
Sezary disease	C8410-C8419
Waldenstrom macroglobulinemia	C880
Heavy chain disease	C882
Immunoproliferative diseases	C883, C888
Multiple myeloma	C9000-C9002

© 2026 Gohil SK et al. *JAMA Network Open*.

Anaplastic large cell lymphoma	C8460-C8479, C847A
T/NK-cell lymphomas	C8490-C8499, C84Z0- C84Z9, C860
T-cell lymphoma	C84A0-C84A9, C8440-C8449, C861-C866, C865
NK-cell lymphomas	C864
T-cell proliferations	C866
Leukemia	C9480-C9482, C9500-C9502, C9510-C9512, C9590-C9592
Plasma cell leukemia	C9010-C9012

Plasmacytoma	C9020-C9022, C9030-C9032
Panmyelosis with myelofibrosis	C9440-C9442
Myelodysplastic disease	C946
Langerhans-cell histiocytosis	C960
Malignant mast cell tumor	C962
Aggressive systemic mastocytosis	C9621
Myelodysplastic syndrome	D469

© 2026 Gohil SK et al. *JAMA Network Open*.

eTable 3: Group Comparisons of Patients with Cancer in the INSPIRE Trials Receiving Either Extended- or Standard-Spectrum Antibiotics, or a Combination of Both During the Empiric Period

	Baseline		Intervention	
	Routine N (%)[a]	CPOE[a,b] N (%)	Routine N (%)[a]	CPOE[a,b] N (%)
**PNEUMONIA[c]**				
**Patients Hospitalized for Pneumonia**	2,511	2,554	2,427	2,431
**Type of Antibiotic Regimen Received[d]**				
Extended-Spectrum Only	951 (37.9)	988 (38.7)	705 (29.0)	494 (20.3)
Extended- and Standard-Spectrum	913 (36.4)	904 (35.4)	1,077 (44.4)	991 (40.8)
Standard-Spectrum Only	523 (20.8)	504 (19.7)	579 (23.9)	876 (36.0)
Fluoroquinolone Only	124 (4.9)	158 (6.2)	66 (2.7)	70 (2.9)
**URINARY TRACT INFECTION[c]**				
**Patients Hospitalized for Urinary Tract Infection**	2,756	2,754	2,296	2,268
**Type of Antibiotic Regimen Received[c]**				
Extended-Spectrum Only	719 (26.1)	694 (25.2)	564 (24.6)	378 (16.7)
Extended- and Standard-Spectrum	820 (29.8)	828 (30.1)	771 (33.6)	750 (33.1)
Standard-Spectrum Only	1,096 (39.8)	1,092 (39.7)	912 (39.7)	1,099 (48.5)
Fluoroquinolone Only	121 (4.4)	140 (5.1)	49 (2.1)	41 (1.8)
**SKIN AND SOFT TISSUE INFECTION[e]**				
**Patients Hospitalized for Skin and Soft Tissue Infection**	1,049	943	1,217	978
**Type of Antibiotic Regimen Received[d]**				
Extended-Spectrum Only	365 (34.8)	331 (35.1)	359 (29.5)	182 (18.6)
Extended- and Standard-Spectrum	360 (34.3)	281 (29.8)	421 (34.6)	321 (32.8)
Standard-Spectrum Only	154 (14.7)	161 (17.1)	228 (18.7)	264 (27.0)
Fluoroquinolone Only	13 (1.2)	3 (0.3)	17 (1.4)	6 (0.6)
**ABDOMINAL INFECTION[e]**				
**Patients Hospitalized for Abdominal Infection**	2,953	2,752	3,817	3,155
**Type of Antibiotic Regimen Received[d]**				
Extended-Spectrum Only	905 (30.6)	841 (30.6)	995 (26.1)	524 (16.6)
Extended- and Standard-Spectrum	970 (32.8)	851 (30.9)	1,223 (32.0)	974 (30.9)

© 2026 Gohil SK et al. *JAMA Network Open*.

Standard-Spectrum Only	1,003 (34.0)	989 (35.9)	1,538 (40.3)	1,605 (50.9)
Fluoroquinolone Only	75 (2.5)	71 (2.6)	61 (1.6)	52 (1.6)

[a]Computerized provider order entry (CPOE) bundle intervention consisted of education, feedback, and CPOE prompts recommending standard-spectrum antibiotics for patients with low MDRO risk.

[b]Percents calculated among total patients within each trial arm and period.

[c]Mutually exclusive categories; patients were classified as “extended-spectrum only” if received only an extended-spectrum antibiotic throughout the empiric period (first 3 calendar days of hospitalization) or “standard-spectrum only” if received only standard-spectrum. Patients were classified as receiving both (“extended- and standard-spectrum”) if receiving one or more antibiotic from either category.

© 2026 Gohil SK et al. *JAMA Network Open*.

eFigure 1: Percent of Patients with Cancer Receiving Any Extended-Spectrum Antibiotics in the INSPIRE Trials

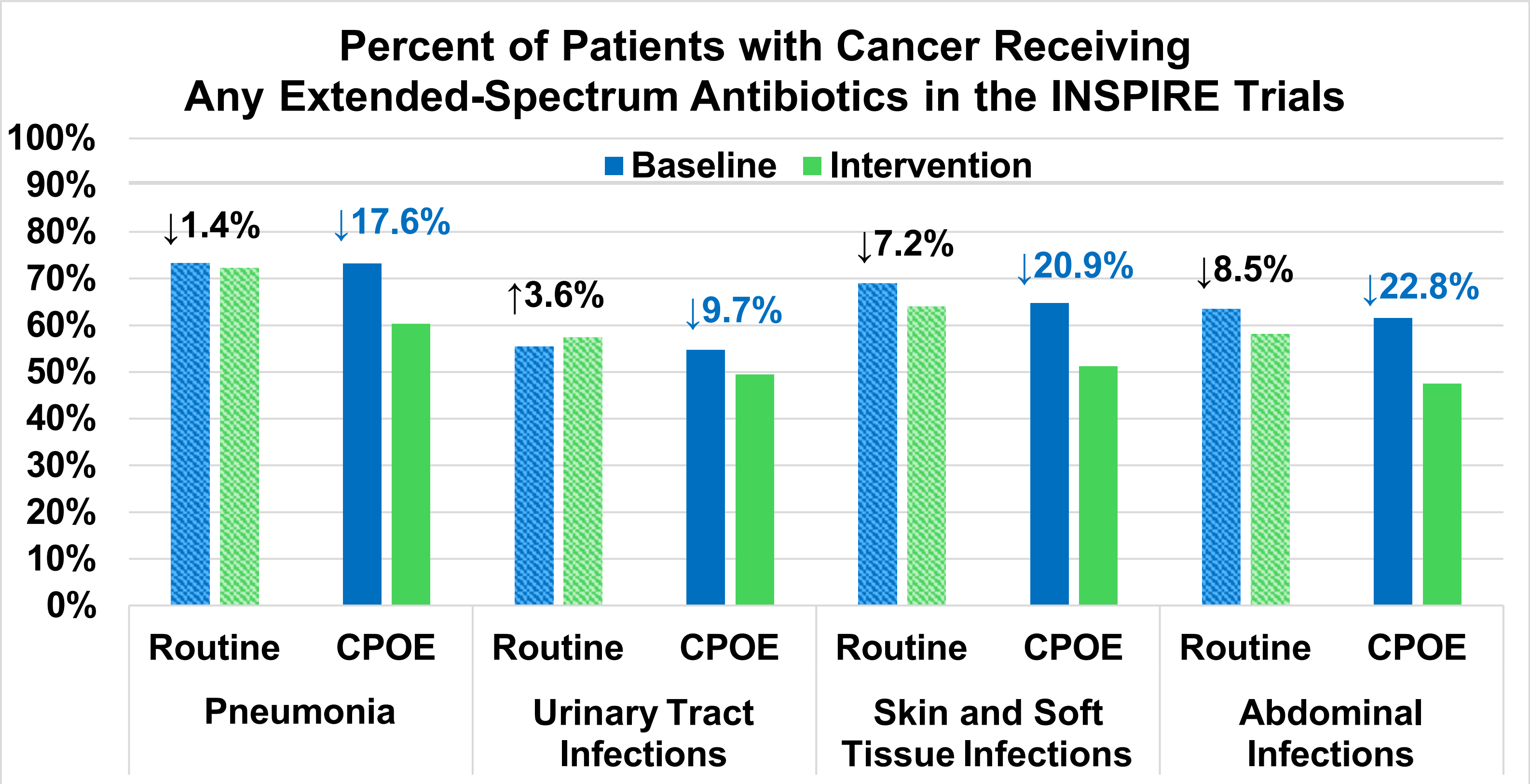

Figure 1: Percentage of patients with cancer who were admitted with pneumonia, urinary tract infection, skin and soft tissue infection, or abdominal infection who were given one or more extended-spectrum antibiotic during the empiric period (hospital days 1-3) during the baseline and intervention periods of each INSPIRE trial.

© 2026 Gohil SK et al. *JAMA Network Open*.

eTable 4: Group Comparisons of Multidrug-Resistant Organism (MDRO) Growth in Patients with Cancer Who Were Included in the INSPIRE Trials and Classified as Low Absolute Risk (<10%) for MDRO Infection

MDRO Risk Estimate Model[a]	Classified Low Risk N (%)	Classified Low Risk and Grew MDRO[b] N (%)	Classified Low Risk N (%)	Classified Low Risk and Grew MDRO[b] N (%)
**PNEUMONIA**				
	**Routine N = 2,427**		**CPOE[c] N = 2,431**	
**Methicillin-Resistant *Staphylococcus aureus* (MRSA)**	2,427 (100)	32 (1.3)	2,422 (99.6)	33 (1.4)
Pseudomonas	2,327 (95.9)	36 (1.5)	2,318 (95.4)	46 (1.9)
**ESBL and MDR-*Acinetobacter*** (Pathogen susceptible to ertapenem)[d]	2,427 (100)	15 (0.6)	2,431 (100)	21 (0.9)
**ESBL, MDR-*Acinetobacter*, and MDR-*Pseudomonas*** (Pathogen susceptible to meropenem, imipenem)[e]	2,427 (100)	16 (0.7)	2,431 (100)	22 (0.9)
**Carbapenem-Resistant Enterobacterales[f]**	2,427 (100)	3 (0.1)	2,431 (100)	2 (0.1)
**URINARY TRACT INFECTION**				
	**Routine N = 2,296**		**CPOE[c] N = 2,268**	
**Methicillin-Resistant *Staphylococcus aureus* (MRSA)**	2,241 (97.6)	20 (0.9)	2,217 (97.8)	36 (1.6)
Pseudomonas	2,154 (93.8)	81 (3.5)	2,125 (93.7)	81 (3.5)
**ESBL and MDR-*Acinetobacter*** (Pathogen susceptible to ertapenem)[d]	2,123 (92.5)	145 (6.3)	2,107 (92.9)	132 (5.7)
**ESBL, MDR-*Acinetobacter*, and MDR-*Pseudomonas*** (Pathogen susceptible to meropenem, imipenem)[e]	2,272 (99)	205 (8.9)	2,219 (97.8)	178 (7.8)
**Carbapenem-Resistant Enterobacterales[f]**	2,296 (100)	12 (0.5)	2,268 (100)	17 (0.7)
**SKIN AND SOFT TISSUE INFECTION**				

© 2026 Gohil SK et al. *JAMA Network Open*.

	Routine N = 1,217		CPOE[c] N = 978	
**Methicillin-Resistant *Staphylococcus aureus* (MRSA)**	672 (55.2)	26 (2.1)	574 (58.7)	20 (2)
Pseudomonas	1,137 (93.4)	49 (4)	926 (94.7)	47 (4.8)
**ESBL and MDR-*Acinetobacter*** (Pathogen susceptible to ertapenem)[d]	1,198 (98.4)	24 (2)	965 (98.7)	13 (1.3)
**ESBL, MDR-*Acinetobacter*, and MDR-*Pseudomonas*** (Pathogen susceptible to meropenem, imipenem)[e]	1,191 (97.9)	26 (2.1)	960 (98.2)	16 (1.6)
**Carbapenem-Resistant Enterobacterales**[f]	1,217 (100)	6 (0.5)	978 (100)	8 (0.8)
**ABDOMINAL INFECTION**				
	**Routine N = 3,817**		**CPOE[c] N = 3,155**	
**Methicillin-Resistant *Staphylococcus aureus* (MRSA)**	3,754 (98.3)	24 (0.6)	3,136 (99.4)	16 (0.4)
Pseudomonas	3,803 (99.6)	24 (0.6)	3,138 (99.5)	34 (0.9)
**ESBL and MDR-*Acinetobacter*** (Pathogen susceptible to ertapenem)[d]	3,739 (98.0)	65 (1.7)	3,088 (97.9)	33 (0.9)
**ESBL, MDR-*Acinetobacter*, and MDR-*Pseudomonas*** (Pathogen susceptible to meropenem, imipenem)[e]	3,731 (97.7)	66 (1.7)	3,077 (97.5)	33 (0.9)
**Carbapenem-Resistant Enterobacterales**[f]	3,817 (100)	5 (0.1)	3,155 (100)	4 (0.1)

[a]Multidrug-resistant organism (MDRO) risk estimate models categorized according to extended-spectrum antibiotic group used to treat each MDRO for each infection syndrome.

[b]Patients classified as low absolute risk (<10%) for pneumonia, urinary tract infection, skin/soft tissue infection, or abdominal infection due to an MDRO who eventually did grow an MDRO from blood or infection-specific source during trial intervention period. Percentage among those classified as low risk.

[c]Computerized provider order entry (CPOE) bundle intervention: consisting of education, feedback, and CPOE prompts recommending standard-spectrum antibiotics for patients with low MDRO risk.

[d]Extended-Spectrum Beta-Lactamase Producing *Enterobacterales* (ESBL) and multidrug-resistant (MDR) *Acinetobacter* susceptible to ertapenem

[e]ESBL and *Acinetobacter* or *Pseudomonas* species with multidrug resistance to antipseudomonal antibiotics but susceptible to meropenem or imipenem or ceftolozane/tazobactam

[f]CRE - Carbapenem-Resistant *Enterobacterales*, including Carbapenem-Resistant *Acinetobacter* and *Pseudomonas* species.

© 2026 Gohil SK et al. *JAMA Network Open*.

eTable 5: Adjusted Analysis of Effectiveness Outcomes in Patients with Cancer Who Do Not Have Severe Neutropenia in the INSPIRE Trials

Pneumonia								
**Effectiveness Outcomes**	**Routine**			**CPOE**			**Overall Rate Ratio Difference-in-Differences**	**P-value**[d]
	**Baseline** Days-of-Therapy Raw Rate[b]	**Intervention** Days-of-Therapy Raw Rate[b]	**Rate Ratio** (95% CI)[c]	**Baseline** Days-of-Therapy Raw Rate[b]	**Intervention** Days-of-Therapy Raw Rate[b]	**Rate Ratio** (95% CI)[c]		
**Primary Outcome**								
Extended-spectrum days-of-therapy	1,007 (7,145/7,099)	983 (6,663/6,781)	0.97 (0.91-1.04)	995 (7,087/7,121)	722 (4,862/6,737)	0.69 (0.64-0.74)	0.71 (0.64-0.79)	<.001
**Secondary Outcomes**								
Vancomycin days-of-therapy	401 (2,846/7,099)	366 (2,481/6,781)	0.92 (0.85-1.00)	388 (2,764/7,121)	263 (1,774/6,737)	0.67 (0.62-0.74)	0.74 (0.65-0.83)	<.001
Antipseudomonal days-of-therapy	548 (3,890/7,099)	557 (3,780/6,781)	1.00 (0.94-1.08)	553 (3,936/7,121)	424 (2,854/6,737)	0.75 (0.70-0.80)	0.74 (0.67-0.82)	<.001
**Urinary Tract Infections**								
**Effectiveness Outcomes**	**Routine**			**CPOE**			**Overall Rate Ratio Difference-in-Differences**	**P-value**[d]
	**Baseline** Days-of-Therapy Raw Rate[b]	**Intervention** Days-of-Therapy Raw Rate[b]	**Rate Ratio** (95% CI)[c]	**Baseline** Days-of-Therapy Raw Rate[b]	**Intervention** Days-of-Therapy Raw Rate[b]	**Rate Ratio** (95% CI)[c]		
**Primary Outcome**								
Extended-spectrum days-of-therapy	628 (4,955/7,892)	648 (4,200/6,477)	1.03 (0.95-1.11)	614 (4,797/7,819)	491 (3,138/6,389)	0.76 (0.70-0.83)	0.74 (0.66-0.83)	<.001
**Secondary Outcomes**								
Vancomycin days-of-therapy	198 (1,561/7,892)	191 (1,236/6,477)	0.95 (0.84-1.06)	191 (1,490/7,819)	147 (941/6,389)	0.74 (0.65-0.83)	0.78 (0.66-0.92)	0.003
Antipseudomonal days-of-therapy	357 (2,818/7,892)	369 (2,390/6,477)	1.04 (0.96-1.13)	340 (2,656/7,819)	278 (1,775/6,389)	0.78 (0.72-0.85)	0.75 (0.67-0.85)	<.001
**Skin and Soft Tissue Infections**								
**Effectiveness Outcomes**	**Routine**			**CPOE**			**Overall Rate Ratio Difference-in-Differences**	**P-value**[d]
	**Baseline** Days-of-Therapy Raw Rate[b]	**Intervention** Days-of-Therapy Raw Rate[b]	**Rate Ratio** (95% CI)[c]	**Baseline** Days-of-Therapy Raw Rate[b]	**Intervention** Days-of-Therapy Raw Rate[b]	**Rate Ratio** (95% CI)[c]		
**Primary Outcome**								
Extended-spectrum days-of-therapy	590 (1,726/2,924)	545 (1,858/3,410)	0.91 (0.84-0.98)	548 (1,429/2,606)	402 (1,086/2,703)	0.74 (0.68-0.81)	0.82 (0.73-0.92)	<.001

© 2026 Gohil SK et al. *JAMA Network Open*.

**Secondary Outcomes**								
Vancomycin days-of-therapy	552 (1,615/2,924)	502 (1,713/3,410)	0.92 (0.86-0.99)	550 (1,432/2,606)	454 (1,228/2,703)	0.84 (0.78-0.91)	0.91 (0.82-1.01)	0.080
Antipseudomonal days-of-therapy	557 (1,629/2,924)	501 (1,708/3,410)	0.89 (0.82-0.96)	508 (1,324/2,606)	373 (1,008/2,703)	0.73 (0.67-0.81)	0.83 (0.73-0.94)	0.003
**ABDOMINAL INFECTIONS**								
**Effectiveness Outcomes**	**Routine**			**CPOE**			**Overall Rate Ratio Difference-in-Differences**	**P-value**[d]
	**Baseline** Days-of-Therapy Raw Rate[b]	**Intervention** Days-of-Therapy Raw Rate[b]	**Rate Ratio** (95% CI)[c]	**Baseline** Days-of-Therapy Raw Rate[b]	**Intervention** Days-of-Therapy Raw Rate[b]	**Rate Ratio** (95% CI)[c]		
**Primary Outcome**								
Extended-spectrum days-of-therapy	679 (5,575/8,213)	598 (6,314/10,564)	0.85 (0.80-0.92)	638 (4,903/7,689)	438 (3,886/8,870)	0.64 (0.59-0.69)	0.75 (0.68-0.83)	<.001
**Secondary Outcomes**								
Vancomycin days-of-therapy	170 (1,398/8,213)	143 (1,513/10,564)	0.80 (0.70-0.90)	168 (1,291/7,689)	112 (991/8,870)	0.61 (0.53-0.70)	0.77 (0.64-0.93)	0.006
Antipseudomonal days-of-therapy	430 (3,530/8,213)	388 (4,101/10,564)	0.87 (0.81-0.94)	404 (3,106/7,689)	274 (2,427/8,870)	0.64 (0.59-0.69)	0.73 (0.66-0.82)	<.001

[a]Analysis adjusted for patients with leukemia, lymphoma, metastatic cancer, cancer without metastasis in situ, solid tumors, or neutropenia. Analysis for abdominal infection only adjusted for abdominal surgery.

[b]Days-of-therapy rate calculated per patient per empiric day (first 3 days of hospitalization) expressed with multiplier 1,000 empiric days.

[c]Rate ratios represent group-specific comparisons of intervention to baseline.

[d]Results are based on generalized linear mixed effects models that accounted for clustering within patients, hospitals, and period within hospital. P-value assessed at 2-tailed significance set at $\alpha = 0.05$ for null hypothesis that the relative rate ratio in each arm is not different for primary outcome; $\alpha=0.025$ for secondary outcomes to account for multiple comparisons.

© 2026 Gohil SK et al. *JAMA Network Open*.

eTable 6: Adjusted Analysis of Effectiveness Outcomes in Patients with Cancer Who Have Severe Neutropenia in the INSPIRE Trials

**PNEUMONIA**								
**Effectiveness Outcomes**	**Routine**			**CPOE**			**Overall Rate Ratio Difference-in-Differences**	**P-value**[d]
	**Baseline** Days-of-Therapy Raw Rate[b]	**Intervention** Days-of-Therapy Raw Rate[b]	**Rate Ratio** (95% CI)[c]	**Baseline** Days-of-Therapy Raw Rate[b]	**Intervention** Days-of-Therapy Raw Rate[b]	**Rate Ratio** (95% CI)[c]		
**Primary Outcome**								
Extended-spectrum days-of-therapy	1,520 (409/269)	1,482 (452/305)	0.96 (0.84-1.11)	1,472 (530/360)	1,329 (521/392)	0.90 (0.80-1.02)	0.93 (0.77-1.13)	0.478
**Secondary Outcomes**								
Vancomycin days-of-therapy	621 (167/269)	584 (178/305)	0.94 (0.75-1.17)	581 (209/360)	513 (201/392)	0.88 (0.72-1.07)	0.94 (0.70-1.26)	0.659
Antipseudomonal days-of-therapy	770 (207/269)	833 (254/305)	1.07 (0.88-1.29)	786 (283/360)	750 (294/392)	0.97 (0.82-1.14)	0.91 (0.71-1.16)	0.444
**URINARY TRACT INFECTIONS**								
**Effectiveness Outcomes**	**Routine**			**CPOE**			**Overall Rate Ratio Difference-in-Differences**	**P-value**[d]
	**Baseline** Days-of-Therapy Raw Rate[b]	**Intervention** Days-of-Therapy Raw Rate[b]	**Rate Ratio** (95% CI)[c]	**Baseline** Days-of-Therapy Raw Rate[b]	**Intervention** Days-of-Therapy Raw Rate[b]	**Rate Ratio** (95% CI)[c]		
**Primary Outcome**								
Extended-spectrum days-of-therapy	1,337 (234/175)	1,223 (263/215)	0.91 (0.76-1.10)	1,371 (325/237)	1,189 (314/264)	0.86 (0.73-1.01)	0.94 (0.74-1.20)	0.640
**Secondary Outcomes**								
Vancomycin days-of-therapy	502.9 (88/175)	423.3 (91/215)	0.85 (0.61-1.17)	523.2 (124/237)	363.6 (96/264)	0.71 (0.53-0.95)	0.84 (0.55-1.29)	0.420
Antipseudomonal days-of-therapy	765.7 (134/175)	623.3 (134/215)	0.86 (0.67-1.10)	742.6 (176/237)	647.7 (171/264)	0.89 (0.71-1.11)	1.04 (0.75-1.44)	0.900
**SKIN AND SOFT TISSUE INFECTIONS**								
**Effectiveness Outcomes**	**Routine**			**CPOE**			**Overall Rate Ratio Difference-in-Differences**	**P-value**[d]
	**Baseline** Days-of-Therapy Raw Rate[b]	**Intervention** Days-of-Therapy Raw Rate[b]	**Rate Ratio** (95% CI)[c]	**Baseline** Days-of-Therapy Raw Rate[b]	**Intervention** Days-of-Therapy Raw Rate[b]	**Rate Ratio** (95% CI)[c]		
**Primary Outcome**								
Extended-spectrum days-of-therapy	819 (136/166)	553 (99/179)	0.65 (0.50-0.85)	890 (145/163)	640 (110/172)	0.75 (0.59-0.97)	1.16 (0.80-1.67)	0.430

© 2026 Gohil SK et al. *JAMA Network Open*.

**Secondary Outcomes**								
Vancomycin days-of-therapy	608 (101/166)	480 (86/179)	0.78 (0.58-1.05)	675 (110/163)	564 (97/172)	0.90 (0.67-1.19)	1.15 (0.76-1.75)	0.500
Antipseudomonal days-of-therapy	699 (116/166)	458 (82/179)	0.89 (0.82-0.96)	804 (131/163)	593 (102/172)	0.73 (0.67-0.81)	0.83 (0.73-0.94)	0.003
**ABDOMINAL INFECTIONS**								
**Effectiveness Outcomes**	**Routine**			**CPOE**			**Overall Rate Ratio Difference-in-Differences**	**P-value**[d]
	**Baseline** Days-of-Therapy Raw Rate[b]	**Intervention** Days-of-Therapy Raw Rate[b]	**Rate Ratio** (95% CI)[c]	**Baseline** Days-of-Therapy Raw Rate[b]	**Intervention** Days-of-Therapy Raw Rate[b]	**Rate Ratio** (95% CI)[c]		
**Primary Outcome**								
Extended-spectrum days-of-therapy	1,263 (562/445)	1,024 (678/662)	0.83 (0.73-0.95)	1,224 (481/393)	909 (388/427)	0.75 (0.65-0.88)	0.90 (0.74-1.11)	0.330
**Secondary Outcomes**								
Vancomycin days-of-therapy	405 (180/445)	319 (211/662)	0.81 (0.63-1.04)	402 (158/393)	239 (102/427)	0.60 (0.44-0.82)	0.74 (0.50-1.10)	0.140
Antipseudomonal days-of-therapy	739 (329/445)	616 (408/662)	0.85 (0.73-0.99)	697 (274/393)	637 (272/427)	0.91 (0.77-1.09)	1.07 (0.85-1.35)	0.560

[a]Analysis adjusted for age, gender, race, insurance status, prior history of nursing home and antibiotic exposure, history of MRSA, Pseudomonas, ESBL, CRE, presence of leukemia, lymphoma, metastatic cancer, cancer without metastasis in situ, solid tumors. Analysis for abdominal infection only adjusted for abdominal surgery.

[b]Days-of-therapy rate calculated per patient per empiric day (first 3 days of hospitalization) expressed with multiplier 1,000 empiric days.

[c]Rate ratios represent group-specific comparisons of intervention to baseline.

[d]Results are based on generalized linear mixed effects models that accounted for clustering within patients, hospitals, and period within hospital. P-value assessed at 2-tailed significance set at $\alpha = 0.05$ for null hypothesis that the relative rate ratio in each arm is not different for primary outcome; $\alpha=0.025$ for secondary outcomes to account for multiple comparisons.

© 2026 Gohil SK et al. *JAMA Network Open*.

eFigure 2. Hospital Recruitment and Randomization in the INSPIRE Pneumonia Trial

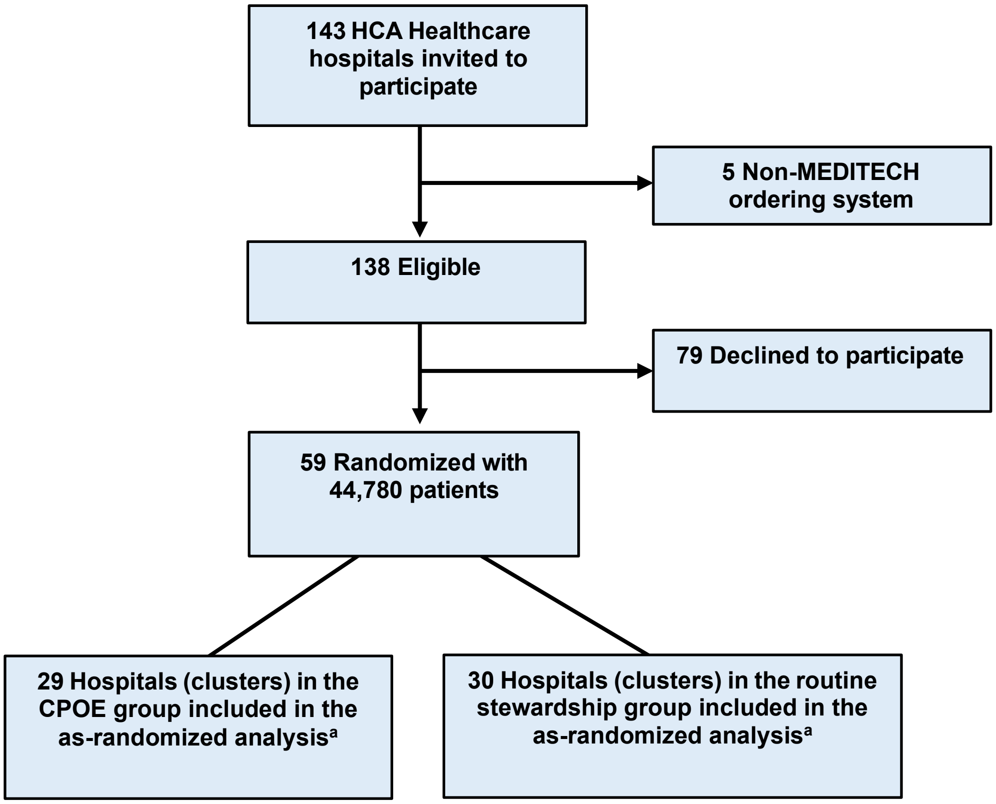

MEDITECH is a hospital electronic health record system. CPOE indicates computerized provider order entry; INSPIRE, Intelligent Stewardship Prompts to Improve Real-time Empiric Antibiotic Selection.

[a]All analyses are as-randomized because all hospitals remained in the trial until end of intervention (no hospital withdrawals after enrollment). There was a median (IQR) of 1,679 (1,019-2,319) patients per hospital in the CPOE bundle group and 1,544 (1,204-1,971) in the routine stewardship group.

© 2026 Gohil SK et al. *JAMA Network Open*.

Hospital Recruitment and Randomization in the INSPIRE Urinary Tract Infection Trial

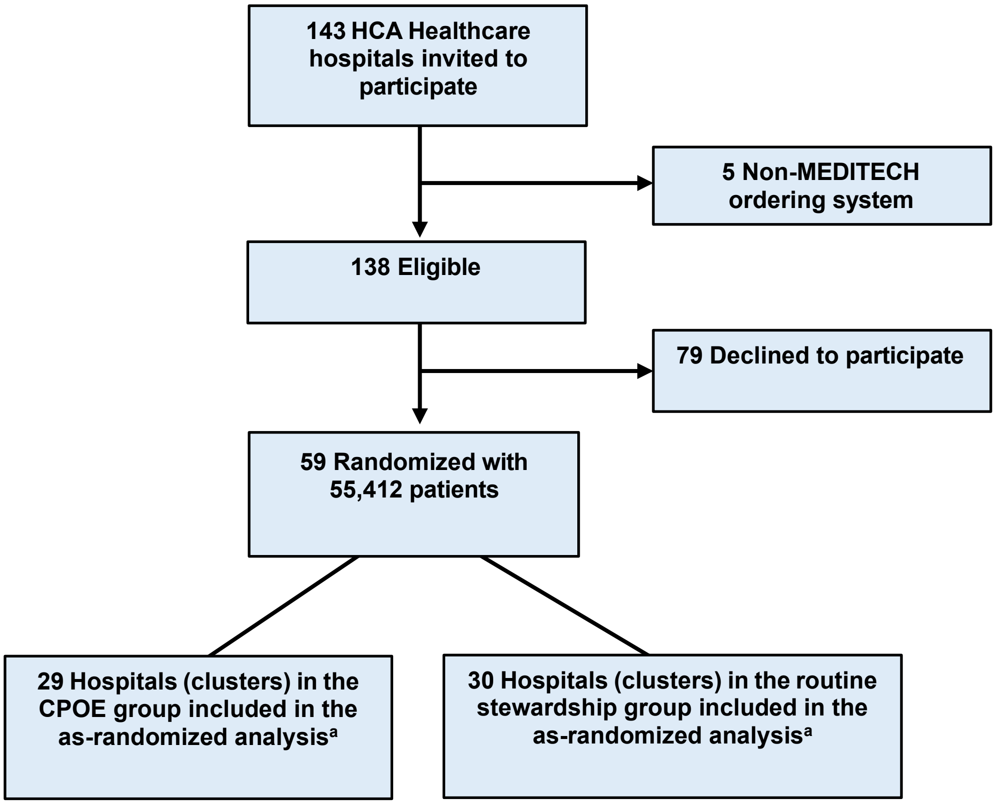

MEDITECH is a hospital electronic health record system. CPOE indicates computerized provider order entry and INSPIRE, Intelligent Stewardship Prompts to Improve Real-time Empiric antibiotic selection.

[a]All analyses are as-randomized because all hospitals remained in the trial until end of intervention (no hospital withdrawals after enrollment). There was a median (IQR) of 2,364 (1,277-2,963) patients per hospital in the CPOE bundle group and 2,008 (1,365-3,064) in the routine stewardship group.

© 2026 Gohil SK et al. *JAMA Network Open*.

Gohil SK, Septimus E, Kleinman K, et al. Improving Empiric Antibiotic Selection for Patients Hospitalized With Skin and Soft Tissue Infection: The INSPIRE 3 Skin and Soft Tissue Randomized Clinical Trial. *JAMA Intern Med.* Published online April 10, 2025. doi:10.1001/jamainternmed.2025.0887

Hospital Recruitment and Randomization in the INSPIRE Skin and Soft Tissue Trial

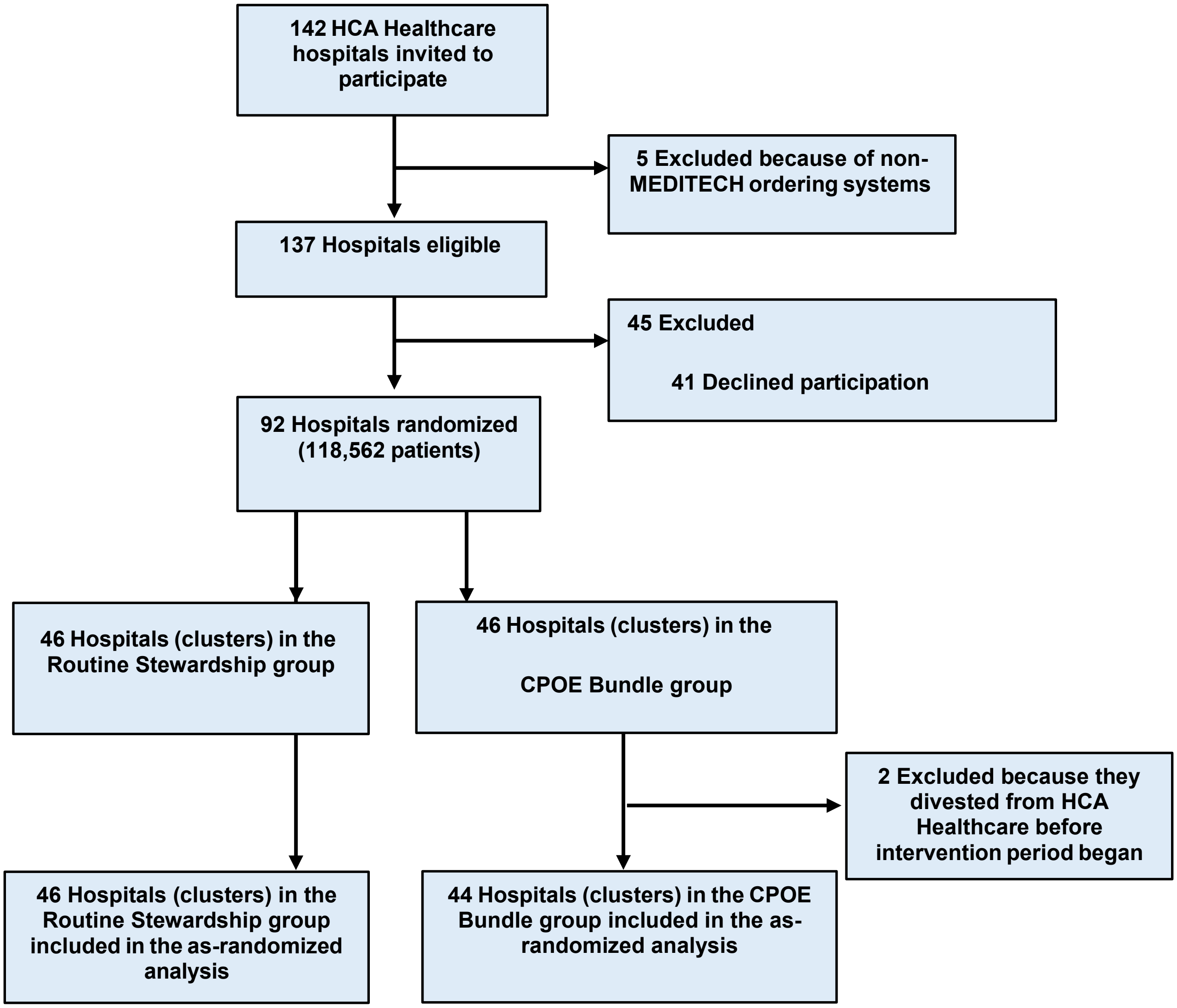

All analyses are as randomized because all hospitals remained in the trial until end of intervention (no hospital withdrawals after intervention period began). There was a median (IQR) of 1,332 (652-1,675) patients per hospital in the routine stewardship group and 1,097 (671-1,528) patients in the CPOE bundle group. MEDITECH is a hospital electronic health record system. CPOE indicates computerized provider order entry.

© 2026 Gohil SK et al. *JAMA Network Open*.

Gohil SK, Septimus E, Kleinman K, et al. Improving Empiric Antibiotic Selection for Patients Hospitalized With Abdominal Infection: The INSPIRE 4 Cluster Randomized Clinical Trial. JAMA Surg. Published online April 10, 2025. doi:10.1001/jamasurg.2025.1108

Hospital Recruitment and Randomization in the INSPIRE Abdominal Infection Trial

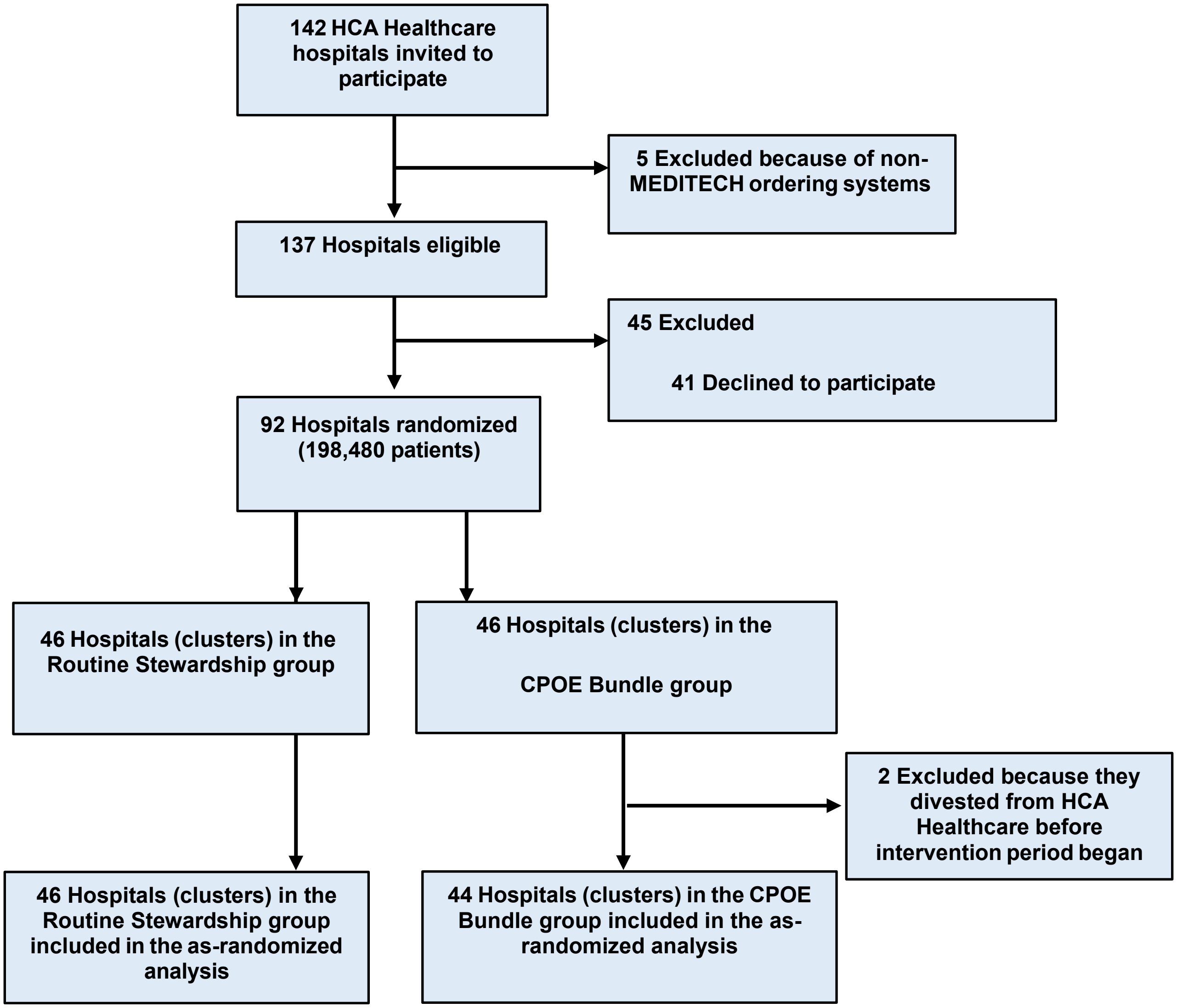

All analyses are as-randomized because all hospitals remained in the trial until end of intervention (no hospital withdrawals after enrollment). There was a median (IQR) of 2,055 (1,289-2,739) patients per hospital in the routine stewardship group and 1,876 (1,157-2,702) in the Computerized Provider Order Entry (CPOE) Bundle group. MEDITECH is a hospital electronic health record system.

© 2026 Gohil SK et al. *JAMA Network Open*.
