# Supplementary material for: Improving Empiric Antibiotic Selection for Patients With Cancer Hospitalized With Infection: Secondary Analysis of the INSPIRE Cluster Randomized Trials
Source: JAMA Netw Open. 2026 Jun 10;9(6):e2616611. doi: 10.1001/jamanetworkopen.2026.16611 (PMC13254732; doi:10.1001/jamanetworkopen.2026.16611)
Supplement: Supplement 3. — Data Sharing Statement [file jamanetwopen-e2616611-s003.pdf]

Data Sharing Statement

Gohil. Improving Empiric Antibiotic Selection for Patients With Cancer Hospitalized With Infection. *JAMA Netw Open*. Published June 05, 2026.
doi:10.1001/jamanetworkopen.2026.16611

Data
Additional Information: NCT05423756, NCT05423743, NCT03697070, NCT03697096.
Data available: Yes
Data types: Data dictionary, Other (please specify)
Additional Information: Since the INSPIRE dataset includes over 900,000 admissions and represents a large majority of HCA Healthcare data, there are both patient confidentiality and proprietary concerns with the release of this dataset. HCA will maintain the study's analysis datasets in a protected data enclave for three years from the date of publication. Requests to analyze the data will be evaluated by HCA and a technical team to determine whether the proposed use is consistent with the original aims of the trial and is technically appropriate. Approved requesters will create executable analysis programs that will be executed by HCA-affiliated analysts. Programs may only generate summary level, not patient-level, data. A data dictionary and request form will be provided upon request. A processing fee will apply.
How to access data: Data sharing requests within three years of publication should be made to the first author at skgohil@hs.uci.edu for provision of the data dictionary and data request form.
When available: With publication

Supporting Documents
Document types: Other (please specify)
Additional Information: Trial protocols, statistical analysis plan.
How to access documents: See supplement 1 and 2 for each publication provided here:
https://jamanetwork.com/journals/jama/fullarticle/2817976
https://jamanetwork.com/journals/jama/fullarticle/2817975
https://jamanetwork.com/journals/jamainternalmedicine/fullarticle/2832776
https://jamanetwork.com/journals/jamasurgery/fullarticle/2832732
When available: With publication

Additional Information
Who can access the data: Data sharing will be permitted with researchers whose proposed use of the data has been approved. As noted above, only aggregate results of analyses will be provided.
Types of analyses: The scope of the request must be limited to the outcomes of the INSPIRE Pneumonia Trial.
Mechanisms of data availability: All data sharing requests will benefit from investigator and health system knowledge of the dataset. A processing fee will be assessed for all requested analyses to cover costs related to time and effort to respond to the request and a portion of costs to support the existence of this service.
