# Supplementary material for: Improving Empiric Antibiotic Selection for Patients With Cancer Hospitalized With Infection: Secondary Analysis of the INSPIRE Cluster Randomized Trials
Source: JAMA Netw Open. 2026 Jun 10;9(6):e2616611. doi: 10.1001/jamanetworkopen.2026.16611 (PMC13254732; doi:10.1001/jamanetworkopen.2026.16611)
Supplement: Supplement 2. — Trial Protocols and Statistical Analysis Plans [file jamanetwopen-e2616611-s002.pdf]

Protocols and Statistical Analysis Plans for INSPIRE Trials

(Originally published in primary manuscripts for each INSPIRE randomized clinical trial.)

Table of Contents

1. **INSPIRE Pneumonia Trial Protocol……………………………………………………………… .2**
    a. Original Toolkit – Routine Stewardship Group
    b. Original Toolkit – INSPIRE Stewardship Group
    c. Final Protocol – N/A, same as original protocol
    d. Summary of changes – N/A, no changes

2. **INSPIRE Pneumonia Trial Statistical Analysis Plan ………………………………………….. 144**
    a. Summary of Changes
    b. Original Statistical Plan – 9/19/18
    c. Final Statistical Plan – 6/3/20

3. **INSPIRE Urinary Tract Infection Trial Protocol ………………………………………………... 154**
    a. Original Toolkit – INSPIRE Stewardship Group
    b. Original Toolkit – Routine Stewardship Group
    c. Final Protocol – N/A, same as original protocol
    d. Summary of changes – N/A, no changes

4. **INSPIRE Urinary Tract Infection Trial Statistical Analysis Plan …………………………… 297**
    a. Summary of Changes
    b. Original Statistical Plan – 9/19/18
    c. Final Statistical Plan – 6/3/20

5. **INSPIRE Skin and Soft Tissue Infection Trial Statistical Analysis Plan…………………… 307**
    a. Summary of changes
    b. Original Statistical Plan – 6/29/22
    c. Final Statistical Plan – 3/29/24

6. **INSPIRE Skin and Soft Tissue Infection Trial Protocol……………………………………… 316**
    a. Original Trial Protocol and Implementation Toolkit – Routine Stewardship Group
    b. Original Trial Protocol and Implementation Toolkit – CPOE Bundle Group
    c. Final Trial Protocol and Implementation Toolkit – N/A, same as original protocol
    d. Summary of changes – N/A, no changes

7. **INSPIRE Abdominal Infection Trial Statistical Analysis Plan……………………………….. 404**
    a. Summary of changes
    b. Original Statistical Plan – 6/29/22
    c. Final Statistical Plan – 3/29/24

8. **INSPIRE Abdominal Infection Trial Safety Outcome Supplemental Methods…………… 411**
    a. Primary safety outcome measures
    b. Safety outcome sensitivity analyses

9. **INSPIRE Abdominal Infection Trial Protocol…………………………………………………… 415**
    a. Original Trial Protocol and Implementation Toolkit – Routine Stewardship Group
    b. Original Trial Protocol and Implementation Toolkit – CPOE Bundle Group
    c. Final Trial Protocol and Implementation Toolkit – N/A, same as original protocol
    d. Summary of changes – N/A, no changes

INSPIRE Pneumonia Trial
Trial Supplement 1

(Originally published in Gohil, et al. Stewardship Prompts to Improve Antibiotic Selection for Pneumonia: The INSPIRE Randomized Clinical Trial. JAMA. 2024;331(23):2007–2017. doi:10.1001/jama.2024.6248)

Table of Contents

1. **INSPIRE Pneumonia Trial Protocol**
    a. Original Toolkit – INSPIRE Stewardship Group
    b. Original Toolkit – Routine Stewardship Group
    c. Final Protocol – N/A, same as original protocol
    d. Summary of changes – N/A, no changes

2. **Statistical Analysis Plan**
    a. Summary of Changes
    b. Original Statistical Plan – 9/19/18
    c. Final Statistical Plan – 6/3/20

Arm 1:
Routine Stewardship

INSPIRE-ASP Pneumonia Trial
and
INSPIRE-ASP UTI Trial

Protocol & Toolkit Binder

Funded by the Centers for Disease Control and Prevention (CDC)

Welcome

Welcome to the **INSPIRE-ASP** (**IN**telligent **S**tewardship **P**rompts to **I**mprove **R**eal-time **E**mpiric **A**ntibiotic **S**election for **P**atients) Trials. The INSPIRE-ASP Pneumonia and INSPIRE-ASP Urinary Tract Infection (UTI) Trials are separate 2-Arm cluster-randomized trials of 59 HCA Healthcare hospitals to assess best practice in antibiotic stewardship. These trials are a partnership between HCA Healthcare and academic investigators who are federally funded by the Centers for Disease Control and Prevention (CDC).

Summary of Goals

Up to 40% of patients who are prescribed antibiotics in U.S. hospitals either do not require these drugs or receive inappropriate antibiotics for the condition being treated. Moreover, inappropriate antibiotics are often broad-spectrum, empirically prescribed due to concerns about infection with multidrug-resistant organisms (MDROs), despite the low risk of MDRO infection in the vast majority of clinical settings. This is the case for patients admitted with pneumonia and urinary tract infections (UTIs), two of the most common conditions for antibiotic use. This trial will compare two strategies to determine best practices for empiric antibiotic selection. Arm 1, Routine Care, will continue antibiotic stewardship practices according to current facility strategies. Arm 2, Smart Prompt Alert Intervention, will implement a precision medicine smart prompt using a computerized physician order entry (CPOE) alert that provides clinicians with a patient's probability of MDRO infection and recommends a standard-spectrum antibiotic choice in patients admitted for pneumonia or UTIs in non-critical care units. This trial will provide essential information to clinicians in real-time to support and optimize standard-spectrum empiric antibiotic use.

HCA

Funded by the Centers for Disease Control and Prevention (CDC)

INSPIRE-ASP Pneumonia and UTI Trials

***IN**telligent **S**tewardship **P**rompts to **I**mprove **R**eal-time **E**mpiric **A**ntibiotic **S**election for **P**atients*

The **INSPIRE-ASP Trials** are a **two-in-one** set of cluster-randomized trials at HCA Healthcare (HCA) hospitals to assess best practice in antibiotic stewardship for patients admitted to non-critical care units for pneumonia (PNA) and urinary tract infection (UTI). It tests the use of smart prompts to optimize prescribing and improve patient outcomes. Participating hospitals will be randomized to:

- **Arm 1 – Routine Care**
- **Arm 2 – Smart Prompt**
  Precision medicine smart prompt using a computerized physician order entry (CPOE) alert that gives a ***patient-specific risk estimate of antibiotic resistant infection*** and recommends standard-spectrum antibiotics in patients admitted for PNA or UTI who are found to have low risk (<10%) for drug resistant infection

Rationale

- Most non-ICU adult patients hospitalized with pneumonia or UTI are low risk for multidrug-resistant organisms (MDROs) and do not need broad-spectrum antibiotics.
- Identifying patients at low vs high risk of MDROs can decrease use of extended-spectrum antibiotics, reduce selection of resistant bacteria, and reduce *C. difficile* infections.

CPOE Details

- MDRO risk estimates are based upon patient-specific risk factors and local antibiograms
- We assessed demographics, comorbidities, MDRO history, prior antibiotic use, prior nursing home stay, and a variety of laboratory values to create a prediction algorithm
- Antibiotic recommendations are based on MDRO risk estimates and approved by trial steering committee, HCA leadership, and CDC experts.
- Recommendations support proper antibiotic choice but do not replace clinical judgment

Outcomes

- Primary: Empiric use of extended-spectrum antibacterials (first 3 days of admission)
- Secondary: Empiric use of (a) vancomycin and (b) antipseudomonal antibacterials
- Safety: Antibacterial escalation, ICU transfer, and length-of-stay
- Outcomes for other manuscripts: *C. difficile* infection, MDRO prevalence, cost-analysis

Anticipated Timeline

- 6 Month Phase In: October 2018 – March 2019
- 15 Month Intervention: April 2019 – June 2020

QUESTIONS? Study Investigators & Coordinators
HCAINSPIRE@gmail.com
844-522-4677

HCA

Funded by the Centers for Disease Control and Prevention (CDC)

INSPIRE-ASP – ARM 1

HCA ED and Inpatient Indication Screens: Clinician Work Flow

The requirement for providing an indication for antibiotic use is a national standard for antibiotic stewardship. HCA Healthcare (HCA) originally released antibiotic indication screens in 2017 and are currently implemented in inpatient areas throughout HCA facilities nationwide. This year, updates were made in these indication screens to allow the Emergency Department (ED) to be included.

Beginning **October 1, 2018** our hospital will launch HCA Antibiotic Indication Screens **in the Emergency Department.**

Ordering an antibiotic will now need an indication when ordered in the ED and on inpatient wards. This document provides details on the HCA antibiotic indication screen clinician workflow with relevant screen shots.

1. **HCA Antibiotic Indication Screens** – physicians will be asked to choose an indication for each antibiotic they order.

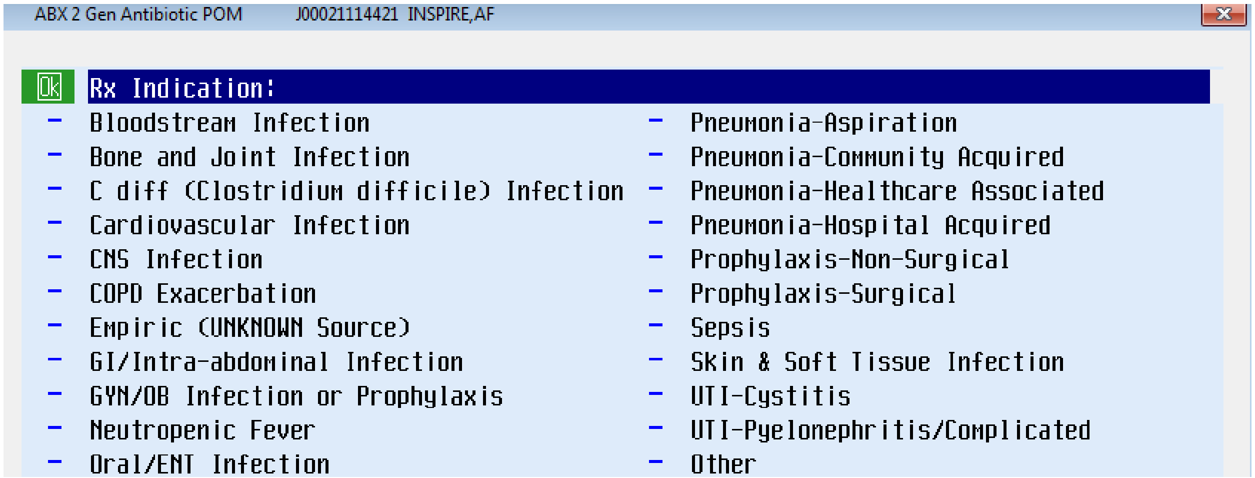
ABX 2 Gen Antibiotic POM J00021114421 INSPIRE,AF

Ok Rx Indication:

- Bloodstream Infection
- Bone and Joint Infection
- C diff (Clostridium difficile) Infection
- Cardiovascular Infection
- CNS Infection
- COPD Exacerbation
- Empiric (UNKNOWN Source)
- GI/Intra-abdominal Infection
- GYN/OB Infection or Prophylaxis
- Neutropenic Fever
- Oral/ENT Infection
- Pneumonia-Aspiration
- Pneumonia-Community Acquired
- Pneumonia-Healthcare Associated
- Pneumonia-Hospital Acquired
- Prophylaxis-Non-Surgical
- Prophylaxis-Surgical
- Sepsis
- Skin & Soft Tissue Infection
- UTI-Cystitis
- UTI-Pyelonephritis/Complicated
- Other

↓

2. If “Empiric Unknown” or “Sepsis” are selected, then you will be asked what type of infection you suspect the patient may have and whether it is suspected to be community, healthcare, or hospital acquired:

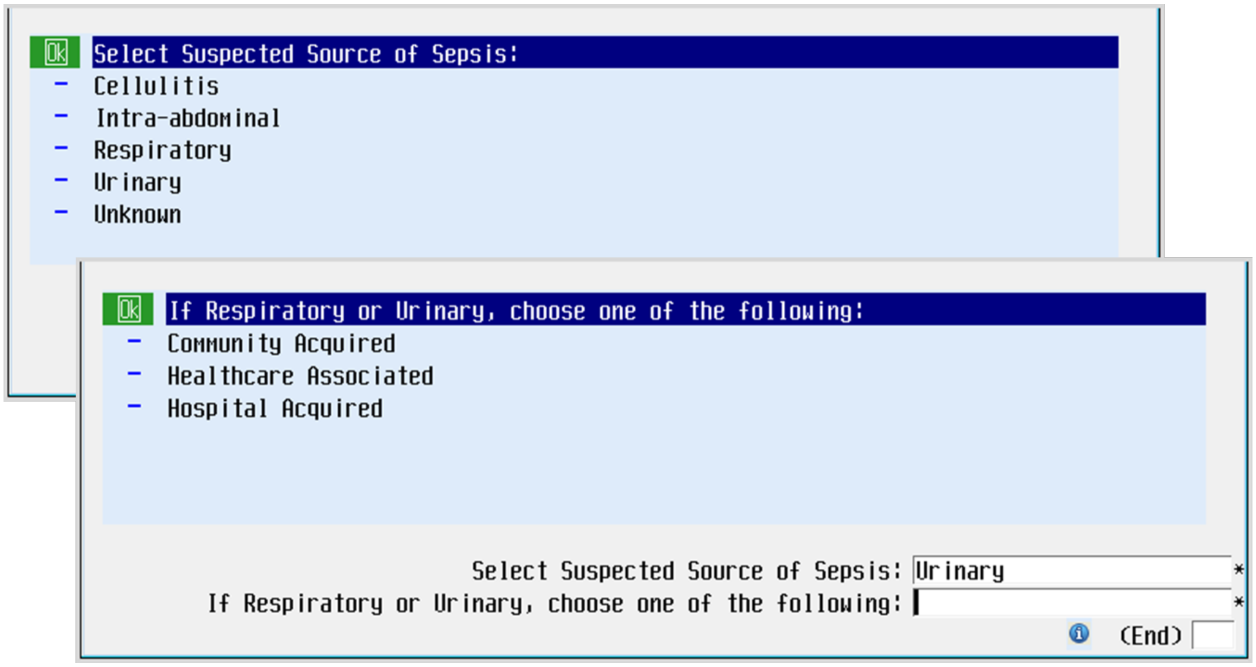

3. If “Pneumonia-Aspiration” or “UTI” are selected, you will be asked whether it is suspected to be community, healthcare, or hospital acquired:

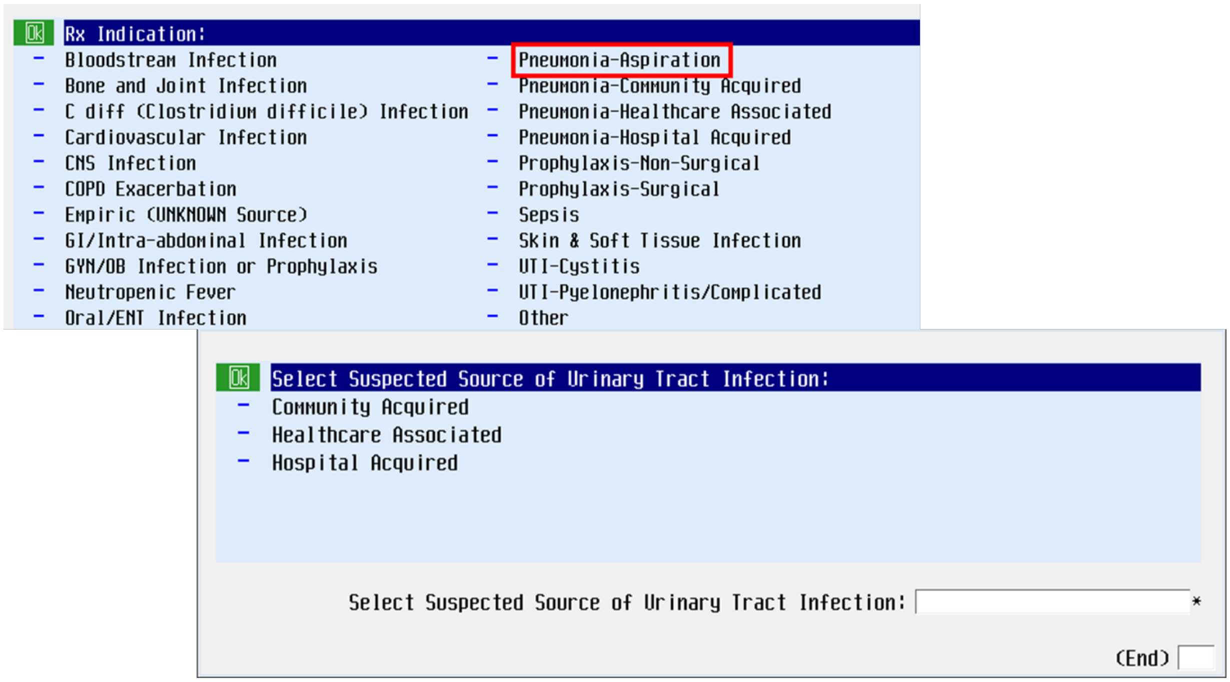

4. There is one additional screen that will be required for Arm 1 INSPIRE facilities that is unique to the trials. This screen asks the ED physician whether the patient is ICU-bound and is ONLY

triggered for patients in the ED in whom extended-spectrum antibiotics are prescribed for the following indications: pneumonia, UTI, empiric (unknown), or sepsis.

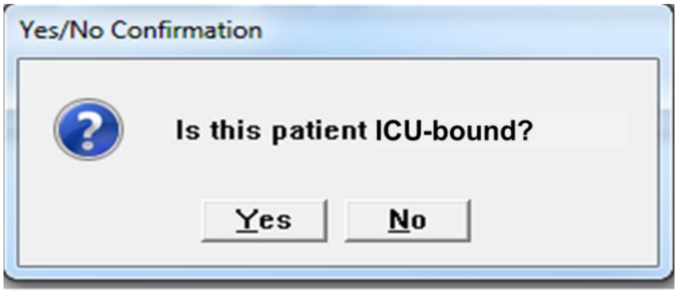

This final screen was necessary for the INSPIRE Arm 2 facilities to ensure adequate exclusion of ICU patients in the intervention arm. It was included for Arm 1 facilities to ensure that both arms are balanced and can be compared properly.

INSPIRE-ASP Trial

INtelligent Stewardship Prompts to Improve Real-time Empiric Antibiotic Selection for Patients

Arm 1 Coaching Call
September 13, 2018

Agenda

- INSPIRE Trials overview
- HCA Meaningful Use indication screens
- Polling questions
- Question & answer

INSPIRE-ASP Trial Arms

Arm 1: Routine Care – 30 Hospitals

- Standard-of-care antibiotic stewardship

Arm 2: Smart Prompt Alert Intervention – 29 Hospitals

- CPOE precision medicine alerts for patient-specific MDRO risk
- Recommends appropriate antibiotic choice
- Provider feedback reports

INSPIRE-ASP Population & Primary Outcome

Includes

- Adults admitted or being admitted for **PNA or UTI**
- ED and non-ICU units
- First 3 days of admission

Excludes

- ICU and pediatric patients

Intervention

- MDRO risk estimate calculates when antibiotic ordered

Primary Outcome

- Extended-spectrum (ES) antibiotics: first 3 days of admission

Trial Timeline

- Randomization: **March, 2018**
- Site visits: **Summer-Fall, 2018**
- Intervention launch: **October 1, 2018**
- Phase In: **6 months**
- Intervention: **15 months**

HCA Meaningful Use Indication Screens

- National guidance to institute meaningful use indications
  - CDC NHSN issued declaration of readiness
  - Leapfrog metric
- HCA indication screens released to inpatient areas 2017
  - Widely implemented throughout HCA
  - Well accepted by inpatient physicians
  - Planned for release in ED enterprise wide
- In Arm 2, INSPIRE CPOE prompts trigger off indication screens
  - Indication screens will be active in both ED and inpatient
- To ensure Arms 1 and 2 are equally balanced, the indication screens will need to be active in both study arms
  - ED Indication screens go live **October 1st** for BOTH arms

INSPIRE BETTER SAFER ANTIBIOTIC USE

HCA Indication Screens Trigger CPOE Smart Prompt

- INSPIRE prompts triggered (Arm 2 only) based on indication:
  - **PNA**, **UTI**, **Sepsis/Empiric** – triggers additional screen asking if possible respiratory or urinary source

- **Indication screens** will be live in **both Arm 1 and Arm 2**

ABX 2 Gen Antibiotic POM J00021114421 INSPIRE,AF

Ok **Rx Indication:**

– Bloodstream Infection	– Pneumonia-Aspiration
– Bone and Joint Infection	– Pneumonia-Community Acquired
– C diff (Clostridium difficile) Infection	– Pneumonia-Healthcare Associated
– Cardiovascular Infection	– Pneumonia-Hospital Acquired
– CNS Infection	– Prophylaxis-Non-Surgical
– COPD Exacerbation	– Prophylaxis-Surgical
– Empiric (UNKNOWN Source)	– Sepsis
– GI/Intra-abdominal Infection	– Skin & Soft Tissue Infection
– GYN/OB Infection or Prophylaxis	– UTI-Cystitis
– Neutropenic Fever	– UTI-Pyelonephritis/Complicated
– Oral/ENT Infection	– Other

HCA Indication Screens: Empiric or Sepsis Indications

- If “Empiric Unknown” or “Sepsis” are selected:

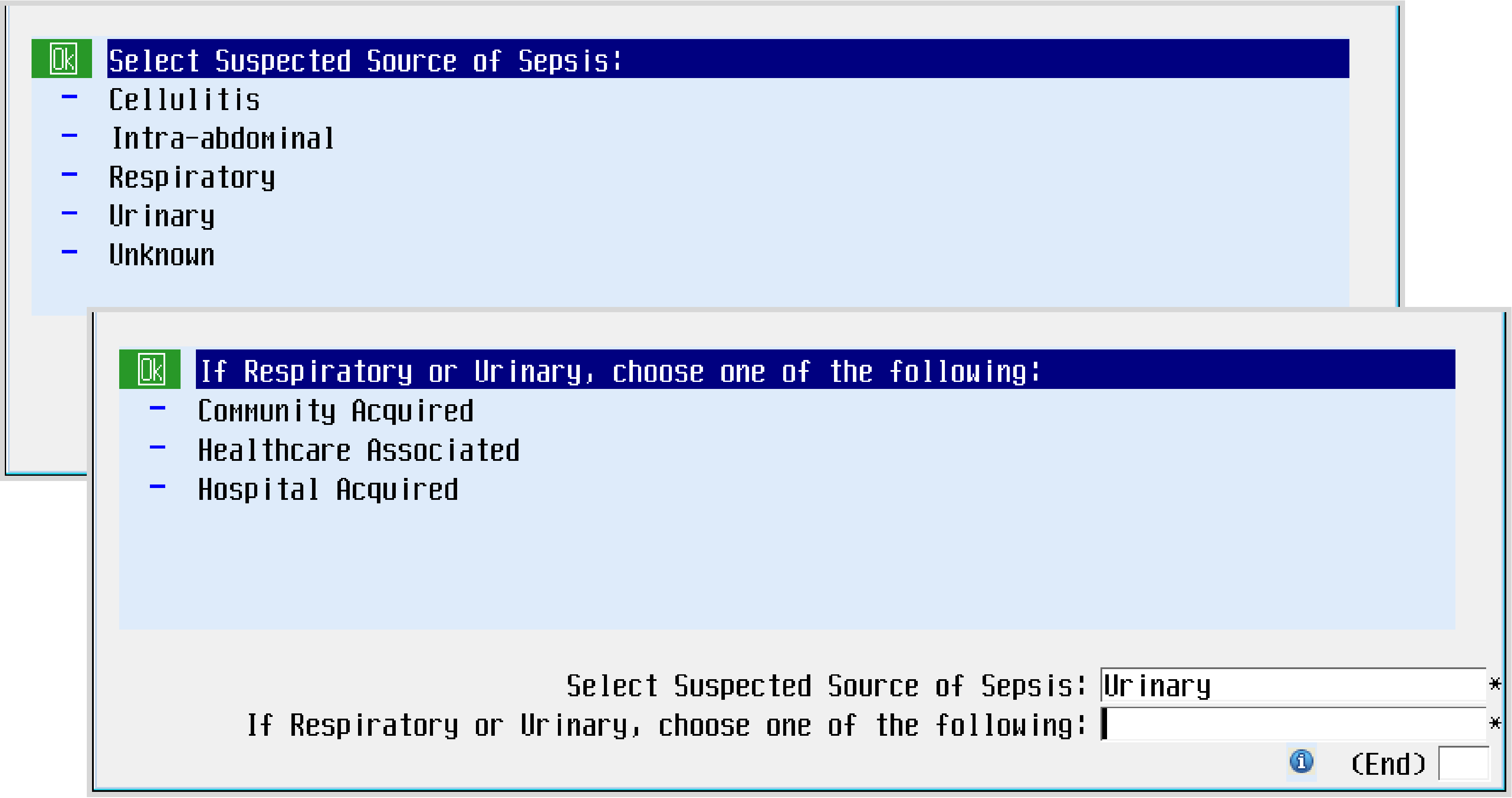

HCA Indication Screens: Empiric or Sepsis Indications

- If "Pneumonia-Aspiration" or "UTI" are selected:

Ok Rx Indication:

- Bloodstream Infection
- Bone and Joint Infection
- C diff (Clostridium difficile) Infection
- Cardiovascular Infection
- CNS Infection
- COPD Exacerbation
- Empiric (UNKNOWN Source)
- GI/Intra-abdominal Infection
- GYN/OB Infection or Prophylaxis
- Neutropenic Fever
- Oral/ENT Infection
- Pneumonia-Aspiration
- Pneumonia-Community Acquired
- Pneumonia-Healthcare Associated
- Pneumonia-Hospital Acquired
- Prophylaxis-Non-Surgical
- Prophylaxis-Surgical
- Sepsis
- Skin & Soft Tissue Infection
- UTI-Cystitis
- UTI-Pyelonephritis/Complicated
- Other

Ok Select Suspected Source of Urinary Tract Infection:

- Community Acquired
- Healthcare Associated
- Hospital Acquired

Select Suspected Source of Urinary Tract Infection: *

(End)

INSPIRE Workflow: ED Patients Going to ICU

- **For ED patients**, if an INSPIRE indication is chosen, then asks if patient is likely to go to the ICU
  - If YES → no further screens
  - If NO → INSPIRE CPOE screens continue (Arm 2 only)
- Will be live in **both Arm 1 and Arm 2**

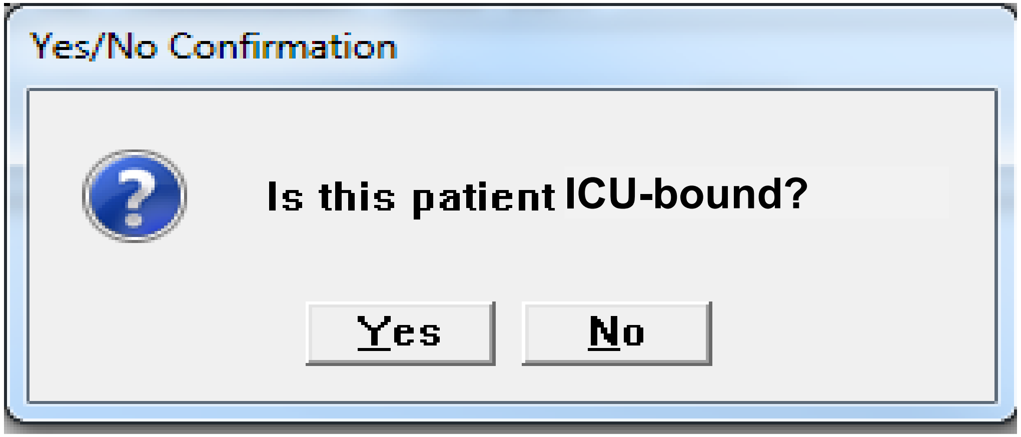

Study Champion Priorities - Arm 1

- Ensure HCA Meaningful Use screen activation in ED and inpatient areas
- Assess facility needs for successful launch
- Inform and educate clinicians locally
- Identify ED or hospitalist champions and other leaders to help physicians get on board

Integrating Smart Prompt into Existing Order Sets

- Smart prompts designed to integrate into existing order sets
- HCA pharmacy IT team can help troubleshoot
- Study champions should assess for redundancy in physician workflow
- Strategies for successful integration if conflicts found:
  - Separate ICU order sets from non-ICU
  - Indications can be pre-populated if using order set

Clinician Outreach Planned

- Webinars
  - Audience: hospitalists, ED physicians, nurse practitioners, physician assistants, pharmacists – any prescribers
  - At least two separate dates/time slots will be offered
- Clinician email draft
  - INSPIRE team will reach out to you with a draft email for you to send out to clinicians
- HCA meaningful use indication screen workflow summary

Will the Indication Screens Impact ED Core Measures?

- Indication Screens **do not** affect "time-to-antibiotics"
  - Does not impact whether antibiotics should be used
  - Supports decision for extended vs standard spectrum antibiotic

- Time-to-antibiotics metric definition concern from the field has been brought to leadership

Ongoing Reminder: Competing Interventions

- Facilities are fully expected to continue stewardship activities
- Examples of activities directly impacting primary outcomes:
  - New empiric antibiotic selection strategy
  - New de-escalation initiative in the ED or empiric period
  - New rapid diagnostic tool for pneumonia or UTI

Email HCAINSPIRE@gmail.com if considering initiatives/new interventions → steering committee can help to evaluate if activity could impact trial outcomes

INSPIRE
BETTER SAFER ANTIBIOTIC USE

Arm 1 - Key Role in INSPIRE-ASP

Your **key role** is to *maintain* U.S. gold standard for antibiotic stewardship practices against which our intervention will be compared. Avoid changing current practices in the empiric period. Avoid new initiatives specifically targeting empiric antibiotic use.

- Implement HCA indication screens
- Maintain best practices for stewardship
- Continue all current stewardship practices
- Inform INSPIRE-ASP team if considering new initiatives HCAINSPIRE@gmail.com → steering committee can help to evaluate if activity could impact trial outcomes

Contact Information

- **Email**: HCAINSPIRE@gmail.com
- **Phone**: (844) 522-4677
- Atlas study link:

http://connect.medcity.net/documents/60285481/0/INSPIRE-ASP+Trial+Coaching+Call+%231+Arm+2+-+4-11-18.pptx/8082a768-c974-d149-b876-0dbc96b07106

NOTE: Emails and phone numbers approved by HCA Security

Do NOT send PHI to these accounts

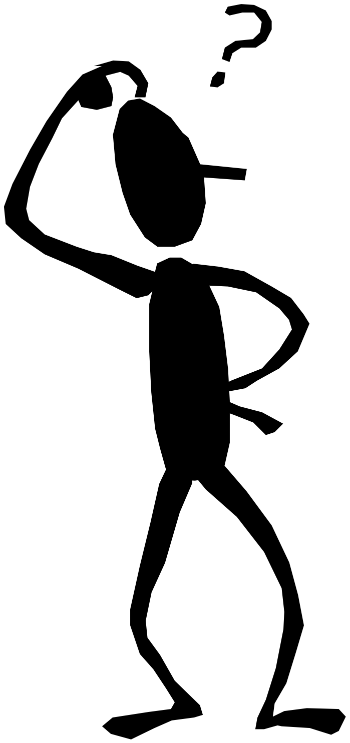

Open Floor Q&A

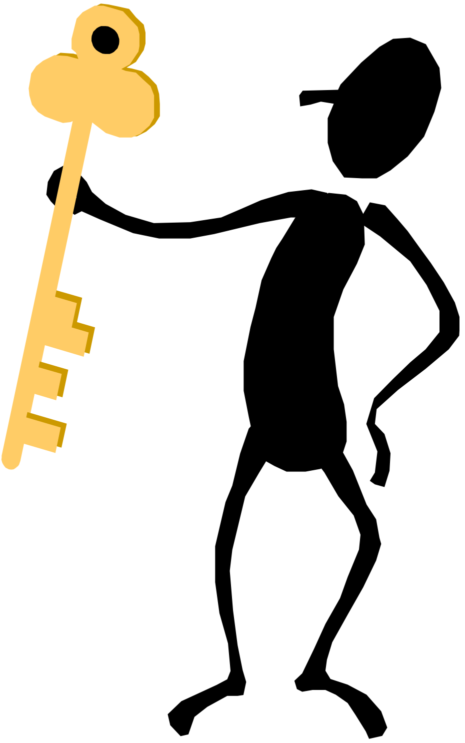

Arm 2:
INSPIRE Stewardship

INSPIRE-ASP Pneumonia Trial
and
INSPIRE-ASP UTI Trial

Protocol & Toolkit Binder

Funded by the Centers for Disease Control and Prevention (CDC)

INtelligent Stewardship Prompts to Improve Real-time Empiric Antibiotic Selection for Patients

Arm 2 – Smart Prompt

Toolkit Binder
Table of Contents

Document	Tab
Welcome & Summary of Goals …………………………………………	Inside Front Cover
Study Investigators……………………………………………………	Inside Front Cover
Project Summary………………………………………………………………………………	1
Phone Matrix……………………………………………………………………………………	2
Roles & Responsibilities…………………………………………………………………	3
Antibiotic Selection Protocol…………………………………………………………	4
Study Champion Materials………………………………………………………………	5
Clinician Education Materials…………………………………………………………	6
CPOE Clinician Work Flow ………………………………………………………………	7
Clinician Quarterly Progress Report Example ………………………………………	8
Clinician Poster………………………………………………………………	Inside Back Cover

HCA

Funded by the Centers for Disease Control and Prevention (CDC)

Welcome

Welcome to the **INSPIRE-ASP** (**IN**telligent **S**tewardship **P**rompts to **I**mprove **R**eal-time **E**mpiric **A**ntibiotic **S**election for **P**atients) Trials. The INSPIRE-ASP Pneumonia and INSPIRE-ASP Urinary Tract Infection (UTI) Trials are separate 2-Arm cluster-randomized trials of 59 HCA Healthcare hospitals to assess best practice in antibiotic stewardship. These trials are a partnership between HCA Healthcare and academic investigators who are federally funded by the Centers for Disease Control and Prevention (CDC).

Summary of Goals

Up to 40% of patients who are prescribed antibiotics in U.S. hospitals either do not require these drugs or receive inappropriate antibiotics for the condition being treated. Moreover, inappropriate antibiotics are often broad-spectrum, empirically prescribed due to concerns about infection with multidrug-resistant organisms (MDROs), despite the low risk of MDRO infection in the vast majority of clinical settings. This is the case for patients admitted with pneumonia and urinary tract infections (UTIs), two of the most common conditions for antibiotic use. This trial will compare two strategies to determine best practices for empiric antibiotic selection. Arm 1, Routine Care, will continue antibiotic stewardship practices according to current facility strategies. Arm 2, Smart Prompt Alert Intervention, will implement a precision medicine smart prompt using a computerized physician order entry (CPOE) alert that provides clinicians with a patient's probability of MDRO infection and recommends a standard-spectrum antibiotic choice in patients admitted for pneumonia or UTIs in non-critical care units. This trial will provide essential information to clinicians in real-time to support and optimize standard-spectrum empiric antibiotic use.

HCA

Funded by the Centers for Disease Control and Prevention (CDC)

Study Investigators and Stakeholders

INSPIRE
BETTER SAFER ANTIBIOTIC USE

University of California Irvine
Shruti Gohil, MD MPH, Lead Investigator
Susan Huang, MD MPH, Senior Investigator

Harvard Medical School/Harvard Pilgrim Health Care Institute
Ed Septimus, MD FACP FIDSA FSHEA
Meghan Baker, MD PhD
Richard Platt, MD MSc

Brigham & Women's Hospital
David Kubiak, Pharm D

University of Massachusetts Amherst
Ken Kleinman, ScD

Rush University
Robert Weinstein, MD
Mary Hayden, MD

Centers for Disease Control and Prevention
Sujan Reddy, MD MSc
Arjun Srinivasan, MD FSHEA
John Jernigan, MD MS
Lauri Hicks, DO
Melinda Neuhauser, Pharm D MPH FCCP FASHP

HCA Healthcare
Mandelin Cooper, Pharm D BCPS, Director, Clinical Pharmacy
Hayley Burgess, Pharm D, AVP Clinical Pharmacy Services
Risa Rahm, Pharm D CPHIMS, Director, Medication Management & Clin. Pharmacy Informatics
Will Cooper, Pharm D CPHIMS, Clinical Pharmacy Manager
Caren Spencer-Smith, Research Clinical Data Analyst, Patient Safety and Research
Julia Moody, MS SM (ASCP), Clinical Director, Infection Prevention
Russell Poland, PhD, AVP, Research and Scientific Communications
Karla Miller, Pharm D, VP Pharmacy Services
Ken Sands, MD MPH, VP Chief Epidemiologist and Patient Safety Officer
Jonathan Perlin, MD PhD, Chief Medical Officer

HCA

Funded by the Centers for Disease Control and Prevention (CDC)

INSPIRE-ASP Pneumonia and UTI Trials

INtelligent Stewardship Prompts to Improve Real-time Empiric Antibiotic Selection for Patients

The **INSPIRE-ASP Trials** are a **two-in-one** set of cluster-randomized trials at HCA Healthcare (HCA) hospitals to assess best practice in antibiotic stewardship for patients admitted to non-critical care units for pneumonia (PNA) and urinary tract infection (UTI). It tests the use of smart prompts to optimize prescribing and improve patient outcomes. Participating hospitals will be randomized to:

- **Arm 1 – Routine Care**
- **Arm 2 – Smart Prompt**

  Precision medicine smart prompt using a computerized physician order entry (CPOE) alert that gives a ***patient-specific risk estimate of antibiotic resistant infection*** and recommends standard-spectrum antibiotics in patients admitted for PNA or UTI who are found to have low risk (<10%) for drug resistant infection

Rationale

- Most non-ICU adult patients hospitalized with pneumonia or UTI are low risk for multidrug-resistant organisms (MDROs) and do not need broad-spectrum antibiotics.
- Identifying patients at low vs high risk of MDROs can decrease use of extended-spectrum antibiotics, reduce selection of resistant bacteria, and reduce *C. difficile* infections.

CPOE Details

- MDRO risk estimates are based upon patient-specific risk factors and local antibiograms
- We assessed demographics, comorbidities, MDRO history, prior antibiotic use, prior nursing home stay, and a variety of laboratory values to create a prediction algorithm
- Antibiotic recommendations are based on MDRO risk estimates and approved by trial steering committee, HCA leadership, and CDC experts.
- Recommendations support proper antibiotic choice but do not replace clinical judgment

Outcomes

- Primary: Empiric use of extended-spectrum antibacterials (first 3 days of admission)
- Secondary: Empiric use of (a) vancomycin and (b) antipseudomonal antibacterials
- Safety: Antibacterial escalation, ICU transfer, and length-of-stay
- Outcomes for other manuscripts: *C. difficile* infection, MDRO prevalence, cost-analysis

Anticipated Timeline

- 6 Month Phase In: October 2018 – March 2019
- 15 Month Intervention: April 2019 – June 2020

QUESTIONS? Study Investigators & Coordinators
HCAINSPIRE@gmail.com
844-522-4677

HCA

Funded by the Centers for Disease Control and Prevention (CDC)

Stewardship Champion Roles and Responsibilities

Pharmacy & Physician Stewardship Champions will partner with the INSPIRE-ASP investigative team and are expected to:

1. Join trial coaching calls
2. Actively engage and educate clinicians on value and safety of the INSPIRE-ASP Trials
3. Review clinician quarterly progress reports and encourage improvements
4. Address clinician outliers in a supportive, knowledgeable way
5. Set reachable local targets and improvement goals
6. Report interventions or activities that may impact trial outcomes

HCA

Funded by the Centers for Disease Control and Prevention (CDC)

INSPIRE-ASP Empiric Antibiotic Selection Protocol

The **INSPIRE-ASP Pneumonia and Urinary Tract Infection (UTI) trials** use a computerized physician order entry (CPOE) alert to provide clinicians with (1) patient-specific risk estimates for infection due to an antibiotic-resistant bacteria and (2) recommendations on the standard-spectrum empiric antibiotic selection based on individualized risk estimates. This intervention will improve the use of standard-spectrum antibiotics for non-ICU patients at low risk of having pneumonia or UTI due to an antibiotic-resistant bacteria.

This document provides pharmacists and clinicians with an overview of the protocol by which antibiotic selection should occur when INSPIRE-ASP CPOE alerts are activated at your facility.

Protocol for Empiric Antibiotic Selection

1. **The CPOE alert is activated when:**
   a. Clinicians order an extended-spectrum antibiotic(s) for pneumonia or UTI in non-ICU patients within the first 3 days of admission, including in the emergency department.
   b. If standard-spectrum antibiotics are ordered (e.g. ceftriaxone or ceftriaxone/azithromycin), no prompt will be triggered.
2. **The CPOE alert provides clinicians with patient-specific risk estimates for antibiotic-resistant infection.**
   a. Risk estimates are calculated for the antibiotic-resistant organism(s) targeted by the antibiotic ordered (Table 1).
      i. Example 1: When a clinicians orders vancomycin for pneumonia, the CPOE alert will calculate the risk that the patient has pneumonia due to MRSA.
      ii. Example 2: When a clinicians orders cefepime for pneumonia, the CPOE alert will calculate the risk that the patient has pneumonia due to *Pseudomonas*.
      iii. Example 3: When a clinicians orders linezolid for UTI, the CPOE alert will calculate the risk that the patient has UTI due to MRSA or VRE.
      iv. Example 4: When a clinicians orders meropenem for UTI, the CPOE alert will calculate the risk that the patient has UTI due to ESBL or other resistant gram negative bacteria.
3. **Clinicians will be provided recommendations on the appropriateness of extended-spectrum antibiotics based upon the patient's calculated risk.**
   a. If the patient's risk for antibiotic resistant pneumonia or UTI is <10%, then the risk is considered low and the CPOE alert will recommend ceftriaxone as the standard empiric antibiotic.

b. If the patient's risk for MDRO pneumonia or UTI is ≥10%, the prompt will automatically allow completion of the extended-spectrum antibiotic order without any prompts.

c. CPOE recommendations are **<u>not intended to supplant clinician judgment</u>**. Patients who are neutropenic, decompensating, or otherwise at high risk for complications or drug resistant infections should be treated as clinically appropriate.

d. Clinicians will always have the option to override the CPOE alert recommendation after indicating the reason for the override.

4. **Prescriber acceptance with CPOE alert recommendations will be monitored by the stewardship team/study champions.**

   a. Clinician Feedback reports, including the number and reason for CPOE recommendation overrides, will be provided to study champions.

   b. Study champions will review acceptance and provide prescribers with written and/or verbal feedback.

Table 1: Extended-Spectrum Antibiotics and Pathogen Included in the CPOE Alert

<table>
<tr><th rowspan="2">Antibiotic Ordered</th><th colspan="2">Pathogen Included in Risk Estimate</th></tr>
<tr><th>PNEUMONIA</th><th>UTI</th></tr>
<tr><td>Daptomycin</td><td>Not Recommended for PNA</td><td rowspan="2">MRSA, VRE</td></tr>
<tr><td>Linezolid[1]</td><td>MRSA</td></tr>
<tr><td>Vancomycin (IV only)</td><td colspan="2" rowspan="2">MRSA</td></tr>
<tr><td>Ceftaroline</td></tr>
<tr><td>Aztreonam</td><td colspan="2" rowspan="4">Pseudomonas</td></tr>
<tr><td>Cefepime</td></tr>
<tr><td>Ceftazidime</td></tr>
<tr><td>Piperacillin/Tazobactam</td></tr>
<tr><td>Ertapenem</td><td colspan="2">ESBL,
GNR Resistant to cephalosporins and penicillins</td></tr>
<tr><td>Doripenem</td><td colspan="2" rowspan="4">ESBL,
GNR+Pseudomonas Resistant to cephalosporins and penicillins</td></tr>
<tr><td>Imipenem</td></tr>
<tr><td>Meropenem</td></tr>
<tr><td>Ceftolozane/Tazobactam</td></tr>
<tr><td>Ceftazidime/Avibactam</td><td colspan="2" rowspan="4">No Screen - Requires ID Approval[2]</td></tr>
<tr><td>Tigecycline</td></tr>
<tr><td>Colistin</td></tr>
<tr><td>Polymyxin B</td></tr>
</table>

1. Both oral and intravenous formulations of drugs in this category are included.
2. For anti-CRE medications, all models show <10% patient risk for having a highly drug resistant pathogen warranting the use of these drugs. Therefore, patient specific risk estimate will not be calculated and a static CPOE screen will be developed that will recommend avoiding empiric use without consultation with antibiotic stewardship team or infectious diseases.
3. Newly released ES medications require ID approval and will be considered for inclusion in the CPOE prompts on a case by case basis
<u>Note</u>: INSPIRE-ASP smart prompt intervention excludes the following routes of administration: inhaled, otic, ophthalmic, or irrigation.
Abbreviations:

MRSA - Methicillin Resistant *Staphylococcus aureus*
VRE – Vancomycin Resistant *Enterococci*
MDR- Multi-Drug Resistant
ESBL – Extended Spectrum Beta Lactamase producer
CRE – Carbapenem Resistant *Enterobacteriaceae*
GNR – Gram Negative Rod

HCA
Funded by the Centers for Disease Control and Prevention (CDC)

INSPIRE-ASP Trials: Arm 2

Study Champion Materials

This section includes:

- Study Champion Checklist
- Abbreviated FAQs
- Study Champion FAQs
- Verbal Order Checklist

INSPIRE-ASP Study Champion Intervention Checklist

As your facility's Study Champion for INSPIRE-ASP, there are a few key things you can do to ensure the CPOE Smart Prompt launch goes smoothly. Here is a quick checklist:

☐ Assess existing order sets

- ✓ Review any sepsis, pneumonia, or UTI order sets currently in place
- ✓ Evaluate for potential conflicts or redundancies with CPOE smart prompts
- ✓ Work with local and corporate IT to resolve
- ✓ Examples include:
  1. Sepsis order set contains only extended-spectrum antibiotics as options for pneumonia. CPOE smart prompt alert will recommend ceftriaxone when MDRO risk is low. This may be seen by clinicians as potentially conflicting recommendations.

     Possible solution(s): (a) Add ceftriaxone to list of acceptable antibiotics in sepsis order sets. (b) Make two separate order sets, one for ICU patients and one for non-ICU patients.

☐ Identify key leaders

- ✓ Hospitalist leader, ED leader, Pharmacy staff
- ✓ Enlist their help to educate their peers, give presentations, and help you disseminate education and field questions from clinicians

☐ Get the word out and educate frontline clinicians/prescribers

- ✓ Present INSPIRE at medical staff/faculty meetings
- ✓ Post flyers/posters in physician lounges
- ✓ "Meet-and-greet" clinicians on the floors with the one-page INSPIRE summary
- ✓ Disseminate emails to all clinicians
- ✓ Encourage clinicians to attend study webinars or access information on Atlas
- ✓ Educate nurses or pharmacists that may be taking verbal orders

☐ Once launched, check if the CPOE smart prompt is working as planned

- ✓ The prompt fires only for extended-spectrum antibiotics
- ✓ The prompt does <u>not</u> fire in the ICU or after hospital day 3
- ✓ Clinicians are not choosing indications other than Pneumonia or UTI to circumvent the smart prompt
- ✓ Verbal orders are placed in Meditech/Order Management, NOT by paper or the "PHA" system

INSPIRE-ASP Abbreviated FAQ
For Clinician Prescribers and Pharmacists

What are the INSPIRE-ASP Trials?

The INSPIRE-ASP (**IN**telligent **S**tewardship **P**rompts to **I**mprove **R**eal-time **E**mpiric **A**ntibiotic **S**election for **P**atients) Trials are two separate cluster-randomized trials of HCA Healthcare (HCA) hospitals to test an antibiotic stewardship intervention for pneumonia and urinary tract infection (UTI).

- **Arm 1** – **Routine Care**
- **Arm 2** – **CPOE Smart Prompt Alert Intervention**
    1. Provides patient-specific risk estimate for pneumonia or UTI due to a multi-drug resistant organism (MDRO) when an antibiotic is ordered for these indications
    2. Recommends standard-spectrum antibiotics in non-ICU pneumonia or UTI patients with low (<10%) MDRO risk

When will the CPOE smart prompt fire?

- Prompts fire when extended-spectrum antibiotics are ordered within 3 days of admission
- Only for adults in ED or **non**-intensive care units (ICU) admitted for pneumonia or UTI

Which antibiotics are considered extended-spectrum?

Category	Extended-Spectrum Antibiotics for INSPIRE CPOE Alerts
**Anti-MRSA**	Ceftaroline, Daptomycin, Linezolid[1], Vancomycin[2]
**Anti-VRE**	Daptomycin, Linezolid[1]
**Anti-Pseudomonas**	Aztreonam, Cefepime, Ceftazidime, Piperacillin/Tazobactam
**Anti-ESBL**	Ertapenem, Doripenem, Imipenem, Meropenem, Ceftolozane/Tazobactam
**Anti-CRE**	Ceftazidime/Avibactam, Colistin, Polymixin B, Tigecycline

[1]**Both oral and intravenous (IV) formulations**
[2]**IV formulation only**

How is the CPOE prompt "smart"?

The CPOE smart prompt uses your specific patient medical record data to calculate MDRO risk. Risk estimate are tailored to the antibiotic selected. For example:

- If ceftriaxone ordered → no prompt fires at all (standard-spectrum antibiotic)
- If vancomycin ordered → returns patient's MRSA risk
- If cefepime or piperacillin/tazobactam ordered → returns *Pseudomonas* risk
- If linezolid ordered → returns MRSA and VRE risk
- If meropenem ordered → returns ESBL + resistant gram negative bacteria
- Recommends ceftriaxone when MDRO risk is low (<10%)

What are the CPOE risk estimates based on?

The INSPIRE study team assessed over 536,000 patients admitted to HCA hospitals over 3 years (May, 2014- March, 2017). We evaluated **(1) your hospital antibiogram** for pneumonia and UTI patients and **(2) patient risk factors of MDRO or *Pseudomonas* infection**, including demographics (age, gender, race, ethnicity, insurance status), comorbid conditions, history of MDRO, history of prior HCA hospital, ED, or nursing home visit, prior antibiotics, and lab values.

Using a special statistical method, we identified which risk factors were most often found in patients who developed pneumonia or UTI with an MDRO or *Pseudomonas*. Using these risk factors (which are different for every MDRO), the CPOE smart prompt gives the patient's **absolute risk** for infection with the MDRO(s) targeted by the antibiotic ordered.

How far back does the CPOE smart prompt look for MDRO history?

The CPOE smart prompt mines your facility's microbiology data in Meditech as far back as 2012. It will capture a patient's MDRO history even if there is no electronic MDRO flag, but it will not capture MDRO history or cultures from an outside hospital.

How quickly can the CPOE smart prompt account for positive cultures? What if a culture was sent recently?

The CPOE smart prompt captures cultures resulted in Meditech at your facility 7 or more days ago; it currently cannot integrate new cultures resulted in the empiric period. For example, if a culture was sent upon admission, this will not be included in a risk estimate when ordering extended-spectrum antibiotics on hospital day 3. We encourage close monitoring of all new microbiology results as they become available and selection of antibiotics accordingly.

How does the CPOE smart prompt exclude ICU patients?

1. Does not fire if a patient is located in the ICU.
2. For emergency department, smart prompt asks clinicians if the patient is ICU-bound and does not fire if clinician responds "yes".
3. If smart prompt fires and patient is going to the ICU, clinicians can override the prompt.

Will INSPIRE impact ED sepsis core measures?

The CPOE smart prompt **does NOT** affect "time to antibiotics" because it does not impact whether antibiotics should be used but rather gives real-time information to help decide whether extended-spectrum or standard-spectrum antibiotics should be used. The recommended standard-spectrum antibiotic, ceftriaxone, is compliant with core measures.

Will patients need to give informed consent?

No. This study has been approved as a minimal risk study and will be implemented as a quality improvement (QI) protocol through the existing infrastructure of your hospital's antibiotic stewardship programs. The assigned protocol will be approved through your usual clinical/leadership committees and become hospital guidance on appropriate empiric antibiotic use.

Whom can I contact for more information?

INSPIRE-ASP Study Team:
HCAINSPIRE@gmail.com, (844)-522-4677

Frequently Asked Questions (FAQ)
Pharmacy & Clinician Study Champions

This FAQ specifically addresses questions that facility Study Champions may have. Please see the "Clinician Prescriber" FAQ for key details on the intervention.

What are the INSPIRE-ASP Trials?

The INSPIRE-ASP (**IN**telligent **S**tewardship **P**rompts to **I**mprove **R**eal-time **E**mpiric **A**ntibiotic **S**election for **P**atients) Trials are two separate cluster-randomized trials of HCA Healthcare (HCA) hospitals to assess best practice in antibiotic stewardship for non-ICU patients admitted with pneumonia and urinary tract infection (UTI). Hospitals will be randomized to:

- **Arm 1** – **Routine Care**
- **Arm 2 – CPOE Smart Prompt Alert Intervention**
  Precision medicine smart prompt using a computerized physician order entry (CPOE) alert that supports antibiotic prescribing with:
  1. Patient-specific estimate of risk for pneumonia or UTI due to a multi-drug resistant organism (MDRO) or *Pseudomonas*
  2. Recommendations for standard spectrum antibiotics in non-ICU patients admitted for pneumonia or UTI with low MDRO risk (<10%)

What are the outcomes of the INSPIRE-ASP Trials?

The goal is to identify a best practice strategy for choosing empiric (within 3 calendar days) antibiotics based on a patient's risk for MDRO infection. Outcomes include:

- Primary: Empiric use of extended-spectrum antibacterials (first 3 days of admission)
- Secondary: Empiric use of (a) vancomycin and (b) antipseudomonal antibacterials
- Safety: Antibacterial escalation, ICU transfer, and length-of-stay
- Outcomes for other manuscripts: *C. difficile* infection, MDRO prevalence, cost-analysis

Which patients are included?

Only adults admitted to a non-ICU ward with pneumonia or UTI who are ordered to receive an extended-spectrum antibiotic within 3 days of admission will be included in the smart prompt alerts. The INSPIRE smart prompt alerts are programmed to not fire for patients younger than 18 years or who are in an ICU location**.** Patients admitted from jail or prison are **not** included in this study.

Is INSPIRE-ASP one trial or two?

The INSPIRE-ASP trials will be rolled out together but are actually two separate trials: the **INSPIRE-ASP Pneumonia Trial** and **INSPIRE-ASP UTI Trial**. Because there are important differences between the two infection syndromes, our smart prompt will need to provide separate risk estimates for pneumonia and UTI. All analyses will need to be done separately to properly evaluate the impact of our intervention. However, all other aspects of the CPOE smart prompt intervention will be rolled out together.

Why will the study focus on Pneumonia and UTI?

Pneumonia and UTI are the top two most common reasons for infection-related admission to the hospital and account for the majority of antibiotic use nationally.

Why is antibiotic stewardship a major contributor to patient safety?

Unnecessary extended-spectrum antibiotic use can increase a patient's risk for MDRO and *Clostridium difficile* infections (CDI). These infections are often harder to manage due to limited treatment options. Both CDI and MDRO infections are associated with higher risks for complication, longer length-of-stay, readmission, and death.

How would patient-specific alerts promote better, safer antibiotic use?

Uncertainty about the risk that an individual patient is infected with an MDRO drives overuse of extended-spectrum antibiotics. Current diagnostic tests do not provide immediate information about which pathogen is responsible for infection. This uncertainty drives clinicians to cover all possible organisms, including MRSA, *Pseudomonas* species, and ESBLs, even when a patient's risk for these MDROs may be very low. Providing clinicians with real-time estimates of a patient's risk for infection due to an MDRO would allow more informed antibiotic choices.

What are the patient specific risk estimates based on?

The INSPIRE study team assessed over 536,000 patients admitted at all HCA hospitals over 3 years (2014-2016) within Meditech. We evaluated (1) your hospital antibiogram for pneumonia and UTI patients and (2) patient risk factors of MDRO infection, including:

- Demographics (age, gender, race, ethnicity, insurance status)
- Comorbid conditions (e.g., diabetes, lung disease, renal failure)
- History of ED visit or admission to an HCA hospital
- History of nursing home admission
- History of antibiotics received during an ED or inpatient admission
- History of MDRO (screening or clinical cultures at your facility)
- Lab values (e.g., BUN, creatinine)

Using a special statistical method, we identified which risk factors were most often found in patients who developed pneumonia or UTI with an MDRO. Using these risk factors (which differ for every MDRO), the CPOE smart prompt gives the patient's **absolute risk** for infection with the MDRO(s) targeted by the antibiotic ordered.

Note that INSPIRE calculates your facility antibiogram as the proportion of MDRO or pseudomonas among patients admitted with PNA or UTI, which is different than the traditional way most microbiology labs calculate this.

For more detailed information, you can review an audio-enabled presentation on the HCA Atlas website, entitled "How the MDRO Risk Estimates Work": http://connect.medcity.net/web/hca-research-academic-affairs/inspire

How far back does the CPOE smart prompt look for MDRO history?

The CPOE smart prompt mines your facility's microbiology data in Meditech as far back as 2012. It will capture a patient's MDRO history even if there is no electronic MDRO flag, but it will not capture MDRO history or cultures from an outside hospital.

How quickly can the CPOE smart prompt account for positive cultures? What if a culture was sent recently?

The CPOE smart prompt captures cultures resulted in Meditech at your facility 7 or more days ago; it currently cannot integrate new cultures resulted in the empiric

period. For example, if a culture was sent upon admission, this will not be included in a risk estimate when ordering extended-spectrum antibiotics on hospital day 3. We encourage close monitoring of all new microbiology results as they become available and selection of antibiotics accordingly.

Will risk estimates be validated after the intervention starts?

Our risk estimates will be revalidated at regular intervals during the intervention. Significant changes in select variables (e.g., changes in your antibiogram) will be updated within the CPOE alert. Note that our estimates are based on 3 years of data over 140 HCA hospitals, it is unlikely that additional accrual of trial time will change risk factors that have already been identified.

What makes the INSPIRE CPOE prompt "smart"?

The INSPIRE smart prompts are automated, real-time alerts built into an electronic medical record that are programmed to return an **individual patient's specific risk** for MDRO or *Pseudomonas* when an extended-spectrum antibiotic is ordered. This risk estimate is tailored to each antibiotic selected. For example:

- If ceftriaxone ordered -→ no prompt (recommended)
- If vancomycin ordered → returns MRSA risk
- If cefepime or piperacillin/tazobactam ordered → returns *Pseudomonas* risk
- If linezolid ordered → returns MRSA and VRE risk
- If meropenem ordered → returns ESBL and other resistant gram negative bacteria risk

What about antibiotics administered through non-systemic routes, such as inhalation, irrigation, or topical?

The INSPIRE smart prompts are only triggered when an extended-spectrum antibiotic is ordered for pneumonia, UTI, or sepsis/empiric source due to respiratory or urinary source. This process would automatically exclude otic, ophthalmic, or topical antibiotics. Antibiotics administered through inhalation (e.g., ceftazidime) or irrigation (e.g., vancomycin use in bladder irrigation) are excluded from INSPIRE smart prompts.

What if someone takes a verbal order for antibiotics?

The CPOE smart prompt will **only fire in Meditech**. It will not fire in the "PHA" system. We ask all clinical staff who take verbal orders (including "vancomycin per pharmacy dosing" orders) to place them within Meditech. This means that you will have to ask clinicians (1) what infection the antibiotic is meant to treat and (2) whether the physician would agree to standard spectrum antibiotics if the risk is found to be low (<10%). Please see the "Verbal Order Checklist for Pharmacists and Nurses" that will help guide how verbal orders can be best handled.

What will be required of participating sites?

Study champions will be asked to:

- Join trial coaching calls
- Engage and educate clinicians on value and safety of the INSPIRE-ASP Trials
- Review clinician quarterly progress reports and encourage improvements
- Address clinician outliers in a supportive, knowledgeable way
- Set reachable local targets and improvement goals
- Report interventions or activities that may impact trial outcomes

Please reference the "Study Champion Intervention Checklist" of key things you can do to ensure the INSPIRE CPOE smart prompt launches successfully at your facility.

How do we avoid competing interventions and how are these defined?

Participating facilities are fully expected to continue their stewardship activities and initiatives but should avoid activities that may directly impact the trial's primary outcome. Such activities are anticipated to be rare.

The following are examples of activities that do **NOT** directly conflict with this study:

- Surveillance and monitoring of antibiotic use
- De-escalation strategies after empiric period has passed (after hospital day 4)
- Using Vigilanz for automatic dosing

If already in place during baseline (Summer 2016 - Fall 2018), the intervention is not considered in conflict.

Examples of interventions that may directly conflict with the trial include:

- A new stewardship strategy that aims to reduce the use of extended-spectrum antibiotics in the Emergency Department (ED) or within 3 days of admission.
- A new real-time antibiotic de-escalation strategy in the ED/within 3 calendar days
- A trial of a new rapid diagnostic tool for pneumonia or UTI that would be used in the empiric period to guide antibiotic therapy within 3 days of admission.
- Ordering antibiotics through Patient Keeper

If a new intervention is thought to be in direct conflict with the study outcomes, we would ask participating facilities to disclose these plans to the research team for further discussion.

Will CPOE acceptance be tracked?

Yes, the investigative team will partner with HCA to provide you with quarterly clinician and hospital progress reports on a monthly basis. This will contain a rolling quarter's worth of data on how often clinicians order extended-spectrum antibiotics for pneumonia or UTI, how often recommendations for standard antibiotics is accepted, and if overridden, what was chosen as the reason. All data will be collected via the centralized Clinical Data Warehouse and will be provided to you.

What will be the process for incorporating smart prompt alerts into the electronic medical record system?

Smart prompt alerts will be developed by the HCA corporate IT team, including programming and initial beta testing. Corporate IT will provide a template for local IT teams to implement in hospitals assigned to the intervention arm.

Will this intervention affect our formulary antibiotic choices?

No. This intervention, including the smart prompt alerts, will focus on guiding clinicians towards optimal antibiotic class choices, not specific drugs or manufacturers.

Will patients need to give informed consent?

No. This study has been approved as a minimal risk study and will be implemented as a quality improvement (QI) protocol through the existing infrastructure of your hospital's antibiotic stewardship programs. The assigned protocol will be approved

through your usual clinical/leadership committees and become hospital guidance on standard spectrum empiric antibiotic use.

Has this study been approved by the Institutional Review Board (IRB)?

Yes. Harvard Pilgrim's IRB will provide ethical oversight for these trials and serve as the central IRB. Both INSPIRE-ASP PNA and UTI trials have been approved by the Harvard IRB. Your hospital has submitted all ceding and reliance agreements necessary to grant oversight by the central IRB at Harvard. HCA's corporate compliance and regulatory affairs will help provide support for this endeavor.

What is the timeline?

- In-person site visits – Summer-Fall 2018
- IT CPOE smart prompt alert implementation – Fall 2018
- Intervention launch – October 1, 2018
- Phase-in – 6 months
- Intervention – 15 months

Who comprises the study team?

These trials are a collaborative between HCA Healthcare and academic partners at the University of California Irvine, Harvard Pilgrim Health Care/Harvard Medical School, and Rush University. The trials are funded through a contract with Centers for Disease Control and Prevention (CDC).

Whom can I contact for more information?

INSPIRE-ASP Study Team
Lead Investigator – Shruti Gohil, MD, MPH, UC Irvine
HCAINSPIRE@gmail.com, (844)-522-4677

Edward Septimus, MD, FACP, Senior Lecturer HPHCI
eseptimus@gmail.com, (281) 714-5689

Hayley Burgess, PharmD, AVP Clinical Pharmacy Services, HCA
Hayley.Burgess@hcahealthcare.com, (615) 344-1857

Verbal Order Checklist for Pharmacists and Nurses

Background

The INSPIRE-ASP CPOE smart prompt intervention will encourage compliance with national guidance for standard-spectrum antibiotics (e.g., ceftriaxone) for pneumonia and UTI. Ordering extended-spectrum antibiotics (e.g., vancomycin, cefepime) for pneumonia or UTI in the ED or non-ICU wards within 3 days of admission will activate a computerized physician order entry (CPOE) smart prompt alert that will use the patient's Meditech data to provide:

1. Patient's risk for pneumonia or UTI due to antibiotic resistant bacteria
2. Recommendation for standard-spectrum antibiotics when risk is low (<10%)

Taking Verbal Orders for Inpatient Extended-Spectrum Antibiotics

The INSPIRE-ASP CPOE prompts apply **ONLY to extended-spectrum antibiotics** ordered on **non-ICU patients** hospitalized for **pneumonia or UTI** within **3 days of admission**.

- The prompt does **NOT** fire for standard-spectrum antibiotics.
- A list of extended-spectrum antibiotics is provided in Table 1 on the following page.

When taking verbal orders for extended-spectrum antibiotics, we generally recommend that you stay on the phone with the ordering clinician while entering orders. If you are unable to place the order directly at the time of verbal order, use the following questions to get key information you will need when placing the order:

1. **Check if patient has been <u>hospitalized for 4 days</u> or more or is <u>in the ICU</u>? If yes to either** → *no further physician questions needed. CPOE will **NOT** fire.*
2. **Is patient in the Emergency Department (ED)?**
    a. **If in ED** → ask if patient is ICU-bound
        i. **If ICU-bound** → no further questions
        ii. If <u>NOT</u> ICU-bound → proceed to Step 3

3. **IF extended-spectrum antibiotic being ordered within 3 days of admission:**
   a. **Ask prescriber what indication/infection the antibiotic is being used to treat (**Remind prescriber that choosing an indication is part of HCA's meaningful use stewardship initiative).
   b. If prescriber chooses any of the following indications, **proceed to step 4**:
      - Pneumonia
      - UTI
      - Sepsis – due to PNA or UTI
      - Empiric (unknown) source – respiratory or urinary source
4. **Remind prescriber that extended-spectrum antibiotics in this patient will trigger the INSPIRE smart prompt and will give the patient's specific risk for infection due to MDRO or *Pseudomonas*. The following suggested script can be used or modified as needed:**
   a. "Just a reminder, Dr. ___, our hospital has an antibiotic protocol for pneumonia and UTI. The computer will calculate this patient's risk of an antibiotic-resistant pneumonia/UTI. If the risk is low (<10%) then ceftriaxone will be recommended. Should I plan to follow this recommendation?"
      i. **If yes** → no further questions.
      ii. **If no** → ask the prescriber what the reason for overriding the recommendation would be:
         - Allergy
         - Neutropenia
         - Positive MDRO culture this admission or outside hospital
         - Patient will go to the ICU
         - Other (ask prescriber to specify)

Table 1: Extended-Spectrum Antibiotics That Will Trigger INSPIRE-ASP CPOE Alerts

Category	Extended-Spectrum Antibiotics for INSPIRE CPOE Alerts
**Anti-MRSA**	Ceftaroline, Daptomycin, Linezolid[1], Vancomycin[2]
**Anti-VRE**	Daptomycin, Linezolid[1]
**Anti-Pseudomonas**	Aztreonam, Cefepime, Ceftazidime, Piperacillin/Tazobactam
**Anti-ESBL**	Ertapenem, Doripenem, Imipenem, Meropenem, Ceftolozane/Tazobactam
**Anti-CRE**	Ceftazidime/Avibactam, Colistin, Polymixin B, Tigecycline

[1]Both oral and intravenous (IV) formulations
[2]IV formulation only

INSPIRE-ASP Trials: Arm 2

Clinician Education Materials

This section includes:

- Abbreviated FAQs
- Clinician FAQs
- Essential Clinician Facts
- Do’s and Don’ts
- Clinician Education Presentation
- Clinician Poster

INSPIRE-ASP Abbreviated FAQ
For Clinician Prescribers and Pharmacists

What are the INSPIRE-ASP Trials?

The INSPIRE-ASP (**IN**telligent **S**tewardship **P**rompts to **I**mprove **R**eal-time **E**mpiric **A**ntibiotic **S**election for **P**atients) Trials are two separate cluster-randomized trials of HCA Healthcare (HCA) hospitals to test an antibiotic stewardship intervention for pneumonia and urinary tract infection (UTI).

- **Arm 1** – **Routine Care**
- **Arm 2** – **CPOE Smart Prompt Alert Intervention**
  1. Provides patient-specific risk estimate for pneumonia or UTI due to a multi-drug resistant organism (MDRO) when an antibiotic is ordered for these indications
  2. Recommends standard-spectrum antibiotics in non-ICU pneumonia or UTI patients with low (<10%) MDRO risk

When will the CPOE smart prompt fire?

- Prompts fire when extended-spectrum antibiotics are ordered within 3 days of admission
- Only for adults in ED or **non**-intensive care units (ICU) admitted for pneumonia or UTI

Which antibiotics are considered extended-spectrum?

Category	Extended-Spectrum Antibiotics for INSPIRE CPOE Alerts
**Anti-MRSA**	Ceftaroline, Daptomycin, Linezolid[1], Vancomycin[2]
**Anti-VRE**	Daptomycin, Linezolid[1]
**Anti-Pseudomonas**	Aztreonam, Cefepime, Ceftazidime, Piperacillin/Tazobactam
**Anti-ESBL**	Ertapenem, Doripenem, Imipenem, Meropenem, Ceftolozane/Tazobactam
**Anti-CRE**	Ceftazidime/Avibactam, Colistin, Polymixin B, Tigecycline

[1]Both oral and intravenous (IV) formulations
[2]IV formulation only

How is the CPOE prompt “smart”?

The CPOE smart prompt uses your specific patient medical record data to calculate MDRO risk. Risk estimate are tailored to the antibiotic selected. For example:

- If ceftriaxone ordered → no prompt fires at all (standard-spectrum antibiotic)
- If vancomycin ordered → returns patient’s MRSA risk
- If cefepime or piperacillin/tazobactam ordered → returns *Pseudomonas* risk
- If linezolid ordered → returns MRSA and VRE risk
- If meropenem ordered → returns ESBL + resistant gram negative bacteria
- Recommends ceftriaxone when MDRO risk is low (<10%)

What are the CPOE risk estimates based on?

The INSPIRE study team assessed over 536,000 patients admitted to HCA hospitals over 3 years (May, 2014- March, 2017). We evaluated **(1) your hospital antibiogram** for pneumonia and UTI patients and **(2) patient risk factors of MDRO or *Pseudomonas* infection**, including demographics (age, gender, race, ethnicity, insurance status), comorbid conditions, history of MDRO, history of prior HCA hospital, ED, or nursing home visit, prior antibiotics, and lab values.

Using a special statistical method, we identified which risk factors were most often found in patients who developed pneumonia or UTI with an MDRO or *Pseudomonas*. Using these risk factors (which are different for every MDRO), the CPOE smart prompt gives the patient's **absolute risk** for infection with the MDRO(s) targeted by the antibiotic ordered.

How far back does the CPOE smart prompt look for MDRO history?

The CPOE smart prompt mines your facility's microbiology data in Meditech as far back as 2012. It will capture a patient's MDRO history even if there is no electronic MDRO flag, but it will not capture MDRO history or cultures from an outside hospital.

How quickly can the CPOE smart prompt account for positive cultures? What if a culture was sent recently?

The CPOE smart prompt captures cultures resulted in Meditech at your facility 7 or more days ago; it currently cannot integrate new cultures resulted in the empiric period. For example, if a culture was sent upon admission, this will not be included in a risk estimate when ordering extended-spectrum antibiotics on hospital day 3. We encourage close monitoring of all new microbiology results as they become available and selection of antibiotics accordingly.

How does the CPOE smart prompt exclude ICU patients?

1. Does not fire if a patient is located in the ICU.
2. For emergency department, smart prompt asks clinicians if the patient is ICU-bound and does not fire if clinician responds "yes".
3. If smart prompt fires and patient is going to the ICU, clinicians can override the prompt.

Will INSPIRE impact ED sepsis core measures?

The CPOE smart prompt **does NOT** affect "time to antibiotics" because it does not impact whether antibiotics should be used but rather gives real-time information to help decide whether extended-spectrum or standard-spectrum antibiotics should be used. The recommended standard-spectrum antibiotic, ceftriaxone, is compliant with core measures.

Will patients need to give informed consent?

No. This study has been approved as a minimal risk study and will be implemented as a quality improvement (QI) protocol through the existing infrastructure of your hospital's antibiotic stewardship programs. The assigned protocol will be approved through your usual clinical/leadership committees and become hospital guidance on appropriate empiric antibiotic use.

Whom can I contact for more information?

INSPIRE-ASP Study Team:
HCAINSPIRE@gmail.com, (844)-522-4677

Frequently Asked Questions
Clinician Prescribers

What are the INSPIRE-ASP Trials?

The INSPIRE-ASP (**IN**telligent **S**tewardship **P**rompts to **I**mprove **R**eal-time **E**mpiric **A**ntibiotic **S**election for **P**atients) Trials are two separate cluster-randomized trials of HCA Healthcare (HCA) hospitals to assess best practice in antibiotic stewardship for non-ICU patients with pneumonia and urinary tract infection (UTI). Hospitals will be randomized to:

- **Arm 1** – **Routine Care**
- **Arm 2** – **CPOE Smart Prompt Alert Intervention**
  Precision medicine smart prompt using a computerized physician order entry (CPOE) alert that supports antibiotic prescribing with:
  1. Patient-specific estimate of risk for pneumonia or UTI due to a multi-drug resistant organism (MDRO) or *Pseudomonas*
  2. Recommendations for standard-spectrum antibiotics in non-ICU patients admitted for pneumonia or UTI with low MDRO risk (<10%)

What are the outcomes of the INSPIRE-ASP Trials?

The goal is to identify a best practice strategy for choosing empiric antibiotics (within 3 calendar days) based on a patient's risk for MDRO infection. Outcomes include:

- Primary: Empiric extended-spectrum antibiotic use (first 3 hospital days)
- Secondary: Post-empiric extended-spectrum antibiotic use (hospital day 4 to discharge), fluoroquinolone use, antibiotic costs, MDRO and *C. difficile* rates
- Safety outcomes: length of stay, 30-day readmissions, antibiotic escalations, and ICU transfers

Which patients are included in the intervention?

Only adults admitted to a non-ICU ward with pneumonia or UTI who are ordered to receive an extended-spectrum antibiotic within 3 days of admission will be included in the smart prompt alerts. The INSPIRE smart prompt alerts are programmed to not fire for patients younger than 18 years or who are in an ICU location. Patients admitted from jail or prison will **not** be included in this study.

What is the rationale for the INSPIRE-ASP Trials?

The Centers for Disease Control (CDC) estimates that 20-40% of extended-spectrum antibiotics in hospitals are unnecessary. One study showed that 33% of pneumonia patients received MRSA treatment even though only 2% were confirmed to have MRSA by culture. Empiric use of standard-spectrum antibiotics is expected to reduce overall extended-spectrum antibiotic exposure during the hospital stay.

How will this help me make better antibiotic choices?

Current approaches to choosing antibiotics should take into account an individual patient's risk for an MDRO infection to decide if extended-spectrum antibiotics can be avoided. However it is cumbersome for clinicians to efficiently and accurately estimate MDRO risk for patients when selecting antibiotics. Current hospital antibiograms do not give clinicians information about a specific patient's risk. The INSPIRE CPOE smart prompt alert will provide clinicians with a patient-specific MDRO risk estimate in real-time to optimize standard antibiotic selection.

How will this help my patient and the community?

Minimizing exposure to extended-spectrum antibiotics can reduce your patient's risk for future MDRO infections, *C. difficile*, and hospital readmission. MDRO infections are harder to treat due to limited treatment options and are associated with higher risks for complication, longer length-of-stay, and death. This trial is expected to decrease extended-spectrum antibiotic use at 29 HCA hospitals across the nation. If successful, findings from this trial have the potential for changing empiric antibiotic selection and stewardship practices at a national level.

What does the INSPIRE-ASP computerized physician order entry (CPOE) smart prompt alert do?

The CPOE smart prompt alert provides clinicians with a patient's probability of having a pneumonia or UTI due to an MDRO or *Pseudomonas*. This estimate is based upon

patient-specific characteristics and hospital antibiotic susceptibility. The alert will give recommendations on standard-spectrum empiric antibiotics in patients admitted for pneumonia or urinary tract infection. The alert is triggered only when an extended-spectrum antibiotic is ordered in non-critical care units.

Is INSPIRE-ASP one trial or two?

The INSPIRE-ASP trials will be rolled out together but are actually two separate trials: the **INSPIRE-ASP Pneumonia Trial** and **INSPIRE-ASP UTI Trial**. Because there are important differences between the two infection syndromes, our smart prompt will need to provide separate risk estimates for pneumonia and UTI. All analyses will need to be done separately to properly evaluate the impact of our intervention. However, all other aspects of the CPOE smart prompt intervention will be rolled out together.

Why will the study focus on pneumonia and UTI?

Pneumonia and UTI are the top two most common reasons for infection-related admission to the hospital and account for the majority of antibiotic use nationally.

What makes the INSPIRE CPOE prompt "smart"?

The INSPIRE smart prompts are automated, real-time alerts built into an electronic medical record that are programmed to return an **individual patient's specific risk** for MDRO or *Pseudomonas* when an extended-spectrum antibiotic is ordered. This risk estimate is tailored to each antibiotic selected. For example:

- If ceftriaxone ordered → no prompt (recommended)
- If vancomycin ordered → returns MRSA risk
- If cefepime or piperacillin/tazobactam ordered → returns *Pseudomonas* risk
- If linezolid ordered → returns MRSA and VRE risk
- If meropenem ordered → returns ESBL and other resistant gram negative bacteria risk

How does the CPOE smart prompt work?

The smart prompt alert will look at your patient's electronic chart for specific risk factors in real-time and calculate the risk that the patient you are seeing has an infection due to an MDRO. If this risk is low (<10%), then the smart prompt will recommend standard-spectrum antibiotics. For example, if you order cefepime on a patient with pneumonia and the smart prompt calculates a <10% risk of *Pseudomonas*, then the smart prompt will recommend using ceftriaxone instead (Figure 1). Choosing

the replace button will automatically take you to the ceftriaxone ordering screen. If the risk is high (>=10%), then no additional screens will fire and you will automatically continue ordering cefepime.

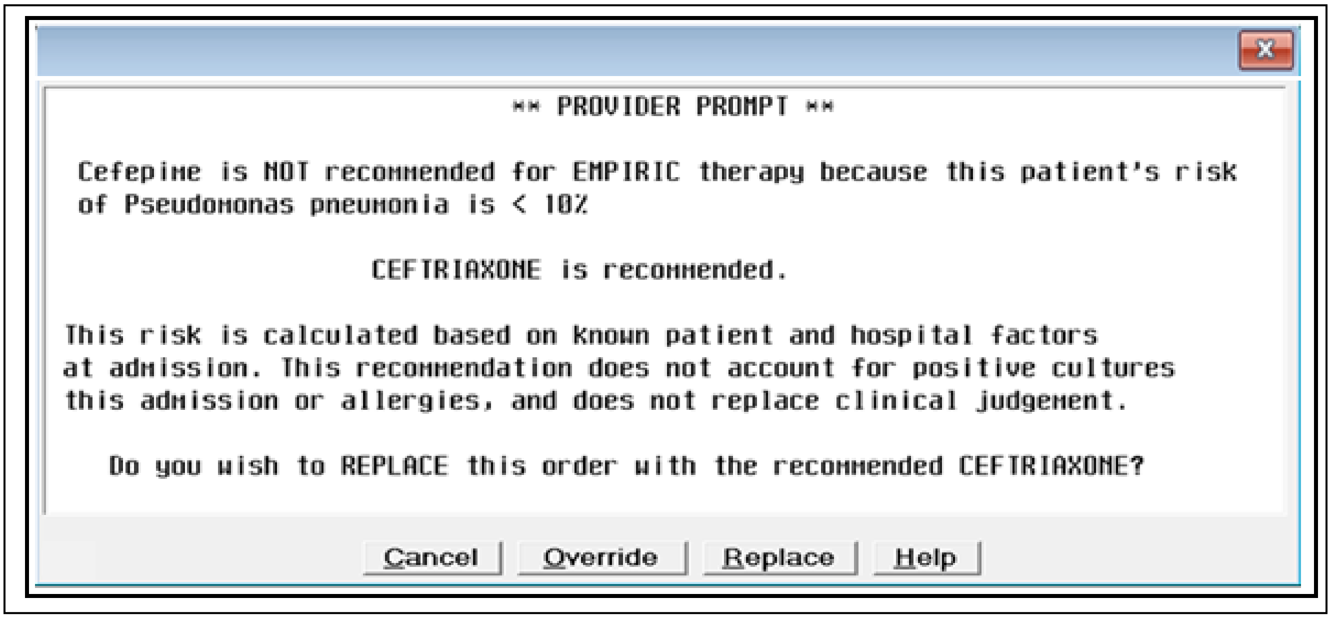

Figure 1. Example screenshot of CPOE smart prompt recommendation triggered when cefepime is ordered for patient with pneumonia.

What if I need to override the CPOE recommendation?

The INSPIRE-ASP smart prompts are meant to support, not replace clinical judgment and there may be good reasons why you may want to override the recommended antibiotic. If you choose to override, you will be asked to provide a reason (Figure 2). You will then continue with your extended-spectrum antibiotic order.

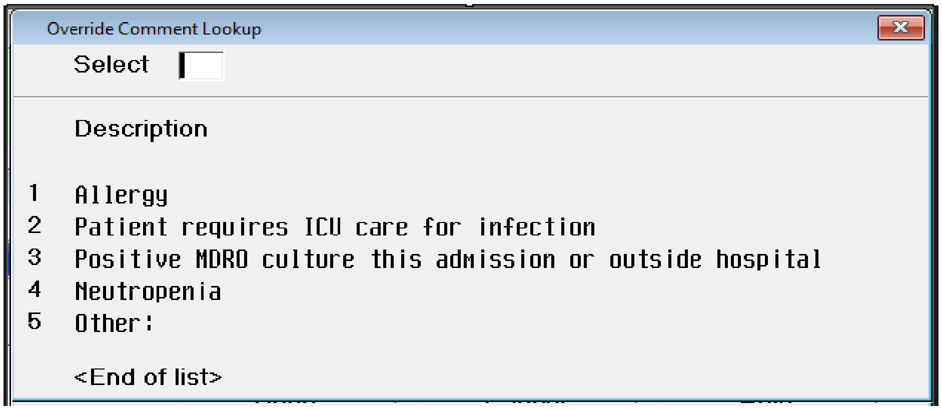

Figure 2. Example screenshot – prescribers will be asked to provide a reason for override.

How are the smart prompts activated?

The CPOE smart prompts are activated when clinicians choose the following indications for extended-spectrum antibiotics: Pneumonia, UTI, sepsis due to pneumonia or UTI, or empiric (unknown) source (Figure 3).

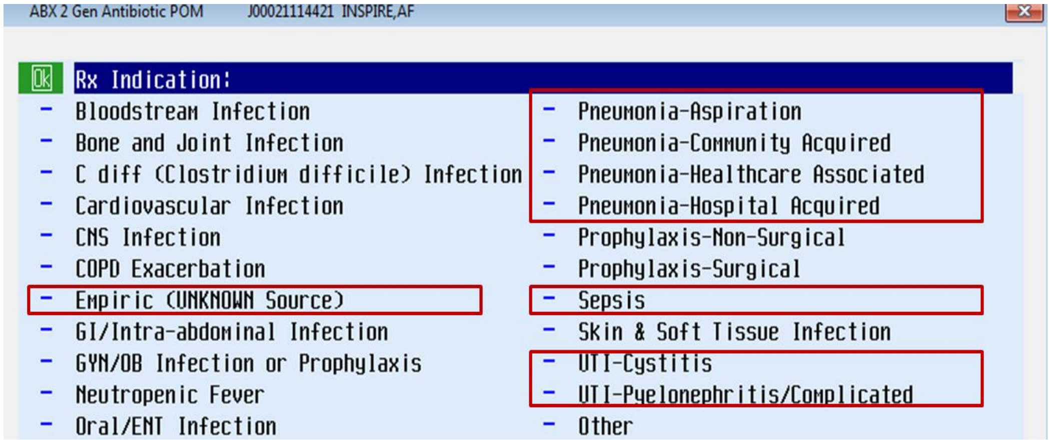

Figure 3. **Screenshot of HCA antibiotic indication screen. Indications highlighted in red will trigger the INSPIRE CPOE smart prompt alert.**

Which antibiotics do NOT trigger the smart prompt?

Narrow spectrum antibiotics do NOT trigger the smart prompt. Examples of standard spectrum antibiotics include:

- For pneumonia: ceftriaxone, ceftriaxone/azithromycin, ampicillin-sulbactam
- For UTI: ceftriaxone, nitrofurantoin

Why was 10% used as a cut-off to identify high versus low risk patients?

National experts advocate the use of standard spectrum antibiotics in patients with less than 10% risk of MDRO in some infections, including pneumonia. Several studies have shown that the risk of MDRO as a cause of pneumonia or UTI is extremely low. For example, our retrospective evaluation of the HCA data shows that among almost 230,000 patients hospitalized with pneumonia, less than 2% grew MRSA by culture. Similar findings have been reported in multiple other studies.

What factors were taken into account when estimating my patient's risk for an MDRO infection?

The INSPIRE study team assessed over 536,000 patients admitted at all HCA hospitals over 3 years (2014-2016) within Meditech. We evaluated (1) your hospital antibiogram for pneumonia and UTI patients and (2) patient risk factors of MDRO infection:

- Demographics (age, gender, race, ethnicity, insurance status)
- Comorbid conditions (e.g., diabetes, lung disease, renal failure)

- History of ED visit or admission to an HCA hospital
- History of nursing home admission
- History of antibiotics received during an ED or inpatient admission
- History of MDRO (screening or clinical cultures at your facility)
- Lab values (e.g., BUN, creatinine)

Using a special statistical method, we identified which risk factors were most often found in patients who developed pneumonia or UTI with an MDRO. Using these risk factors (which differ for every MDRO), the CPOE smart prompt gives the patient's **absolute risk** for infection with the MDRO(s) targeted by the antibiotic ordered.

Note that INSPIRE calculates your facility antibiogram as the proportion of MDRO or pseudomonas among patients admitted with PNA or UTI, which is different than the traditional way most microbiology labs calculate this.

For more detailed information, you can review an audio-enabled presentation on the HCA Atlas website, entitled "How the MDRO Risk Estimates Work": http://connect.medcity.net/web/hca-research-academic-affairs/inspire

Will risk estimates be validated after the intervention starts?

Our risk estimates will be re-validated at regular intervals during the intervention. Significant changes in select variables (e.g., changes in your antibiogram) will be updated within the CPOE alert. Note that our estimates are based on 3 years of data over 140 HCA hospitals, it is unlikely that additional accrual of trial time will change risk factors that have already been identified.

How far back does the CPOE smart prompt look for MDRO history?

The CPOE smart prompt mines your facility's microbiology data in Meditech as far back as 2012. It will capture a patient's MDRO history even if there is no electronic MDRO flag on chart, but it will not capture MDRO history or cultures from an outside hospital.

How quickly can the CPOE smart prompt account for positive cultures? What if a culture was sent recently?

The CPOE smart prompt captures cultures resulted in Meditech at your facility 7 or more days ago; it currently cannot integrate new cultures resulted in the empiric period. For example, if a culture was sent upon admission, this will not be included in a risk estimate when ordering extended-spectrum antibiotics on hospital day 3. We

encourage close monitoring of all new microbiology results as they become available and selection of antibiotics accordingly.

What if my patient is neutropenic, going to the ICU, or has a positive MDRO culture from an outside hospital?

The smart prompt is intended to support, not replace clinical judgment. You should use all available information about a patient as you consider your antibiotic choice, such as: neutropenia, allergy to recommended antibiotics, imminent admission to the ICU, or MDRO positive cultures from this admission or an outside hospital.

What if the smart prompt calculates my patient's MDRO risk to be low but culture eventually grows an MDRO? Does this mean the smart prompt does not work?

As clinicians, we are trained to take our best educated guess on the most likely diagnoses to explain patient symptoms and order testing and treatment accordingly. This smart prompt provides a robust estimate of MDRO risk to support clinical decision-making in the empiric period when cultures are not known. If the prompt finds a patient's risk for MRSA pneumonia is 2.5%, this risk is low, but not zero. It is expected that on rare occasions patients will grow an MDRO when the smart prompt said it was unlikely. With close monitoring, there is time to await cultures before broadening coverage in the non-ICU patient. Clinicians are expected to use clinical judgment, monitor patients daily, and adjust antibiotics as needed based on microbiology results and patient response to treatment.

Are standard spectrum antibiotics safe when MDRO risk is low?

Yes. Studies have shown that in non-ICU patients, there is time to observe patients on standard-spectrum antibiotics, even when an MDRO grows later. A delay in antibiotics in non-ICU patients does not necessarily increase morbidity or mortality.[1-3] The smart prompt is intended to support, not replace clinical judgment. We encourage close monitoring of all patients and if a patient clinically progresses despite starting standard-spectrum antibiotics, clinicians are expected to broaden antibiotic coverage.

References: [1]Rhedin, S., et al. Clinical Microbiology and Infection, 23.4 (2017): 247-252. [2]Kalil, A., et al. Clinical Infectious Disease, CID 2016:63 (1 September). [3]Lin, Michael Y., Antimicrobial agents and chemotherapy 52.9 (2008): 3188-3194

Will I receive feedback on my antibiotic use?

Yes. Your facility's study champions will share provider-specific feedback on empiric antibiotic selections, how often standard-spectrum antibiotics were chosen, how often recommendations were declined, and reasons for not following recommendations. This data is intended to help you evaluate empiric antibiotic selections.

Has the Antibiotic Stewardship Committee and other leadership approved of the smart prompt alert process and recommendations?

Yes. The smart prompt alert and study protocol has been reviewed and approved by your antibiotic stewardship team, hospital committee(s), your hospital leadership, HCA corporate, and subject matter experts at the CDC.

Will patients need to give informed consent?

No. This study has been approved as a minimal risk study and will be implemented as a quality improvement (QI) protocol through the existing infrastructure of your hospital's antibiotic stewardship programs. The assigned protocol will be approved through your usual clinical/leadership committees and become hospital guidance on standard spectrum empiric antibiotic use.

Has this study been approved by the Institutional Review Board (IRB)?

Yes. Harvard Pilgrim's IRB will provide ethical oversight for these trials and serve as the central IRB. Both INSPIRE-ASP PNA and UTI trials have been approved by the Harvard IRB. Your hospital has submitted all ceding and reliance agreements necessary to grant oversight by the central IRB at Harvard. HCA's corporate compliance and regulatory affairs will help provide support for this endeavor.

What is the timeline?

- In-person site visits – Summer-Fall 2018
- IT CPOE smart prompt alert testing – Fall, 2018
- Intervention launch – October 1, 2018
- Phase-in – 6 months
- Intervention – 15 months

Essential Clinician Frequently Asked Questions: What you need to know

What does the INSPIRE Smart Prompt do? (What's smart about it?)

The INSPIRE smart prompt is triggered when MDs order extended-spectrum antibiotics for pneumonia or UTI within the first 3 days of hospitalization in non-ICU patients. The smart prompt uses your patient's specific health data from the HCA electronic medical record and your hospital's antibiogram to estimate the risk that your patient is infected with an antibiotic-resistant organism. It does this in relatively real time during the antibiotic ordering process and gives guidance on whether ordering of empiric extended-spectrum antibiotics is warranted.

The INSPIRE smart prompt also provides the specific multidrug-resistant organism (MDRO) risk targeted by the ordered antibiotic. For example:

- If vancomycin ordered → prompt returns likelihood of MRSA infection
- If cefepime or piperacillin/tazobactam ordered → returns *Pseudomonas* risk
- If linezolid ordered → returns combined MRSA and VRE risk
- If meropenem ordered → returns combined ESBL + resistant gram negative bacteria risk

What does the INSPIRE Smart Prompt NOT do?

The INSPIRE prompt only provides **empiric risk** of an MDRO infection when you do not have culture results to direct therapy. **Smart prompt guidance should be ignored if cultures return positive this admission** (blood/respiratory for pneumonia, and blood/urine for UTI). At that point, therapy should be culture-directed. Specifically, the algorithm only accounts for historical cultures, not cultures taken this admission or within the past 7 days. Additionally, it does not include outside cultures from physician offices or non-HCA facilities.

Which antibiotics do NOT trigger the smart prompt?

Standard antibiotics do NOT trigger the smart prompt. Examples of standard therapy antibiotics include:

- For pneumonia: ceftriaxone, ceftriaxone/azithromycin, levofloxacin, ampicillin-sulbactam
- For UTI: ceftriaxone

What's the harm in empiric anti-MRSA and anti-pseudomonal therapy? Isn't this important for the sepsis guidelines?

Clinicians are pulled in two directions. On one hand, there is urgency to give antibiotics to those who need it. On the other hand, antibiotic stewardship teams urge us to exercise good judgment to prevent antibiotic resistance, unnecessary side effects, and loss of effective agents. INSPIRE smart prompts are directed toward **non-ICU patients** with possible pneumonia and UTI. Evidence supports initial empiric treatment with standard-spectrum antibiotics in non-ICU and non-neutropenic patients while cultures are pending to determine if extended-spectrum antibiotics are appropriate. In addition, **all antibiotics recommended by INSPIRE prompts meet required sepsis measures.**

The prompt says my patient is low risk for an MDRO infection. What does that mean?

If your patient has a 10% or greater risk of an MDRO infection, it is reasonable to use an empiric extended-spectrum antibiotic. However, if your patient's risk is <10%, the prompt will say your patient has a low risk of MDRO infection and standard-spectrum antibiotics should be used while cultures are pending.

My patient just cultured MRSA from sputum, but the prompt told me yesterday that my patient was low risk for MRSA pneumonia. Doesn't that mean the prompt doesn't work and that I should have ordered vancomycin on admission?

Your patient had a low risk of MRSA pneumonia based upon risk factors in your hospital's electronic medical record. Clinicians always need to use their best judgment for estimating risk, so if you had no additional information to the contrary, then this patient's risk was accurately assessed. **Low risk does not mean no risk.** At most, 1 in 10 low risk patients will have an MDRO infection, most have a risk far lower than 10%. Again, evidence suggests that non-ICU, non-neutropenic patients with pneumonia or UTI can be treated with standard-spectrum antibiotics until cultures return.

My patient has a positive blood culture for *Pseudomonas*, but the prompt is telling me that my patient is low risk for *Pseudomonas* UTI. What is wrong with the prompt?

The INSPIRE prompt only provides **empiric risk** of an MDRO infection when you do not have culture results to direct therapy. **Smart prompt guidance should be ignored when cultures return** (blood/respiratory for pneumonia, and blood/urine for UTI). At that point, therapy should be culture-directed. Specifically, the algorithm only accounts for historical cultures, not cultures taken this admission.

How timely is the history of MDRO data that feed into the smart prompt? Does it include positive cultures from today?

Currently, the INSPIRE smart prompt reliably accounts for your hospital's microbiologic culture results up to one week prior to admission. We are actively working to improve the time for capturing more recent culture results, and will keep participating sites updated on our progress.

Why is a history of an MDRO not always enough to warrant extended-spectrum antibiotic use to cover that MDRO?

It depends on the MDRO and the indication (pneumonia or UTI). Sometimes, an MDRO history is sufficient, but other times an MDRO history alone is insufficient to meet the 10% risk threshold without additional risk factors. Remember, colonization with a pathogen in the past does not always mean that it is responsible for a current infection. The value of the smart prompt algorithm is that it used over 500,000 patients over 3 years to determine what generates a risk >10%. An ATLAS webinar is available for those who wish to understand greater details about the algorithm. (http://connect.medcity.net/web/hca-research-academic-affairs/inspire)

Are allergies auto-programmed into the smart prompt?

No. Allergies are often not recorded correctly in the EHR. Those listed may not be real, and real ones may be unlisted. For example, among patients labeled as beta-lactam allergic without anaphylaxis, over 90% are able to tolerate a beta-lactam after thorough assessment of allergy history. We ask all MDs to critically evaluate allergies when ordering antibiotics. In addition, your standard hospital alerts for allergies still apply.

Are the clinician progress reports to be used by HCA as a quality indicator for the physician dashboard?

No. Progress reports are provided for local pharmacy, ID, ED, and hospitalist champions to support judicious antibiotic prescribing. The hope is that it will build collaboration across the intervention arm and potentially provide a best practice strategy for the field.

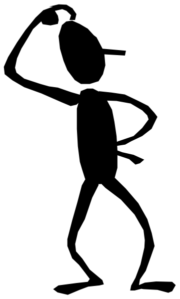

Smart Prompt Clinician Prescriber
Do’s & Don’ts

DO

- Follow the smart prompt recommendations for patients with PNA or UTI admitted to non-ICU wards
- Follow your hospital’s sepsis protocol
- Use your clinical judgment to decide which antibiotic is best for your patient
- Review your patient’s history carefully to identify risk factors for severity of illness or neutropenia
- Review your smart prompt acceptance and respond to your antibiotic stewardship team’s feedback and recommendations

DON’T

- Do not use extended-spectrum antibiotics when they are not indicated
- Do not use medications that are contraindicated for your patient (e.g., allergy to the recommended antibiotic)
- Do not replace your clinical judgment with smart prompt recommendations (e.g., patient is transferred from another hospital for known MRSA pneumonia but prompt recommends ceftriaxone because this history is not in electronic medical record)

Please contact your local Study Champion(s) for INSPIRE-ASP details

Study Investigators & Coordinators
(844) 522-4677
HCAINSPIRE@gmail.com

INSPIRE-ASP Trial

INtelligent Stewardship Prompts to Improve Real-time Empiric Antibiotic Selection for Patients

Agenda

- Welcome
- Context & Rationale
- INSPIRE-ASP Overview
- CPOE Smart Prompt Intervention
- MDRO Risk Estimate Calculations
- Quarterly Clinician Progress Reports
- What to Expect

Welcome to Arm 2

Smart Prompt Alert Intervention

Precision medicine smart prompt alerts for patient-specific empiric antibiotic selection

These trials are a partnership between HCA Healthcare and academic investigators who are federally funded by the Centers for Disease Control and Prevention (CDC)

INSPIRE
BETTER SAFER ANTIBIOTIC USE

Context & Rationale

Overuse of Antibiotics: A Major National Priority

- Broad-spectrum antibiotics increase resistance[1]
- 40% of inpatients receive inappropriate antibiotics[2]
  - Majority due to pneumonia or UTI
- Impact on patient safety (side effects, *C. difficile)*
- National and state mandates for stewardship programs

[1]Paterson, D et al. Clin Infect Dis 2014;33:126-128

[2]Fridkin, S et al. MMWR Morb Mortal Wkly Rep 2014;63(9):194-200

Challenges Driving Broad-Spectrum Antibiotic Use

- **Diagnostics uncertainty**
- **Treatment challenges**
  - Optimal treatment unclear
  - Guidelines broad
- **Medical-legal implications**
- **Physician factors**
  - Knowledge gaps
  - Risk aversion

+

Complicated Hospital Antibiogram

Antibiotic	*E. coli* % Susceptibility	*K. Pneumoniae* % Susceptibility
Ampicillin	51	0
Ampicillin-sulbactam	48	79
Piperacillin-tazobactam	95	95
Cefazolin	58	68
Ceftriaxone	91	92
Cefepime	96	97
Ertapenem	100	100
Meropenem, imipenem	100	100
Gentamicin, tobramycin	90	94
Trimethoprim-sulfa	71	85
Cipro/levofloxacin	77	96
Tigecycline	100	100
Nitrofurantoin	97	31

Causes Broad-Spectrum Antibiotic Overuse

Empiric Antibiotic Choice: There is Time to Broaden

- Growing evidence in non-ICU patients – little/no harm in delay
- Similar outcomes seen in non-ICU patients when treated with empiric standard vs broad-spectrum antibiotics[1]
- Waiting for culture results to start anti-MDRO antibiotics does not adversely impact mortality[1,2]

[1]Rhedin, S., et al. Clinical Microbiology and Infection, 23.4 (2017): 247-252.
[2]Lin, Michael Y., Antimicrobial agents and chemotherapy 52.9 (2008): 3188-3194.

Empiric Antibiotic Choice: There is Time to Broaden

- In **non-ICU** patients admitted for pneumonia:
  - Narrow-spectrum antibiotics safe, no increased mortality[1]
  - Pathogens similar in CAP and HCAP[1]
- HCAP designation removed from IDSA HAP/VAP Guidelines[2]
- Non-ICU patients with ANC >100, waiting for culture results not associated with higher mortality[3]

[1]Rhedin, S., et al. Clinical Microbiology and Infection, 23.4 (2017): 247-252.
[2]Kalil, A., et al. Clinical Infectious Disease, CID 2016:63 (1 September).
[3]Lin, Michael Y., Antimicrobial agents and chemotherapy 52.9 (2008): 3188-3194.

INSPIRE
BETTER SAFER ANTIBIOTIC USE

How Can We Influence Prescribing?

- U.S. far higher use of extended-spectrum antibiotics vs. other 1st world nations[1]
- UK guidelines for pneumonia:
  - Low severity: amoxicillin
  - Moderate severity: amoxicillin + macrolide
- Australian guidelines for severe sepsis due to pneumonia includes penicillin as part of the base combination therapy[2]

[1]https://www.nice.org.uk/guidance/cg191/chapter/1-Recommendations

[2]http://www.cec.health.nsw.gov.au/data/assets/pdf_file/0005/299417/adult-antibiotic-guideline-severe-sepsis-septic-shock-sept2016.pdf

Rationale for INSPIRE-ASP Trial

- Uncertainty about MDRO risk drives overuse of empiric extended-spectrum antibiotics
- Physicians can choose wisely if
  - Actionable information provided in real time
  - Recommendation based on individual patient
  - Feedback provided

Two Trials in One:

INSPIRE-ASP Trials

INtelligent Stewardship Prompts to Improve

Real-time Empiric Antibiotic Selection for Patients

INSPIRE-ASP Study Design

2-in-1 Set of Cluster-Randomized Trials – 59 Hospitals

- Trial 1: Pneumonia (PNA)
- Trial 2: UTI

- One intervention
  - CPOE precision medicine smart prompt alert
  - Real time recommendation for antibiotic choice
  - Feedback on acceptance
- Two separate analyses and manuscripts
- IRB oversight: Harvard Pilgrim
- No informed consent needed

INSPIRE-ASP Trial Arms

Arm 1: Routine Care – 30 Hospitals

- Standard-of-care antibiotic stewardship

Arm 2: Smart Prompt Alert Intervention – 29 Hospitals

- CPOE precision medicine alerts for patient-specific MDRO risk
- Recommends standard-spectrum antibiotic choice
- Provider feedback reports

INSPIRE-ASP Study Population & Primary Outcome – PNA

Includes

- Adults in ED and non-ICU units
- Admitted for **pneumonia (PNA)**

Excludes

- ICU and pediatric patients

Primary Outcome

- Empiric extended-spectrum (ES) antibacterial treatment for **PNA** per at-risk day
  - "Empiric" = first 3 hospital days

INSPIRE-ASP Study Population & Primary Outcome – UTI

Includes

- Adults in ED and non-ICU units
- Admitted for **urinary tract infection (UTI)**

Excludes

- ICU and pediatric patients

Primary Outcome

- Empiric extended-spectrum (ES) antibacterial treatment for **UTI** per at-risk day
  - "Empiric"= first 3 hospital days

Other Outcomes

Secondary Outcomes

- Subgroups of vancomycin, antipseudomonals

Safety Outcomes

- Antibacterial escalation
- Length of stay
- ICU transfers

Other Outcomes (secondary manuscripts)

- *C. difficile* infection
- MDRO prevalence
- Cost-analysis

Note: Two trials in one

Each outcome evaluated separately and separate manuscripts planned for PNA and UTI cohorts

Baseline Pneumonia & UTI Due to MDRO – 2014-2017

- Among >500,000 HCA Healthcare (HCA) patients, <2% of PNA patients and <6% of UTI patients grew drug resistant organisms

Vast majority could have been treated with standard-spectrum
Safe to use standard-spectrum while waiting for culture results

	MDRO Positive Pneumonia (%)	MDRO Positive UTI (%)
**MRSA**	**1.7**	**1.4**
Pseudomonas	**1.5**	**3.4**
**ESBL**	**0.4**	**5.8**
**CRE**	**0.03**	**0.3**
**VRE**	**--**	**0.8**

Extended-Spectrum Overuse in Non-MDRO HCA Patients

- Over 20-fold higher extended-spectrum (ES) use despite very small numbers of MDRO pneumonia or UTI

Model	MDRO Culture Positive		ES Antibiotic Use in Non-MDRO Positive*	
	N	%	N	%
**MRSA PNA**	**3,231**	**1.7**	**67,856**	**34.5**
***Pseudomonas* PNA**	**2,995**	**1.5**	**79,830**	**39.9**
***Pseudomonas* UTI**	**10,492**	**3.4**	**74,657**	**24.5**

HCA baseline data 2014-2017
Pneumonia (PNA), n = 199,903
Urinary tract infection (UTI), n = 304,584
*Note: "ES" = anti-MRSA antibiotic use for MRSA PNA/UTI and antipseudomonal use for *Pseudomonas* PNA/UTI

CPOE Smart Prompt Intervention

When Will CPOE Smart Prompt Fire?

- **Smart prompt only fires if extended-spectrum antibiotics** ordered for **PNA or UTI** in the **ED or non-ICU wards** within **first 3 days** of admission
  (1) Gives patient's risk of PNA or UTI due to MDRO
  (2) Recommends standard-spectrum antibiotics when risk is low (<10%)

- **NO prompt will fire** when ordering standard-spectrum antibiotics (e.g., ceftriaxone)

BETTER SAFER ANTIBIOTIC USE

What Antibiotics Are Considered "Extended-Spectrum"?

Category	Extended-Spectrum Antibiotics Included in CPOE Alerts
**Anti-MRSA**	Ceftaroline, Daptomycin, Linezolid[1], Vancomycin[2]
**Anti-VRE**	Daptomycin, Linezolid[1]
**Anti-Pseudomonas**	Aztreonam, Cefepime, Ceftazidime, Piperacillin/Tazobactam
**Anti-ESBL**	Ertapenem, Doripenem, Imipenem, Meropenem, Ceftolozane/Tazobactam,
**Anti-CRE**	Ceftazidime/Avibactam, Colistin, Polymixin B, Tigecycline

[1]Both oral and intravenous (IV) formulations

[2]IV formulation only

INSPIRE BETTER SAFER ANTIBIOTIC USE

CPOE Smart Prompt

- **How is it Smart?**
  - ✓ Uses ***your specific patient*** EHR data to calculate MDRO risk based on models from 500,000 HCA patients with PNA or UTI
  - ✓ Risk estimate tailored to antibiotic selected. For example:
    - If ceftriaxone ordered → no prompt (recommended)
    - If vancomycin ordered → returns MRSA risk
    - If cefepime or Zosyn ordered → returns *Pseudomonas* risk
    - If linezolid ordered → returns MRSA and VRE risk
    - If meropenem ordered → returns ESBL + other resistant gram negative bacteria risk
  - ✓ Recommends ceftriaxone when MDRO risk is low (<10%)
  - ✓ If risk ≥10%, extended-spectrum order proceeds without any prompts

CPOE Smart Prompt Activated Via Indication Screens

- INSPIRE prompts triggered based upon selected indication:
  - **PNA**
  - **UTI**
  - **Sepsis/Empiric** – triggers additional screen asking if possible respiratory or urinary source

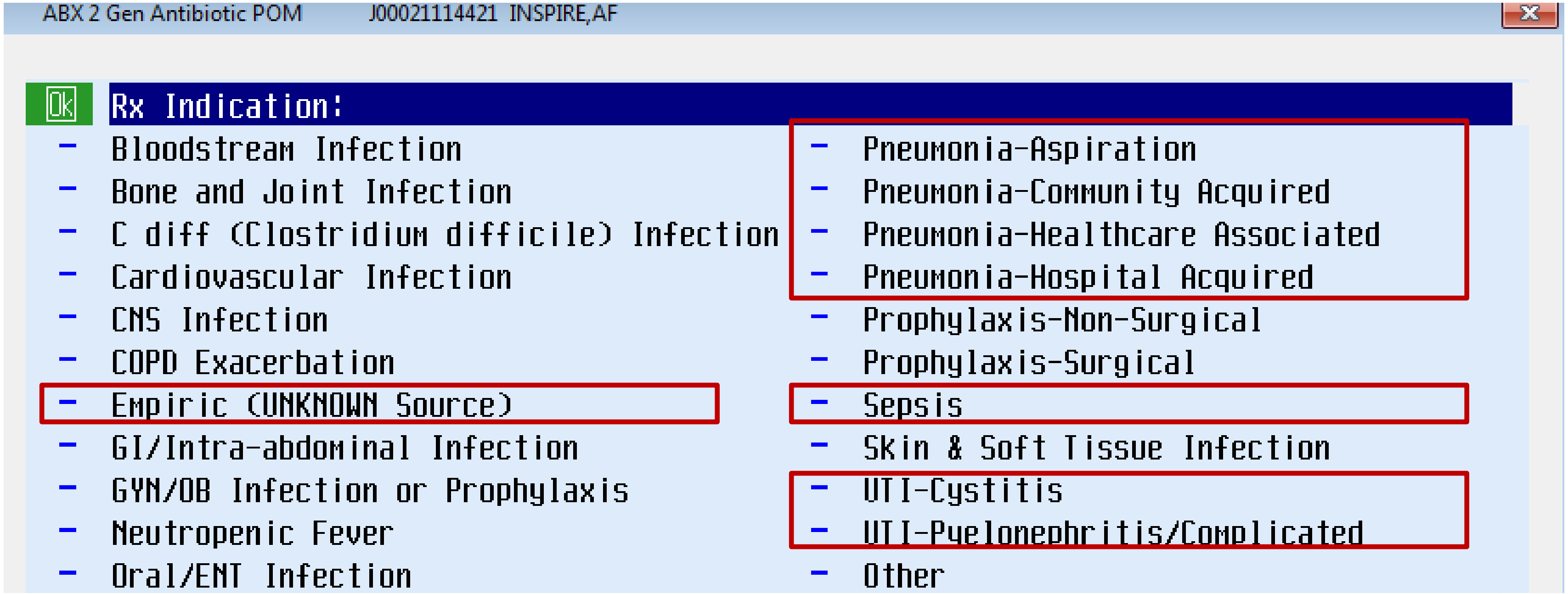

ED Patients Going to ICU

- Smart prompt **will not** fire if patient location = ICU
- **For ED patients**, asks if patient is likely to go to the ICU
  - If YES → no further screens
  - If NO → INSPIRE CPOE screens continue

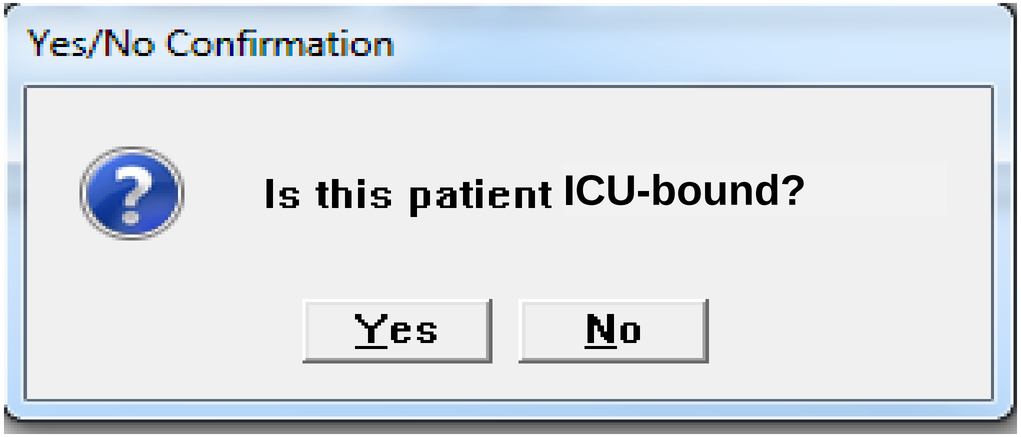

INSPIRE-ASP CPOE Smart Prompt Screen - Example

- Physician orders cefepime for pneumonia and risk of *Pseudomonas* pneumonia calculated to be low (<10%)

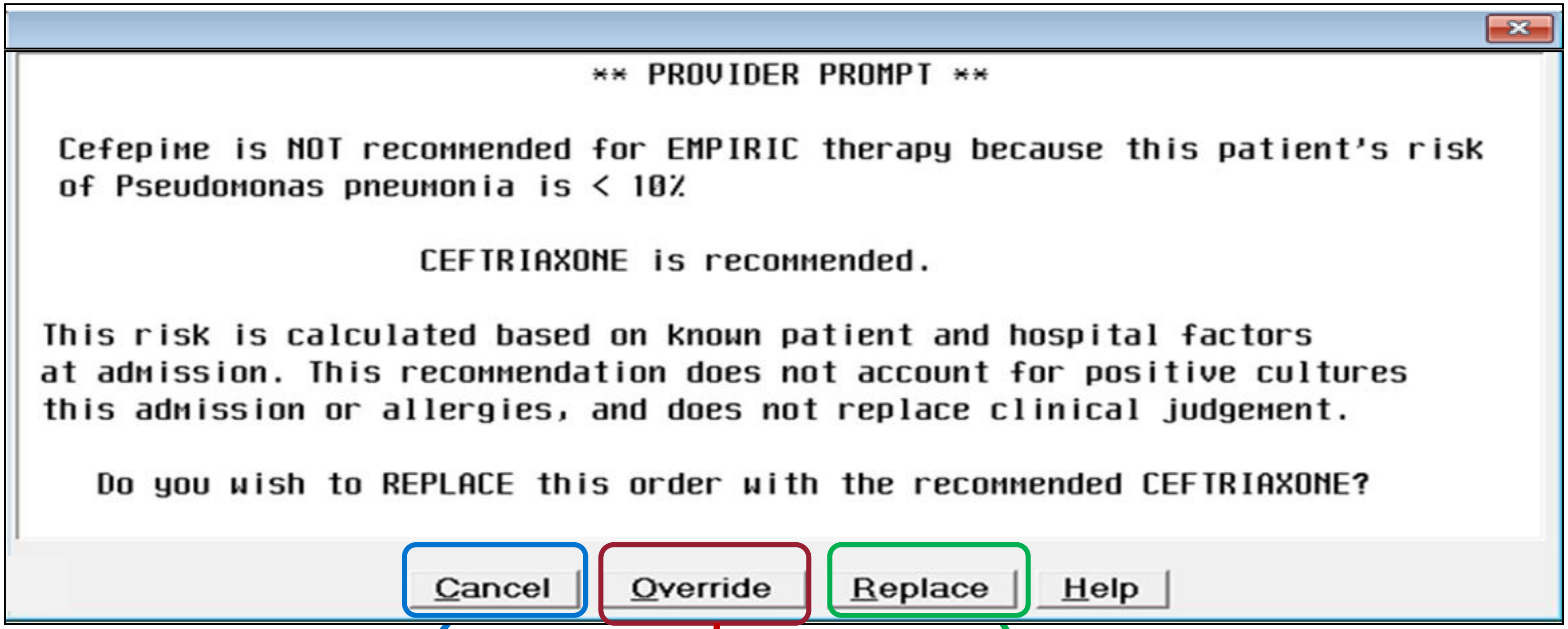

Returns to order screen

Next screen asks reason to keep cefepime

Taken to ceftriaxone order screen

Replacing Extended-Spectrum Antibiotic

- If choose **REPLACE** → CPOE takes physician directly to ceftriaxone ordering screen

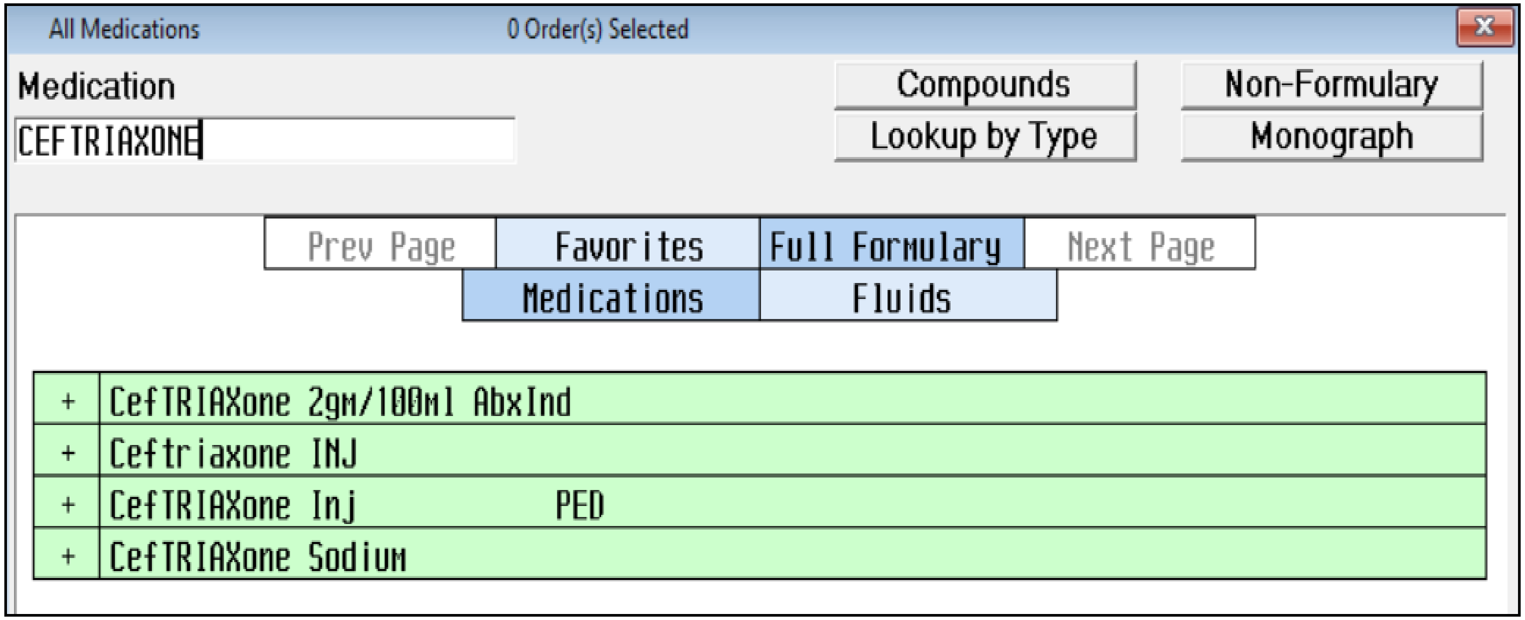

Override Screen and Options

- Clinicians choosing to **override** are asked to indicate the reason:

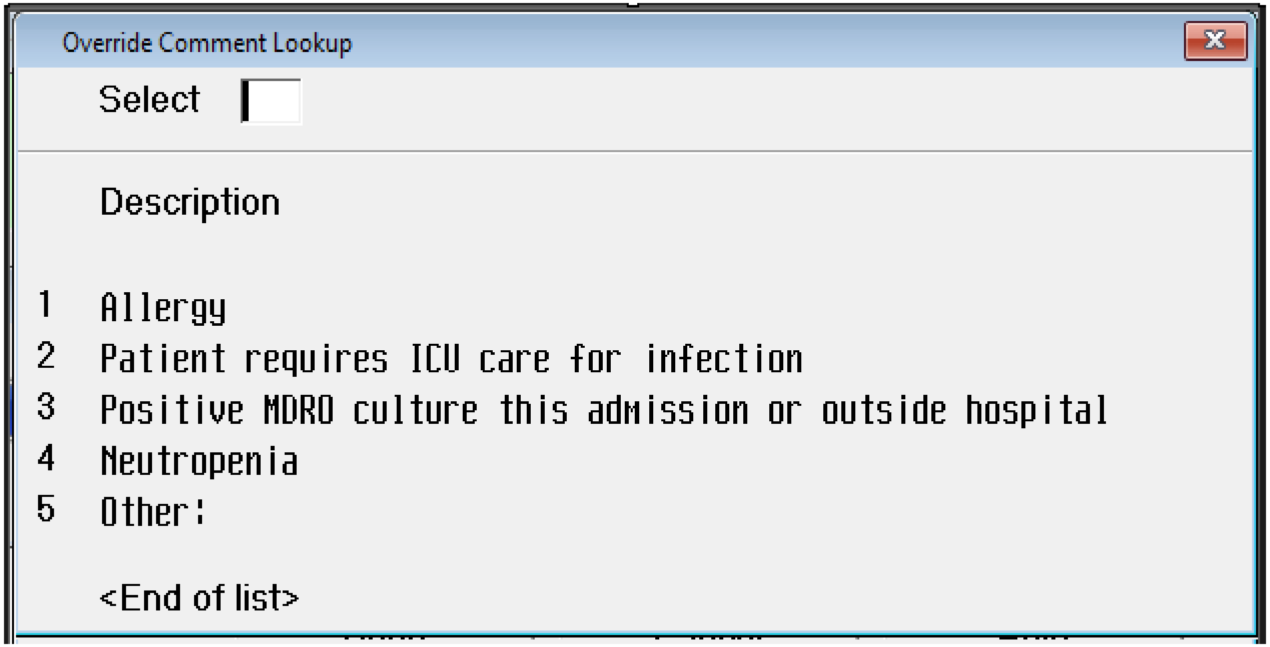

Allergy **<u>not</u> auto-programmed: some allergies missing in EHR, and many in EHR are not true allergies – needs clinical judgment*

** Usual facility allergy prompts continue to appear*

What Happens if Patient Risk is Higher than 10%?

- If patient's MDRO infection risk higher than 10%, the CPOE alert will automatically allow physician to continue order for extended-spectrum antibiotic

- No CPOE screen will be generated

Why 10%?

- No added risk to wait for culture in non-ICU population
- National experts, including at CDC, agree on INSPIRE threshold
- Extended-spectrum antibiotics incur risks
  - Greater impact to microbiome
  - Increase MDRO selection and acquisition
  - Increase risk of *C. difficile*
  - Increase development of resistance
  - Increased side effects (added antibiotics)
  - Unnecessary expense

Patient MDRO Risk Calculation

The Problem with Relative Risk and Odds Ratios (OR)

- Clinicians are taught about relative risk and odds ratios
- We worry that the OR for an MDRO infection is 2 or 3 for certain factors like nursing home residence or diabetes
- However, this tells us nothing about the absolute risk
- An example
  - Let’s say females have a risk of 1 in 1,000 for MRSA pneumonia
  - If males have a risk of 3 in 1,000, that would be OR = 3 for an absolute risk of 0.3%
  - Better to know absolute risk
  - **The INSPIRE trials use absolute risk**

INSPIRE
BETTER SAFER ANTIBIOTIC USE

If Not Odds Ratios, Then What?

- Use recursive partitioning for estimating patient risk
- Fancy term for a decision tree
- Parses variables to see which ones matter and in what order
- Generates a final set of important characteristics of high vs low risk using statistics

For INSPIRE-ASP, high risk defined as >= 10%

Recursive Partitioning Example

Decision tree to identify patients at high vs low risk for cardiac disease

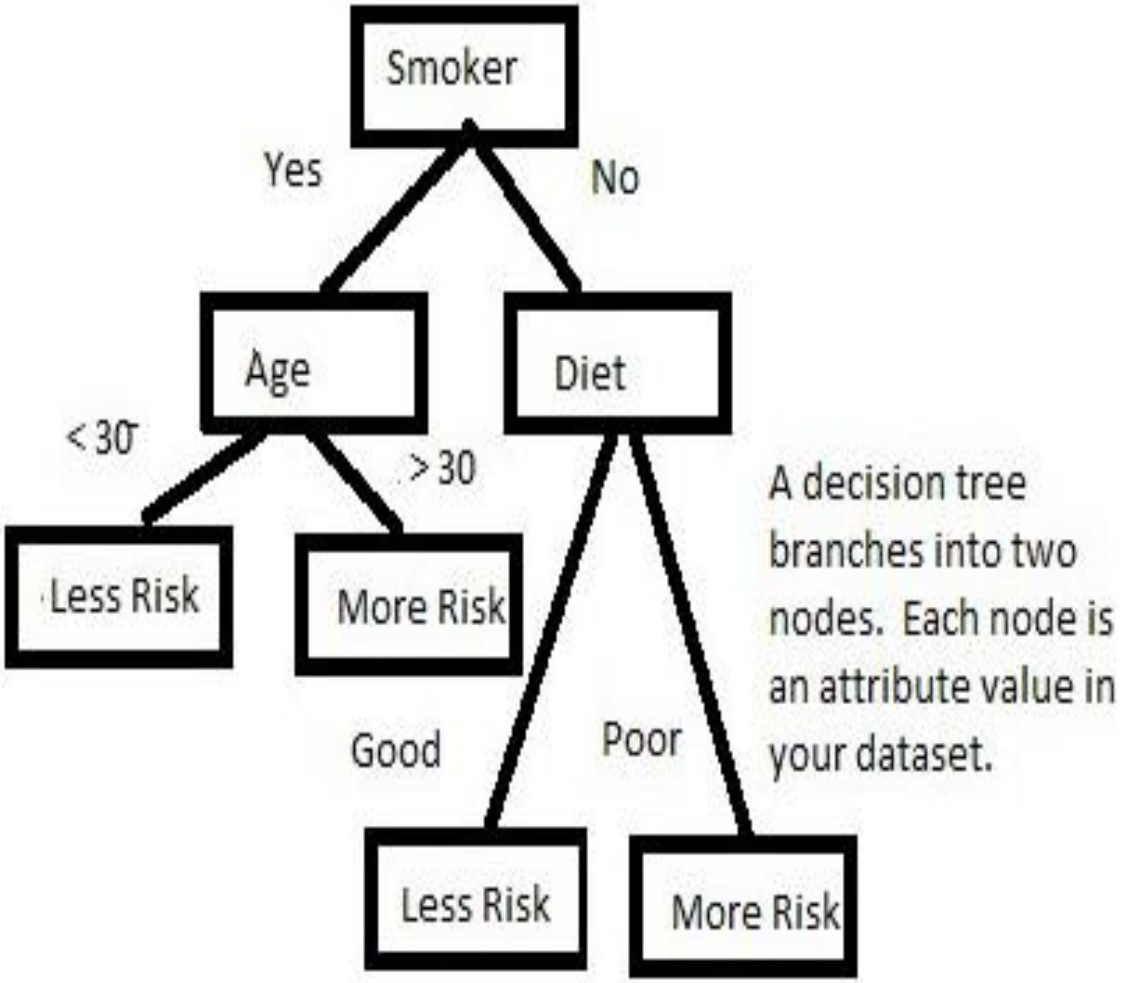

What Variables Were Assessed?

- 536,000 patients admitted to HCA Healthcare hospitals, 2014-2016
- We evaluated:

  (1) Your hospital antibiogram for pneumonia and UTI patients

  (2) Risk factors of MDRO infection
    - Demographics
    - Comorbidities
    - MDRO or *Pseudomonas* history (screening or clinical culture)
    - Recent admission, ED visit, nursing home care
    - Recent antibiotic use
    - Labs (e.g., BUN, creatinine)

Factors predictive of MDRO infection were programmed into Meditech to provide a patient-specific risk estimate for each MDRO.

Pseudomonas Pneumonia Risk Estimate Model

- Among all risk factors only history of *Pseudomonas* and percent facility *Pseudomonas* pneumonia were found to be important in distinguishing high from low risk patients

Rules	N	Proportion Predicted with PSE PNA	Admits Receiving Anti-PSE Antibiotics
PSE Hx, % Facility PSE PNA > 2.0%	1588	**18%**	**64%**
PSE Hx, % Facility PSE PNA <= 2.0%	2068	**11%**	**62%**
All orders not meeting above	156375	<=5%	40%

HCA baseline data 2014-2017
Pneumonia (PNA), n = 199,903
"PSE"= *Pseudomonas*, "Hx"= history, "MDR"= multidrug resistant
"ESBL"= extended-spectrum beta lactamase
NOTE: % Facility PSE PNA = number of patients with positive respiratory or blood cultures positive for *Pseudomonas* among all patients admitted with pneumonia.

Pseudomonas UTI Risk Estimate Model

Rules	N	Proportion Predicted with PSE UTI	Admits Receiving Anti-PSE Antibiotics
PSE hx, Male, MDR-PSE hx	895	**28%**	**56%**
PSE hx, Male, No MDR-PSE hx, No ESBL hx	2276	**24%**	**55%**
PSE hx, Female, MDR-PSE hx	833	**21%**	**50%**
PSE hx, Male, ESBL hx, No MDR-PSE hx	784	**18%**	**55%**
PSE hx, Female, No MDR-PSE hx	3152	**14%**	**41%**
No PSE hx, Male, MRSA hx	2684	9%	45%
No PSE hx, Male, ESBL hx, No MRSA hx	2690	7%	40%
All orders not meeting above	230465	<=5%	25%

HCA baseline data 2014-2017
Urinary tract infection (UTI), n = 304,584
"PSE"= *Pseudomonas*, "Hx" = history, "MDR"= multidrug resistant
"ESBL"= extended-spectrum beta lactamase.
NOTE: % Facility PSE UTI = number of patients with positive urinary or blood cultures positive for *Pseudomonas* among all patients admitted with UTI.

Precedence for 10% Threshold

- Prevalence of many MDRO positive cultures well below 10%
- Pneumonia
  - IDSA CAP 2007: “high level” *S. pneumoniae* resistance to azithromycin defined as > 25%
- UTI
  - IDSA UTI 2010: “high level” *E. coli* resistance defined as:
    - > 20% for trimethoprim/sulfamethoxazole (Bactrim)
    - > 10% for fluoroquinolones

Validation and Updating Smart Prompt Risk Estimates

- Risk estimates will be revalidated periodically to ensure accuracy
- Significant changes in select variables (e.g., changes in your antibiogram) will be updated within the CPOE alert
- Note: given assessment of 3 years of data over 140 HCA Healthcare hospitals, it is unlikely that additional accrual of trial time will change prior identified factors
- INSPIRE is a robust assessment

Clinical Judgment Matters

- CPOE alert provides objective, real-time, patient-specific risk estimates to help you choose the best initial antibiotic
- Alert supports, but does not replace clinical judgment
- You may have information about a patient that is not in the computer that should be considered in your decision:
  - MDRO culture this admission or outside hospital
  - Neutropenic
  - Allergic to recommended antibiotic
  - Imminently headed to the ICU
- Clinicians are expected to use clinical judgment, monitor patients daily, and adjust antibiotics as needed based on microbiology results and patient response to treatment

Quarterly Clinician Progress Reports

WHO Principles of Clinical Decision Support

- Speed
- Simplicity
- Usability
- Integration into workflow
- Actionable and provides alternatives
- Evidence/justification provided
- **Impact should be monitored**

Quarterly Clinician Progress Reports - Example

Clinician	Total PNA Non-ICU Admissions N	Empiric Antibiotics Ordered Per Protocol N (%)	Smart Prompts Triggered N (%)	Smart Prompt Declined/ Overridden N (%)	Acceptance of Smart Prompt Recommendation N (%)
**You**	12	8 (67)	4 (33)	3 (75)	9 (75)
**MD 1**	10	5 (50)	5 (50)	2 (40)	8 (80)
**MD 2**	9	3 (33)	6 (67)	4 (67)	5 (56)
**MD 3**	7	4 (57)	3 (43)	1 (33)	6 (86)
**MD 4**	3	2 (67)	1 (33)	1 (100)	2 (67)
**All Clinicians This Hospital**	41	22 (54)	19 (46)	11 (58)	30 (73)
**All Clinicians Arm 2 Trial**	1189	638 (54)	551 (46)	319 (58)	870 (73)

Tracking Declinations and Overrides

Clinician	Reason for Declining or Overriding Smart Prompt				
	Allergy N (%)	Neutropenia N (%)	MDRO Culture N (%)	ICU Bound N (%)	Other N (%)
**You**	1 (33)	0 (0)	1 (33)	1 (33)	0 (0)
**MD 1**	0 (0)	1 (50)	0 (0)	1 (50)	0 (0)
**MD 2**	1 (25)	1 (25)	0 (0)	1 (25)	1 (25)
**MD 3**	0 ()	0 (0)	1 (100)	0 (0)	0 (0)
**MD 4**	1 (100)	0 (0)	0 (0)	0 (0)	0 (0)
**All Clinicians This Hospital**	3 (27)	2 (18)	2 (18)	3 (27)	1 (9)
**All Clinicians Arm 2 Trial**	87 (27)	59 (18)	58 (18)	86 (27)	29 (9)

Quarterly Hospital Progress Reports

- Acceptance of CPOE smart prompt recommendations will be provided quarterly

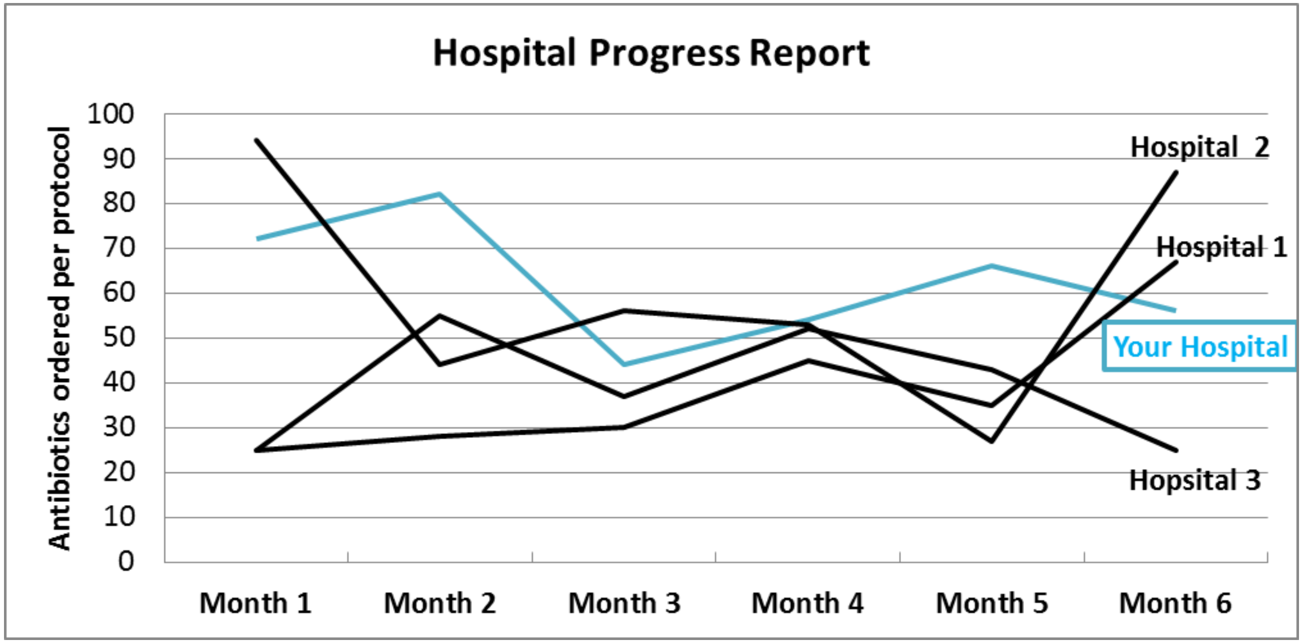

Quarterly Clinician Progress Reports

- Acceptance of CPOE smart prompt recommendations will be provided to stewardship champions and physician quarterly
- Clinician name will only appear on your own report and not shared with other clinicians or hospitals

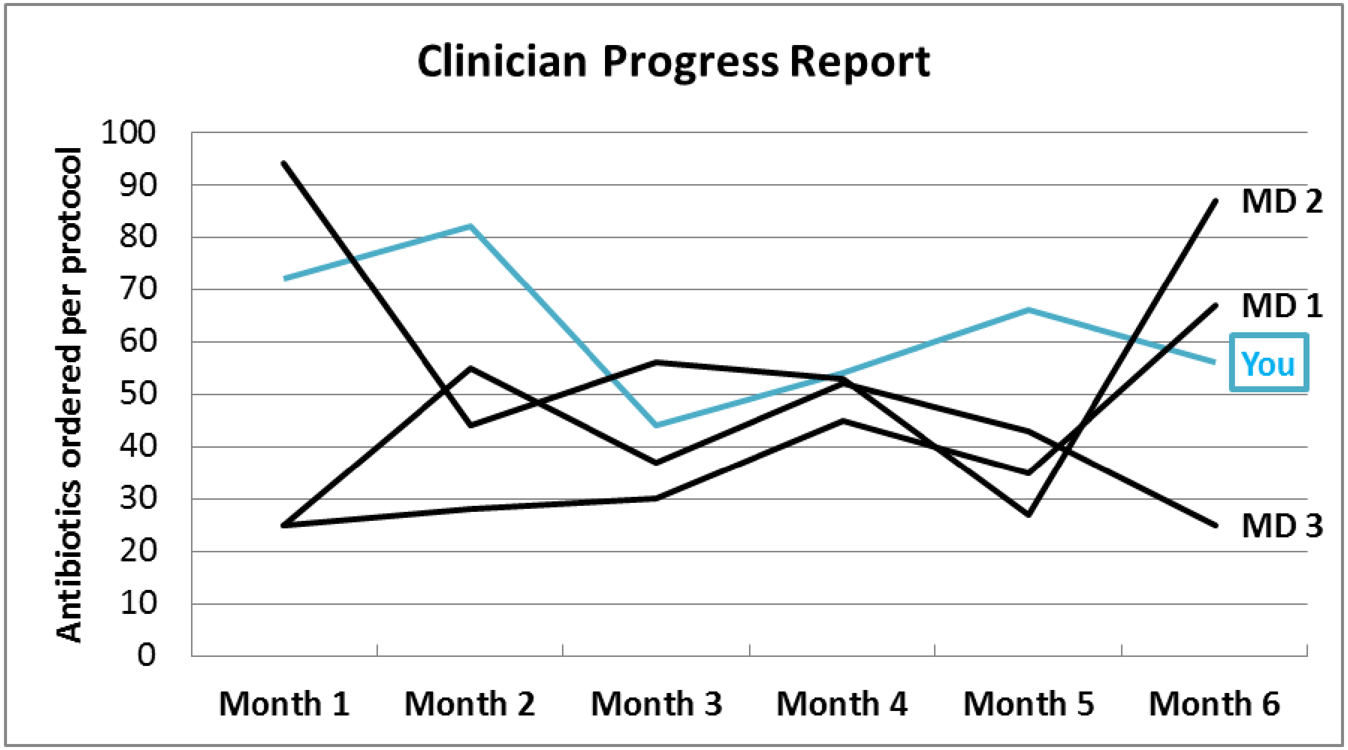

Declinations & Overrides

- Reasons for declining or overriding CPOE recommendations will be monitored

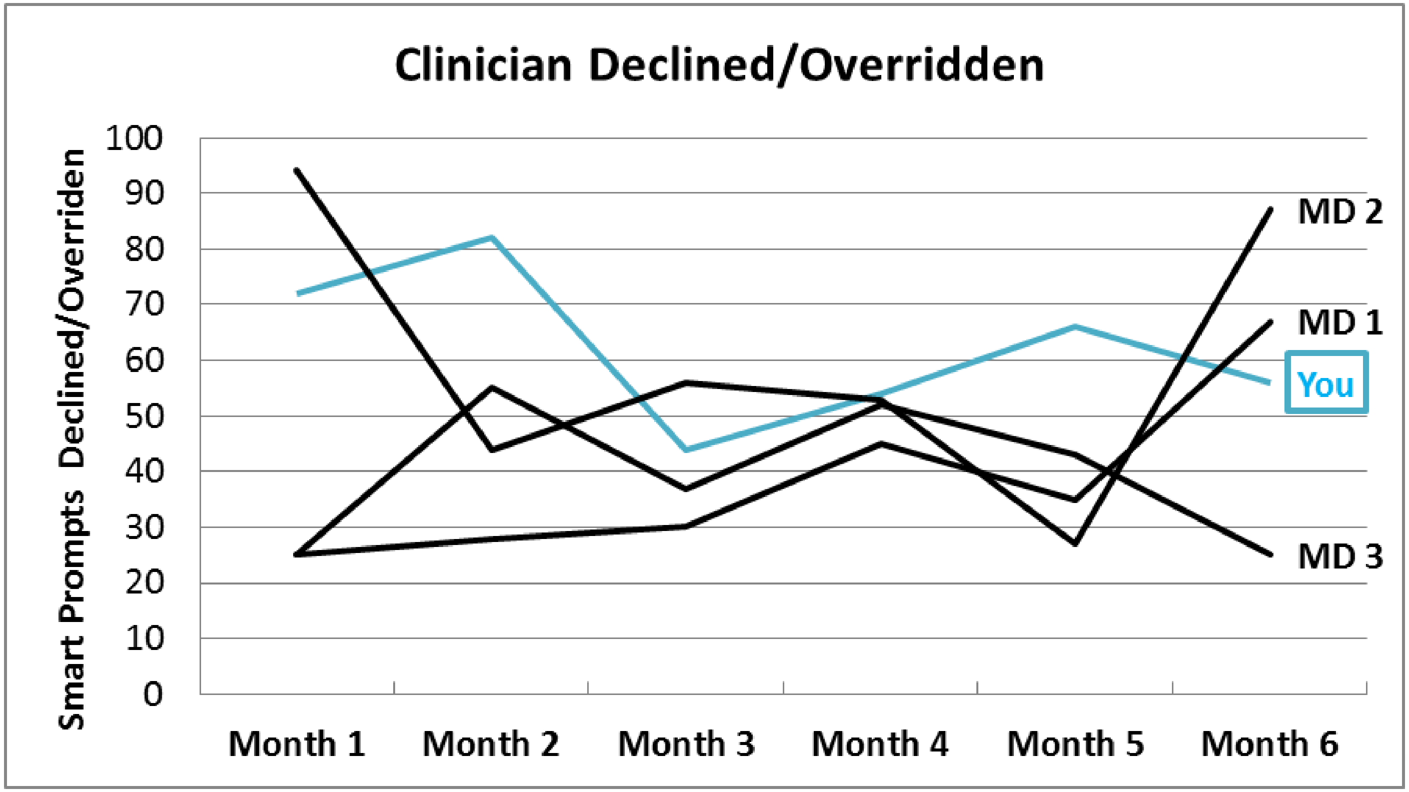

What to Expect

MD and PharmD Champion Roles and Responsibilities

- Educate on value and safety of INSPIRE trial
  - Grand rounds, medical staff meetings (ED, Hospitalists)
- Address clinician outliers in a supportive, knowledgeable way
- Help clinicians understand value of absolute risk
- Review clinician progress reports and encourage improvement
- Set reachable local targets and improvement goals
- Avoid competing interventions

Trial Timeline

- Site visits: **Summer-Fall 2018**
- Intervention launch: **October 1, 2018**
- Phase-in: **6 months**
- Trial duration: **15 months**

Landmark Trial

- First precision medicine tool in antibiotic stewardship
- Real time smart prompt alerts
- Based upon absolute risk, not relative risk
- If successful → best practice electronic support intervention

INSPIRE
BETTER SAFER ANTIBIOTIC USE

INSPIRE-ASP Pneumonia and UTI Trials

__IN__telligent __S__tewardship __P__rompts to __I__mprove __R__eal-time __E__mpiric __A__ntibiotic __S__election for __P__atients

ATTENTION: CLINICIANS

<Hospital> is a proud participant in the INSPIRE-ASP Trials to test the **first precision medicine tool in antibiotic stewardship**.

These trials will encourage the **use of standard-spectrum antibiotics (e.g., ceftriaxone) for non-ICU pneumonia and UTI**.

Starting **October 1st**, physicians ordering extended spectrum antibiotics for pneumonia or UTI in the **ED or non-ICU wards within 3 days of admit** will activate a computerized physician order entry (CPOE) smart prompt that will provide:

- **Your specific patient's risk** for pneumonia or UTI due to antibiotic resistant bacteria using EHR data and your hospital's antibiogram
- Recommendations for standard-spectrum antibiotics if risk is estimated to be low (<10%)

If you have questions, please contact:
<Study Champion Name>
<Study Champion Email>

These landmark trials are a partnership between HCA Healthcare and academic investigators federally funded by the CDC.

INSPIRE-ASP
Computerized Physician Order Entry (CPOE) Alert: Work Flow

Beginning **October 1, 2018** our hospital will launch an antibiotic stewardship intervention to **encourage compliance with national guidance for standard-spectrum antibiotics (e.g., ceftriaxone) for non-ICU pneumonia and UTI.**

Ordering extended-spectrum antibiotics (e.g., vancomycin, cefepime) for pneumonia or UTI in the emergency department (ED) or non-ICU wards within 3 days of admit will activate a computerized physician order entry (CPOE) smart prompt alert that will provide:

- Your patient's risk for pneumonia or UTI due to antibiotic resistant bacteria based upon your patient's EHR data and your hospital's antibiogram
- Recommendation for standard-spectrum antibiotics if risk is estimated to be low (<10%)

This document provides details on the INSPIRE CPOE workflow with relevant screen shots.

1. INSPIRE-ASP CPOE prompts trigger off of the HCA Healthcare Antibiotic Indication Screens. They **will only fire if one of the following indications are chosen in the first 3 days of admission** (outlined in red):

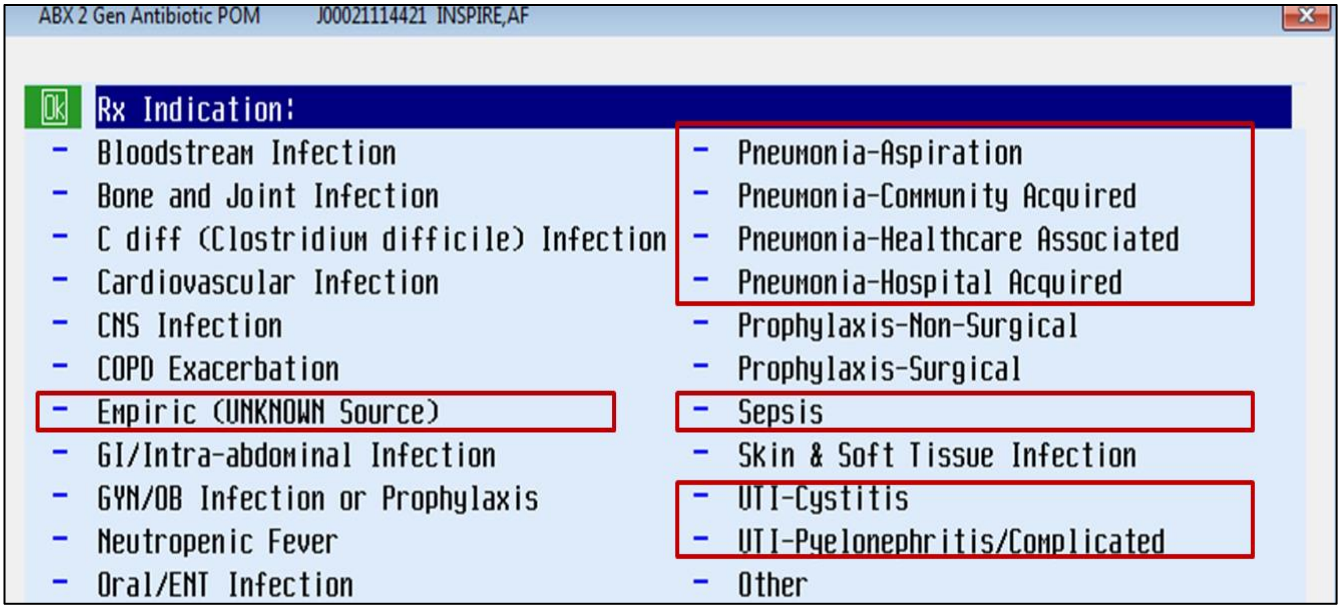

2. INSPIRE prompts **will fire for patients in the ED and non-ICU units only** and are programmed to **NOT** fire if a patient is listed in an ICU location :

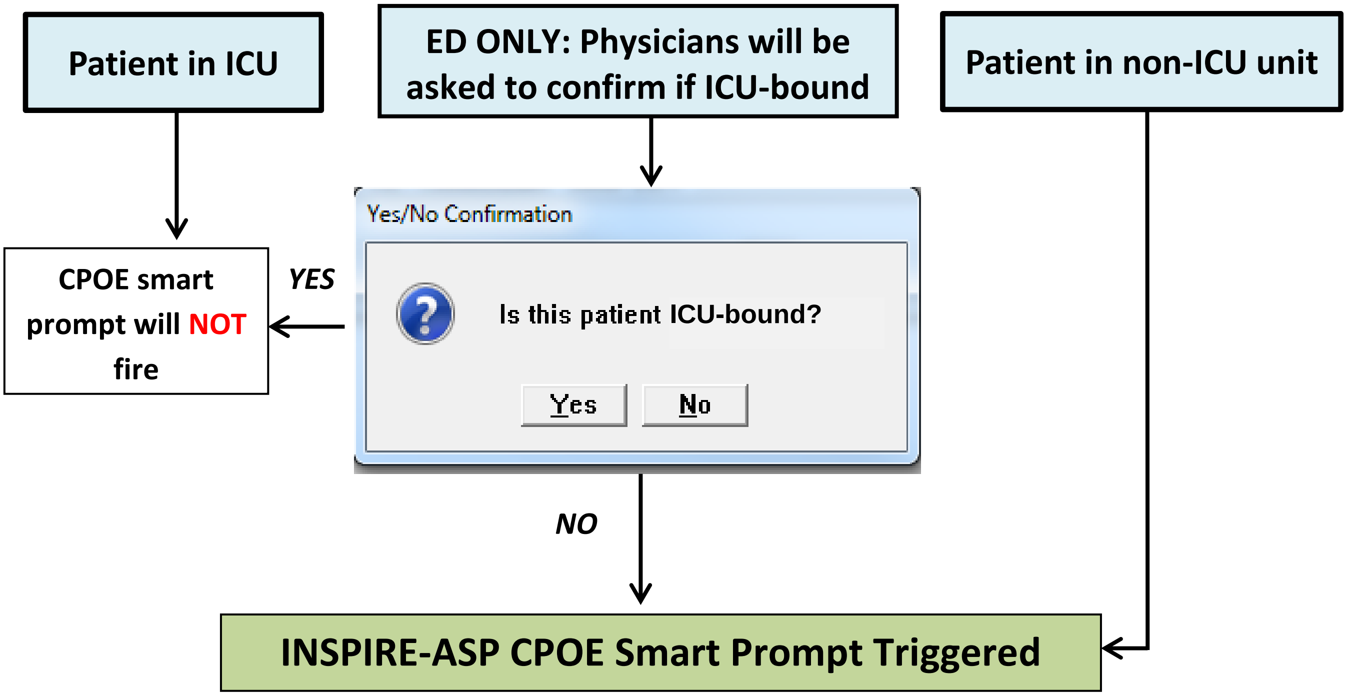

3. INSPIRE prompts will automatically look up the patient's specific risk for infection due to an MDRO.

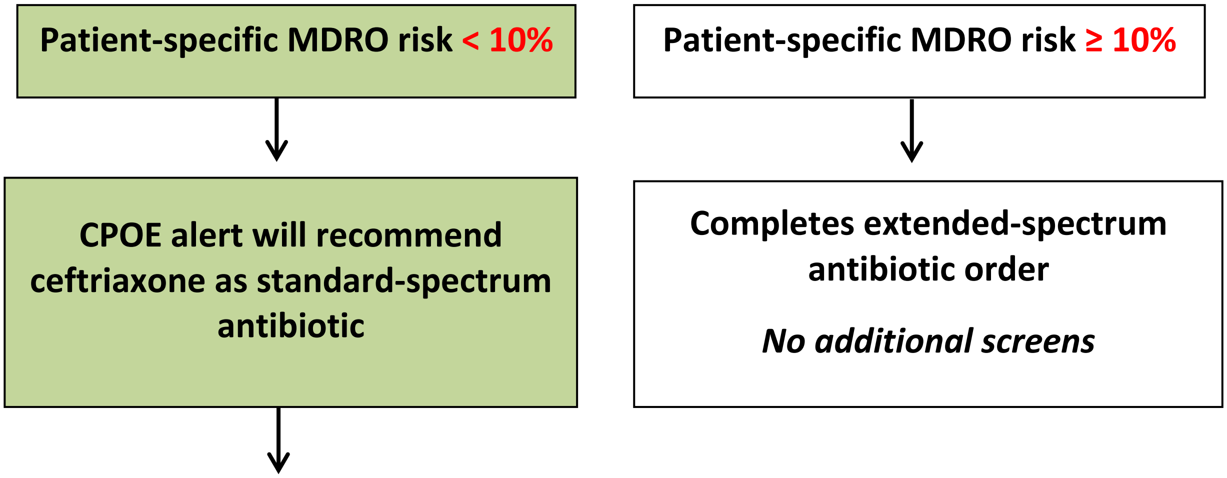

(See following page for mock INSPIRE-ASP CPOE smart prompt)

Mock CPOE Smart Prompt Recommendation Screen

<u>Example</u>:
Physician orders <u>cefepime</u> for pneumonia.
Patient risk for *Pseudomonas* pneumonia is calculated to be <10%.

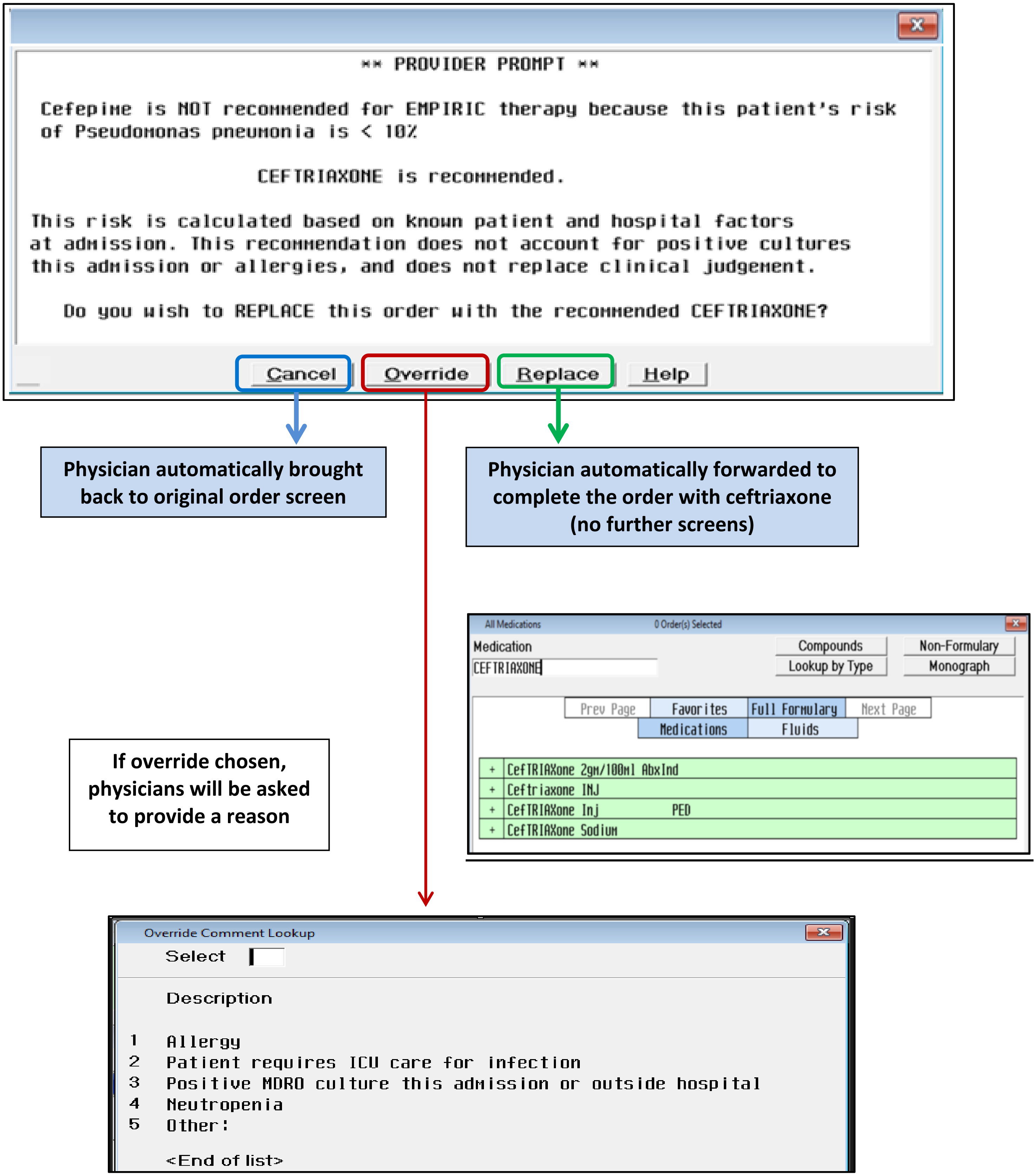

INSPIRE-ASP Quarterly Progress Reports

Regular monitoring and provider feedback are integral components of successful antibiotic stewardship interventions. The investigative team will partner with HCA Healthcare to provide study champions with quarterly clinician and hospital progress reports on a monthly basis. The goal of these progress reports is to:

- Help study champions identify whether additional education or follow-up is needed
- Help clinicians and hospitals track their overall progress

This information will be displayed in table and graph form and will contain a rolling quarter's worth of data and will include how often clinicians order extended-spectrum antibiotics for pneumonia or UTI, how often recommendations for standard antibiotics is accepted, and if overridden, what the chosen reason was for overriding. See below for examples of the tables and graphs that will be provided.

All data will be collected via the centralized HCA Clinical Data Warehouse.

Table 1: Quarterly Clinician Progress Reports - Example

Clinician	Total PNA Non-ICU Admissions N	Empiric Antibiotics Ordered Per Protocol N (%)	Smart Prompts Triggered N (%)	Smart Prompt Declined/ Overridden N (%)	Adherence with Smart Prompt Recommendation N (%)
**You**	12	8 (67)	4 (33)	3 (75)	9 (75)
**MD 1**	10	5 (50)	5 (50)	2 (40)	8 (80)
**MD 2**	9	3 (33)	6 (67)	4 (67)	5 (56)
**MD 3**	7	4 (57)	3 (43)	1 (33)	6 (86)
**MD 4**	3	2 (67)	1 (33)	1 (100)	2 (67)
**All Clinicians This Hospital**	41	22 (54)	19 (46)	11 (58)	30 (73)
**All Clinicians Arm 2 Trial**	1189	638 (54)	551 (46)	319 (58)	870 (73)

Table 2: Quarterly Clinician Overrides and Declinations - Example

Clinician	Reason for Declining or Overriding Smart Prompt				
	Allergy N (%)	Neutropenia N (%)	MDRO Culture Positive N (%)	ICU Bound N (%)	Other N (%)
You	1 (33)	0 (0)	1 (33)	1 (33)	0 (0)
MD 1	0 (0)	1 (50)	0 (0)	1 (50)	0 (0)
MD 2	1 (25)	1 (25)	0 (0)	1 (25)	1 (25)
MD 3	0 ()	0 (0)	1 (100)	0 (0)	0 (0)
MD 4	1 (100)	0 (0)	0 (0)	0 (0)	0 (0)
All Clinicians This Hospital	3 (27)	2 (18)	2 (18)	3 (27)	1 (9)
All Clinicians Arm 2 Trial	87 (27)	59 (18)	58 (18)	86 (27)	29 (9)

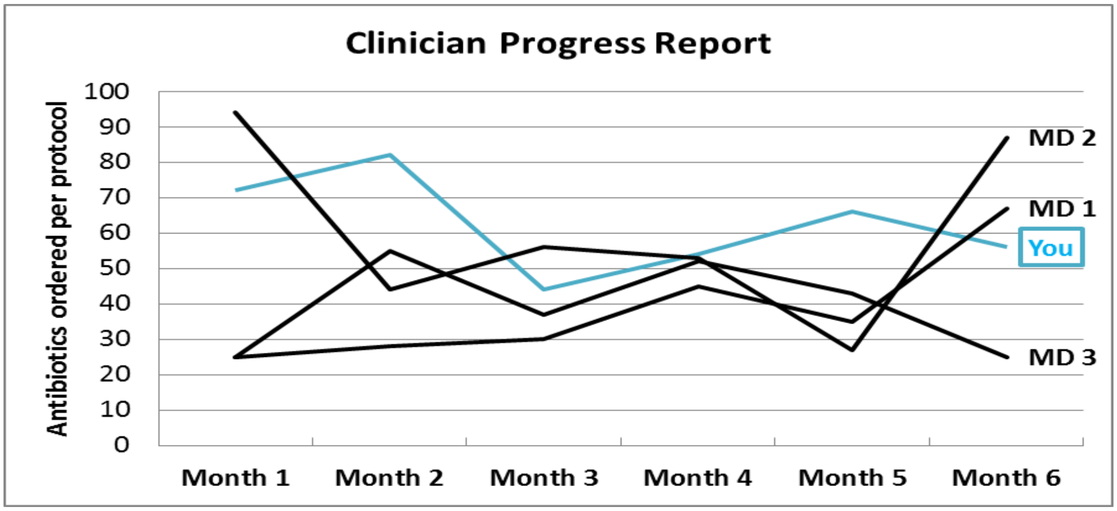

Figure 1: Quarterly Clinician Progress Report – Example

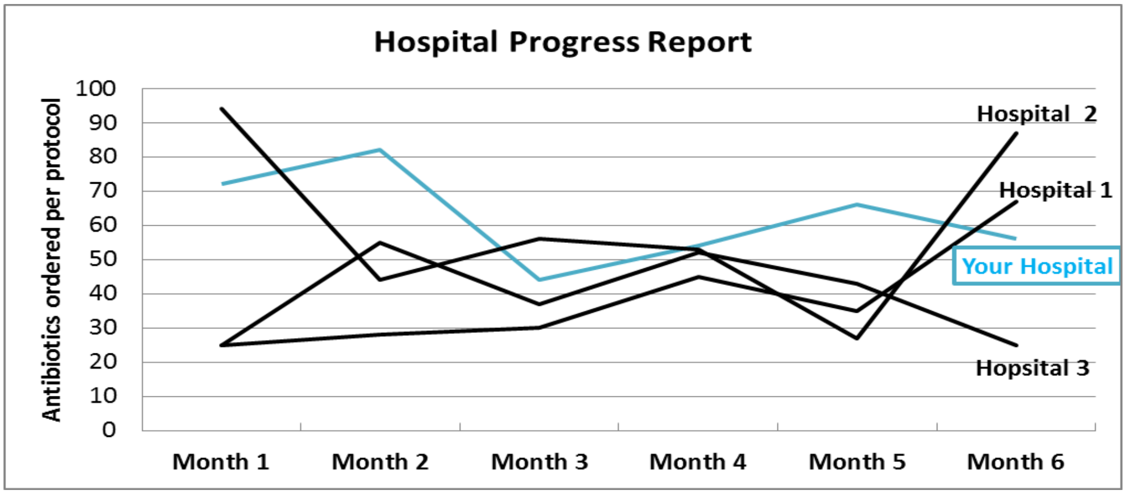

Figure 2: Quarterly Hospital Progress Report - Example

INSPIRE-ASP Trial

INtelligent Stewardship Prompts to Improve Real-time Empiric Antibiotic Selection for Patients

Arm 1 & 2
Coaching Calls
October, 2019

INSPIRE
BETTER SAFER ANTIBIOTIC USE

Agenda

- INSPIRE-ASP Brief Overview
- ATS-IDSA 2019 CAP Guidelines
- Polling Questions
- Question & Answer

INSPIRE-ASP Trial Arms

Arm 1: Routine Care – 30 Hospitals

- Standard-of-care antibiotic stewardship

Arm 2: Smart Prompt Alert Intervention – 29 Hospitals

- CPOE precision medicine alerts for patient-specific MDRO risk
- Recommends appropriate antibiotic choice
- Provider feedback reports

INSPIRE-ASP Population & Primary Outcome

Includes

- Adults admitted or being admitted for **PNA or UTI**
- ED and non-ICU units
- First 3 days of admission

Excludes

- ICU and pediatric patients
- Patients admitted from jail/prison

Intervention

- MDRO risk estimate calculates when antibiotic ordered

Primary Outcome

- Empiric extended-spectrum (ES) antibiotics in first 3 days of admission

What Antibiotics Are Considered "Extended-Spectrum"?

Category	Extended-Spectrum Antibiotics Included in CPOE Alerts
**Anti-MRSA**	Ceftaroline, Daptomycin, Linezolid[1], Vancomycin[2]
**Anti-VRE**	Daptomycin, Linezolid[1]
**Anti-Pseudomonas**	Aztreonam, Cefepime, Ceftazidime, Piperacillin/Tazobactam
**Anti-ESBL**	Ertapenem, Doripenem, Imipenem, Meropenem, Ceftolozane/Tazobactam,
**Anti-CRE**	Ceftazidime/Avibactam, Colistin, Polymixin B, Tigecycline

[1]Both oral and intravenous (IV) formulations
[2]IV formulation only

INSPIRE Timeline

- INSPIRE CPOE Intervention Launch: **October 1, 2018**
- Phase In: **6 months (October, 2018 – March, 2019)**
- Intervention Period: **15 months (April 1, 2019-June 30, 2020)**

Community Acquired Pneumonia
IDSA-ATS Guidelines 2019

CAP Guidelines 2019 - Scope

- Clinical guidelines only
  - Recommendations for diagnostic testing out of scope
- Recommendations for adults in US only
- Does not address:
  - Returning travelers from foreign countries
  - Immunocompromising conditions
  - Specific MDRO pneumonia management
- Severity of illness using IDSA/ATS CAP criteria retained

Metlay, et. al., Am J Respir Crit Care Med Vol 200, Iss 7, pp e45–e67, Oct 1, 2019

CAP 2019 - Sputum Culture Recommendations

- Sputum cultures not recommended unless (strong recommendation, very low quality evidence):
  - Patient hospitalized for severe CAP
  - Risk factors for MRSA, *Pseudomonas* (including local prevalence)
  - Recent hospitalization and parenteral antibiotics (90 days)

- "Most consistently strong predictor" is prior MDRO infection

CAP 2019 - Blood Culture Recommendations

- Blood cultures not routinely recommended unless (strong recommendation, very low quality evidence):
  - Patient hospitalized for severe CAP
  - Being empirically treated with anti-MRSA/anti-pseudomonas antibiotics
  - Previous known MRSA, pseudomonas
  - Recent hospitalization and parenteral antibiotics (90 days)

CAP 2019 Recommendations - Unintended Consequences

- Unintended consequence of restricting cultures to very sick/high risk patients:
  - Will skew MDRO prevalence to look high
  - May negatively impact de-escalation efforts
  - Possibly mitigated by recommendation to assess local prevalence among CAP patients, not cultures collected (supporting INSPIRE approach)

- Conflicts with sepsis guidelines recommendation to obtain blood cultures prior to antibiotics – cultures likely will be sent

CAP 2019 - Legionella/Streptococcus/Influenza Testing

- **Urine streptococcal** antigen testing not routinely recommended unless hospitalized for severe CAP
- **Urine *Legionella*** antigen testing not routinely recommended unless hospitalized for severe CAP, during outbreak, travel
- **Influenza** molecular assay (nucleic acid amplification) preferred over rapid diagnostic antigen test
- New, low cost genomic diagnostics expected to improve pathogen directed treatment

INSPIRE
BETTER SAFER ANTIBIOTIC USE

CAP 2019 - Serum Procalcitonin

- Empiric antibiotics should be started with clinically suspected and radiographically confirmed CAP regardless of procalcitonin level
- Higher procalcitonin associated with higher chance of bacterial vs. viral infection, but there is no clear threshold to differentiate
- Reported sensitivity – 30-90%
- Growing recognition viruses responsible for significant amount of CAP but more data needed for clinical use of procalcitonin

CAP 2019 - Use of Clinical Prediction Rules

- Recommend Pneumonia Severity Index (PSI) over CURB-65 to determine need for inpatient admission
  - PSI Identifies low risk patients well
  - Better predictor of mortality
  - Should be used in conjunction with clinical judgment
- Not recommended to determine inpatient treatment intensity
  - PSI/CURB-65 not designed to determine level of care
- Use 2007 IDSA Severe CAP Criteria to determine ICU admit
  - Patients requiring pressors or mechanical ventilation
  - Patients meeting >= 3 minor criteria
  - IDSA criteria outperform PSI/CURB-65

CAP 2019 - IDSA Severe CAP Criteria

- Severe CAP criteria includes either one major criterion or three or more minor criteria

Major criteria

- Septic shock with need for vasopressors
- Respiratory failure requiring mechanical ventilation

Minor criteria

- Respiratory rate ≥ 30 breaths/min
- Pa_{O2}/FI_{O2} ratio ≤ 250
- Multilobar infiltrates
- Confusion/disorientation
- Uremia (blood urea nitrogen level ≥ 20 mg/dl)
- Leukopenia* (white blood cell count < 4,000 cells/μl)
- Thrombocytopenia (platelet count < 100,000/μl)
- Hypothermia (core temperature < 36°C)
- Hypotension requiring aggressive fluid resuscitation

CAP 2019 - Outpatient Antibiotics for Empiric CAP

- Healthy, no comorbid conditions – very narrow antibiotic choices
- Pathogen directed if prior respiratory MRSA or *P. aeruginosa*
- No recent hospitalization **with** receipt of IV antibiotics in last 90d
- Removed 2007 strong recommendation for monotherapy with macrolide due to high level *S. pneumoniae* resistance > 30%

Table 3. Initial Treatment Strategies for Outpatients with Community-acquired Pneumonia

	Standard Regimen
No comorbidities or risk factors for MRSA or *Pseudomonas aeruginosa**	Amoxicillin or<br>doxycycline or<br>macrolide (if local pneumococcal resistance is <25%)†
With comorbidities‡	Combination therapy with<br>amoxicillin/clavulanate or cephalosporin<br>AND<br>macrolide or doxycycline§<br>OR<br>monotherapy with respiratory fluoroquinolone‖

CAP 2019 - Inpatient Empiric CAP Treatment

- Standard regimen regardless of severity unless prior respiratory isolation of MRSA or *P. aeruginosa*
- More strongly favors beta-lactam/macrolide over fluoroquinolone
- No anaerobic coverage for aspiration unless lung abscess or empyema

	Standard Regimen	Prior Respiratory Isolation of MRSA	Prior Respiratory Isolation of *Pseudomonas aeruginosa*
Nonsevere inpatient pneumonia*	β-Lactam + macrolide† or respiratory fluroquinolone‡	Add MRSA coverage§ and obtain cultures/nasal PCR to allow deescalation or confirmation of need for continued therapy	Add coverage for *P. aeruginosa*‖ and obtain cultures to allow deescalation or confirmation of need for continued therapy
Severe inpatient pneumonia*	β-Lactam + macrolide† or β-lactam + fluroquinolone‡	Add MRSA coverage§ and obtain cultures/nasal PCR to allow deescalation or confirmation of need for continued therapy	Add coverage for *P. aeruginosa*‖ and obtain cultures to allow deescalation or confirmation of need for continued therapy

CAP 2019 - Impact of Unintended Consequences

- If sputum and blood culture collection drops for the less ill, local antibiograms will disproportionately reflect the severely ill
- This could cause undue alarm among clinicians
- Recommendation is to calculate among CAP patients, not among cultures collected however perception from physicians may still be affected
- Example:
  - Suppose 100 patients seen with CAP by your ED
  - 10 are admitted to the hospital
  - 5 admitted to the ICU and/or have severe CAP. Among these, 3 grow out MRSA or *Pseudomonas*
  - This means 95 patients with CAP would get no cultures and meanwhile 60% of severe CAP patients have MDRO

CAP 2019 - "HCAP" Criteria Should Not Guide Treatment

- Recommendation to abandon "HCAP" category to guide use of extended spectrum antibiotics (strong recommendation)
- Cover MRSA or P. aeruginosa only if local risk factors present
- Individual risk factors for antibiotic resistant pathogen weakly associated with infection with these pathogen
- "Most consistently strong individual risk factor for respiratory infection with MRSA or P. aeruginosa are":
  - Prior isolation from respiratory sample
  - Recent hospitalization and exposure to parenteral antibiotics

CAP 2019 - Local MDRO Prevalence to Guide Treatment

- Recommend using local MDRO prevalence among patients with CAP, not cultures collected
- INSPIRE MDRO prevalence calculated among pneumonia patients with cultures collected within the empiric period
  - Specifically tailored for study purposes
  - This approach is valid in the era in which our study was done
  - Pneumonia cohort: mean 86% (SD, 5%) had cultures collected
  - UTI cohort: mean 88% (SD, 6%) had cultures collected

CAP 2019 - Recent Hospitalization With IV Antibiotics

- Non-severe CAP with prior hospitalization and IV antibiotics within 90 days – standard treatment, get nasal PCR/cultures, escalate if positive
- Severe CAP – add MRSA and *P. aeruginosa* coverage, de-escalate

	Recent Hospitalization and Parenteral Antibiotics and Locally Validated Risk Factors for MRSA	Recent Hospitalization and Parenteral Antibiotics and Locally Validated Risk Factors for *P. aeruginosa*
Nonsevere inpatient pneumonia*	Obtain cultures but withhold MRSA coverage unless culture results are positive. If rapid nasal PCR is available, withhold additional empiric therapy against MRSA if rapid testing is negative or add coverage if PCR is positive and obtain cultures	Obtain cultures but initiate coverage for *P. aeruginosa* only if culture results are positive
Severe inpatient pneumonia*	Add MRSA coverage§ and obtain nasal PCR and cultures to allow deescalation or confirmation of need for continued therapy	Add coverage for *P. aeruginosa*‖ and obtain cultures to allow deescalation or confirmation of need for continued therapy

CAP 2019 - Other Treatment Considerations

- Steroids not recommended for routine use in non-severe or severe CAP; endorsed only for CAP with refractory shock
  - Supports Surviving Sepsis Campaign recommendations
- Recommend anti-influenza treatment if influenza-positive, regardless of illness duration – inpatient and outpatient
- Antibacterial treatment with positive influenza recommended
  - Bacterial coinfection associated with mortality
  - Antibiotic choice should follow IDSA CAP 2019 guidelines
- Repeat chest x-ray not needed if resolves within 5-7 days

INSPIRE
BETTER SAFER ANTIBIOTIC USE

CAP 2019 - Antibiotic Duration

- Should be guided by clinical improvement and stability (vital signs, ability to eat, and normal mentation)
- **No less than 5 days**
- Higher mortality and poor clinical outcomes seen if patient fails to achieve stability **by treatment day 5**
  - Assess for drug resistant pathogen, abscess/empyema, or alternate infection source
- Longer course needed for complicated pneumonia (e.g. meningitis, endocarditis) or unusual pathogen
- If suspected MRSA or *pseudomonal* CAP – duration 7 days

CAP 2019 in the Context of INSPIRE

- INSPIRE approach in line with CAP 2019
- Incorporates local MDRO prevalence into smart prompt
- INSPIRE robust modeling approach found history of prior MDRO culture was among the most important risk factor
  - Prior hospitalization/antibiotics not predictive of MDRO above and beyond history of prior MDRO

Within the context of this trial, we are evaluating the safe use of standard spectrum in CAP patients who may have many various individual risk factors but who may not need extended-spectrum

Available Educational Resources

- Toolkit binder
  - HCA Atlas Connect: CSG: Research & Academic Affairs > Research Portfolio > INSPIRE

- **NEW** Powerpoint presentations incorporating slides on HCAP, Aspiration Pneumonia, and UTI

Supplement 1
INSPIRE Pneumonia Trial
Statistical Plan Amendments

<table>
<tr><th>Date</th><th>Summary of Statistical Plan Amendments</th></tr>
<tr><td>9/19/2018</td><td>Original</td></tr>
<tr><td>8/24/2019</td><td>Added language describing that safety outcome analysis will evaluate non-inferiority with a one-tailed significance set at alpha = 0.05 (to increase conservatism).</td></tr>
<tr><td>6/3/2020</td><td>Final
Refined analytic model to include multiple admissions per patient.
Updated language in response to Harrington et al (NEJM 2019;381:285-6) publication on “New Guidelines for Statistical Reporting in the Journal.”
Clarified two-tailed significance for secondary outcomes to be set at alpha = 0.025 to account for multiple comparisons
Improved layout for analytic plan and grammar
Converted outcome tables from a single table to three distinct tables (content same as original)
(All updates were created prior to accessing trial dataset for cleaning and analysis.)

Note: Upon attempting trial analysis, we encountered limitations in hardware and software that prevented implementing this theoretically possible analysis; we then selected a single random admission per patient.</td></tr>
</table>

(Originally published in Gohil, et al. Stewardship Prompts to Improve Antibiotic Selection for Pneumonia: The INSPIRE Randomized Clinical Trial. JAMA. 2024;331(23):2007–2017. doi:10.1001/jama.2024.6248)

INSPIRE-ASP Pneumonia Trial
Statistical Analysis Plan

September 19, 2018

Trial Outcomes

INSPIRE-ASP Pneumonia Trial Outcomes

The goal of this trial is to reduce unnecessary physician prescribing of empiric extended-spectrum antibiotics for pneumonia requiring hospitalization. Trial outcomes are outlined in the table below and will be applied to the study population of adult patients who have a primary or secondary claims code indicating pneumonia that is present on admission, who did not require direct admission from the ED to ICU and was treated with an antibiotic within 3 days of admission. Our primary outcome will be days of empiric extended-spectrum (ES) antibiotic treatment (days of therapy, DOT) per 1,000 at-risk-days. Days of therapy (DOT) is specific to each extended-spectrum antibiotic, where any dose is considered one day's worth of therapy. The ES antibiotics are then aggregated to obtain the total ES DOT. For example, if a patient is started on vancomycin and cefepime for 1 day, this will count as 2 ES DOT for that day. Multiple doses of the same antibiotic received on the same day are only counted as one day of therapy for that extended spectrum antibiotic. The empiric period is defined as within 3 calendar days of admission. This outcome will include antibiotics in Table 3.

Table 1: Primary and Secondary INSPIRE-ASP Pneumonia Trial Outcomes

Outcome	Metric
**Primary Manuscript**	
**Primary Outcome**	
Empiric Extended-Spectrum (ES) Antibiotic Use	Days of empiric ES antibacterial treatment for pneumonia per at-risk day, (first 3 days of admission)[1].
**Secondary Outcomes**	
Empiric Vancomycin Use	Days of empiric Vancomycin treatment for pneumonia per at-risk-day (first 3 days of admission)[1]
Empiric Antipseudomonal Use	Days of empiric antipseudomonal antibacterial treatment for pneumonia per at-risk-day (first 3 days of admission)[1,2]
**Safety Outcomes for Trial (Other Pre-specified Outcomes)**	
Antibacterial Escalation	Days from start of standard-spectrum antibacterial for pneumonia until switch to extended-spectrum antibacterial for pneumonia during hospital stay
ICU Transfers	Days from start of hospitalization until ICU transfer within hospital stay
Length of Stay	Hospital length of stay

[1]For extended-spectrum and antipseudomonal days of therapy (DOT), if more than one different type of antibiotic is given on the same day, then each contributes separately to the DOT.
[2]Does not include aminoglycosides or fluoroquinolones.

Primary Statistical Analysis

The main trial results will be based upon as-randomized, unadjusted analyses of empiric extended-spectrum (ES) antibiotic days of therapy (DOT) per at-risk day. In contrast to the national DOT measure defined by CDC for hospitals, we define DOT at the individual level so that we can perform analyses on individuals. We define a DOT for a particular ES antibiotic as a day in which any number of doses of that antibiotic is given. These are summed across at-

risk days and ES antibiotics within patient, then divided by the at-risk days within that patient to determine each patient's DOT per at-risk day. At-risk days are in the first three days of admission.

The trial will be assessed among the cohort of adult patients who have a primary or secondary claims code indicating pneumonia that is present on admission, who did not require direct admission from the ED to ICU and was treated with an antibiotic within 3 days of admission.

The primary outcome for the trial is ES DOT per at-risk day. Ordinarily, we would perform a Poisson or negative binomial analysis on the count of ES DOT, with an offset of the number of days over which these antibiotics were received. However, the standard metric is the DOT per day, and analysis of this outcome with no offset is equivalent to the count of days of therapy with an offset of the number of days. In analyses of the baseline data, performed before randomization, there was no hint of overdispersion in a Poisson model, and so we plan to use that model, rather than the negative binomial model, in the data analysis.

The planned model for the project is a generalized linear mixed effects model for differences in differences, with random effects accounting for correlation within cluster and period-varying random effects to allow for differences between hospitals from baseline to follow-up. The model can be expressed as follows:

$$log(y_{iph}) = \beta_0 + \beta_1 A_{iph} + \beta_2 T_{iph} + \beta_3 A_{iph} T_{iph} + b_{0h} + b_{1h} T_{iph}$$

where y_{iph} is the DOT per at-risk day for person i in period p at hospital h, and $A_{iph} = 1$ if hospital h is in the intervention arm and 0 if not, and $T_{iph} = 1$ if p is the intervention period and 0 if baseline period. The random effects b_{0h} and b_{1h} allow for different baseline mean ES DOT per at-risk day for each hospital and each hospital per period, respectively. Equivalently, they allow for correlation within hospital at different levels at baseline and at follow-up.

The assessment of trial success will be determined by the significance of the arm by period interaction term β_3, which assesses whether the log relative rate of the outcome due to being in the intervention arm in the intervention period is different from 0. The exponentiated parameter estimate for β_3 is the estimated relative rate of DOT per at-risk day due to the intervention, relative to the baseline period. For example, if β_3 had a negative value and a p-value <0.05, we would conclude that the physician-specific CPOE smart prompts generated a benefit over routine care. Exponentiating the parameter value would give us an estimate of the relative reduction due to the intervention in the expected DOT per at-risk day.

Subsequent analyses will include both as-treated and adjusted models. Adjusted models will account for individual characteristics such as age, gender, comorbidities, and prior history of MDROs as well as hospital characteristics such as hospital antibiotic resistance. We will also account for randomization strata. All analyses will be performed using current versions of SAS (Version 9.4, as of writing, SAS Institute, Cary NC) and/or R (Version 3.3.0, as of writing).

Analysis plan amended with the following paragraph on 8/24/19:

Safety outcomes noted in the above table will be assessed in the most conservative manner to identify potential safety issues. Each safety outcome will be evaluated for non-inferiority using an as-randomized unadjusted model with a one-tailed significance set at alpha = 0.05.

Power Size/Sample Size Calculations

Power assessment proceeded as follows, using a Monte Carlo approach. We used data from the baseline period of 10/1/2015-12/31/2016 in a bootstrap procedure. Individuals were selected with replacement from within each hospital, once to represent baseline data and separately to represent intervention period data. In the randomization portion of the Monte Carlo approach, we ordered the hospitals by "size", meaning the number of patients with pneumonia. Then, within strata of 6 hospitals, we ordered by DOT per at-risk day. Then we took pairs of hospitals with adjacent DOT per at-risk day and assigned one member of the pair to one arm. To do this, a single pseudo-random number uniformly distributed between 0 and 1 was generated for each pair. If it was less than 0.5, the arbitrary "first" member of the pair was assigned to Routine Care and the other to Intervention. If it was greater than or equal to 0.5, then the assignments were reversed. The remaining unpaired hospital of the 59 participants was assigned as the "first" member of a pair with no match.

If a patient was in the intervention arm in the intervention period, then their DOT per at-risk day was reduced by a proportion, given below. The reduction of ES DOT per at-risk day was selected with a lower bound determined by the investigators as the hoped-for effect of the intervention with minimal clinical significance, and the impact in the Monte Carlo calculation on a per-patient basis was to reduce the days of therapy proportionately between this number and 1, on a uniform distribution. In other words, if the effect of the intervention was to multiply doses by as little as .9, each person would have their doses multiplied by between .9 and 1. Then an integer value of doses would be chosen as described above.

We are interested in determining whether the patient-specific CPOE intervention reduces the average days of empiric extended-spectrum antibiotics by at least 12.5% relative to usual care. Using the method above, we have 97% power (CI 91-99%) to detect this effect. Table 2 below shows the calculated power and confidence intervals for the primary and secondary outcomes to be evaluated in our primary manuscript.

Table 2. Power Calculation – Primary and Secondary Outcomes

**Primary Trial Outcome**	**Power (CI), %**
Days of empiric ES antibiotic treatment per at-risk day, (within 3 days of admission).	97 (91-99)
**Secondary Trial Outcomes**	
Days of empiric Vancomycin treatment per at-risk-day (first 3 days of admission)	92 (85-96)
Days of empiric anti-Pseudomonas antibiotic treatment per at-risk-day (first 3 days of admission)	84 (77-91)

Extended-spectrum antibiotics are defined in the study protocol and provided below for convenience.

Table 3: INSPIRE-ASP Pneumonia Extended-Spectrum Antibiotics

**Pathogen-Directed Antibiotic Category**	**Extended-Spectrum Antibiotics Targeted in INSPIRE Pneumonia CPOE Prompts**
**Anti-MRSA**	Ceftaroline, Daptomycin[1], Linezolid[2], Vancomycin[3]
**Anti-VRE**	Daptomycin, Linezolid[2]
**Anti-*Pseudomonas***	Aztreonam, Cefepime, Ceftazidime, Piperacillin/Tazobactam

**Anti-ESBL**	Ertapenem, Ceftolozane/Tazobactam, Meropenem, Imipenem
**Anti-CRE**	Ceftazidime/Avibactam, Colistin, Imipenem/Relebactam, Meropenem/Vaborbactam, Polymixin B, Tigecycline

[1]Daptomycin not an accepted pneumonia treatment but included in trial analysis in case ordered.
[2]Both oral and intravenous (IV) formulations.
[3]IV formulation only.
Abbreviations: **CPOE** – Computerized Physician Order Entry, **MRSA -** Methicillin Resistant *Staphylococcus aureus,* **ESBL –** Extended Spectrum Beta Lactamase Producing *Enterobacterales,* **VRE –** Vancomycin Resistant *Enterococci*, **CRE –** Carbapenem Resistant *Enterobacterales.*

(Originally published in Gohil, et al. Stewardship Prompts to Improve Antibiotic Selection for Pneumonia: The INSPIRE Randomized Clinical Trial. JAMA. 2024;331(23):2007–2017. doi:10.1001/jama.2024.6248)

INSPIRE-ASP Pneumonia Trial
Statistical Analysis Plan

June 3, 2020

Background

The **INSPIRE-ASP Pneumonia Trial** (INtelligent Stewardship Prompts to Improve Real-time Empiric Antibiotic Selection for Patients with Pneumonia) is a cluster-randomized trial of HCA Healthcare (HCA) affiliated hospitals to assess best practice in antibiotic stewardship for adult hospitalized patients admitted to non-critical care units with pneumonia (PNA). It compares routine care to the use of a real-time smart prompt that provides a patient's specific risk of antibiotic-resistant pneumonia and alerts the clinician during the antibiotic ordering process to optimize prescribing and ideally improve patient outcomes. The main goal is to see if there is a difference in the empiric antibiotic prescribing practices for extended-spectrum antibiotics (ES). We define antibiotics administered during the first three days of hospitalization as empiric treatment because infecting pathogens and their antimicrobial susceptibilities are typically not known during this period. We refer to these first three days as the empiric period.

Participating hospitals are randomized to:

- **Arm 1 – Routine Care**
- **Arm 2 – Real-Time Risk Estimation Smart Prompt**
  Precision medicine smart prompt using a computerized physician order entry (CPOE) alert that gives a patient-specific risk estimate of antibiotic resistant infection and recommends standard-spectrum antibiotics in patients admitted for PNA who are found to have low risk (<10%) for recovery of a drug resistant pathogen.

Trial Outcomes

Trial outcomes evaluating intervention effectiveness are found in Table 1 below and trial safety outcomes are found in Table 2 below.

Table 1: Primary and Secondary INSPIRE-ASP Pneumonia Trial Outcomes

Outcome	Metric
**Primary Trial Outcome**	
Extended-Spectrum Days Of Antibacterial Therapy (ES-DOT) per Empiric Day	The summed number of different extended-spectrum antibacterials received each empiric day, measured repeatedly over the first three days of an admission and divided by the number of empiric days of the admission. An empiric day is a day within the first three days of an admission.[1]
**Secondary Trial Outcomes**	
Vancomycin Days of Antibacterial Therapy per Empiric Day	The summed number of days of Vancomycin received each empiric day per at-risk-day (first 3 days of admission)[1]
Antipseudomonal Antibiotic Days Of Therapy (ES-DOT) per Empiric Day	The summed number of different antipseudomonal antibacterials received each empiric day, measured repeatedly over the first three days of an admission and divided by the number of empiric days of the admission. An empiric day is a day within the first three days of an admission.[1,2]

[1]E.g., if a patient is admitted for 2 days, ES-DOT will be calculated for 2 days and divided by 2 empiric days; conversely if a patient is admitted for 4 days, only the first 3 days will be evaluated.

[2]Does not include aminoglycosides or fluoroquinolones.

Safety outcomes planned for the primary manuscript are shown in Table 2. Safety outcomes will be analyzed separately from the main effectiveness outcomes of the trial (see Primary Statistical Analysis section below).

Table 2: Other Pre-Specified Outcomes – Safety Trial Outcomes

**Safety Trial Outcomes (other pre-specified outcomes)**	
Antibacterial Escalations [Safety Outcome]	Days from start of standard-spectrum antibacterial until switch to extended-spectrum antibacterial during hospital stay
ICU Transfers [Safety Outcome]	Days from start of hospitalization until ICU transfer within hospital stay
Length-of-stay [Safety Outcome]	Days from hospital admission to discharge

Primary Statistical Analysis

The primary trial outcome is defined as the summed number of different extended spectrum antibiotics received each day, measured repeatedly over the first three days of an admission and divided by the number of days of the admission within the empiric period. An empiric day is a day within the first three days of an admission. We define this outcome as an Extended-Spectrum Days Of Therapy (ES-DOT) per empiric day. For clarity the calculation is as follows: we define a DOT for a particular ES antibiotic as a day in which any number of doses of that antibiotic is given. Different ES antibiotics are summed across the empiric days for each patient admission, then divided by the empiric days for that patient-admission to determine each admission's DOT per at-risk day. If an admission is less than 3 days, only the number of days the patient is admitted will contribute to the numerator and denominator. Antibiotics given during an associated emergency department visit on the date of hospital admission are counted toward the ES-DOT of the first hospital day.

The main trial results will be based upon as-randomized, unadjusted analyses of admission-level ES-DOT per empiric day. We define ES-DOT per empiric day at the individual admission level so that we can perform analyses on individuals.

The trial will be assessed among the cohort of adult admissions who have an ICD-10 claims code indicating pneumonia is present on admission, who received any antibiotic within 3 days of admission, and who were admitted to a non-ICU location. For admissions initially on a non-ICU floor and transferred to the ICU, analysis will include only admission days on the non-ICU floor within 3 days of admission.

The trial periods are defined as follows: (1) Baseline – April 1, 2017 – September 30, 2018 (18 months); (2) Phase-in – October 1, 2018 – March 31, 2019 (6 months); (3) Intervention – April 1, 2019 – June 30, 2020 (15 months).

The unit of analysis will be individual admissions. Individuals can contribute more than one admission. The analytic model will be a generalized linear mixed effects model for differences in differences, with random effects accounting for correlation within cluster, period-varying random effects to allow for differences between hospitals from baseline to follow-up, and admission-level random effects to account for correlation within person and hospital. Analyses of the baseline data, performed before randomization, found no evidence of overdispersion in a Poisson model, and so we plan to use that model for analysis of outcomes. The model can be expressed as follows:

$$log(y_{ijph}) = \beta_0 + \beta_1 A_{ijph} + \beta_2 T_{ijph} + \beta_3 A_{ijph} T_{ijph} + b_{0h} + b_{1h} T_{ijph} + g_j$$

where y_{ijph} is the ES-DOT per at-risk day for patient i for admission j in period p at hospital h, and $A_{ijph} = 1$ if hospital h is in the intervention arm and 0 if not, and $T_{ijph} = 1$ if p is the intervention period and 0 if baseline period. The random effects b_{0h} and b_{1h} allow for different baseline mean admission-level ES-DOT per at-risk day for each hospital and each hospital per period, respectively, while g_j allows different admissions to have different mean ES-DOT per at-risk day across multiple admissions. Equivalently, they allow for correlation within hospital at different levels at baseline and at follow-up.

The assessment of trial success will be determined by the significance of the arm by period interaction term β_3, which assesses whether the log relative rate of the outcome due to being in the intervention arm in the intervention period is different from 0. The exponentiated parameter estimate for β_3 is the estimated relative rate of ES-DOT per at-risk day due to the intervention, relative to the baseline period. For example, if β_3 had a negative value and a p-value <0.05, we would conclude that the patient-specific CPOE smart prompts generated a benefit over routine care. Exponentiating the parameter value would provides an estimate of the relative reduction due to the intervention in the expected ES-DOT per at-risk day.

The primary trial analysis will use an as-randomized unadjusted model with two-tailed significance set at alpha = 0.05. Secondary outcomes also will be assessed using an as-randomized unadjusted model with adjustment for multiple comparisons with two-tailed significance set at alpha = 0.025 for the two secondary outcomes. Subsequent analyses of the primary and secondary outcomes will include both as-treated and adjusted models. Adjusted models will account for individual characteristics such as age, gender, comorbidities, and prior history of MDROs as well as hospital characteristics such as hospital antibiotic resistance. We will also account for randomization strata and for seasonality. These analyses will be reported as point estimates with confidence intervals without p-value. The reason for including these analyses is to provide additional information related to the trial outcomes for readers to assess the effects of potential confounders. The reason to not include them in a formal multiple comparisons adjustment is because these analyses are non-independent evaluations relative to the as-randomized unadjusted analyses.

Safety outcomes noted in the above table will be assessed in the most conservative manner to identify potential safety issues. This is required because a reduction in ES-DOT might be achieved only by incorrectly withholding ES antibiotics when they were really needed. Each safety outcome will be evaluated for non-inferiority using an as-randomized unadjusted model with a one-tailed significance set at alpha = 0.05. Analyses planned for these assessments are proportional hazards models with random effects (to account for hospital effects). These models are sometimes called frailty or shared frailty models. Because these are safety outcomes, we do not intend to make adjustments for multiple comparisons in testing them, further increasing conservatism.

All analyses will be performed using current versions of SAS (Version 9.4, as of writing, SAS Institute, Cary NC) and/or R (Version 4.0, as of writing).

Power Size/Sample Size Calculations

Power assessment proceeded as follows, using a Monte Carlo approach. We used available data to define a baseline period of 10/1/2015-12/31/2016 in a bootstrap procedure. Individuals were selected with replacement from within each hospital, once to represent baseline data and separately to represent intervention period data. In the randomization portion of the Monte Carlo approach, we ordered the hospitals by “size”, meaning the number of

admissions with pneumonia. Then, within strata of 6 hospitals, we ordered by ES-DOT per at-risk day. Then we took pairs of hospitals with adjacent ES-DOT per at-risk day and assigned one member of the pair to one arm. To do this, a single pseudo-random number uniformly distributed between 0 and 1 was generated for each pair. If it was less than 0.5, the arbitrary "first" member of the pair was assigned to Routine Care and the other to Intervention. If it was greater than or equal to 0.5, then the assignments were reversed. The remaining unpaired hospital of the 59 units were assigned as the "first" member of a pair with no match. (Three sets of hospitals each shared a stewardship program and these hospitals were treated as a single hospital within the routine.)

If an admission was in the intervention arm in the intervention period, then their ES-DOT per at-risk day was reduced by a proportion, given below. The reduction of ES-DOT per at-risk day was selected with a lower bound determined by the investigators as the hoped-for effect of the intervention with minimal clinical significance, and the impact in the Monte Carlo calculation on a per-admission basis was to reduce the days of therapy proportionately between this number and 1, on a uniform distribution. In other words, if the effect of the intervention was to multiply doses by as little as .9, each person would have their doses multiplied by between .9 and 1. Then an integer value of doses would be chosen as described above.

We are interested in determining whether the patient-specific CPOE intervention reduces ES-DOT per at-risk day by at least 12.5% relative to usual care. Using the method above, we have 97% power (CI 91-99%) to detect this effect. Table 4 below shows the calculated power and confidence intervals for the primary and secondary outcomes to be evaluated in our primary manuscript.

Table 4. Power Calculation – Primary and Secondary Outcomes

**Primary Trial Outcome**	**Power (CI), %**
Extended-Spectrum Antibacterial Days Of Therapy (ES-DOT) per Empiric Day	97 (91-99)
**Secondary Trial Outcomes**	
Vancomycin Days of Therapy per Empiric Day	92 (85-96)
Antipseudomonal Antibiotic Days Of Therapy per Empiric Day	84 (77-91)

Extended-spectrum antibiotics are defined in the study protocol and provided below for convenience.

Table 5: INSPIRE-ASP Pneumonia Extended-Spectrum Antibiotics

**Pathogen-Directed Antibiotic Category**	**Extended-Spectrum Antibiotics Targeted in INSPIRE Pneumonia CPOE Prompts**
**Anti-MRSA**	Ceftaroline, Daptomycin[1], Linezolid[2], Vancomycin[3]
**Anti-VRE**	Daptomycin, Linezolid[2]
**Anti-*Pseudomonas***	Aztreonam, Cefepime, Ceftazidime, Piperacillin/Tazobactam
**Anti-ESBL**	Ertapenem, Ceftolozane/Tazobactam, Meropenem, Imipenem
**Anti-CRE**	Ceftazidime/Avibactam, Colistin, Imipenem/Relebactam, Meropenem/Vaborbactam, Polymixin B, Tigecycline

[1]Daptomycin not an accepted pneumonia treatment but included in trial analysis in case ordered.
[2]Both oral and intravenous (IV) formulations.
[3]IV formulation only.

Abbreviations: **CPOE** – Computerized Physician Order Entry, **MRSA -** Methicillin Resistant *Staphylococcus aureus,* **ESBL –** Extended Spectrum Beta Lactamase Producing *Enterobacterales,* including multi-drug *Acinetobacter* and *Pseudomonas* species susceptible to Carbapenem. **VRE –** Vancomycin Resistant *Enterococci*, **CRE –** Carbapenem Resistant *Enterobacterales*

[1]Daptomycin not an accepted pneumonia treatment but included in trial analysis in case ordered.

[2]Both oral and intravenous (IV) formulations.

[3]IV formulation only.

Abbreviations:

MRSA - Methicillin Resistant *Staphylococcus aureus*
VRE – Vancomycin Resistant *Enterococci*
MDR- Multi-Drug Resistant
ESBL – Extended Spectrum Beta Lactamase producer
CRE – Carbapenem Resistant *Enterobacteriaceae*
GNR – Gram Negative Rod

Addendum 3/11/24:

The sentence below has an inadvertent error. In the following, the term "strata" should read "unit", and means that adjusted analyses will include random effects for hospital:
"Adjusted models will account for individual characteristics such as age, gender, comorbidities, and prior history of MDROs as well as hospital characteristics such as hospital antibiotic resistance. We will also account for randomization strata and for seasonality."

INSPIRE Urinary Tract Infection
Trial Supplement 1

(Originally published in Gohil, et al. Stewardship Prompts to Improve Antibiotic Selection for Urinary Tract Infection: The INSPIRE Randomized Clinical Trial. JAMA. 2024;331(23):2018–2028. doi:10.1001/jama.2024.6259)

Table of Contents

1. **INSPIRE Urinary Tract Infection (UTI) Trial Protocol**
    a. Original Toolkit – INSPIRE Stewardship Group
    b. Original Toolkit – Routine Stewardship Group
    c. Final Protocol – N/A, same as original protocol
    d. Summary of changes – N/A, no changes

2. **Statistical Analysis Plan**
    a. Summary of Changes
    b. Original Statistical Plan – 9/19/18
    c. Final Statistical Plan – 6/3/20

Arm 1:
Routine Stewardship

INSPIRE-ASP Pneumonia Trial
and
INSPIRE-ASP UTI Trial

Protocol & Toolkit Binder

Funded by the Centers for Disease Control and Prevention (CDC)

Welcome

Welcome to the **INSPIRE-ASP** (**IN**telligent **S**tewardship **P**rompts to **I**mprove **R**eal-time **E**mpiric **A**ntibiotic **S**election for **P**atients) Trials. The INSPIRE-ASP Pneumonia and INSPIRE-ASP Urinary Tract Infection (UTI) Trials are separate 2-Arm cluster-randomized trials of 59 HCA Healthcare hospitals to assess best practice in antibiotic stewardship. These trials are a partnership between HCA Healthcare and academic investigators who are federally funded by the Centers for Disease Control and Prevention (CDC).

Summary of Goals

Up to 40% of patients who are prescribed antibiotics in U.S. hospitals either do not require these drugs or receive inappropriate antibiotics for the condition being treated. Moreover, inappropriate antibiotics are often broad-spectrum, empirically prescribed due to concerns about infection with multidrug-resistant organisms (MDROs), despite the low risk of MDRO infection in the vast majority of clinical settings. This is the case for patients admitted with pneumonia and urinary tract infections (UTIs), two of the most common conditions for antibiotic use. This trial will compare two strategies to determine best practices for empiric antibiotic selection. Arm 1, Routine Care, will continue antibiotic stewardship practices according to current facility strategies. Arm 2, Smart Prompt Alert Intervention, will implement a precision medicine smart prompt using a computerized physician order entry (CPOE) alert that provides clinicians with a patient's probability of MDRO infection and recommends a standard-spectrum antibiotic choice in patients admitted for pneumonia or UTIs in non-critical care units. This trial will provide essential information to clinicians in real-time to support and optimize standard-spectrum empiric antibiotic use.

Funded by the Centers for Disease Control and Prevention (CDC)

INSPIRE-ASP Pneumonia and UTI Trials

***IN**telligent **S**tewardship **P**rompts to **I**mprove **R**eal-time **E**mpiric **A**ntibiotic **S**election for **P**atients*

The **INSPIRE-ASP Trials** are a **two-in-one** set of cluster-randomized trials at HCA Healthcare (HCA) hospitals to assess best practice in antibiotic stewardship for patients admitted to non-critical care units for pneumonia (PNA) and urinary tract infection (UTI). It tests the use of smart prompts to optimize prescribing and improve patient outcomes. Participating hospitals will be randomized to:

- **Arm 1 – Routine Care**
- **Arm 2 – Smart Prompt**

  Precision medicine smart prompt using a computerized physician order entry (CPOE) alert that gives a ***patient-specific risk estimate of antibiotic resistant infection*** and recommends standard-spectrum antibiotics in patients admitted for PNA or UTI who are found to have low risk (<10%) for drug resistant infection

Rationale

- Most non-ICU adult patients hospitalized with pneumonia or UTI are low risk for multidrug-resistant organisms (MDROs) and do not need broad-spectrum antibiotics.
- Identifying patients at low vs high risk of MDROs can decrease use of extended-spectrum antibiotics, reduce selection of resistant bacteria, and reduce *C. difficile* infections.

CPOE Details

- MDRO risk estimates are based upon patient-specific risk factors and local antibiograms
- We assessed demographics, comorbidities, MDRO history, prior antibiotic use, prior nursing home stay, and a variety of laboratory values to create a prediction algorithm
- Antibiotic recommendations are based on MDRO risk estimates and approved by trial steering committee, HCA leadership, and CDC experts.
- Recommendations support proper antibiotic choice but do not replace clinical judgment

Outcomes

- Primary: Empiric use of extended-spectrum antibacterials (first 3 days of admission)
- Secondary: Empiric use of (a) vancomycin and (b) antipseudomonal antibacterials
- Safety: Antibacterial escalation, ICU transfer, and length-of-stay
- Outcomes for other manuscripts: *C. difficile* infection, MDRO prevalence, cost-analysis

Anticipated Timeline

- 6 Month Phase In: October 2018 – March 2019
- 15 Month Intervention: April 2019 – June 2020

QUESTIONS? Study Investigators & Coordinators
HCAINSPIRE@gmail.com
844-522-4677

HCA

Funded by the Centers for Disease Control and Prevention (CDC)

INSPIRE-ASP – ARM 1

HCA ED and Inpatient Indication Screens: Clinician Work Flow

The requirement for providing an indication for antibiotic use is a national standard for antibiotic stewardship. HCA Healthcare (HCA) originally released antibiotic indication screens in 2017 and are currently implemented in inpatient areas throughout HCA facilities nationwide. This year, updates were made in these indication screens to allow the Emergency Department (ED) to be included.

Beginning **October 1, 2018** our hospital will launch HCA Antibiotic Indication Screens **in the Emergency Department.**

Ordering an antibiotic will now need an indication when ordered in the ED and on inpatient wards. This document provides details on the HCA antibiotic indication screen clinician workflow with relevant screen shots.

1. **HCA Antibiotic Indication Screens** – physicians will be asked to choose an indication for each antibiotic they order.

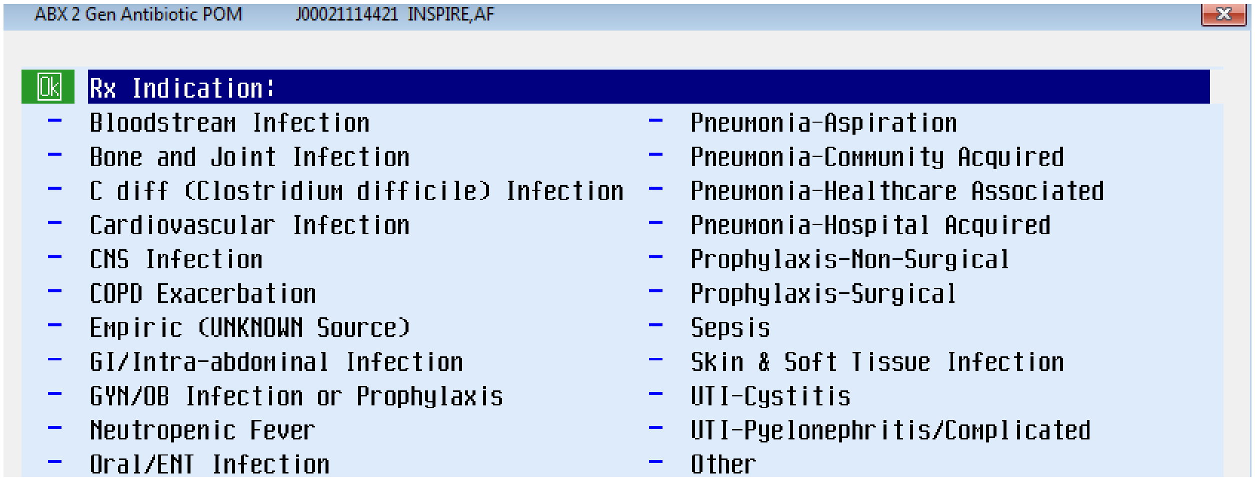

2. If “Empiric Unknown” or “Sepsis” are selected, then you will be asked what type of infection you suspect the patient may have and whether it is suspected to be community, healthcare, or hospital acquired:

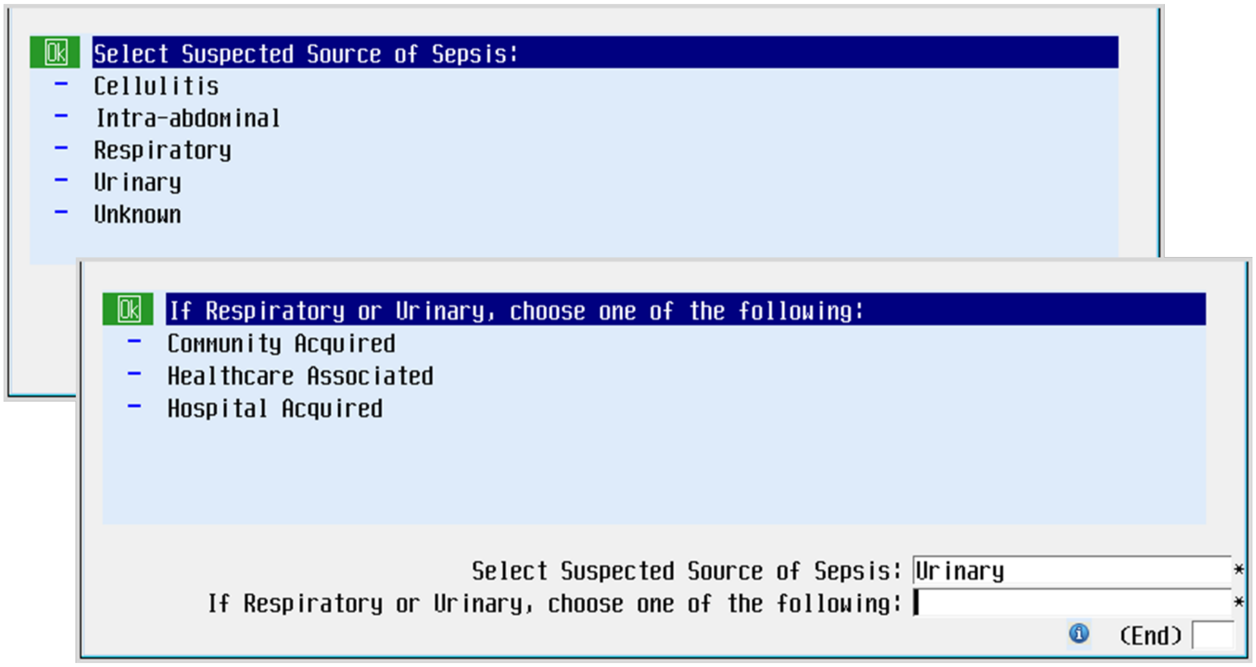

3. If “Pneumonia-Aspiration” or “UTI” are selected, you will be asked whether it is suspected to be community, healthcare, or hospital acquired:

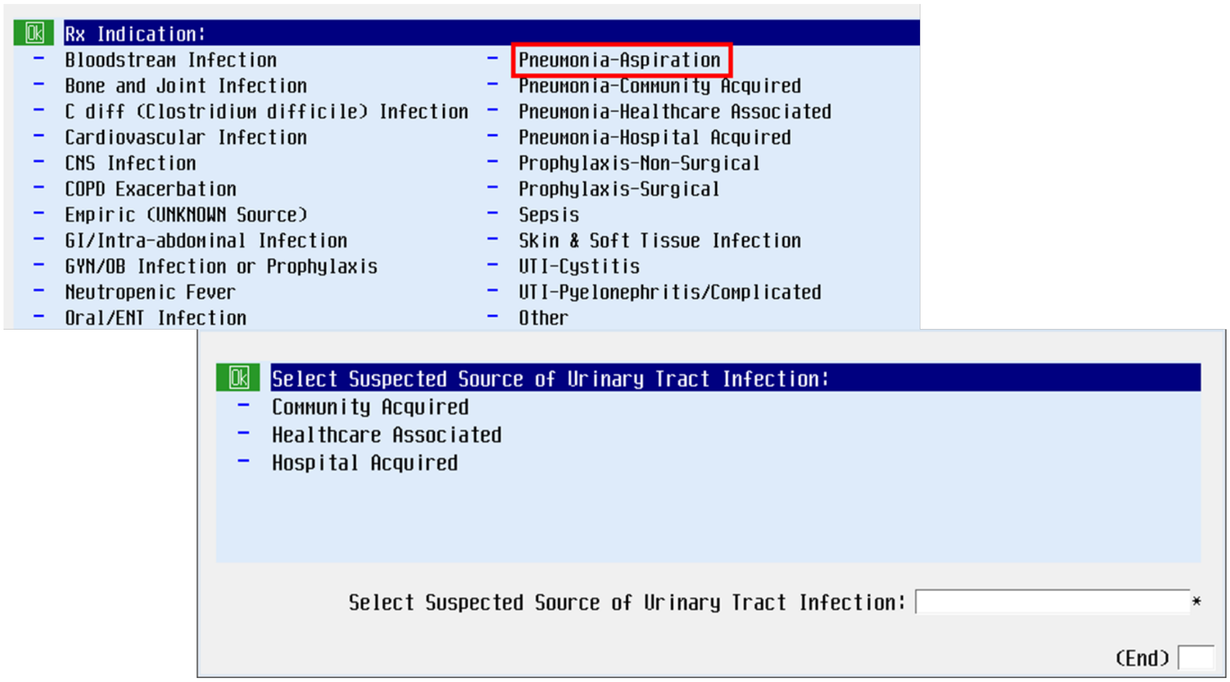

4. There is one additional screen that will be required for Arm 1 INSPIRE facilities that is unique to the trials. This screen asks the ED physician whether the patient is ICU-bound and is ONLY

triggered for patients in the ED in whom extended-spectrum antibiotics are prescribed for the following indications: pneumonia, UTI, empiric (unknown), or sepsis.

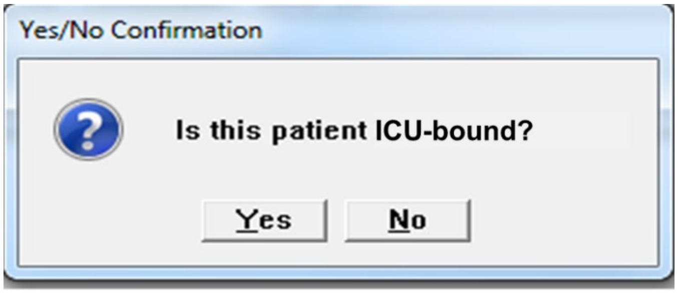

This final screen was necessary for the INSPIRE Arm 2 facilities to ensure adequate exclusion of ICU patients in the intervention arm. It was included for Arm 1 facilities to ensure that both arms are balanced and can be compared properly.

INSPIRE-ASP Trial

INtelligent Stewardship Prompts to Improve Real-time Empiric Antibiotic Selection for Patients

Arm 1 Coaching Call

September 13, 2018

Agenda

- INSPIRE Trials overview
- HCA Meaningful Use indication screens
- Polling questions
- Question & answer

INSPIRE-ASP Trial Arms

Arm 1: Routine Care – 30 Hospitals

- Standard-of-care antibiotic stewardship

Arm 2: Smart Prompt Alert Intervention – 29 Hospitals

- CPOE precision medicine alerts for patient-specific MDRO risk
- Recommends appropriate antibiotic choice
- Provider feedback reports

INSPIRE-ASP Population & Primary Outcome

Includes

- Adults admitted or being admitted for **PNA or UTI**
- ED and non-ICU units
- First 3 days of admission

Excludes

- ICU and pediatric patients

Intervention

- MDRO risk estimate calculates when antibiotic ordered

Primary Outcome

- Extended-spectrum (ES) antibiotics: first 3 days of admission

Trial Timeline

- Randomization: **March, 2018**
- Site visits: **Summer-Fall, 2018**
- Intervention launch: **October 1, 2018**
- Phase In: **6 months**
- Intervention: **15 months**

HCA Meaningful Use Indication Screens

- National guidance to institute meaningful use indications
  - CDC NHSN issued declaration of readiness
  - Leapfrog metric
- HCA indication screens released to inpatient areas 2017
  - Widely implemented throughout HCA
  - Well accepted by inpatient physicians
  - Planned for release in ED enterprise wide
- In Arm 2, INSPIRE CPOE prompts trigger off indication screens
  - Indication screens will be active in both ED and inpatient
- To ensure Arms 1 and 2 are equally balanced, the indication screens will need to be active in both study arms
  - ED Indication screens go live **October 1st** for BOTH arms

HCA Indication Screens Trigger CPOE Smart Prompt

- INSPIRE prompts triggered (Arm 2 only) based on indication:
  - **PNA**, **UTI**, **Sepsis/Empiric** – triggers additional screen asking if possible respiratory or urinary source

- **Indication screens** will be live in **both Arm 1 and Arm 2**

ABX 2 Gen Antibiotic POM J00021114421 INSPIRE,AF

Ok **Rx Indication:**

– Bloodstream Infection	– Pneumonia-Aspiration
– Bone and Joint Infection	– Pneumonia-Community Acquired
– C diff (Clostridium difficile) Infection	– Pneumonia-Healthcare Associated
– Cardiovascular Infection	– Pneumonia-Hospital Acquired
– CNS Infection	– Prophylaxis-Non-Surgical
– COPD Exacerbation	– Prophylaxis-Surgical
– Empiric (UNKNOWN Source)	– Sepsis
– GI/Intra-abdominal Infection	– Skin & Soft Tissue Infection
– GYN/OB Infection or Prophylaxis	– UTI-Cystitis
– Neutropenic Fever	– UTI-Pyelonephritis/Complicated
– Oral/ENT Infection	– Other

HCA Indication Screens: Empiric or Sepsis Indications

- If "Empiric Unknown" or "Sepsis" are selected:

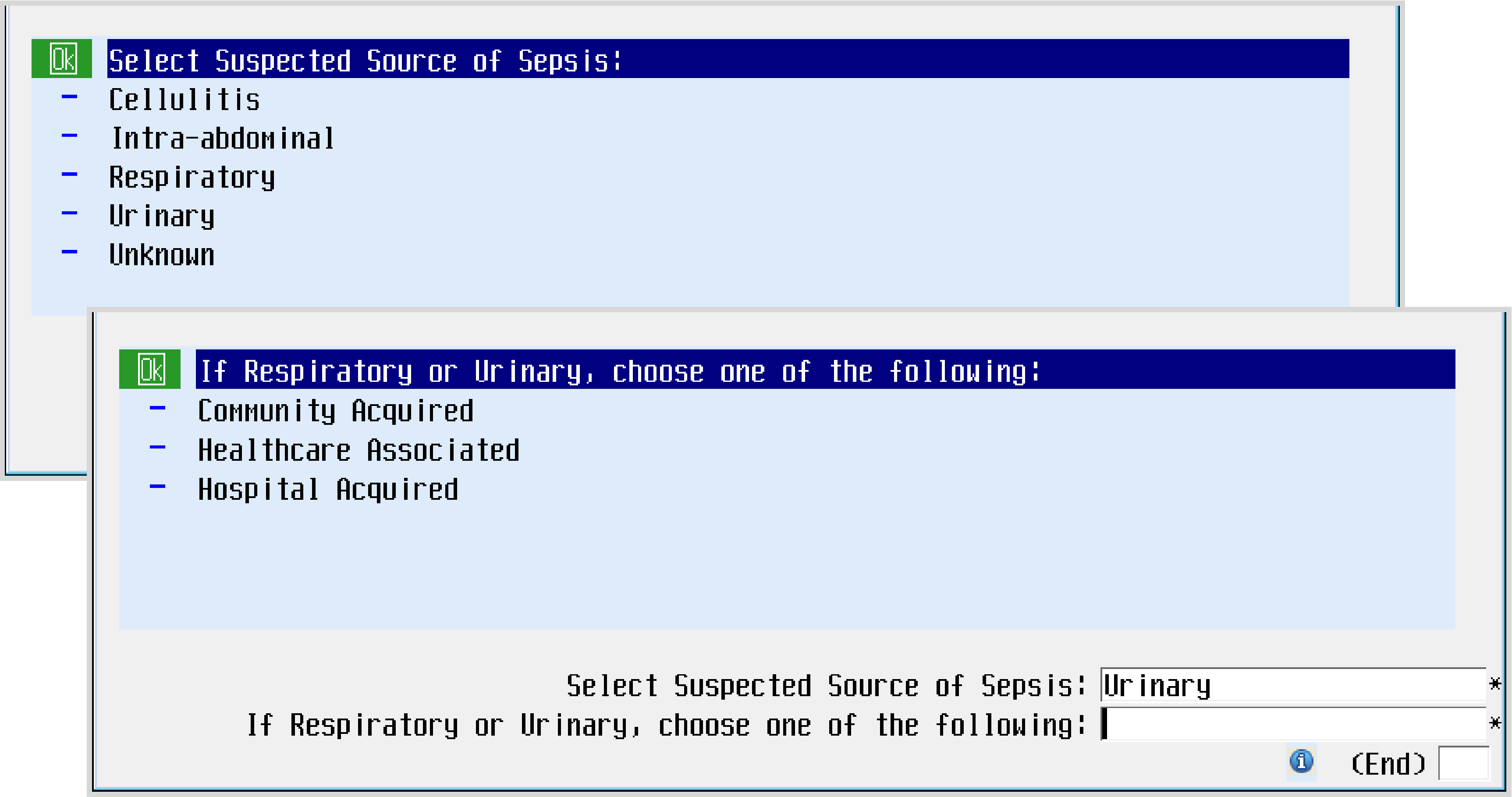

HCA Indication Screens: Empiric or Sepsis Indications

- If “Pneumonia-Aspiration” or “UTI” are selected:

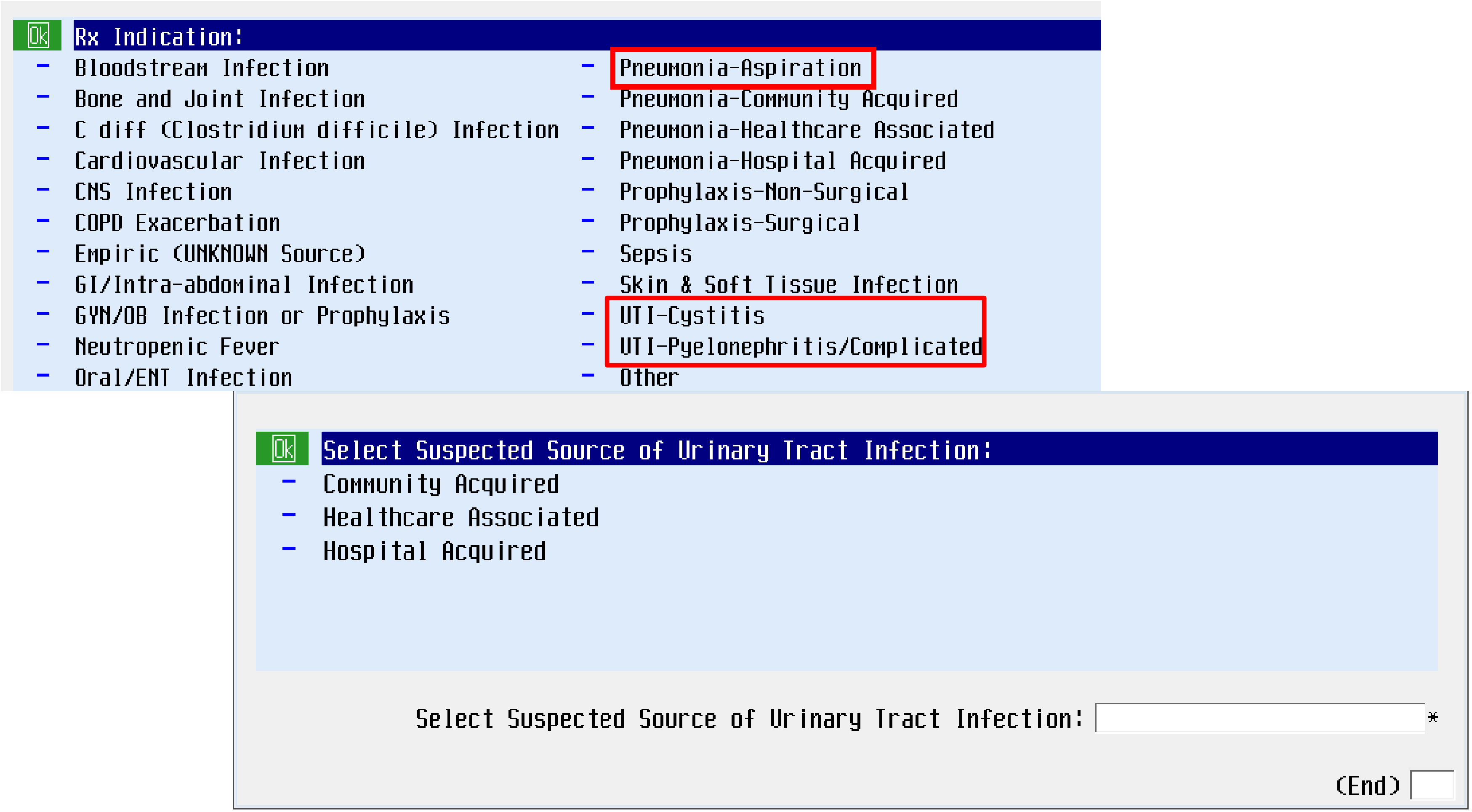

INSPIRE Workflow: ED Patients Going to ICU

- **For ED patients**, if an INSPIRE indication is chosen, then asks if patient is likely to go to the ICU
  - If YES → no further screens
  - If NO → INSPIRE CPOE screens continue (Arm 2 only)
- Will be live in **both Arm 1 and Arm 2**

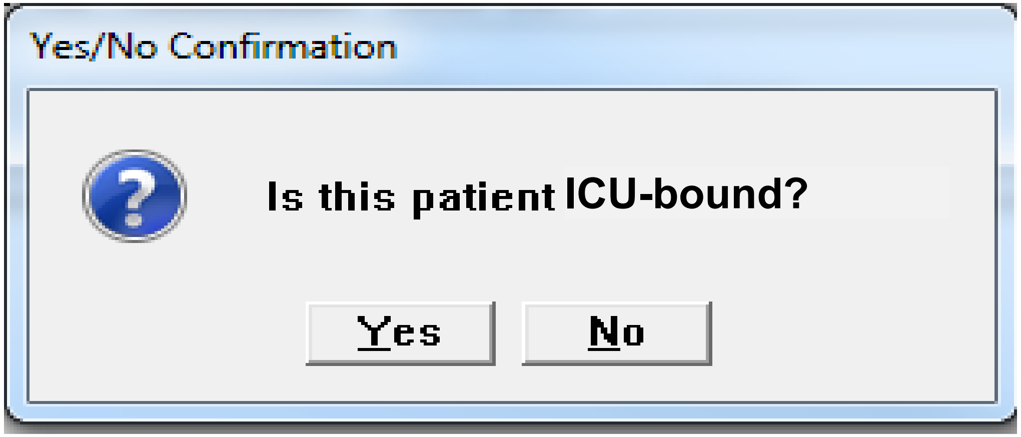

Study Champion Priorities - Arm 1

- Ensure HCA Meaningful Use screen activation in ED and inpatient areas
- Assess facility needs for successful launch
- Inform and educate clinicians locally
- Identify ED or hospitalist champions and other leaders to help physicians get on board

Integrating Smart Prompt into Existing Order Sets

- Smart prompts designed to integrate into existing order sets
- HCA pharmacy IT team can help troubleshoot
- Study champions should assess for redundancy in physician workflow
- Strategies for successful integration if conflicts found:
  - Separate ICU order sets from non-ICU
  - Indications can be pre-populated if using order set

Clinician Outreach Planned

- Webinars
  - Audience: hospitalists, ED physicians, nurse practitioners, physician assistants, pharmacists – any prescribers
  - At least two separate dates/time slots will be offered
- Clinician email draft
  - INSPIRE team will reach out to you with a draft email for you to send out to clinicians
- HCA meaningful use indication screen workflow summary

Will the Indication Screens Impact ED Core Measures?

- Indication Screens **do not** affect "time-to-antibiotics"
  - Does not impact whether antibiotics should be used
  - Supports decision for extended vs standard spectrum antibiotic

- Time-to-antibiotics metric definition concern from the field has been brought to leadership

Ongoing Reminder: Competing Interventions

- Facilities are fully expected to continue stewardship activities
- Examples of activities directly impacting primary outcomes:
  - New empiric antibiotic selection strategy
  - New de-escalation initiative in the ED or empiric period
  - New rapid diagnostic tool for pneumonia or UTI

Email HCAINSPIRE@gmail.com if considering initiatives/new interventions → steering committee can help to evaluate if activity could impact trial outcomes

Arm 1 - Key Role in INSPIRE-ASP

Your **key role** is to *maintain* U.S. gold standard for antibiotic stewardship practices against which our intervention will be compared. Avoid changing current practices in the empiric period. Avoid new initiatives specifically targeting empiric antibiotic use.

- Implement HCA indication screens
- Maintain best practices for stewardship
- Continue all current stewardship practices
- Inform INSPIRE-ASP team if considering new initiatives HCAINSPIRE@gmail.com → steering committee can help to evaluate if activity could impact trial outcomes

Arm 2:
INSPIRE Stewardship

INSPIRE-ASP Pneumonia Trial
and
INSPIRE-ASP UTI Trial

Protocol & Toolkit Binder

Funded by the Centers for Disease Control and Prevention (CDC)

INtelligent Stewardship Prompts to Improve Real-time Empiric Antibiotic Selection for Patients

Arm 2 – Smart Prompt

Toolkit Binder
Table of Contents

Document	Tab
Welcome & Summary of Goals …………………………………………….	Inside Front Cover
Study Investigators……………………………………………………………..	Inside Front Cover
Project Summary…………………………………………………………………………………………	1
Phone Matrix……………………………………………………………………………………………….	2
Roles & Responsibilities…………………………………………………………………………….	3
Antibiotic Selection Protocol……………………………………………………………………….	4
Study Champion Materials………………………………………………………………………….	5
Clinician Education Materials……………………………………………………………………..	6
CPOE Clinician Work Flow ………………………………………………………………………..	7
Clinician Quarterly Progress Report Example ……………………………………………….	8
Clinician Poster……………………………………………………………………..	Inside Back Cover

HCA

Funded by the Centers for Disease Control and Prevention (CDC)

Welcome

Welcome to the **INSPIRE-ASP** (**IN**telligent **S**tewardship **P**rompts to **I**mprove **R**eal-time **E**mpiric **A**ntibiotic **S**election for **P**atients) Trials. The INSPIRE-ASP Pneumonia and INSPIRE-ASP Urinary Tract Infection (UTI) Trials are separate 2-Arm cluster-randomized trials of 59 HCA Healthcare hospitals to assess best practice in antibiotic stewardship. These trials are a partnership between HCA Healthcare and academic investigators who are federally funded by the Centers for Disease Control and Prevention (CDC).

Summary of Goals

Up to 40% of patients who are prescribed antibiotics in U.S. hospitals either do not require these drugs or receive inappropriate antibiotics for the condition being treated. Moreover, inappropriate antibiotics are often broad-spectrum, empirically prescribed due to concerns about infection with multidrug-resistant organisms (MDROs), despite the low risk of MDRO infection in the vast majority of clinical settings. This is the case for patients admitted with pneumonia and urinary tract infections (UTIs), two of the most common conditions for antibiotic use. This trial will compare two strategies to determine best practices for empiric antibiotic selection. Arm 1, Routine Care, will continue antibiotic stewardship practices according to current facility strategies. Arm 2, Smart Prompt Alert Intervention, will implement a precision medicine smart prompt using a computerized physician order entry (CPOE) alert that provides clinicians with a patient’s probability of MDRO infection and recommends a standard-spectrum antibiotic choice in patients admitted for pneumonia or UTIs in non-critical care units. This trial will provide essential information to clinicians in real-time to support and optimize standard-spectrum empiric antibiotic use.

HCA

Funded by the Centers for Disease Control and Prevention (CDC)

Study Investigators and Stakeholders

University of California Irvine

Shruti Gohil, MD MPH, Lead Investigator

Susan Huang, MD MPH, Senior Investigator

Harvard Medical School/Harvard Pilgrim Health Care Institute

Ed Septimus, MD FACP FIDSA FSHEA

Meghan Baker, MD PhD

Richard Platt, MD MSc

Brigham & Women's Hospital

David Kubiak, Pharm D

University of Massachusetts Amherst

Ken Kleinman, ScD

Rush University

Robert Weinstein, MD

Mary Hayden, MD

Centers for Disease Control and Prevention

Sujan Reddy, MD MSc

Arjun Srinivasan, MD FSHEA

John Jernigan, MD MS

Lauri Hicks, DO

Melinda Neuhauser, Pharm D MPH FCCP FASHP

HCA Healthcare

Mandelin Cooper, Pharm D BCPS, Director, Clinical Pharmacy

Hayley Burgess, Pharm D, AVP Clinical Pharmacy Services

Risa Rahm, Pharm D CPHIMS, Director, Medication Management & Clin. Pharmacy Informatics

Will Cooper, Pharm D CPHIMS, Clinical Pharmacy Manager

Caren Spencer-Smith, Research Clinical Data Analyst, Patient Safety and Research

Julia Moody, MS SM (ASCP), Clinical Director, Infection Prevention

Russell Poland, PhD, AVP, Research and Scientific Communications

Karla Miller, Pharm D, VP Pharmacy Services

Ken Sands, MD MPH, VP Chief Epidemiologist and Patient Safety Officer

Jonathan Perlin, MD PhD, Chief Medical Officer

Funded by the Centers for Disease Control and Prevention (CDC)

INSPIRE-ASP Pneumonia and UTI Trials

***IN**telligent **S**tewardship **P**rompts to **I**mprove **R**eal-time **E**mpiric **A**ntibiotic **S**election for **P**atients*

The **INSPIRE-ASP Trials** are a **two-in-one** set of cluster-randomized trials at HCA Healthcare (HCA) hospitals to assess best practice in antibiotic stewardship for patients admitted to non-critical care units for pneumonia (PNA) and urinary tract infection (UTI). It tests the use of smart prompts to optimize prescribing and improve patient outcomes. Participating hospitals will be randomized to:

- **Arm 1 – Routine Care**
- **Arm 2 – Smart Prompt**

  Precision medicine smart prompt using a computerized physician order entry (CPOE) alert that gives a ***patient-specific risk estimate of antibiotic resistant infection*** and recommends standard-spectrum antibiotics in patients admitted for PNA or UTI who are found to have low risk (<10%) for drug resistant infection

Rationale

- Most non-ICU adult patients hospitalized with pneumonia or UTI are low risk for multidrug-resistant organisms (MDROs) and do not need broad-spectrum antibiotics.
- Identifying patients at low vs high risk of MDROs can decrease use of extended-spectrum antibiotics, reduce selection of resistant bacteria, and reduce *C. difficile* infections.

CPOE Details

- MDRO risk estimates are based upon patient-specific risk factors and local antibiograms
- We assessed demographics, comorbidities, MDRO history, prior antibiotic use, prior nursing home stay, and a variety of laboratory values to create a prediction algorithm
- Antibiotic recommendations are based on MDRO risk estimates and approved by trial steering committee, HCA leadership, and CDC experts.
- Recommendations support proper antibiotic choice but do not replace clinical judgment

Outcomes

- Primary: Empiric use of extended-spectrum antibacterials (first 3 days of admission)
- Secondary: Empiric use of (a) vancomycin and (b) antipseudomonal antibacterials
- Safety: Antibacterial escalation, ICU transfer, and length-of-stay
- Outcomes for other manuscripts: *C. difficile* infection, MDRO prevalence, cost-analysis

Anticipated Timeline

- 6 Month Phase In: October 2018 – March 2019
- 15 Month Intervention: April 2019 – June 2020

QUESTIONS? Study Investigators & Coordinators
HCAINSPIRE@gmail.com
844-522-4677

HCA

Funded by the Centers for Disease Control and Prevention (CDC)

Stewardship Champion Roles and Responsibilities

Pharmacy & Physician Stewardship Champions will partner with the INSPIRE-ASP investigative team and are expected to:

1. Join trial coaching calls
2. Actively engage and educate clinicians on value and safety of the INSPIRE-ASP Trials
3. Review clinician quarterly progress reports and encourage improvements
4. Address clinician outliers in a supportive, knowledgeable way
5. Set reachable local targets and improvement goals
6. Report interventions or activities that may impact trial outcomes

Funded by the Centers for Disease Control and Prevention (CDC)

INSPIRE-ASP Empiric Antibiotic Selection Protocol

The **INSPIRE-ASP Pneumonia and Urinary Tract Infection (UTI) trials** use a computerized physician order entry (CPOE) alert to provide clinicians with (1) patient-specific risk estimates for infection due to an antibiotic-resistant bacteria and (2) recommendations on the standard-spectrum empiric antibiotic selection based on individualized risk estimates. This intervention will improve the use of standard-spectrum antibiotics for non-ICU patients at low risk of having pneumonia or UTI due to an antibiotic-resistant bacteria.

This document provides pharmacists and clinicians with an overview of the protocol by which antibiotic selection should occur when INSPIRE-ASP CPOE alerts are activated at your facility.

Protocol for Empiric Antibiotic Selection

1. **The CPOE alert is activated when:**
   a. Clinicians order an extended-spectrum antibiotic(s) for pneumonia or UTI in non-ICU patients within the first 3 days of admission, including in the emergency department.
   b. If standard-spectrum antibiotics are ordered (e.g. ceftriaxone or ceftriaxone/azithromycin), no prompt will be triggered.
2. **The CPOE alert provides clinicians with patient-specific risk estimates for antibiotic-resistant infection.**
   a. Risk estimates are calculated for the antibiotic-resistant organism(s) targeted by the antibiotic ordered (Table 1).
      i. Example 1: When a clinicians orders vancomycin for pneumonia, the CPOE alert will calculate the risk that the patient has pneumonia due to MRSA.
      ii. Example 2: When a clinicians orders cefepime for pneumonia, the CPOE alert will calculate the risk that the patient has pneumonia due to *Pseudomonas*.
      iii. Example 3: When a clinicians orders linezolid for UTI, the CPOE alert will calculate the risk that the patient has UTI due to MRSA or VRE.
      iv. Example 4: When a clinicians orders meropenem for UTI, the CPOE alert will calculate the risk that the patient has UTI due to ESBL or other resistant gram negative bacteria.
3. **Clinicians will be provided recommendations on the appropriateness of extended-spectrum antibiotics based upon the patient's calculated risk.**
   a. If the patient's risk for antibiotic resistant pneumonia or UTI is <10%, then the risk is considered low and the CPOE alert will recommend ceftriaxone as the standard empiric antibiotic.

   b. If the patient's risk for MDRO pneumonia or UTI is ≥10%, the prompt will automatically allow completion of the extended-spectrum antibiotic order without any prompts.
   c. CPOE recommendations are **not intended to supplant clinician judgment**. Patients who are neutropenic, decompensating, or otherwise at high risk for complications or drug resistant infections should be treated as clinically appropriate.
   d. Clinicians will always have the option to override the CPOE alert recommendation after indicating the reason for the override.

4. **Prescriber acceptance with CPOE alert recommendations will be monitored by the stewardship team/study champions.**
   a. Clinician Feedback reports, including the number and reason for CPOE recommendation overrides, will be provided to study champions.
   b. Study champions will review acceptance and provide prescribers with written and/or verbal feedback.

Table 1: Extended-Spectrum Antibiotics and Pathogen Included in the CPOE Alert

Antibiotic Ordered	Pathogen Included in Risk Estimate	
	PNEUMONIA	UTI
Daptomycin	Not Recommended for PNA	MRSA, VRE
Linezolid[1]	MRSA	
Vancomycin (IV only)	MRSA	
Ceftaroline		
Aztreonam	*Pseudomonas*	
Cefepime		
Ceftazidime		
Piperacillin/Tazobactam		
Ertapenem	ESBL, GNR Resistant to cephalosporins and penicillins	
Doripenem	ESBL, GNR+*Pseudomonas* Resistant to cephalosporins and penicillins	
Imipenem		
Meropenem		
Ceftolozane/Tazobactam		
Ceftazidime/Avibactam	No Screen - Requires ID Approval[2]	
Tigecycline		
Colistin		
Polymyxin B		

1. Both oral and intravenous formulations of drugs in this category are included.
2. For anti-CRE medications, all models show <10% patient risk for having a highly drug resistant pathogen warranting the use of these drugs. Therefore, patient specific risk estimate will not be calculated and a static CPOE screen will be developed that will recommend avoiding empiric use without consultation with antibiotic stewardship team or infectious diseases.
3. Newly released ES medications require ID approval and will be considered for inclusion in the CPOE prompts on a case by case basis
Note: INSPIRE-ASP smart prompt intervention excludes the following routes of administration: inhaled, otic, ophthalmic, or irrigation.
Abbreviations:

MRSA - Methicillin Resistant *Staphylococcus aureus*
VRE – Vancomycin Resistant *Enterococci*
MDR- Multi-Drug Resistant
ESBL – Extended Spectrum Beta Lactamase producer
CRE – Carbapenem Resistant *Enterobacteriaceae*
GNR – Gram Negative Rod

HCA
Funded by the Centers for Disease Control and Prevention (CDC)

INSPIRE-ASP Trials: Arm 2

Study Champion Materials

This section includes:

- Study Champion Checklist
- Abbreviated FAQs
- Study Champion FAQs
- Verbal Order Checklist

INSPIRE-ASP Study Champion Intervention Checklist

As your facility's Study Champion for INSPIRE-ASP, there are a few key things you can do to ensure the CPOE Smart Prompt launch goes smoothly. Here is a quick checklist:

☐ Assess existing order sets

- ✓ Review any sepsis, pneumonia, or UTI order sets currently in place
- ✓ Evaluate for potential conflicts or redundancies with CPOE smart prompts
- ✓ Work with local and corporate IT to resolve
- ✓ Examples include:
  1. Sepsis order set contains only extended-spectrum antibiotics as options for pneumonia. CPOE smart prompt alert will recommend ceftriaxone when MDRO risk is low. This may be seen by clinicians as potentially conflicting recommendations.

     Possible solution(s): (a) Add ceftriaxone to list of acceptable antibiotics in sepsis order sets. (b) Make two separate order sets, one for ICU patients and one for non-ICU patients.

☐ Identify key leaders

- ✓ Hospitalist leader, ED leader, Pharmacy staff
- ✓ Enlist their help to educate their peers, give presentations, and help you disseminate education and field questions from clinicians

☐ Get the word out and educate frontline clinicians/prescribers

- ✓ Present INSPIRE at medical staff/faculty meetings
- ✓ Post flyers/posters in physician lounges
- ✓ "Meet-and-greet" clinicians on the floors with the one-page INSPIRE summary
- ✓ Disseminate emails to all clinicians
- ✓ Encourage clinicians to attend study webinars or access information on Atlas
- ✓ Educate nurses or pharmacists that may be taking verbal orders

☐ Once launched, check if the CPOE smart prompt is working as planned

- ✓ The prompt fires only for extended-spectrum antibiotics
- ✓ The prompt does <u>not</u> fire in the ICU or after hospital day 3
- ✓ Clinicians are not choosing indications other than Pneumonia or UTI to circumvent the smart prompt
- ✓ Verbal orders are placed in Meditech/Order Management, NOT by paper or the "PHA" system

INSPIRE-ASP Abbreviated FAQ
For Clinician Prescribers and Pharmacists

What are the INSPIRE-ASP Trials?

The INSPIRE-ASP (**IN**telligent **S**tewardship **P**rompts to **I**mprove **R**eal-time **E**mpiric **A**ntibiotic **S**election for **P**atients) Trials are two separate cluster-randomized trials of HCA Healthcare (HCA) hospitals to test an antibiotic stewardship intervention for pneumonia and urinary tract infection (UTI).

- **Arm 1** – **Routine Care**
- **Arm 2** – **CPOE Smart Prompt Alert Intervention**
    1. Provides patient-specific risk estimate for pneumonia or UTI due to a multi-drug resistant organism (MDRO) when an antibiotic is ordered for these indications
    2. Recommends standard-spectrum antibiotics in non-ICU pneumonia or UTI patients with low (<10%) MDRO risk

When will the CPOE smart prompt fire?

- Prompts fire when extended-spectrum antibiotics are ordered within 3 days of admission
- Only for adults in ED or **non**-intensive care units (ICU) admitted for pneumonia or UTI

Which antibiotics are considered extended-spectrum?

Category	Extended-Spectrum Antibiotics for INSPIRE CPOE Alerts
**Anti-MRSA**	Ceftaroline, Daptomycin, Linezolid[1], Vancomycin[2]
**Anti-VRE**	Daptomycin, Linezolid[1]
**Anti-Pseudomonas**	Aztreonam, Cefepime, Ceftazidime, Piperacillin/Tazobactam
**Anti-ESBL**	Ertapenem, Doripenem, Imipenem, Meropenem, Ceftolozane/Tazobactam
**Anti-CRE**	Ceftazidime/Avibactam, Colistin, Polymixin B, Tigecycline

[1]**Both oral and intravenous (IV) formulations**
[2]**IV formulation only**

How is the CPOE prompt "smart"?

The CPOE smart prompt uses your specific patient medical record data to calculate MDRO risk. Risk estimate are tailored to the antibiotic selected. For example:

- If ceftriaxone ordered → no prompt fires at all (standard-spectrum antibiotic)
- If vancomycin ordered → returns patient's MRSA risk
- If cefepime or piperacillin/tazobactam ordered → returns *Pseudomonas* risk
- If linezolid ordered → returns MRSA and VRE risk
- If meropenem ordered → returns ESBL + resistant gram negative bacteria
- Recommends ceftriaxone when MDRO risk is low (<10%)

What are the CPOE risk estimates based on?

The INSPIRE study team assessed over 536,000 patients admitted to HCA hospitals over 3 years (May, 2014- March, 2017). We evaluated **(1) your hospital antibiogram** for pneumonia and UTI patients and **(2) patient risk factors of MDRO or *Pseudomonas* infection**, including demographics (age, gender, race, ethnicity, insurance status), comorbid conditions, history of MDRO, history of prior HCA hospital, ED, or nursing home visit, prior antibiotics, and lab values.

Using a special statistical method, we identified which risk factors were most often found in patients who developed pneumonia or UTI with an MDRO or *Pseudomonas*. Using these risk factors (which are different for every MDRO), the CPOE smart prompt gives the patient's **absolute risk** for infection with the MDRO(s) targeted by the antibiotic ordered.

How far back does the CPOE smart prompt look for MDRO history?

The CPOE smart prompt mines your facility's microbiology data in Meditech as far back as 2012. It will capture a patient's MDRO history even if there is no electronic MDRO flag, but it will not capture MDRO history or cultures from an outside hospital.

How quickly can the CPOE smart prompt account for positive cultures? What if a culture was sent recently?

The CPOE smart prompt captures cultures resulted in Meditech at your facility 7 or more days ago; it currently cannot integrate new cultures resulted in the empiric period. For example, if a culture was sent upon admission, this will not be included in a risk estimate when ordering extended-spectrum antibiotics on hospital day 3. We encourage close monitoring of all new microbiology results as they become available and selection of antibiotics accordingly.

How does the CPOE smart prompt exclude ICU patients?

1. Does not fire if a patient is located in the ICU.
2. For emergency department, smart prompt asks clinicians if the patient is ICU-bound and does not fire if clinician responds "yes".
3. If smart prompt fires and patient is going to the ICU, clinicians can override the prompt.

Will INSPIRE impact ED sepsis core measures?

The CPOE smart prompt **does NOT** affect "time to antibiotics" because it does not impact whether antibiotics should be used but rather gives real-time information to help decide whether extended-spectrum or standard-spectrum antibiotics should be used. The recommended standard-spectrum antibiotic, ceftriaxone, is compliant with core measures.

Will patients need to give informed consent?

No. This study has been approved as a minimal risk study and will be implemented as a quality improvement (QI) protocol through the existing infrastructure of your hospital's antibiotic stewardship programs. The assigned protocol will be approved through your usual clinical/leadership committees and become hospital guidance on appropriate empiric antibiotic use.

Whom can I contact for more information?

INSPIRE-ASP Study Team:
HCAINSPIRE@gmail.com, (844)-522-4677

Frequently Asked Questions (FAQ)
Pharmacy & Clinician Study Champions

This FAQ specifically addresses questions that facility Study Champions may have. Please see the "Clinician Prescriber" FAQ for key details on the intervention.

What are the INSPIRE-ASP Trials?

The INSPIRE-ASP (**IN**telligent **S**tewardship **P**rompts to **I**mprove **R**eal-time **E**mpiric **A**ntibiotic **S**election for **P**atients) Trials are two separate cluster-randomized trials of HCA Healthcare (HCA) hospitals to assess best practice in antibiotic stewardship for non-ICU patients admitted with pneumonia and urinary tract infection (UTI). Hospitals will be randomized to:

- **Arm 1** – **Routine Care**
- **Arm 2 – CPOE Smart Prompt Alert Intervention**
  Precision medicine smart prompt using a computerized physician order entry (CPOE) alert that supports antibiotic prescribing with:
  1. Patient-specific estimate of risk for pneumonia or UTI due to a multi-drug resistant organism (MDRO) or *Pseudomonas*
  2. Recommendations for standard spectrum antibiotics in non-ICU patients admitted for pneumonia or UTI with low MDRO risk (<10%)

What are the outcomes of the INSPIRE-ASP Trials?

The goal is to identify a best practice strategy for choosing empiric (within 3 calendar days) antibiotics based on a patient's risk for MDRO infection. Outcomes include:

- Primary: Empiric use of extended-spectrum antibacterials (first 3 days of admission)
- Secondary: Empiric use of (a) vancomycin and (b) antipseudomonal antibacterials
- Safety: Antibacterial escalation, ICU transfer, and length-of-stay
- Outcomes for other manuscripts: *C. difficile* infection, MDRO prevalence, cost-analysis

Which patients are included?

Only adults admitted to a non-ICU ward with pneumonia or UTI who are ordered to receive an extended-spectrum antibiotic within 3 days of admission will be included in the smart prompt alerts. The INSPIRE smart prompt alerts are programmed to not fire for patients younger than 18 years or who are in an ICU location**.** Patients admitted from jail or prison are **not** included in this study.

Is INSPIRE-ASP one trial or two?

The INSPIRE-ASP trials will be rolled out together but are actually two separate trials: the **INSPIRE-ASP Pneumonia Trial** and **INSPIRE-ASP UTI Trial**. Because there are important differences between the two infection syndromes, our smart prompt will need to provide separate risk estimates for pneumonia and UTI. All analyses will need to be done separately to properly evaluate the impact of our intervention. However, all other aspects of the CPOE smart prompt intervention will be rolled out together.

Why will the study focus on Pneumonia and UTI?

Pneumonia and UTI are the top two most common reasons for infection-related admission to the hospital and account for the majority of antibiotic use nationally.

Why is antibiotic stewardship a major contributor to patient safety?

Unnecessary extended-spectrum antibiotic use can increase a patient's risk for MDRO and *Clostridium difficile* infections (CDI). These infections are often harder to manage due to limited treatment options. Both CDI and MDRO infections are associated with higher risks for complication, longer length-of-stay, readmission, and death.

How would patient-specific alerts promote better, safer antibiotic use?

Uncertainty about the risk that an individual patient is infected with an MDRO drives overuse of extended-spectrum antibiotics. Current diagnostic tests do not provide immediate information about which pathogen is responsible for infection. This uncertainty drives clinicians to cover all possible organisms, including MRSA, *Pseudomonas* species, and ESBLs, even when a patient's risk for these MDROs may be very low. Providing clinicians with real-time estimates of a patient's risk for infection due to an MDRO would allow more informed antibiotic choices.

What are the patient specific risk estimates based on?

The INSPIRE study team assessed over 536,000 patients admitted at all HCA hospitals over 3 years (2014-2016) within Meditech. We evaluated (1) your hospital antibiogram for pneumonia and UTI patients and (2) patient risk factors of MDRO infection, including:

- Demographics (age, gender, race, ethnicity, insurance status)
- Comorbid conditions (e.g., diabetes, lung disease, renal failure)
- History of ED visit or admission to an HCA hospital
- History of nursing home admission
- History of antibiotics received during an ED or inpatient admission
- History of MDRO (screening or clinical cultures at your facility)
- Lab values (e.g., BUN, creatinine)

Using a special statistical method, we identified which risk factors were most often found in patients who developed pneumonia or UTI with an MDRO. Using these risk factors (which differ for every MDRO), the CPOE smart prompt gives the patient's **absolute risk** for infection with the MDRO(s) targeted by the antibiotic ordered.

Note that INSPIRE calculates your facility antibiogram as the proportion of MDRO or pseudomonas among patients admitted with PNA or UTI, which is different than the traditional way most microbiology labs calculate this.

For more detailed information, you can review an audio-enabled presentation on the HCA Atlas website, entitled "How the MDRO Risk Estimates Work": http://connect.medcity.net/web/hca-research-academic-affairs/inspire

How far back does the CPOE smart prompt look for MDRO history?

The CPOE smart prompt mines your facility's microbiology data in Meditech as far back as 2012. It will capture a patient's MDRO history even if there is no electronic MDRO flag, but it will not capture MDRO history or cultures from an outside hospital.

How quickly can the CPOE smart prompt account for positive cultures? What if a culture was sent recently?

The CPOE smart prompt captures cultures resulted in Meditech at your facility 7 or more days ago; it currently cannot integrate new cultures resulted in the empiric

period. For example, if a culture was sent upon admission, this will not be included in a risk estimate when ordering extended-spectrum antibiotics on hospital day 3. We encourage close monitoring of all new microbiology results as they become available and selection of antibiotics accordingly.

Will risk estimates be validated after the intervention starts?

Our risk estimates will be revalidated at regular intervals during the intervention. Significant changes in select variables (e.g., changes in your antibiogram) will be updated within the CPOE alert. Note that our estimates are based on 3 years of data over 140 HCA hospitals, it is unlikely that additional accrual of trial time will change risk factors that have already been identified.

What makes the INSPIRE CPOE prompt "smart"?

The INSPIRE smart prompts are automated, real-time alerts built into an electronic medical record that are programmed to return an **individual patient's specific risk** for MDRO or *Pseudomonas* when an extended-spectrum antibiotic is ordered. This risk estimate is tailored to each antibiotic selected. For example:

- If ceftriaxone ordered -→ no prompt (recommended)
- If vancomycin ordered → returns MRSA risk
- If cefepime or piperacillin/tazobactam ordered → returns *Pseudomonas* risk
- If linezolid ordered → returns MRSA and VRE risk
- If meropenem ordered → returns ESBL and other resistant gram negative bacteria risk

What about antibiotics administered through non-systemic routes, such as inhalation, irrigation, or topical?

The INSPIRE smart prompts are only triggered when an extended-spectrum antibiotic is ordered for pneumonia, UTI, or sepsis/empiric source due to respiratory or urinary source. This process would automatically exclude otic, ophthalmic, or topical antibiotics. Antibiotics administered through inhalation (e.g., ceftazidime) or irrigation (e.g., vancomycin use in bladder irrigation) are excluded from INSPIRE smart prompts.

What if someone takes a verbal order for antibiotics?

The CPOE smart prompt will **only fire in Meditech**. It will not fire in the "PHA" system. We ask all clinical staff who take verbal orders (including "vancomycin per pharmacy dosing" orders) to place them within Meditech. This means that you will have to ask clinicians (1) what infection the antibiotic is meant to treat and (2) whether the physician would agree to standard spectrum antibiotics if the risk is found to be low (<10%). Please see the "Verbal Order Checklist for Pharmacists and Nurses" that will help guide how verbal orders can be best handled.

What will be required of participating sites?

Study champions will be asked to:

- Join trial coaching calls
- Engage and educate clinicians on value and safety of the INSPIRE-ASP Trials
- Review clinician quarterly progress reports and encourage improvements
- Address clinician outliers in a supportive, knowledgeable way
- Set reachable local targets and improvement goals
- Report interventions or activities that may impact trial outcomes

Please reference the "Study Champion Intervention Checklist" of key things you can do to ensure the INSPIRE CPOE smart prompt launches successfully at your facility.

How do we avoid competing interventions and how are these defined?

Participating facilities are fully expected to continue their stewardship activities and initiatives but should avoid activities that may directly impact the trial's primary outcome. Such activities are anticipated to be rare.

The following are examples of activities that do **NOT** directly conflict with this study:

- Surveillance and monitoring of antibiotic use
- De-escalation strategies after empiric period has passed (after hospital day 4)
- Using Vigilanz for automatic dosing

If already in place during baseline (Summer 2016 - Fall 2018), the intervention is not considered in conflict.

Examples of interventions that may directly conflict with the trial include:

- A new stewardship strategy that aims to reduce the use of extended-spectrum antibiotics in the Emergency Department (ED) or within 3 days of admission.
- A new real-time antibiotic de-escalation strategy in the ED/within 3 calendar days
- A trial of a new rapid diagnostic tool for pneumonia or UTI that would be used in the empiric period to guide antibiotic therapy within 3 days of admission.
- Ordering antibiotics through Patient Keeper

If a new intervention is thought to be in direct conflict with the study outcomes, we would ask participating facilities to disclose these plans to the research team for further discussion.

Will CPOE acceptance be tracked?

Yes, the investigative team will partner with HCA to provide you with quarterly clinician and hospital progress reports on a monthly basis. This will contain a rolling quarter's worth of data on how often clinicians order extended-spectrum antibiotics for pneumonia or UTI, how often recommendations for standard antibiotics is accepted, and if overridden, what was chosen as the reason. All data will be collected via the centralized Clinical Data Warehouse and will be provided to you.

What will be the process for incorporating smart prompt alerts into the electronic medical record system?

Smart prompt alerts will be developed by the HCA corporate IT team, including programming and initial beta testing. Corporate IT will provide a template for local IT teams to implement in hospitals assigned to the intervention arm.

Will this intervention affect our formulary antibiotic choices?

No. This intervention, including the smart prompt alerts, will focus on guiding clinicians towards optimal antibiotic class choices, not specific drugs or manufacturers.

Will patients need to give informed consent?

No. This study has been approved as a minimal risk study and will be implemented as a quality improvement (QI) protocol through the existing infrastructure of your hospital's antibiotic stewardship programs. The assigned protocol will be approved

through your usual clinical/leadership committees and become hospital guidance on standard spectrum empiric antibiotic use.

Has this study been approved by the Institutional Review Board (IRB)?

Yes. Harvard Pilgrim's IRB will provide ethical oversight for these trials and serve as the central IRB. Both INSPIRE-ASP PNA and UTI trials have been approved by the Harvard IRB. Your hospital has submitted all ceding and reliance agreements necessary to grant oversight by the central IRB at Harvard. HCA's corporate compliance and regulatory affairs will help provide support for this endeavor.

What is the timeline?

- In-person site visits – Summer-Fall 2018
- IT CPOE smart prompt alert implementation – Fall 2018
- Intervention launch – October 1, 2018
- Phase-in – 6 months
- Intervention – 15 months

Who comprises the study team?

These trials are a collaborative between HCA Healthcare and academic partners at the University of California Irvine, Harvard Pilgrim Health Care/Harvard Medical School, and Rush University. The trials are funded through a contract with Centers for Disease Control and Prevention (CDC).

Whom can I contact for more information?

INSPIRE-ASP Study Team
Lead Investigator – Shruti Gohil, MD, MPH, UC Irvine
HCAINSPIRE@gmail.com, (844)-522-4677

Edward Septimus, MD, FACP, Senior Lecturer HPHCI
eseptimus@gmail.com, (281) 714-5689

Hayley Burgess, PharmD, AVP Clinical Pharmacy Services, HCA
Hayley.Burgess@hcahealthcare.com, (615) 344-1857

Verbal Order Checklist for Pharmacists and Nurses

Background

The INSPIRE-ASP CPOE smart prompt intervention will encourage compliance with national guidance for standard-spectrum antibiotics (e.g., ceftriaxone) for pneumonia and UTI. Ordering extended-spectrum antibiotics (e.g., vancomycin, cefepime) for pneumonia or UTI in the ED or non-ICU wards within 3 days of admission will activate a computerized physician order entry (CPOE) smart prompt alert that will use the patient's Meditech data to provide:

1. Patient's risk for pneumonia or UTI due to antibiotic resistant bacteria
2. Recommendation for standard-spectrum antibiotics when risk is low (<10%)

Taking Verbal Orders for Inpatient Extended-Spectrum Antibiotics

The INSPIRE-ASP CPOE prompts apply **ONLY to extended-spectrum antibiotics** ordered on **non-ICU patients** hospitalized for **pneumonia or UTI** within **3 days of admission**.

- ➢ The prompt does **NOT** fire for standard-spectrum antibiotics.
- ➢ A list of extended-spectrum antibiotics is provided in Table 1 on the following page.

When taking verbal orders for extended-spectrum antibiotics, we generally recommend that you stay on the phone with the ordering clinician while entering orders. If you are unable to place the order directly at the time of verbal order, use the following questions to get key information you will need when placing the order:

1. **Check if patient has been <u>hospitalized for 4 days</u> or more or is <u>in the ICU</u>? If yes to either →** *no further physician questions needed. CPOE will **NOT** fire.*
2. **Is patient in the Emergency Department (ED)?**
   a. **If in ED →** ask if patient is ICU-bound
      i. **If ICU-bound →** no further questions
      ii. If <u>NOT</u> ICU-bound → proceed to Step 3

3. **IF extended-spectrum antibiotic being ordered within 3 days of admission:**
   a. **Ask prescriber what indication/infection the antibiotic is being used to treat (**Remind prescriber that choosing an indication is part of HCA's meaningful use stewardship initiative).
   b. If prescriber chooses any of the following indications, **proceed to step 4**:
      - Pneumonia
      - UTI
      - Sepsis – due to PNA or UTI
      - Empiric (unknown) source – respiratory or urinary source
4. **Remind prescriber that extended-spectrum antibiotics in this patient will trigger the INSPIRE smart prompt and will give the patient's specific risk for infection due to MDRO or *Pseudomonas*. The following suggested script can be used or modified as needed:**
   a. "Just a reminder, Dr. ___, our hospital has an antibiotic protocol for pneumonia and UTI. The computer will calculate this patient's risk of an antibiotic-resistant pneumonia/UTI. If the risk is low (<10%) then ceftriaxone will be recommended. Should I plan to follow this recommendation?"
      i. **If yes** → no further questions.
      ii. **If no** → ask the prescriber what the reason for overriding the recommendation would be:
         - Allergy
         - Neutropenia
         - Positive MDRO culture this admission or outside hospital
         - Patient will go to the ICU
         - Other (ask prescriber to specify)

Table 1: Extended-Spectrum Antibiotics That Will Trigger INSPIRE-ASP CPOE Alerts

Category	Extended-Spectrum Antibiotics for INSPIRE CPOE Alerts
**Anti-MRSA**	Ceftaroline, Daptomycin, Linezolid[1], Vancomycin[2]
**Anti-VRE**	Daptomycin, Linezolid[1]
**Anti-Pseudomonas**	Aztreonam, Cefepime, Ceftazidime, Piperacillin/Tazobactam
**Anti-ESBL**	Ertapenem, Doripenem, Imipenem, Meropenem, Ceftolozane/Tazobactam
**Anti-CRE**	Ceftazidime/Avibactam, Colistin, Polymixin B, Tigecycline

[1]Both oral and intravenous (IV) formulations
[2]IV formulation only

INSPIRE-ASP Trials: Arm 2

Clinician Education Materials

This section includes:

- Abbreviated FAQs
- Clinician FAQs
- Essential Clinician Facts
- Do’s and Don’ts
- Clinician Education Presentation
- Clinician Poster

INSPIRE-ASP Abbreviated FAQ
For Clinician Prescribers and Pharmacists

What are the INSPIRE-ASP Trials?

The INSPIRE-ASP (**IN**telligent **S**tewardship **P**rompts to **I**mprove **R**eal-time **E**mpiric **A**ntibiotic **S**election for **P**atients) Trials are two separate cluster-randomized trials of HCA Healthcare (HCA) hospitals to test an antibiotic stewardship intervention for pneumonia and urinary tract infection (UTI).

- **Arm 1** – **Routine Care**
- **Arm 2** – **CPOE Smart Prompt Alert Intervention**
    1. Provides patient-specific risk estimate for pneumonia or UTI due to a multi-drug resistant organism (MDRO) when an antibiotic is ordered for these indications
    2. Recommends standard-spectrum antibiotics in non-ICU pneumonia or UTI patients with low (<10%) MDRO risk

When will the CPOE smart prompt fire?

- Prompts fire when extended-spectrum antibiotics are ordered within 3 days of admission
- Only for adults in ED or **non**-intensive care units (ICU) admitted for pneumonia or UTI

Which antibiotics are considered extended-spectrum?

Category	Extended-Spectrum Antibiotics for INSPIRE CPOE Alerts
**Anti-MRSA**	Ceftaroline, Daptomycin, Linezolid[1], Vancomycin[2]
**Anti-VRE**	Daptomycin, Linezolid[1]
**Anti-Pseudomonas**	Aztreonam, Cefepime, Ceftazidime, Piperacillin/Tazobactam
**Anti-ESBL**	Ertapenem, Doripenem, Imipenem, Meropenem, Ceftolozane/Tazobactam
**Anti-CRE**	Ceftazidime/Avibactam, Colistin, Polymixin B, Tigecycline

[1]**Both oral and intravenous (IV) formulations**
[2]**IV formulation only**

How is the CPOE prompt "smart"?

The CPOE smart prompt uses your specific patient medical record data to calculate MDRO risk. Risk estimate are tailored to the antibiotic selected. For example:

- If ceftriaxone ordered → no prompt fires at all (standard-spectrum antibiotic)
- If vancomycin ordered → returns patient's MRSA risk
- If cefepime or piperacillin/tazobactam ordered → returns *Pseudomonas* risk
- If linezolid ordered → returns MRSA and VRE risk
- If meropenem ordered → returns ESBL + resistant gram negative bacteria
- Recommends ceftriaxone when MDRO risk is low (<10%)

What are the CPOE risk estimates based on?

The INSPIRE study team assessed over 536,000 patients admitted to HCA hospitals over 3 years (May, 2014- March, 2017). We evaluated **(1) your hospital antibiogram** for pneumonia and UTI patients and **(2) patient risk factors of MDRO or *Pseudomonas* infection**, including demographics (age, gender, race, ethnicity, insurance status), comorbid conditions, history of MDRO, history of prior HCA hospital, ED, or nursing home visit, prior antibiotics, and lab values.

Using a special statistical method, we identified which risk factors were most often found in patients who developed pneumonia or UTI with an MDRO or *Pseudomonas*. Using these risk factors (which are different for every MDRO), the CPOE smart prompt gives the patient's **absolute risk** for infection with the MDRO(s) targeted by the antibiotic ordered.

How far back does the CPOE smart prompt look for MDRO history?

The CPOE smart prompt mines your facility's microbiology data in Meditech as far back as 2012. It will capture a patient's MDRO history even if there is no electronic MDRO flag, but it will not capture MDRO history or cultures from an outside hospital.

How quickly can the CPOE smart prompt account for positive cultures? What if a culture was sent recently?

The CPOE smart prompt captures cultures resulted in Meditech at your facility 7 or more days ago; it currently cannot integrate new cultures resulted in the empiric period. For example, if a culture was sent upon admission, this will not be included in a risk estimate when ordering extended-spectrum antibiotics on hospital day 3. We encourage close monitoring of all new microbiology results as they become available and selection of antibiotics accordingly.

How does the CPOE smart prompt exclude ICU patients?

1. Does not fire if a patient is located in the ICU.
2. For emergency department, smart prompt asks clinicians if the patient is ICU-bound and does not fire if clinician responds "yes".
3. If smart prompt fires and patient is going to the ICU, clinicians can override the prompt.

Will INSPIRE impact ED sepsis core measures?

The CPOE smart prompt **does NOT** affect "time to antibiotics" because it does not impact whether antibiotics should be used but rather gives real-time information to help decide whether extended-spectrum or standard-spectrum antibiotics should be used. The recommended standard-spectrum antibiotic, ceftriaxone, is compliant with core measures.

Will patients need to give informed consent?

No. This study has been approved as a minimal risk study and will be implemented as a quality improvement (QI) protocol through the existing infrastructure of your hospital's antibiotic stewardship programs. The assigned protocol will be approved through your usual clinical/leadership committees and become hospital guidance on appropriate empiric antibiotic use.

Whom can I contact for more information?

INSPIRE-ASP Study Team:
HCAINSPIRE@gmail.com, (844)-522-4677

Frequently Asked Questions
Clinician Prescribers

What are the INSPIRE-ASP Trials?

The INSPIRE-ASP (**IN**telligent **S**tewardship **P**rompts to **I**mprove **R**eal-time **E**mpiric **A**ntibiotic **S**election for **P**atients) Trials are two separate cluster-randomized trials of HCA Healthcare (HCA) hospitals to assess best practice in antibiotic stewardship for non-ICU patients with pneumonia and urinary tract infection (UTI). Hospitals will be randomized to:

- **Arm 1** – **Routine Care**
- **Arm 2** – **CPOE Smart Prompt Alert Intervention**
  Precision medicine smart prompt using a computerized physician order entry (CPOE) alert that supports antibiotic prescribing with:
  1. Patient-specific estimate of risk for pneumonia or UTI due to a multi-drug resistant organism (MDRO) or *Pseudomonas*
  2. Recommendations for standard-spectrum antibiotics in non-ICU patients admitted for pneumonia or UTI with low MDRO risk (<10%)

What are the outcomes of the INSPIRE-ASP Trials?

The goal is to identify a best practice strategy for choosing empiric antibiotics (within 3 calendar days) based on a patient's risk for MDRO infection. Outcomes include:

- Primary: Empiric extended-spectrum antibiotic use (first 3 hospital days)
- Secondary: Post-empiric extended-spectrum antibiotic use (hospital day 4 to discharge), fluoroquinolone use, antibiotic costs, MDRO and *C. difficile* rates
- Safety outcomes: length of stay, 30-day readmissions, antibiotic escalations, and ICU transfers

Which patients are included in the intervention?

Only adults admitted to a non-ICU ward with pneumonia or UTI who are ordered to receive an extended-spectrum antibiotic within 3 days of admission will be included in the smart prompt alerts. The INSPIRE smart prompt alerts are programmed to not fire for patients younger than 18 years or who are in an ICU location**.** Patients admitted from jail or prison will **not** be included in this study.

What is the rationale for the INSPIRE-ASP Trials?

The Centers for Disease Control (CDC) estimates that 20-40% of extended-spectrum antibiotics in hospitals are unnecessary. One study showed that 33% of pneumonia patients received MRSA treatment even though only 2% were confirmed to have MRSA by culture. Empiric use of standard-spectrum antibiotics is expected to reduce overall extended-spectrum antibiotic exposure during the hospital stay.

How will this help me make better antibiotic choices?

Current approaches to choosing antibiotics should take into account an individual patient's risk for an MDRO infection to decide if extended-spectrum antibiotics can be avoided. However it is cumbersome for clinicians to efficiently and accurately estimate MDRO risk for patients when selecting antibiotics. Current hospital antibiograms do not give clinicians information about a specific patient's risk. The INSPIRE CPOE smart prompt alert will provide clinicians with a patient-specific MDRO risk estimate in real-time to optimize standard antibiotic selection.

How will this help my patient and the community?

Minimizing exposure to extended-spectrum antibiotics can reduce your patient's risk for future MDRO infections, *C. difficile*, and hospital readmission. MDRO infections are harder to treat due to limited treatment options and are associated with higher risks for complication, longer length-of-stay, and death. This trial is expected to decrease extended-spectrum antibiotic use at 29 HCA hospitals across the nation. If successful, findings from this trial have the potential for changing empiric antibiotic selection and stewardship practices at a national level.

What does the INSPIRE-ASP computerized physician order entry (CPOE) smart prompt alert do?

The CPOE smart prompt alert provides clinicians with a patient's probability of having a pneumonia or UTI due to an MDRO or *Pseudomonas*. This estimate is based upon

patient-specific characteristics and hospital antibiotic susceptibility. The alert will give recommendations on standard-spectrum empiric antibiotics in patients admitted for pneumonia or urinary tract infection. The alert is triggered only when an extended-spectrum antibiotic is ordered in non-critical care units.

Is INSPIRE-ASP one trial or two?

The INSPIRE-ASP trials will be rolled out together but are actually two separate trials: the **INSPIRE-ASP Pneumonia Trial** and **INSPIRE-ASP UTI Trial**. Because there are important differences between the two infection syndromes, our smart prompt will need to provide separate risk estimates for pneumonia and UTI. All analyses will need to be done separately to properly evaluate the impact of our intervention. However, all other aspects of the CPOE smart prompt intervention will be rolled out together.

Why will the study focus on pneumonia and UTI?

Pneumonia and UTI are the top two most common reasons for infection-related admission to the hospital and account for the majority of antibiotic use nationally.

What makes the INSPIRE CPOE prompt "smart"?

The INSPIRE smart prompts are automated, real-time alerts built into an electronic medical record that are programmed to return an **individual patient's specific risk** for MDRO or *Pseudomonas* when an extended-spectrum antibiotic is ordered. This risk estimate is tailored to each antibiotic selected. For example:

- If ceftriaxone ordered → no prompt (recommended)
- If vancomycin ordered → returns MRSA risk
- If cefepime or piperacillin/tazobactam ordered → returns *Pseudomonas* risk
- If linezolid ordered → returns MRSA and VRE risk
- If meropenem ordered → returns ESBL and other resistant gram negative bacteria risk

How does the CPOE smart prompt work?

The smart prompt alert will look at your patient's electronic chart for specific risk factors in real-time and calculate the risk that the patient you are seeing has an infection due to an MDRO. If this risk is low (<10%), then the smart prompt will recommend standard-spectrum antibiotics. For example, if you order cefepime on a patient with pneumonia and the smart prompt calculates a <10% risk of *Pseudomonas*, then the smart prompt will recommend using ceftriaxone instead (Figure 1). Choosing

the replace button will automatically take you to the ceftriaxone ordering screen. If the risk is high (>=10%), then no additional screens will fire and you will automatically continue ordering cefepime.

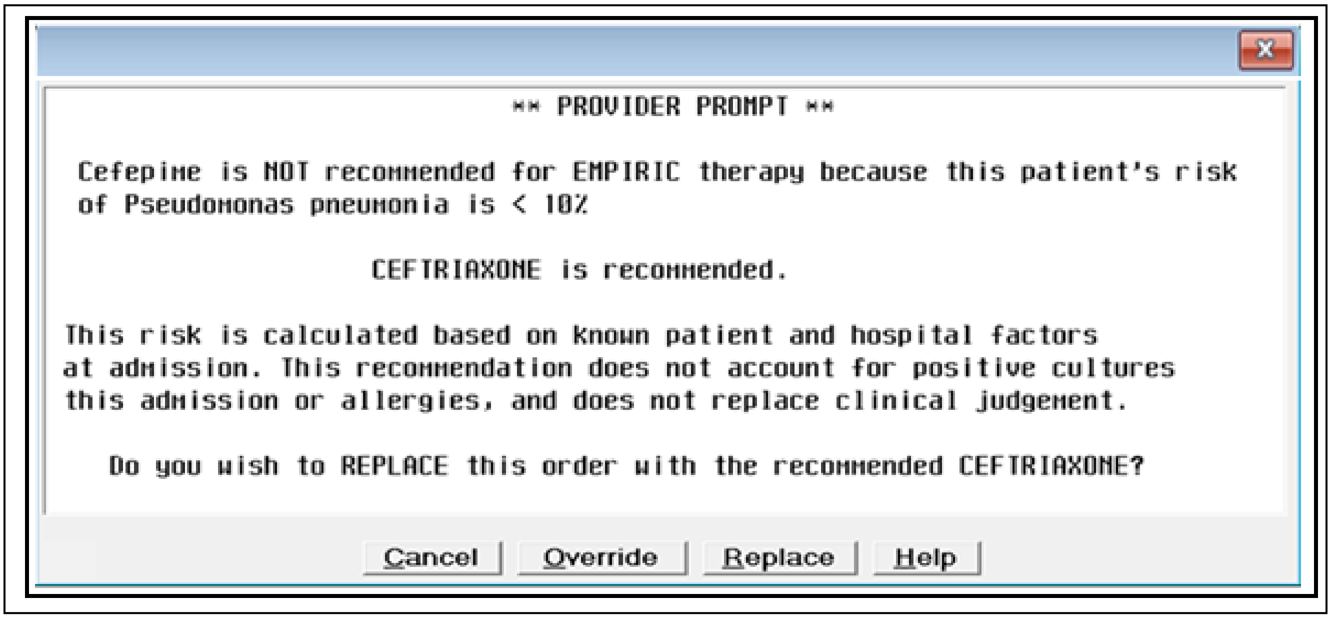

Figure 1. Example screenshot of CPOE smart prompt recommendation triggered when cefepime is ordered for patient with pneumonia.

What if I need to override the CPOE recommendation?

The INSPIRE-ASP smart prompts are meant to support, not replace clinical judgment and there may be good reasons why you may want to override the recommended antibiotic. If you choose to override, you will be asked to provide a reason (Figure 2). You will then continue with your extended-spectrum antibiotic order.

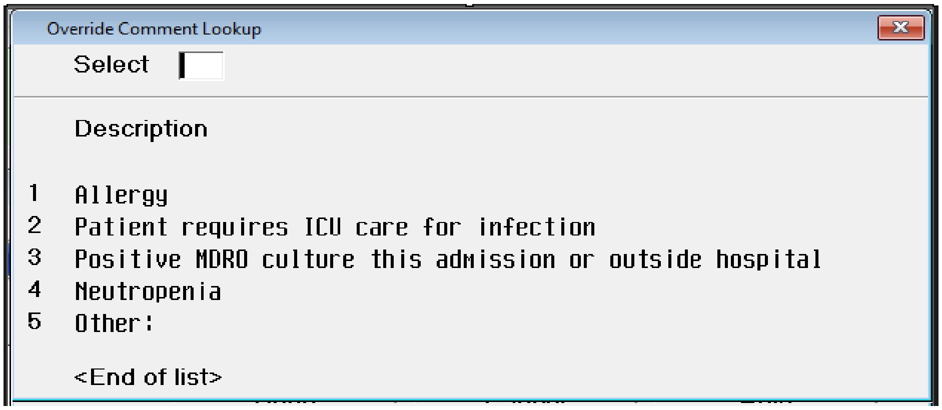

Figure 2. Example screenshot – prescribers will be asked to provide a reason for override.

How are the smart prompts activated?

The CPOE smart prompts are activated when clinicians choose the following indications for extended-spectrum antibiotics: Pneumonia, UTI, sepsis due to pneumonia or UTI, or empiric (unknown) source (Figure 3).

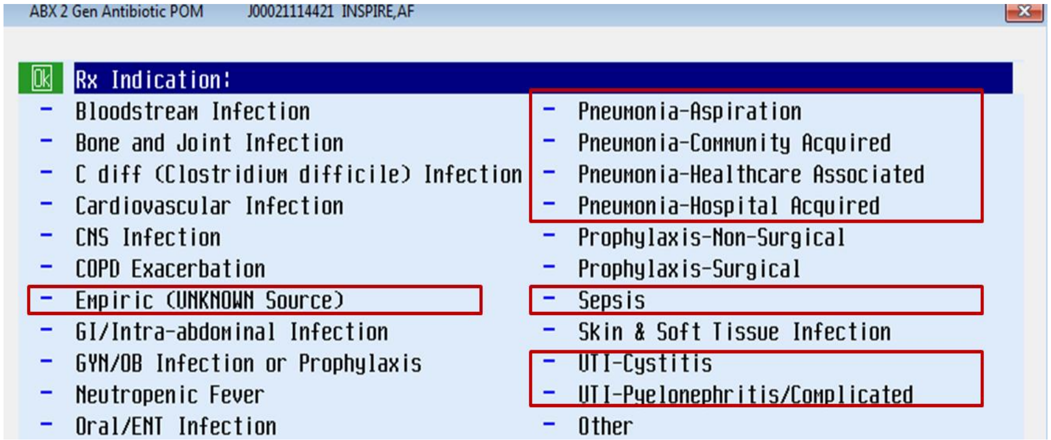

Figure 3. **Screenshot of HCA antibiotic indication screen. Indications highlighted in red will trigger the INSPIRE CPOE smart prompt alert.**

Which antibiotics do NOT trigger the smart prompt?

Narrow spectrum antibiotics do NOT trigger the smart prompt. Examples of standard spectrum antibiotics include:

- For pneumonia: ceftriaxone, ceftriaxone/azithromycin, ampicillin-sulbactam
- For UTI: ceftriaxone, nitrofurantoin

Why was 10% used as a cut-off to identify high versus low risk patients?

National experts advocate the use of standard spectrum antibiotics in patients with less than 10% risk of MDRO in some infections, including pneumonia. Several studies have shown that the risk of MDRO as a cause of pneumonia or UTI is extremely low. For example, our retrospective evaluation of the HCA data shows that among almost 230,000 patients hospitalized with pneumonia, less than 2% grew MRSA by culture. Similar findings have been reported in multiple other studies.

What factors were taken into account when estimating my patient's risk for an MDRO infection?

The INSPIRE study team assessed over 536,000 patients admitted at all HCA hospitals over 3 years (2014-2016) within Meditech. We evaluated (1) your hospital antibiogram for pneumonia and UTI patients and (2) patient risk factors of MDRO infection:

- Demographics (age, gender, race, ethnicity, insurance status)
- Comorbid conditions (e.g., diabetes, lung disease, renal failure)

- History of ED visit or admission to an HCA hospital
- History of nursing home admission
- History of antibiotics received during an ED or inpatient admission
- History of MDRO (screening or clinical cultures at your facility)
- Lab values (e.g., BUN, creatinine)

Using a special statistical method, we identified which risk factors were most often found in patients who developed pneumonia or UTI with an MDRO. Using these risk factors (which differ for every MDRO), the CPOE smart prompt gives the patient's **absolute risk** for infection with the MDRO(s) targeted by the antibiotic ordered.

Note that INSPIRE calculates your facility antibiogram as the proportion of MDRO or pseudomonas among patients admitted with PNA or UTI, which is different than the traditional way most microbiology labs calculate this.

For more detailed information, you can review an audio-enabled presentation on the HCA Atlas website, entitled "How the MDRO Risk Estimates Work": http://connect.medcity.net/web/hca-research-academic-affairs/inspire

Will risk estimates be validated after the intervention starts?

Our risk estimates will be re-validated at regular intervals during the intervention. Significant changes in select variables (e.g., changes in your antibiogram) will be updated within the CPOE alert. Note that our estimates are based on 3 years of data over 140 HCA hospitals, it is unlikely that additional accrual of trial time will change risk factors that have already been identified.

How far back does the CPOE smart prompt look for MDRO history?

The CPOE smart prompt mines your facility's microbiology data in Meditech as far back as 2012. It will capture a patient's MDRO history even if there is no electronic MDRO flag on chart, but it will not capture MDRO history or cultures from an outside hospital.

How quickly can the CPOE smart prompt account for positive cultures? What if a culture was sent recently?

The CPOE smart prompt captures cultures resulted in Meditech at your facility 7 or more days ago; it currently cannot integrate new cultures resulted in the empiric period. For example, if a culture was sent upon admission, this will not be included in a risk estimate when ordering extended-spectrum antibiotics on hospital day 3. We

encourage close monitoring of all new microbiology results as they become available and selection of antibiotics accordingly.

What if my patient is neutropenic, going to the ICU, or has a positive MDRO culture from an outside hospital?

The smart prompt is intended to support, not replace clinical judgment. You should use all available information about a patient as you consider your antibiotic choice, such as: neutropenia, allergy to recommended antibiotics, imminent admission to the ICU, or MDRO positive cultures from this admission or an outside hospital.

What if the smart prompt calculates my patient's MDRO risk to be low but culture eventually grows an MDRO? Does this mean the smart prompt does not work?

As clinicians, we are trained to take our best educated guess on the most likely diagnoses to explain patient symptoms and order testing and treatment accordingly. This smart prompt provides a robust estimate of MDRO risk to support clinical decision-making in the empiric period when cultures are not known. If the prompt finds a patient's risk for MRSA pneumonia is 2.5%, this risk is low, but not zero. It is expected that on rare occasions patients will grow an MDRO when the smart prompt said it was unlikely. With close monitoring, there is time to await cultures before broadening coverage in the non-ICU patient. Clinicians are expected to use clinical judgment, monitor patients daily, and adjust antibiotics as needed based on microbiology results and patient response to treatment.

Are standard spectrum antibiotics safe when MDRO risk is low?

Yes. Studies have shown that in non-ICU patients, there is time to observe patients on standard-spectrum antibiotics, even when an MDRO grows later. A delay in antibiotics in non-ICU patients does not necessarily increase morbidity or mortality.[1-3] The smart prompt is intended to support, not replace clinical judgment. We encourage close monitoring of all patients and if a patient clinically progresses despite starting standard-spectrum antibiotics, clinicians are expected to broaden antibiotic coverage.

References: [1]Rhedin, S., et al. Clinical Microbiology and Infection, 23.4 (2017): 247-252. [2]Kalil, A., et al. Clinical Infectious Disease, CID 2016:63 (1 September). [3]Lin, Michael Y., Antimicrobial agents and chemotherapy 52.9 (2008): 3188-3194

Will I receive feedback on my antibiotic use?

Yes. Your facility's study champions will share provider-specific feedback on empiric antibiotic selections, how often standard-spectrum antibiotics were chosen, how often recommendations were declined, and reasons for not following recommendations. This data is intended to help you evaluate empiric antibiotic selections.

Has the Antibiotic Stewardship Committee and other leadership approved of the smart prompt alert process and recommendations?

Yes. The smart prompt alert and study protocol has been reviewed and approved by your antibiotic stewardship team, hospital committee(s), your hospital leadership, HCA corporate, and subject matter experts at the CDC.

Will patients need to give informed consent?

No. This study has been approved as a minimal risk study and will be implemented as a quality improvement (QI) protocol through the existing infrastructure of your hospital's antibiotic stewardship programs. The assigned protocol will be approved through your usual clinical/leadership committees and become hospital guidance on standard spectrum empiric antibiotic use.

Has this study been approved by the Institutional Review Board (IRB)?

Yes. Harvard Pilgrim's IRB will provide ethical oversight for these trials and serve as the central IRB. Both INSPIRE-ASP PNA and UTI trials have been approved by the Harvard IRB. Your hospital has submitted all ceding and reliance agreements necessary to grant oversight by the central IRB at Harvard. HCA's corporate compliance and regulatory affairs will help provide support for this endeavor.

What is the timeline?

- In-person site visits – Summer-Fall 2018
- IT CPOE smart prompt alert testing – Fall, 2018
- Intervention launch – October 1, 2018
- Phase-in – 6 months
- Intervention – 15 months

Essential Clinician Frequently Asked Questions: What you need to know

What does the INSPIRE Smart Prompt do? (What's smart about it?)

The INSPIRE smart prompt is triggered when MDs order extended-spectrum antibiotics for pneumonia or UTI within the first 3 days of hospitalization in non-ICU patients. The smart prompt uses your patient's specific health data from the HCA electronic medical record and your hospital's antibiogram to estimate the risk that your patient is infected with an antibiotic-resistant organism. It does this in relatively real time during the antibiotic ordering process and gives guidance on whether ordering of empiric extended-spectrum antibiotics is warranted.

The INSPIRE smart prompt also provides the specific multidrug-resistant organism (MDRO) risk targeted by the ordered antibiotic. For example:

- If vancomycin ordered → prompt returns likelihood of MRSA infection
- If cefepime or piperacillin/tazobactam ordered → returns *Pseudomonas* risk
- If linezolid ordered → returns combined MRSA and VRE risk
- If meropenem ordered → returns combined ESBL + resistant gram negative bacteria risk

What does the INSPIRE Smart Prompt NOT do?

The INSPIRE prompt only provides **empiric risk** of an MDRO infection when you do not have culture results to direct therapy. **Smart prompt guidance should be ignored if cultures return positive this admission** (blood/respiratory for pneumonia, and blood/urine for UTI). At that point, therapy should be culture-directed. Specifically, the algorithm only accounts for historical cultures, not cultures taken this admission or within the past 7 days. Additionally, it does not include outside cultures from physician offices or non-HCA facilities.

Which antibiotics do NOT trigger the smart prompt?

Standard antibiotics do NOT trigger the smart prompt. Examples of standard therapy antibiotics include:

- For pneumonia: ceftriaxone, ceftriaxone/azithromycin, levofloxacin, ampicillin-sulbactam
- For UTI: ceftriaxone

What's the harm in empiric anti-MRSA and anti-pseudomonal therapy? Isn't this important for the sepsis guidelines?

Clinicians are pulled in two directions. On one hand, there is urgency to give antibiotics to those who need it. On the other hand, antibiotic stewardship teams urge us to exercise good judgment to prevent antibiotic resistance, unnecessary side effects, and loss of effective agents. INSPIRE smart prompts are directed toward **non-ICU patients** with possible pneumonia and UTI. Evidence supports initial empiric treatment with standard-spectrum antibiotics in non-ICU and non-neutropenic patients while cultures are pending to determine if extended-spectrum antibiotics are appropriate. In addition, **all antibiotics recommended by INSPIRE prompts meet required sepsis measures.**

The prompt says my patient is low risk for an MDRO infection. What does that mean?

If your patient has a 10% or greater risk of an MDRO infection, it is reasonable to use an empiric extended-spectrum antibiotic. However, if your patient's risk is <10%, the prompt will say your patient has a low risk of MDRO infection and standard-spectrum antibiotics should be used while cultures are pending.

My patient just cultured MRSA from sputum, but the prompt told me yesterday that my patient was low risk for MRSA pneumonia. Doesn't that mean the prompt doesn't work and that I should have ordered vancomycin on admission?

Your patient had a low risk of MRSA pneumonia based upon risk factors in your hospital's electronic medical record. Clinicians always need to use their best judgment for estimating risk, so if you had no additional information to the contrary, then this patient's risk was accurately assessed. **Low risk does not mean no risk.** At most, 1 in 10 low risk patients will have an MDRO infection, most have a risk far lower than 10%. Again, evidence suggests that non-ICU, non-neutropenic patients with pneumonia or UTI can be treated with standard-spectrum antibiotics until cultures return.

My patient has a positive blood culture for *Pseudomonas*, but the prompt is telling me that my patient is low risk for *Pseudomonas* UTI. What is wrong with the prompt?

The INSPIRE prompt only provides **empiric risk** of an MDRO infection when you do not have culture results to direct therapy. **Smart prompt guidance should be ignored when cultures return** (blood/respiratory for pneumonia, and blood/urine for UTI). At that point, therapy should be culture-directed. Specifically, the algorithm only accounts for historical cultures, not cultures taken this admission.

How timely is the history of MDRO data that feed into the smart prompt? Does it include positive cultures from today?

Currently, the INSPIRE smart prompt reliably accounts for your hospital's microbiologic culture results up to one week prior to admission. We are actively working to improve the time for capturing more recent culture results, and will keep participating sites updated on our progress.

Why is a history of an MDRO not always enough to warrant extended-spectrum antibiotic use to cover that MDRO?

It depends on the MDRO and the indication (pneumonia or UTI). Sometimes, an MDRO history is sufficient, but other times an MDRO history alone is insufficient to meet the 10% risk threshold without additional risk factors. Remember, colonization with a pathogen in the past does not always mean that it is responsible for a current infection. The value of the smart prompt algorithm is that it used over 500,000 patients over 3 years to determine what generates a risk >10%. An ATLAS webinar is available for those who wish to understand greater details about the algorithm.
(http://connect.medcity.net/web/hca-research-academic-affairs/inspire)

Are allergies auto-programmed into the smart prompt?

No. Allergies are often not recorded correctly in the EHR. Those listed may not be real, and real ones may be unlisted. For example, among patients labeled as beta-lactam allergic without anaphylaxis, over 90% are able to tolerate a beta-lactam after thorough assessment of allergy history. We ask all MDs to critically evaluate allergies when ordering antibiotics. In addition, your standard hospital alerts for allergies still apply.

Are the clinician progress reports to be used by HCA as a quality indicator for the physician dashboard?

No. Progress reports are provided for local pharmacy, ID, ED, and hospitalist champions to support judicious antibiotic prescribing. The hope is that it will build collaboration across the intervention arm and potentially provide a best practice strategy for the field.

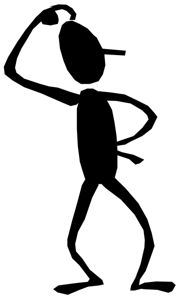

Smart Prompt Clinician Prescriber
Do's & Don'ts

DO

- Follow the smart prompt recommendations for patients with PNA or UTI admitted to non-ICU wards
- Follow your hospital's sepsis protocol
- Use your clinical judgment to decide which antibiotic is best for your patient
- Review your patient's history carefully to identify risk factors for severity of illness or neutropenia
- Review your smart prompt acceptance and respond to your antibiotic stewardship team's feedback and recommendations

DON'T

- Do not use extended-spectrum antibiotics when they are not indicated
- Do not use medications that are contraindicated for your patient (e.g., allergy to the recommended antibiotic)
- Do not replace your clinical judgment with smart prompt recommendations (e.g., patient is transferred from another hospital for known MRSA pneumonia but prompt recommends ceftriaxone because this history is not in electronic medical record)

Please contact your local Study Champion(s) for INSPIRE-ASP details

Study Investigators & Coordinators
(844) 522-4677
HCAINSPIRE@gmail.com

INSPIRE-ASP Trial

INtelligent Stewardship Prompts to Improve Real-time Empiric Antibiotic Selection for Patients

Agenda

- Welcome
- Context & Rationale
- INSPIRE-ASP Overview
- CPOE Smart Prompt Intervention
- MDRO Risk Estimate Calculations
- Quarterly Clinician Progress Reports
- What to Expect

Welcome to Arm 2

Smart Prompt Alert Intervention

Precision medicine smart prompt alerts for patient-specific empiric antibiotic selection

These trials are a partnership between HCA Healthcare and academic investigators who are federally funded by the Centers for Disease Control and Prevention (CDC)

Context & Rationale

Overuse of Antibiotics: A Major National Priority

- Broad-spectrum antibiotics increase resistance[1]
- 40% of inpatients receive inappropriate antibiotics[2]
  - Majority due to pneumonia or UTI
- Impact on patient safety (side effects, *C. difficile)*
- National and state mandates for stewardship programs

[1]Paterson, D et al. Clin Infect Dis 2014;33:126-128

[2]Fridkin, S et al. MMWR Morb Mortal Wkly Rep 2014;63(9):194-200

INSPIRE
BETTER SAFER ANTIBIOTIC USE

Challenges Driving Broad-Spectrum Antibiotic Use

- **Diagnostics uncertainty**
- **Treatment challenges**
  - Optimal treatment unclear
  - Guidelines broad
- **Medical-legal implications**
- **Physician factors**
  - Knowledge gaps
  - Risk aversion

+

Complicated Hospital Antibiogram

Antibiotic	*E. coli* % Susceptibility	*K. Pneumoniae* % Susceptibility
Ampicillin	51	0
Ampicillin-sulbactam	48	79
Piperacillin-tazobactam	95	95
Cefazolin	58	68
Ceftriaxone	91	92
Cefepime	96	97
Ertapenem	100	100
Meropenem, imipenem	100	100
Gentamicin, tobramycin	90	94
Trimethoprim-sulfa	71	85
Cipro/levofloxacin	77	96
Tigecycline	100	100
Nitrofurantoin	97	31

Causes Broad-Spectrum Antibiotic Overuse

INSPIRE BETTER SAFER ANTIBIOTIC USE

Empiric Antibiotic Choice: There is Time to Broaden

- Growing evidence in non-ICU patients – little/no harm in delay
- Similar outcomes seen in non-ICU patients when treated with empiric standard vs broad-spectrum antibiotics[1]
- Waiting for culture results to start anti-MDRO antibiotics does not adversely impact mortality[1,2]

[1]Rhedin, S., et al. Clinical Microbiology and Infection, 23.4 (2017): 247-252.
[2]Lin, Michael Y., Antimicrobial agents and chemotherapy 52.9 (2008): 3188-3194.

Empiric Antibiotic Choice: There is Time to Broaden

- In **non-ICU** patients admitted for pneumonia:
  - Narrow-spectrum antibiotics safe, no increased mortality[1]
  - Pathogens similar in CAP and HCAP[1]
- HCAP designation removed from IDSA HAP/VAP Guidelines[2]
- Non-ICU patients with ANC >100, waiting for culture results not associated with higher mortality[3]

[1]Rhedin, S., et al. Clinical Microbiology and Infection, 23.4 (2017): 247-252.
[2]Kalil, A., et al. Clinical Infectious Disease, CID 2016:63 (1 September).
[3]Lin, Michael Y., Antimicrobial agents and chemotherapy 52.9 (2008): 3188-3194.

How Can We Influence Prescribing?

- U.S. far higher use of extended-spectrum antibiotics vs. other 1st world nations[1]
- UK guidelines for pneumonia:
  - Low severity: amoxicillin
  - Moderate severity: amoxicillin + macrolide
- Australian guidelines for severe sepsis due to pneumonia includes penicillin as part of the base combination therapy[2]

[1]https://www.nice.org.uk/guidance/cg191/chapter/1-Recommendations

[2]http://www.cec.health.nsw.gov.au/data/assets/pdf_file/0005/299417/adult-antibiotic-guideline-severe-sepsis-septic-shock-sept2016.pdf

Rationale for INSPIRE-ASP Trial

- Uncertainty about MDRO risk drives overuse of empiric extended-spectrum antibiotics
- Physicians can choose wisely if
  - Actionable information provided in real time
  - Recommendation based on individual patient
  - Feedback provided

Two Trials in One:

INSPIRE-ASP Trials

INtelligent Stewardship Prompts to Improve

Real-time Empiric Antibiotic Selection for Patients

INSPIRE
BETTER SAFER ANTIBIOTIC USE

INSPIRE-ASP Study Design

2-in-1 Set of Cluster-Randomized Trials – 59 Hospitals

  - Trial 1: Pneumonia (PNA)
  - Trial 2: UTI
- One intervention
  - CPOE precision medicine smart prompt alert
  - Real time recommendation for antibiotic choice
  - Feedback on acceptance
- Two separate analyses and manuscripts
- IRB oversight: Harvard Pilgrim
- No informed consent needed

INSPIRE-ASP Trial Arms

Arm 1: Routine Care – 30 Hospitals

- Standard-of-care antibiotic stewardship

Arm 2: Smart Prompt Alert Intervention – 29 Hospitals

- CPOE precision medicine alerts for patient-specific MDRO risk
- Recommends standard-spectrum antibiotic choice
- Provider feedback reports

INSPIRE-ASP Study Population & Primary Outcome – PNA

Includes

- Adults in ED and non-ICU units
- Admitted for **pneumonia (PNA)**

Excludes

- ICU and pediatric patients

Primary Outcome

- Empiric extended-spectrum (ES) antibacterial treatment for **PNA** per at-risk day
  - "Empiric" = first 3 hospital days

INSPIRE-ASP Study Population & Primary Outcome – UTI

Includes

- Adults in ED and non-ICU units
- Admitted for **urinary tract infection (UTI)**

Excludes

- ICU and pediatric patients

Primary Outcome

- Empiric extended-spectrum (ES) antibacterial treatment for **UTI** per at-risk day
  - "Empiric"= first 3 hospital days

Other Outcomes

Secondary Outcomes

- Subgroups of vancomycin, antipseudomonals

Safety Outcomes

- Antibacterial escalation
- Length of stay
- ICU transfers

Other Outcomes (secondary manuscripts)

- *C. difficile* infection
- MDRO prevalence
- Cost-analysis

Note: Two trials in one

Each outcome evaluated separately and separate manuscripts planned for PNA and UTI cohorts

Baseline Pneumonia & UTI Due to MDRO – 2014-2017

- Among >500,000 HCA Healthcare (HCA) patients, <2% of PNA patients and <6% of UTI patients grew drug resistant organisms

Vast majority could have been treated with standard-spectrum
Safe to use standard-spectrum while waiting for culture results

	MDRO Positive Pneumonia (%)	MDRO Positive UTI (%)
MRSA	1.7	1.4
Pseudomonas	1.5	3.4
ESBL	0.4	5.8
CRE	0.03	0.3
VRE	--	0.8

INSPIRE
BETTER SAFER ANTIBIOTIC USE

Extended-Spectrum Overuse in Non-MDRO HCA Patients

- Over 20-fold higher extended-spectrum (ES) use despite very small numbers of MDRO pneumonia or UTI

Model	MDRO Culture Positive		ES Antibiotic Use in Non-MDRO Positive*	
	N	%	N	%
**MRSA PNA**	**3,231**	**1.7**	**67,856**	**34.5**
***Pseudomonas* PNA**	**2,995**	**1.5**	**79,830**	**39.9**
***Pseudomonas* UTI**	**10,492**	**3.4**	**74,657**	**24.5**

HCA baseline data 2014-2017
Pneumonia (PNA), n = 199,903
Urinary tract infection (UTI), n = 304,584
*Note: “ES” = anti-MRSA antibiotic use for MRSA PNA/UTI and antipseudomonal use for *Pseudomonas* PNA/UTI

CPOE Smart Prompt Intervention

When Will CPOE Smart Prompt Fire?

- **Smart prompt only fires if extended-spectrum antibiotics** ordered for **PNA or UTI** in the **ED or non-ICU wards** within **first 3 days** of admission
  - (1) Gives patient's risk of PNA or UTI due to MDRO
  - (2) Recommends standard-spectrum antibiotics when risk is low (<10%)

- **NO prompt will fire** when ordering standard-spectrum antibiotics (e.g., ceftriaxone)

What Antibiotics Are Considered "Extended-Spectrum"?

Category	Extended-Spectrum Antibiotics Included in CPOE Alerts
**Anti-MRSA**	Ceftaroline, Daptomycin, Linezolid[1], Vancomycin[2]
**Anti-VRE**	Daptomycin, Linezolid[1]
**Anti-Pseudomonas**	Aztreonam, Cefepime, Ceftazidime, Piperacillin/Tazobactam
**Anti-ESBL**	Ertapenem, Doripenem, Imipenem, Meropenem, Ceftolozane/Tazobactam,
**Anti-CRE**	Ceftazidime/Avibactam, Colistin, Polymixin B, Tigecycline

[1]Both oral and intravenous (IV) formulations

[2]IV formulation only

CPOE Smart Prompt

- **How is it Smart?**
  - ✓ Uses ***your specific patient*** EHR data to calculate MDRO risk based on models from 500,000 HCA patients with PNA or UTI
  - ✓ Risk estimate tailored to antibiotic selected. For example:
    - ➢ If ceftriaxone ordered → no prompt (recommended)
    - ➢ If vancomycin ordered → returns MRSA risk
    - ➢ If cefepime or Zosyn ordered → returns *Pseudomonas* risk
    - ➢ If linezolid ordered → returns MRSA and VRE risk
    - ➢ If meropenem ordered → returns ESBL + other resistant gram negative bacteria risk
  - ✓ Recommends ceftriaxone when MDRO risk is low (<10%)
  - ✓ If risk ≥10%, extended-spectrum order proceeds without any prompts

CPOE Smart Prompt Activated Via Indication Screens

- INSPIRE prompts triggered based upon selected indication:
  - **PNA**
  - **UTI**
  - **Sepsis/Empiric** – triggers additional screen asking if possible respiratory or urinary source

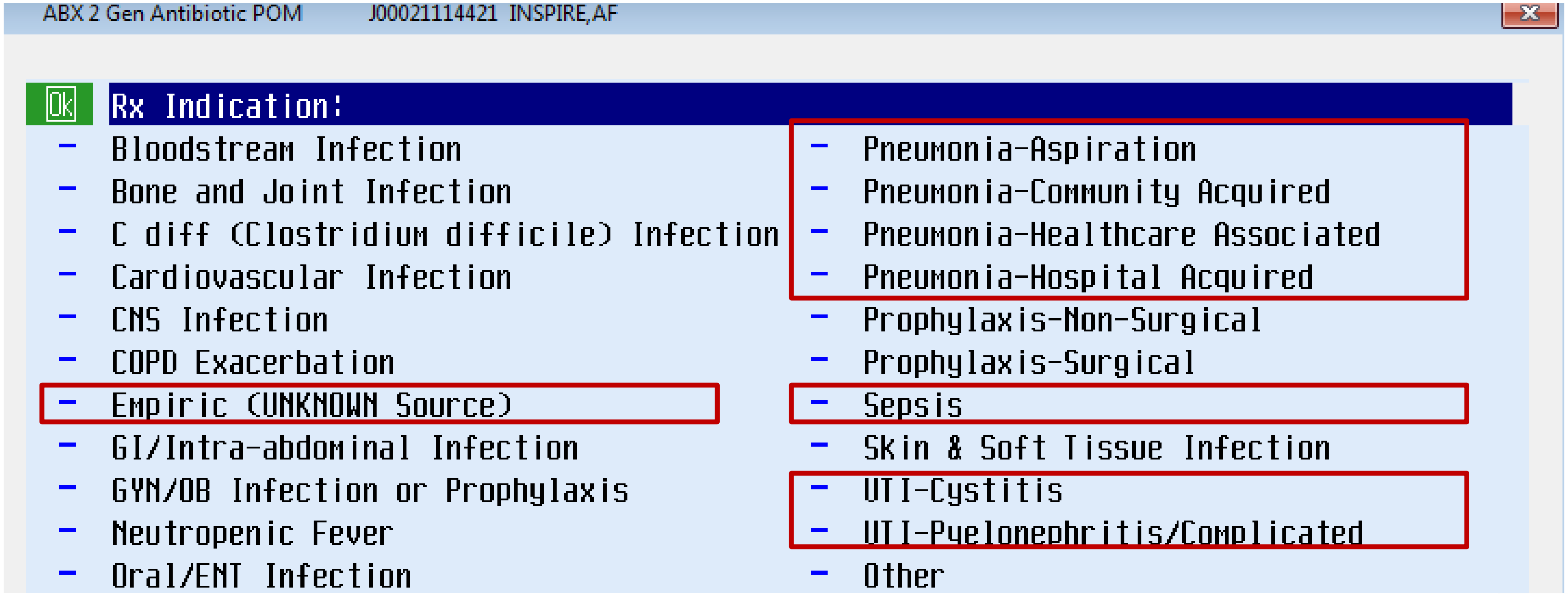

ED Patients Going to ICU

- Smart prompt **<u>will not</u>** fire if patient location = ICU
- **For ED patients**, asks if patient is likely to go to the ICU
  - If YES → no further screens
  - If NO → INSPIRE CPOE screens continue

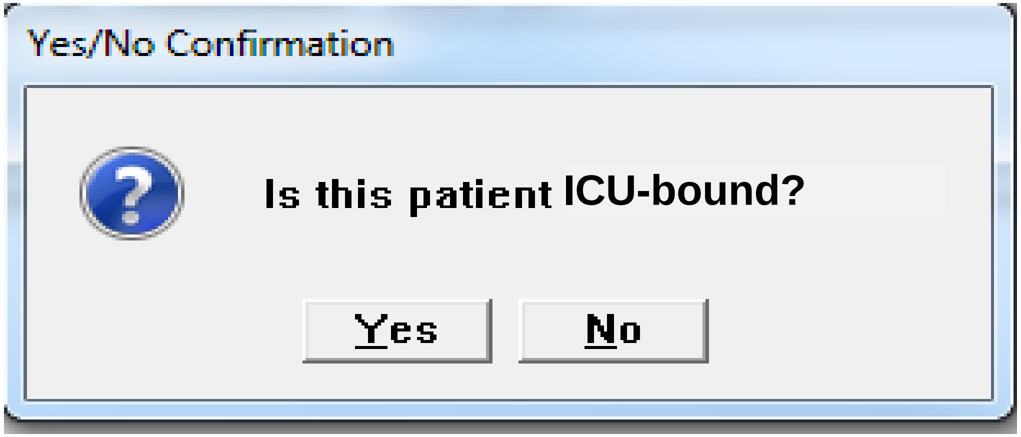

INSPIRE-ASP CPOE Smart Prompt Screen - Example

- Physician orders cefepime for pneumonia and risk of *Pseudomonas* pneumonia calculated to be low (<10%)

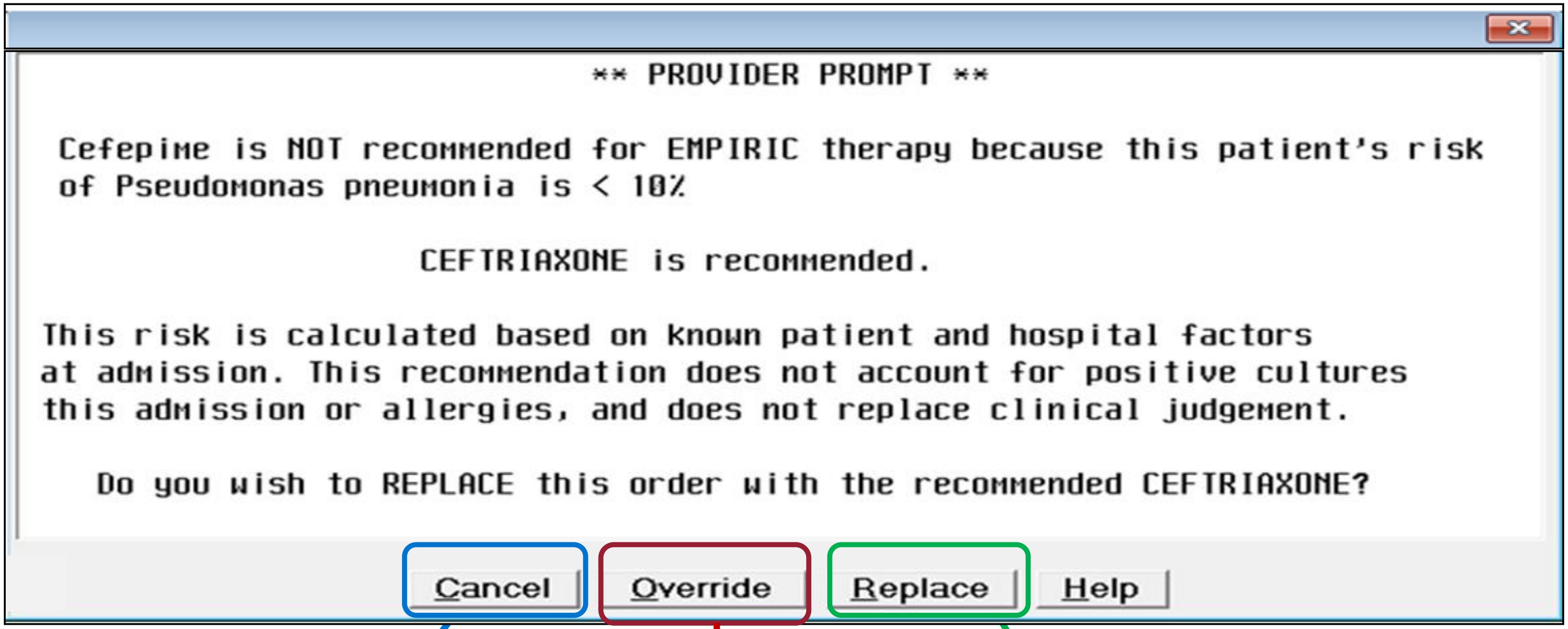

Returns to order screen

Next screen asks reason to keep cefepime

Taken to ceftriaxone order screen

Replacing Extended-Spectrum Antibiotic

- If choose **REPLACE** → CPOE takes physician directly to ceftriaxone ordering screen

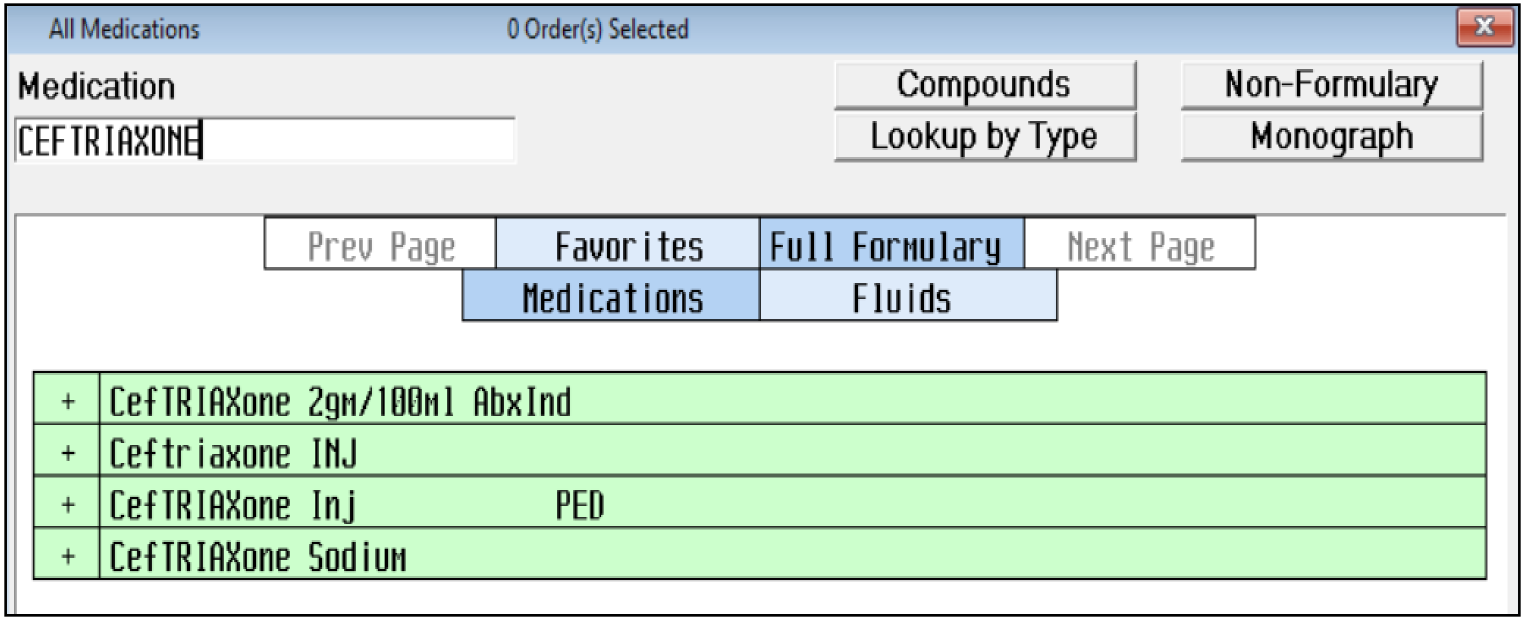

Override Screen and Options

- Clinicians choosing to **override** are asked to indicate the reason:

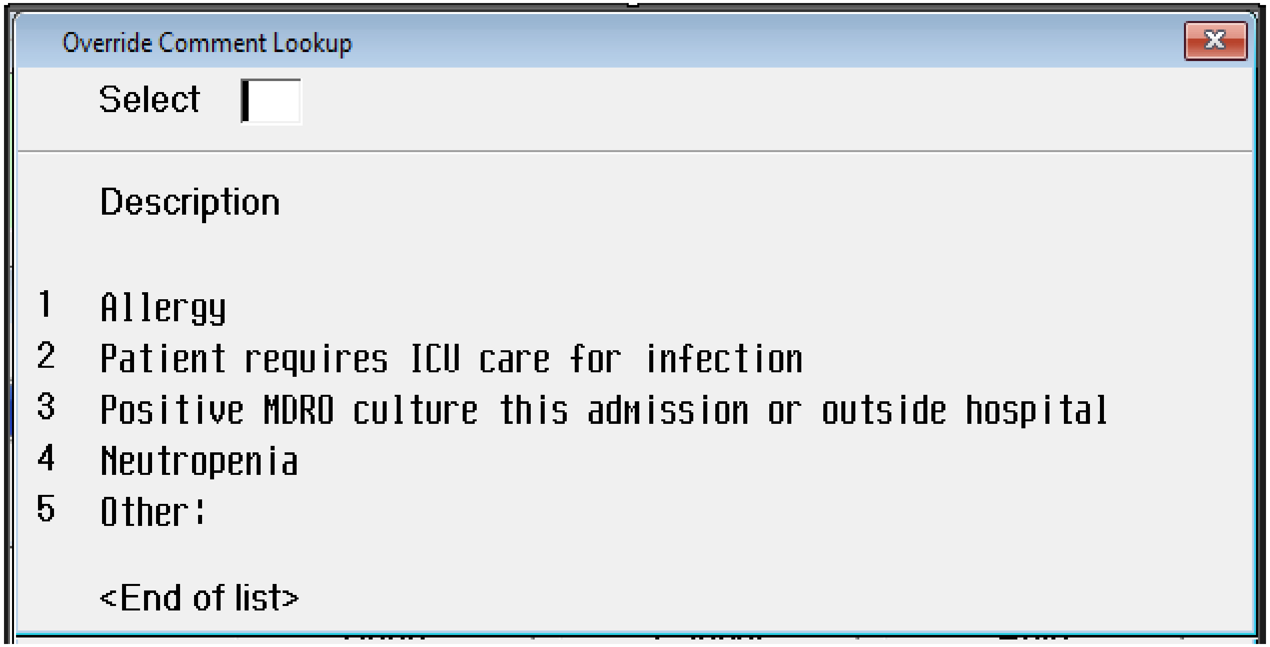

******Allergy* ***not*** *auto-programmed:* *some allergies missing in EHR, and many in EHR are not true allergies – needs clinical judgment*

** Usual facility allergy prompts continue to appear*

What Happens if Patient Risk is Higher than 10%?

- If patient's MDRO infection risk higher than 10%, the CPOE alert will automatically allow physician to continue order for extended-spectrum antibiotic

- No CPOE screen will be generated

Why 10%?

- No added risk to wait for culture in non-ICU population
- National experts, including at CDC, agree on INSPIRE threshold
- Extended-spectrum antibiotics incur risks
  - Greater impact to microbiome
  - Increase MDRO selection and acquisition
  - Increase risk of *C. difficile*
  - Increase development of resistance
  - Increased side effects (added antibiotics)
  - Unnecessary expense

Patient MDRO Risk Calculation

The Problem with Relative Risk and Odds Ratios (OR)

- Clinicians are taught about relative risk and odds ratios
- We worry that the OR for an MDRO infection is 2 or 3 for certain factors like nursing home residence or diabetes
- However, this tells us nothing about the absolute risk
- An example
  - Let's say females have a risk of 1 in 1,000 for MRSA pneumonia
  - If males have a risk of 3 in 1,000, that would be OR = 3 for an absolute risk of 0.3%
  - Better to know absolute risk
  - **The INSPIRE trials use absolute risk**

If Not Odds Ratios, Then What?

- Use recursive partitioning for estimating patient risk
- Fancy term for a decision tree
- Parses variables to see which ones matter and in what order
- Generates a final set of important characteristics of high vs low risk using statistics

For INSPIRE-ASP, high risk defined as >= 10%

INSPIRE
BETTER SAFER ANTIBIOTIC USE

Recursive Partitioning Example

Decision tree to identify patients at high vs low risk for cardiac disease

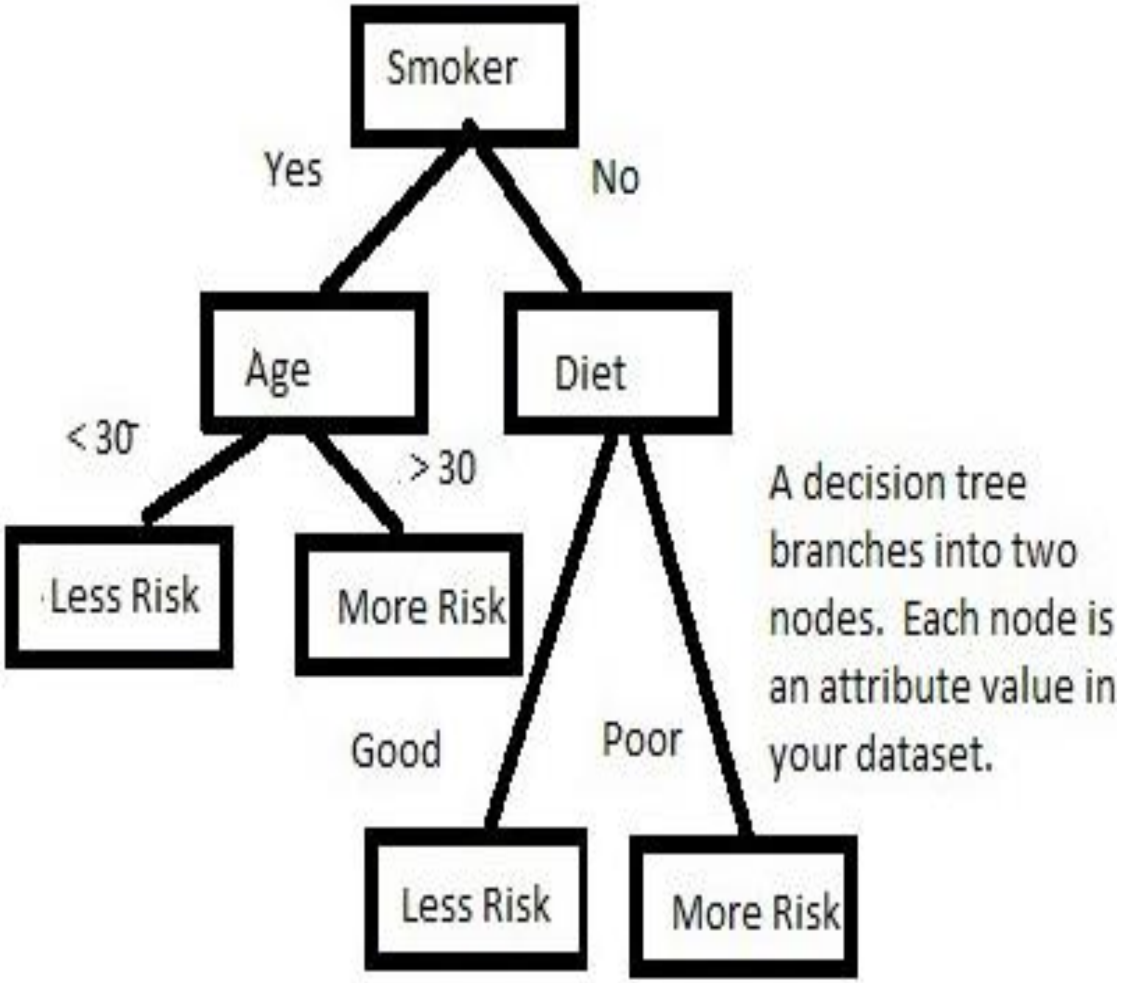

What Variables Were Assessed?

- 536,000 patients admitted to HCA Healthcare hospitals, 2014-2016
- We evaluated:

(1) Your hospital antibiogram for pneumonia and UTI patients

(2) Risk factors of MDRO infection

- Demographics
- Comorbidities
- MDRO or *Pseudomonas* history (screening or clinical culture)
- Recent admission, ED visit, nursing home care
- Recent antibiotic use
- Labs (e.g., BUN, creatinine)

Factors predictive of MDRO infection were programmed into Meditech to provide a patient-specific risk estimate for each MDRO.

INSPIRE
BETTER SAFER ANTIBIOTIC USE

Pseudomonas Pneumonia Risk Estimate Model

- Among all risk factors only history of *Pseudomonas* and percent facility *Pseudomonas* pneumonia were found to be important in distinguishing high from low risk patients

Rules	N	Proportion Predicted with PSE PNA	Admits Receiving Anti-PSE Antibiotics
PSE Hx, % Facility PSE PNA > 2.0%	1588	**18%**	**64%**
PSE Hx, % Facility PSE PNA <= 2.0%	2068	**11%**	**62%**
All orders not meeting above	156375	<=5%	40%

HCA baseline data 2014-2017
Pneumonia (PNA), n = 199,903
"PSE"= *Pseudomonas*, "Hx"= history, "MDR"= multidrug resistant
"ESBL"= extended-spectrum beta lactamase
NOTE: % Facility PSE PNA = number of patients with positive respiratory or blood cultures positive for *Pseudomonas* among all patients admitted with pneumonia.

Pseudomonas UTI Risk Estimate Model

Rules	N	Proportion Predicted with PSE UTI	Admits Receiving Anti-PSE Antibiotics
PSE hx, Male, MDR-PSE hx	895	**28%**	**56%**
PSE hx, Male, No MDR-PSE hx, No ESBL hx	2276	**24%**	**55%**
PSE hx, Female, MDR-PSE hx	833	**21%**	**50%**
PSE hx, Male, ESBL hx, No MDR-PSE hx	784	**18%**	**55%**
PSE hx, Female, No MDR-PSE hx	3152	**14%**	**41%**
No PSE hx, Male, MRSA hx	2684	9%	45%
No PSE hx, Male, ESBL hx, No MRSA hx	2690	7%	40%
All orders not meeting above	230465	<=5%	25%

HCA baseline data 2014-2017
Urinary tract infection (UTI), n = 304,584
"PSE"= *Pseudomonas*, "Hx" = history, "MDR"= multidrug resistant
"ESBL"= extended-spectrum beta lactamase.
NOTE: % Facility PSE UTI = number of patients with positive urinary or blood cultures positive for *Pseudomonas* among all patients admitted with UTI.

Precedence for 10% Threshold

- Prevalence of many MDRO positive cultures well below 10%
- Pneumonia
  - IDSA CAP 2007: “high level” *S. pneumoniae* resistance to azithromycin defined as > 25%
- UTI
  - IDSA UTI 2010: “high level” *E. coli* resistance defined as:
    - > 20% for trimethoprim/sulfamethoxazole (Bactrim)
    - > 10% for fluoroquinolones

Validation and Updating Smart Prompt Risk Estimates

- Risk estimates will be revalidated periodically to ensure accuracy
- Significant changes in select variables (e.g., changes in your antibiogram) will be updated within the CPOE alert
- Note: given assessment of 3 years of data over 140 HCA Healthcare hospitals, it is unlikely that additional accrual of trial time will change prior identified factors
- INSPIRE is a robust assessment

Clinical Judgment Matters

- CPOE alert provides objective, real-time, patient-specific risk estimates to help you choose the best initial antibiotic
- Alert supports, but does not replace clinical judgment
- You may have information about a patient that is not in the computer that should be considered in your decision:
  - MDRO culture this admission or outside hospital
  - Neutropenic
  - Allergic to recommended antibiotic
  - Imminently headed to the ICU
- Clinicians are expected to use clinical judgment, monitor patients daily, and adjust antibiotics as needed based on microbiology results and patient response to treatment

Quarterly Clinician Progress Reports

WHO Principles of Clinical Decision Support

- Speed
- Simplicity
- Usability
- Integration into workflow
- Actionable and provides alternatives
- Evidence/justification provided
- **Impact should be monitored**

Quarterly Clinician Progress Reports - Example

Clinician	Total PNA Non-ICU Admissions N	Empiric Antibiotics Ordered Per Protocol N (%)	Smart Prompts Triggered N (%)	Smart Prompt Declined/ Overridden N (%)	Acceptance of Smart Prompt Recommendation N (%)
**You**	12	8 (67)	4 (33)	3 (75)	9 (75)
**MD 1**	10	5 (50)	5 (50)	2 (40)	8 (80)
**MD 2**	9	3 (33)	6 (67)	4 (67)	5 (56)
**MD 3**	7	4 (57)	3 (43)	1 (33)	6 (86)
**MD 4**	3	2 (67)	1 (33)	1 (100)	2 (67)
**All Clinicians This Hospital**	41	22 (54)	19 (46)	11 (58)	30 (73)
**All Clinicians Arm 2 Trial**	1189	638 (54)	551 (46)	319 (58)	870 (73)

Tracking Declinations and Overrides

Clinician	Reason for Declining or Overriding Smart Prompt				
	Allergy N (%)	Neutropenia N (%)	MDRO Culture N (%)	ICU Bound N (%)	Other N (%)
You	1 (33)	0 (0)	1 (33)	1 (33)	0 (0)
MD 1	0 (0)	1 (50)	0 (0)	1 (50)	0 (0)
MD 2	1 (25)	1 (25)	0 (0)	1 (25)	1 (25)
MD 3	0 ()	0 (0)	1 (100)	0 (0)	0 (0)
MD 4	1 (100)	0 (0)	0 (0)	0 (0)	0 (0)
All Clinicians This Hospital	3 (27)	2 (18)	2 (18)	3 (27)	1 (9)
All Clinicians Arm 2 Trial	87 (27)	59 (18)	58 (18)	86 (27)	29 (9)

INSPIRE BETTER SAFER ANTIBIOTIC USE

Quarterly Hospital Progress Reports

- Acceptance of CPOE smart prompt recommendations will be provided quarterly

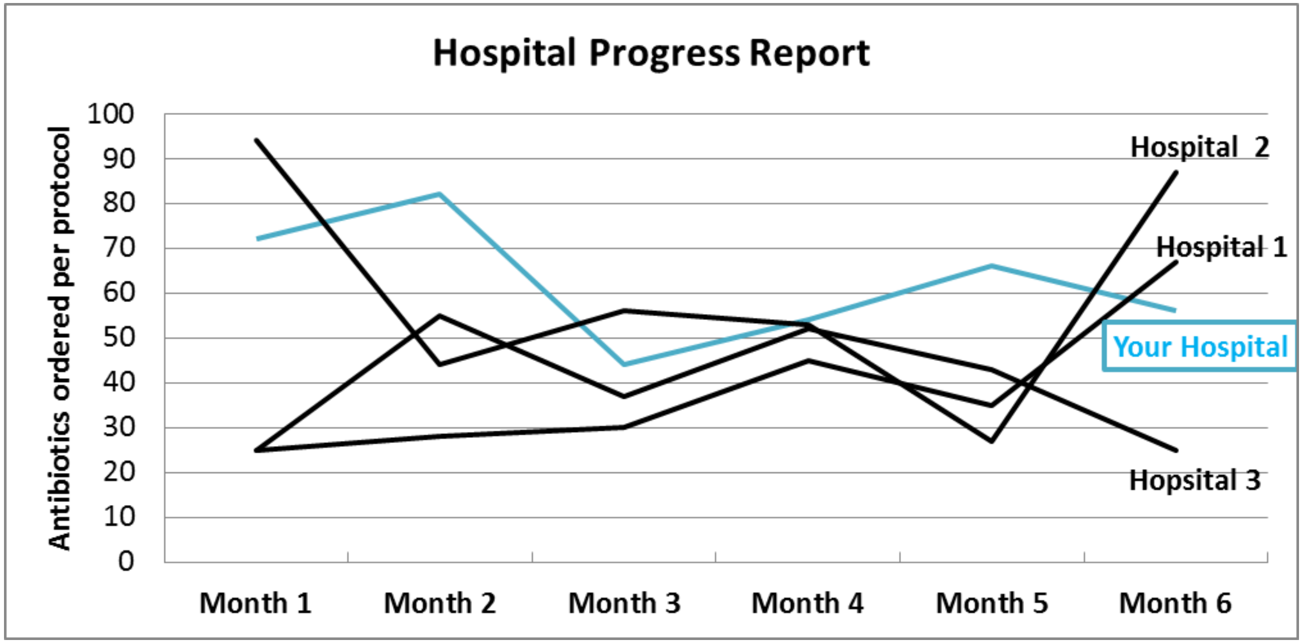

Quarterly Clinician Progress Reports

- Acceptance of CPOE smart prompt recommendations will be provided to stewardship champions and physician quarterly
- Clinician name will only appear on your own report and not shared with other clinicians or hospitals

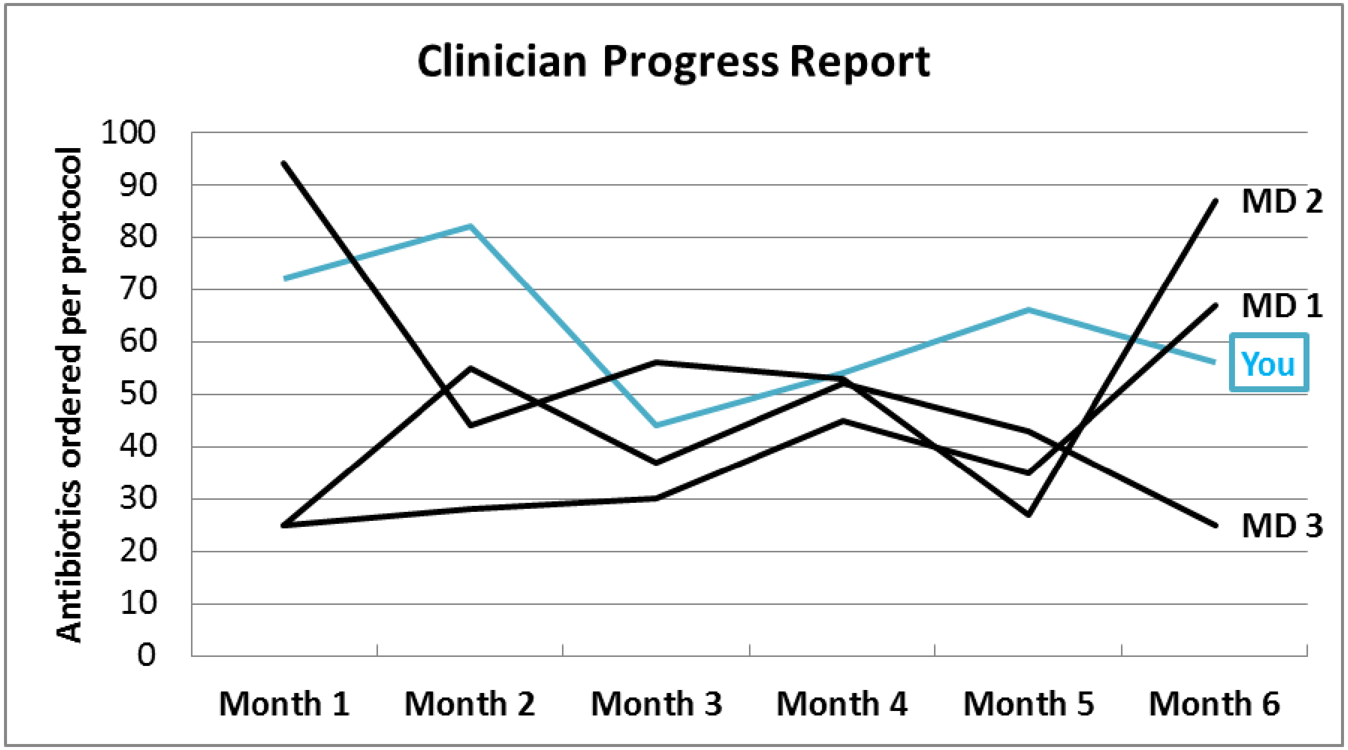

Declinations & Overrides

- Reasons for declining or overriding CPOE recommendations will be monitored

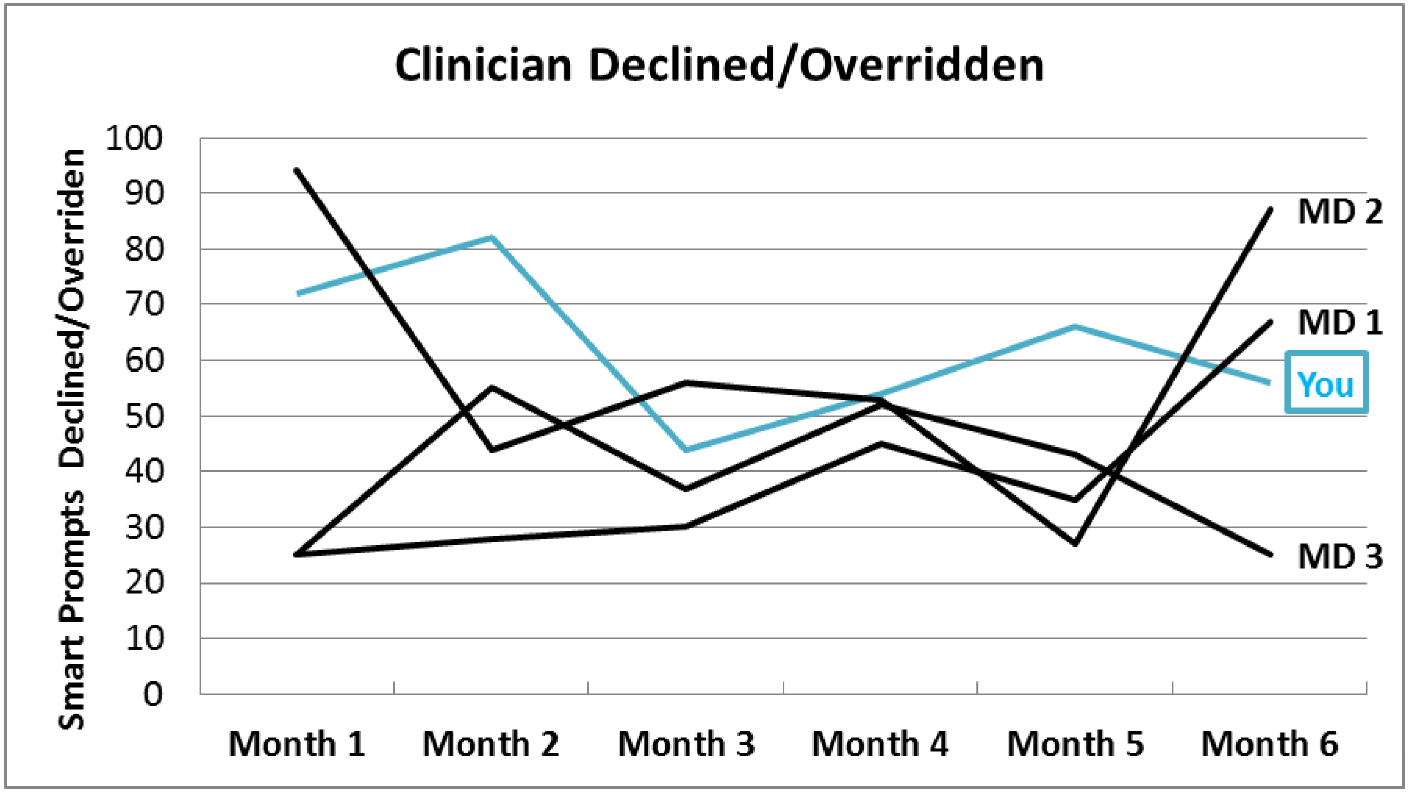

What to Expect

MD and PharmD Champion Roles and Responsibilities

- Educate on value and safety of INSPIRE trial
  - Grand rounds, medical staff meetings (ED, Hospitalists)
- Address clinician outliers in a supportive, knowledgeable way
- Help clinicians understand value of absolute risk
- Review clinician progress reports and encourage improvement
- Set reachable local targets and improvement goals
- Avoid competing interventions

Trial Timeline

- Site visits: **Summer-Fall 2018**
- Intervention launch: **October 1, 2018**
- Phase-in: **6 months**
- Trial duration: **15 months**

Landmark Trial

- First precision medicine tool in antibiotic stewardship
- Real time smart prompt alerts
- Based upon absolute risk, not relative risk
- If successful → best practice electronic support intervention

INSPIRE-ASP Pneumonia and UTI Trials

INtelligent Stewardship Prompts to Improve Real-time Empiric Antibiotic Selection for Patients

ATTENTION: CLINICIANS

<Hospital> is a proud participant in the INSPIRE-ASP Trials to test the **first precision medicine tool in antibiotic stewardship**.

These trials will encourage the **use of standard-spectrum antibiotics (e.g., ceftriaxone) for non-ICU pneumonia and UTI**.

Starting **October 1st**, physicians ordering extended spectrum antibiotics for pneumonia or UTI in the **ED or non-ICU wards within 3 days of admit** will activate a computerized physician order entry (CPOE) smart prompt that will provide:

- **Your specific patient's risk** for pneumonia or UTI due to antibiotic resistant bacteria using EHR data and your hospital's antibiogram
- Recommendations for standard-spectrum antibiotics if risk is estimated to be low (<10%)

If you have questions, please contact:

<Study Champion Name>

<Study Champion Email>

These landmark trials are a partnership between HCA Healthcare and academic investigators federally funded by the CDC.

INSPIRE-ASP
Computerized Physician Order Entry (CPOE) Alert: Work Flow

Beginning **October 1, 2018** our hospital will launch an antibiotic stewardship intervention to **encourage compliance with national guidance for standard-spectrum antibiotics (e.g., ceftriaxone) for non-ICU pneumonia and UTI.**

Ordering extended-spectrum antibiotics (e.g., vancomycin, cefepime) for pneumonia or UTI in the emergency department (ED) or non-ICU wards within 3 days of admit will activate a computerized physician order entry (CPOE) smart prompt alert that will provide:

- Your patient's risk for pneumonia or UTI due to antibiotic resistant bacteria based upon your patient's EHR data and your hospital's antibiogram
- Recommendation for standard-spectrum antibiotics if risk is estimated to be low (<10%)

This document provides details on the INSPIRE CPOE workflow with relevant screen shots.

1. INSPIRE-ASP CPOE prompts trigger off of the HCA Healthcare Antibiotic Indication Screens. They **will only fire if one of the following indications are chosen in the first 3 days of admission** (outlined in red):

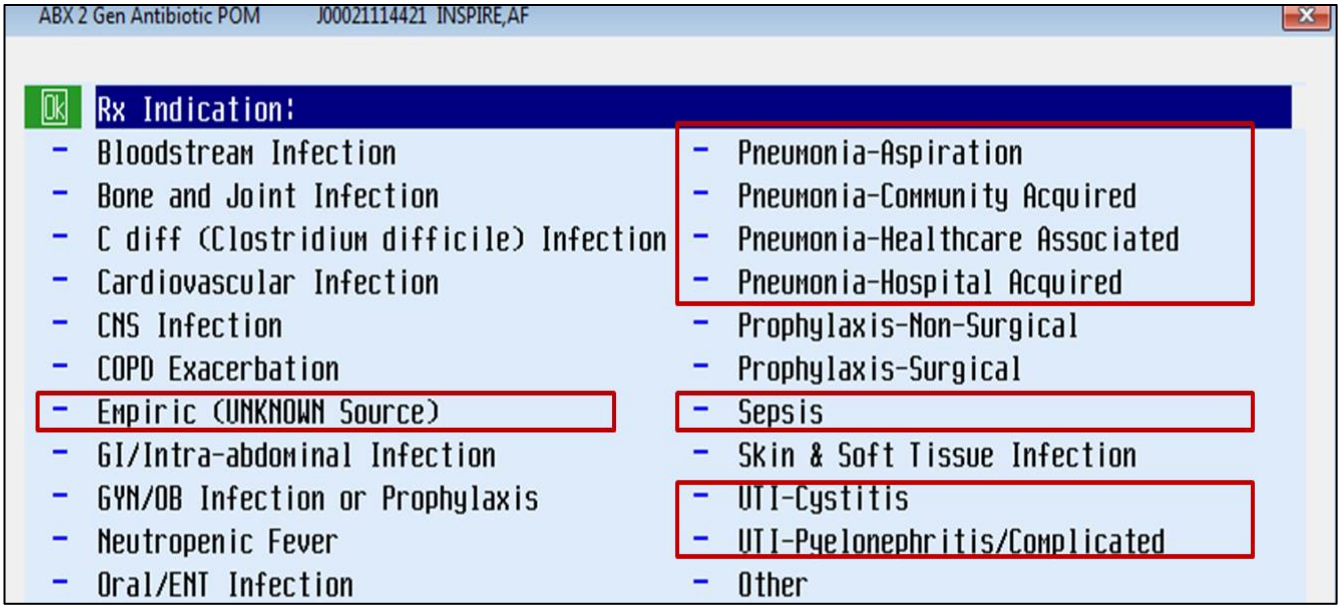

2. INSPIRE prompts **will fire for patients in the ED and non-ICU units only** and are programmed to **NOT** fire if a patient is listed in an ICU location :

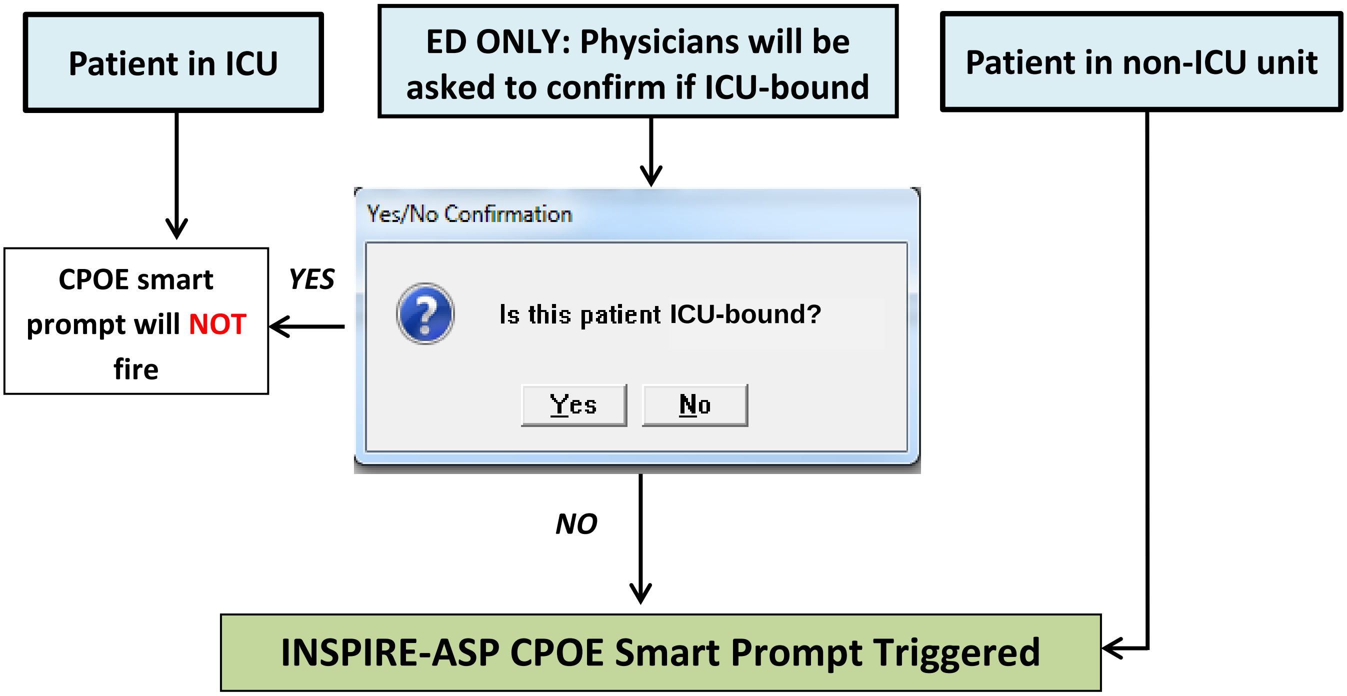

3. INSPIRE prompts will automatically look up the patient's specific risk for infection due to an MDRO.

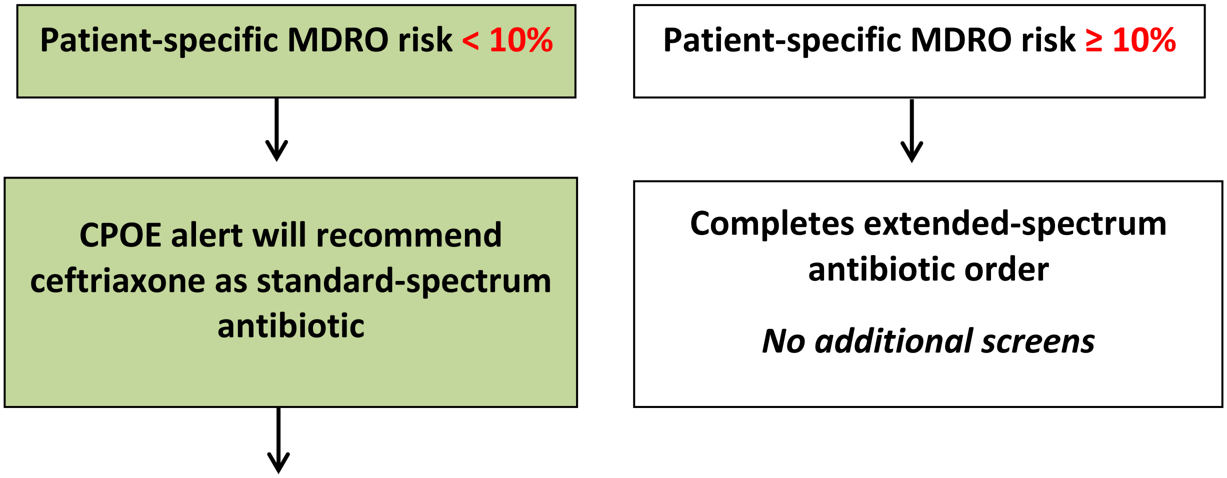

(See following page for mock INSPIRE-ASP CPOE smart prompt)

Mock CPOE Smart Prompt Recommendation Screen

__Example__:

Physician orders __cefepime__ for pneumonia.

Patient risk for *Pseudomonas* pneumonia is calculated to be <10%.

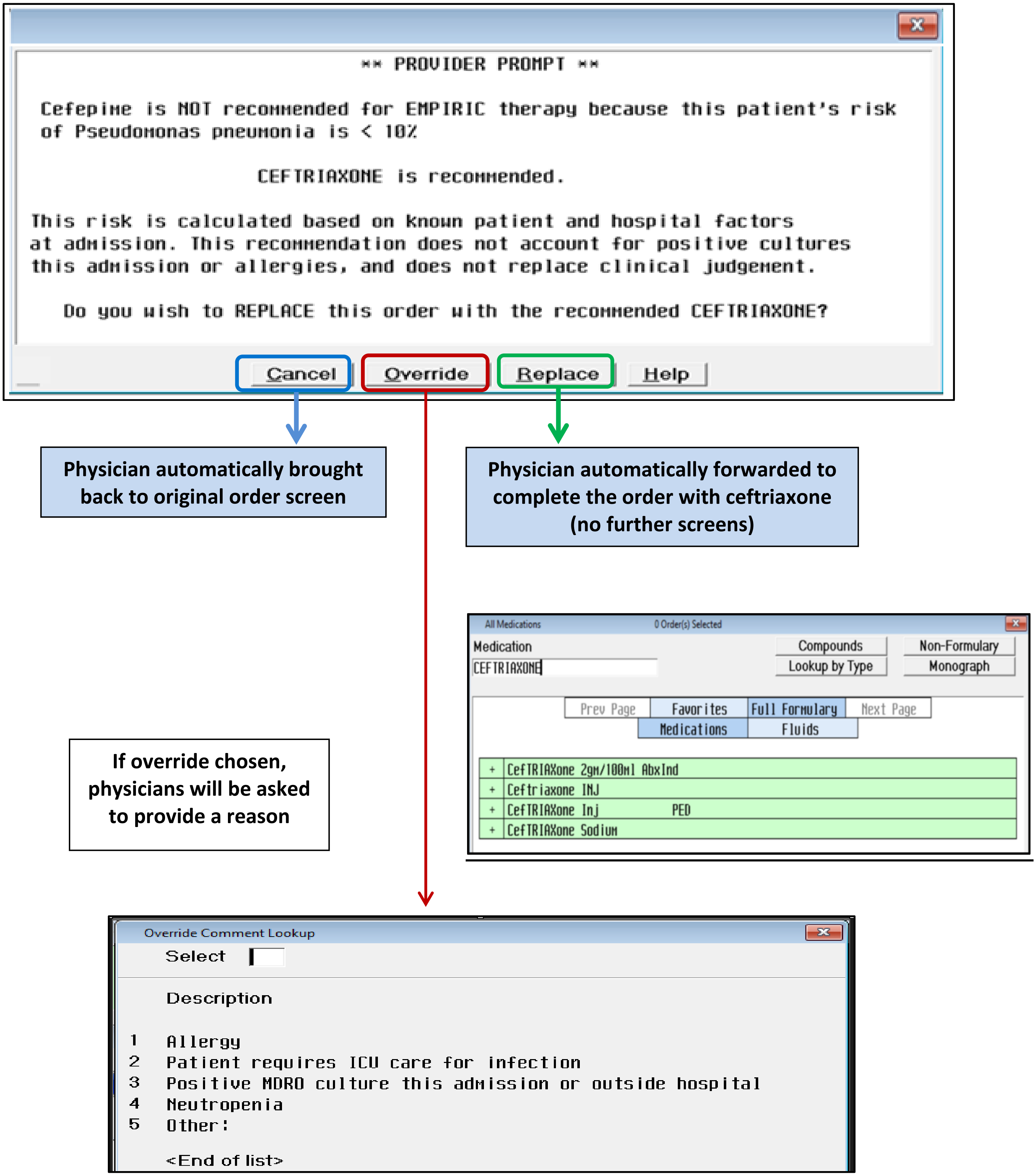

INSPIRE-ASP Quarterly Progress Reports

Regular monitoring and provider feedback are integral components of successful antibiotic stewardship interventions. The investigative team will partner with HCA Healthcare to provide study champions with quarterly clinician and hospital progress reports on a monthly basis. The goal of these progress reports is to:

- Help study champions identify whether additional education or follow-up is needed
- Help clinicians and hospitals track their overall progress

This information will be displayed in table and graph form and will contain a rolling quarter's worth of data and will include how often clinicians order extended-spectrum antibiotics for pneumonia or UTI, how often recommendations for standard antibiotics is accepted, and if overridden, what the chosen reason was for overriding. See below for examples of the tables and graphs that will be provided.

All data will be collected via the centralized HCA Clinical Data Warehouse.

Table 1: Quarterly Clinician Progress Reports - Example

Clinician	Total PNA Non-ICU Admissions N	Empiric Antibiotics Ordered Per Protocol N (%)	Smart Prompts Triggered N (%)	Smart Prompt Declined/ Overridden N (%)	Adherence with Smart Prompt Recommendation N (%)
**You**	12	8 (67)	4 (33)	3 (75)	9 (75)
**MD 1**	10	5 (50)	5 (50)	2 (40)	8 (80)
**MD 2**	9	3 (33)	6 (67)	4 (67)	5 (56)
**MD 3**	7	4 (57)	3 (43)	1 (33)	6 (86)
**MD 4**	3	2 (67)	1 (33)	1 (100)	2 (67)
**All Clinicians This Hospital**	41	22 (54)	19 (46)	11 (58)	30 (73)
**All Clinicians Arm 2 Trial**	1189	638 (54)	551 (46)	319 (58)	870 (73)

Table 2: Quarterly Clinician Overrides and Declinations - Example

Clinician	Reason for Declining or Overriding Smart Prompt				
	Allergy N (%)	Neutropenia N (%)	MDRO Culture Positive N (%)	ICU Bound N (%)	Other N (%)
You	1 (33)	0 (0)	1 (33)	1 (33)	0 (0)
MD 1	0 (0)	1 (50)	0 (0)	1 (50)	0 (0)
MD 2	1 (25)	1 (25)	0 (0)	1 (25)	1 (25)
MD 3	0 ()	0 (0)	1 (100)	0 (0)	0 (0)
MD 4	1 (100)	0 (0)	0 (0)	0 (0)	0 (0)
All Clinicians This Hospital	3 (27)	2 (18)	2 (18)	3 (27)	1 (9)
All Clinicians Arm 2 Trial	87 (27)	59 (18)	58 (18)	86 (27)	29 (9)

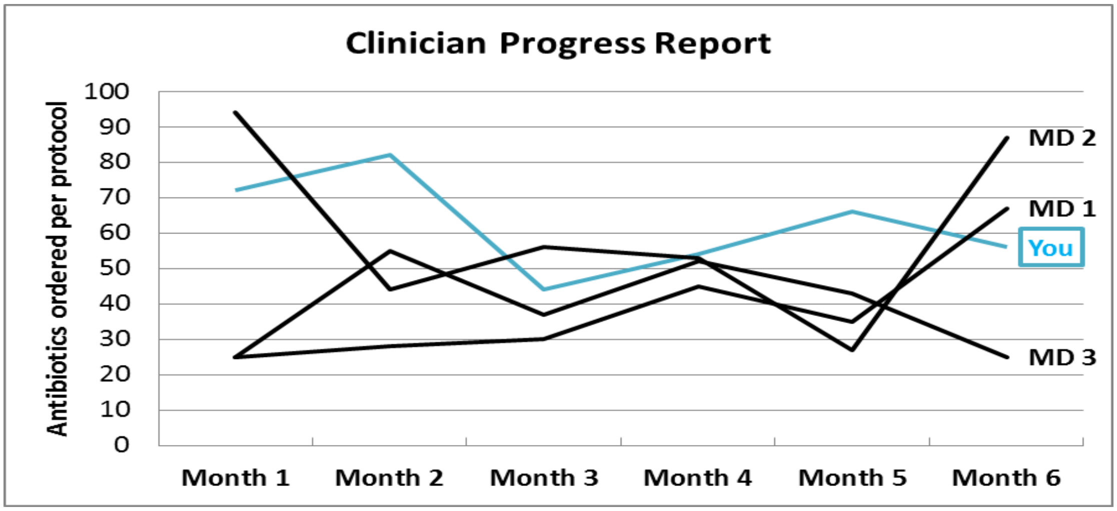

Figure 1: Quarterly Clinician Progress Report – Example

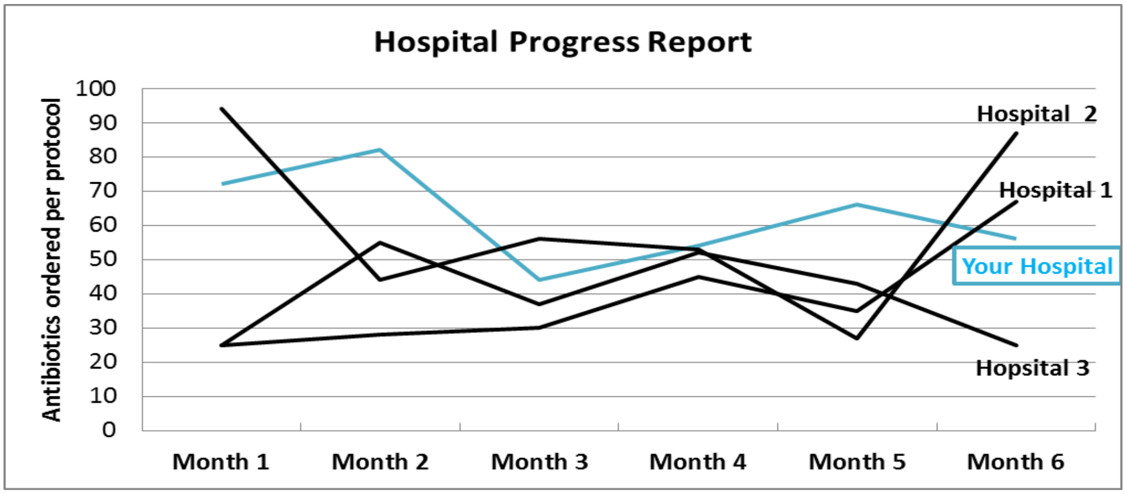

Figure 2: Quarterly Hospital Progress Report - Example

INSPIRE-ASP Trial

INtelligent Stewardship Prompts to Improve Real-time Empiric Antibiotic Selection for Patients

November 4, 2019

Agenda

- High ESBL UTI Prevalence and Extended-Spectrum Use
- Asymptomatic Bacteriuria
- The Case for Empiric Standard Spectrum Use in UTI
- Alternatives to Carbapenems
- How INSPIRE Smart Prompts Help Physicians Choose Wisely
- Question & Answer

Association Between High ESBL UTI Prevalence and Extended Spectrum Use

The Race to Antibiotic Resistance

Antibiotic Introduced to US Market

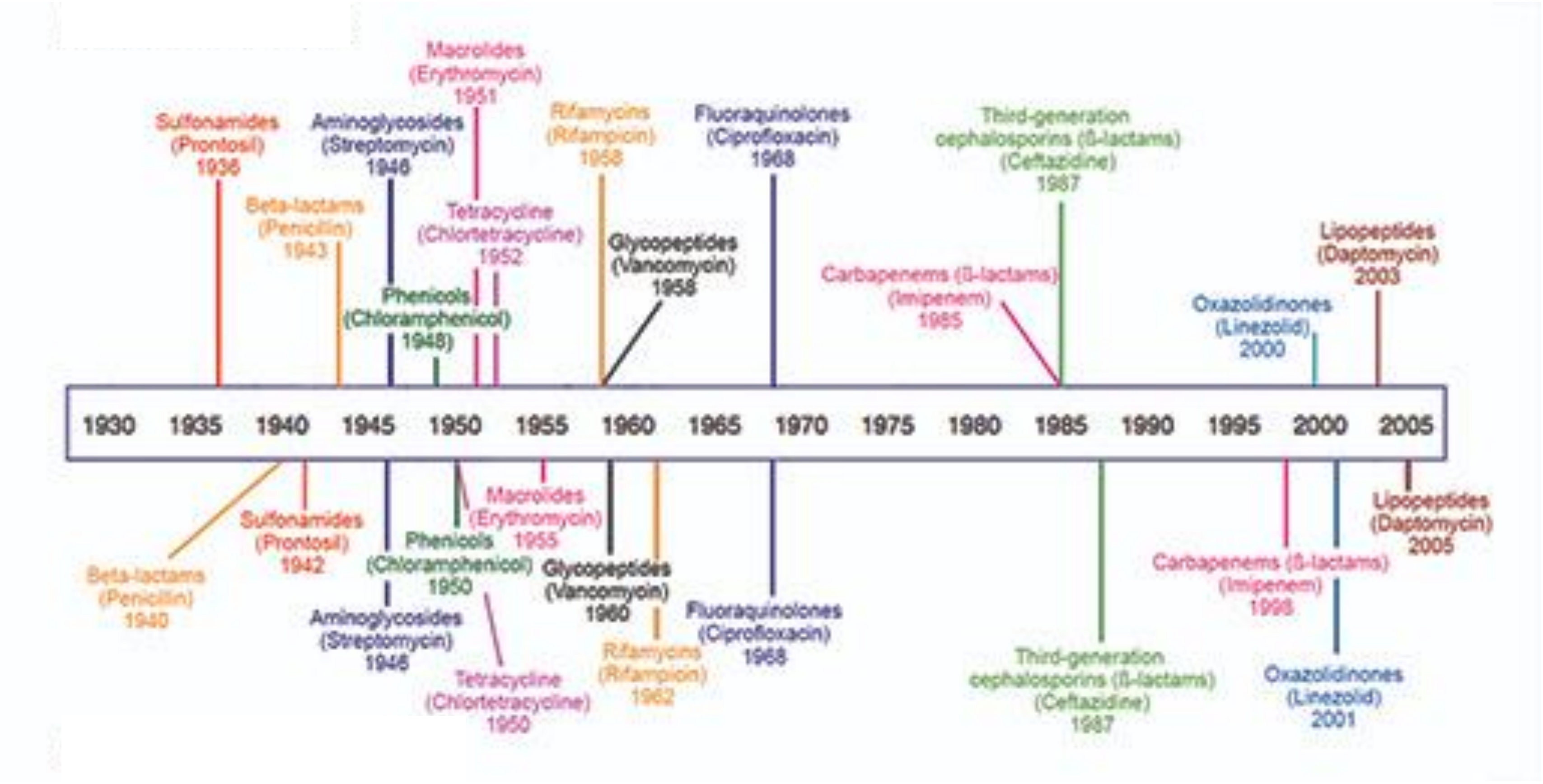

Antibiotic Resistance Detected

ESBL UTI – High Prevalence INSPIRE Facilities

- Among >300,000 HCA patients, mean percent ESBL UTI – 5.7%

Facility	% ESBL Among UTI
Valley Regional Medical Center	12.4%
Riverside Community Hospital	10.1%
Regional Medical Center of San Jose	9.5%
Kendall Regional Medical Center	9.0%
TriStar Stonecrest Medical Center	8.7%
Putnam Community Hospital	7.9%

HCA Data Warehouse - 2014-2017

INSPIRE
BETTER SAFER ANTIBIOTIC USE

Extended Spectrum Use Among High ESBL UTI Facilities

- Facilities with high ESBL UTI also have the highest antibiotic use

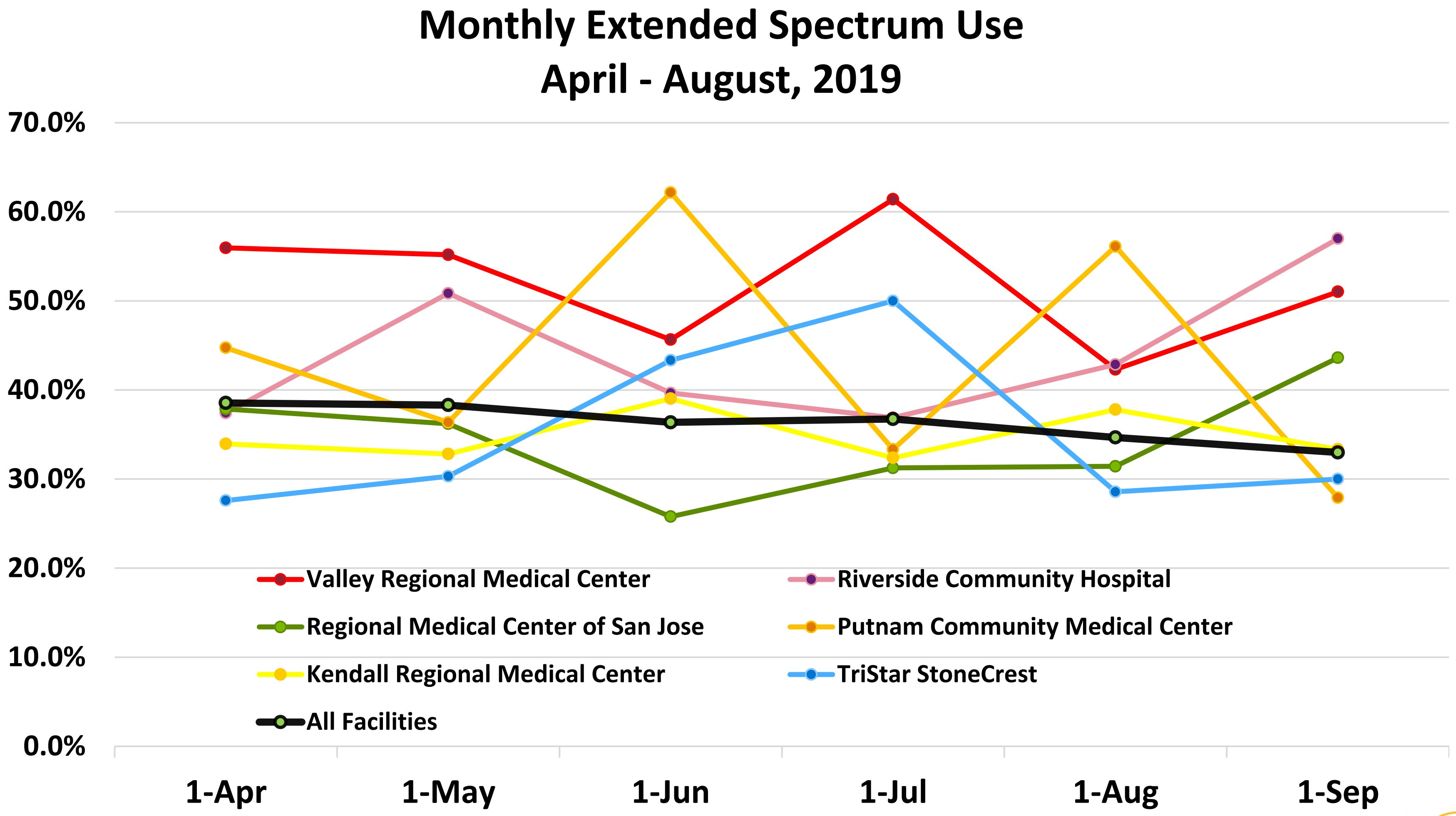

Asymptomatic Bacteriuria

Asymptomatic Bacteriuria and Antibiotic Overuse

- **Almost 40% of hospitalized UTI patients receive overly broad or unnecessary antibiotics**
  - Overuse tied to asymptomatic bacteriuria (ASB) in women, elderly, and patients with neurologic impairment
  - No need to treat at all if culture growth < 100,000
- INSPIRE data: Extended spectrum not needed in vast majority
  - HCA UTI patients – 3.4% *Pseudomonas*, 5.8% ESBL

1. HICPAC. Antibiotic Stewardship Statement for Antibiotic Guidelines – The Recommendations of the Healthcare Infection Control Practices Advisory Committee (HICPAC). 2016
2. Fridkin S, Baggs J, Fagan R, Magill S, Pollack LA, Malpiedi P, Slayton R, Khader K, Rubin MA, Jones M, Samore MH, Dumyati G, Dodds-Ashley E, Meek J, Yousey-Hindes K, Jernigan J, Shehab N, Herrera R, McDonald CL, Schneider A, Srinivasan A, Centers for Disease C, Prevention. Vital signs: improving antibiotic use among hospitalized patients. MMWR Morb Mortal Wkly Rep 2014;63:194-200
3. Nicolle LE, Gupta K, Bradley SF, Colgan R, DeMuri GP, Drekonja D, Eckert LO, Geerlings SE, Koves B, Hooton TM, Juthani-Mehta M, Knight SL, Saint S, Schaeffer AJ, Trautner B, Wullt B, Siemieniuk R. Clinical Practice Guideline for the Management of Asymptomatic Bacteriuria: 2019 Update by the Infectious Diseases Society of Americaa. Clin Infect Dis 2019

Asymptomatic Bacteriuria in Chronically Instrumented Patients

- Bacteriuria is common in patients with chronic catheters
- Biofilm develops within 72 hours of catheter placement
  - Increases risk for resistant organisms
- Among nursing home residents, over 80% of antibiotics in catheterized patients were for individuals with ASB[1]
- Retrospective study of immobile patients with chronic catheters in whom urine cultures were sent (n = 405)[2]
  - Leukocyturia in 77%, nitrite positive 69%
  - Gram negative bacteria 89% → **ESBL positivity – 47%**

ESBL positive urine cultures should be clinically assessed for appropriateness of treatment

1. Phillips, CD et al. BMC Geriatr. 2012 Nov 23;12:73.
2. Albu, S et al. Medicine: August 2018 - Volume 97 - Issue 33

The Case for Empiric Standard Spectrum Treatment of UTI

UTI Empiric Treatment – There is Time to Await Cultures

- IDSA 2016 Cystitis, Pyelonephritis Guidelines
  - Standard spectrum – no need for anti-pseudomonal coverage
- Emerging data: empiric extended spectrum antibiotics do NOT improve outcomes in patients with complicated UTI
- **Using standard spectrum while awaiting cultures does not negatively impact mortality, clinical cure, length of stay, or adverse events**
  - Includes ESBL pyelonephritis treated with non-ESBL antibiotics
  - Includes bacteremia - no adverse impact on mortality in non-neutropenic patients

[1]HICPAC. Antibiotic Stewardship Statement for Antibiotic Guidelines – The Recommendations of the Healthcare Infection Control Practices Advisory Committee (HICPAC). 2016
[2]Eliakim-Raz N, Babitch T, Shaw E, Addy I, Wiegand I, Vank C, Torre-Vallejo L, Joan-Miquel V, Steve M, Grier S, Stoddart M, Nienke C, Leo VDH, Vuong C, MacGowan A, Carratala J, Leibovici L, Pujol M, Group RS. Risk Factors for Treatment Failure and Mortality Among Hospitalized Patients With Complicated Urinary Tract Infection: A Multicenter Retrospective Cohort Study (RESCUING Study Group). Clin Infect Dis 2019;68:29-36
[3]Kim SH, Oh S, Huh K, Cho SY, Kang CI, Chung DR, Peck KR. Inappropriate empirical antibiotic therapy does not adversely affect the clinical outcomes of patients with acute pyelonephritis caused by extended-spectrum beta-lactamase-producing Enterobacteriales. Eur J Clin Microbiol Infect Dis 2019;38:937-44.
[4]Lin MY, Weinstein RA, Hota B. Delay of active antimicrobial therapy and mortality among patients with bacteremia: impact of severe neutropenia. Antimicrob Agents Chemother 2008;52:3188-94

Empiric Treatment and Survival in Complicated UTI

- RESCUING Study Group: Multinational, multi-center retrospective cohort study
- Complicated UTI patients (n=981)
- Outcomes
  - Treatment failure
  - 30-day mortality
- Evaluated management variables:
  - "Appropriate empiric antibiotics" - matching pathogen in blood or urine
  - Days to starting antibiotics

Eliakim-Raz N, Babitch T, Shaw E, Addy I, Wiegand I, Vank C, Torre-Vallejo L, Joan-Miquel V, Steve M, Grier S, Stoddart M, Nienke C, Leo VDH, Vuong C, MacGowan A, Carratala J, Leibovici L, Pujol M, Group RS. Risk Factors for Treatment Failure and Mortality Among Hospitalized Patients With Complicated Urinary Tract Infection: A Multicenter Retrospective Cohort Study (RESCUING Study Group). Clin Infect Dis 2019;68:29-36

UTI Treatment – There Is Time To Await Culture

- Treatment with inappropriate empiric antibiotics not associated with treatment failure or mortality

Table 2. Multivariable Analysis for Treatment Failure

Risk Factor	Multivariable Generalized Estimating Equation, OR (95% CI)	*P* Value
Metastatic cancer	2.89 (1.46–5.73)	.02
Septic shock	1.92 (.93–3.98)	.079
Infection source: indwelling catheter	1.48 (1.04–2.11)	.028
Appropriate treatment[a]	0.81 (.57–1.15)	.246
Duration of symptoms before hospitalization	0.99 (.97–1.02)	.656
Corticosteroid therapy	1.92 (1.12–3.54)	.018
Functional capacity: bedridden	2.11 (1.4–3.18)	.000
Age (per 1 y)	1.02 (1.007–1.03)	.001
ICU admission	5.07 (3.18–8.07)	.000

Appropriate treatment defined as antibiotics matching the isolated pathogen in blood or urine

Eliakim-Raz N, Babitch T, Shaw E, Addy I, Wiegand I, Vank C, Torre-Vallejo L, Joan-Miquel V, Steve M, Grier S, Stoddart M, Nienke C, Leo VDH, Vuong C, MacGowan A, Carratala J, Leibovici L, Pujol M, Group RS. Risk Factors for Treatment Failure and Mortality Among Hospitalized Patients With Complicated Urinary Tract Infection: A Multicenter Retrospective Cohort Study (RESCUING Study Group). Clin Infect Dis 2019;68:29-36

INSPIRE BETTER SAFER ANTIBIOTIC USE

Impact of Delay in Antibiotics for ESBL Pyelonephritis

- Retrospective cohort study, tertiary care hospital
- ESBL positive culture (blood or urine), n=1185
- Evaluated patients receiving inappropriate antibiotics for 48hrs or longer before receiving culture-directed treatment
- Outcome – clinical or microbiologic failure, LOS, recurrence (2 months)
- Asymptomatic bacteriuria found in 60% (718)
- Among patients with acute pyelonephritis (175)
  - Inappropriate empiric regimen given in 34% (59)
  - Treatment failure found in 8% (14)

Kim, et al. Inappropriate empirical antibiotic therapy does not adversely affect the clinical outcomes of patients with acute pyelonephritis caused by extended-spectrum β-lactamase-producing Enterobacteriales. European Journal of Clinical Microbiology & Infectious Diseases (2019) 38:937–944

Impact of Delay in Antibiotics for ESBL Pyelonephritis

- No difference in treatment failure, clinical or microbiologic cure, adverse event, or LOS

Table 3 Comparison of clinical outcomes between appropriate and inappropriate empirical treatment groups

	Non-matched					Adjusted (PS weight)				
	Event		OR	95% CI	*P* value	Event		OR	95% CI	*P* value
	AT (116)	IAT (59)				AT (101)	IAT (47)			
Primary outcome										
Treatment failure	9 (7.8)	5 (8.5)	1.10	0.35–3.47	0.870	8.2 (8.2)	4.0 (8.5)	1.05	0.26–4.15	0.947
Secondary outcome										
Clinical cure	112 (96.6)	58 (98.3)	2.07	0.22–19.20	0.522	98.1 (97.2)	46.0 (97.9)	1.34	0.14–13.06	0.799
Microbiological cure	109 (94.0)	54 (91.5)	0.69	0.21–2.30	0.550	94.2 (93.3)	43.0 (91.5)	0.78	0.18–3.31	0.732
Adverse events during treatment	18 (15.5)[a]	3 (5.1)[b]	0.29	0.08-1.04	0.058	15.4 (15.2)	3.0 (6.4)	0.38	0.11–1.30	0.379
Length of hospital stay (Median, IQR) (days)	11.5 (10–14)	10 (7–14)			0.124	11 (10–14)	11 (7–14)			0.717

Kim, et al. Inappropriate empirical antibiotic therapy does not adversely affect the clinical outcomes of patients with acute pyelonephritis caused by extended-spectrum β-lactamase-producing Enterobacteriales. European Journal of Clinical Microbiology & Infectious Diseases (2019) 38:937–944

ESBL UTI Treatment

Alternatives to Carbapenems

Sparing Carbapenem Use in ESBL Urinary Tract

- Carbapenems widely regarded first choice treatment of ESBL
- Use of carbapenem contributes to emergence of CRE
- Several carbapenem-sparing options available for UTI
  - Capitalize on ability to reach high renal concentration
  - Data is emerging

Nitrofurantoin and Fosfomycin for ESBL Cystitis

- Recommended as first line options for treatment of cystitis[1]
- Therapeutic concentration only achieved in lower urinary tract
- Reserve for uncomplicated lower tract UTIs e.g. cystitis[2]
- Resistance uncommon – most MDR *E coli* still susceptible
- Oral administration only

1. Clin Infect Dis 2011; 52:e103-e120
2. J Antimicrob Chemother 2015; 70:2456-2464

Nitrofurantoin and Fosfomycin Administration

- Fosfomycin administered in 3 gram single-dose (repeat 48 to 72 hours if symptomatic)
  - Expensive
  - Intravenous not available in US

- Nitrofurantoin contraindications
  - Not recommended in pregnancy beyond 38 weeks
  - Cannot use for CrCl <30 mL/min
  - Use cautiously between 30-60 mL/min

Cephamycins

- ESBLs cannot hydrolyze cephamycins (cefoxitin and cefotetan)
- In vitro activity preserved
- Intravenous therapeutic option for pyelonephritis
- Not active against AmpC producers
- Two recent reviews concluded cephamycins may be alternative to carbapenems for non-severe UTI/pyelonephritis[1,2,3]
- Recent study evaluated 31 patients treated with cefoxitin for ESBL *E coli* and *K pneumonia*[4]
  - Reported > 90% cure at day 30 and 83.3% at day 90
  - Cefoxitin dose used was 1 gram q6h.
  - One patient relapsed

1. Clin Infect Dis 2017; 64:972-980
2. Clin Microbiol Rev 2018; 31:1-42
3. Eur J Clin Microbiol Infect Dis. 2019 Sep 11
4. Ther Adv Infectious Dis 2019; 6:1-7

INSPIRE BETTER SAFER ANTIBIOTIC USE

Cefoxitin Intravenous Treatment

- Cefoxitin compared to carbapenem for ESBL *E. coli* UTI[1]
  - Study 23 patients treated with cefoxitin, 27 carbapenem
  - **<u>No significant difference</u>** in clinical (OR = 0.90, CI 0.12-6.70) or microbiological (OR = 0.85, CI 0.05; 14.00) success
  - High-dose (8 gm/day) and continuous infusion associated with clinical success
- Prolonged or continuous infusion increases probability of achieving pharmacokinetic/pharmacodynamic targets for cefoxitin for ESBL-*E coli* UTI treatment[2]

1. Eur J Clin Microb Infect Dis published online September 11, 2019
2. Antimicrob Agents Chemother 2014; 58:4899-4902

Cephamycin Dosing

- Most labs do not due routinely do susceptibility testing to cefoxitin, but studies show ~90% activity against ESBL+ *E coli*
- Prolonged (over 4-hours) or continuous (over 24-hours) infusions recommended for isolates with higher MIC (4-8 µg/mL)

Test/Report Group	Antimicrobial Agent	Disk Content	Interpretive Categories and Zone Diameter Breakpoints, nearest whole mm				Interpretive Categories and MIC Breakpoints, µg/mL				Comments
			S	SDD	I	R	S	SDD	I	R	
B	Cefoxitin	30 µg	≥ 18	-	15-17	≤ 14	≤ 8	-	16	≥ 32	**(18)** Breakpoints are based on a dosage regimen of at least 8 g per day (eg, 2 g **administered** every 6 h).

CLSI M100-ED29:2019 Performance Standards for Antimicrobial Susceptibility Testing

Cefoxitin Dose Optimization for ESBLs

- Continuous infusions > prolonged infusions >> intermittent

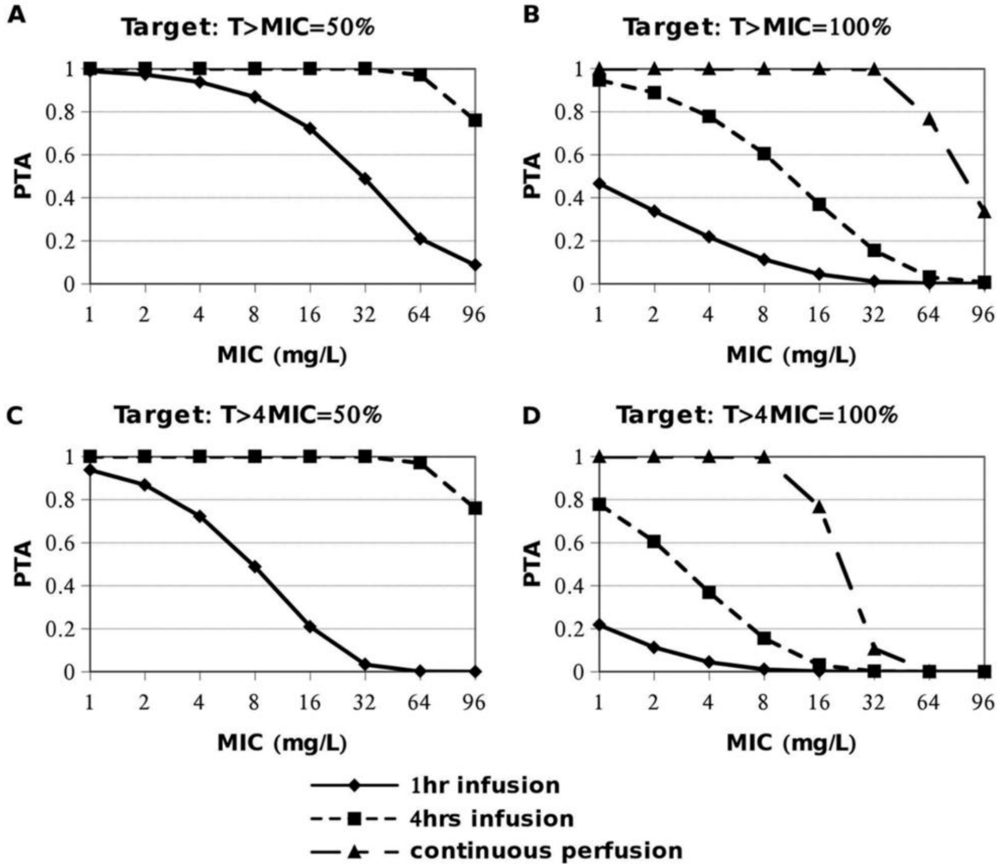

FIG 2 Probability of target attainment (PTA), depending on the MIC, for a dosage of 2 g 4 times/day and the following targets: T>MIC = 50% (A), T>MIC = 100% (B), T>4MIC = 50% (C), and T>4MIC = 100% (D).

Antimicrob Agents Chemother 2014; 58:4899-901

Other Carbapenem-Sparing Options for ESBL UTIs

- Trimethoprim/sulfamethoxazole and fluoroquinolones
  - Up to 30% of ESBL-producing pathogens may be susceptible[2,3]
  - If susceptible, either drug reasonable
  - Should not be used for empiric treatment if known ESBL
- Aminoglycosides
  - Reasonable alternative in certain cases
  - Can lead to renal or cochlear-vestibular toxicity

1. Med Mal Infect 2018; 48:327-358 (European UTI guidelines)
2. Antimicrob Agents Chemother. 2010 Sep; 54(9): 4006–4008
3. J Microbiol Immunol Infect. 2011 Oct;44(5):364-8.

How INSPIRE ESBL UTI Risk Estimates Can Help Clinicians Choose Wisely

What Variables Were Assessed?

- 536,000 patients admitted to HCA Healthcare hospitals, 2014-2016
- We evaluated:

  (1) Your hospital antibiogram for pneumonia and UTI patients

  (2) Risk factors of MDRO infection
  - Demographics
  - Comorbidities
  - MDRO or *Pseudomonas* history
  - Recent admission, ED visit, nursing home care
  - Recent antibiotic use
  - Labs (e.g., BUN, creatinine)

Factors predictive of MDRO infection were programmed into Meditech to provide a patient-specific risk estimate for each MDRO.

ESBL UTI Risk Estimate Model

Rules	N	%Predicted with ESBL UTI
ESBL Hx, No CRE Hx, No PSE Hx, % Fac ESBL UTI ≥5.0%	4932	35%
ESBL Hx, No CRE Hx, No PSE Hx, % Fac ESBL UTI <5.0%, No VRE Hx	2079	29%
ESBL Hx, CRE Hx, % Fac ESBL UTI ≥5.0%, No Medicaid, PSE Hx	303	25%
ESBL Hx, No CRE Hx, PSE Hx	1317	25%
ESBL Hx, CRE Hx, % Fac ESBL UTI ≥5.0%, Medicaid	1263	24%
ESBL Hx, CRE Hx, % Fac ESBL UTI ≥5.0%, No Medicaid, No PSE Hx	1368	18%
No ESBL Hx, % Fac ESBL UTI ≥5.0%, Medicaid, BUN <20, Male, VRE Hx	53	17%
ESBL Hx, CRE Hx, % Fac ESBL UTI <5.0%	1518	14%
ESBL Hx, No CRE Hx, No PSE Hx, % Fac ESBL UTI <5.0%, VRE Hx	134	14%
No ESBL Hx, % Fac ESBL UTI ≥5.0%, Medicaid, BUN ≥20	18657	8%
No ESBL Hx, % Fac ESBL UTI ≥5.0%, No Medicaid, BUN ≥20, PSE Hx	1341	8%
No ESBL Hx, % Fac ESBL UTI ≥5.0%, Medicaid, BUN<20, Male, No VRE Hx	4728	7%
All orders not meeting above	206084	<=5%

HCA baseline data 2014-2017
Urinary tract infection (UTI), n = 304,584
"PSE"= *Pseudomonas*, "Hx" = history, "MDR"=multidrug resistant
"ESBL"= extended-spectrum beta lactamase.

INSPIRE smart prompt only fires for low risk patients

ESBL UTI Smart Prompt

- Robust modeling approach to guide **empiric** coverage
- Identifies low risk patients in whom carbapenem can be spared
- Clinical judgment paramount
  - Does not capture cultures outside hospital
  - History of ESBL capture – up to 24 hours prior to admission

Physician can use INSPIRE smart prompt to identify patients in whom alternatives to extended spectrum can be considered

Supplement 1
INSPIRE Urinary Tract Infection Trial
Statistical Plan Amendments

**Date**	**Statistical Plan Amendments**
**9/19/2018**	**Original**
**8/24/2019**	Added language describing that safety outcome analysis will evaluate non-inferiority with a one-tailed significance set at alpha = 0.05 (to increase conservatism).
**6/3/2020**	**Final**<br>• Refined analytic model to include multiple admissions per patient.<br>• Updated language in response to Harrington et al (NEJM 2019; 381:285-6) publication on "New Guidelines for Statistical Reporting in the Journal."<br>• Clarified two-tailed significance for secondary outcomes to be set at alpha = 0.025 to account for multiple comparisons<br>• Improved layout for analytic plan and grammar<br>• Converted outcome tables from a single table to three distinct tables (content same as original)<br>*(All updates were created prior to accessing trial dataset for cleaning and analysis.)*<br><br>**Note**: Upon attempting trial analysis, we encountered limitations in hardware and software that prevented implementing this theoretically possible analysis; we then selected a single random admission per patient.

(Originally published in Gohil, et al. Stewardship Prompts to Improve Antibiotic Selection for Urinary Tract Infection: The INSPIRE Randomized Clinical Trial. JAMA. 2024;331(23):2018–2028. doi:10.1001/jama.2024.6259)

INSPIRE-ASP Urinary Tract Infection Trial Statistical Analysis Plan

September 19, 2018

Trial Outcomes

INSPIRE-ASP Urinary Tract Infection (UTI) Trial Outcomes

The goal of this trial is to reduce unnecessary physician prescribing of empiric extended-spectrum antibiotics for UTI requiring hospitalization. Trial outcomes are outlined in the table below and will be applied to the study population of adult patients who have a primary or secondary claims code indicating UTI that is present on admission, who did not require direct admission from the ED to ICU and was treated with an antibiotic within 3 days of admission. Our primary outcome will be days of empiric extended-spectrum (ES) antibiotic treatment (days of therapy, DOT) per 1,000 at-risk-days. Days of therapy (DOT) is specific to each extended-spectrum antibiotic, where any dose is considered one day's worth of therapy. The ES antibiotics are then aggregated to obtain the total ES DOT. For example, if a patient is started on vancomycin and cefepime for 1 day, this will count as 2 ES DOT for that day. Multiple doses of the same antibiotic received on the same day are only counted as one day of therapy for that extended spectrum antibiotic. The empiric period is defined as within 3 calendar days of admission. This outcome will include antibiotics in Table 3.

Table 1: Primary and Secondary INSPIRE-ASP UTI Trial Outcomes

Outcome	Metric
**Primary Manuscript**	
**Primary Outcome**	
Empiric Extended-Spectrum (ES) Antibiotic Use	Days of empiric ES antibacterial treatment for UTI per at-risk day, (first 3 days of admission)[1].
**Secondary Outcomes**	
Empiric Vancomycin Use	Days of empiric Vancomycin treatment for UTI per at-risk-day (first 3 days of admission)[1]
Empiric Antipseudomonal Use	Days of empiric antipseudomonal antibacterial treatment for UTI per at-risk-day (first 3 days of admission)[1,2]
**Safety Outcomes for Trial (Other Pre-specified Outcomes)**	
Antibacterial Escalation	Days from start of standard-spectrum antibacterial for UTI until switch to extended-spectrum antibacterial for UTI during hospital stay
ICU Transfers	Days from start of hospitalization until ICU transfer within hospital stay
Length of Stay	Hospital length of stay

[1]For extended-spectrum and antipseudomonal days of therapy (DOT), if more than one different type of antibiotic is given on the same day, then each contributes separately to the DOT.
[2]Does not include aminoglycosides or fluoroquinolones.

Primary Statistical Analysis

The main trial results will be based upon as-randomized, unadjusted analyses of empiric extended-spectrum (ES) antibiotic days of therapy (DOT) per at-risk day. In contrast to the national DOT measure defined by CDC for hospitals, we define DOT at the individual level so that we can perform analyses on individuals. We define a DOT for a particular ES antibiotic as a day in which any number of doses of that antibiotic is given. These are summed across at-risk days and ES antibiotics within patient, then divided by the at-risk days within that patient to

determine each patient's DOT per at-risk day. At-risk days are in the first three days of admission.

The trial will be assessed among the cohort of adult patients who have a primary or secondary claims code indicating UTI that is present on admission, who did not require direct admission from the ED to ICU and was treated with an antibiotic within 3 days of admission.

The primary outcome for the trial is ES DOT per at-risk day. Ordinarily, we would perform a Poisson or negative binomial analysis on the count of ES DOT, with an offset of the number of days over which these antibiotics were received. However, the standard metric is the DOT per day, and analysis of this outcome with no offset is equivalent to the count of days of therapy with an offset of the number of days. In analyses of the baseline data, performed before randomization, there was no hint of overdispersion in a Poisson model, and so we plan to use that model, rather than the negative binomial model, in the data analysis.

The planned model for the project is a generalized linear mixed effects model for differences in differences, with random effects accounting for correlation within cluster and period-varying random effects to allow for differences between hospitals from baseline to follow-up. The model can be expressed as follows:

$$log(y_{iph}) = \beta_0 + \beta_1 A_{iph} + \beta_2 T_{iph} + \beta_3 A_{iph} T_{iph} + b_{0h} + b_{1h} T_{iph}$$

where y_{iph} is the DOT per at-risk day for person i in period p at hospital h, and $A_{iph} = 1$ if hospital h is in the intervention arm and 0 if not, and $T_{iph} = 1$ if p is the intervention period and 0 if baseline period. The random effects b_{0h} and b_{1h} allow for different baseline mean ES DOT per at-risk day for each hospital and each hospital per period, respectively. Equivalently, they allow for correlation within hospital at different levels at baseline and at follow-up.

The assessment of trial success will be determined by the significance of the arm by period interaction term β_3, which assesses whether the log relative rate of the outcome due to being in the intervention arm in the intervention period is different from 0. The exponentiated parameter estimate for β_3 is the estimated relative rate of DOT per at-risk day due to the intervention, relative to the baseline period. For example, if β_3 had a negative value and a p-value <0.05, we would conclude that the physician-specific CPOE smart prompts generated a benefit over routine care. Exponentiating the parameter value would give us an estimate of the relative reduction due to the intervention in the expected DOT per at-risk day.

Subsequent analyses will include both as-treated and adjusted models. Adjusted models will account for individual characteristics such as age, gender, comorbidities, and prior history of MDROs as well as hospital characteristics such as hospital antibiotic resistance. We will also account for randomization strata. All analyses will be performed using current versions of SAS (Version 9.4, as of writing, SAS Institute, Cary NC) and/or R (Version 3.3.0, as of writing).

Analysis plan amended with the following paragraph on 8/24/19:

Safety outcomes noted in the above table will be assessed in the most conservative manner to identify potential safety issues. Each safety outcome will be evaluated for non-inferiority using an as-randomized unadjusted model with a one-tailed significance set at alpha = 0.05.

Power Size/Sample Size Calculations

Power assessment proceeded as follows, using a Monte Carlo approach. We used data from the baseline period of 10/1/2015-12/31/2016 in a bootstrap procedure. Individuals were selected with replacement from within each hospital, once to represent baseline data and separately to represent intervention period data. In the randomization portion of the Monte Carlo approach, we ordered the hospitals by "size", meaning the number of patients with UTI. Then, within strata of 6 hospitals, we ordered by DOT per at-risk day. Then we took pairs of hospitals

with adjacent DOT per at-risk day and assigned one member of the pair to one arm. To do this, a single pseudo-random number uniformly distributed between 0 and 1 was generated for each pair. If it was less than 0.5, the arbitrary “first” member of the pair was assigned to Routine Care and the other to Intervention. If it was greater than or equal to 0.5, then the assignments were reversed. The remaining unpaired hospital of the 59 participants was assigned as the “first” member of a pair with no match.

If a patient was in the intervention arm in the intervention period, then their DOT per at-risk day was reduced by a proportion, given below. The reduction of ES DOT per at-risk day was selected with a lower bound determined by the investigators as the hoped-for effect of the intervention with minimal clinical significance, and the impact in the Monte Carlo calculation on a per-patient basis was to reduce the days of therapy proportionately between this number and 1, on a uniform distribution. In other words, if the effect of the intervention was to multiply doses by as little as .9, each person would have their doses multiplied by between .9 and 1. Then an integer value of doses would be chosen as described above.

We are interested in determining whether the patient-specific CPOE intervention reduces the average days of empiric extended-spectrum antibiotics by at least 12.5% relative to usual care. Using the method above, we have 97% power (CI 91-99%) to detect this effect. Table 2 below shows the calculated power and confidence intervals for the primary and secondary outcomes to be evaluated in our primary manuscript.

Table 2. Power Calculation – Primary and Secondary Outcomes

**Primary Trial Outcome**	**Power (CI), %**
Days of empiric ES antibiotic treatment per at-risk day, (within 3 days of admission).	97 (91-99)
**Secondary Trial Outcomes**	
Days of empiric Vancomycin treatment per at-risk-day (first 3 days of admission)	92 (85-96)
Days of empiric anti-Pseudomonas antibiotic treatment per at-risk-day (first 3 days of admission)	84 (77-91)

Extended-spectrum antibiotics are defined in the study protocol and provided below for convenience.

Table 3: INSPIRE-ASP UTI Extended-Spectrum Antibiotics

**Pathogen-Directed Antibiotic Category**	**Extended-Spectrum Antibiotics Targeted in INSPIRE UTI CPOE Prompts**
**Anti-MRSA**	Ceftaroline, Daptomycin, Linezolid[1], Vancomycin[2]
**Anti-VRE**	Daptomycin, Linezolid
**Anti-*Pseudomonas***	Aztreonam, Cefepime, Ceftazidime, Piperacillin/Tazobactam
**Anti-ESBL**	Ertapenem, Ceftolozane/Tazobactam, Meropenem, Imipenem
**Anti-CRE**	Ceftazidime/Avibactam, Colistin, Imipenem/Relebactam, Meropenem/Vaborbactam, Polymixin B, Tigecycline

[1]Both oral and intravenous (IV) formulations, [2]IV formulation only.

Abbreviations: **CPOE** – Computerized Physician Order Entry, **MRSA -** Methicillin Resistant *Staphylococcus aureus,* **ESBL –** Extended Spectrum Beta Lactamase Producing *Enterobacterales,* **VRE –** Vancomycin Resistant *Enterococci*, **CRE –** Carbapenem Resistant *Enterobacterales.*

(Originally published in Gohil, et al. Stewardship Prompts to Improve Antibiotic Selection for Urinary Tract Infection: The INSPIRE Randomized Clinical Trial. JAMA. 2024;331(23):2018–2028. doi:10.1001/jama.2024.6259)

INSPIRE-ASP Urinary Tract Infection Trial
Statistical Analysis Plan

June 3, 2020

Background

The **INSPIRE-ASP Urinary Tract Infection (UTI) Trial** (INtelligent Stewardship Prompts to Improve Real-time Empiric Antibiotic Selection for Patients with UTI) is a cluster-randomized trial of HCA Healthcare (HCA) affiliated hospitals to assess best practice in antibiotic stewardship for adult hospitalized patients admitted to non-critical care units with UTI. It compares routine care to the use of a real-time smart prompt that provides a patient's specific risk of antibiotic-resistant UTI and alerts the clinician during the antibiotic ordering process to optimize prescribing and ideally improve patient outcomes. The main goal is to see if there is a difference in the empiric antibiotic prescribing practices for extended-spectrum antibiotics (ES). We define antibiotics administered during the first three days of hospitalization as empiric treatment because infecting pathogens and their antimicrobial susceptibilities are typically not known during this period. We refer to these first three days as the empiric period.

Participating hospitals are randomized to:

- **Arm 1 – Routine Care**
- **Arm 2 – Real-Time Risk Estimation Smart Prompt**
  Precision medicine smart prompt using a computerized physician order entry (CPOE) alert that gives a patient-specific risk estimate of antibiotic resistant infection and recommends standard-spectrum antibiotics in patients admitted for UTI who are found to have low risk (<10%) for recovery of a drug resistant pathogen.

Trial Outcomes

Trial outcomes evaluating intervention effectiveness are found in Table 1 below and trial safety outcomes are found in Table 2 below.

Table 1: Primary and Secondary INSPIRE-ASP UTI Trial Outcomes

Outcome	Metric
**Primary Trial Outcome**	
Extended-Spectrum Days Of Antibacterial Therapy (ES-DOT) per Empiric Day	The summed number of different extended-spectrum antibacterials received each empiric day, measured repeatedly over the first three days of an admission and divided by the number of empiric days of the admission. An empiric day is a day within the first three days of an admission.[1]
**Secondary Trial Outcomes**	
Vancomycin Days of Antibacterial Therapy per Empiric Day	The summed number of days of Vancomycin received each empiric day per at-risk-day (first 3 days of admission)[1]
Antipseudomonal Antibiotic Days Of Therapy (ES-DOT) per Empiric Day	The summed number of different antipseudomonal antibacterials received each empiric day, measured repeatedly over the first three days of an admission and divided by the number of empiric days of the admission. An empiric day is a day within the first three days of an admission.[1,2]

[1]E.g., if a patient is admitted for 2 days, ES-DOT will be calculated for 2 days and divided by 2 empiric days; conversely if a patient is admitted for 4 days, only the first 3 days will be evaluated.

[2]Does not include aminoglycosides or fluoroquinolones.

Safety outcomes planned for the primary manuscript are shown in Table 2. Safety outcomes will be analyzed separately from the main effectiveness outcomes of the trial (see Primary Statistical Analysis section below).

Table 2: Other Pre-Specified Outcomes – Safety Trial Outcomes

**Safety Trial Outcomes (other pre-specified outcomes)**	
Antibacterial Escalations [Safety Outcome]	Days from start of standard-spectrum antibacterial until switch to extended-spectrum antibacterial during hospital stay
ICU Transfers [Safety Outcome]	Days from start of hospitalization until ICU transfer within hospital stay
Length-of-stay [Safety Outcome]	Days from hospital admission to discharge

Primary Statistical Analysis

The primary trial outcome is defined as the summed number of different extended spectrum antibiotics received each day, measured repeatedly over the first three days of an admission and divided by the number of days of the admission within the empiric period. An empiric day is a day within the first three days of an admission. We define this outcome as an Extended-Spectrum Days Of Therapy (ES-DOT) per empiric day. For clarity the calculation is as follows: we define a DOT for a particular ES antibiotic as a day in which any number of doses of that antibiotic is given. Different ES antibiotics are summed across the empiric days for each patient admission, then divided by the empiric days for that patient-admission to determine each admission's DOT per at-risk day. If an admission is less than 3 days, only the number of days the patient is admitted will contribute to the numerator and denominator. Antibiotics given during an associated emergency department visit on the date of hospital admission are counted toward the ES-DOT of the first hospital day.

The main trial results will be based upon as-randomized, unadjusted analyses of admission-level ES-DOT per empiric day. We define ES-DOT per empiric day at the individual admission level so that we can perform analyses on individuals.

The trial will be assessed among the cohort of adult admissions who have an ICD-10 claims code indicating UTI is present on admission, who received any antibiotic within 3 days of admission, and who were admitted to a non-ICU location. For admissions initially on a non-ICU floor and transferred to the ICU, analysis will include only admission days on the non-ICU floor within 3 days of admission.

The trial periods are defined as follows: (1) Baseline – April 1, 2017 – September 30, 2018 (18 months); (2) Phase-in – October 1, 2018 – March 31, 2019 (6 months); (3) Intervention – April 1, 2019 – June 30, 2020 (15 months).

The unit of analysis will be individual admissions. Individuals can contribute more than one admission. The analytic model will be a generalized linear mixed effects model for differences in differences, with random effects accounting for correlation within cluster, period-varying random effects to allow for differences between hospitals from baseline to follow-up, and admission-level random effects to account for correlation within person and hospital. Analyses of the baseline data, performed before randomization, found no evidence of overdispersion in a Poisson model, and so we plan to use that model for analysis of outcomes. The model can be expressed as follows:

$$log(y_{ijph}) = \beta_0 + \beta_1 A_{ijph} + \beta_2 T_{ijph} + \beta_3 A_{ijph} T_{ijph} + b_{0h} + b_{1h} T_{ijph} + g_j$$

where y_{ijph} is the ES-DOT per at-risk day for patient i for admission j in period p at hospital h, and $A_{ijph} = 1$ if hospital h is in the intervention arm and 0 if not, and $T_{ijph} = 1$ if p is the intervention period and 0 if baseline period. The random effects b_{0h} and b_{1h} allow for different baseline mean admission-level ES-DOT per at-risk day for each hospital and each hospital per period, respectively, while g_j allows different admissions to have different mean ES-DOT per at-

risk day across multiple admissions. Equivalently, they allow for correlation within hospital at different levels at baseline and at follow-up.

The assessment of trial success will be determined by the significance of the arm by period interaction term β_3, which assesses whether the log relative rate of the outcome due to being in the intervention arm in the intervention period is different from 0. The exponentiated parameter estimate for β_3 is the estimated relative rate of ES-DOT per at-risk day due to the intervention, relative to the baseline period. For example, if β_3 had a negative value and a p-value <0.05, we would conclude that the patient-specific CPOE smart prompts generated a benefit over routine care. Exponentiating the parameter value would provides an estimate of the relative reduction due to the intervention in the expected ES-DOT per at-risk day.

The primary trial analysis will use an as-randomized unadjusted model with two-tailed significance set at alpha = 0.05. Secondary outcomes also will be assessed using an as-randomized unadjusted model with adjustment for multiple comparisons with two-tailed significance set at alpha = 0.025 for the two secondary outcomes. Subsequent analyses of the primary and secondary outcomes will include both as-treated and adjusted models. Adjusted models will account for individual characteristics such as age, gender, comorbidities, and prior history of MDROs as well as hospital characteristics such as hospital antibiotic resistance. We will also account for randomization strata and for seasonality. These analyses will be reported as point estimates with confidence intervals without p-value. The reason for including these analyses is to provide additional information related to the trial outcomes for readers to assess the effects of potential confounders. The reason to not include them in a formal multiple comparisons adjustment is because these analyses are non-independent evaluations relative to the as-randomized unadjusted analyses.

Safety outcomes noted in the above table will be assessed in the most conservative manner to identify potential safety issues. This is required because a reduction in ES-DOT might be achieved only by incorrectly withholding ES antibiotics when they were really needed. Each safety outcome will be evaluated for non-inferiority using an as-randomized unadjusted model with a one-tailed significance set at alpha = 0.05. Analyses planned for these assessments are proportional hazards models with random effects (to account for hospital effects). These models are sometimes called frailty or shared frailty models. Because these are safety outcomes, we do not intend to make adjustments for multiple comparisons in testing them, further increasing conservatism.

All analyses will be performed using current versions of SAS (Version 9.4, as of writing, SAS Institute, Cary NC) and/or R (Version 4.0, as of writing).

Power Size/Sample Size Calculations

Power assessment proceeded as follows, using a Monte Carlo approach. We used available data to define a baseline period of 10/1/2015-12/31/2016 in a bootstrap procedure. Individuals were selected with replacement from within each hospital, once to represent baseline data and separately to represent intervention period data. In the randomization portion of the Monte Carlo approach, we ordered the hospitals by “size”, meaning the number of admissions with UTI. Then, within strata of 6 hospitals, we ordered by ES-DOT per at-risk day. Then we took pairs of hospitals with adjacent ES-DOT per at-risk day and assigned one member of the pair to one arm. To do this, a single pseudo-random number uniformly distributed between 0 and 1 was generated for each pair. If it was less than 0.5, the arbitrary “first” member of the pair was assigned to Routine Care and the other to Intervention. If it was greater than or equal to 0.5, then the assignments were reversed. The remaining unpaired hospital of the 59 units were assigned as the “first” member of a pair with no match. (Three sets

of hospitals each shared a stewardship program and these hospitals were treated as a single hospital within the routine.)

If an admission was in the intervention arm in the intervention period, then their ES-DOT per at-risk day was reduced by a proportion, given below. The reduction of ES-DOT per at-risk day was selected with a lower bound determined by the investigators as the hoped-for effect of the intervention with minimal clinical significance, and the impact in the Monte Carlo calculation on a per-admission basis was to reduce the days of therapy proportionately between this number and 1, on a uniform distribution. In other words, if the effect of the intervention was to multiply doses by as little as .9, each person would have their doses multiplied by between .9 and 1. Then an integer value of doses would be chosen as described above.

We are interested in determining whether the patient-specific CPOE intervention reduces ES-DOT per at-risk day by at least 12.5% relative to usual care. Using the method above, we have 97% power (CI 91-99%) to detect this effect. Table 4 below shows the calculated power and confidence intervals for the primary and secondary outcomes to be evaluated in our primary manuscript.

Table 4. Power Calculation – Primary and Secondary Outcomes

**Primary Trial Outcome**	**Power (CI), %**
Extended-Spectrum Antibacterial Days Of Therapy (ES-DOT) per Empiric Day	97 (91-99)
**Secondary Trial Outcomes**	
Vancomycin Days of Therapy per Empiric Day	92 (85-96)
Antipseudomonal Antibiotic Days Of Therapy per Empiric Day	84 (77-91)

Extended-spectrum antibiotics are defined in the study protocol and provided below for convenience.

Table 5: INSPIRE-ASP UTI Extended-Spectrum Antibiotics

**Pathogen-Directed Antibiotic Category**	**Extended-Spectrum Antibiotics Targeted in INSPIRE UTI CPOE Prompts**
**Anti-MRSA**	Ceftaroline, Daptomycin, Linezolid[1], Vancomycin[2]
**Anti-VRE**	Daptomycin, Linezolid
**Anti-*Pseudomonas***	Aztreonam, Cefepime, Ceftazidime, Piperacillin/Tazobactam
**Anti-ESBL**	Ertapenem, Ceftolozane/Tazobactam, Meropenem, Imipenem
**Anti-CRE**	Ceftazidime/Avibactam, Colistin, Imipenem/Relebactam, Meropenem/Vaborbactam, Polymixin B, Tigecycline

[1]Both oral and intravenous (IV) formulations, [2]IV formulation only.

Abbreviations: **CPOE** – Computerized Physician Order Entry, **MRSA -** Methicillin Resistant *Staphylococcus aureus,* **ESBL –** Extended Spectrum Beta Lactamase Producing *Enterobacterales,* including multi-drug *Acinetobacter* and *Pseudomonas* species susceptible to Carbapenem. **VRE –** Vancomycin Resistant *Enterococci*, **CRE –** Carbapenem Resistant *Enterobacterales.*

Addendum 3/11/24:

The sentence below has an inadvertent error. In the following, the term "strata" should read "unit", and means that adjusted analyses will include random effects for hospital:
"Adjusted models will account for individual characteristics such as age, gender, comorbidities, and prior history of MDROs as well as hospital characteristics such as hospital antibiotic resistance. We will also account for randomization strata and for seasonality."

INSPIRE Skin and Soft Tissue Infection Trial
Trial Supplement 1

(Originally published in Gohil, et al. Improving Empiric Antibiotic Selection for Patients Hospitalized With Skin and Soft Tissue Infection: The INSPIRE 3 Skin and Soft Tissue Randomized Clinical Trial. JAMA Intern Med. 2025;185(6):680–691. doi:10.1001/ jamainternmed.2025.0887)

Table of Contents

1. **Statistical Analysis Plan**
   a. Summary of changes
   b. Original Statistical Plan – 6/29/22
   c. Final Statistical Plan – 3/29/24

2. **INSPIRE Skin and Soft Tissue Infection Trial Protocol**
   a. Original Trial Protocol and Implementation Toolkit – Routine Stewardship Group
   b. Original Trial Protocol and Implementation Toolkit – CPOE Bundle Group
   c. Final Trial Protocol and Implementation Toolkit – N/A, same as original protocol
   d. Summary of changes – N/A, no changes

Supplement 1
INSPIRE Skin and Soft Tissue Infection Trial
Trial Protocol Amendments

Date	Summary of Trial Protocol Analytic Plan Amendments
**6/29/2022**	**Original**
**3/29/2024**	**Final**<br>Added language to clarify non-inferiority margins for safety outcomes. Removed power calculation language from the SAP. Minor wording changes for clarity.

(Originally published in Gohil, et al. Improving Empiric Antibiotic Selection for Patients Hospitalized With Skin and Soft Tissue Infection: The INSPIRE 3 Skin and Soft Tissue Randomized Clinical Trial. JAMA Intern Med. 2025;185(6):680–691. doi:10.1001/jamainternmed.2025.0887)

INSPIRE Skin/Soft Tissue (SST) Infections Trial
(**IN**telligent **S**tewardship **P**rompts to **I**mprove **R**eal-time **E**mpiric Antibiotic Selection for Patients)

Statistical Analytic Plan

Date: 6.29.2022

Background

The **INSPIRE Skin/Soft Tissue (SST) Infections Trial** (INtelligent Stewardship Prompts to Improve Real-time Empiric Antibiotic Selection for Patients with SST Infections) is a cluster-randomized trial of HCA Healthcare (HCA) affiliated hospitals to determine whether antibiotic prescribing practice for adult hospitalized patients admitted to non-critical care units with SST infections can be affected by providing physicians with individualized risk estimates for whether a patient with an SST infection is likely to have an antibiotic-resistant infection. It compares routine care to the use of a real-time smart prompt for standard-spectrum antibiotics when a physician orders an extended-spectrum antibiotic (ES) for a patient whose risk of antibiotic resistant infection is low (<10%). The main goal is to see if there is a difference in the empiric antibiotic prescribing practices for ES. The term "antibiotics" refers to antibacterial antibiotics in this trial. We define antibiotics administered during the first three days of hospitalization as empiric treatment because infecting pathogens and their antimicrobial susceptibilities are typically not known during this period. We refer to these first three days as the empiric period.

Extended-spectrum antibiotics are defined in Table 1 below.

Table 1: Extended-Spectrum Antibiotics in the INSPIRE-ASP SST Infections Trial

Category	Extended-Spectrum Antibiotics for INSPIRE CPOE Alerts
**Anti-VRE**	Daptomycin, Linezolid[1], Tigecycline
**Anti-Pseudomonas**	Aztreonam, Cefepime, Ceftazidime, Piperacillin/tazobactam
**Anti-ESBL**	Ertapenem, Imipenem, Meropenem, Ceftolozane/tazobactam
**Anti-CRE**	Ceftazidime/avibactam, Imipenem/relebactam, Meropenem/vaborbactam, Polymixin B, Colistin, Tigecycline

[1]Both oral and intravenous (IV) formulations

Note: Does not include aminoglycosides or fluoroquinolones.

Abbreviations: **VRE –** Vancomycin Resistant *Enterococci*; **ESBL –** Extended Spectrum Beta Lactamase producer **CRE** – Carbapenem Resistant Enterobacteriaceae

Participating hospitals are randomized to:

- **Arm 1 – Routine Care**
- **Arm 2 – Real-Time Risk Estimation Smart Prompt**
  Precision medicine smart prompt using a computerized physician order entry (CPOE) alert that calculates a patient-specific risk estimate of antibiotic resistant infection and recommends standard-spectrum antibiotics in patients admitted for SST infections who have low risk (<10%) for an antibiotic resistant infection.

Trial Outcomes

Table 2: Primary and Secondary INSPIRE-ASP SST Infections Trial Outcomes

Outcome	Metric
**Primary Trial Outcome**	
Total Empiric Extended-Spectrum Antibiotic Days of Therapy (ES-DOT)	The number of different extended-spectrum antibiotics each empiric day, summed across the first 3 days of hospitalization.[1]
**Secondary Trial Outcomes**	
Total Empiric Antipseudomonal Antibiotic Days of Therapy (ES-DOT)	The number of different antipseudomonal antibiotics each empiric day, summed across the first 3 days of hospitalization.[1,2]

[1]E.g., if a patient is admitted for 2 days, ES-DOT will be calculated across those 2 days; however if a patient is admitted for 4 days, only the first 3 days will be evaluated. Note that anti-MRSA antibiotics will not be included as extended-spectrum antibiotics.

Safety outcomes planned for the primary manuscript are shown in Table 3. Safety outcomes will be analyzed separately from the main effectiveness outcomes of the trial (see Primary Statistical Analysis section below).

Table 3: Other Pre-Specified Outcomes – Safety Trial Outcomes

**Safety Trial Outcomes (other pre-specified outcomes)**	
ICU Transfer [Safety Outcome]	Days to ICU transfer, between hospital days ≥ 3 and ≤ 14.
Length-of-stay [Safety Outcome]	Days from hospital admission until discharge or hospital day 14, whichever comes first, where admission day is hospital day 1.

Primary Statistical Analysis

The primary trial outcome is defined as the summed number of different ES antibiotics received during each empiric day. An empiric day is a day within the first three days of an admission. We define this outcome as the Total Empiric Extended-Spectrum Days of Therapy (ES-DOT). For clarity, the calculation is as follows: we define a DOT for a particular ES antibiotic as a day in which any number of doses of that antibiotic is given. Different ES antibiotics are summed within each empiric day for each patient admission to determine DOT in that at-risk day. Antibiotics given during an associated emergency department visit on the date of hospital admission are counted toward the ES-DOT of the first hospital day.

The main trial results will be based upon as-randomized, unadjusted analyses of admission-level ES-DOT. In contrast to the national DOT measure defined by CDC for hospitals, we define ES-DOT at the individual admission level so that we can perform analyses on individuals.

The trial will be assessed among the cohort of adult admissions who: 1) have administrative claims codes indicating an SST infection is present on admission; 2) received any antibiotic within 3 days of admission; and 3) were admitted to a non-ICU location. For admissions initially on a non-ICU floor and transferred to the ICU, analysis will include only admission days on the non-ICU floor within 3 days of admission.

The trial periods are defined as follows: (1) Baseline: January 1, 2019 – December 31, 2019 (12 months); (2) Phase-in: August 2, 2022 – December 31, 2022 (5 months, does not contribute to analysis); (3) Intervention: January 1, 2023 – December 30, 2023 (12 months). The main evaluation of all outcomes will be difference-in-difference between the Intervention and Baseline periods and between study arms. Trial analysis will use a baseline period prior to the arrival of the COVID-19 pandemic since hospital operations and case mix were severely impacted by this pandemic between Winter 2020-Spring, 2022.[1-4] Although at the time of this writing (June, 2022), COVID-19 surges continue to occur nationally, clinical operations and patient case mix at most hospitals have largely returned to near pre-pandemic activity in the setting of widespread

vaccination and milder virus strains. Phase-In is scheduled for August, 2022 as participating hospitals are in a state of recovery sufficient to launch the trial intervention with pharmacy/physician engagement.

The unit of analysis will be individual admissions. Individuals can contribute more than one admission. The analytic model will be a generalized linear mixed effects model for differences in differences, with random effects accounting for correlation within cluster, period-varying random effects to allow for differences within hospitals between baseline and follow-up, and admission-level random effects to account for correlation within person and hospital, if sufficient data exist. We plan to use a Poisson model for analysis of outcomes as follows:

$$log(y_{ijph}) = \beta_0 + \beta_1 A_{ijph} + \beta_2 T_{ijph} + \beta_3 A_{ijph} T_{ijph} + b_{0h} + b_{1h} T_{ijph} + g_j + \log(ed_j)$$

where y_{ijph} is the ES-DOT for patient i for admission j in period p at hospital h, and $A_{ijph} = 1$ if hospital h is in the intervention arm and 0 if not, and $T_{ijph} = 1$ if p is the intervention period and 0 if baseline period. The random effects b_{0h} and b_{1h} allow for different baseline mean admission-level ES-DOT for each hospital and each hospital in the intervention period, respectively. Equivalently, they allow for correlation within hospital at different levels at baseline and at follow-up. The random effect g_j allows each patient to have a different mean ES-DOT. Finally, ed_j is the number of empiric days in the admission.

The assessment of trial success will be determined by the significance of the arm by period interaction term β_3, which assesses whether the log relative rate of the outcome due to being in the intervention arm in the intervention period is different from 0. The exponentiated parameter estimate for β_3 is the estimated relative rate of ES-DOT per at-risk day due to the intervention, relative to the baseline period. For example, if β_3 had a negative value, we would conclude that the patient-specific CPOE smart prompts generated a benefit over routine care. Exponentiating the parameter value would provide an estimate of the relative reduction due to the intervention in the expected ES-DOT per at-risk day.

The primary trial analysis will use an as-randomized unadjusted model with two-tailed significance set at alpha = 0.05. Secondary outcomes will be assessed using an as-randomized unadjusted model and will include adjustment for multiple comparisons with two-tailed significance set at alpha = 0.025 for the secondary outcome.

Subsequent analyses of the primary and secondary outcomes will include both as-treated and adjusted models. Adjusted models will account for individual characteristics such as age, gender, comorbidities, and prior history of MDROs as well as hospital characteristics such as hospital antibiotic resistance. We will also account for seasonality. All adjusters will be determined a priori. These analyses will be reported as point estimates with confidence intervals and without p-values. The reason for including these analyses is to provide additional information related to the trial outcomes for readers to assess the effects of potential confounders. The reason to not include them in a formal multiple comparisons adjustment is because these analyses are non-independent evaluations relative to the as-randomized unadjusted analyses.

We will separately evaluate intervention period effects by study arm alone.

Safety Outcomes

Safety outcomes noted in the above table will be assessed in the most conservative manner to identify potential safety issues. Safety outcomes are required because a reduction in ES-DOT might be achieved only by incorrectly withholding ES antibiotics when they were really needed. Each safety outcome will be evaluated for non-inferiority using an as-randomized unadjusted analysis with a one-tailed significance set at alpha = 0.05. Length-of-stay and days to ICU transfer are evaluated within 14 days of admission since empiric antibiotic selection within the first 3 hospital days is not expected to be the main driver of either outcome beyond this time period; evaluation of baseline data also shows that 91% of SST patients have length-of-stay equal

to or less than 14 days. Analyses planned for these assessments are proportional hazards models with random effects for each hospital and admission These models are sometimes called frailty or shared frailty models. Because these are safety outcomes, we do not intend to make adjustments for multiple comparisons in testing them, further increasing conservatism.

All analyses will be performed using current versions of SAS (Version 9.4, as of writing, SAS Institute, Cary NC) and/or R (Version 4.0, as of writing).

C.2.11 Power Size/Sample Size Calculations

Power assessment proceeded as follows, using a Monte Carlo approach. We used available data to define a baseline period of 1/1/2019-12/31/2019. In a bootstrap procedure, individuals were selected with replacement from within each hospital, once to represent baseline data and separately to represent "intervention period" data. Hospitals were then "randomized" to intervention or control according to multiple parameters, including number of admissions with SST infection and ES-DOT. We then modified the outcomes for the bootstrapped individuals in the "intervention" period in the "intervention" arm to reflect the effect of the intervention.[5]

We assessed power to identify an overall 12.5% relative reduction in ES-DOT. This effect was implemented by reducing the relative empiric days of therapy for each admission which was exposed to the CPOE prompt. The amount of reduction was selected from a uniform random variate between 0.75 and 1. For example, if the bootstrapped patient initially received 8 ES-DOT and the random reduction was 0.875, then that admission intervention ES-DOT would be reduced to (8 * 0.875) = 7 days. The reduction of ES-DOT was selected with a lower bound determined by the investigators as the minimal effect achieved that would be clinically meaningful. Using this method, we have ≥99.9% (99.6-99.9%) power to detect this effect.

Since adoption of the intervention will be heavily influenced by the ability to demonstrate safety, our study size was based on the power to identify at least a one day increase in length-of-stay for 12.5% of patients and a 2% increase in ICU stay on hospital day 3. . We used methods similar to the above to calculate the power to identify at least a one day increase in length-of-stay for 12.5% of patients who had hospitalizations less than 14 days and found the power was 68.9% (CI 65.9-71.8%), where non-inferiority is defined as a hazard ratio no smaller than 0.98. For days to ICU transfer, we estimated the power to detect an absolute 2% increase in transfers to the ICU on hospital day 3 through 14 to be >99.9% (CI 99.6-99.9%), where non-inferiority is defined as having a hazard ratio no greater than 1.1 (fewer days to ICU results in a hazard ratio above 1).

For completeness, we note that there are other pre-specified outcomes intended for exploratory analysis in secondary papers for the INSPIRE ABD trial in Table 5 below.

Table 5: Secondary Manuscripts - Other Pre-Specified Outcomes

**Secondary Manuscripts (other pre-specified secondary exploratory analyses for later manuscripts)**	
Inpatient Extended-Spectrum Days of Therapy after the Empiric Period	The number of different ES antibiotics received each day, on hospital days ≥4 and ≤14.
Empiric and Total Antibiotic Costs	Empiric and total antibiotic costs during hospitalization
Incidence of Hospital-Onset *C. difficile*	Hospital-onset *C. difficile* positive tests (specimen obtained) on hospital days ≥4 and ≤14.
Incidence of Hospital-Onset MDRO-positive Cultures	Newly-detected hospital-onset MDRO-positive cultures on hospital days ≥4 and ≤14. Includes total MDRO and specific MDRO subsets.

1. Moynihan R, et al. Impact of COVID-19 pandemic on utilisation of healthcare services: a systematic review. BMJ Open 2021;11:e045343. doi:10.1136/ bmjopen-2020-045343.
2. Berkmeyer, JD, et al. The Impact Of The COVID-19 Pandemic On Hospital Admissions In The United States. Health Affairs (39):11, September, 2020. https://doi.org/10.1377/hlthaff.2020.00980
3. Richmond BK, et al. The Impact of the COVID-19 Pandemic on Surgical Practice in the Southeastern United States: Results of a Survey of the Membership of the Southeastern Surgical Congress. Am Surg. 2020 Aug;86(8):916-925. doi: 10.1177/0003134820945203. Epub 2020 Sep 14.
4. Nguyen JL, et al. Pandemic-related declines in hospitalization for non-COVID-19-related illness in the United States from January through July 2020. PLoS ONE 17(1): e0262347. https://doi.org/10.1371/journal.pone.0262347.
5. Kleinman K, Huang SS. Calculating power by bootstrap, with an application to cluster-randomized trials. eGEMs (Generating Evidence & Methods to improve patient outcomes) 2016 4:1, Article 32. doi: http://dx.doi.org/10.13063/2327-9214.1202

(Originally published in Gohil, et al. Improving Empiric Antibiotic Selection for Patients Hospitalized With Skin and Soft Tissue Infection: The INSPIRE 3 Skin and Soft Tissue Randomized Clinical Trial. JAMA Intern Med. 2025;185(6):680–691. doi:10.1001/jamainternmed.2025.0887)

INSPIRE Skin and Soft Tissue Infections Trial
(**IN**telligent **S**tewardship **P**rompts to **I**mprove **R**eal-time **E**mpiric Antibiotic Selection for Patients)

Statistical Analytic Plan, 3/29/2024

Ken Kleinman, ScD

Background

The **INSPIRE Skin and Soft Tissue (SST) Infections Trial** (INtelligent Stewardship Prompts to Improve Real-time Empiric Antibiotic Selection for Patients with Skin and Soft Tissue Infections) is a cluster-randomized trial of HCA Healthcare (HCA) affiliated hospitals to determine whether antibiotic prescribing practice for adult hospitalized patients admitted to non-critical care units with skin and soft tissue (SST) infections can be affected by providing physicians with individualized risk estimates for whether a patient with an SST infection is likely to have an antibiotic-resistant infection. It compares routine care to a real-time smart prompt to use a standard-spectrum antibiotics when a physician orders an extended-spectrum antibiotic (ES) for a patient whose risk of antibiotic resistant infection is low (<10%). The main goal is to see if there is a difference in the empiric antibiotic prescribing practices for ES. The term “antibiotics” refers to antibacterial antibiotics in this trial. We define antibiotics administered during the first three days of hospitalization as empiric treatment because infecting pathogens and their antimicrobial susceptibilities are typically not known during this period. We refer to these first three days as the empiric period.

Extended-spectrum antibiotics are defined in Table 1 below.

Table 1: Extended-Spectrum Antibiotics in the INSPIRE-ASP Skin and Soft Tissue Infections Trial

Category	Extended-Spectrum Antibiotics for INSPIRE CPOE Alerts
**Anti-VRE**	Daptomycin, Linezolid[1], Tigecycline
**Anti-Pseudomonals**	Aztreonam, Cefepime, Ceftazidime, Piperacillin/tazobactam
**Anti-ESBL**	Ertapenem, Imipenem, Meropenem, Ceftolozane/tazobactam
**Anti-CRE**	Ceftazidime/avibactam, Imipenem/relebactam, Meropenem/vaborbactam, Polymixin B, Colistin, Tigecycline

[1]Both oral and intravenous (IV) formulations

Note: Does not include aminoglycosides or fluoroquinolones.

Abbreviations: **MRSA -** Methicillin Resistant *Staphylococcus aureus*; **VRE –** Vancomycin Resistant *Enterococci*; **ESBL –** Extended Spectrum Beta Lactamase producer; CRE – Carbapenem Resistant Enterobacteriaceae

Participating hospitals are randomized to:

- **Arm 1 – Routine Care**
- **Arm 2 – Real-Time Risk Estimation Smart Prompt**

Computerized physician order entry (CPOE) prompt that recommends standard-spectrum antibiotics in patients admitted for SST infections who are prescribed extended-spectrum antibiotics and calculated to have low risk (<10%) for an antibiotic-resistant infection.

Trial Outcomes

Table 2: Primary and Secondary INSPIRE-ASP Skin and Soft Tissue Infections Trial Outcomes

Outcome	Metric
**Primary Trial Outcome**	
Total Empiric Extended-Spectrum Antibiotic Days of Therapy (ES-DOT)	The number of different extended-spectrum antibiotics received by the patient each empiric day, summed across the first 3 days of hospitalization.[1]
**Secondary Trial Outcome**	
Total Empiric Antipseudomonal Antibiotic Days of Therapy	The number of different antipseudomonal antibiotics received by the patient each empiric day, summed across the first 3 days of hospitalization.[1]

[1]E.g., if a patient is admitted for 2 days, ES-DOT will be calculated across those 2 days; however if a patient is admitted for 4 days, only the first 3 days will be evaluated.

Safety outcomes planned for the primary manuscript are shown in Table 3. Length of stay and days to ICU transfer are evaluated within 14 days of admission since empiric antibiotic selection within the first 3 hospital days is not expected to be the main driver of either outcome beyond this time; evaluation of baseline data also shows that 91% of patients with skin and soft tissue infections have length-of-stay equal to or less than 14 days.

Table 3: Other Pre-Specified Outcomes – Trial Safety Outcomes

**Safety Trial Outcomes (other pre-specified outcomes)**	
ICU Transfer [Safety Outcome]	Days to ICU transfer, between hospital days ≥ 3 and ≤ 14.
Length-of-stay [Safety Outcome]	Days from hospital admission until discharge or hospital day 14, whichever comes first, where admission day is hospital day 1.

Data Collection Details

The trial will be assessed among the cohort of adult admissions who: 1) have administrative claims codes indicating an SST infection is present on admission; 2) received any antibiotic within 3 days of admission; and 3) were admitted to a non-ICU location.

The rationale for including only the first three days of the admission is that this is when the actual status of the patient with respect to the need for an ES antibiotic is unknown. Bearing this in mind, antibiotics given during an associated emergency department visit on the date of hospital admission are counted toward the ES-DOT of the first hospital day.

The trial baseline period includes January 1, 2019 – December 31, 2019 (12 months). The intervention period includes January 1, 2023 – December 31, 2023 (12 months). The baseline was selected to include time prior to the arrival of the COVID-19 pandemic since hospital operations and case mix were dramatically affected between Winter 2020-Spring, 2022.[1-4] Although COVID-19 surges have continued to occur, clinical operations and patient case mix at most hospitals have largely returned to near pre-pandemic activity. We considered that a state of pre-pandemic normalcy returned by summer of 2022, and introduced our intervention on August 2, 2022. The period between then and December 31, 2022 is considered a phase-in period and is not used in trial evaluation.

Primary Statistical Analysis

The primary trial outcome is defined as the summed number of different ES antibiotics received during each empiric day, on the patient-admission level. An empiric day is a day within the first three days of an admission. We define this outcome as the Total Empiric Extended-Spectrum Days of Therapy (ES-DOT). For clarity, the calculation is as follows: we define a DOT for a particular ES antibiotic as a day in which any number of doses of that antibiotic is given. Different

ES antibiotics are summed within each empiric day for each patient admission to determine DOT in that at-risk day.

The main trial results will be based upon as-randomized, unadjusted analyses of admission-level ES-DOT. We note that the national DOT measure defined by CDC is measured at the hospital level. We define ES-DOT at the individual admission level so that we can perform analyses on individuals.

The main evaluation of all outcomes will be based on the difference in differences between the intervention and baseline periods and between study arms.

The unit of analysis will be individual admissions. Individual patients can contribute more than one admission. The analytic model will be a generalized linear mixed effects model for differences in differences, with random effects to account for correlation within hospital between period, within hospital within period between patient, and within patient between admission. We plan to use a Poisson model for analysis of outcomes as follows:

$$log(y_{ijph}) = \beta_0 + \beta_1 A_{ijph} + \beta_2 T_{ijph} + \beta_3 A_{ijph} T_{ijph} + b_{0h} + b_{1h} T_{ijph} + g_j + \log{(ed_j)}$$

where y_{ijph} is the ES-DOT for patient i for admission j in period p at hospital h, and $A_{ijph} = 1$ if hospital h is in the intervention arm and 0 if not, and $T_{ijph} = 1$ if p is the intervention period and 0 if baseline period. The random effects b_{0h} and b_{1h} allow for different baseline mean admission-level ES-DOT for each hospital and each hospital in the intervention period, respectively. Equivalently, they allow for correlation within hospital at different levels at baseline and at follow-up. The random effect g_j allows each patient to have a different mean ES-DOT. Finally, ed_j is the number of empiric days in the admission. We note that it may not be possible to fit models with the g_j parameter, for various numerical and computational reasons—the most prominent being a relative sparsity of patients with readmissions. If that proves to be the case, we will randomly select one admission per patient and use the model above without that parameter and omitting the j subscript.

The assessment of trial success will be determined by assessing the significance of the arm by period interaction term β_3, which estimates the log relative rate of the outcome due to being in the intervention arm in the intervention period. The exponentiated parameter estimate for β_3 is the estimated relative rate of ES-DOT per at-risk day due to the intervention, relative to the baseline period. For example, if β_3 had a negative value and a p-value <0.05, we would conclude that the patient-specific CPOE smart prompts generated a benefit over routine care. Exponentiating the parameter value would provide an estimate of the relative reduction due to the intervention in the expected ES-DOT per at-risk day.

The primary trial analysis will use an as-randomized unadjusted model with two-tailed significance set at alpha = 0.05. The secondary outcome will be assessed using an as-randomized unadjusted model with alpha = 0.05.

Subsequent analyses of the primary and secondary outcome will include both as-treated and adjusted models. Adjusted models will account for individual characteristics such as age, gender, comorbidities, and prior history of MDROs as well as hospital characteristics such as hospital antibiotic resistance. We will also account for seasonality. All adjusters will be determined a priori. These analyses will be reported as point estimates with confidence intervals and without p-values. The reason for including these analyses is to provide additional information related to the trial outcomes for readers to assess the effects of potential confounders. The reason to not include them in a formal multiple comparisons adjustment is because these analyses are non-independent evaluations relative to the as-randomized unadjusted analyses.

Safety Outcomes

Safety outcomes are required because a reduction in ES-DOT might be achieved only by incorrectly withholding ES antibiotics when they were necessary. Each safety outcome will be

evaluated for non-inferiority using an as-randomized unadjusted analysis with a one-tailed significance set at alpha = 0.05. For length of stay, the non-inferiority margin is a hazard ratio of 0.98. For days to ICU transfer, the non-inferiority margin is a hazard ratio of 1.1 (fewer days to ICU results in a hazard ratio above 1). Analyses planned for these assessments are proportional hazards models with random effects for each time period, hospital, and admission. These models are sometimes called frailty or shared frailty models. Because these are safety outcomes, we do not intend to make adjustments for multiple comparisons in testing them, further increasing conservatism. As above, it may not be possible to include multiple admissions.

All analyses will be performed using current versions of SAS/STAT and/or R packages lme4 and coxme, unless better packages become available in the meantime.

Further Planned Analyses

We note that there are other pre-specified outcomes intended for exploratory analysis in secondary papers for the INSPIRE SST trial in Table 5 below.

Table 5: Secondary Manuscripts - Other Pre-Specified Outcomes

**Secondary Manuscripts (other pre-specified secondary exploratory analyses for later manuscripts)**	
Inpatient Extended-Spectrum Days of Therapy after the Empiric Period	The number of different ES antibiotics received each day, on hospital days ≥4 and ≤14.
Empiric and Total Antibiotic Costs	Empiric and total antibiotic costs during hospitalization
Incidence of Hospital-Onset *C. difficile*	Hospital-onset *C. difficile* positive tests (specimen obtained) on hospital days ≥4 and ≤14.
Incidence of Hospital-Onset MDRO-positive Cultures	Newly-detected hospital-onset MDRO-positive cultures on hospital days ≥4 and ≤14. Includes total MDRO and specific MDRO subsets.

1. Moynihan R, et al. Impact of COVID-19 pandemic on utilisation of healthcare services: a systematic review. BMJ Open 2021;11:e045343. doi:10.1136/ bmjopen-2020-045343.
2. Berkmeyer, JD, et al. The Impact Of The COVID-19 Pandemic On Hospital Admissions In The United States. Health Affairs (39):11, September, 2020. https://doi.org/10.1377/hlthaff.2020.00980
3. Richmond BK, et al. The Impact of the COVID-19 Pandemic on Surgical Practice in the Southeastern United States: Results of a Survey of the Membership of the Southeastern Surgical Congress. Am Surg. 2020 Aug;86(8):916-925. doi: 10.1177/0003134820945203. Epub 2020 Sep 14.
4. Nguyen JL, et al. Pandemic-related declines in hospitalization for non-COVID-19-related illness in the United States from January through July 2020. PLoS ONE 17(1): e0262347. https://doi.org/10.1371/journal.pone.0262347.

Arm 1:
INSPIRE Routine Stewardship

Abdominal & Skin/Soft Tissue Infection Trials

Toolkit Binder

Funded by the National Institutes of Health (NIH)

Welcome

Welcome to the **INSPIRE-ASP** (**IN**telligent **S**tewardship **P**rompts to **I**mprove **R**eal-time **E**mpiric **A**ntibiotic **S**election for **P**atients) Trials. The INSPIRE-ASP Abdominal and INSPIRE-ASP Skin & Soft Tissue Infection Trials are cluster-randomized trials of 102 HCA Healthcare hospitals to assess best practice in antibiotic stewardship. These trials are a partnership between HCA Healthcare and academic investigators who are federally funded by the National Institutes of Health (NIH).

Summary of Goals

Up to 40% of patients who are prescribed antibiotics in U.S. hospitals either do not require these drugs or receive inappropriate antibiotics for the condition being treated. Moreover, inappropriate broad-spectrum antibiotics are often prescribed empirically due to concerns about infection with multidrug-resistant organisms (MDROs), despite the low risk of MDRO infection in the vast majority of clinical settings. This is the case for patients admitted with abdominal or skin/soft tissue infections, two common conditions for antibiotic use in hospitalized patients. These trials will compare two strategies to determine best practices for empiric antibiotic selection. Arm 1, Routine Care, will continue antibiotic stewardship practices according to current facility strategies. Arm 2, Smart Prompt Intervention, will implement a precision medicine smart prompt using a computerized physician order entry (CPOE) alert that provides clinicians with a patient's individual probability of MDRO infection and recommends a standard-spectrum antibiotic for patients at low risk for MDRO abdominal or skin/soft tissue infections in non-critical care units. This trial will provide real-time essential information to clinicians to support and optimize empiric antibiotic selection.

Funded by National Institute of Allergy and Infectious Diseases

INtelligent Stewardship Prompts to Improve Real-time Empiric Antibiotic Selection for Patients

Arm 1 – Routine Stewardship

Trial Protocol Implementation Toolkit
Table of Contents

Title **Page**

Welcome & Summary of Goals 2

Table of Contents 3

INSPIRE Team 4

Study Champion Roles and Responsibilities 7

Study Champion FAQs 8

Smart Prompt Workflow 10

Funded by National Institute of Allergy and Infectious Diseases

Study Investigators and Stakeholders

University of California Irvine
Shruti Gohil, MD MPH, Lead Investigator
Susan Huang, MD MPH, Senior Investigator

Harvard Medical School/Harvard Pilgrim Health Care Institute
Ed Septimus, MD FACP FIDSA FSHEA
Richard Platt, MD MSc

HCA Healthcare
Jackie Blanchard, MSN, RN, CIC, VP, Infection Prevention
Chenette Burks, PharmD, Director Pharmacy Services
Will Cooper, PharmD CPHIMS, Manager, Clinical Pharmacy Informatics
Michael Cuffe, MD, Executive VP and Chief Clinical Officer
Samir Fakhry, MD, VP, Center for Trauma and Acute Care Surgery Research
Robert Foster, RN, VP of Emergency Services
Jeffrey Guy, MD, MSc, VP, Care Process Design and Clinical Engineering
Karla Miller, PharmD, VP Pharmacy Services, Chief Patient Safety Officer
Julia Moody, MS, SM (ASCP), Clinical Director, Infection Prevention
Russell Poland, PhD, AVP, Research and Extramural Collaborations/Partnerships
Risa Rahm, PharmD CPHIMS, Senior Director, Clinical Pharmacy Informatics
Kaleb Roemer, PharmD, Manager, Clinical Pharmacy Services
Kenneth Sands, MD MPH, VP Chief Epidemiologist

Brigham & Women's Hospital
David Kubiak, PharmD

University of Massachusetts Amherst
Ken Kleinman, ScD

Rush University
Robert A. Weinstein, MD
Mary Hayden, MD

Funded by National Institute of Allergy and Infectious Diseases

Study Administrative Team

University of California Irvine

Victoria Ngo, BS, Assistant Clinical Research Coordinator

Harvard Medical School/Harvard Pilgrim Health Care Institute

Taliser Avery, MS, Lead Research Analyst

Micaela Coady, MS, Senior Program Manager

Allison Froman, MPH, Research Analyst

Selsebil Sljivo, MPH, Senior Project Coordinator

Neha Varma, MPH, Senior Research Analyst

Chamaine Washington, MPH, Project Coordinator

HCA Healthcare

Thomas Johnson, Senior MEDITECH Programmer & NPR Report Writer

Laura McLean, M.Ed, Senior Data Analyst

Brittany Meador, BS, Program Coordinator, Extramural Collaborations & Partnerships

Naoise Nickolay, RPh, Pharmacy Informatics Consultant

Kimberly Smith, MBA, Director, Research Operations

Funded by National Institute of Allergy and Infectious Diseases

Contact Matrix

Topic	What To Do	Who To Contact	Contact Information
**Study and Protocol Related Questions**	Call or Email	**Victoria Ngo**<br>**Seby Sljivo**	HCAINSPIRE@gmail.com<br>(844) 522-4677
**Pharmacy**	Email	**Chenette Burks, PharmD**<br>Director, Clinical Pharmacy Services<br><br>**Kaleb Roemer, PharmD**<br>Manager, Clinical Pharmacy Services	Ruby.Burks@hcahealthcare.com<br><br>Kaleb.Roemer@hcahealthcare.com
**IT**	Email	**Risa Rahm, PharmD CPHIMS**<br>Senior Director<br>Clinical Pharmacy Informatics	Risa.Rahm@hcahealthcare.com
**IRB Questions**	Call or Email	**Seby Sljivo**	HCAINSPIRE@gmail.com<br>(844) 522-4677

***Do not disclose protected health information (PHI) to INSPIRE Study Staff. If you need to share PHI, we will direct you to HCA Healthcare Study Investigators.**

For questions related to HCA Healthcare (HCA) hospital policy and processes:

HCA Investigator	Phone Number	Email
**Kenneth Sands, MD MPH**<br>VP, Chief Epidemiologist and Patient Safety Officer	(615) 344-1123	Kenneth.Sands@hcahealthcare.com
**Russell Poland, PhD**<br>AVP, Research and<br>Extramural and Collaborative Partnerships	(615) 344-2330	Russell.Poland@hcahealthcare.com

Funded by National Institute of Allergy and Infectious Diseases

Study Champion Roles and Responsibilities

Pharmacy & Physician Study Champions will partner with the INSPIRE investigative team and are expected to:

1. Join trial coaching calls
2. Actively engage and educate staff, leadership, and other stakeholders on the value of participating in the INSPIRE Trials
3. Support current antimicrobial stewardship practices
4. Avoid and/or report interventions or activities that may impact trial outcomes or those targeting empiric antibiotic selection for abdominal and skin/soft tissue infection interventions

Funded by National Institute of Allergy and Infectious Diseases

INSPIRE Abdominal and Skin & Soft Tissue Infection Trials
Study Champion FAQs – Arm 1

What are the INSPIRE-ASP Trials?
The INSPIRE-ASP (**IN**telligent **S**tewardship **P**rompts to **I**mprove **R**eal-time **E**mpiric **A**ntibiotic **S**election for **P**atients) Trials are two separate cluster-randomized trials of HCA Healthcare hospitals to test an antibiotic stewardship intervention abdominal (ABD) and skin and soft tissue (SST) infections.

- **Arm 1** – **Continue usual, gold-standard antibiotic stewardship activities**
- **Arm 2** – **CPOE Smart Prompt Alert Intervention**
    1. Provides patient-specific risk estimate for ABD or SST infections due to a multi-drug resistant organism (MDRO) when extended-spectrum (ES) antibiotics are ordered
    2. Recommends standard antibiotics in non-ICU patients with low (<10%) MDRO risk

What is the study timeline?
- **Prompt go-live** – August 2, 2022
- **Phase-In** (5 months) – August-December, 2022
- **Intervention** (12 months) – January-December, 2023

What are the outcomes of the INSPIRE-ASP Trials
The goal is to identify a best practice strategy for choosing empiric (within 3 calendar days) antibiotics based on a patient's risk for MDRO infection. Outcomes include:

- **Primary**: Empiric extended-spectrum days of therapy
- **Secondary**: Empiric Vancomycin (ABD infection only) and antipseudomonal days of therapy
- **Safety outcomes**: length of stay, ICU transfers
- **Other**: post-empiric ES antibiotic use, MDRO and *C. difficile* rates, antibiotic costs

Why does INSPIRE focus on ABD and SST infections?
ABD and SST infections are among the most common reasons for infection-related admission and ES antibiotic use. Yet, few studies have evaluated effective empiric antibiotic stewardship strategies for these infections.

What will be required of participating Arm 1 sites?
Study champions will be asked to:

- Join trial coaching calls
- Actively engage and educate staff, leadership, and other stakeholders on the value of participating in the INSPIRE Trials
- Continue usual antibiotic stewardship activities
- Avoid and/or report interventions or activities that may impact trial outcomes or those targeting empiric antibiotic selection for abdominal and skin/soft tissue infection interventions

How do we avoid competing interventions and how are these defined?

Participating facilities are fully expected to continue their stewardship activities and initiatives but should avoid activities that may directly impact trial outcomes. Such activities are anticipated to be rare. Examples of interventions that may directly conflict with the trial include:

- A new stewardship strategy that aims to reduce the use of ES antibiotics in the Emergency Department (ED) or within 3 days of admission trial coaching calls
- A new real-time antibiotic de-escalation strategy in the ED or within 3 calendar days of admission
- A new rapid diagnostic tool for ABD or SST infection that would be used in the empiric period to guide antibiotic therapy within 3 days of admission
- Ordering antibiotics through Patient Keeper

Has this study received IRB approval? Will patients need to give informed consent?

Yes. Harvard Pilgrim's IRB will provide ethical oversight for these trials and serve as the central IRB. Both INSPIRE ABD and SST infection trials have been approved by the Harvard IRB. Your hospital has submitted all ceding and reliance agreements. Informed consent will not be required. This study has been approved as a minimal risk study and will be implemented as a quality improvement (QI) protocol through the existing infrastructure of your hospital's antibiotic stewardship programs. The assigned protocol will be approved through your usual clinical/leadership committees and become hospital guidance on appropriate empiric antibioticuse.

Who can I contact for more information?

INSPIRE Study Team
Research Coordinator
HCAINSPIRE@gmail.com, **949-824-7806**

Lead Investigator – Shruti Gohil, MD, MPH, UC Irvine
skgohil@hs.uci.edu

Co-Investigator – Edward Septimus, MD, FACP, Senior Lecturer HPHCI
eseptimus@gmail.com

Manager, Clinical Pharmacy Services – Kaleb Roemer, PharmD
Kaleb.Roemer@hcahealthcare.com

Director, Clinical Pharmacy Services – Chenette Burks, PharmD
Ruby.Burks@hcahealthcare.com

Funded by National Institute of Allergy and Infectious Diseases

Arm 1 – Antibiotic Indication Screen Workflow

The requirement for providing an indication for antibiotic use is a national standard for antibiotic stewardship. HCA Healthcare originally released antibiotic indication screens in 2017 and are currently implemented in the emergency department and inpatient areas throughout HCA Healthcare facilities nationwide.

As an Arm 1 facility, our hospital's primary role is to serve as the gold standard against which intervention (Arm 2) can be compared. To ensure that the antibiotic indication screen workflow is balanced between both study arms, beginning **August 2, 2022** the following screens will appear when extended-spectrum antibiotics are ordered**.**

INSPIRE Arm 1 Abdominal/Skin & Soft Tissue Infection Workflow

1. **When an extended-spectrum antibiotic is ordered for patients with abdominal or skin/soft tissue infection AND the patient is located in the ED at the time of order, then** you will be asked whether the patient is ICU-bound (already live for patients with pneumonia and UTI).

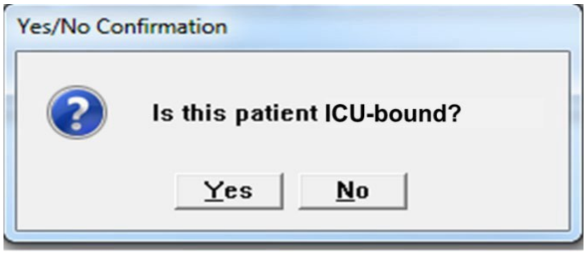

2. **For abdominal patients only, if Anti-MRSA and antipseudomonal antibiotics are ordered and patient has a history of MRSA or antipseudomonal antibiotics then** you will be asked whether the patient has a history of abdominal surgery.

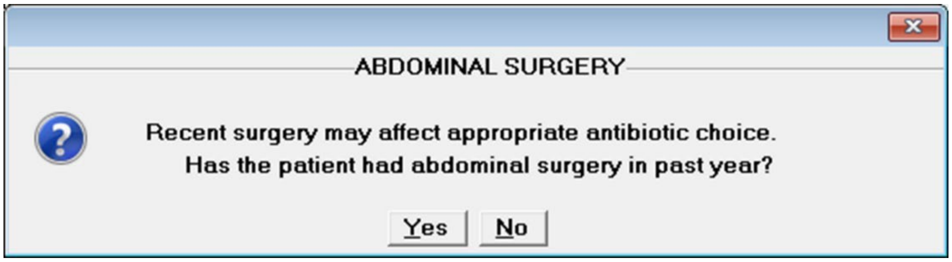

Arm 2:
INSPIRE Stewardship Bundle

Abdominal & Skin/Soft Tissue Infection Trials

Toolkit Binder

Funded by the National Institutes of Health (NIH)

Welcome

Welcome to the **INSPIRE-ASP** (**IN**telligent **S**tewardship **P**rompts to **I**mprove **R**eal-time **E**mpiric **A**ntibiotic **S**election for **P**atients) Trials. The INSPIRE-ASP Abdominal and INSPIRE-ASP Skin & Soft Tissue Infection Trials are cluster-randomized trials of 102 HCA Healthcare hospitals to assess best practice in antibiotic stewardship. These trials are a partnership between HCA Healthcare and academic investigators who are federally funded by the National Institutes of Health (NIH).

Summary of Goals

Up to 40% of patients who are prescribed antibiotics in U.S. hospitals either do not require these drugs or receive inappropriate antibiotics for the condition being treated. Moreover, inappropriate broad-spectrum antibiotics are often prescribed empirically due to concerns about infection with multidrug-resistant organisms (MDROs), despite the low risk of MDRO infection in the vast majority of clinical settings. This is the case for patients admitted with abdominal or skin/soft tissue infections, two common conditions for antibiotic use in hospitalized patients. These trials will compare two strategies to determine best practices for empiric antibiotic selection. Arm 1, Routine Care, will continue antibiotic stewardship practices according to current facility strategies. Arm 2, Smart Prompt Intervention, will implement a precision medicine smart prompt using a computerized physician order entry (CPOE) alert that provides clinicians with a patient's individual probability of MDRO infection and recommends a standard-spectrum antibiotic for patients at low risk for MDRO abdominal or skin/soft tissue infections in non-critical care units. This trial will provide real-time essential information to clinicians to support and optimize empiric antibiotic selection.

Funded by National Institute of Allergy and Infectious Diseases

INtelligent Stewardship Prompts to Improve Real-time Empiric Antibiotic Selection for Patients

Arm 2 – Smart Prompt

Trial Protocol Implementation Toolkit
Table of Contents

Title **Page**

Welcome & Summary of Goals… 2

Table of Contents 3

INSPIRE Team 4

Study Champion Materials 7

Clinician Education Materials 17

Clinician Hot Topics 27

INSPIRE Presentation.. 32

Smart Prompt Workflow 72

Promotional Material 78

Funded by National Institute of Allergy and Infectious Diseases

Study Investigators and Stakeholders

University of California Irvine
Shruti Gohil, MD MPH, Lead Investigator
Susan Huang, MD MPH, Senior Investigator

Harvard Medical School/Harvard Pilgrim Health Care Institute
Ed Septimus, MD FACP FIDSA FSHEA
Richard Platt, MD MSc

HCA Healthcare
Jackie Blanchard, MSN, RN, CIC, VP, Infection Prevention
Chenette Burks, PharmD, Director Pharmacy Services
Will Cooper, PharmD CPHIMS, Manager, Clinical Pharmacy Informatics
Michael Cuffe, MD, Executive VP and Chief Clinical Officer
Samir Fakhry, MD, VP, Center for Trauma and Acute Care Surgery Research
Robert Foster, RN, VP of Emergency Services
Jeffrey Guy, MD, MSc, VP, Care Process Design and Clinical Engineering
Karla Miller, PharmD, VP Pharmacy Services, Chief Patient Safety Officer
Julia Moody, MS, SM (ASCP), Clinical Director, Infection Prevention
Russell Poland, PhD, AVP, Research and Extramural Collaborations/Partnerships
Risa Rahm, PharmD CPHIMS, Senior Director, Clinical Pharmacy Informatics
Kaleb Roemer, PharmD, Manager, Clinical Pharmacy Services
Kenneth Sands, MD MPH, VP Chief Epidemiologist

Brigham & Women's Hospital
David Kubiak, PharmD

University of Massachusetts Amherst
Ken Kleinman, ScD

Rush University
Robert A. Weinstein, MD
Mary Hayden, MD

Funded by National Institute of Allergy and Infectious Diseases

Study Administrative Team

University of California Irvine

Victoria Ngo, BS, Assistant Clinical Research Coordinator

Harvard Medical School/Harvard Pilgrim Health Care Institute

Taliser Avery, MS, Lead Research Analyst

Micaela Coady, MS, Senior Program Manager

Allison Froman, MPH, Research Analyst

Selsebil Sljivo, MPH, Senior Project Coordinator

Neha Varma, MPH, Senior Research Analyst

Chamaine Washington, MPH, Project Coordinator

HCA Healthcare

Thomas Johnson, Senior MEDITECH Programmer & NPR Report Writer

Laura McLean, M.Ed, Senior Data Analyst

Brittany Meador, BS, Program Coordinator, Extramural Collaborations & Partnerships

Naoise Nickolay, RPh, Pharmacy Informatics Consultant

Kimberly Smith, MBA, Director, Research Operations

Funded by National Institute of Allergy and Infectious Diseases

Contact Matrix

Topic	What To Do	Who To Contact	Contact Information
**Study and Protocol Related Questions**	Call or Email	**Victoria Ngo**<br>**Seby Sljivo**	HCAINSPIRE@gmail.com<br>(844) 522-4677
**Pharmacy**	Email	**Chenette Burks, PharmD**<br>Director, Clinical Pharmacy Services<br><br>**Kaleb Roemer, PharmD**<br>Manager, Clinical Pharmacy Services	Ruby.Burks@hcahealthcare.com<br><br>Kaleb.Roemer@hcahealthcare.com
**IT**	Email	**Risa Rahm, PharmD CPHIMS**<br>Senior Director<br>Clinical Pharmacy Informatics	Risa.Rahm@hcahealthcare.com
**IRB Questions**	Call or Email	**Seby Sljivo**	HCAINSPIRE@gmail.com<br>(844) 522-4677

***Do not disclose protected health information (PHI) to INSPIRE Study Staff. If you need to share PHI, we will direct you to HCA Healthcare Study Investigators.**

For questions related to HCA Healthcare (HCA) hospital policy and processes:

HCA Investigator	Phone Number	Email
**Kenneth Sands, MD MPH**<br>VP, Chief Epidemiologist and Patient Safety Officer	(615) 344-1123	Kenneth.Sands@hcahealthcare.com
**Russell Poland, PhD**<br>AVP, Research and<br>Extramural and Collaborative Partnerships	(615) 344-2330	Russell.Poland@hcahealthcare.com

Funded by National Institute of Allergy and Infectious Diseases

INSPIRE Trials: Arm 2

Study Champion Materials

This section includes:

- Study Champion Roles and Responsibilities
- Study Champion Intervention Checklist
- Study Champion FAQs
- Smart Prompt Implementation Protocol

➢

Study Champion Roles and Responsibilities

Pharmacy & Physician Study Champions will partner with the INSPIRE investigative team and are expected to:

1. Join trial coaching calls
2. Actively engage and educate hospital clinicians, leadership, and other stakeholders on the value and safety of the INSPIRE Trials
3. Develop and implement strategies to educate and disseminate study materials to reach all prescribers at your facility
4. Review clinician progress reports and encourage reductions in antibiotic use
5. Avoid and/or report interventions or activities targeting empiric antibiotic selection for abdominal and skin/soft tissue infection that may impact trial outcomes

Funded by National Institute of Allergy and Infectious Diseases

INSPIRE Study Champion Intervention Checklist

As Study Champion for the INSPIRE Abdominal & Skin/Soft Tissue (SST) Trials, there are several key things you can do to ensure the INSPIRE Prompt launches smoothly:

Engage key leaders & stakeholders to help educate their peers

- ☐ Hospitalist, ED, Surgery (General, Trauma, Vascular, OB/GYN) leaders
- ☐ Graduate Medical Education Program Directors
- ☐ Pharmacy (e.g., managers, directors)
- ☐ Other prescribers (Physician Assistants, Nurses, Nurse Practitioners, Pharmacists)

Get the word out and educate frontline clinicians/prescribers

- ☐ Present INSPIRE at medical staff/faculty meetings or grand rounds
- ☐ Disseminate educational materials:
    - ☐ Flyers, posters, one-page INSPIRE summary in physician lounges
    - ☐ "Meet-and-greet" clinicians on the floors with handouts, FAQs
    - ☐ Encourage clinicians attend study webinars or access information on Atlas/Media Connect

Assure existing order sets are aligned with INSPIRE prompts

- ☐ Assess sepsis, abdominal infection, and SST infection order sets for potential conflicts or redundancies with INSPIRE CPOE prompts
    - ☐ Work with local and corporate IT to resolve as appropriate
- ☐ Examples include:
    - ☐ Sepsis order set contains only extended-spectrum (ES) antibiotic options for abdominal/SST infections. CPOE smart prompt alert will recommend ceftriaxone when MDRO risk is low. This may be seen by clinicians as a potentially conflicting recommendation.
    - ☐ **Possible solution(s):** (a) Add ceftriaxone to list of antibiotics in sepsis order sets. (b) Make two separate order sets, one for ICU and one for non-ICU patients since the smart prompt only alerts for non-ICU patients

Identify and educate other clinicians involved in antibiotic ordering

- ☐ Assess whether antibiotics are commonly given as verbal orders and if so, which staff take verbal orders (e.g., pharmacists, nurses, physician assistants)
- ☐ If appropriate, include leaders and staff from these areas in your education efforts using INSPIRE study materials
    - ☐ The "Verbal Order Checklist" in this toolkit provides an overview of questions to ask physicians when taking verbal orders for ES antibiotics, including a suggested script to use

Give clinicians feedback on antibiotic use

- ☐ Regularly review Tableau reports on physician antibiotic use and override reasons
- ☐ Look for physician prescribing patterns for sepsis, abdominal, and skin/soft tissue infections and education opportunities
- ☐ Share reports with leadership, stewardship/other pharmacy and physician staff
- ☐ Provide direct physician education and feedback.
  For example:
    - ☐ Send physicians emails with their individual ES antibiotic use monthly
    - ☐ Bring physician-specific antibiotic use reports with you when rounding on the floors to provide direct verbal feedback
    - ☐ Identify and address education opportunities that become apparent after review of override reasons or conversations with physicians

Contact study investigators for assistance

Always feel free to contact investigators for help in addressing clinician questions

INSPIRE Study Team (Research Coordinator – Victoria Ngo), UC Irvine
HCAINSPIRE@gmail.com, 949-824-7806

Lead Investigator – Shruti Gohil, MD, MPH, UC Irvine
skgohil@hs.uci.edu

Co-Investigator – Edward Septimus, MD, FACP, Senior Lecturer HPHCI
eseptimus@gmail.com

Manager, Clinical Pharmacy Services – Kaleb Roemer, PharmD
Kaleb.Roemer@hcahealthcare.com

Director, Clinical Pharmacy Services – Chenette Burks, PharmD
Ruby.Burks@hcahealthcare.com

Funded by National Institute of Allergy and Infectious Diseases

INSPIRE Abdominal and Skin & Soft Tissue Infection Trials

Study Champion FAQs

What are the INSPIRE-ASP Trials?

The INSPIRE-ASP (**IN**telligent **S**tewardship **P**rompts to **I**mprove **R**eal-time **E**mpiric **A**ntibiotic **S**election for **P**atients) Trials are two separate cluster-randomized trials of HCA Healthcare hospitals to test an antibiotic stewardship intervention abdominal (ABD) and skin and soft tissue (SST) infections.

- **Arm 1 – Routine Care**
- **Arm 2 – CPOE Smart Prompt Alert Intervention**
    1. Provides patient-specific risk estimate for ABD or SST infections due to a multi-drug resistant organism (MDRO) when extended-spectrum (ES) antibiotics are ordered
    2. Recommends standard antibiotics in non-ICU patients with low (<10%) MDRO risk

When will the computerized physician order entry (CPOE) smart prompt fire?

- **Prompts fire when ES antibiotics are ordered within 3 days ofadmission**
- Only for **adults** in **ED or non-intensive care units (ICU)** admitted for **ABD or SST infections**
- Risk of antibiotic resistant infection is calculated & recorded using **real-time, patient-specific data**
    - **If ≥ 10%**, **extended-spectrum antibiotic allowed**
    - **If <10%**, **prompt recommends standard antibiotics**

Which antibiotics are considered extended-spectrum?

Category	Extended-Spectrum Antibiotics for INSPIRE CPOE Alerts[1]
**Anti-MRSA**	Ceftaroline, Daptomycin, Linezolid, Vancomycin[2]
**Anti-VRE**	Daptomycin, Linezolid
**Anti-Pseudomonal**	Aztreonam, Cefepime, Ceftazidime, Piperacillin/Tazobactam
**Anti-ESBL**	Ertapenem, Imipenem, Meropenem, Ceftolozane/Tazobactam

[1]Additional restricted antibiotics will not activate INSPIRE alerts, [2]IV formulation only

How is the INSPIRE CPOE prompt "smart"?

The CPOE smart prompt uses your patient's risk factors (from medical record data) to calculate MDRO risk for abdominal or skin/soft tissue infection. Risk estimates are tailored to the antibiotic selected. Example:

- If **ceftriaxone** ordered → **no prompt** fires at all (standard-spectrum antibiotic)
- If **vancomycin** ordered → **returns patient's MRSA risk**
- If cefepime or **piperacillin/tazobactam** ordered → returns ***Pseudomonas* risk**
- If **linezolid** ordered → returns **MRSA and VRE risk**
- If **meropenem** ordered → returns **ESBL + resistant gram-negative bacteria**

What is the study timeline?

- **Prompt go-live** – August 2, 2022
- **Phase-In** (5 months) – August-December, 2022
- **Intervention** (12 months) – January-December, 2023

What are the outcomes of the INSPIRE-ASP Trials

The goal is to identify a best practice strategy for choosing empiric (within 3 calendar days) antibiotics based on a patient's risk for MDRO infection. Outcomes include:

- **Primary**: Empiric extended-spectrum days of therapy
- **Secondary**: Empiric Vancomycin (ABD infection only) and antipseudomonal days of therapy
- **Safety outcomes**: length of stay, ICU transfers
- **Other**: post-empiric ES antibiotic use, MDRO and *C. difficile* rates, antibiotic costs

Which patients are included?

Only adults admitted to a non-ICU ward with ABD or SST infection who are ordered to receive an extended-spectrum antibiotic within 3 days of admission will be included in the smart prompt alerts. The INSPIRE smart prompts are programmed to not fire for patients younger than 18 years or who are in an ICU location. Patients admitted from jail or prison will not be included in this study.

How does the INSPIRE CPOE prompt exclude ICU patients?

1. Does not fire if a patient is located in the ICU.
2. For emergency department, smart prompt asks clinicians if the patient is ICU-bound and does not fire if clinician responds "yes".
3. If smart prompt fires and patient is going to the ICU, clinicians can override theprompt.

What are the INSPIRE CPOE risk estimates based on?

The INSPIRE study team assessed over 422,000 patients admitted to HCA Healthcare hospitals over 3 years. We evaluated **(1) your hospital antibiogram** for ABD or SST infection patients and **(2) patient risk factors of MDRO infection**, including demographics (age, gender, race, ethnicity, insurance status), comorbid conditions, history of MDRO, history of prior surgery, history of prior HCA hospital, ED, or nursing home admission, prior antibiotics, and admission lab values.

Using a special statistical method, we identified which risk factors were most often found in patients who developed ABD or SST infection with an MDRO. Using these risk factors (which are different for every MDRO), the CPOE smart prompt gives the patient's **individual absolute risk** for infection with the MDRO(s) targeted by the antibiotic ordered.

What does the INSPIRE CPOE prompt account for at the moment of order entry?

The prompt can only account for information available in your hospital's Meditech system at the time of order entry. **The prompt can capture**:

- **History of MDRO** from microbiology data as far back as available in Meditech through 2012
- **Cultures resulted in Meditech at your facility 3 or more days ago**

The prompt cannot capture:

- MDRO history or cultures from an **outside hospital**
- **Culture results < 3 days** from admission
    - Monitor all new cultures as they become available and adjust accordingly

Will risk estimates be validated after the intervention starts?

The INSPIRE risk estimates models are robust and based on 3 years of data across the HCA Healthcare hospital enterprise. Estimates for a single facility are unlikely to change significantly within 1 year. Our evaluation also shows that facility MDRO prevalence appears stable over the short term. Risk estimates will be re-validated periodically as needed.

What if someone takes a verbal order for antibiotics?

The INSPIRE prompt fires in the following CPOE modules within Meditech: "OM" (Order Management), "EDM" (Emergency Department Management), and PWM (Provider/Physician Workflow Management). **It will NOT fire in the "PHA" (Pharmacy) module.** We ask all staff taking verbal orders (including "vancomycin per pharmacy dosing" orders) to place them using OM, EDM, or PWM modules. Staff taking verbal orders will also need pertinent information from physicians to be able to respond to the INSPIRE prompt, such as (1) what infection the antibiotic is meant to treat and (2) whether the physician would agree to standard-spectrum antibiotics if the risk is found to be low (<10%). Please see the "Verbal Order Checklist for Pharmacists and Nurses" which guides how verbal orders are best handled.

Why does INSPIRE focus on ABD and SST infections?

ABD and SST infections are among the most common reasons for infection-related admission and ES antibiotic use. Yet, few studies have evaluated effective empiric antibiotic stewardship strategies for these infections.

What will be required of participating sites?

Please reference the "Study Champion Intervention Checklist" of key things you can do to ensure the INSPIRE CPOE smart prompt launches successfully at your facility. Study champions will be asked to:

- Join trial coaching calls
- Engage and educate clinicians on value and safety of the INSPIRE-ASPTrials
- Review clinician quarterly progress reports and encourageimprovements
- Address clinician outliers in a supportive, knowledgeable way
- Set reachable local targets and improvement goals
- Report interventions or activities that may impact trial outcomes

How do we avoid competing interventions and how are these defined?

Participating facilities are fully expected to continue their stewardship activities and initiatives but should avoid activities that may directly impact trial outcomes. Such activities are anticipated to be rare. Examples of interventions that may directly conflict with the trial include:

- A new stewardship strategy that aims to reduce the use of ES antibiotics in the Emergency Department (ED) or within 3 days of admission trial coaching calls
- A new real-time antibiotic de-escalation strategy in the ED/within 3 calendar days
- A new rapid diagnostic tool for ABD or SST infection that would be used in the empiric period to guide antibiotic therapy within 3 days of admission
- Ordering antibiotics through Patient Keeper

Will INSPIRE impact ED sepsis core measures?

The INSPIRE prompt **does NOT** affect "time to antibiotics" because it does not impact whether antibiotics should be used but rather gives real-time information to help decide whether extended- or standard-spectrum antibiotics should be used. The standard-spectrum antibiotics in the INSPIRE prompts are fully compliant with core measures.

Has this study received IRB approval? Will patients need to give informed consent?

Yes. Harvard Pilgrim's IRB will provide ethical oversight for these trials and serve as the central IRB. Both INSPIRE ABD and SST infection trials have been approved by the Harvard IRB. Your hospital has submitted all ceding and reliance agreements. Informed consent will not be required. This study has been approved as a minimal risk study and will be implemented as a quality improvement (QI) protocol through the existing infrastructure of your hospital's antibiotic stewardship programs. The assigned protocol will be approved through your usual clinical/leadership committees and become hospital guidance on appropriate empiric antibioticuse.

Who can I contact for more information?

INSPIRE Study Team
Research Coordinator – Victoria Ngo, UC Irvine
HCAINSPIRE@gmail.com, 949-824-7806

Lead Investigator – Shruti Gohil, MD, MPH, UC Irvine
skgohil@hs.uci.edu

Co-Investigator – Edward Septimus, MD, FACP, Senior Lecturer HPHCI
eseptimus@gmail.com

Manager, Clinical Pharmacy Services – Kaleb Roemer, PharmD
Kaleb.Roemer@hcahealthcare.com

Director, Clinical Pharmacy Services – Chenette Burks, PharmD
Ruby.Burks@hcahealthcare.com

Funded by National Institute of Allergy and Infectious Diseases

INSPIRE Smart Prompt Implementation Protocol

The **INSPIRE Abdominal and Skin/Soft Tissue (ABD/SST) trials** use a computerized physician order entry (CPOE) alert to provide clinicians with (1) patient-specific risk estimates for infection due to an antibiotic-resistant bacteria and (2) recommendations on the standard-spectrum empiric antibiotic selection based on individualized risk estimates. This intervention will improve the use of extended-spectrum (ES) antibiotics for non-intensive care unit (ICU) patients at low risk of having abdominal or skin/soft tissue infection due to an antibiotic-resistant bacteria.

This document provides pharmacists and clinicians with an overview of the protocol by which antibiotic selection should occur when INSPIRE CPOE alerts are activated at your facility.

Empiric Antibiotic Selection Process

1. **The CPOE alert is activated when:**
   a. Clinicians order an extended-spectrum antibiotic(s) for abdominal (ABD) or skin/soft tissue infection (SST) in non-ICU patients within the first 3 days of admission, including in the emergency department.
   b. No prompt will be triggered if standard-spectrum antibiotics are ordered (e.g. antibiotics that are not listed in Table 1, such as ceftriaxone or ampicillin/sulbactam.
2. **The CPOE alert provides clinicians with patient-specific risk estimates for antibiotic-resistant infection.**
   a. Risk estimates are calculated for the antibiotic-resistant organism(s) targeted by the antibiotic ordered (Table 1).
      i. Example 1: When a clinician orders vancomycin for ABD infection, the CPOE alert will calculate the risk that the patient has ABD infection due to MRSA.
      ii. Example 2: When a clinician orders cefepime for SST infection, the CPOE alert will calculate the risk that the patient has SST infection due to *Pseudomonas*.
      iii. Example 3: When a clinician orders linezolid for SST infection, the CPOE alert will calculate the risk that the patient has SST infection due to MRSA or VRE.
      iv. Example 4: When a clinician orders meropenem for ABD infection, the CPOE alert will calculate the risk that the patient has ABD infection due to ESBL or other resistant gram-negative bacteria.
3. **Clinicians will be given recommendations on the appropriateness of extended-spectrum antibiotics based upon the patient's calculated risk.**
   a. If the patient's risk for antibiotic-resistant ABD or SST infection is <10%, then the risk is considered low and the CPOE alert will recommend ceftriaxone (abdominal infection) and cefazolin (skin/soft tissue infections) as the standard empiric antibiotic. Other standard-spectrum antibiotic can be used to treat low risk patients as per physician preferences.

b. If the patient's risk for antibiotic-resistant ABD or SST infection is >10%, the prompt will automatically allow completion of the ES antibiotic order without any prompts.
c. CPOE recommendations are **not intended to supplant clinician judgment**. Patients who are neutropenic, decompensating, or otherwise at high risk for complications or drug resistant infections should be treated as clinically appropriate.
d. Clinicians will always have the option to override the CPOE alert recommendation after indicating the reason for the override.

4. Prescriber acceptance with CPOE alert recommendations will be monitored by the stewardship team/study champions.

a. Clinician Feedback reports, including the number and reason for CPOE recommendation overrides, will be provided to study champions.
b. Study champions will review acceptance and provide prescribers with written and/or verbal feedback.

Table 1: Extended-Spectrum Antibiotics That Will Trigger INSPIRE CPOE Alerts

Category	Extended-Spectrum Antibiotics for INSPIRE CPOE Alerts[1]
**Anti-MRSA**	Ceftaroline, Daptomycin, Linezolid, Vancomycin[2]
**Anti-VRE**	Daptomycin, Linezolid
**Anti-Pseudomonal**	Aztreonam, Cefepime, Ceftazidime, Piperacillin/Tazobactam
**Anti-ESBL**	Ertapenem, Imipenem, Meropenem, Ceftolozane/Tazobactam

[1]Additional restricted antibiotics will not activate INSPIRE alerts, [2]IV formulation only
Note: INSPIRE smart prompt excludes the following routes of administration: inhaled, otic, ophthalmic, or irrigation.
Abbreviations: MRSA - Methicillin Resistant *Staphylococcus aureus;* **VRE –** Vancomycin Resistant *Enterococci;* **ESBL –** Extended-Spectrum Beta Lactamase producer

INSPIRE Trials: Arm 2

Clinician Education Materials

This section includes:

- INSPIRE Trials One-Page Summary
- Clinician FAQs with Prompt Screenshots
- Clinician FAQs (no Prompt Screenshots)
- Smart Prompt “Top 5” Reminders for Clinicians
- Extended-Spectrum Antibiotics List
- Verbal Order Checklist for Pharmacists and Nurses

Funded by National Institute of Allergy and Infectious Diseases

INSPIRE-ASP Abdominal & Skin/Soft Tissue Trials

INtelligent Stewardship Prompts to Improve Real-time Empiric Antibiotic Selection for Patients

The **INSPIRE-ASP Trials** are a set of two large cluster-randomized trials to improve antibiotic stewardship for non-ICU patients with abdominal (ABD) and skin & soft tissue (SST) infection in HCA Healthcare (HCA) hospitals. Participating hospitals are randomized to:

- **Arm 1 – Routine Care**
- **Arm 2 – Smart Prompt -** Precision medicine smart prompt using a computerized physician order entry (CPOE) alert that gives a ***patient-specific risk estimate of antibiotic resistant infection*** and recommends standard-spectrum antibiotics in patients admitted for ABD or SST infection who are calculated to have low risk (<10%) for antibiotic resistant infection.

Rationale

- Prior INSPIRE Pneumonia & UTI Trials reduced unnecessary extended-spectrum (ES) antibiotics by 30%

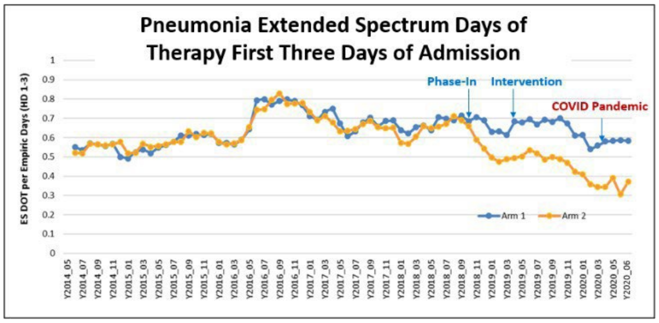

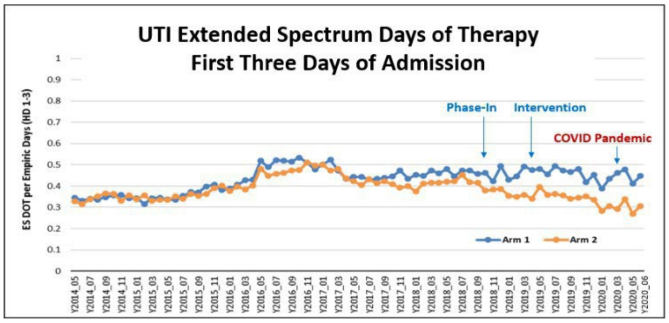

CONFIDENTIAL for HCA internal use ONLY – Preliminary results, do not cite or distribute

- Most inpatients with ABD or SST infection do not benefit from ES antibiotics.
- Smart prompt reduces ES use by documenting low risk patients and encouraging standard antibiotics that have fewer adverse effects (e.g. kidney injury, future antibiotic resistance, *C. difficile*)

CPOE Details

- MDRO risk estimates are based on **patient-specific** risk factors and **local** antibiograms.
- Demographics, comorbidities, MDRO history, prior antibiotics or nursing home stays, and multiple lab values from 422,000 HCA patients helped create the prediction algorithm.
- Antibiotic recommendations are based on MDRO risk estimates and approved by the trial steering committee, national experts, and HCA leadership.
- Recommendations support proper antibiotic choice but do not replace clinical judgment.

Outcomes

- Primary: Empiric use of ES antibacterial (first 3 days of admission)
- Secondary: Empiric use of (a) vancomycin and (b) antipseudomonal antibacterial
- Safety: ICU transfer and length-of-stay
- Other Outcomes: *C. difficile* infection, MDRO prevalence, cost-analysis

Timeline

- Phase In: Aug 2022 – Dec 2022 (5 months); Intervention: Jan 2023 – Dec 2023 (12 months)

QUESTIONS? Study Investigators & Coordinators: HCAINSPIRE@gmail.com; (844)-522-4677

INSPIRE Abdominal & Skin/Soft Tissue Infection Trials
Clinician FAQs with Prompt Screenshots

What are the INSPIRE-ASP Trials?

The INSPIRE-ASP (**IN**telligent **S**tewardship **P**rompts to **I**mprove **R**eal-time **E**mpiric **A**ntibiotic **S**election for **P**atients) Trials are two cluster-randomized trials of HCA Healthcare hospitals to test an antibiotic stewardship intervention for abdominal (ABD) and skin and soft tissue (SST) infections.

- **Arm 1** – **Routine Care**
- **Arm 2** – **CPOE Smart Prompt Alert Intervention**
  1. Provides patient-specific risk estimate for ABD or SST infections due to a multidrug-resistant organism (MDRO) when extended-spectrum (ES) antibiotics are ordered
  2. Recommends standard antibiotics in non-ICU patients with low MDRO risk

When will the computerized physician order entry (CPOE) smart prompt fire?

- **Prompts fire when ES antibiotics are ordered within 3 days ofadmission**
- Only for **adults** in **ED or non-intensive care units (ICU)** admitted for **ABD or SST infections**
- Risk of antibiotic resistant infection is calculated & recorded using **real-time, patient-specific data**
  - **If ≥ 10%**, **extended-spectrum antibiotic allowed**
  - **If <10%**, **prompt recommends standard antibiotics**

Which antibiotics are considered extended-spectrum?

Category	Extended-Spectrum Antibiotics for INSPIRE CPOE Alerts[1]
**Anti-MRSA**	Ceftaroline, Daptomycin, Linezolid, Vancomycin[2]
**Anti-VRE**	Daptomycin, Linezolid
**Anti-Pseudomonal**	Aztreonam, Cefepime, Ceftazidime, Piperacillin/Tazobactam
**Anti-ESBL**	Ertapenem, Imipenem, Meropenem, Ceftolozane/Tazobactam

[1]Additional restricted antibiotics will not activate INSPIRE alerts, [2]IV formulation only

How is the INSPIRE CPOE prompt "smart"?

The smart prompt uses your patient's risk factors to calculate MDRO infection risk. For example:

- If **ceftriaxone** ordered → **no prompt** fires at all (standard-spectrum antibiotic)
- If **vancomycin** ordered → **returns patient's MRSA risk**
- If cefepime or **piperacillin/tazobactam** ordered → returns ***Pseudomonas* risk**
- If **linezolid** ordered → returns **MRSA and VRE risk**
- If **meropenem** ordered → returns **ESBL + resistant gram-negative bacteria**

What does the INSPIRE CPOE prompt account for at the moment of order entry?

The prompt can only account for information available in your hospital's Meditech system at the time of order entry. **The prompt can capture**:

- **History of MDRO** from microbiology data as far back as available in Meditech through 2012
- **Cultures resulted in Meditech at your facility 3 or more days ago**

The prompt cannot capture:

- MDRO history or cultures from an **outside hospital**
- **Culture results < 3 days** from admission
  - Monitor all new cultures as they become available and adjust accordingly

What are the INSPIRE CPOE risk estimates based on?

We assessed over 422,000 patients admitted to HCA Healthcare hospitals over 3 years. We evaluated **(1) your hospital antibiogram** for ABD or SST infection patients and **(2) patient risk factors of MDRO infection**, including demographics (age, gender, race, ethnicity, insurance status), comorbid conditions, history of MDRO, prior surgery, prior HCA hospitalization, ED, or nursing home admission, prior antibiotics, and admission lab values. Using a special statistical method, we identified which risk factors were most often found in patients with ABD or SST MDRO infection. Using these risk factors (which are different for every MDRO), the CPOE smart prompt gives the patient's **individual absolute risk** for infection with the MDRO(s) targeted by the antibiotic ordered.

How does the INSPIRE CPOE prompt exclude ICU patients?

1. Does not fire if a patient is located in the ICU.
2. For emergency department, smart prompt asks clinicians if the patient is ICU-bound and does not fire if clinician responds "yes".
3. If smart prompt fires and patient is going to the ICU, clinicians can override theprompt.

Will INSPIRE impact ED sepsis core measures?

The INSPIRE prompt **does NOT** affect "time to antibiotics" because it does not impact whether antibiotics should be used but rather gives real-time information to help decide whether extended-spectrum or standard-spectrum antibiotics should be used. The standard-spectrum antibiotics in the INSPIRE prompts are fully compliant with core measures.

What does the INSPIRE smart prompt intervention look like?

In the below example smart prompt screen, a clinician ordered Vancomycin for abdominal infection. Patient risk for MRSA abdominal infection is calculated to be <10%.

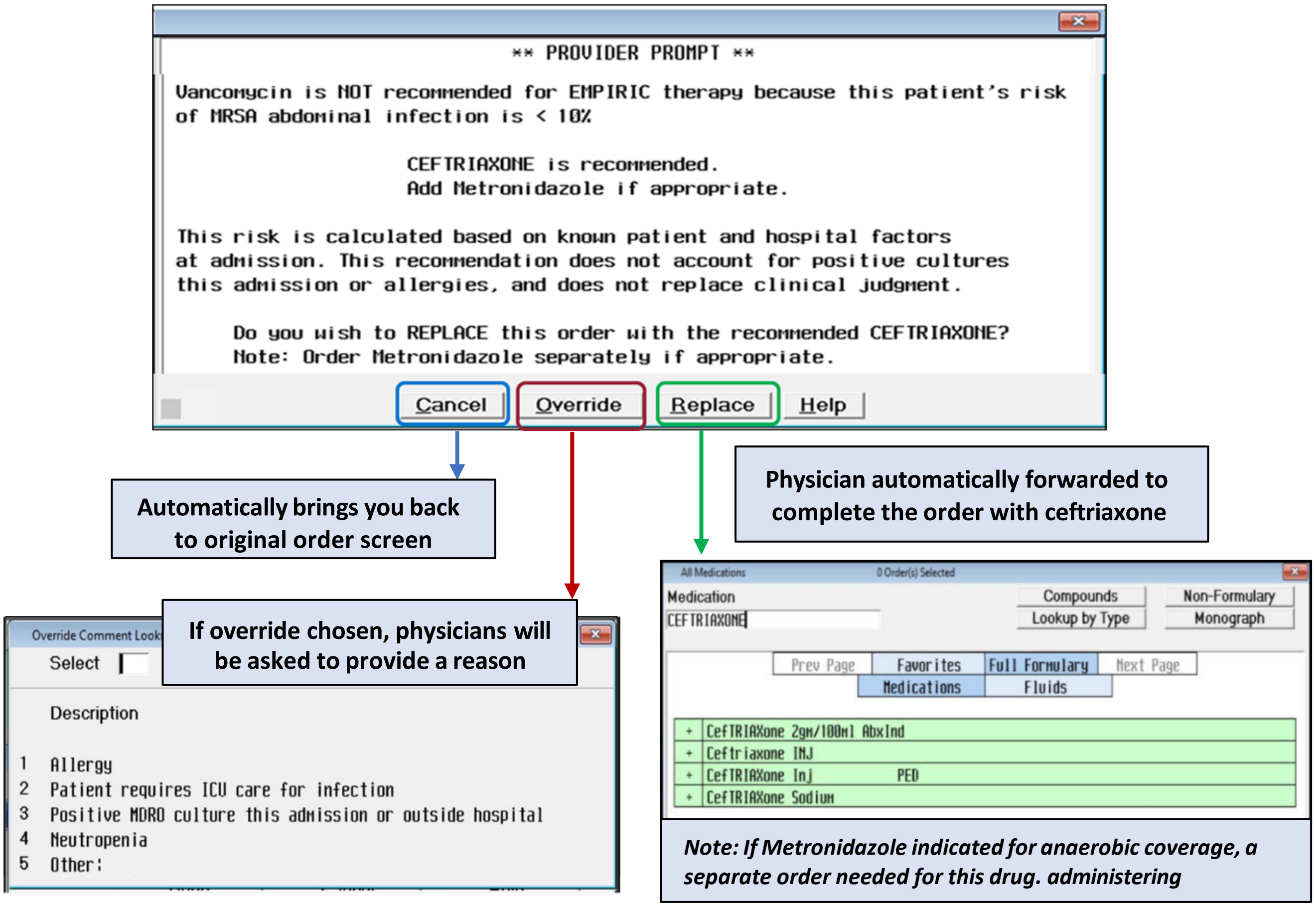

INSPIRE Abdominal and Skin & Soft Tissue Infection Trials
Clinician FAQs

What are the INSPIRE-ASP Trials?

The INSPIRE-ASP (**IN**telligent **S**tewardship **P**rompts to **I**mprove **R**eal-time **E**mpiric **A**ntibiotic **S**election for **P**atients) Trials are two separate cluster-randomized trials of HCA Healthcare hospitals to test an antibiotic stewardship intervention abdominal (ABD) and skin and soft tissue (SST) infections.

- **Arm 1 – Routine Care**
- **Arm 2 – CPOE Smart Prompt Alert Intervention**
    1. Provides patient-specific risk estimate for ABD or SST infections due to a multidrug-resistant organism (MDRO) when extended-spectrum (ES) antibiotics are ordered
    2. Recommends standard antibiotics in non-ICU patients with low (<10%) MDRO risk

When will the computerized physician order entry (CPOE) smart prompt fire?

- **Prompts fire when ES antibiotics are ordered within 3 days ofadmission**
- Only for **adults** in **ED or non-intensive care units (ICU)** admitted for **ABD or SST infections**
- Risk of antibiotic resistant infection is calculated & recorded using **real-time, patient-specific data**
    - **If ≥ 10%**, **extended-spectrum antibiotic allowed**
    - **If <10%**, **prompt recommends standard antibiotics**

Which antibiotics are considered extended-spectrum?

Category	Extended-Spectrum Antibiotics for INSPIRE CPOE Alerts[1]
**Anti-MRSA**	Ceftaroline, Daptomycin, Linezolid, Vancomycin[2]
**Anti-VRE**	Daptomycin, Linezolid
**Anti-Pseudomonal**	Aztreonam, Cefepime, Ceftazidime, Piperacillin/Tazobactam
**Anti-ESBL**	Ertapenem, Imipenem, Meropenem, Ceftolozane/Tazobactam

[1]Additional restricted antibiotics will not activate INSPIRE alerts, [2]IV formulation only

How is the INSPIRE CPOE prompt "smart"?

The CPOE smart prompt uses your patient's risk factors (from medical record data) to calculate MDRO risk for abdominal or skin/soft tissue infection. Risk estimates are tailored to the antibiotic selected. Examples:

- If **ceftriaxone** ordered → **no prompt** fires at all (standard-spectrum antibiotic)
- If **vancomycin** ordered → **returns patient's MRSA risk**
- If cefepime or **piperacillin/tazobactam** ordered → returns ***Pseudomonas* risk**
- If **linezolid** ordered → returns **MRSA and VRE risk**
- If **meropenem** ordered → returns **ESBL + resistant gram-negative bacteria**

How does the INSPIRE CPOE prompt exclude ICU patients?

1. Does not fire if a patient is located in the ICU.
2. For emergency department, smart prompt asks clinicians if the patient is ICU-bound and does not fire if clinician responds "yes".

3. If smart prompt fires and patient is going to the ICU, clinicians can override theprompt.

What are the INSPIRE CPOE risk estimates based on?

The INSPIRE study team assessed over 422,000 patients admitted to HCA Healthcare hospitals over 3 years. We evaluated **(1) your hospital antibiogram** for ABD or SST infection patients and **(2) patient risk factors of MDRO infection**, including demographics (age, gender, race, ethnicity, insurance status), comorbid conditions, history of MDRO, history of prior surgery, history of prior HCA hospital, ED, or nursing home admission, prior antibiotics, and admission lab values.

Using a special statistical method, we identified which risk factors were most often found in patients who developed ABD or SST infection with an MDRO. Using these risk factors (which are different for every MDRO), the CPOE smart prompt gives the patient's **individual absolute risk** for infection with the MDRO(s) targeted by the antibiotic ordered.

What does the INSPIRE CPOE prompt account for at the moment of order entry?

The prompt can only account for information available in your hospital's Meditech system at the time of order entry. **The prompt can capture**:

- **History of MDRO** from microbiology data as far back as available in Meditech through 2012
- **Cultures resulted in Meditech at your facility 3 or more days ago**

The prompt cannot capture:

- MDRO history or cultures from an **outside hospital**
- **Culture results < 3 days** from admission
    - Monitor all new cultures as they become available and adjust accordingly

Will INSPIRE impact ED sepsis core measures?

The INSPIRE prompt **does NOT** affect "time to antibiotics" because it does not impact whether antibiotics should be used but rather gives real-time information to help decide whether extended-spectrum or standard-spectrum antibiotics should be used. The standard-spectrum antibiotics in the INSPIRE prompts are fully compliant with core measures.

Will patients need to give informed consent?

No. This study has been approved as a minimal risk study and will be implemented as a quality improvement (QI) protocol through the existing infrastructure of your hospital's antibiotic stewardship programs. The assigned protocol will be approved through your usual clinical/leadership committees and become hospital guidance on appropriate empiric antibioticuse.

Who can I contact for more information?

INSPIRE-ASP Study Team:
HCAINSPIRE@gmail.com
(844) 522-4677

Funded by National Institute of Allergy and Infectious Diseases

INSPIRE Abdominal & Skin/Soft Tissue Trials

Smart Prompt "Top 5" Reminders for Clinicians

Top 5 Things Providers Should Know About the INSPIRE Prompts

1. INSPIRE **smart prompts calculate your patient's risk** for antibiotic resistant infection using real-time, patient-specific data
   - **If ≥ 10%, extended-spectrum (ES) antibiotic allowed**
   - **If <10%, prompts for standard antibiotics**
2. Risk estimates are **recorded in Meditech and give documentation** to justify use of standard-spectrum antibiotics when risk is low enough to await cultures
3. Prompt **cannot account for information that is not in Meditech**:
   - History of drug resistant bacteria or culture results from an outside hospital
   - Culture results finalized in the past 3 days in your hospital
   - Neutropenia
   - Allergy
4. **You will get feedback on antibiotic use and prompt acceptance** to help you minimize ES antibiotic use
5. The INSPIRE Pneumonia and UTI trials **achieved up to 30% reduction in ES use, without prolonging length-of-stay and without increasing ICU transfers**

Please contact your Antibiotic Stewardship Team for questions or details

Study Investigators & Coordinators
(844) 522-4677
HCAINSPIRE@gmail.com

Funded by National Institute of Allergy and Infectious Diseases

Extended-Spectrum Antibiotics List

Which Antibiotics Are Considered Extended-Spectrum?

Antibiotics with activity against extensive types of bacteria, including multidrug-resistant organisms (MDROs), are considered "extended-spectrum" (ES) antibiotics. Choosing ES antibiotics increases a patient's risk of *C. difficile*, future MDROs, and adverse drug effects (e.g., renal or liver insufficiency). The goal of the INSPIRE Abdominal and Skin/Soft Tissue Trials is to decrease ES antibiotic use and improve patient safety.

The antibiotics in **Table 1** are considered ES antibiotics in the INSPIRE trials and will activate the INSPIRE computerized physician order entry prompt (CPOE) when ordered for abdominal or skin/soft tissue infections in the emergency department or non-intensive care units within 3 days of admission.

The INSPIRE prompt does not trigger for restricted antibiotics that require prior approval or are non-formulary.

Table 1: Extended-Spectrum Antibiotics That Will Trigger INSPIRE CPOE Alerts

Category	Extended-Spectrum Antibiotics for INSPIRE CPOE Alerts[1]
**Anti-MRSA**	Ceftaroline, Daptomycin, Linezolid, Vancomycin[2]
**Anti-VRE**	Daptomycin, Linezolid
**Anti-Pseudomonal**	Aztreonam, Cefepime, Ceftazidime, Piperacillin/Tazobactam
**Anti-ESBL**	Ertapenem, Imipenem, Meropenem, Ceftolozane/Tazobactam

[1]Additional restricted antibiotics will not activate INSPIRE alerts, [2]IV formulation only

Funded by National Institute of Allergy and Infectious Diseases

Verbal Order Checklist for Pharmacists and Nurses

Background

The INSPIRE CPOE smart prompt intervention encourages compliance with national guidance for standard-spectrum antibiotics (e.g., ceftriaxone for abdominal (ABD) and cefazolin for skin/soft tissue (SST) infections). Ordering extended-spectrum (ES) antibiotics (e.g., vancomycin, cefepime) for ABD or SST infections in the Emergency Department (ED) or non-Intensive Care Unit (ICU) wards within 3 days of admission will activate a computerized physician order entry (CPOE) smart prompt alert that will use data available in the patient's electronic medical record to provide:

1. Patient's individual risk for antibiotic resistant infection based on the patient's own data in your hospital's electronic medical record
2. Recommendation for standard-spectrum antibiotics when risk is low (<10%)

Use CPOE Meditech Modules When Placing Verbal Orders

The INSPIRE prompt fires in the following CPOE modules within Meditech: "OM" (Order Management), "EDM" (Emergency Department Management), and PWM (Provider/Physician Workflow Management). **It will NOT fire in the "PHA" (Pharmacy) module.** We ask all staff taking verbal orders (including "vancomycin per pharmacy dosing" orders) to place them using OM, EDM, or PWM modules. Staff taking verbal orders will also need pertinent information from physicians to be able to respond to the INSPIRE prompt, such as (1) what infection the antibiotic is meant to treat and (2) whether the physician would agree to standard-spectrum antibiotics if the risk is found to be low (<10%).

Taking Verbal Orders for Inpatient Extended-Spectrum Antibiotics

The INSPIRE CPOE prompts apply **ONLY to ES antibiotics** ordered on **non-ICU patients** hospitalized for **ABD or SST infection** within **3 days of admission**.

- The prompt does **NOT** fire for standard-spectrum antibiotics.
- A list of ES antibiotics is provided in Table 1 below.

When taking verbal orders for ES antibiotics, we recommend that you stay on the phone with the ordering clinician while entering orders. If you are unable to place the order when taking the verbal order, use the following questions to get the information you will need prior to placing the order:

1. **Check if patient has been <u>hospitalized for 4 or more days</u> OR is <u>in the ICU</u>? If yes to either →** *no further physician questions needed. CPOE will **NOT** fire.*

2. **Is patient in the Emergency Department?**
   a. **If in ED →** ask if patient is ICU-bound
      i. **If ICU-bound →** no further questions
      ii. **If <u>NOT</u> ICU-bound → proceed to Step 3**

3. **IF ES antibiotic being ordered within 3 days of admission:**
   a. **Ask prescriber what the indication is for the antibiotic.** Remind prescriber that indications are required for all antibiotic orders as part of HCA Healthcare's meaningful use stewardship initiative.
   b. If prescriber chooses any of the following indications, **proceed to step 4**:
      - Abdominal infections
      - Skin/soft tissue infections
      - Sepsis – due to ABD or SST infections
      - Empiric (unknown) source – possibly from an abdominal or skin/soft tissue source

4. **Remind prescriber that ES antibiotics in this patient will trigger the INSPIRE smart prompt and will give the patient's specific risk for infection due to multidrug-resistant organism (MDRO) or *Pseudomonas*. The following suggested script can be used or modified:**
   a. "Just a reminder, Dr., our hospital has an antibiotic selection protocol for abdominal and skin/soft tissue infections. The computer will calculate and document your patient's risk of an antibiotic-resistant ABD/SST infection that justifies an ES antibiotic. I will need to ask you a couple of questions so I can place the order."
   b. If ordering anti-MRSA or antipseudomonal for abdominal infection then: "Has the patient had abdominal surgery in the past year? This may affect the risk estimate calculation."
   c. "If the risk is low (<10%) then [ceftriaxone for abdominal infection; cefazolin for skin/soft tissue infection] will be recommended. Should I plan to follow this recommendation?"
      i. **If yes** → no further questions.
      ii. **If no** → ask the prescriber the reason for override:
         - Allergy
         - Neutropenia
         - Positive MDRO culture this admission or outside hospital
         - Patient expected to go to the ICU
         - Other (ask prescriber to specify)

Table 1: Extended-Spectrum Antibiotics That Will Trigger INSPIRE CPOE Alerts

Category	Extended-Spectrum Antibiotics for INSPIRE CPOE Alerts[1]
**Anti-MRSA**	Ceftaroline, Daptomycin, Linezolid, Vancomycin[2]
**Anti-VRE**	Daptomycin, Linezolid
**Anti-Pseudomonal**	Aztreonam, Cefepime, Ceftazidime, Piperacillin/Tazobactam
**Anti-ESBL**	Ertapenem, Imipenem, Meropenem, Ceftolozane/Tazobactam

[1]Additional restricted antibiotics will not activate INSPIRE alerts, [2]IV formulation only

INSPIRE Trials: Arm 2

Clinician Hot Topics

This section includes:

- How MDRO Risk Estimates Are Calculated
- Safety of Choosing Standard-Spectrum Empirically
- Clinician Feedback on Antibiotic Use

INSPIRE Abdominal and Skin & Soft Tissue Infection Trials
Hot Topic: How MDRO Risk Estimates Are Calculated

What are the INSPIRE Trials?
The INSPIRE (**IN**telligent **S**tewardship **P**rompts to **I**mprove **R**eal-time **E**mpiric Antibiotic Selection for Patients) Trials are two separate cluster-randomized trials of HCA Healthcare hospitals to test an antibiotic stewardship intervention for abdominal (ABD) and skin/soft tissue (SST) infections.

- **Arm 1 – Routine Care**
- **Arm 2 – CPOE Smart Prompt Alert Intervention**
  1. Provides patient-specific risk estimate for ABD or SST infections due to a multidrug-resistant organism (MDRO) when extended-spectrum (ES) antibiotics are ordered
  2. Recommends standard antibiotics in non-ICU patients with low (<10%) MDRO risk

When will the INSPIRE prompt fire?
- **Prompts fire when ES antibiotics are ordered within 3 days ofadmission**
- For **adults** in **ED or non-intensive care units (ICUs)** admitted for **ABD or SST infections**
- Risk of MDRO infection is calculated & recorded using **real-time, patient-specific data**
  - **If ≥ 10%**, **extended-spectrum antibiotic allowed**
  - **If <10%**, **prompt recommends standard antibiotics**

What are the INSPIRE risk estimates based on?
The INSPIRE study team assessed over 422,000 patients admitted to HCA Healthcare hospitals over 3 years. We evaluated **(1) the hospital antibiogram** for ABD or SST infection patients and **(2) patient risk factors of MDRO infection**, including demographics (age, gender, race, ethnicity, insurance status), comorbid conditions, history of MDRO, history of prior surgery, history of prior HCA hospital, emergency room, or nursing home admission, prior antibiotics, and admission lab values.

What statistical method was used to calculate the INSPIRE risk estimates?
The INSPIRE prompt gives the **absolute risk** for a patient to have an abdominal or skin/soft tissue infection due to an MDRO. To estimate absolute risk, we used a statistical method called recursive partitioning. This approach evaluates the study population by each patient risk factor repeatedly until it identifies the set of risk factors most commonly found in patients with ABD or SST infection due to an MDRO. This analysis was done separately for each MDRO and for each infection so that **risk estimates are specific to (1) your patient, (2) the MDRO targeted by the antibiotic ordered, and (3) the infection type**.

Why is it important that the INSPIRE prompt gives absolute risk?
Clinicians currently select antibiotics according to their perception of MDRO risk. This is often based on relative risks or odds ratios published in studies that single out selected patient risk factors. For example, if a patient has diabetes or is coming from a nursing home, we worry that they have higher risk for MDRO infection because of studies that show that such patients have an odds ratio of 2 or 3 for MDRO infection

compared to the general population. However **relative risks do not tell us anything about a patient's actual risk** for MDRO infection. To illustrate the problem with selecting antibiotics using relative risk, consider the following hypothetical scenario:

- Let's say a study reports females have a risk of 1 in 1,000 for ESBL abdominal infection and males have a risk of 3 in 1,000.
- That would be calculated as an odds ratio of 3 and interpreted by clinicians as 3-fold higher risk
- Yet, the absolute risk is actually only 0.3%

The INSPIRE trials use absolute risk because this is a more accurate assessment of individual risk.

How is the INSPIRE CPOE prompt "smart"?

The INSPIRE prompt uses your patient's risk factors (from medical record data) to calculate MDRO risk for abdominal or skin/soft tissue infection. Risk estimates are tailored to the antibiotic selected. Example:

- If **ceftriaxone** ordered → **no prompt** fires at all (standard-spectrum antibiotic)
- If **vancomycin** ordered → **returns patient's MRSA risk**
- If cefepime or **piperacillin/tazobactam** ordered → returns ***Pseudomonas* risk**
- If **linezolid** ordered → returns **MRSA and VRE risk**
- If **meropenem** ordered → returns **ESBL + resistant gram-negative bacteria**

What does the INSPIRE CPOE prompt account for at the moment of order entry?

The prompt can only account for information available in your hospital's Meditech system at the time of order entry. **The prompt can capture**:

- **History of MDRO** from microbiology data as far back as available in Meditech through 2012
- **Cultures resulted in Meditech at your facility 3 or more days ago**

The prompt cannot capture:

- MDRO history or cultures from an **outside hospital**
- **Culture results < 3 days** from admission
    - Monitor all new cultures as they become available and adjust accordingly

Will risk estimates be validated after the intervention starts?

The INSPIRE risk estimates models are robust and based on 3 years of data across the HCA Healthcare hospital enterprise. Estimates for a single facility are unlikely to change significantly within 1 year. Our evaluation also shows that facility MDRO prevalence appears stable over the short-term. Risk estimates will be re-validated periodically as needed.

Who can I contact for more information?

INSPIRE Research Coordinator – Victoria Ngo, UC Irvine - HCAINSPIRE@gmail.com, 949-824-7806

Lead Investigator – Shruti Gohil, MD, MPH, UC Irvine - skgohil@hs.uci.edu

Co-Investigator – Ed Septimus, MD, Senior Lecturer HPHCI - eseptimus@gmail.com

Manager, Clinical Pharmacy Services – Kaleb Roemer, PharmD - Kaleb.Roemer@hcahealthcare.com

Director, Clinical Pharmacy Services – Chenette Burks, PharmD - Ruby.Burks@hcahealthcare.com

Funded by National Institute of Allergy and Infectious Diseases

INSPIRE Abdominal and Skin & Soft Tissue Infection Trials

Hot Topic: Clinician Feedback on Antibiotic Use

Why is my antibiotic use being monitored and why is feedback important?

Extended-spectrum (ES) antibiotic overuse is a major patient safety concern because it puts patients at high risk for renal insufficiency, *C. difficile* colitis, future development of multidrug-resistant organisms, and adverse drug effects. Studies show that antibiotic stewardship interventions that provide clinicians with direct feedback on their antibiotic choices can reduce overuse by as much as 40%.

How will feedback be provided?

Your antibiotic stewardship team will generate antibiotic use reports at the individual physician and specialty level. They will also generate reports on how often you choose to take the INSPIRE prompt recommendation in low risk (<10%) patients, how often you override, and what your reasons for override were. They will share these data with leadership and with you to address any questions or concerns you may have. Below are examples of feedback reports that may be shared with you:

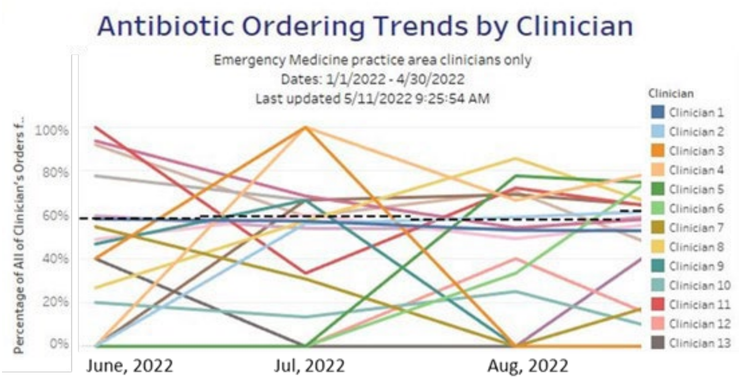

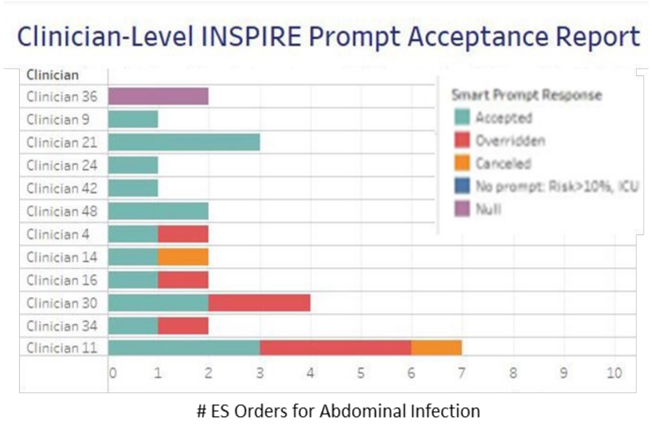

What is the goal of feedback and what response is expected of me?

The goal of feedback is to reduce ES antibiotic use at your facility and help target antibiotic stewardship education. You are fully expected to select antibiotics according to your best clinical judgment at all times. Feedback on antibiotic use is expected to facilitate meaningful and collegial conversations between you and your antibiotic stewardship team.

Funded by National Institute of Allergy and Infectious Diseases

INSPIRE Abdominal and Skin & Soft Tissue Infection Trials

Hot Topic: Safety of Choosing Standard-Spectrum Empirically

MDRO rates are rising, doesn't that mean my patient's risk for MDRO is high?

Multidrug-resistant organism (MDRO) rates are rising due to overuse of extended-spectrum (ES) antibiotics. While MDRO rates may rise at the population level, this does not directly translate to an increased risk of MDRO infection at the individual level. In fact, the **vast majority of patients do not have infection from an MDRO**.

We assessed over 422,000 HCA Healthcare patients over 3 years and found that <1% of patients with abdominal (ABD) infection grew MDROs. Similarly, <4% of patients with skin/soft tissue (SST) infection were infected with *Pseudomonas* and 12% grew MRSA. Yet, ES antibiotic use was overwhelmingly higher than needed. The vast majority could have been safely treated with standard-spectrum.

Antibiotic Use & MDRO Prevalence – All HCA Facilities

ABD Infection Antibiotic Use	
Extended-Spectrum	62.6%
Vancomycin	24.4%
Antipseudomonal	53.3%
**SST Infection Antibiotic Use**	
Extended-Spectrum	89.4%
Vancomycin	80.5%
Antipseudomonal	60.8%

HCA baseline data 2017-2019

→

MDRO ABD Infection	
MRSA	0.9%
Pseudomonas	0.8%

MDRO SST Infection	
MRSA	12.1%
Pseudomonas	3.7%

Is it safe to choose empiric standard-spectrum antibiotics?

Yes. Studies have shown that non-critically ill patients who are started on ES antibiotics have similar length of stay, mortality, and clinical cure rates when compared with those started on standard-spectrum antibiotics. The INSPIRE Pneumonia and UTI Trials successfully reduced empiric ES use by up to 30% without impact on length-of-stay, ICU transfers, or need for antibiotic escalation.

Why not start with ES and de-escalate after cultures are back?

Every dose and day of ES antibiotics can put your patient at higher risk for renal insufficiency, *C. difficile* colitis, future development of MDRO, adverse drug effects and healthcare costs. Emerging data show that as little as a few doses of antibiotics can alter a patient's gut flora, which has been linked to increased risk of numerous chronic, metabolic, oncologic, and inflammatory diseases.

Data indicate that 80% of antibiotics originally started in the emergency department are continued throughout the admission. So, starting with standard-spectrum antibiotics up front can significantly reduce your patient's risk of negative effects of ES antibiotics.

Funded by National Institute of Allergy and Infectious Diseases

INSPIRE Abdominal & Skin/Soft Tissue Infection Trials

INtelligent Stewardship Prompts to Improve Real-time Empiric Antibiotic Selection for Patients

Clinician Education

INSPIRE

BETTER SAFER ANTIBIOTIC USE

Context & Rationale

Overuse of Antibiotics: A Major National Priority

- Extended-spectrum (ES) antibiotics increase resistance[1]
- 40% inpatients receive inappropriate antibiotics[2]
- Adversely affects patient safety metrics – *C. difficile*, MRSA bacteremia
- National and state mandates for stewardship programs
- Gaps in empiric stewardship
  - National efforts focus on de-escalation not empiric choice
  - Dedicated strategies needed for empiric selection

[1]Paterson, D et al. Clin Infect Dis 2014;33:126-128

[2]Fridkin, S et al. MMWR 2014;63(9):194-200

Selecting Empiric Antibiotics: There is Time to Broaden

- Evidence in non-ICUs - no harm in waiting for cultures before ES antibiotics used
- Similar outcomes with empiric standard vs ES antibiotics[1]
- Waiting for culture results to start ES antibiotics in those who eventually grow an MDRO does not adversely impact mortality[1,2]

[1]Rhedin, S., et al. Clinical Microbiology and Infection, 23.4 (2017): 247-252.

[2]Lin, Michael Y., Antimicrobial agents and chemotherapy 52.9 (2008): 3188-3194.

INSPIRE
BETTER SAFER ANTIBIOTIC USE

Rationale for INSPIRE-ASP Trial

- Uncertainty about possible MDRO drives overuse of ES antibiotics
- Efforts needed to improve empiric prescribing
- Prescribers can choose wisely if
  - Useful, actionable information provided at time of order entry
  - Information individualized to their patient
  - Information documented to support their prescribing choice
  - Feedback on compliance routinely given

INSPIRE Pneumonia & UTI Trials
Success Reducing Empiric ES Antibiotics

INSPIRE: Pneumonia Trial Primary Outcome

- **28% absolute reduction** in ES-Days of Therapy in Arm 2
- Benefit persisted despite distractions of COVID-19
- **No increase in ICU transfer, LOS, antibiotic escalations**

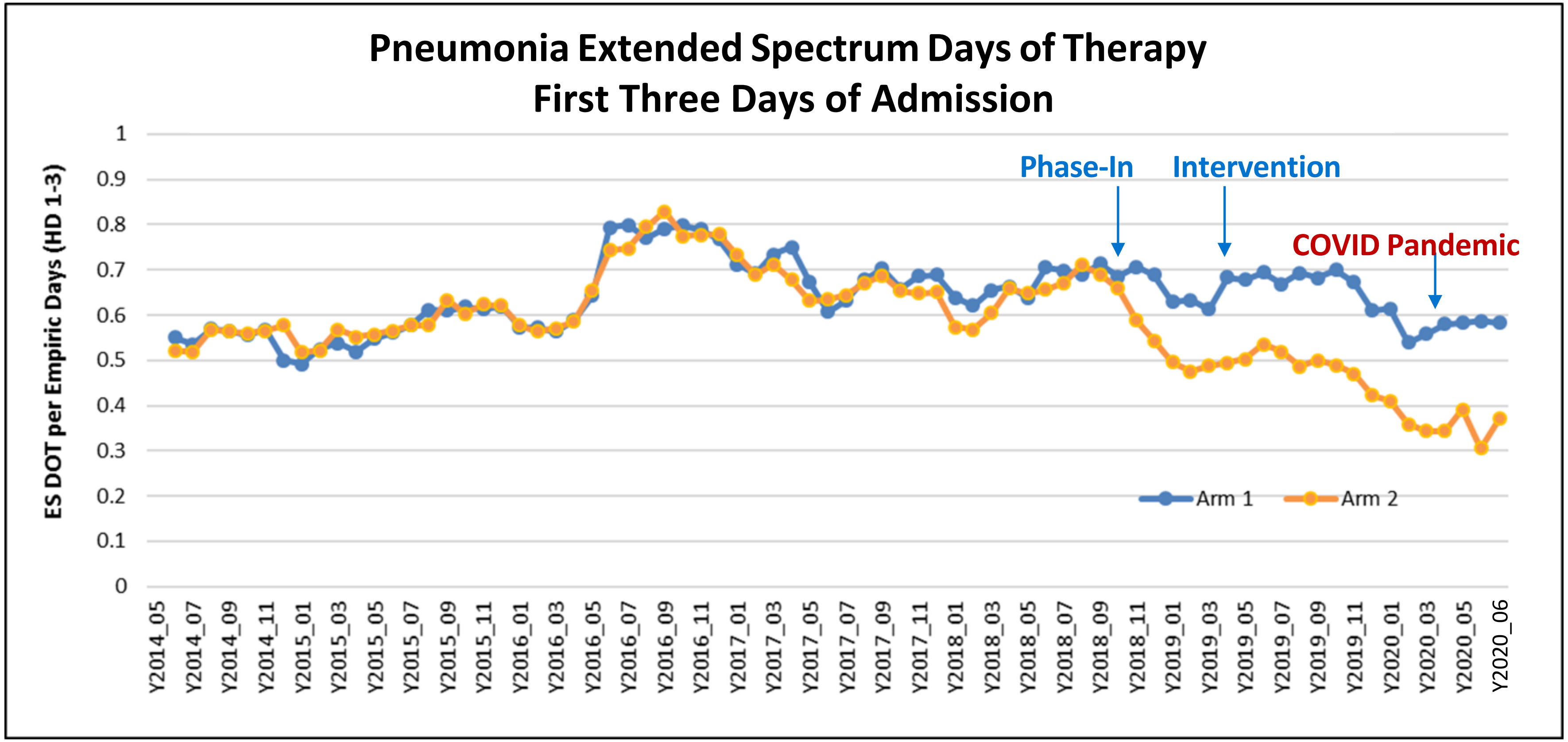

CONFIDENTIAL* - DO NOT DISTRIBUTE

INSPIRE: UTI Trial Primary Outcome

- **17% absolute reduction** in ES-Days of Therapy in Arm 2
- Benefit persisted despite distractions of COVID-19
- **No increased ICU transfer, LOS, antibiotic escalations**

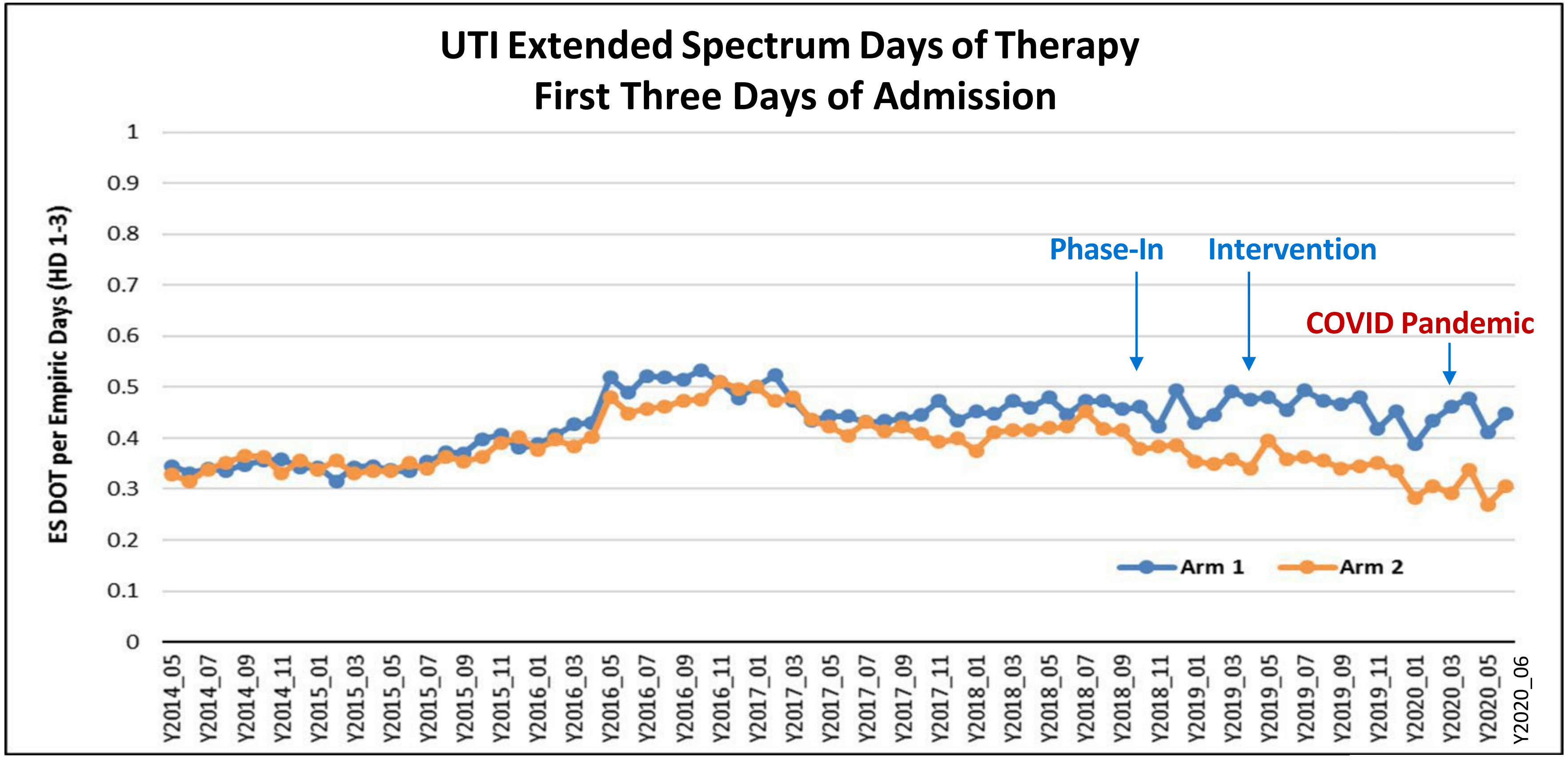

CONFIDENTIAL - DO NOT DISTRIBUTE

INSPIRE Pneumonia and UTI Trials

Major Achievements

- INSPIRE Prompt yielded rapid durable response when coupled with provider acceptance and feedback
- Benefit persisted during distractions of COVID
- Net $1.7 million saved in 15 months across 29 hospitals
- INSPIRE Pneumonia & UTI smart prompts rolled out HCA-wide Summer 2021

Suggests value in extension to other diseases

INSPIRE Abdominal (ABD) & Skin/Soft Tissue (SST) Infection Trials

Welcome to Arm 2

Intervention

Precision Medicine Smart Prompts for Patient-specific
Empiric Antibiotic Selection for Abdominal and Skin/Soft Tissue Infections

INSPIRE
BETTER SAFER ANTIBIOTIC USE

What Antibiotics Are Considered "Extended-Spectrum"?

- INSPIRE smart prompt will not trigger for restricted antibiotics
  - E.g., requiring prior approval or non-formulary

Category	ES Antibiotics Included in CPOE Alerts
**Anti-MRSA**	Ceftaroline, Daptomycin, Linezolid[1], Vancomycin[2]
**Anti-VRE**	Daptomycin, Linezolid[1]
**Anti-Pseudomonas**	Aztreonam, Cefepime, Ceftazidime, Piperacillin/Tazobactam
**Anti-ESBL**	Ertapenem, Imipenem, Meropenem, Ceftolozane/Tazobactam

[1]Both oral and intravenous (IV) formulations

[2]IV formulation only

The Gap Between MDRO Prevalence & ES Antibiotic Use

- ES antibiotic use far exceeds MDRO prevalence!
- Among >422,000 HCA Healthcare patients, 2017-2019
  - **<2% of ABD and <4% of SST patients grew MDROs**
  - MRSA SST prevalence higher but ES prescribing 8-fold higher than needed
- Vast majority could have received standard-spectrum

Antibiotic Use & MDRO Prevalence –All HCA Facilities

ABD Infection Antibiotic Use	
Extended-Spectrum	62.6%
Vancomycin	24.4%
Antipseudomonal	53.3%
**SST Infection Antibiotic Use**	
Extended-Spectrum	89.4%
Vancomycin	80.5%
Antipseudomonal	60.8%

→

MDRO ABD Infection	
MRSA	0.9%
Pseudomonas	0.8%

MDRO SST Infection	
MRSA	12.1%
Pseudomonas	3.7%

HCA baseline data 2017-2019

INSPIRE — BETTER SAFER ANTIBIOTIC USE

INSPIRE ABD and SST Trials - Design

Set of two 92-hospital, cluster-randomized trials

Arm 1: Routine Care – 46 Hospitals

- Standard-of-care antibiotic stewardship

Arm 2: INSPIRE Smart Prompt Intervention – 46 Hospitals

- Physician "smart prompts" for patient-specific MDRO risk
- Recommends appropriate antibiotic choice
- Provider encouragement and feedback reports

INSPIRE Study Population and Timeline

Includes

- Adults admitted for **Abdominal or Skin/Soft Tissue Infections**
- Excludes patients admitted to ICUs
- Focuses on first 3 days of admission

Trial Timeline

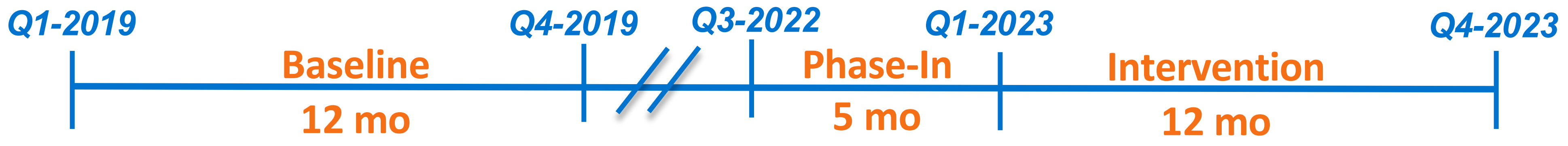

INSPIRE Abdominal & Skin/Soft Tissue Trial Outcomes

Primary Outcome

- ES antibiotic days of therapy (ES-DOT) first 3 days of admission

Secondary Outcomes

- Vancomycin DOT first 3 days of admission **(ABD only)**
- Antipseudomonal DOT first 3 days of admission

Safety Outcomes

- ICU transfers
- Length-of-stay

Other Outcomes (Secondary Manuscripts)

- Incidence of *C. difficile,* MDROs, and other adverse effects
- Cost analysis

INSPIRE MDRO Risk Estimates

How Are They Calculated?

The Problem with Relative Risk and Odds Ratios (OR)

- Clinicians are taught about relative risk and ORs
- OR for MDRO infection often 2 or 3 for nursing home residence or diabetes
- However, this tells us nothing about the absolute risk
- Example
  - Let's say females have a risk of 1 in 1,000 for ESBL abdominal infection
  - If males have a risk of 3 in 1,000, that would be OR = 3 for an absolute risk of 0.3%
  - Better to know absolute risk
  - **The INSPIRE trials use absolute risk**

Models Assessing Absolute Risk

- **We assume ES antibiotics are prescribed for the MDROs they treat**
  - ✓ Models exist for every ES antibiotic–MDRO pair, for example
    - ➢ Vancomycin -- MRSA
    - ➢ Linezolid ordered -- MRSA or VRE
    - ➢ Cefepime -- *Pseudomonas*
    - ➢ Piperacillin/Tazobactam -- *Pseudomonas*
    - ➢ Meropenem -- ESBL or *Pseudomonas*
  - ✓ Goals: find best set of factors that predict risk ≥10% → allow ES antibiotic
  - ✓ Patients with risk <10% → standard spectrum antibiotic can be recommended

Which Variables Assessed for Every ES Antibiotic-MDRO Model?

- 195,000 SST and 227,000 ABD patients admitted to HCA hospitals, 2017-2019
- We evaluated:

  (1) Hospital antibiogram for ABD/SST infection patients

  (2) Risk factors of MDRO infection
  - Demographics
  - Comorbidities
  - MDRO or *Pseudomonas* history
  - Recent admission, ED visit, nursing home care
  - Abdominal surgery within 1 year
  - Recent antibiotic use
  - Labs (e.g., BUN, creatinine)

Risk Model – *Pseudomonas* ABD Infection

- If absolute risk for *Pseudomonas* infection ≥10%, ES order proceeds w/o prompts
- Among risk factors, only history of *Pseudomonas* and abdominal surgery mattered

Rules	N	Proportion Predicted with *Pseudomonas* ABD Infection
Pseudomonas Hx<br>History of abdominal surgery within 1 year	574	10%
Pseudomonas Hx<br>No abdominal surgery within 1 year	546	6%
All others not meeting above	180977	<=5%

HCA baseline data 2017-2019

INSPIRE Smart Prompt

Risk Model - Pseudomonas SST Infection

- Only predictive risk factor for SST infection: history of prior *Pseudomonas*
- No other traditional risk factor e.g., diabetes, nursing home residence, labs mattered

Rules	N	Proportion Predicted with *Pseudomonas* SST Infection
Pseudomonas Hx	6776	15%
All others not meeting above	141953	4%

HCA baseline data 2017-2019

→ ***INSPIRE Smart Prompt***

INSPIRE CPOE Smart Prompt Intervention

CPOE Smart Prompt Activated Via Indication Screens

- INSPIRE prompts triggered by indications for ABD or SST
- If sepsis or empiric selected, a secondary screen is triggered for infection source

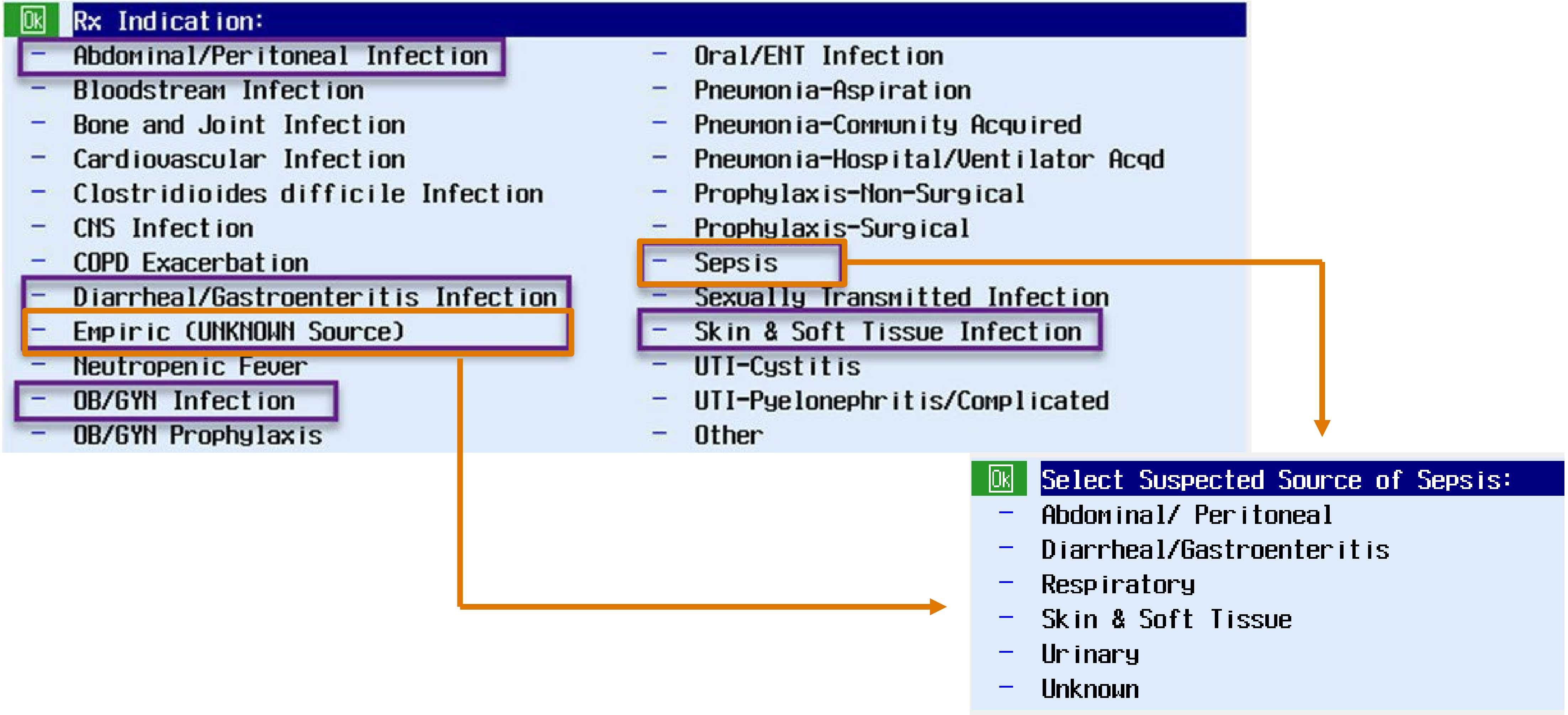

CPOE Smart Prompt Intervention

- **How will it be "smart"?**
  - ✓ Uses ***patient-specific*** EHR data to calculate MDRO risk based on models from 422,000+ HCA patients with ABD or SST
  - ✓ Risk estimate tailored to antibiotic selected. For example:
    - ➢ If ceftriaxone ordered → no prompt (standard-spectrum antibiotic)
    - ➢ If vancomycin ordered → returns MRSA risk
    - ➢ If cefepime ordered → returns *Pseudomonas* risk
    - ➢ If linezolid ordered → returns MRSA and VRE risk
    - ➢ If meropenem ordered → returns ESBL + *Pseudomonas*
  - ✓ If risk $\geq$10%, ES order proceeds without prompts

Excluding ED Patients Going to ICU

- Smart prompt **<u>will not</u>** fire if patient located in ICU
- **For ED patients**, asks if patient is likely to go to the ICU
  - If YES → no further screens
  - If NO → INSPIRE CPOE screens continue

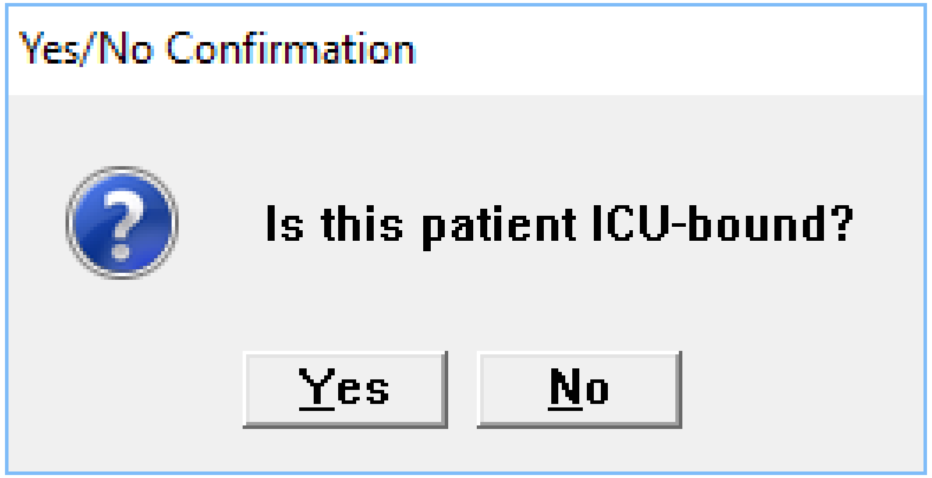

Abdominal Surgery History for Select ES Antibiotics

- INSPIRE risk models – abdominal surgery within past year significantly impacts risk of MRSA and *Pseudomonas* abdominal infection
- **If physician orders anti-MRSA or anti-Pseudomonal for abdominal infection:**

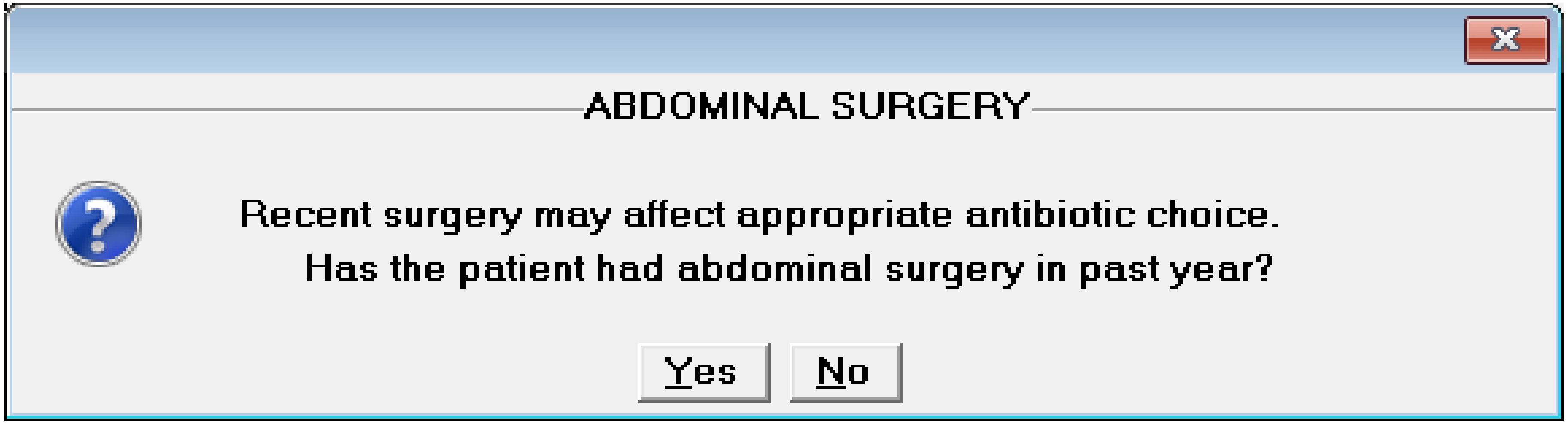

INSPIRE CPOE Smart Prompt Screen - Example

- Physician orders cefepime for SST infection, and
- Risk of *Pseudomonas* SST infection is low (<10%)

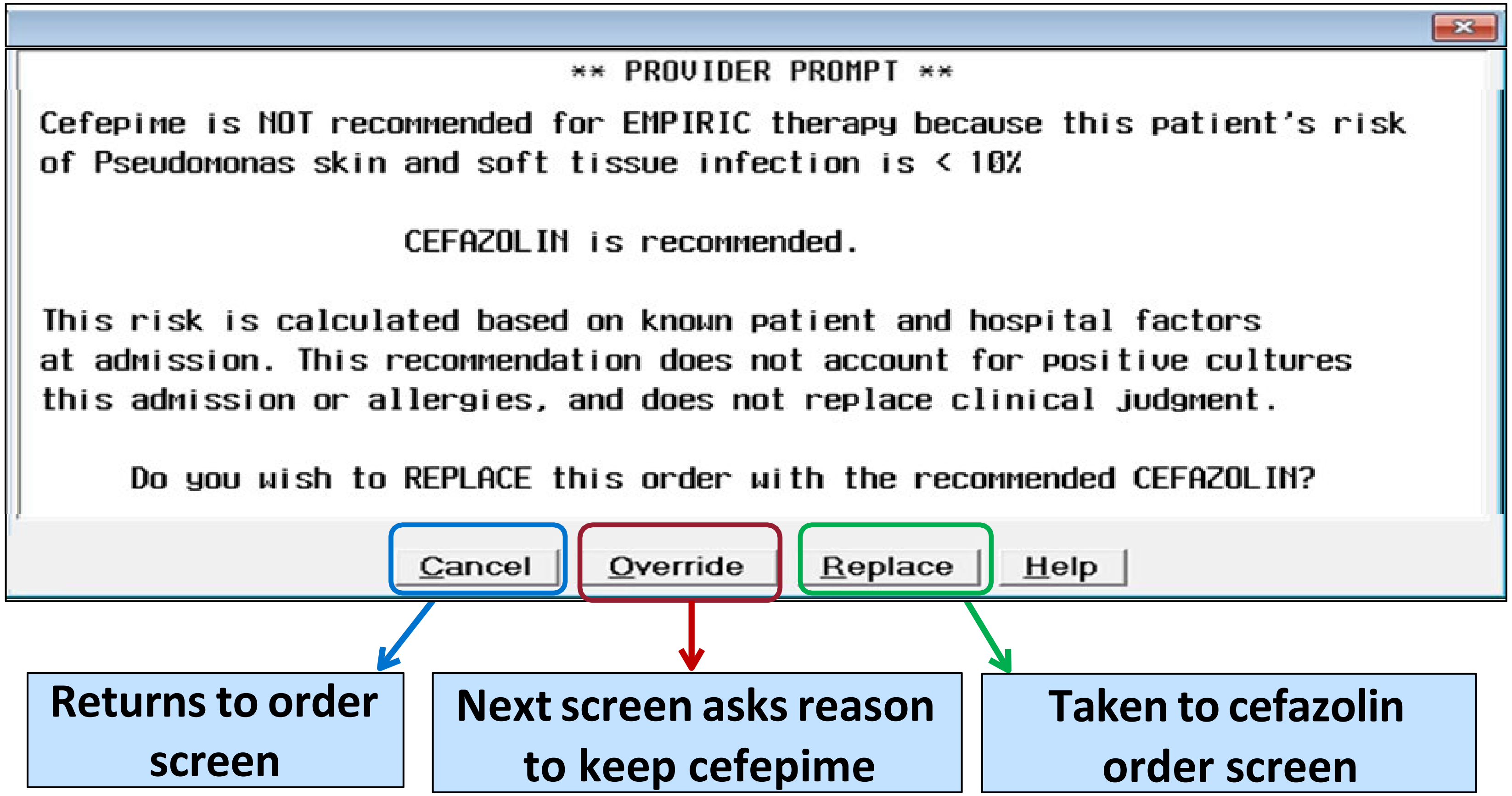

INSPIRE CPOE Smart Prompt Screen - Example

- Physician orders vancomycin for abdominal infection and
- Risk of *MRSA* abdominal infection calculated to be low (<10%)

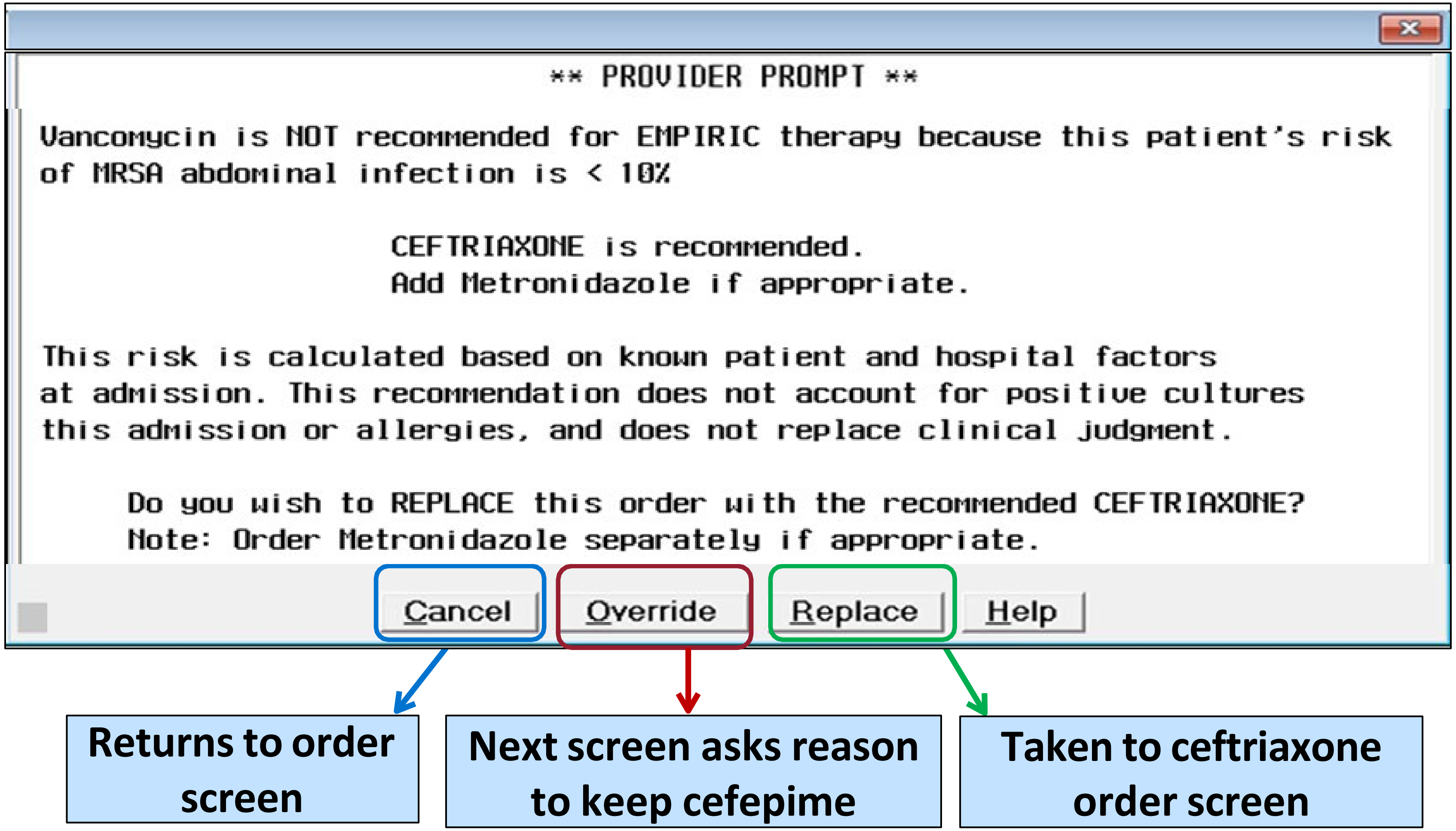

Override Screen and Options

- Clinicians choosing to override will be asked to indicate reason:

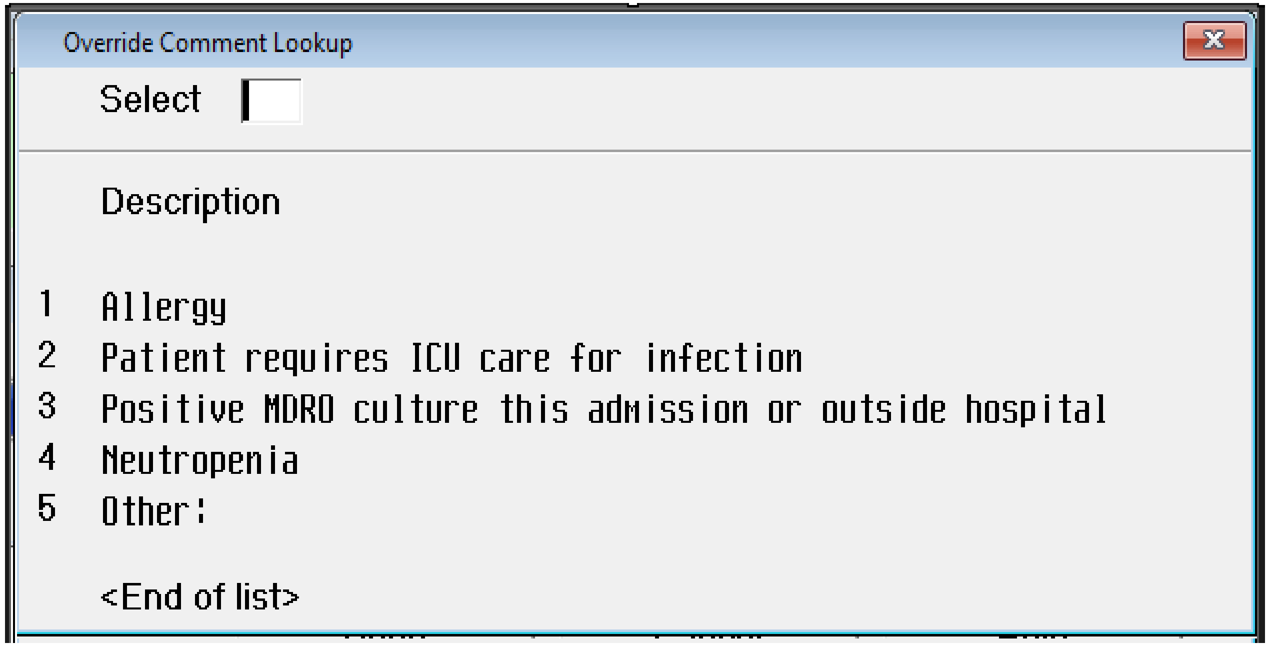

*****Allergy **not** auto-programmed:* *some allergies missing in EHR, and many in EHR are not true allergies – needs clinical judgment*

*****Usual facility allergy prompts continue to appear*

Clinical Judgment Matters

- INSPIRE prompt provides real-time, patient-specific risk estimates to help you choose the best initial antibiotic
- Alert supports, but does not replace clinical judgment
- You may have information not in Meditech that you should consider:
  - Any MDRO cultures this admission or at outside hospital
  - Neutropenia
  - Allergy to recommended antibiotic
  - Imminently headed to ICU
- Adjust antibiotics as needed based on culture results and response to treatment

Will Model Be Validated? What if Local Prevalence Changes?

- INSPIRE models are robust
- Based on 3 years of data across HCA Healthcare
  - Estimates for single facility unlikely to change significantly in 1 year
- Facility prevalence stable during entire baseline evaluation
- Risk estimates re-validated periodically to ensure accuracy

Will Patients Need to Give Informed Consent?

- Informed consent waived by Harvard IRB
- Rationale
  - Comparison of Quality Improvement strategies
  - Patient consent is not required for antibiotic stewardship initiatives
  - Minimal risk

Quarterly Clinician Progress Reports

Principles of Clinical Decision Support

WHO Principles of Clinical Decision Support:

- Speed
- Simplicity
- Usability
- Integration into workflow
- Actionable and provides alternatives
- Evidence/justification provided
- **Impact should be monitored**

Smart prompt provides (Speed through Evidence/justification provided)

CDC: Core Elements of Antibiotic Stewardship

- Prescriber feedback - improves use up to 40%

INSPIRE — BETTER SAFER ANTIBIOTIC USE

Your Antibiotic Stewardship Team Will Provide Active Feedback

Antibiotic use reports will be reviewed and shared with you and hospital leaders

- Clinician-specific
- Department-specific
- Infection-specific

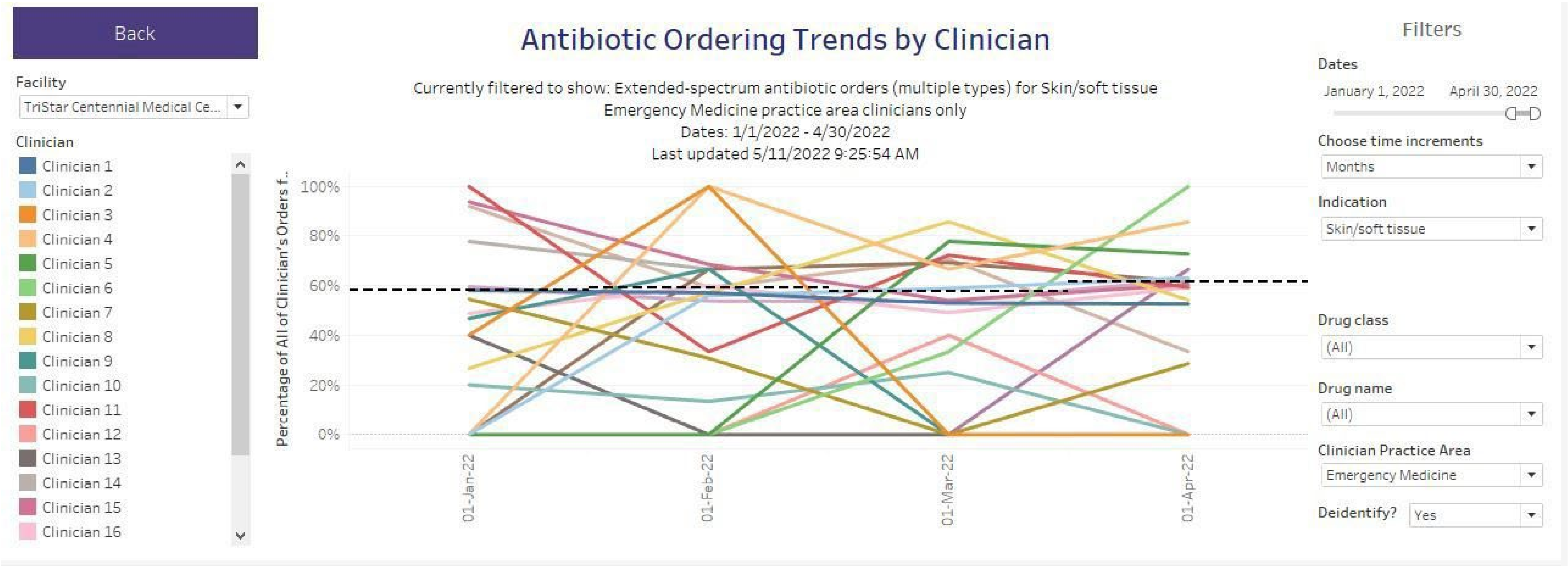

Active Feedback Will Include Reviewing Prompt Response

Response to INSPIRE prompt will be monitored, including

- Prompt acceptance
- Override reasons

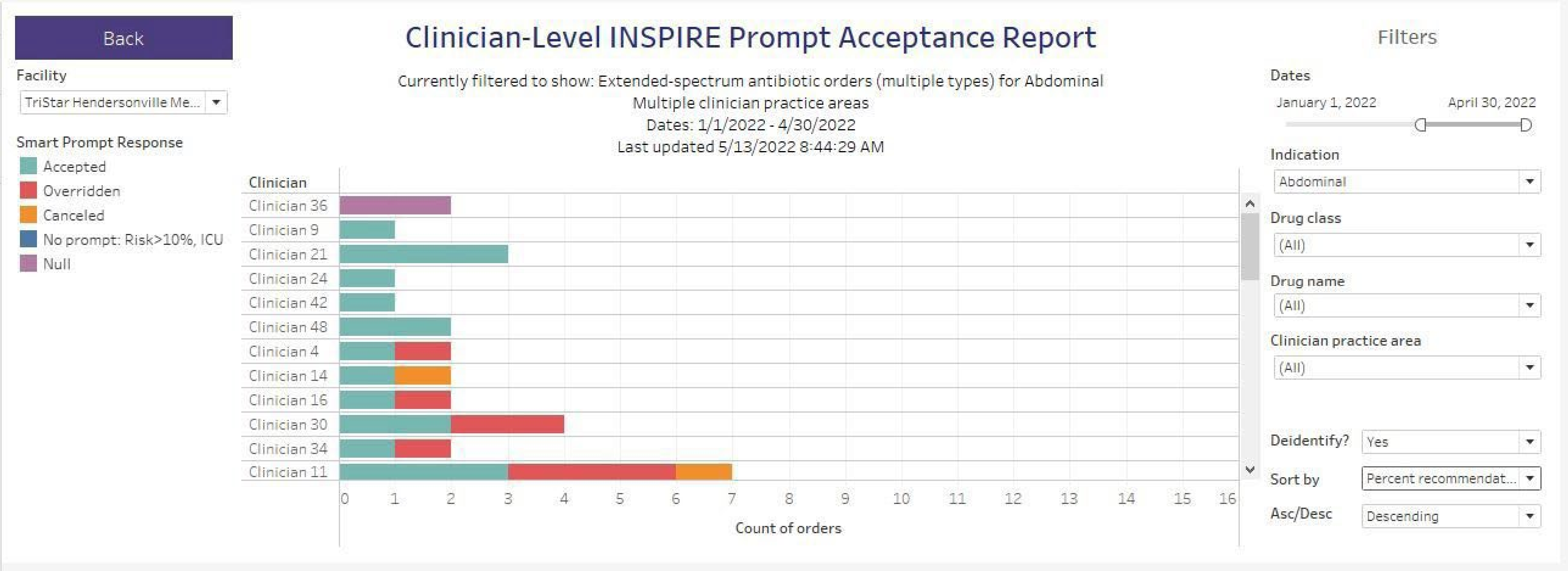

Landmark Trials

- Precision-medicine trials for antibiotic stewardship
- Real-time, patient-specific smart prompts for ABD and SST infections
- Based upon absolute risk, not relative risk
- If successful → best practice for the U.S.

HCAINSPIRE@gmail.com

INSPIRE Contacts

- **Shruti Gohil, MD, MPH**
  Lead Investigator, HCA INSPIRE Trials
  Phone: (844) 522-4677
  HCAINSPIRE@gmail.com

- **Kaleb Roemer, PharmD**
  Clinical Pharmacy Services Manager, HCA
  Phone: (615) 344-5055
  Email: Kaleb.Roemer@hcahealthcare.com

- **Edward Septimus, MD**
  Harvard-HCA Liaison
  Phone: (281) 714-5689
  Email: Edward.Septimus@hcahealthcare.com

- **Kenneth Sands, MD**
  Chief Epidemiologist, HCA
  Phone: (615) 344-2791
  Email: Kenneth.Sands@hcahealthcare.com

NOTE: Emails and phone numbers approved by HCA Security

Do NOT send PHI to these accounts

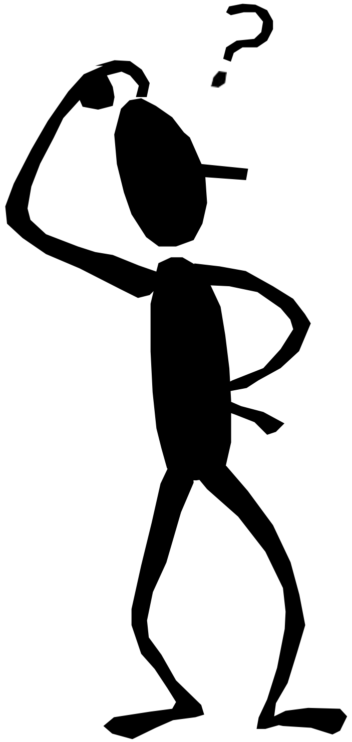

Open Floor Q&A

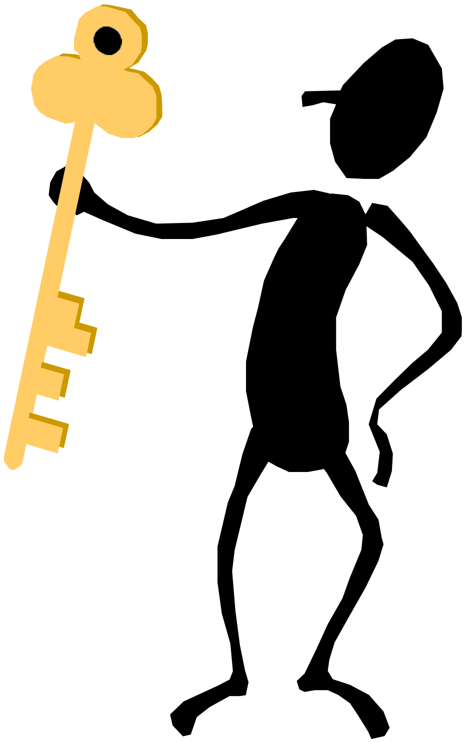

INSPIRE Computerized Physician Order Entry (CPOE) Prompt
Skin & Soft Tissue Infection Workflow

Beginning **August 2, 2022,** our hospital will launch an antibiotic stewardship intervention to **encourage compliance with national guidance for standard-spectrum antibiotic use for non-ICU abdominal and skin/soft tissue (SST) infections.**

Ordering extended-spectrum (ES) antibiotics (e.g., vancomycin, cefepime) for SST infection in the emergency department (ED) or non-ICUs within 3 days of admit will activate a computerized physician order entry (CPOE) smart prompt that will:

- Calculate your patient's risk of infection due to antibiotic resistant bacteria, based upon:
    - Your patient's risk factors available in your hospital's electronic medical record
    - Your hospital's antibiogram
- Recommend standard-spectrum antibiotics if risk is estimated to be low (<10%)

INSPIRE CPOE Workflow: Skin & Soft Tissue Infection Workflow

1. Indications are required for all antibiotics per HCA Healthcare protocol
2. If **an ES antibiotic is ordered for an SST infection indication** (outlined in red) **in a non-ICU patient during the first 3 days of admission, the INSPIRE CPOE prompts will be triggered.**
    a. Note: INSPIRE prompts for pneumonia and urinary tract infection have been active at all HCA facilities since 2021.

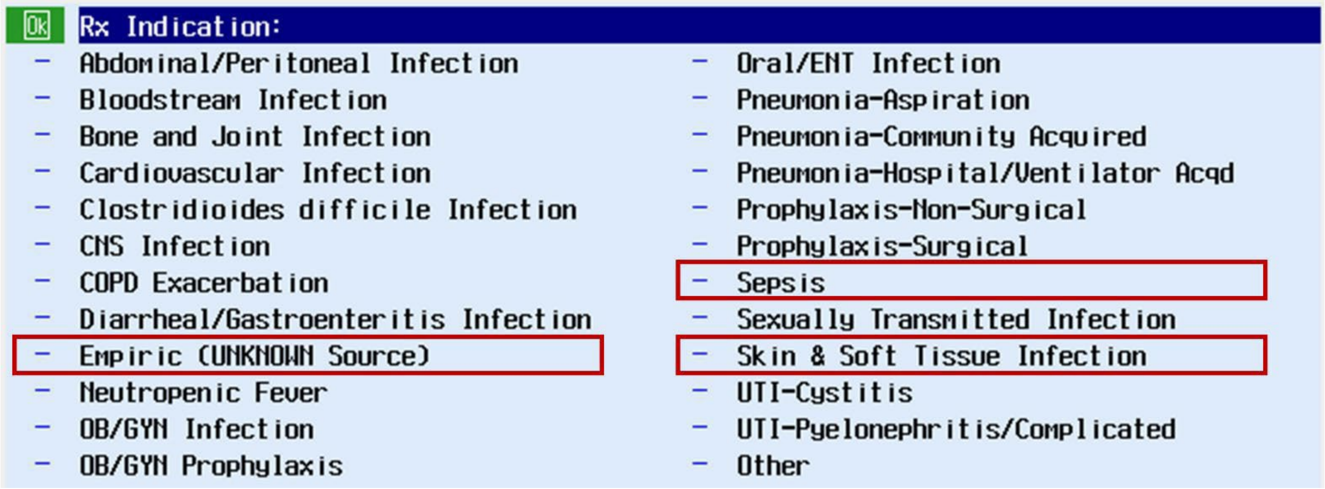

3. INSPIRE CPOE prompts are programmed to **fire for patients in the ED and non-ICUs only** and will **NOT** fire if a patient is in an ICU location:

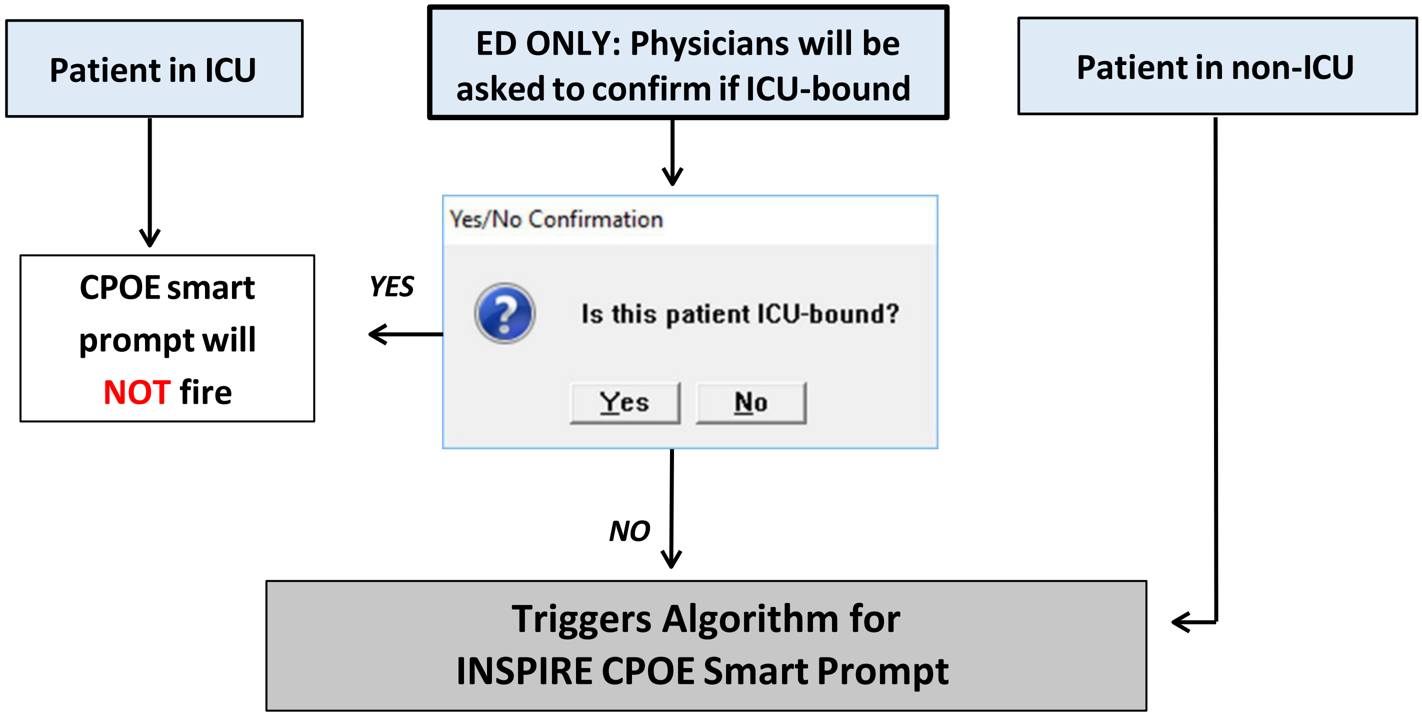

4. INSPIRE CPOE prompts will automatically assess your **patient's individual risk for antibiotic resistant skin & soft tissue infection using data available in your hospital's electronic medical record**. This risk estimate is tailored to the antibiotic ordered.
    a. For example:
        i. If vancomycin ordered → returns MRSA risk
        ii. If cefepime ordered → returns *Pseudomonas* risk
        iii. If linezolid ordered → returns MRSA and VRE risk
        iv. If meropenem ordered → returns ESBL + *Pseudomonas* risk

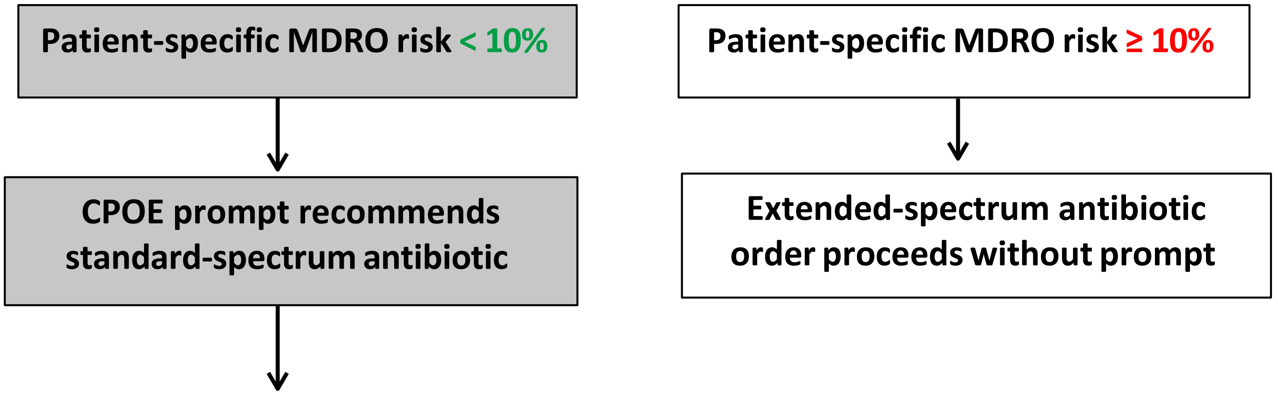

(See following page for mock INSPIRE CPOE smart prompt)

Mock CPOE Smart Prompt Recommendation Screen

<u>Example INSPIRE Skin & Soft Tissue Infection Smart Prompt</u>:

Scenario: Physician orders cefepime for skin & soft tissue infection.
Patient risk for *Pseudomonal* skin & soft tissue infection is calculated to be <10%.

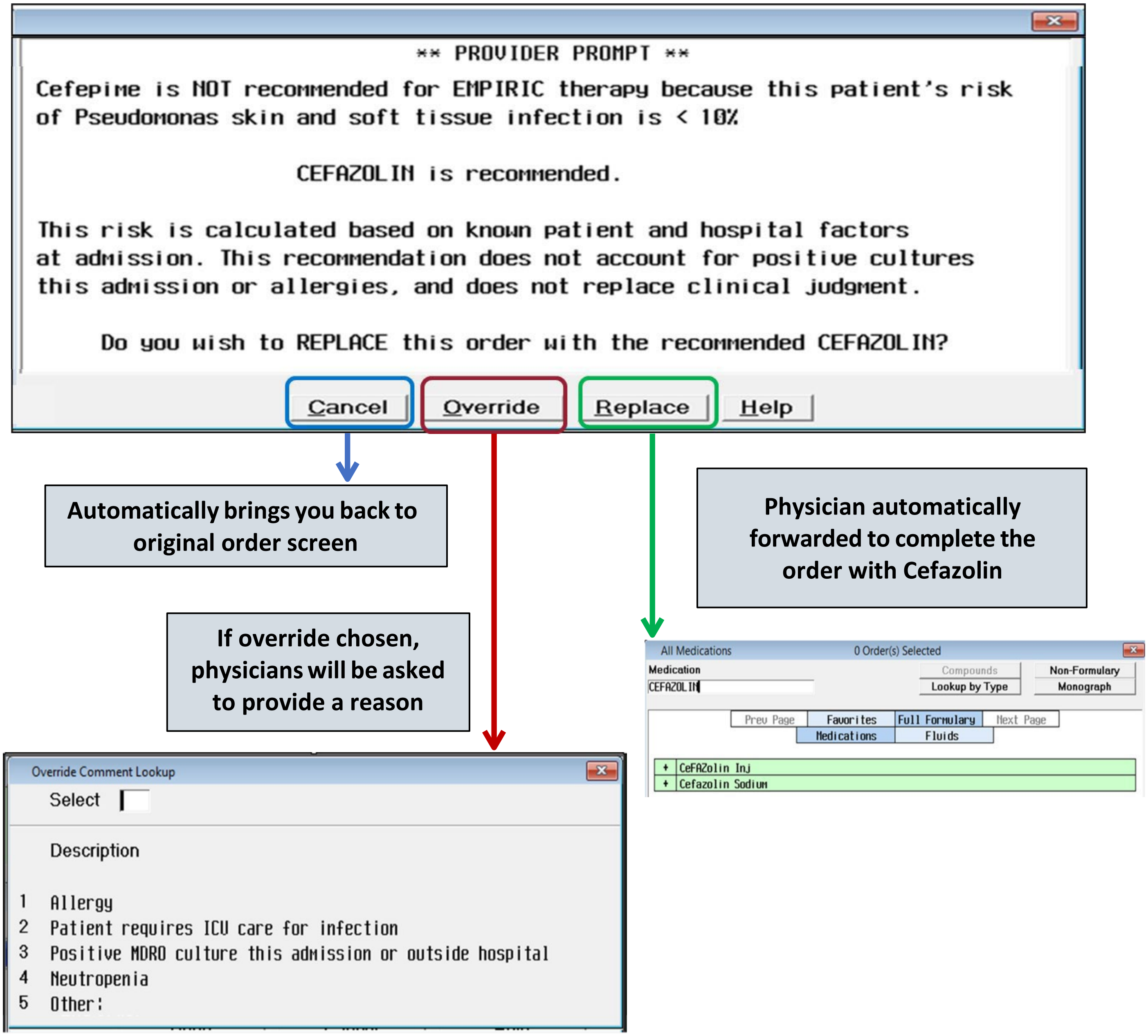

INSPIRE Computerized Physician Order Entry (CPOE) Prompt
Abdominal Infection Workflow

Beginning **August 2, 2022,** our hospital will launch an antibiotic stewardship intervention to **encourage compliance with national guidance for standard-spectrum antibiotic use for non-ICU abdominal and skin/soft tissue (SST) infections.**

Ordering extended-spectrum (ES) antibiotics (e.g., vancomycin, cefepime) for abdominal infections in the emergency department (ED) or non-ICUs within 3 days of admit will activate a computerized physician order entry (CPOE) smart prompt that will:

- Calculate your patient's risk of infection due to antibiotic resistant bacteria, based upon:
    - Your patient's risk factors available in your hospital's electronic medical record
    - Your hospital's antibiogram
- Recommend standard-spectrum antibiotics if risk is estimated to be low (<10%)

INSPIRE CPOE Workflow: Abdominal Infection Workflow

1. Indications are required for all antibiotics per HCA Healthcare protocol
2. If **an ES antibiotic is ordered for an SST infection indication** (outlined in red) **in a non-ICU patient during the first 3 days of admission, the INSPIRE CPOE prompts will be triggered.**
    a. Note: INSPIRE prompts for pneumonia and urinary tract infection have been active at all HCA facilities since 2021.

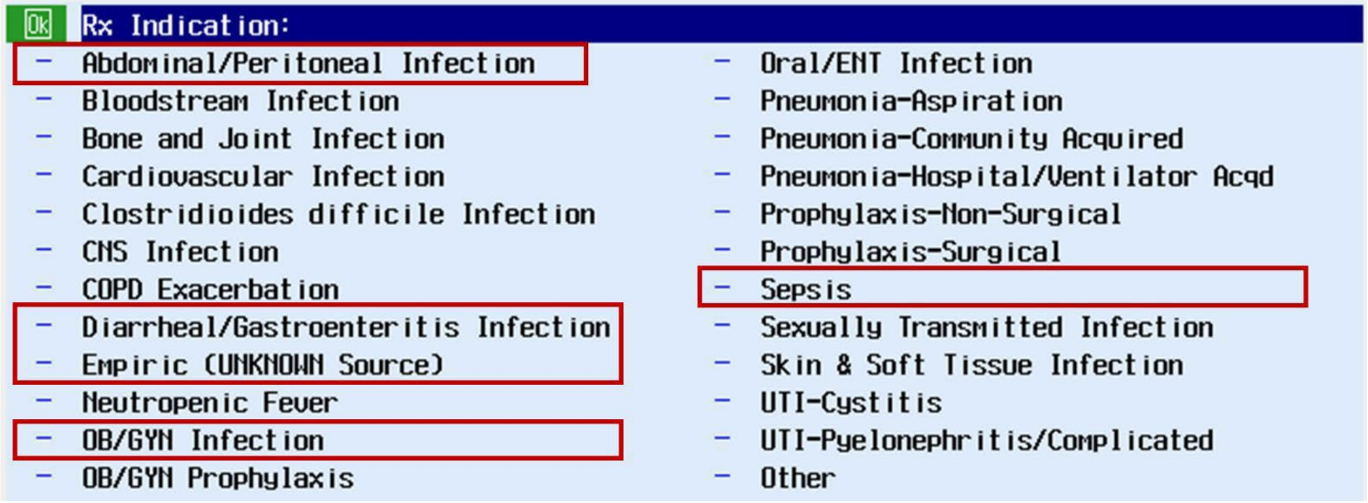

↓

3. INSPIRE CPOE prompts are programmed to **fire for patients in the ED and non-ICU units only** and will **NOT** fire if a patient is in an ICU location:

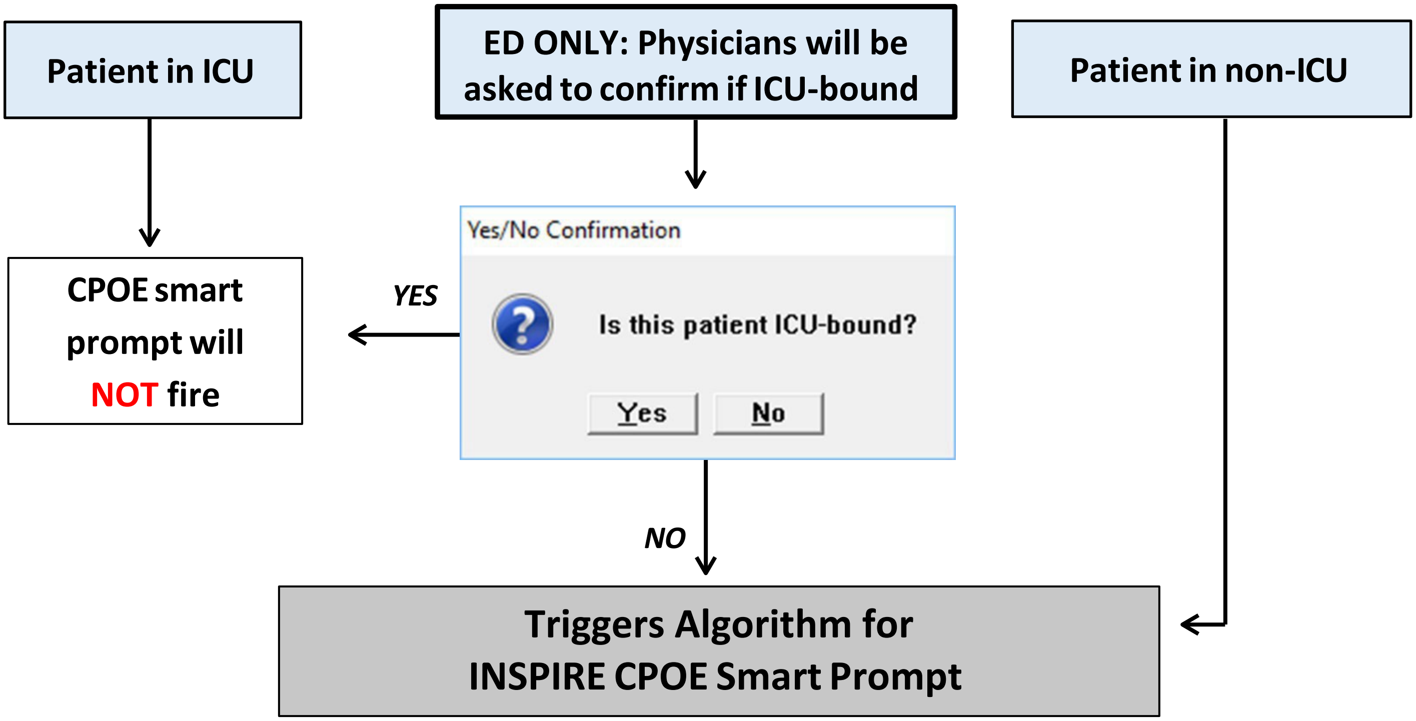

4. INSPIRE CPOE prompts will automatically assess your **patient's individual risk for antibiotic resistant abdominal infection using data available in your hospital's electronic medical record**. This risk estimate is tailored to the antibiotic ordered.
    a. For example:
        i. If vancomycin ordered → returns MRSA risk
        ii. If cefepime ordered → returns *Pseudomonas* risk
        iii. If linezolid ordered → returns MRSA and VRE risk
        iv. If meropenem ordered → returns ESBL + *Pseudomonas* risk

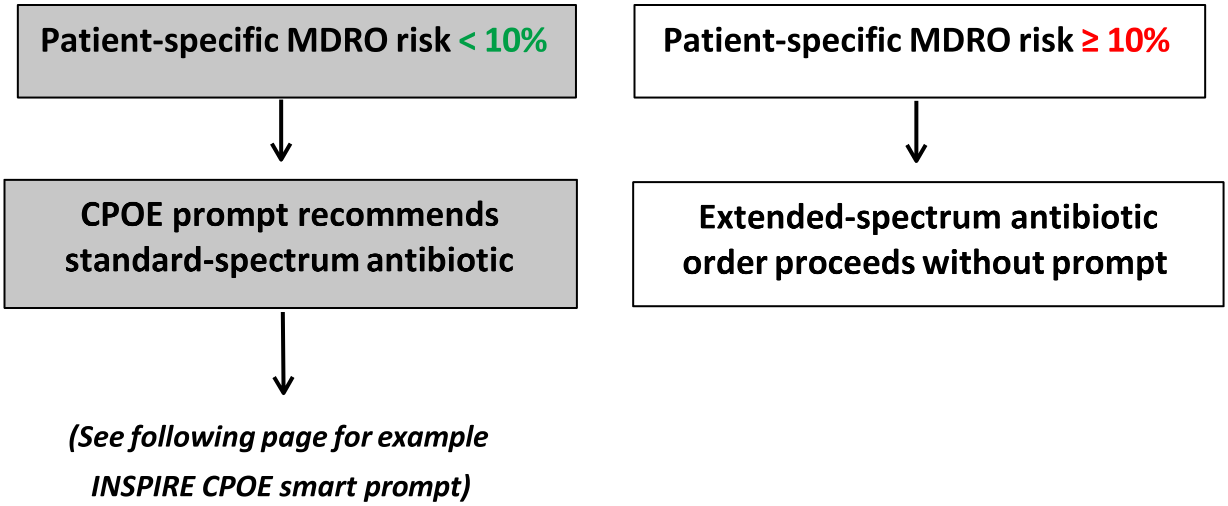

5. **For Anti-MRSA and antipseudomonal antibiotics only**:
    a. INSPIRE risk estimate models found that abdominal surgery within past year significantly impacts risk of MRSA and *Pseudomonas* abdominal infection.
    b. If the ordered extended-spectrum antibiotic targets these pathogens, then an additional screen will ask about surgical history before the final prompt recommendation screen:

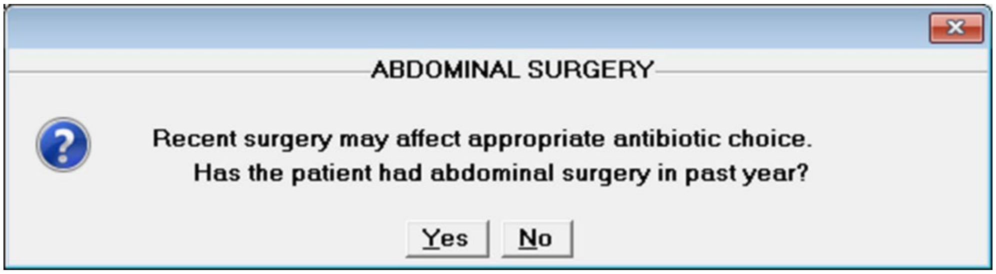

Mock CPOE Smart Prompt Recommendation Screen

<u>Example INSPIRE Abdominal Infection Smart Prompt</u>:
Scenario: Physician orders Vancomycin for abdominal infection.
Patient risk for *MRSA* abdominal infection is calculated to be <10%.

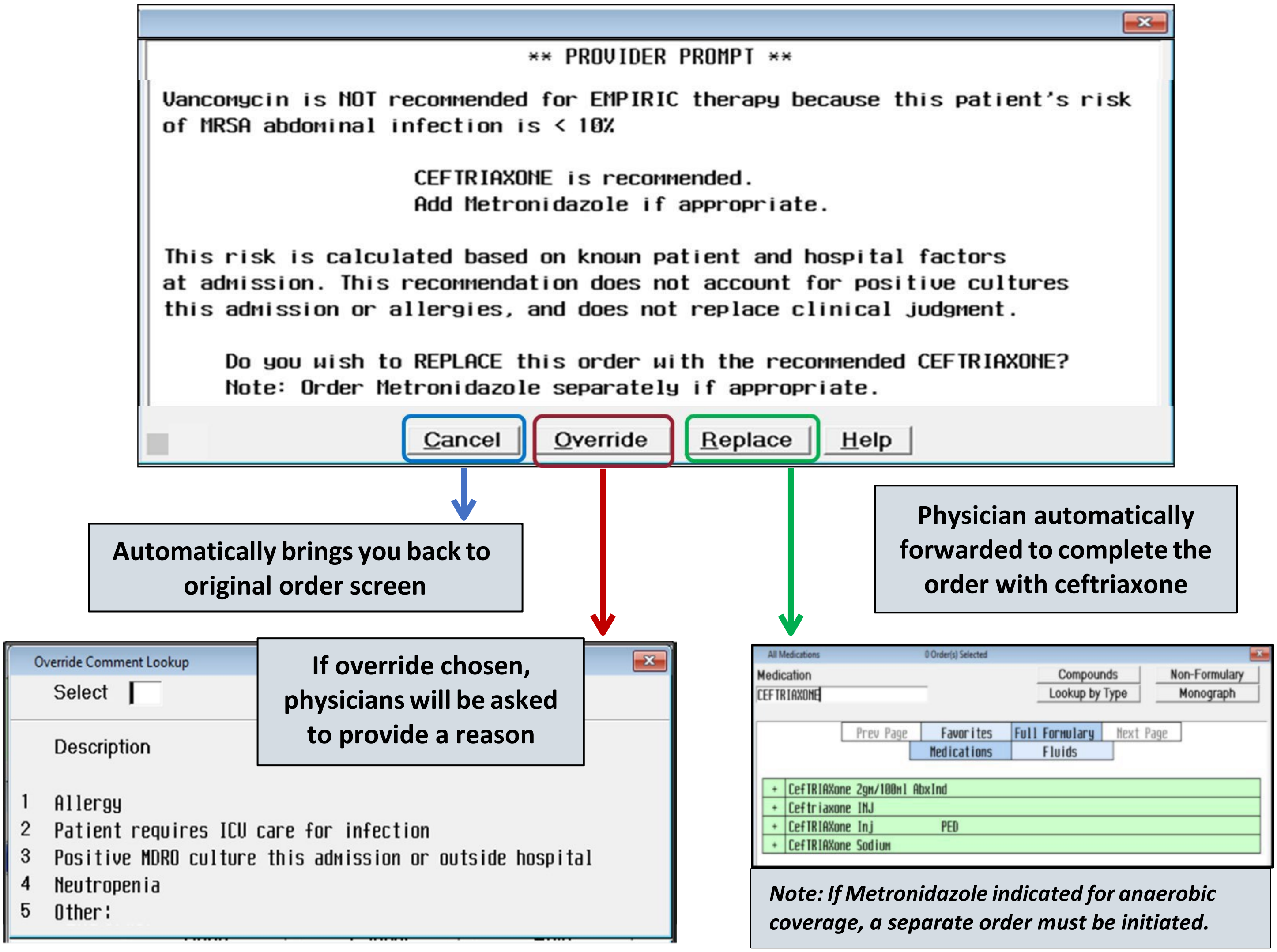

Promoting INSPIRE Trials at Your Facility

How can Study Champions increase clinician awareness of INSPIRE prompts?

It is important that clinicians understand that your hospital is a proud participant in the INSPIRE trials and that the INSPIRE smart prompts are intended to provide real-time, patient specific risk estimates to guide appropriate antibiotic ordering for abdominal and skin/soft tissue infections.

In addition to the “Clinician Education” materials in this Toolkit, we have provided the following items to help promote INSPIRE at your facility:

- **Poster Clings** – this self-adhesive poster is designed for immediate posting in clinicians work/lounge areas. These clings are compliant with regulatory standards; they do not require tape and will not leave sticky residue behind when removed. Study champions should hang these in communal areas frequented by clinicians, such as:
  - Clinician lounges
  - Workrooms – ED/hospitalist charting and/or rounding rooms
  - Operating room lounges
- **CPOE Workflow Laminates** – this series of key ring bound laminates is intended to be placed in centralized workstations where active ordering occurs.

You know your facility best. Study Champions should plan to adapt the materials we have provided and use them to best address your facility’s needs. Successful strategies used by facilities in the past include:

- Computer screensavers – announcing INSPIRE or judicious antibiotic use messaging
- QR-code based links to educational documents
- Poster boards
- Presentations
- Emails

Funded by National Institute of Allergy and Infectious Diseases

INSPIRE Abdominal Infection Trial
Trial Supplement 1

(Originally published in Gohil, et al. Improving Empiric Antibiotic Selection for Patients Hospitalized With Abdominal Infection: The INSPIRE 4 Cluster Randomized Clinical Trial. JAMA Surg. 2025;160(7):733–743. doi:10.1001/jamasurg.2025.1108)

Table of Contents

1. **Statistical Analysis Plan**
    a. Summary of changes
    b. Original Statistical Plan – 6/29/22
    c. Final Statistical Plan – 3/29/24

2. **Safety Outcome Supplemental Methods**
    a. Primary safety outcome measures
    b. Safety outcome sensitivity analyses

3. **INSPIRE Abdominal Infection Trial Protocol**
    a. Original Trial Protocol and Implementation Toolkit – Routine Stewardship Group
    b. Original Trial Protocol and Implementation Toolkit – CPOE Bundle Group
    c. Final Trial Protocol and Implementation Toolkit – N/A, same as original protocol
    d. Summary of changes – N/A, no changes

Supplement 1
INSPIRE AbdominalInfection Trial Trial
Protocol Amendments

Date	Summary of Trial Protocol Analytic Plan Amendments
**6/29/2022**	**Original**
**3/29/2024**	**Final**<br>Added language to clarify non-inferiority margins for safety outcomes. Removed power calculation language from the SAP. Minor wording changes for clarity.

(Originally published in Gohil, et al. Improving Empiric Antibiotic Selection for Patients Hospitalized With Abdominal Infection: The INSPIRE 4 Cluster Randomized Clinical Trial. JAMA Surg. 2025;160(7):733–743. doi:10.1001/jamasurg.2025.1108)

INSPIRE Abdominal Infections Trial
(INtelligent Stewardship Prompts to Improve Real-time Empiric Antibiotic Selection for Patients)

Statistical Analytic Plan

Date: 6.29.2022

Background

The **INSPIRE Abdominal Infections Trial** (INtelligent Stewardship Prompts to Improve Real-time Empiric Antibiotic Selection for Patients with Abdominal Infections) is a cluster-randomized trial of HCA Healthcare (HCA) affiliated hospitals to determine whether antibiotic prescribing practice for adult hospitalized patients admitted to non-critical care units with abdominal (ABD) infections can be affected by providing physicians with individualized risk estimates for whether a patient with an abdominal infection is likely to have an antibiotic-resistant infection. It compares routine care to the use of a real-time smart prompt for standard-spectrum antibiotics when a physician orders an extended-spectrum antibiotic (ES) for a patient whose risk of antibiotic resistant infection is low (<10%). The main goal is to see if there is a difference in the empiric antibiotic prescribing practices for ES. The term "antibiotics" refers to antibacterial antibiotics in this trial. We define antibiotics administered during the first three days of hospitalization as empiric treatment because infecting pathogens and their antimicrobial susceptibilities are typically not known during this period. We refer to these first three days as the empiric period.

Extended-spectrum antibiotics are defined in Table 1 below.

Table 1: Extended-Spectrum Antibiotics in the INSPIRE-ASP Abdominal Infections Trial

Category	Extended-Spectrum Antibiotics for INSPIRE CPOE Alerts
**Anti-MRSA**	Ceftaroline, Daptomycin, Linezolid[1], Vancomycin[2]
**Anti-VRE**	Daptomycin, Linezolid[1], Tigecycline
**Anti-Pseudomonas**	Aztreonam, Cefepime, Ceftazidime, Piperacillin/tazobactam
**Anti-ESBL**	Ertapenem, Imipenem, Meropenem, Ceftolozane/tazobactam
**Anti-CRE**	Ceftazidime/avibactam, Imipenem/relebactam, Meropenem/vaborbactam, Polymixin B, Colistin, Tigecycline

[1]Both oral and intravenous (IV) formulations
[2]IV formulation only

Note: Does not include aminoglycosides or fluoroquinolones.

Abbreviations: **MRSA -** Methicillin Resistant *Staphylococcus aureus*; **VRE –** Vancomycin Resistant *Enterococci*; **ESBL –** Extended Spectrum Beta Lactamase producer; CRE – Carbapenem Resistant Enterobacteriaceae

Participating hospitals are randomized to:

- **Arm 1 – Routine Care**
- **Arm 2 – Real-Time Risk Estimation Smart Prompt**

Precision medicine smart prompt using a computerized physician order entry (CPOE) alert that calculates a patient-specific risk estimate of antibiotic resistant infection and recommends standard-spectrum antibiotics in patients admitted for ABD infections who have low risk (<10%) for an antibiotic resistant infection.

Trial Outcomes

Table 2: Primary and Secondary INSPIRE-ASP Abdominal Infections Trial Outcomes

Outcome	Metric
**Primary Trial Outcome**	
Total Empiric Extended-Spectrum Antibiotic Days of Therapy (ES-DOT)	The number of different extended-spectrum antibiotics each empiric day, summed across the first 3 days of hospitalization.[1]
**Secondary Trial Outcomes**	
Vancomycin Days of Therapy per Empiric Day	The number of days Vancomycin was received on the first 3 days of hospitalization.[1]
Total Empiric Antipseudomonal Antibiotic Days of Therapy (ES-DOT)	The number of different antipseudomonal antibiotics each empiric day, summed across the first 3 days of hospitalization.[1,2]

[1]E.g., if a patient is admitted for 2 days, ES-DOT will be calculated across those 2 days; however if a patient is admitted for 4 days, only the first 3 days will be evaluated.

Safety outcomes planned for the primary manuscript are shown in Table 3. Safety outcomes will be analyzed separately from the main effectiveness outcomes of the trial (see Primary Statistical Analysis section below).

Table 3: Other Pre-Specified Outcomes – Safety Trial Outcomes

**Safety Trial Outcomes (other pre-specified outcomes)**	
ICU Transfer [Safety Outcome]	Days to ICU transfer, between hospital days ≥ 3 and ≤ 14.
Length-of-stay [Safety Outcome]	Days from hospital admission until discharge or hospital day 14, whichever comes first, where admission day is hospital day 1.

Primary Statistical Analysis

The primary trial outcome is defined as the summed number of different ES antibiotics received during each empiric day. An empiric day is a day within the first three days of an admission. We define this outcome as the Total Empiric Extended-Spectrum Days of Therapy (ES-DOT). For clarity, the calculation is as follows: we define a DOT for a particular ES antibiotic as a day in which any number of doses of that antibiotic is given. Different ES antibiotics are summed within each empiric day for each patient admission to determine DOT in that at-risk day. Antibiotics given during an associated emergency department visit on the date of hospital admission are counted toward the ES-DOT of the first hospital day.

The main trial results will be based upon as-randomized, unadjusted analyses of admission-level ES-DOT. In contrast to the national DOT measure defined by CDC for hospitals, we define ES-DOT at the individual admission level so that we can perform analyses on individuals.

The trial will be assessed among the cohort of adult admissions who: 1) have administrative claims codes indicating an abdominal infection is present on admission; 2) received any antibiotic within 3 days of admission; and 3) were admitted to a non-ICU location. For admissions initially on a non-ICU floor and transferred to the ICU, analysis will include only admission days on the non-ICU floor within 3 days of admission.

The trial periods are defined as follows: (1) Baseline: January 1, 2019 – December 31, 2019 (12 months); (2) Phase-in: August 2, 2022 – December 31, 2022 (5 months, does not contribute to analysis); (3) Intervention: January 1, 2023 – December 30, 2023 (12 months). The main evaluation of all outcomes will be difference-in-difference between the Intervention and

Baseline periods and between study arm. Trial analysis will use a baseline period prior to the arrival of the COVID-19 pandemic since hospital operations and case mix were severely impacted by this pandemic between Winter 2020-Spring, 2022.[1-4] Although at the time of this writing (June, 2022), COVID-19 surges continue to occur nationally, clinical operations and patient case mix at most hospitals have largely returned to near pre-pandemic activity in the setting of widespread vaccination and milder virus strains. Phase-In is scheduled for August, 2022 as participating hospitals are in a state of recovery sufficient to launch the trial intervention with pharmacy/physician engagement.

The unit of analysis will be individual admissions. Individuals can contribute more than one admission. The analytic model will be a generalized linear mixed effects model for differences in differences, with random effects accounting for correlation within cluster, period-varying random effects to allow for differences within hospitals between baseline and follow-up, and admission-level random effects to account for correlation within person and hospital, if sufficient data exist. We plan to use a Poisson model for analysis of outcomes as follows:

$$log(y_{ijph}) = \beta_0 + \beta_1 A_{ijph} + \beta_2 T_{ijph} + \beta_3 A_{ijph} T_{ijph} + b_{0h} + b_{1h} T_{ijph} + g_j + \log(ed_j)$$

where y_{ijph} is the ES-DOT for patient i for admission j in period p at hospital h, and $A_{ijph} = 1$ if hospital h is in the intervention arm and 0 if not, and $T_{ijph} = 1$ if p is the intervention period and 0 if baseline period. The random effects b_{0h} and b_{1h} allow for different baseline mean admission-level ES-DOT for each hospital and each hospital in the intervention period, respectively. Equivalently, they allow for correlation within hospital at different levels at baseline and at follow-up. The random effect g_j allows each patient to have a different mean ES-DOT. Finally, ed_j is the number of empiric days in the admission.

The assessment of trial success will be determined by the significance of the arm by period interaction term β_3, which assesses whether the log relative rate of the outcome due to being in the intervention arm in the intervention period is different from 0. The exponentiated parameter estimate for β_3 is the estimated relative rate of ES-DOT per at-risk day due to the intervention, relative to the baseline period. For example, if β_3 had a negative value and a p-value <0.05, we would conclude that the patient-specific CPOE smart prompts generated a benefit over routine care. Exponentiating the parameter value would provide an estimate of the relative reduction due to the intervention in the expected ES-DOT per at-risk day.

The primary trial analysis will use an as-randomized unadjusted model with two-tailed significance set at alpha = 0.05. Secondary outcomes will be assessed using an as-randomized unadjusted model and will include adjustment for multiple comparisons with two-tailed significance set at alpha = 0.025 for the two secondary outcomes.

Subsequent analyses of the primary and secondary outcomes will include both as-treated and adjusted models. Adjusted models will account for individual characteristics such as age, gender, comorbidities, and prior history of MDROs as well as hospital characteristics such as hospital antibiotic resistance. We will also account for seasonality. All adjusters will be determined a priori. These analyses will be reported as point estimates with confidence intervals and without p-values. The reason for including these analyses is to provide additional information related to the trial outcomes for readers to assess the effects of potential confounders. The reason to not include them in a formal multiple comparisons adjustment is because these analyses are non-independent evaluations relative to the as-randomized unadjusted analyses.

We will separately evaluate intervention period effects by arm alone.

Safety Outcomes

Safety outcomes noted in the above table will be assessed in the most conservative

manner to identify potential safety issues. Safety outcomes are required because a reduction in ES-DOT might be achieved only by incorrectly withholding ES antibiotics when they were really needed. Each safety outcome will be evaluated for non-inferiority using an as-randomized unadjusted analysis with a one-tailed significance set at alpha = 0.05. Length-of-stay and days to ICU transfer are evaluated within 14 days of admission since empiric antibiotic selection within the first 3 hospital days is not expected to be the main driver of either outcome beyond this time period; evaluation of baseline data also shows that 93% of abdominal patients have length-of-stay equal to or less than 14 days. Analyses planned for these assessments are proportional hazards models with random effects for each hospital and admission These models are sometimes called frailty or shared frailty models. Because these are safety outcomes, we do not intend to make adjustments for multiple comparisons in testing them, further increasing conservatism.

All analyses will be performed using current versions of SAS (Version 9.4, as of writing, SAS Institute, Cary NC) and/or R (Version 4.0, as of writing).

C.2.11 Power Size/Sample Size Calculations

Power assessment proceeded as follows, using a Monte Carlo approach. We used available data to define a baseline period of 1/1/2019-12/31/2019. In a bootstrap procedure, individuals were selected with replacement from within each hospital, once to represent baseline data and separately to represent "intervention period" data. Hospitals were then "randomized" to intervention or control according to multiple parameters, including number of admissions with abdominal infection and ES-DOT. We then modified the outcomes for the bootstrapped individuals in the "intervention" period in the "intervention" arm to reflect the effect of the intervention.[5]

We assessed power to identify an overall 12.5% relative reduction in ES-DOT. This effect was implemented by reducing the relative empiric days of therapy for each admission which was exposed to the CPOE prompt. The amount of reduction was a uniform random variate between 0.75 and 1. For example, if the bootstrapped patient initially received 8 ES-DOT and was assigned to have a have a 12.5% reduction, then that admission intervention ES-DOT would be reduced to 7 days. The reduction of ES-DOT was selected with a lower bound determined by the investigators as the minimal effect achieved that would be clinically meaningful. Using this method, we have ≥99.9% (99.6-99.9%) power to detect this effect.

Since adoption of the intervention will be heavily influenced by the ability to demonstrate safety, our study size was based on the power to detect non-inferiority for safety outcomes. We used methods similar to the above to calculate the power to identify at least a one day increase in length-of-stay for 12.5% of patients who had hospitalizations less than 14 days and found the power was 91.5% (CI 89.6-93.2%), where non-inferiority is defined as a hazard ratio no smaller than 0.98. For days to ICU transfer, we estimated the power to detect a 2% increase in transfers to the ICU on hospital day 3 through 14 to be >99.9% (CI 99.6-99.9%), where non-inferiority is defined as having a hazard ratio no greater than 1.1 (fewer days to ICU results in a hazard ratio above 1).

For completeness, we note that there are other pre-specified outcomes intended for exploratory analysis in secondary papers for the INSPIRE ABD trial in Table 5 below.

Table 5: Secondary Manuscripts - Other Pre-Specified Outcomes

**Secondary Manuscripts (other pre-specified secondary exploratory analyses for later manuscripts)**	
Inpatient Extended-Spectrum Days of Therapy after the Empiric Period	The number of different ES antibiotics received each day, on hospital days ≥4 and ≤14.
Empiric and Total Antibiotic Costs	Empiric and total antibiotic costs during hospitalization

Incidence of Hospital-Onset *C. difficile*	Hospital-onset *C. difficile* positive tests (specimen obtained) on hospital days ≥4 and ≤14.
Incidence of Hospital-Onset MDRO-positive Cultures	Newly-detected hospital-onset MDRO-positive cultures on hospital days ≥4 and ≤14. Includes total MDRO and specific MDRO subsets.

1. Moynihan R, et al. Impact of COVID-19 pandemic on utilisation of healthcare services: a systematic review. BMJ Open 2021;11:e045343. doi:10.1136/ bmjopen-2020-045343.
2. Berkmeyer, JD, et al. The Impact Of The COVID-19 Pandemic On Hospital Admissions In The United States. Health Affairs (39):11, September, 2020. https://doi.org/10.1377/hlthaff.2020.00980
3. Richmond BK, et al. The Impact of the COVID-19 Pandemic on Surgical Practice in the Southeastern United States: Results of a Survey of the Membership of the Southeastern Surgical Congress. Am Surg. 2020 Aug;86(8):916-925. doi: 10.1177/0003134820945203. Epub 2020 Sep 14.
4. Nguyen JL, et al. Pandemic-related declines in hospitalization for non-COVID-19-related illness in the United States from January through July 2020. PLoS ONE 17(1): e0262347. https://doi.org/10.1371/journal.pone.0262347.
5. Kleinman K, Huang SS. Calculating power by bootstrap, with an application to cluster-randomized trials. eGEMs (Generating Evidence & Methods to improve patient outcomes) 2016 4:1, Article 32. doi: http://dx.doi.org/10.13063/2327-9214.1202

(Originally published in Gohil, et al. Improving Empiric Antibiotic Selection for Patients Hospitalized With Abdominal Infection: The INSPIRE 4 Cluster Randomized Clinical Trial. JAMA Surg. 2025;160(7):733–743. doi:10.1001/jamasurg.2025.1108)

INSPIRE Abdominal Infections Trial
(**IN**telligent **S**tewardship **P**rompts to **I**mprove **R**eal-time **E**mpiric Antibiotic Selection for Patients)

Statistical Analytic Plan, 3/29/2024

Ken Kleinman, ScD

Background

The **INSPIRE Abdominal Infections Trial** (INtelligent Stewardship Prompts to Improve Real-time Empiric Antibiotic Selection for Patients with Abdominal Infections) is a cluster-randomized trial of HCA Healthcare (HCA) affiliated hospitals to determine whether antibiotic prescribing practice for adult hospitalized patients admitted to non-critical care units with abdominal (ABD) infections can be affected by providing physicians with individualized risk estimates for whether a patient with an abdominal infection is likely to have an antibiotic-resistant infection. It compares routine care to a real-time smart prompt to use a standard-spectrum antibiotics when a physician orders an extended-spectrum antibiotic (ES) for a patient whose risk of antibiotic resistant infection is low (<10%). The main goal is to see if there is a difference in the empiric antibiotic prescribing practices for ES. The term "antibiotics" refers to antibacterial antibiotics in this trial. We define antibiotics administered during the first three days of hospitalization as empiric treatment because infecting pathogens and their antimicrobial susceptibilities are typically not known during this period. We refer to these first three days as the empiric period.

Extended-spectrum antibiotics are defined in Table 1 below.

Table 1: Extended-Spectrum Antibiotics in the INSPIRE-ASP Abdominal Infections Trial

Category	Extended-Spectrum Antibiotics for INSPIRE CPOE Alerts
**Anti-MRSA**	Ceftaroline, Daptomycin, Linezolid[1], Vancomycin[2]
**Anti-VRE**	Daptomycin, Linezolid[1], Tigecycline
**Anti-Pseudomonals**	Aztreonam, Cefepime, Ceftazidime, Piperacillin/tazobactam
**Anti-ESBL**	Ertapenem, Imipenem, Meropenem, Ceftolozane/tazobactam
**Anti-CRE**	Ceftazidime/avibactam, Imipenem/relebactam, Meropenem/vaborbactam, Polymixin B, Colistin, Tigecycline

[1]Both oral and intravenous (IV) formulations
[2]IV formulation only

Note: Does not include aminoglycosides or fluoroquinolones.

Abbreviations: **MRSA -** Methicillin Resistant *Staphylococcus aureus*; **VRE –** Vancomycin Resistant *Enterococci*; **ESBL –** Extended Spectrum Beta Lactamase producer; CRE – Carbapenem Resistant Enterobacteriaceae

Participating hospitals are randomized to:

- **Arm 1 – Routine Care**
- **Arm 2 – Real-Time Risk Estimation Smart Prompt**

Computerized physician order entry (CPOE) prompt that recommends standard-spectrum

antibiotics in patients admitted for ABD infections who are prescribed extended-spectrum antibiotics and calculated to have low risk (<10%) for an antibiotic resistant infection.

Trial Outcomes

Table 2: Primary and Secondary INSPIRE-ASP Abdominal Infections Trial Outcomes

Outcome	Metric
**Primary Trial Outcome**	
Total Empiric Extended-Spectrum Antibiotic Days of Therapy (ES-DOT)	The number of different extended-spectrum antibiotics received by the patient each empiric day, summed across the first 3 days of hospitalization.[1]
**Secondary Trial Outcomes**	
Vancomycin Days of Therapy per Empiric Day	The number of days Vancomycin was received by the patient on the first 3 days of hospitalization.[1]
Total Empiric Antipseudomonal Antibiotic Days of Therapy	The number of different antipseudomonal antibiotics received by the patient each empiric day, summed across the first 3 days of hospitalization.[1]

[1]E.g., if a patient is admitted for 2 days, ES-DOT will be calculated across those 2 days; however if a patient is admitted for 4 days, only the first 3 days will be evaluated.

Safety outcomes planned for the primary manuscript are shown in Table 3. Length of stay and days to ICU transfer are evaluated within 14 days of admission since empiric antibiotic selection within the first 3 hospital days is not expected to be the main driver of either outcome beyond this time; evaluation of baseline data also shows that 93% of abdominal patients have length-of-stay equal to or less than 14 days.

Table 3: Other Pre-Specified Outcomes – Trial Safety Outcomes

**Safety Trial Outcomes (other pre-specified outcomes)**	
ICU Transfer [Safety Outcome]	Days to ICU transfer, between hospital days ≥ 3 and ≤ 14.
Length-of-stay [Safety Outcome]	Days from hospital admission until discharge or hospital day 14, whichever comes first, where admission day is hospital day 1.

Data Collection Details

The trial will be assessed among the cohort of adult admissions who: 1) have administrative claims codes indicating an abdominal infection is present on admission; 2) received any antibiotic within 3 days of admission; and 3) were admitted to a non-ICU location.

The rationale for including only the first three days of the admission is that this is when the actual status of the patient with respect to the need for an ES antibiotic is unknown. Bearing this in mind, antibiotics given during an associated emergency department visit on the date of hospital admission are counted toward the ES-DOT of the first hospital day.

The trial baseline period includes January 1, 2019 – December 31, 2019 (12 months). The intervention period includes January 1, 2023 – December 31, 2023 (12 months). The baseline was selected to include time prior to the arrival of the COVID-19 pandemic since hospital operations and case mix were dramatically affected between Winter 2020-Spring, 2022.[1-4] Although COVID-19 surges have continued to occur, clinical operations and patient case mix at most hospitals have largely returned to near pre-pandemic activity. We considered that a state of pre-pandemic normalcy returned by summer of 2022, and introduced our intervention on August 2, 2022. The period between then and December 31, 2022 is considered a phase-in period and is not used in trial evaluation.

Primary Statistical Analysis

The primary trial outcome is defined as the summed number of different ES antibiotics received during each empiric day, on the patient-admission level. An empiric day is a day within the first three days of an admission. We define this outcome as the Total Empiric Extended-Spectrum Days of Therapy (ES-DOT). For clarity, the calculation is as follows: we define a DOT for a particular ES antibiotic as a day in which any number of doses of that antibiotic is given. Different ES antibiotics are summed within each empiric day for each patient admission to determine DOT in that at-risk day.

The main trial results will be based upon as-randomized, unadjusted analyses of admission-level ES-DOT. We note that the national DOT measure defined by CDC is measured at the hospital level. We define ES-DOT at the individual admission level so that we can perform analyses on individuals.

The main evaluation of all outcomes will be based on the difference in differences between the intervention and baseline periods and between study arms.

The unit of analysis will be individual admissions. Individual patients can contribute more than one admission. The analytic model will be a generalized linear mixed effects model for differences in differences, with random effects to account for correlation within hospital between period, within hospital within period between patient, and within patient between admission. We plan to use a Poisson model for analysis of outcomes as follows:

$$log(y_{ijph}) = \beta_0 + \beta_1 A_{ijph} + \beta_2 T_{ijph} + \beta_3 A_{ijph} T_{ijph} + b_{0h} + b_{1h} T_{ijph} + g_j + \log{(ed_j)}$$

where y_{ijph} is the ES-DOT for patient i for admission j in period p at hospital h, and $A_{ijph} = 1$ if hospital h is in the intervention arm and 0 if not, and $T_{ijph} = 1$ if p is the intervention period and 0 if baseline period. The random effects b_{0h} and b_{1h} allow for different baseline mean admission-level ES-DOT for each hospital and each hospital in the intervention period, respectively. Equivalently, they allow for correlation within hospital at different levels at baseline and at follow-up. The random effect g_j allows each patient to have a different mean ES-DOT. Finally, ed_j is the number of empiric days in the admission. We note that it may not be possible to fit models with the g_j parameter, for various numerical and computational reasons—the most prominent being a relative sparsity of patients with readmissions. If that proves to be the case, we will randomly select one admission per patient and use the model above without that parameter and omitting the j subscript.

The assessment of trial success will be determined by assessing the significance of the arm by period interaction term β_3, which estimates the log relative rate of the outcome due to being in the intervention arm in the intervention period. The exponentiated parameter estimate for β_3 is the estimated relative rate of ES-DOT per at-risk day due to the intervention, relative to the baseline period. For example, if β_3 had a negative value and a p-value <0.05, we would conclude that the patient-specific CPOE smart prompts generated a benefit over routine care. Exponentiating the parameter value would provide an estimate of the relative reduction due to the intervention in the expected ES-DOT per at-risk day.

The primary trial analysis will use an as-randomized unadjusted model with two-tailed significance set at alpha = 0.05. Secondary outcomes will be assessed using an as-randomized unadjusted model and will include adjustment for multiple comparisons with two-tailed significance set at alpha = 0.025 for the two secondary outcomes.

Subsequent analyses of the primary and secondary outcomes will include both as-treated and adjusted models. Adjusted models will account for individual characteristics such as age, gender, comorbidities, and prior history of MDROs as well as hospital characteristics such as hospital antibiotic resistance. We will also account for seasonality. All adjusters will be determined a priori. These analyses will be reported as point estimates with confidence intervals and without p-values. The reason for including these analyses is to provide additional

information related to the trial outcomes for readers to assess the effects of potential confounders. The reason to not include them in a formal multiple comparisons adjustment is because these analyses are non-independent evaluations relative to the as-randomized unadjusted analyses.

Safety Outcomes

Safety outcomes are required because a reduction in ES-DOT might be achieved only by incorrectly withholding ES antibiotics when they were necessary. Each safety outcome will be evaluated for non-inferiority using an as-randomized unadjusted analysis with a one-tailed significance set at alpha = 0.05. For length of stay, the non-inferiority margin is a hazard ratio of 0.98. For days to ICU transfer, the non-inferiority margin is a hazard ratio of 1.1 (fewer days to ICU results in a hazard ratio above 1). Analyses planned for these assessments are proportional hazards models with random effects for each time period, hospital, and admission. These models are sometimes called frailty or shared frailty models. Because these are safety outcomes, we do not intend to make adjustments for multiple comparisons in testing them, further increasing conservatism. As above, it may not be possible to include multiple admissions.

All analyses will be performed using current versions of SAS/STAT and/or R packages lme4 and coxme, unless better packages become available in the meantime.

Further Planned Analyses

We note that there are other pre-specified outcomes intended for exploratory analysis in secondary papers for the INSPIRE ABD trial in Table 5 below.

Table 5: Secondary Manuscripts - Other Pre-Specified Outcomes

**Secondary Manuscripts (other pre-specified secondary exploratory analyses for later manuscripts)**	
Inpatient Extended-Spectrum Days of Therapy after the Empiric Period	The number of different ES antibiotics received each day, on hospital days ≥4 and ≤14.
Empiric and Total Antibiotic Costs	Empiric and total antibiotic costs during hospitalization
Incidence of Hospital-Onset *C. difficile*	Hospital-onset *C. difficile* positive tests (specimen obtained) on hospital days ≥4 and ≤14.
Incidence of Hospital-Onset MDRO-positive Cultures	Newly-detected hospital-onset MDRO-positive cultures on hospital days ≥4 and ≤14. Includes total MDRO and specific MDRO subsets.

6. Moynihan R, et al. Impact of COVID-19 pandemic on utilisation of healthcare services: a systematic review. BMJ Open 2021;11:e045343. doi:10.1136/ bmjopen-2020-045343.
7. Berkmeyer, JD, et al. The Impact Of The COVID-19 Pandemic On Hospital Admissions In The United States. Health Affairs (39):11, September, 2020. https://doi.org/10.1377/hlthaff.2020.00980
8. Richmond BK, et al. The Impact of the COVID-19 Pandemic on Surgical Practice in the Southeastern United States: Results of a Survey of the Membership of the Southeastern Surgical Congress. Am Surg. 2020 Aug;86(8):916-925. doi: 10.1177/0003134820945203. Epub 2020 Sep 14.
9. Nguyen JL, et al. Pandemic-related declines in hospitalization for non-COVID-19-related illness in the United States from January through July 2020. PLoS ONE 17(1): e0262347. https://doi.org/10.1371/journal.pone.0262347.

Arm 1:
INSPIRE Routine Stewardship

Abdominal & Skin/Soft Tissue Infection Trials

Toolkit Binder

Funded by the National Institutes of Health (NIH)

Welcome

Welcome to the **INSPIRE-ASP** (**IN**telligent **S**tewardship **P**rompts to **I**mprove **R**eal-time **E**mpiric **A**ntibiotic **S**election for **P**atients) Trials. The INSPIRE-ASP Abdominal and INSPIRE-ASP Skin & Soft Tissue Infection Trials are cluster-randomized trials of 102 HCA Healthcare hospitals to assess best practice in antibiotic stewardship. These trials are a partnership between HCA Healthcare and academic investigators who are federally funded by the National Institutes of Health (NIH).

Summary of Goals

Up to 40% of patients who are prescribed antibiotics in U.S. hospitals either do not require these drugs or receive inappropriate antibiotics for the condition being treated. Moreover, inappropriate broad-spectrum antibiotics are often prescribed empirically due to concerns about infection with multidrug-resistant organisms (MDROs), despite the low risk of MDRO infection in the vast majority of clinical settings. This is the case for patients admitted with abdominal or skin/soft tissue infections, two common conditions for antibiotic use in hospitalized patients. These trials will compare two strategies to determine best practices for empiric antibiotic selection. Arm 1, Routine Care, will continue antibiotic stewardship practices according to current facility strategies. Arm 2, Smart Prompt Intervention, will implement a precision medicine smart prompt using a computerized physician order entry (CPOE) alert that provides clinicians with a patient's individual probability of MDRO infection and recommends a standard-spectrum antibiotic for patients at low risk for MDRO abdominal or skin/soft tissue infections in non-critical care units. This trial will provide real-time essential information to clinicians to support and optimize empiric antibiotic selection.

Funded by National Institute of Allergy and Infectious Diseases

INtelligent Stewardship Prompts to Improve Real-time Empiric Antibiotic Selection for Patients

Arm 1 – Routine Stewardship

Trial Protocol Implementation Toolkit
Table of Contents

Title **Page**

Welcome & Summary of Goals 2

Table of Contents 3

INSPIRE Team 4

Study Champion Roles and Responsibilities 7

Study Champion FAQs 8

Smart Prompt Workflow 10

Funded by National Institute of Allergy and Infectious Diseases

Study Investigators and Stakeholders

University of California Irvine
Shruti Gohil, MD MPH, Lead Investigator
Susan Huang, MD MPH, Senior Investigator

Harvard Medical School/Harvard Pilgrim Health Care Institute
Ed Septimus, MD FACP FIDSA FSHEA
Richard Platt, MD MSc

HCA Healthcare
Jackie Blanchard, MSN, RN, CIC, VP, Infection Prevention
Chenette Burks, PharmD, Director Pharmacy Services
Will Cooper, PharmD CPHIMS, Manager, Clinical Pharmacy Informatics
Michael Cuffe, MD, Executive VP and Chief Clinical Officer
Samir Fakhry, MD, VP, Center for Trauma and Acute Care Surgery Research
Robert Foster, RN, VP of Emergency Services
Jeffrey Guy, MD, MSc, VP, Care Process Design and Clinical Engineering
Karla Miller, PharmD, VP Pharmacy Services, Chief Patient Safety Officer
Julia Moody, MS, SM (ASCP), Clinical Director, Infection Prevention
Russell Poland, PhD, AVP, Research and Extramural Collaborations/Partnerships
Risa Rahm, PharmD CPHIMS, Senior Director, Clinical Pharmacy Informatics
Kaleb Roemer, PharmD, Manager, Clinical Pharmacy Services
Kenneth Sands, MD MPH, VP Chief Epidemiologist

Brigham & Women's Hospital
David Kubiak, PharmD

University of Massachusetts Amherst
Ken Kleinman, ScD

Rush University
Robert A. Weinstein, MD
Mary Hayden, MD

Funded by National Institute of Allergy and Infectious Diseases

Study Administrative Team

University of California Irvine

Victoria Ngo, BS, Assistant Clinical Research Coordinator

Harvard Medical School/Harvard Pilgrim Health Care Institute

Taliser Avery, MS, Lead Research Analyst

Micaela Coady, MS, Senior Program Manager

Allison Froman, MPH, Research Analyst

Selsebil Sljivo, MPH, Senior Project Coordinator

Neha Varma, MPH, Senior Research Analyst

Chamaine Washington, MPH, Project Coordinator

HCA Healthcare

Thomas Johnson, Senior MEDITECH Programmer & NPR Report Writer

Laura McLean, M.Ed, Senior Data Analyst

Brittany Meador, BS, Program Coordinator, Extramural Collaborations & Partnerships

Naoise Nickolay, RPh, Pharmacy Informatics Consultant

Kimberly Smith, MBA, Director, Research Operations

Funded by National Institute of Allergy and Infectious Diseases

Study Champion Roles and Responsibilities

Pharmacy & Physician Study Champions will partner with the INSPIRE investigative team and are expected to:

1. Join trial coaching calls
2. Actively engage and educate staff, leadership, and other stakeholders on the value of participating in the INSPIRE Trials
3. Support current antimicrobial stewardship practices
4. Avoid and/or report interventions or activities that may impact trial outcomes or those targeting empiric antibiotic selection for abdominal and skin/soft tissue infection interventions

Funded by National Institute of Allergy and Infectious Diseases

INSPIRE Abdominal and Skin & Soft Tissue Infection Trials
Study Champion FAQs – Arm 1

What are the INSPIRE-ASP Trials?
The INSPIRE-ASP (**IN**telligent **S**tewardship **P**rompts to **I**mprove **R**eal-time **E**mpiric **A**ntibiotic **S**election for **P**atients) Trials are two separate cluster-randomized trials of HCA Healthcare hospitals to test an antibiotic stewardship intervention abdominal (ABD) and skin and soft tissue (SST) infections.

- **Arm 1** – **Continue usual, gold-standard antibiotic stewardship activities**
- **Arm 2** – **CPOE Smart Prompt Alert Intervention**
    1. Provides patient-specific risk estimate for ABD or SST infections due to a multi-drug resistant organism (MDRO) when extended-spectrum (ES) antibiotics are ordered
    2. Recommends standard antibiotics in non-ICU patients with low (<10%) MDRO risk

What is the study timeline?
- **Prompt go-live** – August 2, 2022
- **Phase-In** (5 months) – August-December, 2022
- **Intervention** (12 months) – January-December, 2023

What are the outcomes of the INSPIRE-ASP Trials
The goal is to identify a best practice strategy for choosing empiric (within 3 calendar days) antibiotics based on a patient's risk for MDRO infection. Outcomes include:

- **Primary**: Empiric extended-spectrum days of therapy
- **Secondary**: Empiric Vancomycin (ABD infection only) and antipseudomonal days of therapy
- **Safety outcomes**: length of stay, ICU transfers
- **Other**: post-empiric ES antibiotic use, MDRO and *C. difficile* rates, antibiotic costs

Why does INSPIRE focus on ABD and SST infections?
ABD and SST infections are among the most common reasons for infection-related admission and ES antibiotic use. Yet, few studies have evaluated effective empiric antibiotic stewardship strategies for these infections.

What will be required of participating Arm 1 sites?
Study champions will be asked to:

- Join trial coaching calls
- Actively engage and educate staff, leadership, and other stakeholders on the value of participating in the INSPIRE Trials
- Continue usual antibiotic stewardship activities
- Avoid and/or report interventions or activities that may impact trial outcomes or those targeting empiric antibiotic selection for abdominal and skin/soft tissue infection interventions

How do we avoid competing interventions and how are these defined?

Participating facilities are fully expected to continue their stewardship activities and initiatives but should avoid activities that may directly impact trial outcomes. Such activities are anticipated to be rare. Examples of interventions that may directly conflict with the trial include:

- A new stewardship strategy that aims to reduce the use of ES antibiotics in the Emergency Department (ED) or within 3 days of admission trial coaching calls
- A new real-time antibiotic de-escalation strategy in the ED or within 3 calendar days of admission
- A new rapid diagnostic tool for ABD or SST infection that would be used in the empiric period to guide antibiotic therapy within 3 days of admission
- Ordering antibiotics through Patient Keeper

Has this study received IRB approval? Will patients need to give informed consent?

Yes. Harvard Pilgrim's IRB will provide ethical oversight for these trials and serve as the central IRB. Both INSPIRE ABD and SST infection trials have been approved by the Harvard IRB. Your hospital has submitted all ceding and reliance agreements. Informed consent will not be required. This study has been approved as a minimal risk study and will be implemented as a quality improvement (QI) protocol through the existing infrastructure of your hospital's antibiotic stewardship programs. The assigned protocol will be approved through your usual clinical/leadership committees and become hospital guidance on appropriate empiric antibioticuse.

Who can I contact for more information?

INSPIRE Study Team
Research Coordinator
HCAINSPIRE@gmail.com, **949-824-7806**

Lead Investigator – Shruti Gohil, MD, MPH, UC Irvine
skgohil@hs.uci.edu

Co-Investigator – Edward Septimus, MD, FACP, Senior Lecturer HPHCI
eseptimus@gmail.com

Manager, Clinical Pharmacy Services – Kaleb Roemer, PharmD
Kaleb.Roemer@hcahealthcare.com

Director, Clinical Pharmacy Services – Chenette Burks, PharmD
Ruby.Burks@hcahealthcare.com

Funded by National Institute of Allergy and Infectious Diseases

Arm 1 – Antibiotic Indication Screen Workflow

The requirement for providing an indication for antibiotic use is a national standard for antibiotic stewardship. HCA Healthcare originally released antibiotic indication screens in 2017 and are currently implemented in the emergency department and inpatient areas throughout HCA Healthcare facilities nationwide.

As an Arm 1 facility, our hospital's primary role is to serve as the gold standard against which intervention (Arm 2) can be compared. To ensure that the antibiotic indication screen workflow is balanced between both study arms, beginning **August 2, 2022** the following screens will appear when extended-spectrum antibiotics are ordered**.**

INSPIRE Arm 1 Abdominal/Skin & Soft Tissue Infection Workflow

1. **When an extended-spectrum antibiotic is ordered for patients with abdominal or skin/soft tissue infection AND the patient is located in the ED at the time of order, then** you will be asked whether the patient is ICU-bound (already live for patients with pneumonia and UTI).

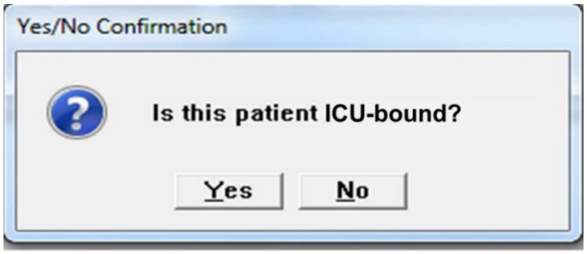

2. **For abdominal patients only, if Anti-MRSA and antipseudomonal antibiotics are ordered and patient has a history of MRSA or antipseudomonal antibiotics then** you will be asked whether the patient has a history of abdominal surgery.

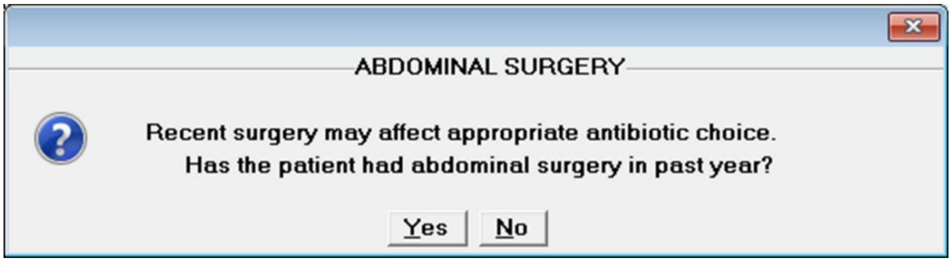

Funded by National Institute of Allergy and Infectious Diseases

Arm 2:
INSPIRE Stewardship Bundle

Abdominal & Skin/Soft Tissue Infection Trials

Toolkit Binder

Funded by the National Institutes of Health (NIH)

INSPIRE
BETTER SAFER ANTIBIOTIC USE

Welcome

Welcome to the **INSPIRE-ASP** (**IN**telligent **S**tewardship **P**rompts to **I**mprove **R**eal-time **E**mpiric **A**ntibiotic **S**election for **P**atients) Trials. The INSPIRE-ASP Abdominal and INSPIRE-ASP Skin & Soft Tissue Infection Trials are cluster-randomized trials of 102 HCA Healthcare hospitals to assess best practice in antibiotic stewardship. These trials are a partnership between HCA Healthcare and academic investigators who are federally funded by the National Institutes of Health (NIH).

Summary of Goals

Up to 40% of patients who are prescribed antibiotics in U.S. hospitals either do not require these drugs or receive inappropriate antibiotics for the condition being treated. Moreover, inappropriate broad-spectrum antibiotics are often prescribed empirically due to concerns about infection with multidrug-resistant organisms (MDROs), despite the low risk of MDRO infection in the vast majority of clinical settings. This is the case for patients admitted with abdominal or skin/soft tissue infections, two common conditions for antibiotic use in hospitalized patients. These trials will compare two strategies to determine best practices for empiric antibiotic selection. Arm 1, Routine Care, will continue antibiotic stewardship practices according to current facility strategies. Arm 2, Smart Prompt Intervention, will implement a precision medicine smart prompt using a computerized physician order entry (CPOE) alert that provides clinicians with a patient's individual probability of MDRO infection and recommends a standard-spectrum antibiotic for patients at low risk for MDRO abdominal or skin/soft tissue infections in non-critical care units. This trial will provide real-time essential information to clinicians to support and optimize empiric antibiotic selection.

DPM HCA Healthcare UC Irvine Health BWH
Funded by National Institute of Allergy and Infectious Diseases

INtelligent Stewardship Prompts to Improve Real-time Empiric Antibiotic Selection for Patients

Arm 2 – Smart Prompt

Trial Protocol Implementation Toolkit
Table of Contents

Title **Page**

Welcome & Summary of Goals... 2
Table of Contents 3
INSPIRE Team 4
Study Champion Materials 7
Clinician Education Materials 17
Clinician Hot Topics 27
INSPIRE Presentation 32
Smart Prompt Workflow 72
Promotional Material 78

Funded by National Institute of Allergy and Infectious Diseases

Study Investigators and Stakeholders

University of California Irvine
Shruti Gohil, MD MPH, Lead Investigator
Susan Huang, MD MPH, Senior Investigator

Harvard Medical School/Harvard Pilgrim Health Care Institute
Ed Septimus, MD FACP FIDSA FSHEA
Richard Platt, MD MSc

HCA Healthcare
Jackie Blanchard, MSN, RN, CIC, VP, Infection Prevention
Chenette Burks, PharmD, Director Pharmacy Services
Will Cooper, PharmD CPHIMS, Manager, Clinical Pharmacy Informatics
Michael Cuffe, MD, Executive VP and Chief Clinical Officer
Samir Fakhry, MD, VP, Center for Trauma and Acute Care Surgery Research
Robert Foster, RN, VP of Emergency Services
Jeffrey Guy, MD, MSc, VP, Care Process Design and Clinical Engineering
Karla Miller, PharmD, VP Pharmacy Services, Chief Patient Safety Officer
Julia Moody, MS, SM (ASCP), Clinical Director, Infection Prevention
Russell Poland, PhD, AVP, Research and Extramural Collaborations/Partnerships
Risa Rahm, PharmD CPHIMS, Senior Director, Clinical Pharmacy Informatics
Kaleb Roemer, PharmD, Manager, Clinical Pharmacy Services
Kenneth Sands, MD MPH, VP Chief Epidemiologist

Brigham & Women's Hospital
David Kubiak, PharmD

University of Massachusetts Amherst
Ken Kleinman, ScD

Rush University
Robert A. Weinstein, MD
Mary Hayden, MD

Funded by National Institute of Allergy and Infectious Diseases

Study Administrative Team

University of California Irvine

Victoria Ngo, BS, Assistant Clinical Research Coordinator

Harvard Medical School/Harvard Pilgrim Health Care Institute

Taliser Avery, MS, Lead Research Analyst

Micaela Coady, MS, Senior Program Manager

Allison Froman, MPH, Research Analyst

Selsebil Sljivo, MPH, Senior Project Coordinator

Neha Varma, MPH, Senior Research Analyst

Chamaine Washington, MPH, Project Coordinator

HCA Healthcare

Thomas Johnson, Senior MEDITECH Programmer & NPR Report Writer

Laura McLean, M.Ed, Senior Data Analyst

Brittany Meador, BS, Program Coordinator, Extramural Collaborations & Partnerships

Naoise Nickolay, RPh, Pharmacy Informatics Consultant

Kimberly Smith, MBA, Director, Research Operations

Funded by National Institute of Allergy and Infectious Diseases

INSPIRE Trials: Arm 2

Study Champion Materials

This section includes:

- Study Champion Roles and Responsibilities
- Study Champion Intervention Checklist
- Study Champion FAQs
- Smart Prompt Implementation Protocol

➢

Study Champion Roles and Responsibilities

Pharmacy & Physician Study Champions will partner with the INSPIRE investigative team and are expected to:

1. Join trial coaching calls
2. Actively engage and educate hospital clinicians, leadership, and other stakeholders on the value and safety of the INSPIRE Trials
3. Develop and implement strategies to educate and disseminate study materials to reach all prescribers at your facility
4. Review clinician progress reports and encourage reductions in antibiotic use
5. Avoid and/or report interventions or activities targeting empiric antibiotic selection for abdominal and skin/soft tissue infection that may impact trial outcomes

Funded by National Institute of Allergy and Infectious Diseases

INSPIRE Study Champion Intervention Checklist

As Study Champion for the INSPIRE Abdominal & Skin/Soft Tissue (SST) Trials, there are several key things you can do to ensure the INSPIRE Prompt launches smoothly:

Engage key leaders & stakeholders to help educate their peers

- ☐ Hospitalist, ED, Surgery (General, Trauma, Vascular, OB/GYN) leaders
- ☐ Graduate Medical Education Program Directors
- ☐ Pharmacy (e.g., managers, directors)
- ☐ Other prescribers (Physician Assistants, Nurses, Nurse Practitioners, Pharmacists)

Get the word out and educate frontline clinicians/prescribers

- ☐ Present INSPIRE at medical staff/faculty meetings or grand rounds
- ☐ Disseminate educational materials:
    - ☐ Flyers, posters, one-page INSPIRE summary in physician lounges
    - ☐ "Meet-and-greet" clinicians on the floors with handouts, FAQs
    - ☐ Encourage clinicians attend study webinars or access information on Atlas/Media Connect

Assure existing order sets are aligned with INSPIRE prompts

- ☐ Assess sepsis, abdominal infection, and SST infection order sets for potential conflicts or redundancies with INSPIRE CPOE prompts
    - ☐ Work with local and corporate IT to resolve as appropriate
- ☐ Examples include:
    - ☐ Sepsis order set contains only extended-spectrum (ES) antibiotic options for abdominal/SST infections. CPOE smart prompt alert will recommend ceftriaxone when MDRO risk is low. This may be seen by clinicians as a potentially conflicting recommendation.
    - ☐ **Possible solution(s):** (a) Add ceftriaxone to list of antibiotics in sepsis order sets. (b) Make two separate order sets, one for ICU and one for non-ICU patients since the smart prompt only alerts for non-ICU patients

Identify and educate other clinicians involved in antibiotic ordering

- ☐ Assess whether antibiotics are commonly given as verbal orders and if so, which staff take verbal orders (e.g., pharmacists, nurses, physician assistants)
- ☐ If appropriate, include leaders and staff from these areas in your education efforts using INSPIRE study materials
  - ☐ The "Verbal Order Checklist" in this toolkit provides an overview of questions to ask physicians when taking verbal orders for ES antibiotics, including a suggested script to use

Give clinicians feedback on antibiotic use

- ☐ Regularly review Tableau reports on physician antibiotic use and override reasons
- ☐ Look for physician prescribing patterns for sepsis, abdominal, and skin/soft tissue infections and education opportunities
- ☐ Share reports with leadership, stewardship/other pharmacy and physician staff
- ☐ Provide direct physician education and feedback.
  For example:
  - ☐ Send physicians emails with their individual ES antibiotic use monthly
  - ☐ Bring physician-specific antibiotic use reports with you when rounding on the floors to provide direct verbal feedback
  - ☐ Identify and address education opportunities that become apparent after review of override reasons or conversations with physicians

Contact study investigators for assistance

Always feel free to contact investigators for help in addressing clinician questions

INSPIRE Study Team (Research Coordinator – Victoria Ngo), UC Irvine
HCAINSPIRE@gmail.com, 949-824-7806

Lead Investigator – Shruti Gohil, MD, MPH, UC Irvine
skgohil@hs.uci.edu

Co-Investigator – Edward Septimus, MD, FACP, Senior Lecturer HPHCI
eseptimus@gmail.com

Manager, Clinical Pharmacy Services – Kaleb Roemer, PharmD
Kaleb.Roemer@hcahealthcare.com

Director, Clinical Pharmacy Services – Chenette Burks, PharmD
Ruby.Burks@hcahealthcare.com

Funded by National Institute of Allergy and Infectious Diseases

INSPIRE Abdominal and Skin & Soft Tissue Infection Trials
Study Champion FAQs

What are the INSPIRE-ASP Trials?

The INSPIRE-ASP (**IN**telligent **S**tewardship **P**rompts to **I**mprove **R**eal-time **E**mpiric **A**ntibiotic **S**election for **P**atients) Trials are two separate cluster-randomized trials of HCA Healthcare hospitals to test an antibiotic stewardship intervention abdominal (ABD) and skin and soft tissue (SST) infections.

- **Arm 1** – **Routine Care**
- **Arm 2** – **CPOE Smart Prompt Alert Intervention**
    1. Provides patient-specific risk estimate for ABD or SST infections due to a multi-drug resistant organism (MDRO) when extended-spectrum (ES) antibiotics are ordered
    2. Recommends standard antibiotics in non-ICU patients with low (<10%) MDRO risk

When will the computerized physician order entry (CPOE) smart prompt fire?

- **Prompts fire when ES antibiotics are ordered within 3 days ofadmission**
- Only for **adults** in **ED or non-intensive care units (ICU)** admitted for **ABD or SST infections**
- Risk of antibiotic resistant infection is calculated & recorded using **real-time, patient-specific data**
    - **If ≥ 10%**, **extended-spectrum antibiotic allowed**
    - **If <10%**, **prompt recommends standard antibiotics**

Which antibiotics are considered extended-spectrum?

Category	Extended-Spectrum Antibiotics for INSPIRE CPOE Alerts[1]
**Anti-MRSA**	Ceftaroline, Daptomycin, Linezolid, Vancomycin[2]
**Anti-VRE**	Daptomycin, Linezolid
**Anti-Pseudomonal**	Aztreonam, Cefepime, Ceftazidime, Piperacillin/Tazobactam
**Anti-ESBL**	Ertapenem, Imipenem, Meropenem, Ceftolozane/Tazobactam

[1]Additional restricted antibiotics will not activate INSPIRE alerts, [2]IV formulation only

How is the INSPIRE CPOE prompt "smart"?

The CPOE smart prompt uses your patient's risk factors (from medical record data) to calculate MDRO risk for abdominal or skin/soft tissue infection. Risk estimates are tailored to the antibiotic selected. Example:

- If **ceftriaxone** ordered → **no prompt** fires at all (standard-spectrum antibiotic)
- If **vancomycin** ordered → **returns patient's MRSA risk**
- If cefepime or **piperacillin/tazobactam** ordered → returns ***Pseudomonas* risk**
- If **linezolid** ordered → returns **MRSA and VRE risk**
- If **meropenem** ordered → returns **ESBL + resistant gram-negative bacteria**

What is the study timeline?

- **Prompt go-live** – August 2, 2022
- **Phase-In** (5 months) – August-December, 2022
- **Intervention** (12 months) – January-December, 2023

What are the outcomes of the INSPIRE-ASP Trials

The goal is to identify a best practice strategy for choosing empiric (within 3 calendar days) antibiotics based on a patient's risk for MDRO infection. Outcomes include:

- **Primary**: Empiric extended-spectrum days of therapy
- **Secondary**: Empiric Vancomycin (ABD infection only) and antipseudomonal days of therapy
- **Safety outcomes**: length of stay, ICU transfers
- **Other**: post-empiric ES antibiotic use, MDRO and *C. difficile* rates, antibiotic costs

Which patients are included?

Only adults admitted to a non-ICU ward with ABD or SST infection who are ordered to receive an extended-spectrum antibiotic within 3 days of admission will be included in the smart prompt alerts. The INSPIRE smart prompts are programmed to not fire for patients younger than 18 years or who are in an ICU location. Patients admitted from jail or prison will not be included in this study.

How does the INSPIRE CPOE prompt exclude ICU patients?

1. Does not fire if a patient is located in the ICU.
2. For emergency department, smart prompt asks clinicians if the patient is ICU-bound and does not fire if clinician responds "yes".
3. If smart prompt fires and patient is going to the ICU, clinicians can override theprompt.

What are the INSPIRE CPOE risk estimates based on?

The INSPIRE study team assessed over 422,000 patients admitted to HCA Healthcare hospitals over 3 years. We evaluated **(1) your hospital antibiogram** for ABD or SST infection patients and **(2) patient risk factors of MDRO infection**, including demographics (age, gender, race, ethnicity, insurance status), comorbid conditions, history of MDRO, history of prior surgery, history of prior HCA hospital, ED, or nursing home admission, prior antibiotics, and admission lab values.

Using a special statistical method, we identified which risk factors were most often found in patients who developed ABD or SST infection with an MDRO. Using these risk factors (which are different for every MDRO), the CPOE smart prompt gives the patient's **individual absolute risk** for infection with the MDRO(s) targeted by the antibiotic ordered.

What does the INSPIRE CPOE prompt account for at the moment of order entry?

The prompt can only account for information available in your hospital's Meditech system at the time of order entry. **The prompt can capture**:

- **History of MDRO** from microbiology data as far back as available in Meditech through 2012
- **Cultures resulted in Meditech at your facility 3 or more days ago**

The prompt cannot capture:

- MDRO history or cultures from an **outside hospital**
- **Culture results < 3 days** from admission
    - Monitor all new cultures as they become available and adjust accordingly

Will risk estimates be validated after the intervention starts?

The INSPIRE risk estimates models are robust and based on 3 years of data across the HCA Healthcare hospital enterprise. Estimates for a single facility are unlikely to change significantly within 1 year. Our evaluation also shows that facility MDRO prevalence appears stable over the short term. Risk estimates will be re-validated periodically as needed.

What if someone takes a verbal order for antibiotics?

The INSPIRE prompt fires in the following CPOE modules within Meditech: "OM" (Order Management), "EDM" (Emergency Department Management), and PWM (Provider/Physician Workflow Management). **It will NOT fire in the "PHA" (Pharmacy) module.** We ask all staff taking verbal orders (including "vancomycin per pharmacy dosing" orders) to place them using OM, EDM, or PWM modules. Staff taking verbal orders will also need pertinent information from physicians to be able to respond to the INSPIRE prompt, such as (1) what infection the antibiotic is meant to treat and (2) whether the physician would agree to standard-spectrum antibiotics if the risk is found to be low (<10%). Please see the "Verbal Order Checklist for Pharmacists and Nurses" which guides how verbal orders are best handled.

Why does INSPIRE focus on ABD and SST infections?

ABD and SST infections are among the most common reasons for infection-related admission and ES antibiotic use. Yet, few studies have evaluated effective empiric antibiotic stewardship strategies for these infections.

What will be required of participating sites?

Please reference the "Study Champion Intervention Checklist" of key things you can do to ensure the INSPIRE CPOE smart prompt launches successfully at your facility. Study champions will be asked to:

- Join trial coaching calls
- Engage and educate clinicians on value and safety of the INSPIRE-ASPTrials
- Review clinician quarterly progress reports and encourageimprovements
- Address clinician outliers in a supportive, knowledgeable way
- Set reachable local targets and improvement goals
- Report interventions or activities that may impact trial outcomes

How do we avoid competing interventions and how are these defined?

Participating facilities are fully expected to continue their stewardship activities and initiatives but should avoid activities that may directly impact trial outcomes. Such activities are anticipated to be rare. Examples of interventions that may directly conflict with the trial include:

- A new stewardship strategy that aims to reduce the use of ES antibiotics in the Emergency Department (ED) or within 3 days of admission trial coaching calls
- A new real-time antibiotic de-escalation strategy in the ED/within 3 calendar days
- A new rapid diagnostic tool for ABD or SST infection that would be used in the empiric period to guide antibiotic therapy within 3 days of admission
- Ordering antibiotics through Patient Keeper

Will INSPIRE impact ED sepsis core measures?

The INSPIRE prompt **does NOT** affect "time to antibiotics" because it does not impact whether antibiotics should be used but rather gives real-time information to help decide whether extended- or standard-spectrum antibiotics should be used. The standard-spectrum antibiotics in the INSPIRE prompts are fully compliant with core measures.

Has this study received IRB approval? Will patients need to give informed consent?

Yes. Harvard Pilgrim's IRB will provide ethical oversight for these trials and serve as the central IRB. Both INSPIRE ABD and SST infection trials have been approved by the Harvard IRB. Your hospital has submitted all ceding and reliance agreements. Informed consent will not be required. This study has been approved as a minimal risk study and will be implemented as a quality improvement (QI) protocol through the existing infrastructure of your hospital's antibiotic stewardship programs. The assigned protocol will be approved through your usual clinical/leadership committees and become hospital guidance on appropriate empiric antibioticuse.

Who can I contact for more information?

INSPIRE Study Team
Research Coordinator – Victoria Ngo, UC Irvine
HCAINSPIRE@gmail.com, 949-824-7806

Lead Investigator – Shruti Gohil, MD, MPH, UC Irvine
skgohil@hs.uci.edu

Co-Investigator – Edward Septimus, MD, FACP, Senior Lecturer HPHCI
eseptimus@gmail.com

Manager, Clinical Pharmacy Services – Kaleb Roemer, PharmD
Kaleb.Roemer@hcahealthcare.com

Director, Clinical Pharmacy Services – Chenette Burks, PharmD
Ruby.Burks@hcahealthcare.com

Funded by National Institute of Allergy and Infectious Diseases

INSPIRE Smart Prompt Implementation Protocol

The **INSPIRE Abdominal and Skin/Soft Tissue (ABD/SST) trials** use a computerized physician order entry (CPOE) alert to provide clinicians with (1) patient-specific risk estimates for infection due to an antibiotic-resistant bacteria and (2) recommendations on the standard-spectrum empiric antibiotic selection based on individualized risk estimates. This intervention will improve the use of extended-spectrum (ES) antibiotics for non-intensive care unit (ICU) patients at low risk of having abdominal or skin/soft tissue infection due to an antibiotic-resistant bacteria.

This document provides pharmacists and clinicians with an overview of the protocol by which antibiotic selection should occur when INSPIRE CPOE alerts are activated at your facility.

Empiric Antibiotic Selection Process

1. **The CPOE alert is activated when:**
   a. Clinicians order an extended-spectrum antibiotic(s) for abdominal (ABD) or skin/soft tissue infection (SST) in non-ICU patients within the first 3 days of admission, including in the emergency department.
   b. No prompt will be triggered if standard-spectrum antibiotics are ordered (e.g. antibiotics that are not listed in Table 1, such as ceftriaxone or ampicillin/sulbactam.
2. **The CPOE alert provides clinicians with patient-specific risk estimates for antibiotic-resistant infection.**
   a. Risk estimates are calculated for the antibiotic-resistant organism(s) targeted by the antibiotic ordered (Table 1).
      i. Example 1: When a clinician orders vancomycin for ABD infection, the CPOE alert will calculate the risk that the patient has ABD infection due to MRSA.
      ii. Example 2: When a clinician orders cefepime for SST infection, the CPOE alert will calculate the risk that the patient has SST infection due to *Pseudomonas*.
      iii. Example 3: When a clinician orders linezolid for SST infection, the CPOE alert will calculate the risk that the patient has SST infection due to MRSA or VRE.
      iv. Example 4: When a clinician orders meropenem for ABD infection, the CPOE alert will calculate the risk that the patient has ABD infection due to ESBL or other resistant gram-negative bacteria.
3. **Clinicians will be given recommendations on the appropriateness of extended-spectrum antibiotics based upon the patient's calculated risk.**
   a. If the patient's risk for antibiotic-resistant ABD or SST infection is <10%, then the risk is considered low and the CPOE alert will recommend ceftriaxone (abdominal infection) and cefazolin (skin/soft tissue infections) as the standard empiric antibiotic. Other standard-spectrum antibiotic can be used to treat low risk patients as per physician preferences.

b. If the patient's risk for antibiotic-resistant ABD or SST infection is $\geq$10%, the prompt will automatically allow completion of the ES antibiotic order without any prompts.

c. CPOE recommendations are **not intended to supplant clinician judgment**. Patients who are neutropenic, decompensating, or otherwise at high risk for complications or drug resistant infections should be treated as clinically appropriate.

d. Clinicians will always have the option to override the CPOE alert recommendation after indicating the reason for the override.

4. Prescriber acceptance with CPOE alert recommendations will be monitored by the stewardship team/study champions.

a. Clinician Feedback reports, including the number and reason for CPOE recommendation overrides, will be provided to study champions.

b. Study champions will review acceptance and provide prescribers with written and/or verbal feedback.

Table 1: Extended-Spectrum Antibiotics That Will Trigger INSPIRE CPOE Alerts

Category	Extended-Spectrum Antibiotics for INSPIRE CPOE Alerts[1]
**Anti-MRSA**	Ceftaroline, Daptomycin, Linezolid, Vancomycin[2]
**Anti-VRE**	Daptomycin, Linezolid
**Anti-Pseudomonal**	Aztreonam, Cefepime, Ceftazidime, Piperacillin/Tazobactam
**Anti-ESBL**	Ertapenem, Imipenem, Meropenem, Ceftolozane/Tazobactam

[1]Additional restricted antibiotics will not activate INSPIRE alerts, [2]IV formulation only

Note: INSPIRE smart prompt excludes the following routes of administration: inhaled, otic, ophthalmic, or irrigation.

Abbreviations: MRSA - Methicillin Resistant *Staphylococcus aureus;* **VRE –** Vancomycin Resistant *Enterococci;* **ESBL –** Extended-Spectrum Beta Lactamase producer

INSPIRE Trials: Arm 2

Clinician Education Materials

This section includes:

- INSPIRE Trials One-Page Summary
- Clinician FAQs with Prompt Screenshots
- Clinician FAQs (no Prompt Screenshots)
- Smart Prompt “Top 5” Reminders for Clinicians
- Extended-Spectrum Antibiotics List
- Verbal Order Checklist for Pharmacists and Nurses

Funded by National Institute of Allergy and Infectious Diseases

INSPIRE-ASP Abdominal & Skin/Soft Tissue Trials

INtelligent Stewardship Prompts to Improve Real-time Empiric Antibiotic Selection for Patients

The **INSPIRE-ASP Trials** are a set of two large cluster-randomized trials to improve antibiotic stewardship for non-ICU patients with abdominal (ABD) and skin & soft tissue (SST) infection in HCA Healthcare (HCA) hospitals. Participating hospitals are randomized to:

- **Arm 1 – Routine Care**
- **Arm 2 – Smart Prompt -** Precision medicine smart prompt using a computerized physician order entry (CPOE) alert that gives a ***patient-specific risk estimate of antibiotic resistant infection*** and recommends standard-spectrum antibiotics in patients admitted for ABD or SST infection who are calculated to have low risk (<10%) for antibiotic resistant infection.

Rationale

- Prior INSPIRE Pneumonia & UTI Trials reduced unnecessary extended-spectrum (ES) antibiotics by 30%

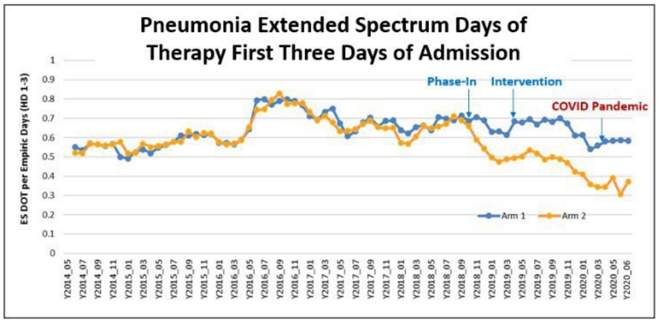

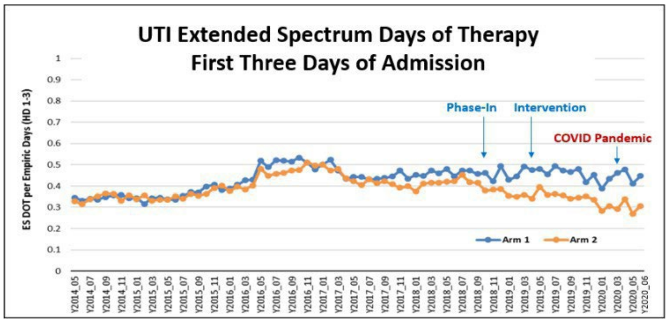

****CONFIDENTIAL for HCA internal use ONLY – Preliminary results, do not cite or distribute****

- Most inpatients with ABD or SST infection do not benefit from ES antibiotics.
- Smart prompt reduces ES use by documenting low risk patients and encouraging standard antibiotics that have fewer adverse effects (e.g. kidney injury, future antibiotic resistance, *C. difficile*)

CPOE Details

- MDRO risk estimates are based on **patient-specific** risk factors and **local** antibiograms.
- Demographics, comorbidities, MDRO history, prior antibiotics or nursing home stays, and multiple lab values from 422,000 HCA patients helped create the prediction algorithm.
- Antibiotic recommendations are based on MDRO risk estimates and approved by the trial steering committee, national experts, and HCA leadership.
- Recommendations support proper antibiotic choice but do not replace clinical judgment.

Outcomes

- Primary: Empiric use of ES antibacterial (first 3 days of admission)
- Secondary: Empiric use of (a) vancomycin and (b) antipseudomonal antibacterial
- Safety: ICU transfer and length-of-stay
- Other Outcomes: *C. difficile* infection, MDRO prevalence, cost-analysis

Timeline

- Phase In: Aug 2022 – Dec 2022 (5 months); Intervention: Jan 2023 – Dec 2023 (12 months)

INSPIRE Abdominal & Skin/Soft Tissue Infection Trials
Clinician FAQs with Prompt Screenshots

What are the INSPIRE-ASP Trials?

The INSPIRE-ASP (**IN**telligent **S**tewardship **P**rompts to **I**mprove **R**eal-time **E**mpiric **A**ntibiotic **S**election for **P**atients) Trials are two cluster-randomized trials of HCA Healthcare hospitals to test an antibiotic stewardship intervention for abdominal (ABD) and skin and soft tissue (SST) infections.

- **Arm 1** – **Routine Care**
- **Arm 2** – **CPOE Smart Prompt Alert Intervention**
  1. Provides patient-specific risk estimate for ABD or SST infections due to a multidrug-resistant organism (MDRO) when extended-spectrum (ES) antibiotics are ordered
  2. Recommends standard antibiotics in non-ICU patients with low MDRO risk

When will the computerized physician order entry (CPOE) smart prompt fire?

- **Prompts fire when ES antibiotics are ordered within 3 days ofadmission**
- Only for **adults** in **ED or non-intensive care units (ICU)** admitted for **ABD or SST infections**
- Risk of antibiotic resistant infection is calculated & recorded using **real-time, patient-specific data**
  - **If ≥ 10%**, **extended-spectrum antibiotic allowed**
  - **If <10%**, **prompt recommends standard antibiotics**

Which antibiotics are considered extended-spectrum?

Category	Extended-Spectrum Antibiotics for INSPIRE CPOE Alerts[1]
**Anti-MRSA**	Ceftaroline, Daptomycin, Linezolid, Vancomycin[2]
**Anti-VRE**	Daptomycin, Linezolid
**Anti-Pseudomonal**	Aztreonam, Cefepime, Ceftazidime, Piperacillin/Tazobactam
**Anti-ESBL**	Ertapenem, Imipenem, Meropenem, Ceftolozane/Tazobactam

[1]Additional restricted antibiotics will not activate INSPIRE alerts, [2]IV formulation only

How is the INSPIRE CPOE prompt "smart"?

The smart prompt uses your patient's risk factors to calculate MDRO infection risk. For example:

- If **ceftriaxone** ordered → **no prompt** fires at all (standard-spectrum antibiotic)
- If **vancomycin** ordered → **returns patient's MRSA risk**
- If cefepime or **piperacillin/tazobactam** ordered → returns ***Pseudomonas* risk**
- If **linezolid** ordered → returns **MRSA and VRE risk**
- If **meropenem** ordered → returns **ESBL + resistant gram-negative bacteria**

What does the INSPIRE CPOE prompt account for at the moment of order entry?

The prompt can only account for information available in your hospital's Meditech system at the time of order entry. **The prompt can capture**:

- **History of MDRO** from microbiology data as far back as available in Meditech through 2012
- **Cultures resulted in Meditech at your facility 3 or more days ago**

The prompt cannot capture:

- MDRO history or cultures from an **outside hospital**
- **Culture results < 3 days** from admission
  - Monitor all new cultures as they become available and adjust accordingly

What are the INSPIRE CPOE risk estimates based on?

We assessed over 422,000 patients admitted to HCA Healthcare hospitals over 3 years. We evaluated **(1) your hospital antibiogram** for ABD or SST infection patients and **(2) patient risk factors of MDRO infection**, including demographics (age, gender, race, ethnicity, insurance status), comorbid conditions, history of MDRO, prior surgery, prior HCA hospitalization, ED, or nursing home admission, prior antibiotics, and admission lab values. Using a special statistical method, we identified which risk factors were most often found in patients with ABD or SST MDRO infection. Using these risk factors (which are different for every MDRO), the CPOE smart prompt gives the patient's **individual absolute risk** for infection with the MDRO(s) targeted by the antibiotic ordered.

How does the INSPIRE CPOE prompt exclude ICU patients?

1. Does not fire if a patient is located in the ICU.
2. For emergency department, smart prompt asks clinicians if the patient is ICU-bound and does not fire if clinician responds "yes".
3. If smart prompt fires and patient is going to the ICU, clinicians can override theprompt.

Will INSPIRE impact ED sepsis core measures?

The INSPIRE prompt **does NOT** affect "time to antibiotics" because it does not impact whether antibiotics should be used but rather gives real-time information to help decide whether extended-spectrum or standard-spectrum antibiotics should be used. The standard-spectrum antibiotics in the INSPIRE prompts are fully compliant with core measures.

What does the INSPIRE smart prompt intervention look like?

In the below example smart prompt screen, a clinician ordered Vancomycin for abdominal infection. Patient risk for MRSA abdominal infection is calculated to be <10%.

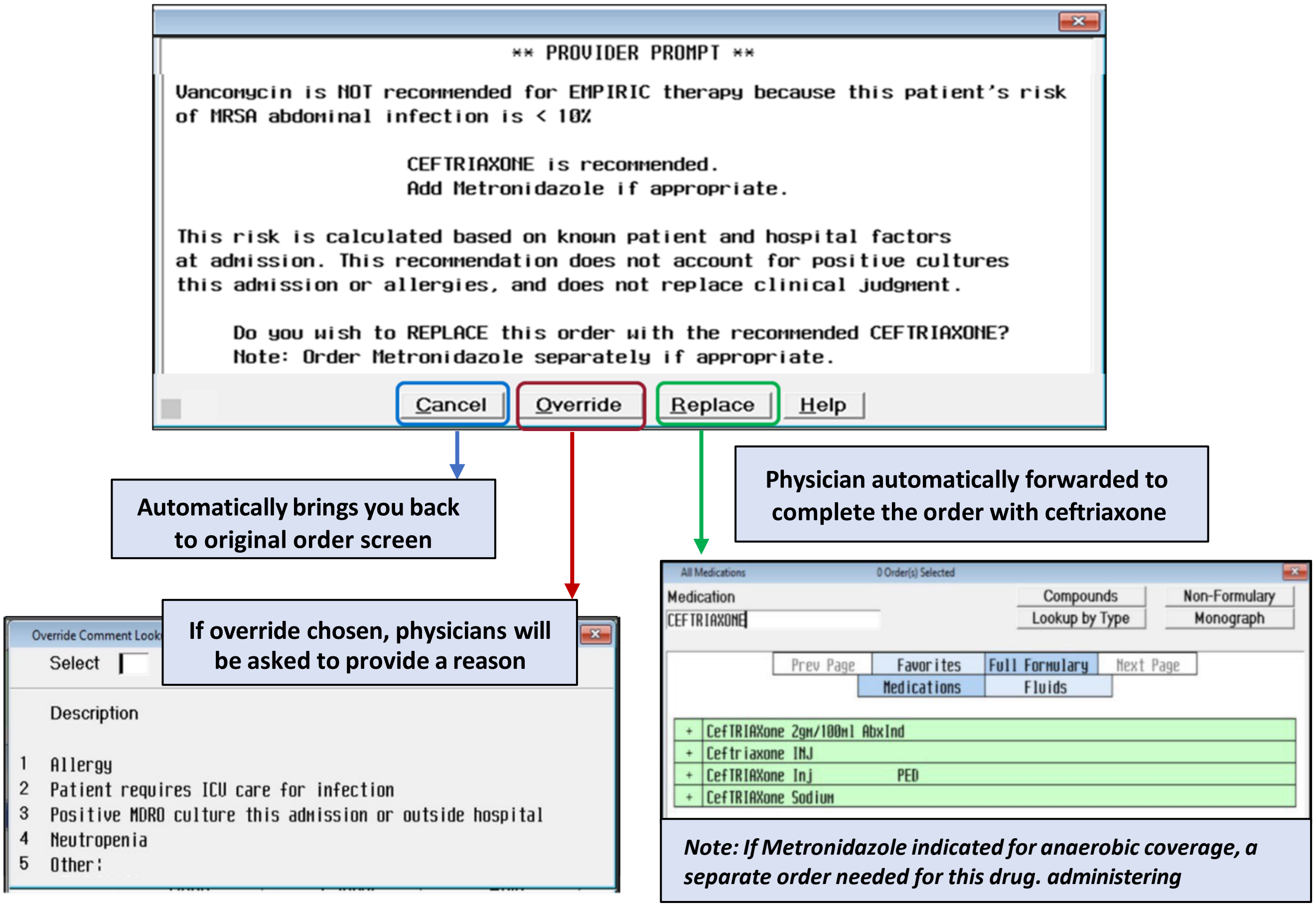

INSPIRE Abdominal and Skin & Soft Tissue Infection Trials
Clinician FAQs

What are the INSPIRE-ASP Trials?

The INSPIRE-ASP (**IN**telligent **S**tewardship **P**rompts to **I**mprove **R**eal-time **E**mpiric **A**ntibiotic **S**election for **P**atients) Trials are two separate cluster-randomized trials of HCA Healthcare hospitals to test an antibiotic stewardship intervention abdominal (ABD) and skin and soft tissue (SST) infections.

- **Arm 1 – Routine Care**
- **Arm 2 – CPOE Smart Prompt Alert Intervention**
    1. Provides patient-specific risk estimate for ABD or SST infections due to a multidrug-resistant organism (MDRO) when extended-spectrum (ES) antibiotics are ordered
    2. Recommends standard antibiotics in non-ICU patients with low (<10%) MDRO risk

When will the computerized physician order entry (CPOE) smart prompt fire?

- **Prompts fire when ES antibiotics are ordered within 3 days ofadmission**
- Only for **adults** in **ED or non-intensive care units (ICU)** admitted for **ABD or SST infections**
- Risk of antibiotic resistant infection is calculated & recorded using **real-time, patient-specific data**
    - **If ≥ 10%**, **extended-spectrum antibiotic allowed**
    - **If <10%**, **prompt recommends standard antibiotics**

Which antibiotics are considered extended-spectrum?

Category	Extended-Spectrum Antibiotics for INSPIRE CPOE Alerts[1]
**Anti-MRSA**	Ceftaroline, Daptomycin, Linezolid, Vancomycin[2]
**Anti-VRE**	Daptomycin, Linezolid
**Anti-Pseudomonal**	Aztreonam, Cefepime, Ceftazidime, Piperacillin/Tazobactam
**Anti-ESBL**	Ertapenem, Imipenem, Meropenem, Ceftolozane/Tazobactam

[1]Additional restricted antibiotics will not activate INSPIRE alerts, [2]IV formulation only

How is the INSPIRE CPOE prompt "smart"?

The CPOE smart prompt uses your patient's risk factors (from medical record data) to calculate MDRO risk for abdominal or skin/soft tissue infection. Risk estimates are tailored to the antibiotic selected. Examples:

- If **ceftriaxone** ordered → **no prompt** fires at all (standard-spectrum antibiotic)
- If **vancomycin** ordered → **returns patient's MRSA risk**
- If cefepime or **piperacillin/tazobactam** ordered → returns ***Pseudomonas* risk**
- If **linezolid** ordered → returns **MRSA and VRE risk**
- If **meropenem** ordered → returns **ESBL + resistant gram-negative bacteria**

How does the INSPIRE CPOE prompt exclude ICU patients?

1. Does not fire if a patient is located in the ICU.
2. For emergency department, smart prompt asks clinicians if the patient is ICU-bound and does not fire if clinician responds "yes".

3. If smart prompt fires and patient is going to the ICU, clinicians can override theprompt.

What are the INSPIRE CPOE risk estimates based on?

The INSPIRE study team assessed over 422,000 patients admitted to HCA Healthcare hospitals over 3 years. We evaluated **(1) your hospital antibiogram** for ABD or SST infection patients and **(2) patient risk factors of MDRO infection**, including demographics (age, gender, race, ethnicity, insurance status), comorbid conditions, history of MDRO, history of prior surgery, history of prior HCA hospital, ED, or nursing home admission, prior antibiotics, and admission lab values.

Using a special statistical method, we identified which risk factors were most often found in patients who developed ABD or SST infection with an MDRO. Using these risk factors (which are different for every MDRO), the CPOE smart prompt gives the patient's **individual absolute risk** for infection with the MDRO(s) targeted by the antibiotic ordered.

What does the INSPIRE CPOE prompt account for at the moment of order entry?

The prompt can only account for information available in your hospital's Meditech system at the time of order entry. **The prompt can capture**:

- **History of MDRO** from microbiology data as far back as available in Meditech through 2012
- **Cultures resulted in Meditech at your facility 3 or more days ago**

The prompt cannot capture:

- MDRO history or cultures from an **outside hospital**
- **Culture results < 3 days** from admission
    - Monitor all new cultures as they become available and adjust accordingly

Will INSPIRE impact ED sepsis core measures?

The INSPIRE prompt **does NOT** affect "time to antibiotics" because it does not impact whether antibiotics should be used but rather gives real-time information to help decide whether extended-spectrum or standard-spectrum antibiotics should be used. The standard-spectrum antibiotics in the INSPIRE prompts are fully compliant with core measures.

Will patients need to give informed consent?

No. This study has been approved as a minimal risk study and will be implemented as a quality improvement (QI) protocol through the existing infrastructure of your hospital's antibiotic stewardship programs. The assigned protocol will be approved through your usual clinical/leadership committees and become hospital guidance on appropriate empiric antibioticuse.

Who can I contact for more information?

INSPIRE-ASP Study Team:
HCAINSPIRE@gmail.com
(844) 522-4677

Funded by National Institute of Allergy and Infectious Diseases

INSPIRE Abdominal & Skin/Soft Tissue Trials

Smart Prompt "Top 5" Reminders for Clinicians

Top 5 Things Providers Should Know About the INSPIRE Prompts

1. INSPIRE **smart prompts calculate your patient's risk** for antibiotic resistant infection using real-time, patient-specific data
   - **If ≥ 10%, extended-spectrum (ES) antibiotic allowed**
   - **If <10%, prompts for standard antibiotics**
2. Risk estimates are **recorded in Meditech and give documentation** to justify use of standard-spectrum antibiotics when risk is low enough to await cultures
3. Prompt **cannot account for information that is not in Meditech**:
   - History of drug resistant bacteria or culture results from an outside hospital
   - Culture results finalized in the past 3 days in your hospital
   - Neutropenia
   - Allergy
4. **You will get feedback on antibiotic use and prompt acceptance** to help you minimize ES antibiotic use
5. The INSPIRE Pneumonia and UTI trials **achieved up to 30% reduction in ES use, without prolonging length-of-stay and without increasing ICU transfers**

Please contact your Antibiotic Stewardship Team for questions or details

Study Investigators & Coordinators

(844) 522-4677

HCAINSPIRE@gmail.com

Funded by National Institute of Allergy and Infectious Diseases

Extended-Spectrum Antibiotics List

Which Antibiotics Are Considered Extended-Spectrum?

Antibiotics with activity against extensive types of bacteria, including multidrug-resistant organisms (MDROs), are considered "extended-spectrum" (ES) antibiotics. Choosing ES antibiotics increases a patient's risk of *C. difficile*, future MDROs, and adverse drug effects (e.g., renal or liver insufficiency). The goal of the INSPIRE Abdominal and Skin/Soft Tissue Trials is to decrease ES antibiotic use and improve patient safety.

The antibiotics in **Table 1** are considered ES antibiotics in the INSPIRE trials and will activate the INSPIRE computerized physician order entry prompt (CPOE) when ordered for abdominal or skin/soft tissue infections in the emergency department or non-intensive care units within 3 days of admission.

The INSPIRE prompt does not trigger for restricted antibiotics that require prior approval or are non-formulary.

Table 1: Extended-Spectrum Antibiotics That Will Trigger INSPIRE CPOE Alerts

Category	Extended-Spectrum Antibiotics for INSPIRE CPOE Alerts[1]
**Anti-MRSA**	Ceftaroline, Daptomycin, Linezolid, Vancomycin[2]
**Anti-VRE**	Daptomycin, Linezolid
**Anti-Pseudomonal**	Aztreonam, Cefepime, Ceftazidime, Piperacillin/Tazobactam
**Anti-ESBL**	Ertapenem, Imipenem, Meropenem, Ceftolozane/Tazobactam

[1]Additional restricted antibiotics will not activate INSPIRE alerts, [2]IV formulation only

Funded by National Institute of Allergy and Infectious Diseases

Verbal Order Checklist for Pharmacists and Nurses

Background

The INSPIRE CPOE smart prompt intervention encourages compliance with national guidance for standard-spectrum antibiotics (e.g., ceftriaxone for abdominal (ABD) and cefazolin for skin/soft tissue (SST) infections). Ordering extended-spectrum (ES) antibiotics (e.g., vancomycin, cefepime) for ABD or SST infections in the Emergency Department (ED) or non-Intensive Care Unit (ICU) wards within 3 days of admission will activate a computerized physician order entry (CPOE) smart prompt alert that will use data available in the patient's electronic medical record to provide:

1. Patient's individual risk for antibiotic resistant infection based on the patient's own data in your hospital's electronic medical record
2. Recommendation for standard-spectrum antibiotics when risk is low (<10%)

Use CPOE Meditech Modules When Placing Verbal Orders

The INSPIRE prompt fires in the following CPOE modules within Meditech: "OM" (Order Management), "EDM" (Emergency Department Management), and PWM (Provider/Physician Workflow Management). **It will NOT fire in the "PHA" (Pharmacy) module.** We ask all staff taking verbal orders (including "vancomycin per pharmacy dosing" orders) to place them using OM, EDM, or PWM modules. Staff taking verbal orders will also need pertinent information from physicians to be able to respond to the INSPIRE prompt, such as (1) what infection the antibiotic is meant to treat and (2) whether the physician would agree to standard-spectrum antibiotics if the risk is found to be low (<10%).

Taking Verbal Orders for Inpatient Extended-Spectrum Antibiotics

The INSPIRE CPOE prompts apply **ONLY to ES antibiotics** ordered on **non-ICU patients** hospitalized for **ABD or SST infection** within **3 days of admission**.

- ➢ The prompt does **NOT** fire for standard-spectrum antibiotics.
- ➢ A list of ES antibiotics is provided in Table 1 below.

When taking verbal orders for ES antibiotics, we recommend that you stay on the phone with the ordering clinician while entering orders. If you are unable to place the order when taking the verbal order, use the following questions to get the information you will need prior to placing the order:

1. **Check if patient has been hospitalized for 4 or more days OR is in the ICU? If yes to either →** *no further physician questions needed. CPOE will **NOT** fire.*

2. **Is patient in the Emergency Department?**
   a. **If in ED →** ask if patient is ICU-bound
      i. **If ICU-bound →** no further questions
      ii. **If NOT ICU-bound → proceed to Step 3**

3. **IF ES antibiotic being ordered within 3 days of admission:**
   a. **Ask prescriber what the indication is for the antibiotic.** Remind prescriber that indications are required for all antibiotic orders as part of HCA Healthcare's meaningful use stewardship initiative.
   b. If prescriber chooses any of the following indications, **proceed to step 4**:
      - Abdominal infections
      - Skin/soft tissue infections
      - Sepsis – due to ABD or SST infections
      - Empiric (unknown) source – possibly from an abdominal or skin/soft tissue source

4. **Remind prescriber that ES antibiotics in this patient will trigger the INSPIRE smart prompt and will give the patient's specific risk for infection due to multidrug-resistant organism (MDRO) or *Pseudomonas*. The following suggested script can be used or modified:**
   a. "Just a reminder, Dr., our hospital has an antibiotic selection protocol for abdominal and skin/soft tissue infections. The computer will calculate and document your patient's risk of an antibiotic-resistant ABD/SST infection that justifies an ES antibiotic. I will need to ask you a couple of questions so I can place the order."
   b. If ordering anti-MRSA or antipseudomonal for abdominal infection then: "Has the patient had abdominal surgery in the past year? This may affect the risk estimate calculation."
   c. "If the risk is low (<10%) then [ceftriaxone for abdominal infection; cefazolin for skin/soft tissue infection] will be recommended. Should I plan to follow this recommendation?"
      i. **If yes** → no further questions.
      ii. **If no** → ask the prescriber the reason for override:
         - Allergy
         - Neutropenia
         - Positive MDRO culture this admission or outside hospital
         - Patient expected to go to the ICU
         - Other (ask prescriber to specify)

Table 1: Extended-Spectrum Antibiotics That Will Trigger INSPIRE CPOE Alerts

Category	Extended-Spectrum Antibiotics for INSPIRE CPOE Alerts[1]
**Anti-MRSA**	Ceftaroline, Daptomycin, Linezolid, Vancomycin[2]
**Anti-VRE**	Daptomycin, Linezolid
**Anti-Pseudomonal**	Aztreonam, Cefepime, Ceftazidime, Piperacillin/Tazobactam
**Anti-ESBL**	Ertapenem, Imipenem, Meropenem, Ceftolozane/Tazobactam

[1]Additional restricted antibiotics will not activate INSPIRE alerts, [2]IV formulation only

INSPIRE Trials: Arm 2

Clinician Hot Topics

This section includes:

- How MDRO Risk Estimates Are Calculated
- Safety of Choosing Standard-Spectrum Empirically
- Clinician Feedback on Antibiotic Use

INSPIRE Abdominal and Skin & Soft Tissue Infection Trials
Hot Topic: How MDRO Risk Estimates Are Calculated

What are the INSPIRE Trials?

The INSPIRE (**IN**telligent **S**tewardship **P**rompts to **I**mprov**e** **R**eal-time **E**mpiric Antibiotic Selection for Patients) Trials are two separate cluster-randomized trials of HCA Healthcare hospitals to test an antibiotic stewardship intervention for abdominal (ABD) and skin/soft tissue (SST) infections.

- **Arm 1 – Routine Care**
- **Arm 2 – CPOE Smart Prompt Alert Intervention**
  1. Provides patient-specific risk estimate for ABD or SST infections due to a multidrug-resistant organism (MDRO) when extended-spectrum (ES) antibiotics are ordered
  2. Recommends standard antibiotics in non-ICU patients with low (<10%) MDRO risk

When will the INSPIRE prompt fire?

- **Prompts fire when ES antibiotics are ordered within 3 days ofadmission**
- For **adults** in **ED or non-intensive care units (ICUs)** admitted for **ABD or SST infections**
- Risk of MDRO infection is calculated & recorded using **real-time, patient-specific data**
  - **If ≥ 10%**, **extended-spectrum antibiotic allowed**
  - **If <10%**, **prompt recommends standard antibiotics**

What are the INSPIRE risk estimates based on?

The INSPIRE study team assessed over 422,000 patients admitted to HCA Healthcare hospitals over 3 years. We evaluated **(1) the hospital antibiogram** for ABD or SST infection patients and **(2) patient risk factors of MDRO infection**, including demographics (age, gender, race, ethnicity, insurance status), comorbid conditions, history of MDRO, history of prior surgery, history of prior HCA hospital, emergency room, or nursing home admission, prior antibiotics, and admission lab values.

What statistical method was used to calculate the INSPIRE risk estimates?

The INSPIRE prompt gives the **absolute risk** for a patient to have an abdominal or skin/soft tissue infection due to an MDRO. To estimate absolute risk, we used a statistical method called recursive partitioning. This approach evaluates the study population by each patient risk factor repeatedly until it identifies the set of risk factors most commonly found in patients with ABD or SST infection due to an MDRO. This analysis was done separately for each MDRO and for each infection so that **risk estimates are specific to (1) your patient, (2) the MDRO targeted by the antibiotic ordered, and (3) the infection type**.

Why is it important that the INSPIRE prompt gives absolute risk?

Clinicians currently select antibiotics according to their perception of MDRO risk. This is often based on relative risks or odds ratios published in studies that single out selected patient risk factors. For example, if a patient has diabetes or is coming from a nursing home, we worry that they have higher risk for MDRO infection because of studies that show that such patients have an odds ratio of 2 or 3 for MDRO infection

compared to the general population. However **relative risks do not tell us anything about a patient's actual risk** for MDRO infection. To illustrate the problem with selecting antibiotics using relative risk, consider the following hypothetical scenario:

- Let's say a study reports females have a risk of 1 in 1,000 for ESBL abdominal infection and males have a risk of 3 in 1,000.
- That would be calculated as an odds ratio of 3 and interpreted by clinicians as 3-fold higher risk
- Yet, the absolute risk is actually only 0.3%

The INSPIRE trials use absolute risk because this is a more accurate assessment of individual risk.

How is the INSPIRE CPOE prompt "smart"?

The INSPIRE prompt uses your patient's risk factors (from medical record data) to calculate MDRO risk for abdominal or skin/soft tissue infection. Risk estimates are tailored to the antibiotic selected. Example:

- If **ceftriaxone** ordered → **no prompt** fires at all (standard-spectrum antibiotic)
- If **vancomycin** ordered → **returns patient's MRSA risk**
- If cefepime or **piperacillin/tazobactam** ordered → returns ***Pseudomonas* risk**
- If **linezolid** ordered → returns **MRSA and VRE risk**
- If **meropenem** ordered → returns **ESBL + resistant gram-negative bacteria**

What does the INSPIRE CPOE prompt account for at the moment of order entry?

The prompt can only account for information available in your hospital's Meditech system at the time of order entry. **The prompt can capture**:

- **History of MDRO** from microbiology data as far back as available in Meditech through 2012
- **Cultures resulted in Meditech at your facility 3 or more days ago**

The prompt cannot capture:

- MDRO history or cultures from an **outside hospital**
- **Culture results < 3 days** from admission
  - Monitor all new cultures as they become available and adjust accordingly

Will risk estimates be validated after the intervention starts?

The INSPIRE risk estimates models are robust and based on 3 years of data across the HCA Healthcare hospital enterprise. Estimates for a single facility are unlikely to change significantly within 1 year. Our evaluation also shows that facility MDRO prevalence appears stable over the short-term. Risk estimates will be re-validated periodically as needed.

Who can I contact for more information?

INSPIRE Research Coordinator – Victoria Ngo, UC Irvine - HCAINSPIRE@gmail.com, 949-824-7806

Lead Investigator – Shruti Gohil, MD, MPH, UC Irvine - skgohil@hs.uci.edu

Co-Investigator – Ed Septimus, MD, Senior Lecturer HPHCI - eseptimus@gmail.com

Manager, Clinical Pharmacy Services – Kaleb Roemer, PharmD - Kaleb.Roemer@hcahealthcare.com

Director, Clinical Pharmacy Services – Chenette Burks, PharmD - Ruby.Burks@hcahealthcare.com

Funded by National Institute of Allergy and Infectious Diseases

INSPIRE Abdominal and Skin & Soft Tissue Infection Trials

Hot Topic: Clinician Feedback on Antibiotic Use

Why is my antibiotic use being monitored and why is feedback important?

Extended-spectrum (ES) antibiotic overuse is a major patient safety concern because it puts patients at high risk for renal insufficiency, *C. difficile* colitis, future development of multidrug-resistant organisms, and adverse drug effects. Studies show that antibiotic stewardship interventions that provide clinicians with direct feedback on their antibiotic choices can reduce overuse by as much as 40%.

How will feedback be provided?

Your antibiotic stewardship team will generate antibiotic use reports at the individual physician and specialty level. They will also generate reports on how often you choose to take the INSPIRE prompt recommendation in low risk (<10%) patients, how often you override, and what your reasons for override were. They will share these data with leadership and with you to address any questions or concerns you may have. Below are examples of feedback reports that may be shared with you:

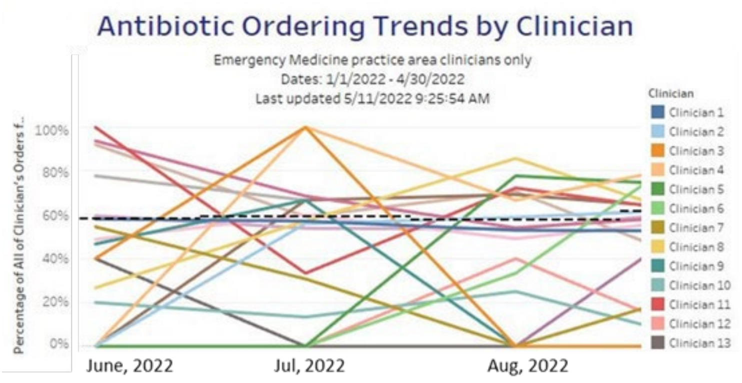

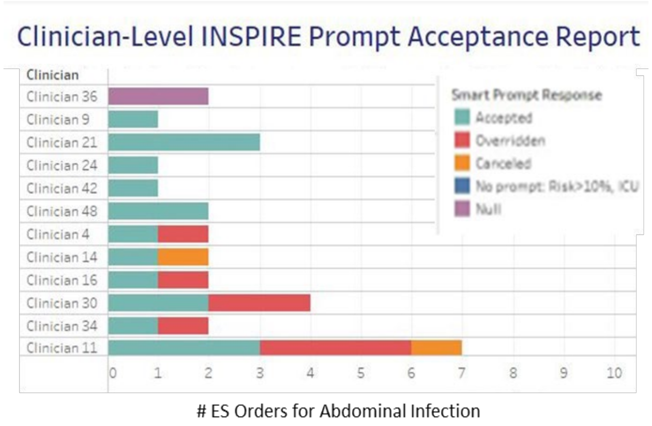

What is the goal of feedback and what response is expected of me?

The goal of feedback is to reduce ES antibiotic use at your facility and help target antibiotic stewardship education. You are fully expected to select antibiotics according to your best clinical judgment at all times. Feedback on antibiotic use is expected to facilitate meaningful and collegial conversations between you and your antibiotic stewardship team.

Funded by National Institute of Allergy and Infectious Diseases

INSPIRE Abdominal and Skin & Soft Tissue Infection Trials

Hot Topic: Safety of Choosing Standard-Spectrum Empirically

MDRO rates are rising, doesn't that mean my patient's risk for MDRO is high?

Multidrug-resistant organism (MDRO) rates are rising due to overuse of extended-spectrum (ES) antibiotics. While MDRO rates may rise at the population level, this does not directly translate to an increased risk of MDRO infection at the individual level. In fact, the **vast majority of patients do not have infection from an MDRO**.

We assessed over 422,000 HCA Healthcare patients over 3 years and found that <1% of patients with abdominal (ABD) infection grew MDROs. Similarly, <4% of patients with skin/soft tissue (SST) infection were infected with *Pseudomonas* and 12% grew MRSA. Yet, ES antibiotic use was overwhelmingly higher than needed. The vast majority could have been safely treated with standard-spectrum.

Antibiotic Use & MDRO Prevalence – All HCA Facilities

ABD Infection Antibiotic Use	
Extended-Spectrum	62.6%
Vancomycin	24.4%
Antipseudomonal	53.3%
**SST Infection Antibiotic Use**	
Extended-Spectrum	89.4%
Vancomycin	80.5%
Antipseudomonal	60.8%

HCA baseline data 2017-2019

→

MDRO ABD Infection	
MRSA	0.9%
Pseudomonas	0.8%

MDRO SST Infection	
MRSA	12.1%
Pseudomonas	3.7%

Is it safe to choose empiric standard-spectrum antibiotics?

Yes. Studies have shown that non-critically ill patients who are started on ES antibiotics have similar length of stay, mortality, and clinical cure rates when compared with those started on standard-spectrum antibiotics. The INSPIRE Pneumonia and UTI Trials successfully reduced empiric ES use by up to 30% without impact on length-of-stay, ICU transfers, or need for antibiotic escalation.

Why not start with ES and de-escalate after cultures are back?

Every dose and day of ES antibiotics can put your patient at higher risk for renal insufficiency, *C. difficile* colitis, future development of MDRO, adverse drug effects and healthcare costs. Emerging data show that as little as a few doses of antibiotics can alter a patient's gut flora, which has been linked to increased risk of numerous chronic, metabolic, oncologic, and inflammatory diseases.

Data indicate that 80% of antibiotics originally started in the emergency department are continued throughout the admission. So, starting with standard-spectrum antibiotics up front can significantly reduce your patient's risk of negative effects of ES antibiotics.

Funded by National Institute of Allergy and Infectious Diseases

INSPIRE Abdominal & Skin/Soft Tissue Infection Trials

INtelligent Stewardship Prompts to Improve Real-time Empiric Antibiotic Selection for Patients

Clinician Education

Context & Rationale

Overuse of Antibiotics: A Major National Priority

- Extended-spectrum (ES) antibiotics increase resistance[1]
- 40% inpatients receive inappropriate antibiotics[2]
- Adversely affects patient safety metrics – *C. difficile*, MRSA bacteremia
- National and state mandates for stewardship programs
- Gaps in empiric stewardship
  - National efforts focus on de-escalation not empiric choice
  - Dedicated strategies needed for empiric selection

[1]Paterson, D et al. Clin Infect Dis 2014;33:126-128

[2]Fridkin, S et al. MMWR 2014;63(9):194-200

Selecting Empiric Antibiotics: There is Time to Broaden

- Evidence in non-ICUs - no harm in waiting for cultures before ES antibiotics used
- Similar outcomes with empiric standard vs ES antibiotics[1]
- Waiting for culture results to start ES antibiotics in those who eventually grow an MDRO does not adversely impact mortality[1,2]

[1]Rhedin, S., et al. Clinical Microbiology and Infection, 23.4 (2017): 247-252.
[2]Lin, Michael Y., Antimicrobial agents and chemotherapy 52.9 (2008): 3188-3194.

Rationale for INSPIRE-ASP Trial

- Uncertainty about possible MDRO drives overuse of ES antibiotics
- Efforts needed to improve empiric prescribing
- Prescribers can choose wisely if
  - Useful, actionable information provided at time of order entry
  - Information individualized to their patient
  - Information documented to support their prescribing choice
  - Feedback on compliance routinely given

INSPIRE
BETTER SAFER ANTIBIOTIC USE

INSPIRE Pneumonia & UTI Trials
Success Reducing Empiric ES Antibiotics

INSPIRE: Pneumonia Trial Primary Outcome

- **28% absolute reduction** in ES-Days of Therapy in Arm 2
- Benefit persisted despite distractions of COVID-19
- **No increase in ICU transfer, LOS, antibiotic escalations**

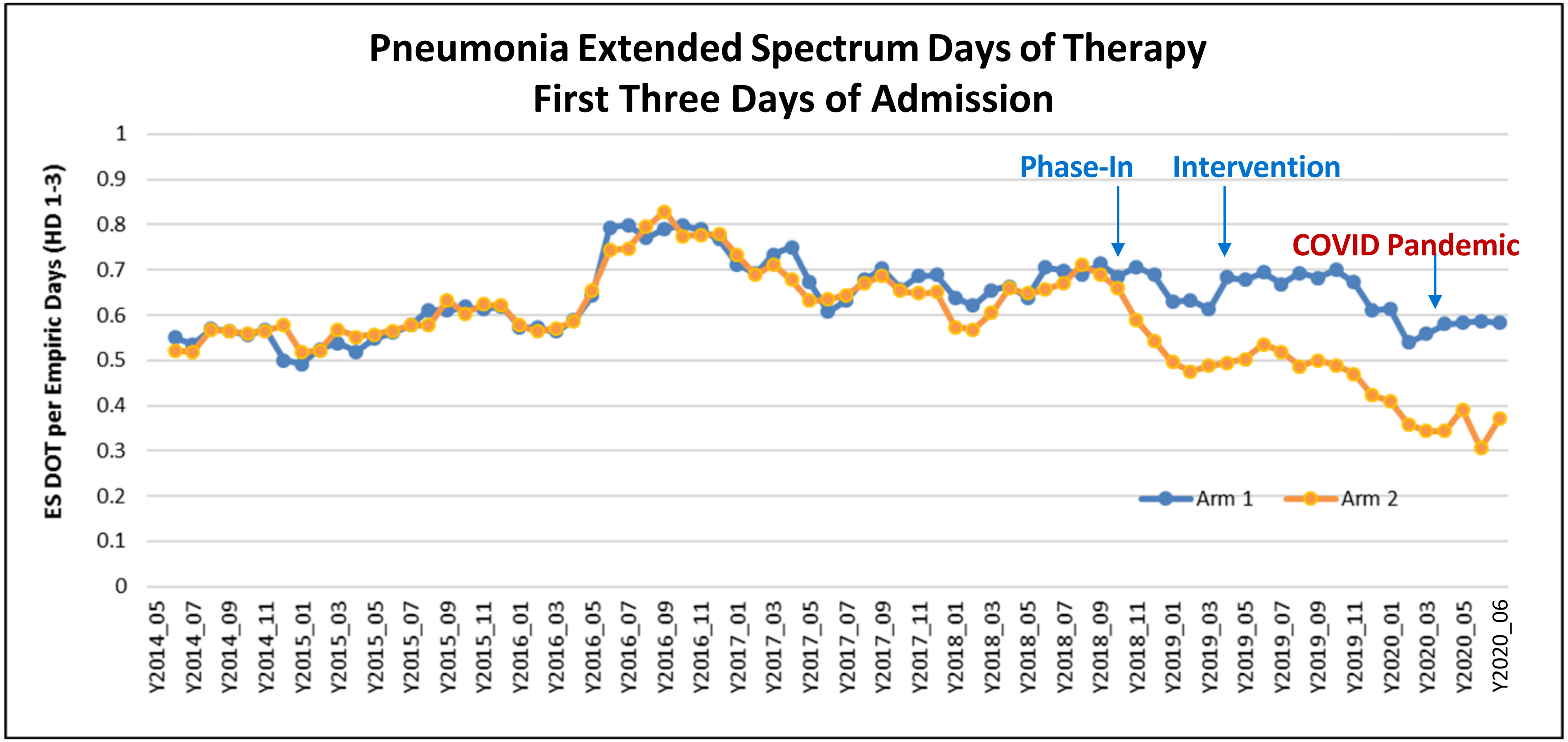

CONFIDENTIAL - DO NOT DISTRIBUTE

INSPIRE: UTI Trial Primary Outcome

- **17% absolute reduction** in ES-Days of Therapy in Arm 2
- Benefit persisted despite distractions of COVID-19
- No increased ICU transfer, LOS, antibiotic escalations

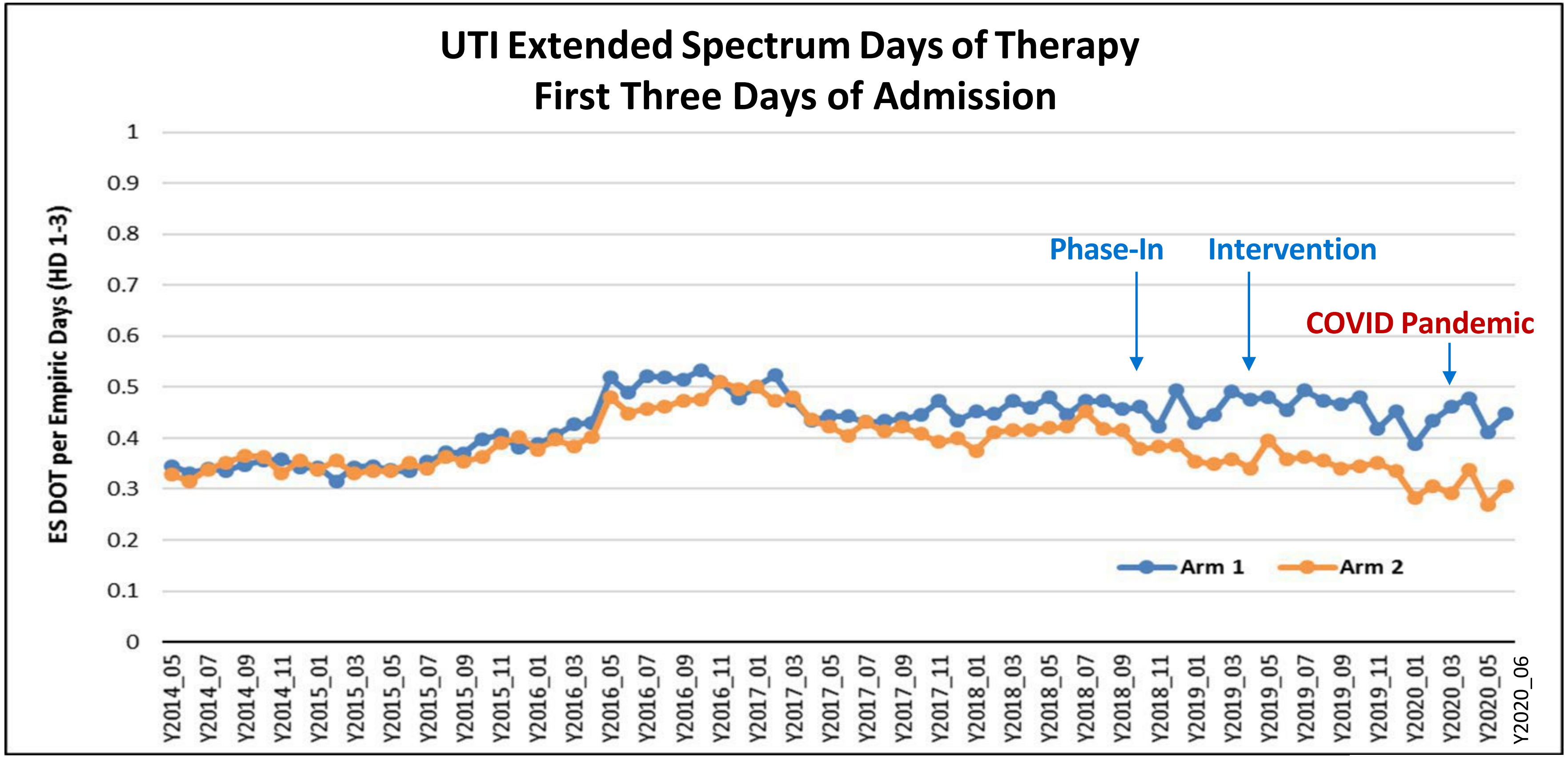

***CONFIDENTIAL* - DO NOT DISTRIBUTE**

INSPIRE Pneumonia and UTI Trials

Major Achievements

- INSPIRE Prompt yielded rapid durable response when coupled with provider acceptance and feedback
- Benefit persisted during distractions of COVID
- Net $1.7 million saved in 15 months across 29 hospitals
- INSPIRE Pneumonia & UTI smart prompts rolled out HCA-wide Summer 2021

Suggests value in extension to other diseases

INSPIRE Abdominal (ABD) & Skin/Soft Tissue (SST) Infection Trials

Welcome to Arm 2

Intervention

Precision Medicine Smart Prompts for Patient-specific
Empiric Antibiotic Selection for Abdominal and Skin/Soft Tissue Infections

What Antibiotics Are Considered "Extended-Spectrum"?

- INSPIRE smart prompt will not trigger for restricted antibiotics
  - E.g., requiring prior approval or non-formulary

Category	ES Antibiotics Included in CPOE Alerts
**Anti-MRSA**	Ceftaroline, Daptomycin, Linezolid[1], Vancomycin[2]
**Anti-VRE**	Daptomycin, Linezolid[1]
**Anti-Pseudomonas**	Aztreonam, Cefepime, Ceftazidime, Piperacillin/Tazobactam
**Anti-ESBL**	Ertapenem, Imipenem, Meropenem, Ceftolozane/Tazobactam

[1]Both oral and intravenous (IV) formulations

[2]IV formulation only

The Gap Between MDRO Prevalence & ES Antibiotic Use

- ES antibiotic use far exceeds MDRO prevalence!
- Among >422,000 HCA Healthcare patients, 2017-2019
  - **<2% of ABD and <4% of SST patients grew MDROs**
  - MRSA SST prevalence higher but ES prescribing 8-fold higher than needed
- Vast majority could have received standard-spectrum

Antibiotic Use & MDRO Prevalence –All HCA Facilities

ABD Infection Antibiotic Use	
Extended-Spectrum	62.6%
Vancomycin	24.4%
Antipseudomonal	53.3%
**SST Infection Antibiotic Use**	
Extended-Spectrum	89.4%
Vancomycin	80.5%
Antipseudomonal	60.8%

HCA baseline data 2017-2019

MDRO ABD Infection	
MRSA	0.9%
Pseudomonas	0.8%

MDRO SST Infection	
MRSA	12.1%
Pseudomonas	3.7%

INSPIRE ABD and SST Trials - Design

Set of two 92-hospital, cluster-randomized trials

Arm 1: Routine Care – 46 Hospitals

- Standard-of-care antibiotic stewardship

Arm 2: INSPIRE Smart Prompt Intervention – 46 Hospitals

- Physician "smart prompts" for patient-specific MDRO risk
- Recommends appropriate antibiotic choice
- Provider encouragement and feedback reports

INSPIRE Study Population and Timeline

Includes

- Adults admitted for **Abdominal or Skin/Soft Tissue Infections**
- Excludes patients admitted to ICUs
- Focuses on first 3 days of admission

Trial Timeline

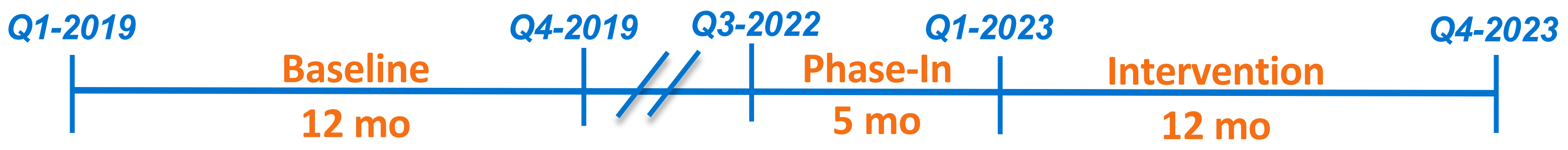

INSPIRE Abdominal & Skin/Soft Tissue Trial Outcomes

Primary Outcome

- ES antibiotic days of therapy (ES-DOT) first 3 days of admission

Secondary Outcomes

- Vancomycin DOT first 3 days of admission **(ABD only)**
- Antipseudomonal DOT first 3 days of admission

Safety Outcomes

- ICU transfers
- Length-of-stay

Other Outcomes (Secondary Manuscripts)

- Incidence of *C. difficile,* MDROs, and other adverse effects
- Cost analysis

INSPIRE MDRO Risk Estimates

How Are They Calculated?

The Problem with Relative Risk and Odds Ratios (OR)

- Clinicians are taught about relative risk and ORs
- OR for MDRO infection often 2 or 3 for nursing home residence or diabetes
- However, this tells us nothing about the absolute risk
- Example
  - Let’s say females have a risk of 1 in 1,000 for ESBL abdominal infection
  - If males have a risk of 3 in 1,000, that would be OR = 3 for an absolute risk of 0.3%
  - Better to know absolute risk
  - **The INSPIRE trials use absolute risk**

Models Assessing Absolute Risk

- **We assume ES antibiotics are prescribed for the MDROs they treat**
  - ✓ Models exist for every ES antibiotic–MDRO pair, for example
    - Vancomycin -- MRSA
    - Linezolid ordered -- MRSA or VRE
    - Cefepime -- *Pseudomonas*
    - Piperacillin/Tazobactam -- *Pseudomonas*
    - Meropenem -- ESBL or *Pseudomonas*
  - ✓ Goals: find best set of factors that predict risk $\geq$10% → allow ES antibiotic
  - ✓ Patients with risk <10% → standard spectrum antibiotic can be recommended

Which Variables Assessed for Every ES Antibiotic-MDRO Model?

- 195,000 SST and 227,000 ABD patients admitted to HCA hospitals, 2017-2019
- We evaluated:

  (1) Hospital antibiogram for ABD/SST infection patients

  (2) Risk factors of MDRO infection
  - Demographics
  - Comorbidities
  - MDRO or *Pseudomonas* history
  - Recent admission, ED visit, nursing home care
  - Abdominal surgery within 1 year
  - Recent antibiotic use
  - Labs (e.g., BUN, creatinine)

Risk Model – *Pseudomonas* ABD Infection

- If absolute risk for *Pseudomonas* infection ≥10%, ES order proceeds w/o prompts
- Among risk factors, only history of *Pseudomonas* and abdominal surgery mattered

Rules	N	Proportion Predicted with *Pseudomonas* ABD Infection
Pseudomonas Hx<br>History of abdominal surgery within 1 year	574	10%
Pseudomonas Hx<br>No abdominal surgery within 1 year	546	6%
All others not meeting above	180977	<=5%

HCA baseline data 2017-2019

INSPIRE Smart Prompt

INSPIRE BETTER SAFER ANTIBIOTIC USE

Risk Model - Pseudomonas SST Infection

- Only predictive risk factor for SST infection: history of prior *Pseudomonas*
- No other traditional risk factor e.g., diabetes, nursing home residence, labs mattered

Rules	N	Proportion Predicted with *Pseudomonas* SST Infection
Pseudomonas Hx	6776	15%
All others not meeting above	141953	4%

HCA baseline data 2017-2019

INSPIRE Smart Prompt

INSPIRE CPOE Smart Prompt Intervention

CPOE Smart Prompt Activated Via Indication Screens

- INSPIRE prompts triggered by indications for ABD or SST
- If sepsis or empiric selected, a secondary screen is triggered for infection source

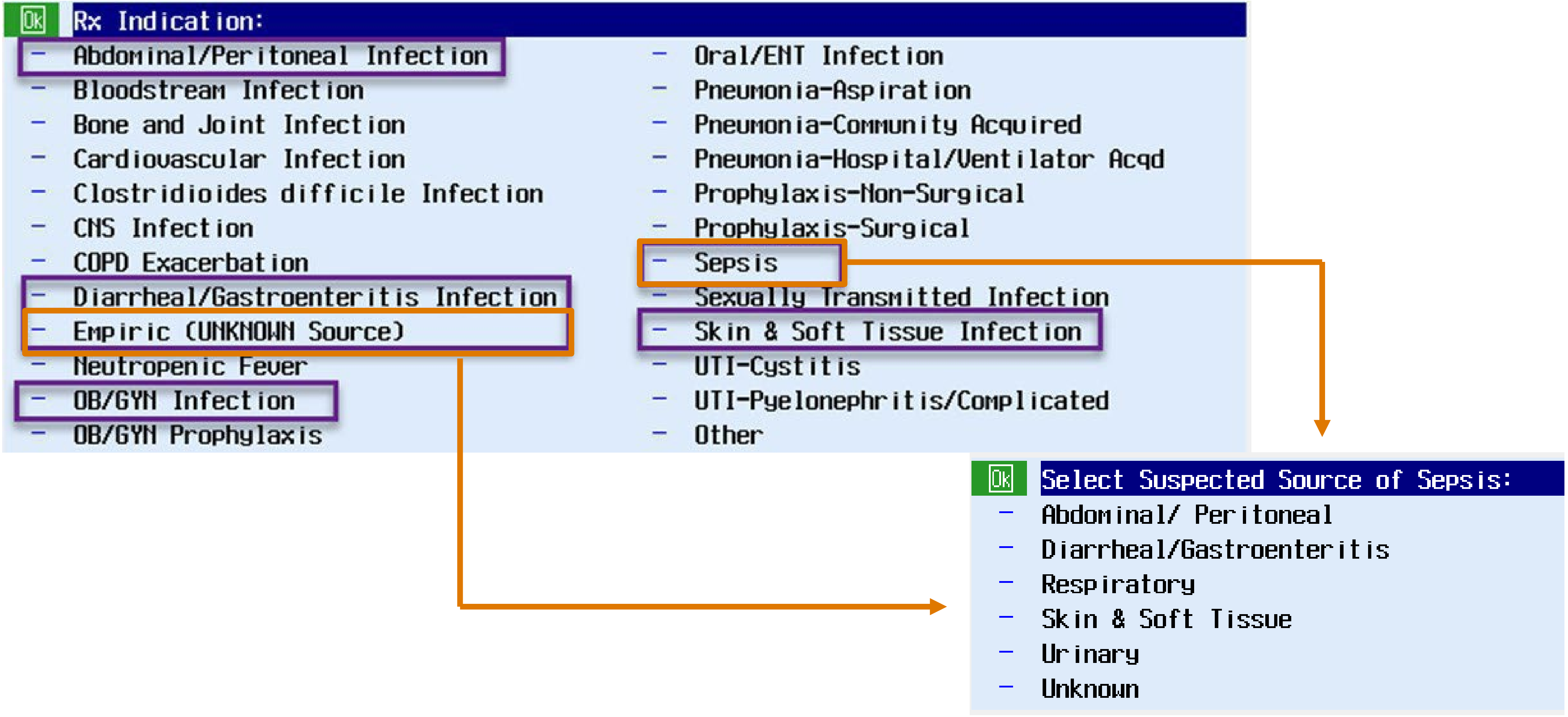

CPOE Smart Prompt Intervention

- **How will it be “smart”?**
  - ✓ Uses ***patient-specific*** EHR data to calculate MDRO risk based on models from 422,000+ HCA patients with ABD or SST
  - ✓ Risk estimate tailored to antibiotic selected. For example:
    - ➢ If ceftriaxone ordered → no prompt (standard-spectrum antibiotic)
    - ➢ If vancomycin ordered → returns MRSA risk
    - ➢ If cefepime ordered → returns *Pseudomonas* risk
    - ➢ If linezolid ordered → returns MRSA and VRE risk
    - ➢ If meropenem ordered → returns ESBL + *Pseudomonas*
  - ✓ If risk $\geq$10%, ES order proceeds without prompts

Excluding ED Patients Going to ICU

- Smart prompt **<u>will not</u>** fire if patient located in ICU
- **For ED patients**, asks if patient is likely to go to the ICU
  - If YES → no further screens
  - If NO → INSPIRE CPOE screens continue

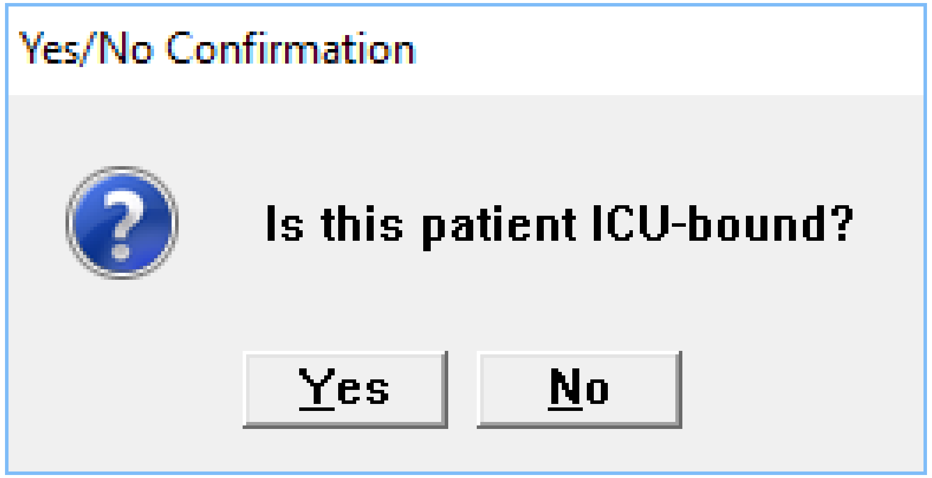

Abdominal Surgery History for Select ES Antibiotics

- INSPIRE risk models – abdominal surgery within past year significantly impacts risk of MRSA and *Pseudomonas* abdominal infection
- **If physician orders anti-MRSA or anti-Pseudomonal for abdominal infection:**

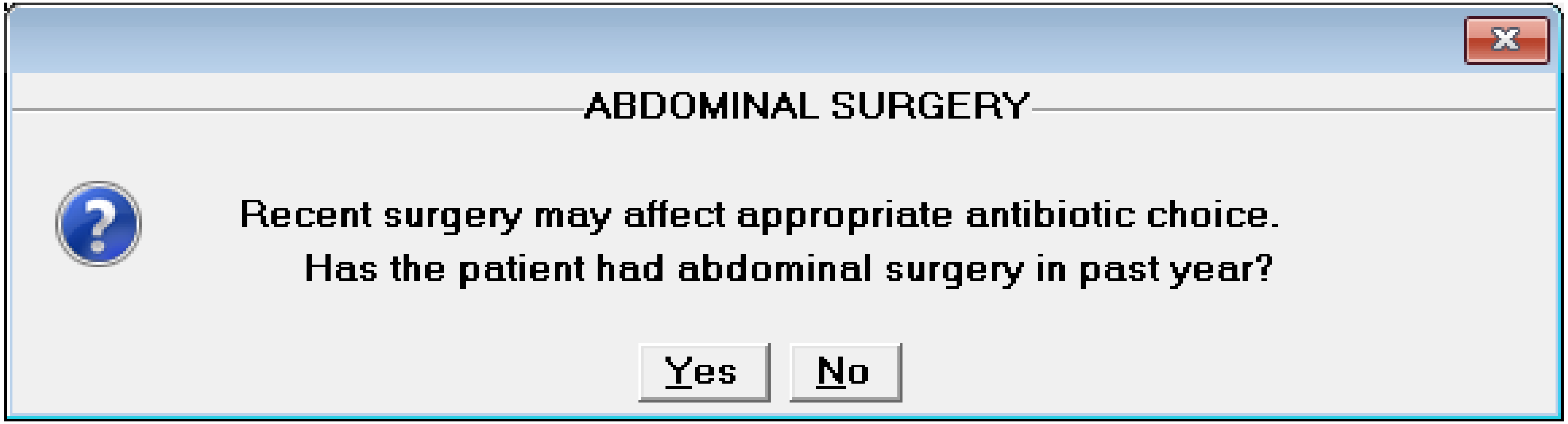

INSPIRE CPOE Smart Prompt Screen - Example

- Physician orders cefepime for SST infection, and
- Risk of *Pseudomonas* SST infection is low (<10%)

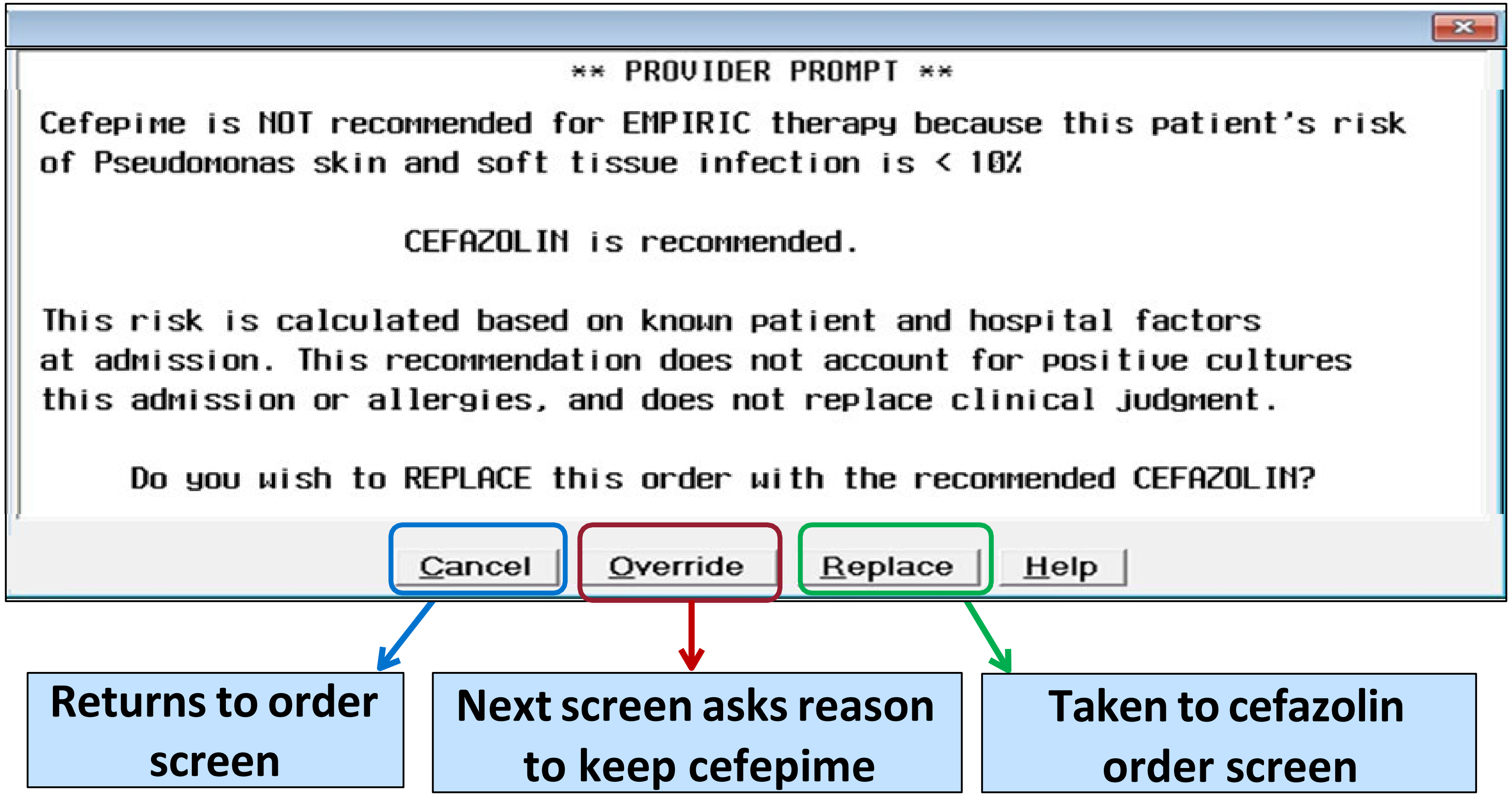

INSPIRE CPOE Smart Prompt Screen - Example

- Physician orders vancomycin for abdominal infection and
- Risk of *MRSA* abdominal infection calculated to be low (<10%)

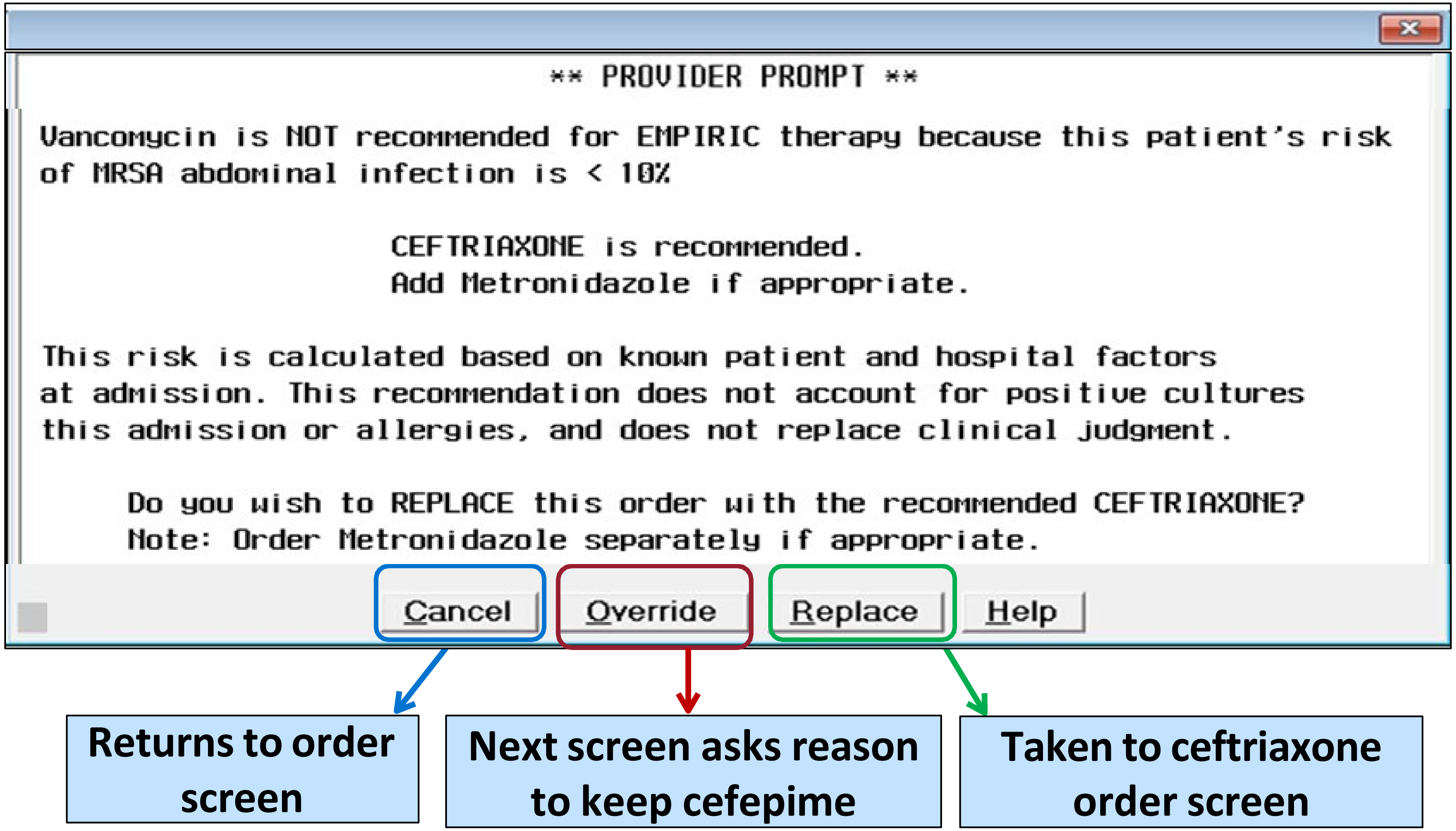

Override Screen and Options

- Clinicians choosing to override will be asked to indicate reason:

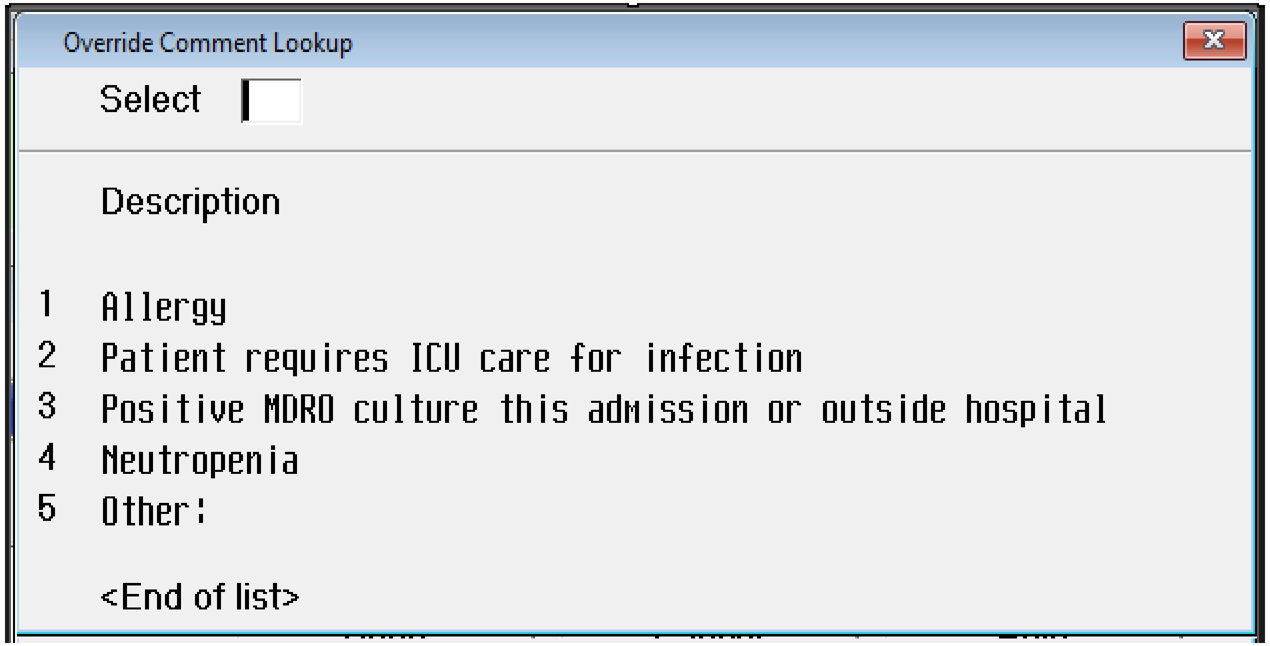

Allergy* ***not* *auto-programmed:* *some allergies missing in EHR, and many in EHR are not true allergies – needs clinical judgment*

**Usual facility allergy prompts continue to appear*

Clinical Judgment Matters

- INSPIRE prompt provides real-time, patient-specific risk estimates to help you choose the best initial antibiotic
- Alert supports, but does not replace clinical judgment
- You may have information not in Meditech that you should consider:
  - Any MDRO cultures this admission or at outside hospital
  - Neutropenia
  - Allergy to recommended antibiotic
  - Imminently headed to ICU
- Adjust antibiotics as needed based on culture results and response to treatment

Will Model Be Validated? What if Local Prevalence Changes?

- INSPIRE models are robust
- Based on 3 years of data across HCA Healthcare
    - Estimates for single facility unlikely to change significantly in 1 year
- Facility prevalence stable during entire baseline evaluation
- Risk estimates re-validated periodically to ensure accuracy

Will Patients Need to Give Informed Consent?

- Informed consent waived by Harvard IRB
- Rationale
  - Comparison of Quality Improvement strategies
  - Patient consent is not required for antibiotic stewardship initiatives
  - Minimal risk

Quarterly Clinician Progress Reports

Principles of Clinical Decision Support

WHO Principles of Clinical Decision Support:

- Speed
- Simplicity
- Usability
- Integration into workflow
- Actionable and provides alternatives
- Evidence/justification provided
- **Impact should be monitored**

Smart prompt provides (Speed through Evidence/justification provided)

CDC: Core Elements of Antibiotic Stewardship

- Prescriber feedback - improves use up to 40%

INSPIRE
BETTER SAFER ANTIBIOTIC USE

Your Antibiotic Stewardship Team Will Provide Active Feedback

Antibiotic use reports will be reviewed and shared with you and hospital leaders

- Clinician-specific
- Department-specific
- Infection-specific

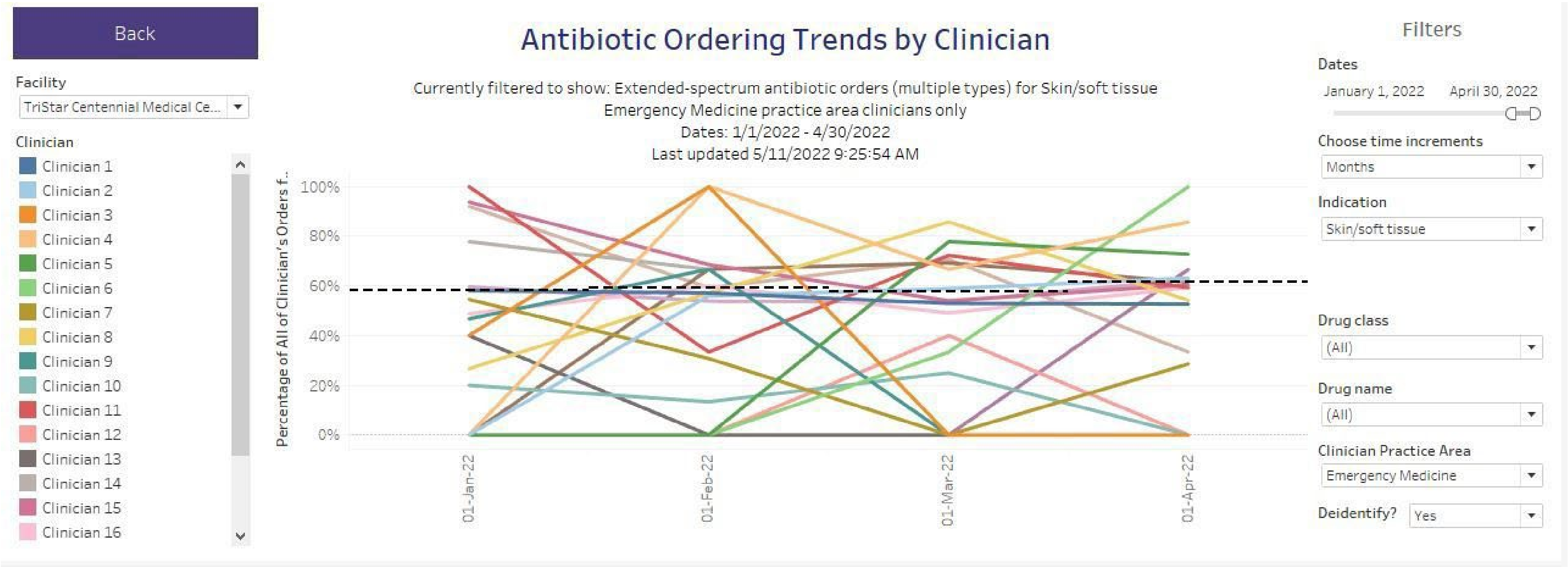

Active Feedback Will Include Reviewing Prompt Response

Response to INSPIRE prompt will be monitored, including

- Prompt acceptance
- Override reasons

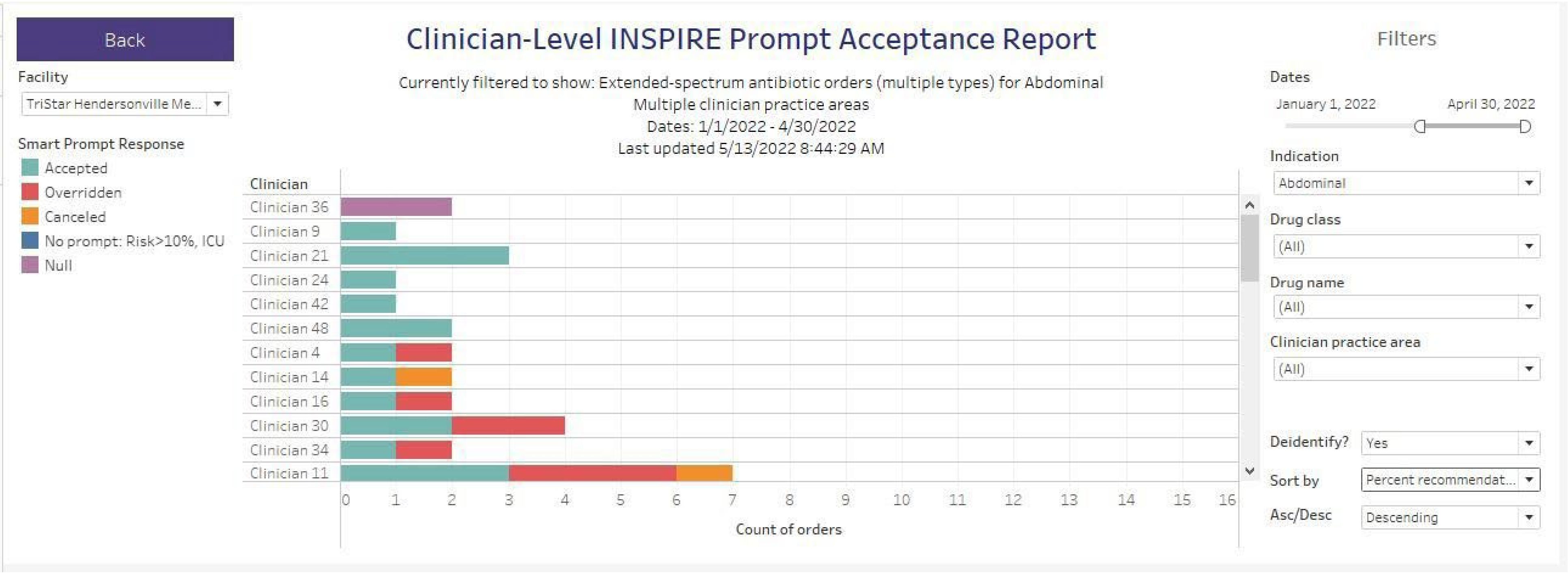

Landmark Trials

- Precision-medicine trials for antibiotic stewardship
- Real-time, patient-specific smart prompts for ABD and SST infections
- Based upon absolute risk, not relative risk
- If successful → best practice for the U.S.

INSPIRE
BETTER SAFER ANTIBIOTIC USE

HCAINSPIRE@gmail.com

INSPIRE Contacts

- **Shruti Gohil, MD, MPH**
  Lead Investigator, HCA INSPIRE Trials
  Phone: (844) 522-4677
  HCAINSPIRE@gmail.com
- **Kaleb Roemer, PharmD**
  Clinical Pharmacy Services Manager, HCA
  Phone: (615) 344-5055
  Email: Kaleb.Roemer@hcahealthcare.com
- **Edward Septimus, MD**
  Harvard-HCA Liaison
  Phone: (281) 714-5689
  Email: Edward.Septimus@hcahealthcare.com
- **Kenneth Sands, MD**
  Chief Epidemiologist, HCA
  Phone: (615) 344-2791
  Email: Kenneth.Sands@hcahealthcare.com

NOTE: Emails and phone numbers approved by HCA Security

Do NOT send PHI to these accounts

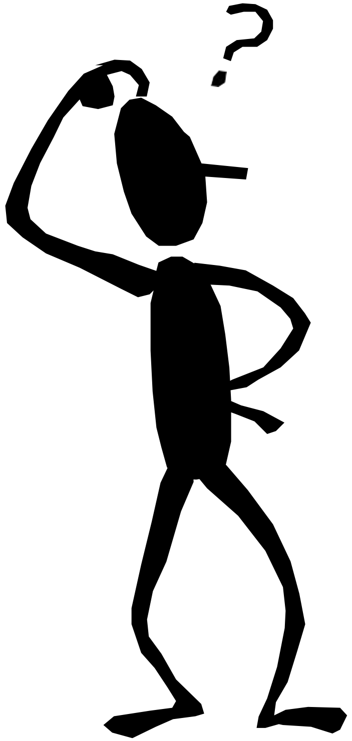

Open Floor Q&A

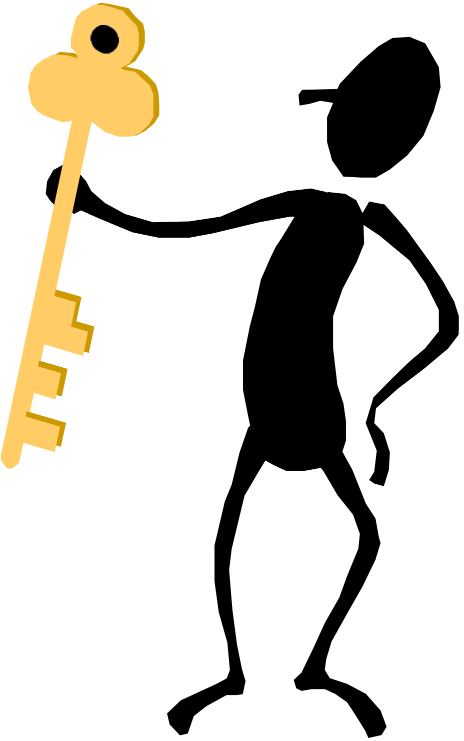

INSPIRE Computerized Physician Order Entry (CPOE) Prompt

Skin & Soft Tissue Infection Workflow

Beginning **August 2, 2022,** our hospital will launch an antibiotic stewardship intervention to **encourage compliance with national guidance for standard-spectrum antibiotic use for non-ICU abdominal and skin/soft tissue (SST) infections.**

Ordering extended-spectrum (ES) antibiotics (e.g., vancomycin, cefepime) for SST infection in the emergency department (ED) or non-ICUs within 3 days of admit will activate a computerized physician order entry (CPOE) smart prompt that will:

- Calculate your patient's risk of infection due to antibiotic resistant bacteria, based upon:
  - Your patient's risk factors available in your hospital's electronic medical record
  - Your hospital's antibiogram
- Recommend standard-spectrum antibiotics if risk is estimated to be low (<10%)

INSPIRE CPOE Workflow: Skin & Soft Tissue Infection Workflow

1. Indications are required for all antibiotics per HCA Healthcare protocol
2. If **an ES antibiotic is ordered for an SST infection indication** (outlined in red) **in a non-ICU patient during the first 3 days of admission, the INSPIRE CPOE prompts will be triggered.**
   a. Note: INSPIRE prompts for pneumonia and urinary tract infection have been active at all HCA facilities since 2021.

Ok	Rx Indication:	
	- Abdominal/Peritoneal Infection	- Oral/ENT Infection
	- Bloodstream Infection	- Pneumonia-Aspiration
	- Bone and Joint Infection	- Pneumonia-Community Acquired
	- Cardiovascular Infection	- Pneumonia-Hospital/Ventilator Acqd
	- Clostridioides difficile Infection	- Prophylaxis-Non-Surgical
	- CNS Infection	- Prophylaxis-Surgical
	- COPD Exacerbation	- Sepsis
	- Diarrheal/Gastroenteritis Infection	- Sexually Transmitted Infection
	- Empiric (UNKNOWN Source)	- Skin & Soft Tissue Infection
	- Neutropenic Fever	- UTI-Cystitis
	- OB/GYN Infection	- UTI-Pyelonephritis/Complicated
	- OB/GYN Prophylaxis	- Other

↓

3. INSPIRE CPOE prompts are programmed to **fire for patients in the ED and non-ICUs only** and will **NOT** fire if a patient is in an ICU location:

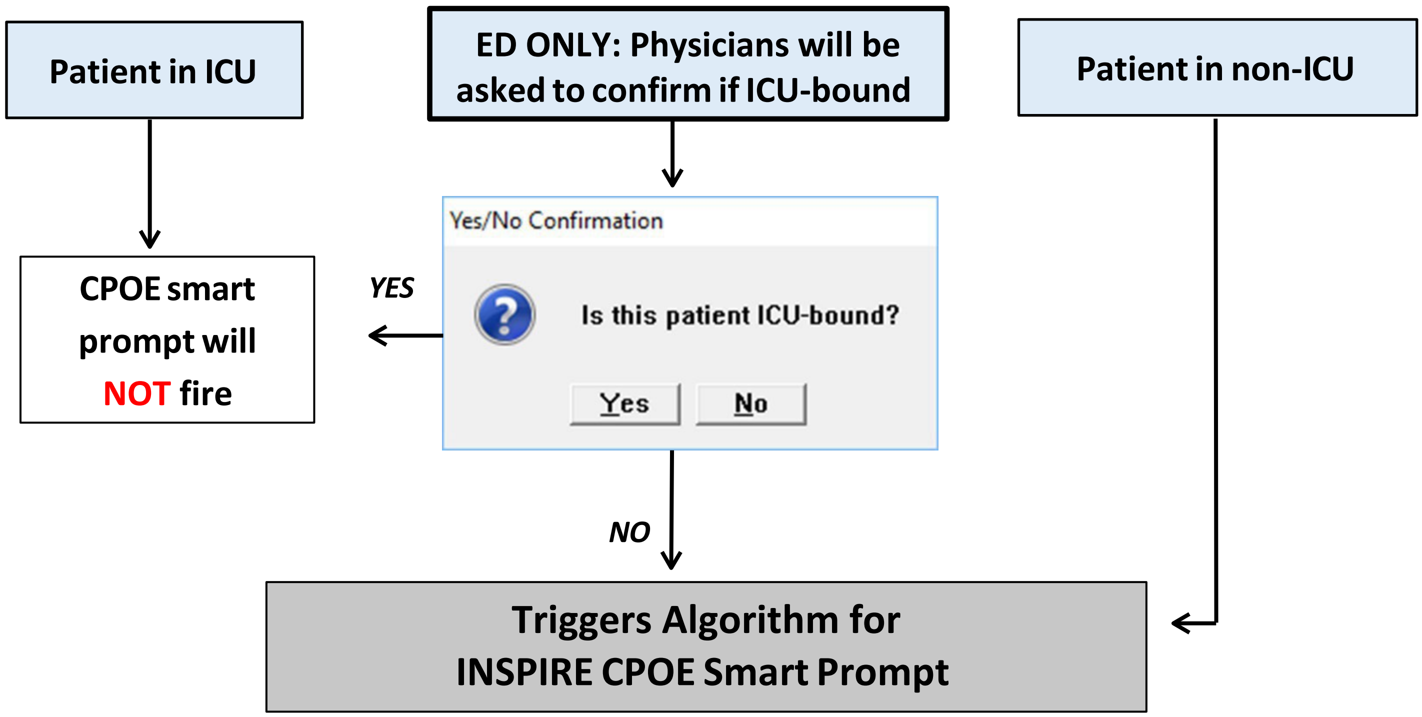

4. INSPIRE CPOE prompts will automatically assess your **patient's individual risk for antibiotic resistant skin & soft tissue infection using data available in your hospital's electronic medical record**. This risk estimate is tailored to the antibiotic ordered.
   a. For example:
      i. If vancomycin ordered → returns MRSA risk
      ii. If cefepime ordered → returns *Pseudomonas* risk
      iii. If linezolid ordered → returns MRSA and VRE risk
      iv. If meropenem ordered → returns ESBL + *Pseudomonas* risk

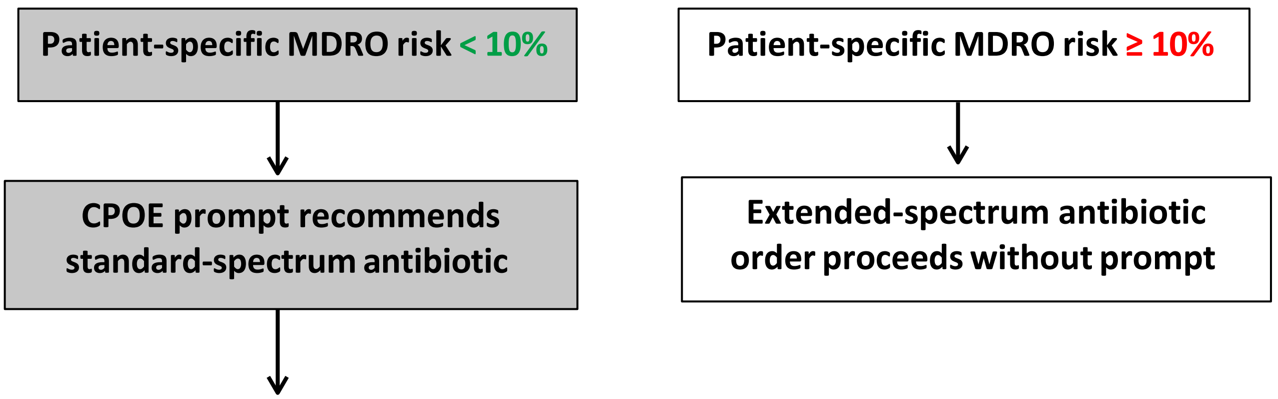

(See following page for mock INSPIRE CPOE smart prompt)

Mock CPOE Smart Prompt Recommendation Screen

Example INSPIRE Skin & Soft Tissue Infection Smart Prompt:

Scenario: Physician orders cefepime for skin & soft tissue infection.
Patient risk for *Pseudomonal* skin & soft tissue infection is calculated to be <10%.

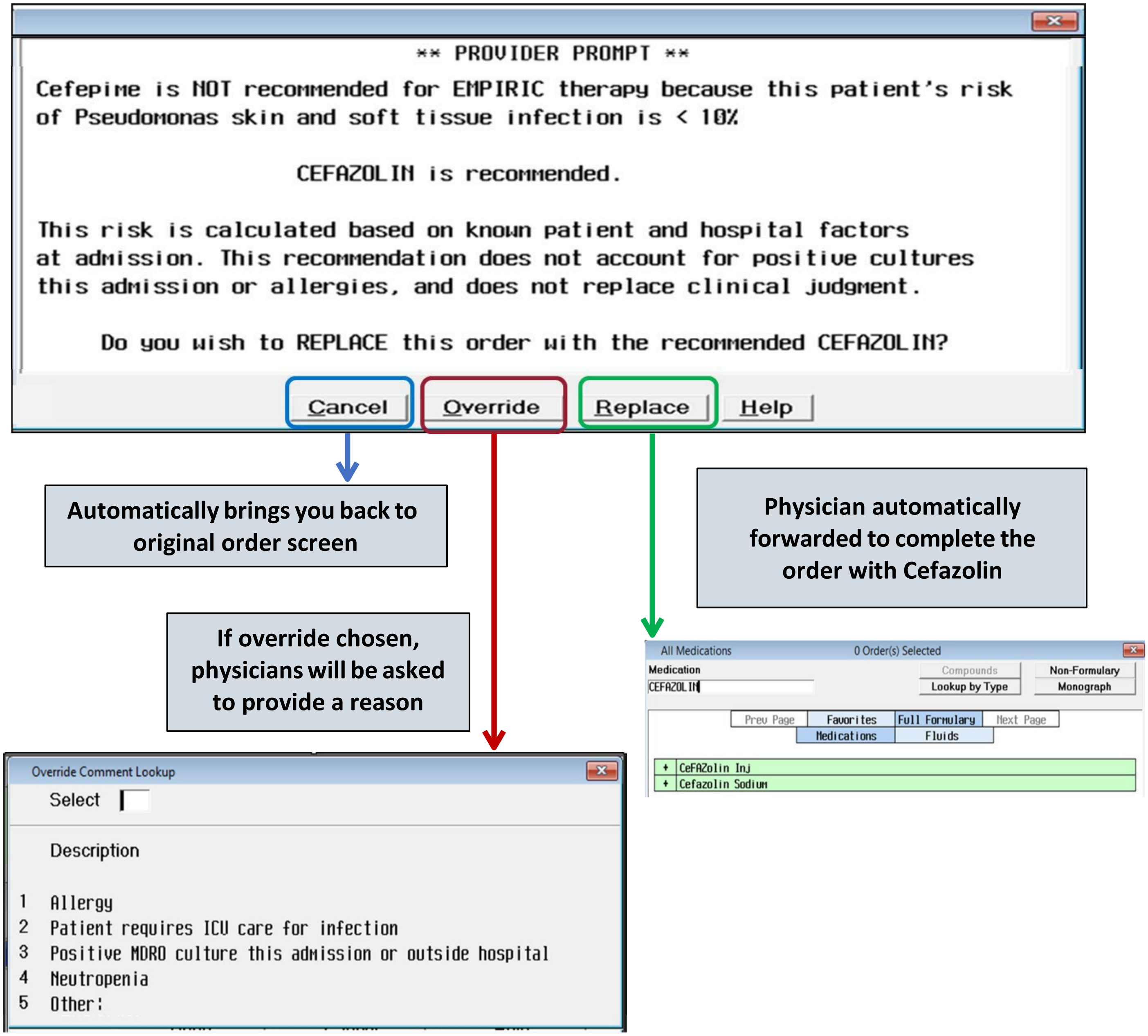

INSPIRE Computerized Physician Order Entry (CPOE) Prompt
Abdominal Infection Workflow

Beginning **August 2, 2022,** our hospital will launch an antibiotic stewardship intervention to **encourage compliance with national guidance for standard-spectrum antibiotic use for non-ICU abdominal and skin/soft tissue (SST) infections.**

Ordering extended-spectrum (ES) antibiotics (e.g., vancomycin, cefepime) for abdominal infections in the emergency department (ED) or non-ICUs within 3 days of admit will activate a computerized physician order entry (CPOE) smart prompt that will:

- Calculate your patient's risk of infection due to antibiotic resistant bacteria, based upon:
    - Your patient's risk factors available in your hospital's electronic medical record
    - Your hospital's antibiogram
- Recommend standard-spectrum antibiotics if risk is estimated to be low (<10%)

INSPIRE CPOE Workflow: Abdominal Infection Workflow

1. Indications are required for all antibiotics per HCA Healthcare protocol
2. If **an ES antibiotic is ordered for an SST infection indication** (outlined in red) **in a non-ICU patient during the first 3 days of admission, the INSPIRE CPOE prompts will be triggered.**
    a. Note: INSPIRE prompts for pneumonia and urinary tract infection have been active at all HCA facilities since 2021.

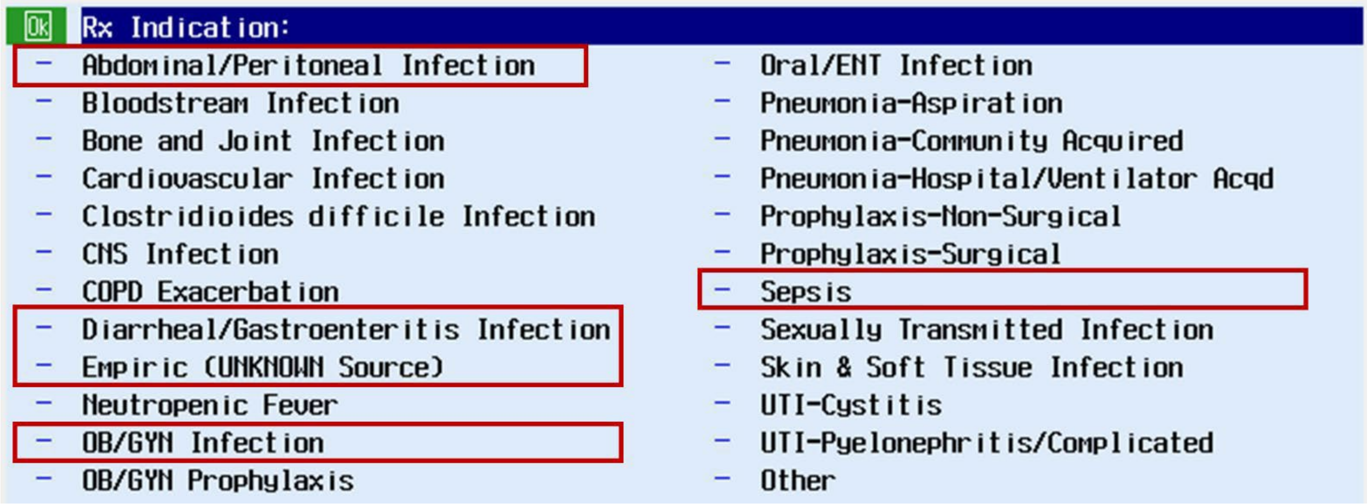

↓

3. INSPIRE CPOE prompts are programmed to **fire for patients in the ED and non-ICU units only** and will **NOT** fire if a patient is in an ICU location:

Patient in ICU

ED ONLY: Physicians will be asked to confirm if ICU-bound

Patient in non-ICU

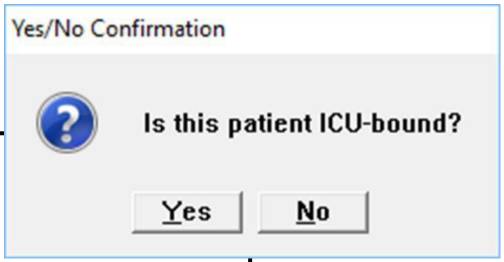

YES

CPOE smart prompt will NOT fire

NO

Triggers Algorithm for INSPIRE CPOE Smart Prompt

4. INSPIRE CPOE prompts will automatically assess your **patient's individual risk for antibiotic resistant abdominal infection using data available in your hospital's electronic medical record**. This risk estimate is tailored to the antibiotic ordered.
   a. For example:
      i. If vancomycin ordered → returns MRSA risk
      ii. If cefepime ordered → returns *Pseudomonas* risk
      iii. If linezolid ordered → returns MRSA and VRE risk
      iv. If meropenem ordered → returns ESBL + *Pseudomonas* risk

Patient-specific MDRO risk < 10%

CPOE prompt recommends standard-spectrum antibiotic

(See following page for example INSPIRE CPOE smart prompt)

Patient-specific MDRO risk ≥ 10%

Extended-spectrum antibiotic order proceeds without prompt

5. **For Anti-MRSA and antipseudomonal antibiotics only**:
   a. INSPIRE risk estimate models found that abdominal surgery within past year significantly impacts risk of MRSA and *Pseudomonas* abdominal infection.
   b. If the ordered extended-spectrum antibiotic targets these pathogens, then an additional screen will ask about surgical history before the final prompt recommendation screen:

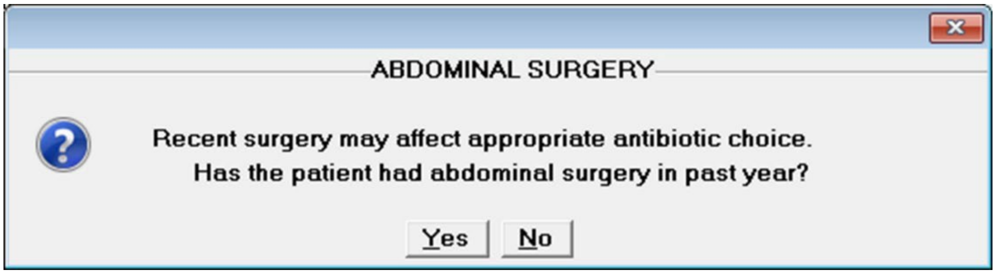

Mock CPOE Smart Prompt Recommendation Screen

<u>Example INSPIRE Abdominal Infection Smart Prompt</u>:
Scenario: Physician orders Vancomycin for abdominal infection.
Patient risk for *MRSA* abdominal infection is calculated to be <10%.

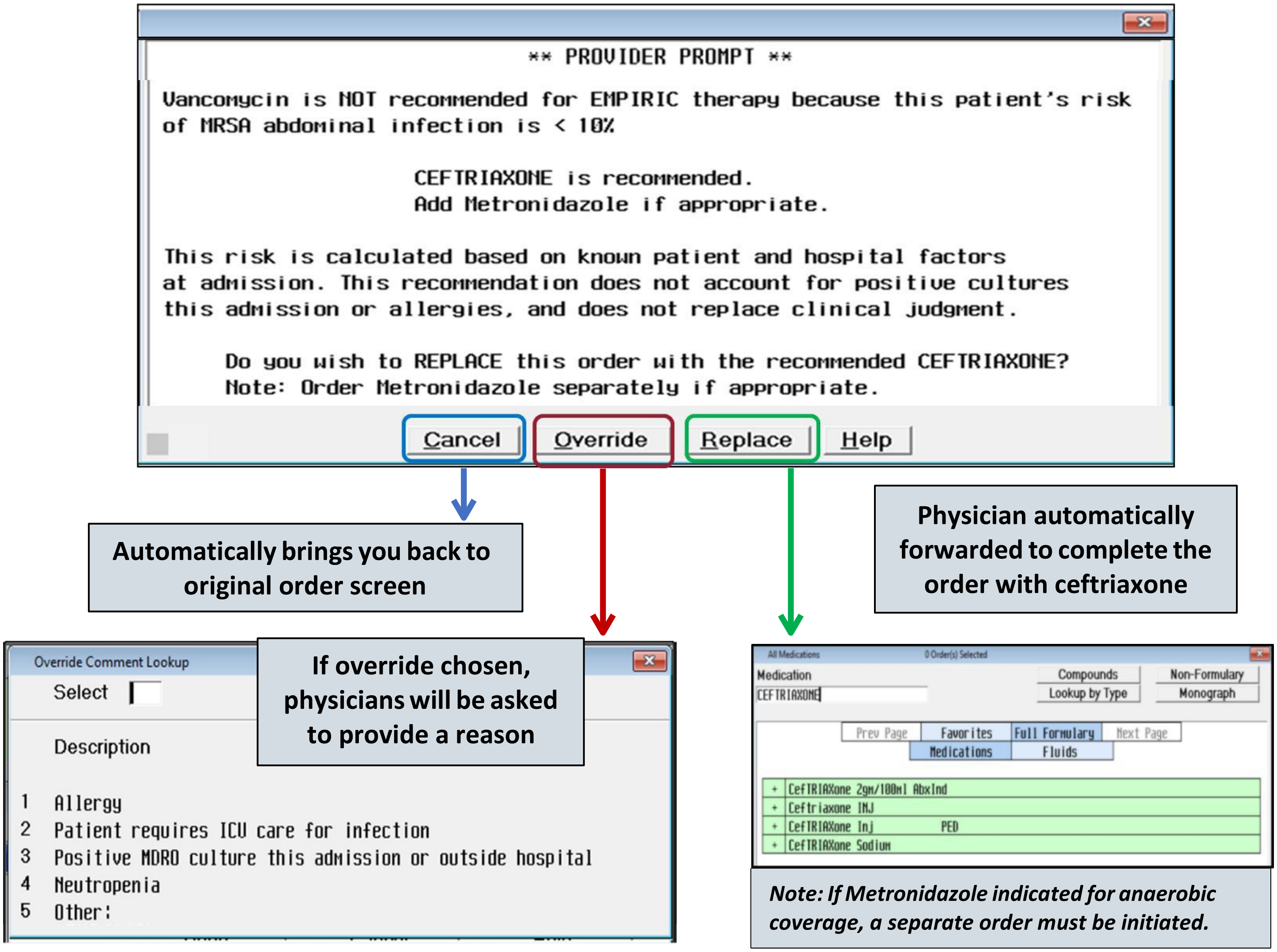

Promoting INSPIRE Trials at Your Facility

How can Study Champions increase clinician awareness of INSPIRE prompts?

It is important that clinicians understand that your hospital is a proud participant in the INSPIRE trials and that the INSPIRE smart prompts are intended to provide real-time, patient specific risk estimates to guide appropriate antibiotic ordering for abdominal and skin/soft tissue infections.

In addition to the "Clinician Education" materials in this Toolkit, we have provided the following items to help promote INSPIRE at your facility:

- **Poster Clings** – this self-adhesive poster is designed for immediate posting in clinicians work/lounge areas. These clings are compliant with regulatory standards; they do not require tape and will not leave sticky residue behind when removed. Study champions should hang these in communal areas frequented by clinicians, such as:
    - Clinician lounges
    - Workrooms – ED/hospitalist charting and/or rounding rooms
    - Operating room lounges
- **CPOE Workflow Laminates** – this series of key ring bound laminates is intended to be placed in centralized workstations where active ordering occurs.

You know your facility best. Study Champions should plan to adapt the materials we have provided and use them to best address your facility's needs. Successful strategies used by facilities in the past include:

- Computer screensavers – announcing INSPIRE or judicious antibiotic use messaging
- QR-code based links to educational documents
- Poster boards
- Presentations
- Emails

Funded by National Institute of Allergy and Infectious Diseases
